# Design of Phosphine-Heteroarenesulfonamide Ligands as Dinuclear Silver Catalysts for Enantioselective Construction of α,β-Diamino Acids

**DOI:** 10.1021/jacs.5c18426

**Published:** 2025-12-11

**Authors:** Yuka Iizuka, Sayuri Okajima, Yamato Ueno, Tsunayoshi Takehara, Takeyuki Suzuki, Satoshi Maeda, Shuichi Nakamura

**Affiliations:** † Department of Life Science and Applied Chemistry, Graduate School of Engineering, 12982 Nagoya Institute of Technology, Gokiso, Showa-ku, Nagoya 466-8555, Japan; ‡ Department of Chemistry, Faculty of Science, 12810 Hokkaido University, Kita 10 Nishi 8, Kita-ku, Sapporo, Hokkaido 060-0810, Japan; § The Institute of Scientific and Industrial Research, 13013 Osaka University, 8-1 Mihogaoka, Ibaraki-shi, Osaka 567-0047, Japan; ∥ Institute for Chemical Reaction Design and Discovery (WPI-ICReDD), Hokkaido University, Kita 21 Nishi 10, Kita-ku, Sapporo, Hokkaido 001-0021, Japan; ⊥ JST, ERATO Maeda Artificial Intelligence in Chemical Reaction Design and Discovery Project, Kita 10 Nishi 8, Kita-ku, Sapporo, Hokkaido 060-0810, Japan

## Abstract

We designed and developed a phosphine–heteroarenesulfonamide ligand and found that it functions as a chiral dinuclear silver catalyst capable of cooperatively activating both nucleophiles and electrophiles. As a result, a highly enantioselective Mannich-type reaction between glycinate Schiff bases and acyclic ketiminoesters, unattainable by mononuclear catalytic systems, was achieved, affording α,β-diamino acid derivatives bearing tetrasubstituted chiral carbon centers in excellent yields and enantioselectivities. The resulting α,β-diamino acid derivatives were readily transformed into optically active 2-imidazolidinone and a dipeptide. Comprehensive mechanistic studies combining global reaction route mapping (GRRM), artificial force-induced reaction (AFIR), and density functional theory calculations revealed a cooperative bimetallic transition-state architecture responsible for the observed stereocontrol. These findings establish a new design principle for asymmetric catalysis based on homodinuclear activation and highlight the potential of dinuclear silver catalysis for the construction of complex chiral molecules.

## Graphical abstract

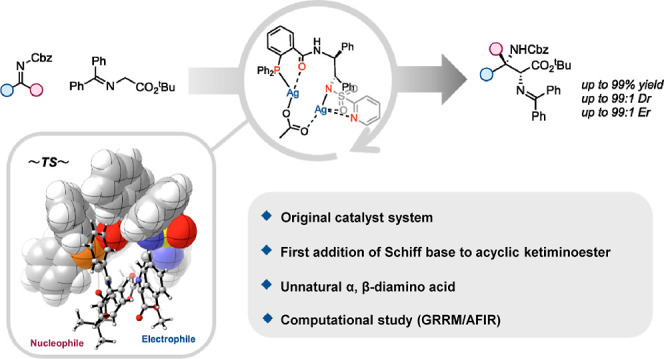

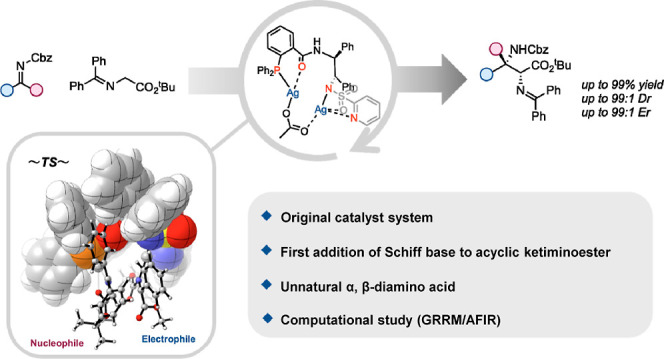

## Introduction

The design of new chiral catalytic systems is important for the efficient and stereoselective synthesis of chiral natural products, pharmaceuticals, and agrochemicals that are challenging to synthesize. Among previously developed chiral catalysts, chiral phosphine ligands, in particular, have enabled a broad range of asymmetric syntheses. Incorporation of the 2-(diphenylphosphino)­benzoic acid framework into a chiral diamine backbone has therefore become an important strategy in chiral ligand design. For example, Trost ligands^1^ and Dixon ligands,^2^ both of which adopt this framework, are highly valued as extremely versatile chiral ligands for single-metal catalysis and have been widely applied in asymmetric and total synthesis (Figure 1A,B).^3^ More recently, catalysts comprising a chiral diamine bearing a 2-(diphenylphosphino)­carbonyl group together with organocatalytic activation sites (e.g., urea, thiourea, or squaramide) have been extensively studied, enabling simultaneous activation through metal coordination and hydrogen bonding; however, these systems are fundamentally based on mononuclear catalysis (Figure 1C).^4^ To move beyond the traditional mononuclear paradigm, we envisioned that a chiral ligand capable of binding two metal centers cooperatively could open a new dimension in asymmetric catalysis. We recently reported the efficient activation and asymmetric reactions of acyclic ketiminoesters using our originally designed ligand bearing a heteroarenesulfonamide group. In this system, the Lewis acidity of the metal center enhances the reactivity of electrophiles such as acyclic ketimines, while the heteroarylamide moiety contributes to the formation of an advanced chiral pocket by strongly coordinating to the metal center.^5^ Based on these findings, we designed a chiral ligand incorporating a heteroarenesulfonamide group into a chiral diamine–phosphine framework as a novel catalyst to enable asymmetric reactions that have remained difficult to achieve (Figure 1D). We hypothesized that this chiral ligand could coordinate a dinuclear complex catalyst in which one metal binds to the phosphine moiety and the other to the heteroarenesulfonamide group, thereby strongly activating nucleophiles and electrophiles at each metal center. Such chiral dinuclear catalysts are recognized as powerful tools for achieving high efficiency and selectivity in asymmetric reactions,^6^ and we selected silver acetate as a Lewis acid that can simultaneously activate both nucleophiles and electrophiles.^7^ To verify this concept, a preliminary conformational search was performed using the single-component artificial force induced reaction (SC-AFIR) method.^8,9^ Interestingly, it was predicted that the designed ligand coordinates two silver centers bridged by an acetate anion, with the nucleophile and electrophile appropriately positioned, suggesting that each metal center can strongly activate the respective substrate (Figure 1E). Furthermore, computational analysis of the dinuclear catalyst using 3D structural and steric mapping indicated that the phosphine and pyridinesulfonyl groups of the ligand formed an appropriate asymmetric reaction site by covering the metal center (Figure 1F, where the percentage of buried volume is 62.2). From these preliminary conformational studies, the designed 2-pyridinesulfonylated diamine phosphine ligand was expected to create a highly favorable asymmetric environment by accommodating the reactive substrate within the formed chiral pocket.

**Figure 1:**
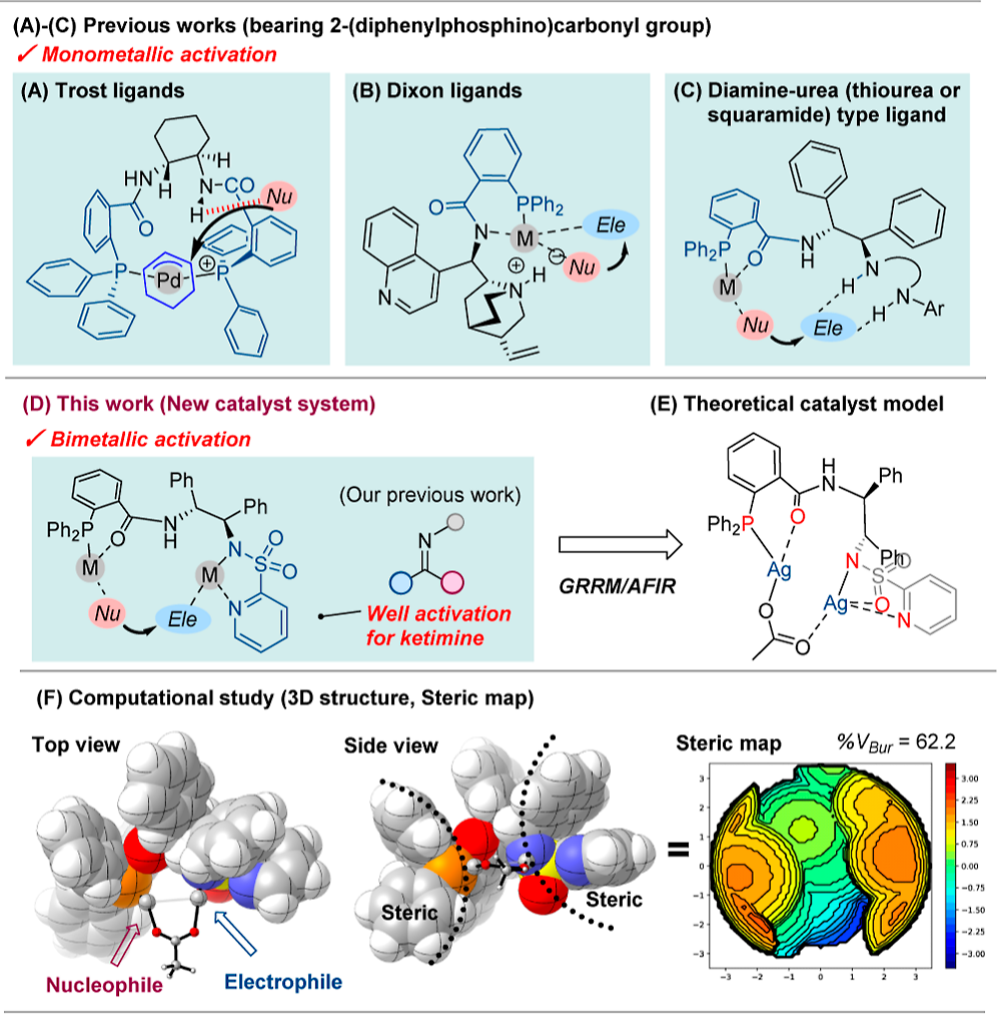
Chiral ligands using 2-(diphenylphosphino)­benzoic acid.

Building upon this ligand design, we sought to apply the dinuclear catalytic system to a synthetically significant target class, the asymmetric construction of unnatural α-amino acids. These compounds, particularly those bearing tetrasubstituted chiral carbon centers, have attracted considerable attention as key structural motifs in modern medicinal chemistry, and asymmetric nucleophilic additions to ketiminoesters have emerged as a powerful strategy for their synthesis. However, asymmetric syntheses involving acyclic ketimines to obtain α-amino acids with tetrasubstituted chiral carbons remain challenging owing to their low reactivity and severe steric hindrance.^10,11^ Among such α-amino acids, chiral α,β-diamino acids bearing tetrasubstituted chiral carbon centers are key structural motifs widely found in bioactive natural products, chiral ligands, and catalysts, and they serve as highly useful and versatile synthetic building blocks in organic synthesis;^12,13^ however, their synthetic methods remain limited. Enantioselective Mannich-type reactions of glycinate Schiff base nucleophiles with imines represent a promising route to enantioenriched α,β-diamino acid cores containing contiguous stereocenters.^4a,14,15^ From this perspective, reactions between ketiminoesters and glycinate Schiff base nucleophiles can furnish intriguing amino acid derivatives in which the amino acid skeletons are directly connected through a carbon atom.^16^ Recently, Xie and Deng independently reported the construction of tetrasubstituted chiral carbons with high stereoselectivity through asymmetric nucleophilic addition of glycinate Schiff bases to cyclic ketiminoesters derived from saccharin and isatin using copper/phosphinooxazoline (PHOX)-type catalysts, respectively.^15a,15b^ Wu and co-workers reported a similar reaction using N,P-ligands bearing an amide moiety, enabling bifunctional activation through enolate-metal/Brønsted acid dual activation.^15c^ Despite these advances, no reports have described the synthesis of precursors of acyclic α,β-diamino acids and α,β-diamino-α,β-dicarboxylic acids using acyclic ketimines (Figure 2A).^15d^ To address this long-standing challenge and demonstrate the effectiveness of our newly designed dinuclear complex catalyst incorporating a heteroaryl unit, we developed a highly diastereo- and enantioselective synthesis of α,β-diamino acid derivatives bearing tetrasubstituted carbon centers (Figure 2B). This work establishes a general design framework for bimetallic asymmetric catalysis based on easily modifiable diamine–phosphine scaffolds, providing a new strategy for constructing complex chiral molecules through cooperative homodinuclear activation.

**Figure 2:**
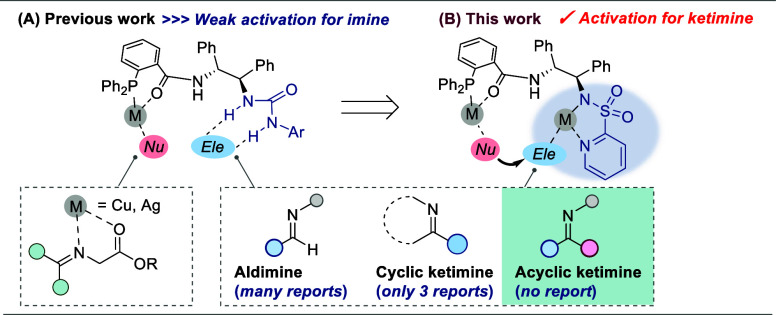
Asymmetric Mannich-type reaction of glycinate Schiff bases to ketimines.

## Results and Discussion

### Reaction Optimization

Initially, α-ketiminoester **1a** (1.1 equiv) and glycinate Schiff bases **2** were selected as model substrates to screen for suitable chiral diamine-based N,P-ligands. The reaction was performed with 10 mol % AgOAc, 5 mol % chiral ligand, and 50 mol % K_2_CO_3_ as the base in THF at 0 °C (Table 1). Based on our preliminary conformational search, the chiral ligand **A**, which forms a dinuclear complex, provided excellent results, affording product **3a** in 99% yield with 99:1 dr and 98:2 er. Although ligands **B** and **C** contain different heteroarylamide groups, they afforded only moderate yields and showed poor enantioselectivity. Furthermore, ligand **D** (lacking a heteroaryl moiety), ligand **E** (without the phosphine moiety), and ligand **F** (phosphine replaced with an amine) all exhibited low enantioselectivity. Similarly, ligand **G**, in which the N–H in the sulfonamide functionality was protected by a methyl group, and ligand **H**, in which the carbonyl group was changed to an imine form, were ineffective under these conditions. These results indicate that both the heteroarenesulfonamide and phosphine moieties of the catalyst are crucial in controlling the stereoselectivity of the reaction. Replacement of the Ph substituent on the diamine backbone with Mes groups (ligand **I**) afforded the product with low enantioselectivity. A previously reported phosphine–urea ligand **J**, which was employed in the addition of Schiff bases to aldimines,^4a^ was also tested but gave low yield and enantioselectivity, confirming the superiority of our newly designed chiral ligand. Furthermore, the use of cinchona alkaloid-derived ligand **K**, previously developed by our group and bearing a heteroarylamide group, resulted in low enantioselectivity.

**Table 1:**
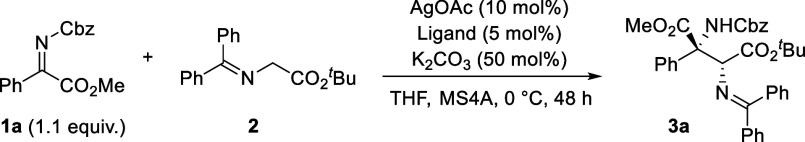

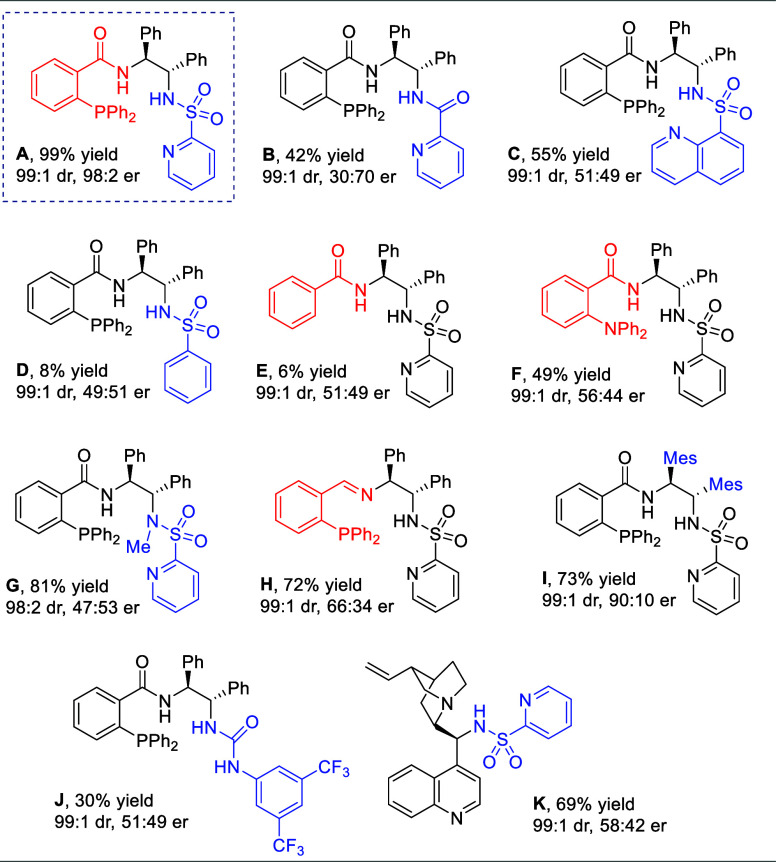
Optimization of Dinuclear Catalysts Using Phosphine-Heteroarylamide Ligands^a^

a Yield determined by ^1^H NMR using 1,3,5-trimethoxybenzene as an internal standard.

Subsequently, we examined other reaction parameters using ligand **A** (Table 2). Reducing the amount of silver acetate to 5 mol % and adjusting the silver-to-ligand ratio to 1:1 resulted in a considerably lower yield and slightly reduced enantioselectivity (Table 2, entry 2). This reaction proceeded in the absence of a ligand using only the silver salt (Table 2, entry 3). Conversely, when only the ligand was added, the reaction scarcely proceeded and showed no stereoselectivity (Table 2, entry 4). Under the optimal conditions (Table 2, entry 1), if the dinuclear silver complex was not formed, unbound silver acetate would remain in the reaction system, which should lead to a decrease in stereoselectivity; however, in practice, no loss of selectivity was observed. Thus, the contrasting outcomes of entries 1–4 strongly suggest that a dinuclear silver complex serves as the catalytically active species, a conclusion that is corroborated by subsequent spectroscopic and theoretical investigations (see the Mechanistic Study section). Because copper salts are often used for the activation of Schiff bases,^4a,4c,14,15^ we examined copper acetate instead of silver acetate; however, the reaction was inefficient (Table 2, entry 5). Because the ionic radius of Cu^+^ is significantly smaller than that of Ag^+^, steric repulsion between the chiral ligand and the substrate is likely to destabilize the Cu-based complex, preventing effective dual activation. Replacing K_2_CO_3_ with triethylamine (TEA) as the base also gave a markedly lower yield (Table 2, entry 6). Since the yield was significantly reduced without the base (Table 2, entry 7), these results indicate that the base is essential for deprotonation of the Schiff base nucleophile. In addition, K_2_CO_3_ remains undissolved in the reaction mixture and gradually dissolves as the reaction proceeds. It was also confirmed that reducing the amount of K_2_CO_3_ to below 50 mol % results in a significant decrease in yield (see the Supporting Information).

**Table 2:**
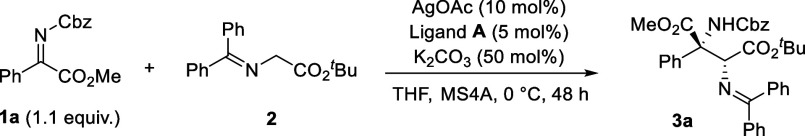
Deviations from Standard Conditions

entry	deviations from the standard conditions	yield (%)^a^	Dr	Er
1	None	99	99:1	98:2
2	AgOAc (5 mol %) was used	41	99:1	95:5
3	without Ligand	56	99:1	50:50
4	without AgOAc	8	99:1	51:49
5	CuOAc was used instead of AgOAc	2	99:1	44:56
6	TEA was used instead of K_2_CO_3_	29	99:1	82:18
7	without K_2_CO_3_	37	99:1	95:5

a Yield determined by ^1^H NMR using 1,3,5-trimethoxybenzene as an internal standard.

### Substrate Scope

With the optimal catalytic system established and the reaction mechanism elucidated, we next investigated the substrate scope of ketimines **1** in a Mannich-type reaction with *tert*-butyl glycinate Schiff base **2** (Table 3). Ketiminoesters bearing both electron-deficient and electron-rich substituents on the phenyl ring (**1a**–**i**) gave products (**3a**–**i**) in excellent yields and stereoselectivities (99:1 dr, 96:4–98:2 er). Replacing the phenyl group with a 2-naphthyl group (**1j**) or a heteroaryl group (**1k** and **1l**) afforded products **3j**–**l** in high yield with high enantioselectivities. Alkynyl ketiminoesters (**1m**–**o**) furnished the desired products (**3m**–**o**) with good enantioselectivity. Modification of the ester group in the ketiminoesters (**1p**–**r**) also afforded the corresponding products (**3p**–**r**) with excellent yields and enantioselectivities. Furthermore, iminonitrile (**1s**) underwent the reaction smoothly, providing **3s** with a 94:6 er. Finally, isatinketimine (**1t**) and aldimine (**1u**) were also tolerated in the reaction, although with low diastereoselectivity. The absolute configuration of products was determined to be (2*S*,3*R*) by X-ray crystallographic analysis of an *N*-bromobenzoylated derivative of the α,β-amino acid obtained by treatment of compound **3c** with 1 M HCl, and the configurations of the other products were assigned by analogy (see the Supporting Information)

**Table 3:**
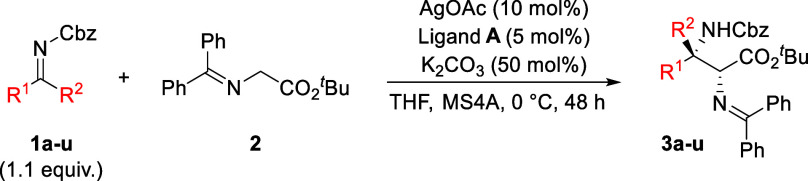

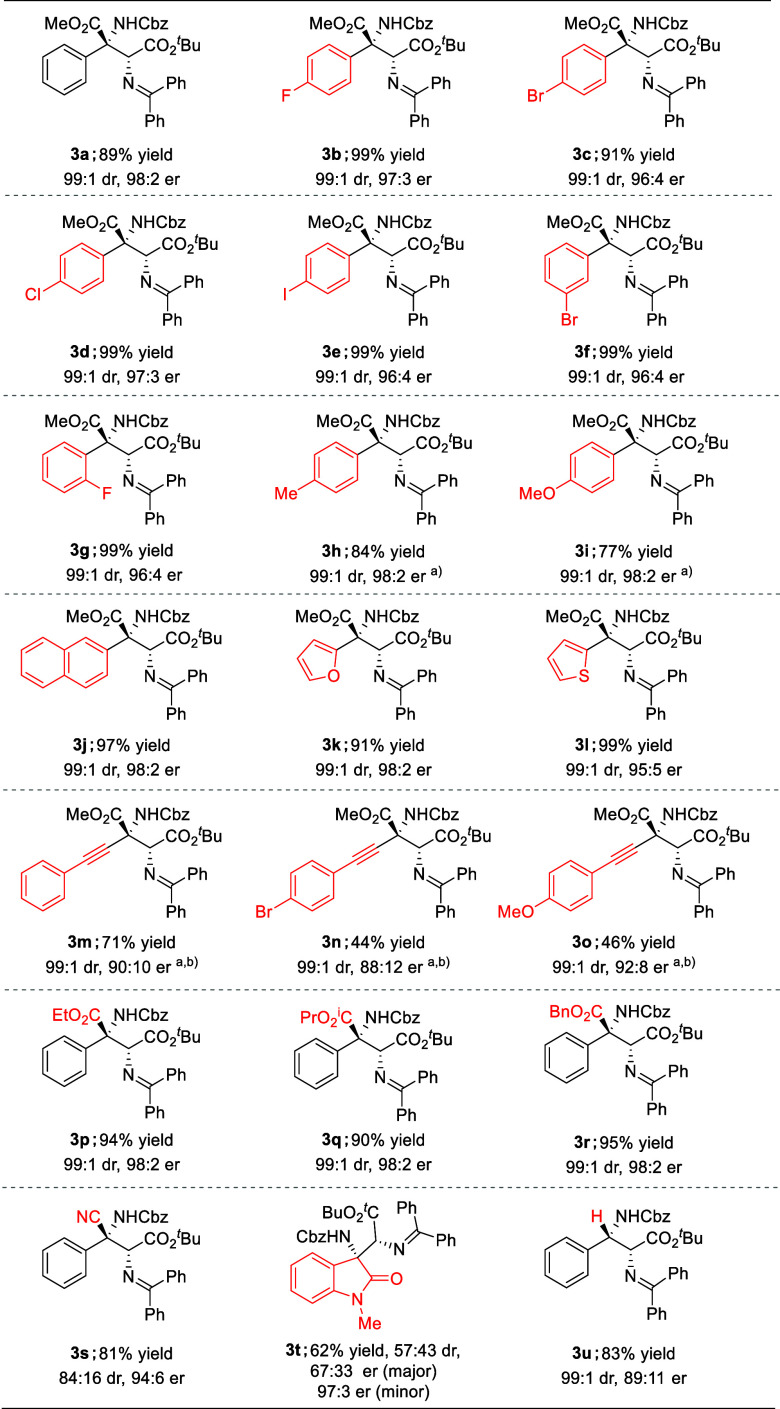
Substrate Scope of the Enantioselective Mannich-Type Reaction of the Schiff Base

a Ligand (10 mol %) and AgOAc (20 mol %) were used.

b At – 20 °C.

### Product Transformations

The synthetic utility of this protocol was further investigated (Scheme 1). The reaction between **1a** and **2** was performed on a gram scale, affording compound **3a** in an 83% yield, 99:1 dr, and 96% ee (Scheme 1A). Compound **3a** was subsequently converted to several useful derivatives, including 2-imidazolidinone **4**, dipeptide **5**, and α,β-diamino acid **6** with fewer operations (Scheme 1B). Specifically, acid hydrolysis of **3a** afforded compound **4** (99% yield, 99:1 dr, 96% ee), which was then transformed to cyclic urea with triphosgene. Condensation of a α,β-diamino acid derivative from **3a** with Boc-protected phenylalanine afforded the corresponding dipeptide **5** in a moderate yield without epimerization. Furthermore, the reaction of **3r** with Pd/C under a hydrogen atmosphere simultaneously removed the Cbz and Bn groups, affording the unprotected amino acid **6** in excellent yield with good stereoselectivity.

**Scheme 1:**
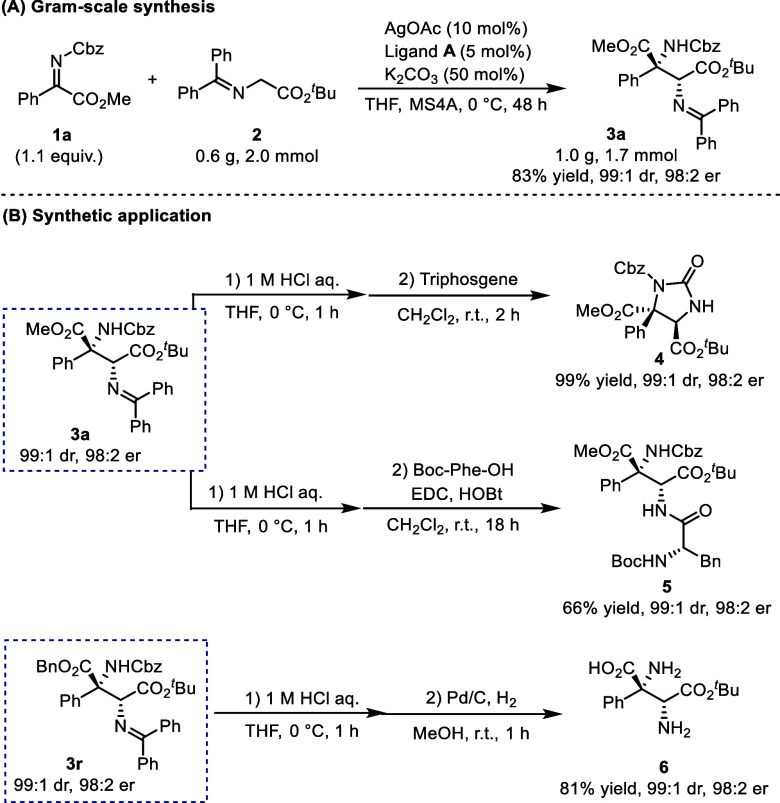
Gram-Scale Mannich-Type Reaction and Synthetic Application

### Mechanistic Study

To gain insight into the stereocontrol mechanism of this chiral dinuclear catalyst, density functional theory (DFT) calculations were performed (Figure 3). Equilibrium structures (EQs), including Ag-chiral ligand conformations and Ag/ligand-substrate structures, were systematically explored using the AFIR methodology at the GFN2-xTB level of theory (ORCA 4.2.0).^17^ Geometries were then optimized at the B3LYP/def2-SV­(P)/CPCM­(THF) level of theory (Gaussian16), followed by single-point energy calculations at M06-D3/def2-TZVPP/CPCM­(THF).^18^ The results for the energy diagram of the reaction are shown in Figure 3A. Consistent with Figure 1, in the conformation of the dinuclear complex calculated using SC-AFIR, the most stable structure featured one silver atom coordinated to the phosphine carbonyl oxygen, while the other coordinated via deprotonation of the 2-pyridinesulfonamide moiety, with the acetate anion bridging both silver centers. Supporting this model was ESI-MS analysis of a THF solution of ligand **A** with 2 equiv of AgOAc detected **Int-1** (**Int-1** ^+^ calcd. for C_38_H_31_N_3_O_3_PSAg_2_^+^: 853.9921, found 853.9916; see the Supporting Information), which was more stable than the mononuclear complex (**Int-1′**; see the Supporting Information). The multicomponent artificial force induced reaction (MC-AFIR) method was applied to explore the reaction pathway from the intermediate state to the transition state. First, the nucleophile (**2**) coordinates with the silver metal on the phosphine side (**Int-2**), which was observed by ESI-MS analysis in the cation mode (calcd. for C_57_H_52_N_4_O_5_PSAg_2_^+^: 1149.1481, found: 1149.1481; see the Supporting Information), and was assigned as the intermediate (**Int-2** ^+^). Subsequently, the electrophile (**1a**) coordinates to the silver center on the sulfonamide side (**Int-3**). Deprotonation of the α-position of the nucleophile by the base forms **Int-4**. Notably, if deprotonation occurs prior to the formation of **Int-3**, the resulting species (**Int-4′**) is energetically less favorable. Subsequently, a nucleophilic attack on the ketimine in **Int-4** produces **Int-5**, followed by ligand exchange with potassium acetate to produce **Int-1**. For the isatin-derived ketimine **3t**, the imine nitrogen and the carbonyl oxygen adopt an s*-cis* conformation in **TS-2S3R**, and therefore, the reaction cannot proceed through the same transition state, leading to reduced selectivity. To further clarify the suitability of the developed ligand, we performed a computational analysis of transition state **TS-2S3R** using its 3D structure (Figure 3B). It was revealed that nucleophiles and electrophiles were appropriately inserted within the asymmetric space created by the steric influence of the phosphine and pyridinesulfonyl groups of the ligand, each coordinated to one of the silver cations. Calculation of the transition states of four isomers (Figure 3C) showed that **TS-2S3R**, corresponding to the major enantiomer, was more stable than its enantiomer **TS-2R3S**, with a difference in transition state energy (ΔΔ*G* ^⧧^) of +2.29 kcal/mol. The diastereomeric transition states (**TS-2R3R** and **TS-2S3S**) were less stable by +3.33 and +8.98 kcal/mol, respectively. These results confirm that the experimental diastereo- and enantioselectivities (dr = 99:1, er = 98:2) are consistent with the theoretical values obtained from DFT calculations (dr = 99.6:0.4, er = 98.5:1.5). Furthermore, DFT calculations confirmed that the “normal mode transition state” (Figure 3) is more stable than the “reverse mode transition state”, in which the nucleophile and electrophile exchange coordination sites (see, Figure S6). To investigate the origin of enantioselectivity, distortion-interaction analysis was performed (Figure 3D).^19^ The results showed that stabilization from catalyst–substrate interactions was significantly greater for the major transition state than for the minor one (Table S1–S3). Furthermore, Independent Gradient Model based on Hirshfeld (IGMH) analyses revealed key interactions between the catalyst and the substrate in the major isomer, such as CH···O, Ag–O, Ag–N, Ag···H–C, CH–π, and π–π. On the phosphine side, in addition to the interactions with silver, a CH···O hydrogen bond between the phosphine aromatic ring and the carbonyl oxygen of the nucleophile, as well as CH–π and π–π interactions with the aromatic ring of the benzophenoneimine moiety of the nucleophile, were identified (Figure 3E). On the heteroarenesulfonamide side, silver coordination was accompanied primarily by π–π interactions between the ligand’s pyridine ring and the aromatic ring of the electrophile’s Cbz group. In addition, a CH–π interaction between the methylene moiety of the electrophile and the phosphine aromatic ring was observed. These findings demonstrate that this catalytic system exhibits a high level of stereoselectivity through intricate interactions, highlighting its remarkable catalytic capability.

**Figure 3:**
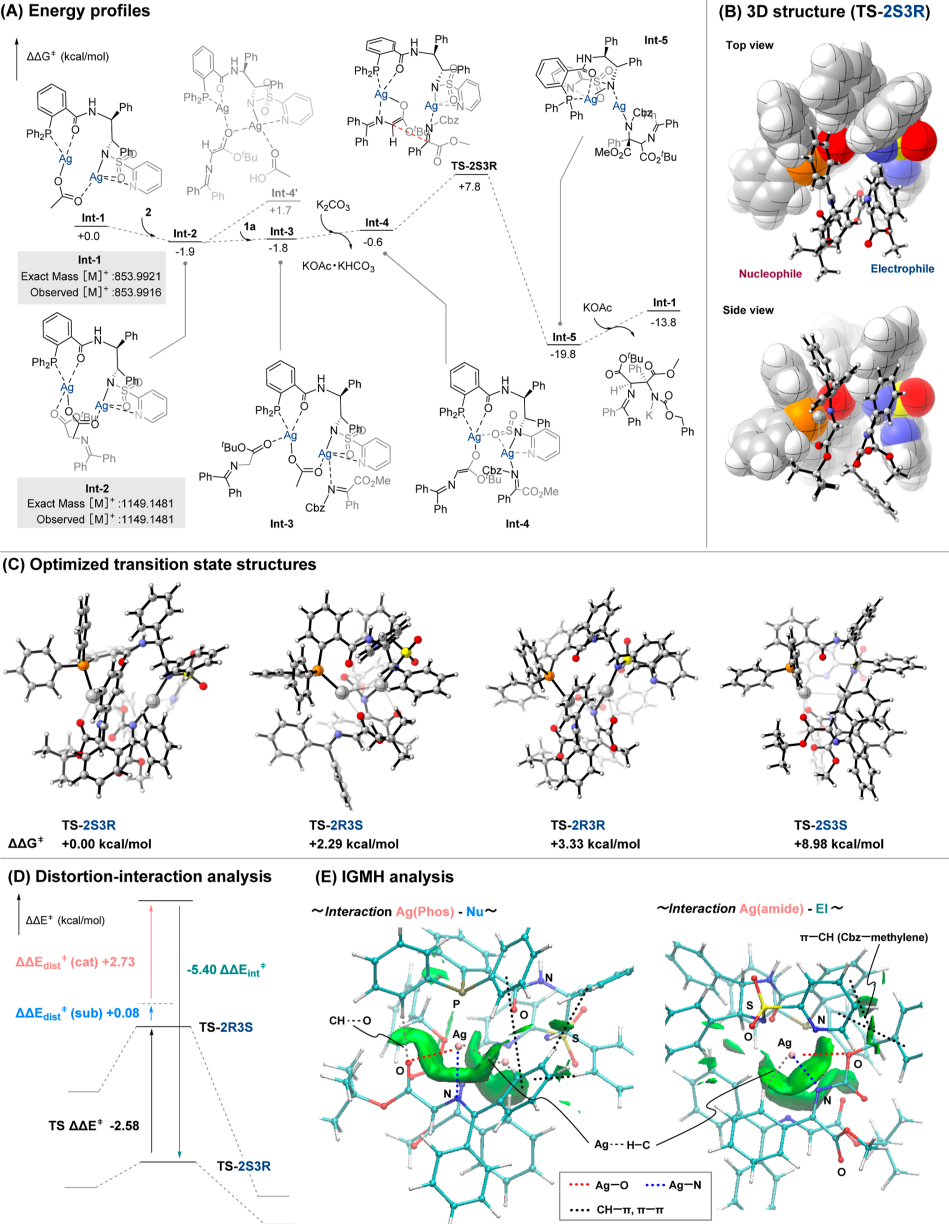
(A) Energy profiles for the asymmetric Mannich-type reaction of **1a** and **2** using ligand **A** and AgOAc. (B) 3D structure of **TS-2S3R**. (C) DFT calculation of transition states. (D) Distortion-interaction analysis. (E) IGMH analysis of **TS-2S3R**.

## Conclusion

In summary, we have established the first enantioselective Mannich-type reaction between glycinate Schiff base nucleophiles and acyclic ketimines, affording products in high yields with excellent enantioselectivities (up to 99% yield and 96% ee) using our newly developed phosphine-heteroarenesulfonamide ligand. Experimental and computational studies revealed that this ligand coordinates two silver centers, which cooperatively activate both nucleophiles and electrophiles within a chiral environment. This work introduces a design principle for asymmetric catalysis based on homobimetallic cooperation, demonstrating that metal–metal synergy and ligand-defined chirality can jointly control stereochemical outcomes. This cooperative homodinuclear design principle expands the frontier of asymmetric catalysis beyond mononuclear paradigms, offering a conceptual framework for next-generation chiral transformation. Because the donor sites of this ligand exhibit different degrees of Lewis basicity and softness, this design concept also holds great potential for extension to heterodinuclear metal complexes that cooperatively combine distinct metal species.

## Supplementary Material



## Notes

Experimental procedure and characterization data for all reactions and products, including ^1^H, ^13^C, ^19^F, and ^31^P NMR spectra, HPLC spectra, HRMS, and crystallographic data for (2*S*,3*R*)-**7** (CCDC: 2405070) (PDF)
