# Supplementary material for: Design of Phosphine-Heteroarenesulfonamide Ligands as Dinuclear Silver Catalysts for Enantioselective Construction of α,β-Diamino Acids
Source: J Am Chem Soc. 2025 Dec 11;148(2):2600–8. doi: 10.1021/jacs.5c18426 (PMC12833799; doi:10.1021/jacs.5c18426)

Supporting Information

Design of Phosphine-Heteroarenesulfonamide Ligands as Dinuclear Silver Catalysts for Enantioselective Construction of α,β-Diamino Acids

Yuka Iizuka,[a] Sayuri Okajima,[a] Yamato Ueno,[b] Tsunayoshi Takehara,[c] Takeyuki Suzuki,[c] Satoshi Maeda,[b,d,e] Shuichi Nakamura*[a]

[a] Department of Life Science and Applied Chemistry, Graduate School of Engineering, Nagoya Institute of Technology, Gokiso, Showa-ku, Nagoya 466-8555, Japan. E-mail: snakamur@nitech.ac.jp

[b] Department of Chemistry, Faculty of Science, Hokkaido University, Kita 10 Nishi 8, Kita-ku, Sapporo, Hokkaido 060-0810, Japan

[c] The Institute of Scientific and Industrial Research, Osaka University, 8-1 Mihogaoka, Ibaraki-shi, Osaka 567-0047, Japan.

[d] Institute for Chemical Reaction Design and Discovery (WPI-ICReDD), Hokkaido University, Kita 21 Nishi 10, Kita-ku, Sapporo, Hokkaido 001-0021, Japan.

[e] JST, ERATO Maeda Artificial Intelligence in Chemical Reaction Design and Discovery Project, Kita 10 Nishi 8, Kita-ku, Sapporo, Hokkaido 060-0810, Japan.

Table of Contents

Experimental section..S2

General procedure for preparation of catalysts..S3-15

DFT Calculation S16-231

Optimization of the reaction conditions S232-235

Limitation of substrates..S236-237

General procedure for preparation of Cbz-ketimines..S238-239

General procedure for the addition of glycinate Schiff base to acyclic ketimine..S240-263

X-ray crystallographic structure..S264-265

ESI-MS analysis S266-273

References..S274-275

^{1}H, ^{13}C, ^{19}F and ^{31}P NMR spectra..S276-323

1. Experimental section

General information: All reactions were performed in oven-dried glassware under a positive pressure of nitrogen. Solvents were transferred via syringe and introduced into the reaction vessels through a rubber septum. All reactions were monitored by thin-layer chromatography (TLC) carried out on 0.25 mm Merck silica-gel (60-F254). The TLC plates were visualized with UV light. Column chromatography was carried out on a column packed with silica gel 60 N (spherical, neutral, 63–210 μm). ^{1}H NMR (300, 400, 500 or 700 MHz) spectra in $CDCl_3$ or CD_3OD were recorded on Varian Mercury 300, Bruker Avance 400Plus, Bruker Avance 500, or JEOL ECZ700R spectrometers at room temperature. ^{13}C NMR (125 MHz or 176 MHz) spectra in $CDCl_3$ or CD_3OD were recorded on Bruker Avance 500 or JEOL ECZ700R spectrometers at room temperature. ^{19}F NMR (282, 376, or 659 MHz), ^{31}P NMR (121 or 283 MHz) spectra in $CDCl_3$ were recorded on Varian Mercury 300, Bruker Avance 400Plus, or JEOL ECZ700R spectrometers at room temperature. Chemical shifts (δ) are expressed in ppm downfield from internal TMS. HPLC analyses were performed on a JASCO LC-2000plus using CHIRALPAK® IF, IK, IM, IBN-3, IG-3, and AD-3 columns (4.6 x 250 mm). High-resolution mass spectra (HRMS) were recorded on a Waters SYNAPT G2 HDMS (ESI). ESI mass spectra were recorded on a Shimadzu LCMS-2020 using positive-ion mode. Optical rotations were measured using a JASCO P-2200 polarimeter. Infrared spectra were recorded on a JASCO FT/IR-4600 spectrometer with ZnSe ATR unit. X-ray crystallographic analyses were conducted on an X-ray generator or a Rigaku XtaLAB PRO MM007 DW diffractometer system equipped with a MicroMax007HFM-DW(Cu/Mo) X-ray generator and a HyPix-6000HE detector.

2. General procedure for preparation of catalysts

Ligands **J** and **K** were synthesized by the published procedure.[1)]

2.1 Synthesis of Ligand A

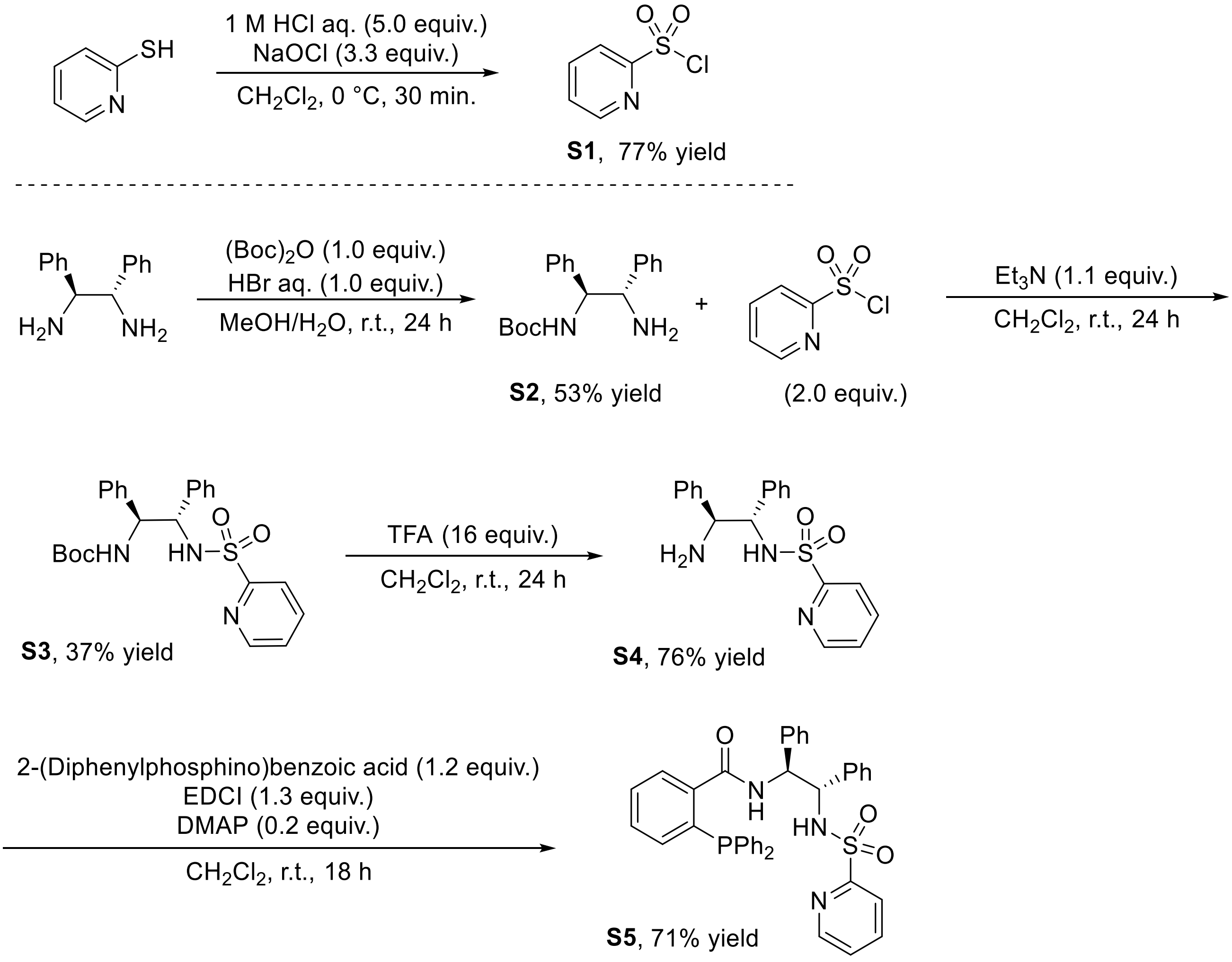

Step 1: 2-Mercaptopyridine (2.2 g, 30 mmol) was dissolved in CH_2Cl_2 (100 mL) and 1 M HCl aq. (100 mL) at 0 °C. After stirring for 30 min, an aqueous solution of NaOCl·5H_2O (10.9 g, 66 mmol) was added dropwise. The reaction mixture was stirred for an additional 30 min at the same temperature. The organic layer was separated, dried over Na_2SO_4, filtered, and concentrated under reduced pressure to afford **S1** as a known compound (4.1 g, 77% yield). The spectroscopic data were consistent with those reported in the literature.[2)]

Step 2: Aqueous HBr (2.4 mL, 20 mmol) was added to a solution of (1*S*,2*S*)-1,2-diphenylethanediamine (4.2 g, 20 mmol) in MeOH (52 mL) at room temperature. To the resulting mixture, H_2O (6.4 mL) and Boc_2O (4.8 g, 22 mmol) were subsequently added. After stirring for 24 h at room temperature, MeOH was removed under reduced pressure. The residue was treated with 25% aqueous NaOH until the pH exceeded

14, followed by extraction with EtOAc (2 times). The combined organic layers were dried over Na_2SO_4, filtered, and concentrated under reduced pressure. The crude product was purified by silica gel column chromatography (hexane:EtOAc:MeOH = 3:1:0.1) to afford **S2** (3.3 g, 53% yield). The product was identified as a known compound, exhibiting spectroscopic data consistent with those reported in the literature.[3)]

Step 3: **S1** (2.8 g, 12 mmol) and Et_3N (1.2 mL, 6.6 mmol) were added to a solution of **S2** (2.5 g, 6.0 mmol) in CH_2Cl_2 (12 mL) at 0 °C. The reaction mixture was stirred at room temperature for 24 h and then quenched with water. The resulting mixture was extracted with CH_2Cl_2, and the combined organic layers were dried over Na_2SO_4, filtered, and concentrated under reduced pressure. The crude product was purified by silica gel column chromatography (hexane: EtOAc = 7:3) to afford **S3** (1.0 g, 37% yield).

Step 4: **S3** (0.96 g, 2.2 mmol) was added to a solution of TFA (2.7 g, 35 mmol) in CH_2Cl_2 (44 mL) at room temperature, and the mixture was stirred for 24 h. The reaction was quenched by the addition of saturated aqueous $NaHCO_3$, and the mixture was extracted with CH_2Cl_2 (3 times). The combined organic layers were washed with brine, dried over Na_2SO_4, filtered, and concentrated under reduced pressure. The crude product was purified by silica gel column chromatography (EtOAc) to afford **S4** (0.59 g, 76% yield).

Step 5: 2-(Diphenylphosphino)benzoic acid (0.61 g, 2.0 mmol), EDCI (0.34 g, 2.2 mmol), and DMAP (42 mg, 0.34 mmol) were added to a solution of **S4** (0.58 g, 1.7 mmol) in CH_2Cl_2 (6.0 mL) at room temperature. The reaction mixture was stirred for 18 h at room temperature and then concentrated under reduced pressure. The crude product was purified by silica gel column chromatography in two steps (first eluent: hexane: EtOAc = 6:4; second eluent: toluene: EtOAc = 7:3) to afford **S5** (0.76 g, 71% yield).

***N*-[(1*S*,2*S*)-2-[(2-Pyridinesulfonyl)amino]-1,2-diphenylethyl]-2-(diphenylphosphino)benzamide (S5)**

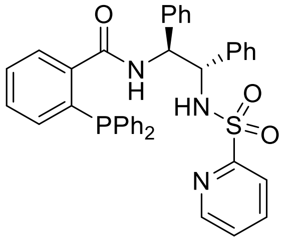

White solid, 71% yield, $[\alpha]_D^{25}$ +51.1 (*c* 0.15, $CHCl_3$); **m.p.** 130.0–131.0 ºC; **^{1}H NMR** (300 MHz, $CDCl_3$) δ 8.33–8.31 (m, 1H), 7.71–7.70 (m, 1H), 7.67–7.65 (m, 1H), 7.58–7.56 (m, 1H), 7.40–7.38 (m, 1H), 7.34–7.23 (m, 5H), 7.21–7.08 (m, 11H), 7.00–6.91 (m, 4H), 6.89–6.85 (m, 4H), 6.40–6.38 (m, 1H), 5.25–5.21 (m, 1H), 4.88–4.84 (m, 1H); **^{13}C NMR** (176 MHz, $CDCl_3$) δ 169.91, 158.23, 149.52, 140.74 (d,

J = 22.9 Hz), 138.09, 137.65, 137.52, 136.73 (d, J = 10.6 Hz), 136.57, 136.51, 136.02 (d, J = 22.9 Hz), 134.10, 133.98, 133.89 (d, J = 22.9 Hz), 130.55, 129.00, 128.92, 128.73, 128.70, 128.63, 128.60, 128.09, 127.93, 127.76, 127.71, 127.57, 126.18, 121.89, 64.10, 59.04; **^{31}P NMR** (121 MHz, $CDCl_3$) δ −10.77; **IR** (ATR) 3342, 3286, 3054, 1963, 1644, 1525, 1433, 1331, 1174, 695 cm^{-1}; **HRMS** (ESI): m/z calcd. for $C_{38}H_{33}N_3O_3SP$ $[M+H]^+$ 642.1975; found 642.1982.

2.2 Synthesis of Ligand B

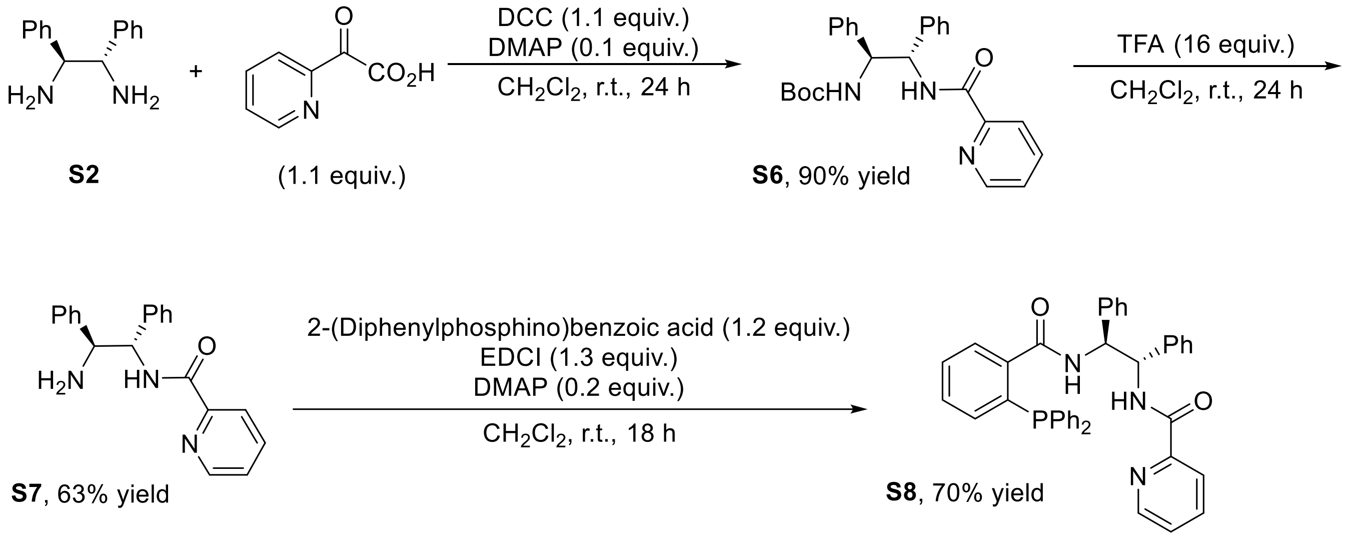

Step 6: 2-Picolinamide (0.54 g, 4.4 mmol), DCC (0.91 g, 4.4 mmol), and DMAP (48 mg, 0.4 mmol) were added to a solution of **S2** (1.2 g, 4.0 mmol) in CH_2Cl_2 (33 mL) at room temperature. The mixture was stirred for 24 h at room temperature and then filtered. The filtrate was concentrated under reduced pressure, and the crude product was purified by silica gel column chromatography (hexane: EtOAc = 6:4) to afford **S6** (1.5 g, 90% yield).

Step 7: **S6** (1.5 g, 3.6 mmol) was added to a solution of TFA (4.4 mL, 58 mmol) in CH_2Cl_2 (72 mL) at room temperature, and the mixture was stirred for 24 h. The reaction was quenched by the addition of saturated aqueous $NaHCO_3$, and the mixture was extracted with CH_2Cl_2 (3 times). The combined organic layers were washed with brine, dried over Na_2SO_4, filtered, and concentrated under reduced pressure. The crude product was purified by silica gel column chromatography (EtOAc) to afford **S7** (0.72 g, 63% yield).

Step 8: 2-(Diphenylphosphino)benzoic acid (0.80 g, 2.6 mmol), EDCI (0.45 g, 2.9 mmol), and DMAP (54 mg, 0.44 mmol) were added to a solution of **S7** (0.70 g, 2.2 mmol) in CH_2Cl_2 (8.0 mL) at room temperature. The mixture was stirred for 18 h at room temperature and then concentrated under reduced pressure. The crude product was purified by silica gel column chromatography in two steps (first eluent: hexane: EtOAc = 6:4; second eluent: toluene: EtOAc = 92:8) to afford **S8** (0.93 g, 70% yield).

***N*-[(1*S*,2*S*)-2-[(2-Pyridinecarbonyl)amino]-1,2-diphenylethyl]-2-(diphenylphosphino)benzamide (S8)**

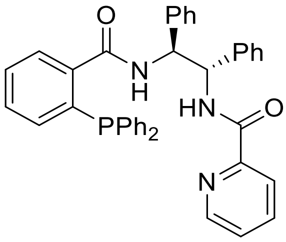

White solid, 70% yield, $[\alpha]_D^{25}$ −10.4 (*c* 0.10, $CHCl_3$); **m.p.** 118.0–119.0 ºC; **^{1}H NMR** (300 MHz, $CDCl_3$) δ 9.10–9.07 (m, 1H), 8.58–8.56 (m, 1H), 7.92–7.89 (m, 1H), 7.67–7.62 (m, 1H), 7.48–7.43 (m, 1H), 7.36–7.28 (m, 3H), 7.26–7.23 (m, 14H), 7.20–7.02 (m, 5H), 6.99–6.96 (m, 2H), 6.90–6.85 (m, 1H), 5.59–5.46 (m, 2H); **^{13}C NMR** (125 MHz, $CDCl_3$) δ 169.06, 164.96, 149.64, 148.34, 141.38 (d, *J* = 20.1 Hz), 138.64 (d, *J* = 42.1 Hz), 137.87, 137.47 (d, *J* = 11.7 Hz), 137.04, 136.84 (d, *J* = 11.7Hz), 135.96 (d, *J* = 20.1 Hz), 134.04 (d, *J* = 42.1 Hz), 133.71, 133.60 (d, *J* = 20.1 Hz), 130.05, 128.86 (d, *J* = 42.1 Hz), 128.52, 128.32 (d, *J* = 20.1 Hz), 128.43, 128.41, 128.32 (d, *J* = 20.1 Hz), 127.88, 128.83, 127.70 (d, *J* = 11.7 Hz), 127.61 (d, *J* = 20.1 Hz), 126.97, 126.08, 125.29, 122.12, 59.34, 59.28; **^{31}P NMR** (121 MHz, $CDCl_3$) δ −11.18; **IR** (ATR) 3290, 3055, 1818, 1644, 1523, 1434, 1334, 1332, 743, 695 cm^{-1}; **HRMS** (ESI): m/z calcd. for $C_{39}H_{33}N_3O_2P$ $[M+H]^+$ 606.2305; found 606.2313.

2.3 Synthesis of Ligand C

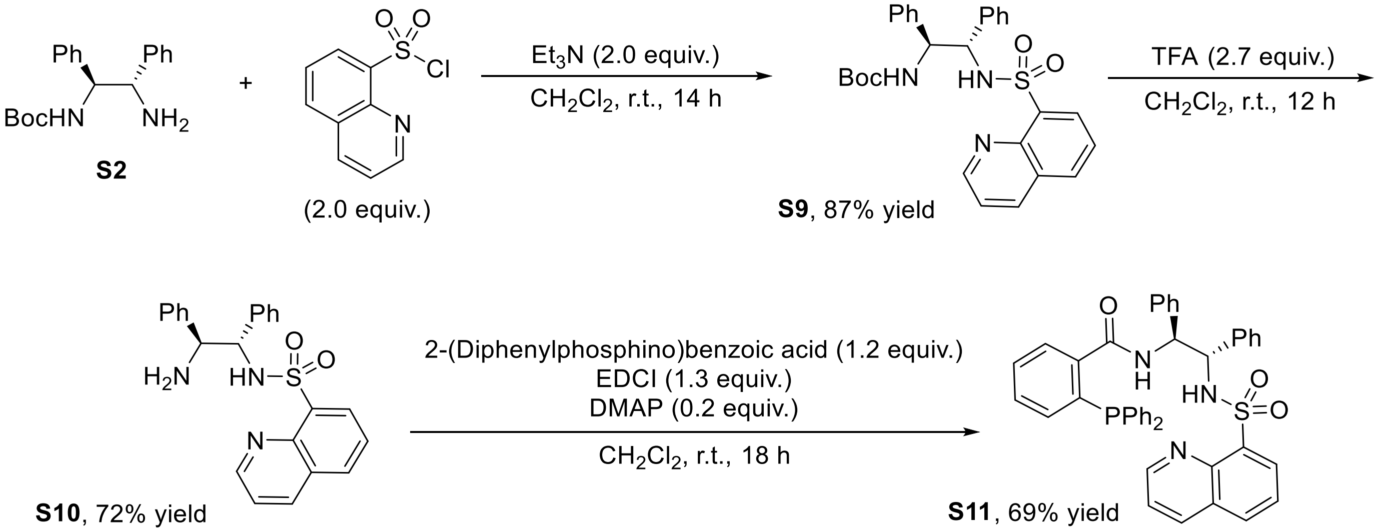

Step 9: 8-Quinolinesulfonyl chloride (0.46 g, 2.0 mmol) and Et_3N (0.15 mL, 1.1 mmol) were added to a solution of **S2** (0.30 g, 1.0 mmol) in CH_2Cl_2 (2.0 mL) at 0 °C. The reaction mixture was stirred at room temperature for 14 h and then quenched with water. The resulting mixture was extracted with CH_2Cl_2, and the combined organic layers were dried over Na_2SO_4, filtered, and concentrated under reduced pressure.

The crude product was purified by silica gel column chromatography (hexane: EtOAc = 7:3) to afford **S9** (0.44 g, 87% yield).

Step 10: **S9** (0.43 g, 0.85 mmol) was added to a solution of TFA (1.0 mL, 14 mmol) in CH_2Cl_2 (17 mL) at room temperature, and the mixture was stirred for 12 h. The reaction was quenched by the addition of saturated aqueous $NaHCO_3$, and the mixture was extracted with CH_2Cl_2 (3 times). The combined organic layers were washed with brine, dried over Na_2SO_4, filtered, and concentrated under reduced pressure. The crude product was purified by silica gel column chromatography (EtOAc) to afford **S10** (0.25 g, 72% yield).

Step 11: 2-(Diphenylphosphino)benzoic acid (0.22 g, 0.72 mmol), EDCI (0.12 g, 0.78 mmol), and DMAP (14 mg, 0.12 mmol) were added to a solution of **S10** (0.24 g, 0.60 mmol) in CH_2Cl_2 (2.2 mL) at room temperature. The mixture was stirred for 18 h at room temperature and then concentrated under reduced pressure. The crude product was purified by silica gel column chromatography in two steps (first eluent: hexane: EtOAc = 6:4; second eluent: toluene: EtOAc = 88:12) to afford **S11** (0.28 g, 69% yield).

***N*-[(1*S*,2*S*)-2-[(8-Quinolinesulfonyl)amino]-1,2-diphenylethyl]-2-(diphenylphosphino)benzamide (S11)**

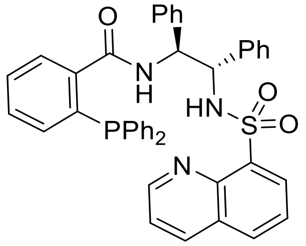

White solid, 69% yield, $[\alpha]_D^{25}$ +21.1 (*c* 0.06, $CHCl_3$); **m.p.** 171.0–172.0 ºC; **^{1}H NMR** (400 MHz, $CDCl_3$) δ 8.79–8.78 (m, 1H), 8.15–8.13 (m, 1H), 8.02–8.00 (m, 1H), 7.85–7.82 (m, 1H), 7.80–7.77 (m, 1H), 7.44–7.26 (m, 10H), 7.25–7.18 (m, 4H), 7.05–6.97 (m, 6H), 6.89–6.86 (m, 2H), 6.65–6.61 (m, 1H), 6.53–6.46 (m, 4H), 5.37–5.32 (m, 1H), 4.58–4.53 (m, 1H); **^{13}C NMR** (176 MHz, $CDCl_3$) δ 169.21, 150.92, 142.89, 141.33, 141.18, 138.27, 137.59, 137.53, 137.44, 137.37, 136.63, 136.52 (d, *J* = 22.9 Hz), 136.30, 134.38, 134.06 (d, *J* = 22.9 Hz), 133.93 (d, *J* = 22.9 Hz), 132.99, 130.39, 129.09, 128.68, 128.60, 128.56, 128.45, 128.28, 128.24, 128.21, 127.61, 127.48, 127.34, 127.12, 125.27, 121.98, 63.76, 58.08; **^{31}P NMR** (283 MHz, $CDCl_3$) δ −9.91; **IR** (ATR) 3331, 3055, 1886, 1645, 1523, 1434, 1319, 1146, 747, 695 cm^{-1}; **HRMS** (ESI): m/z calcd. for $C_{42}H_{35}N_3O_3SP$ $[M+H]^+$ 692.2132; found 692.2148.

2.4 Synthesis of Ligand D

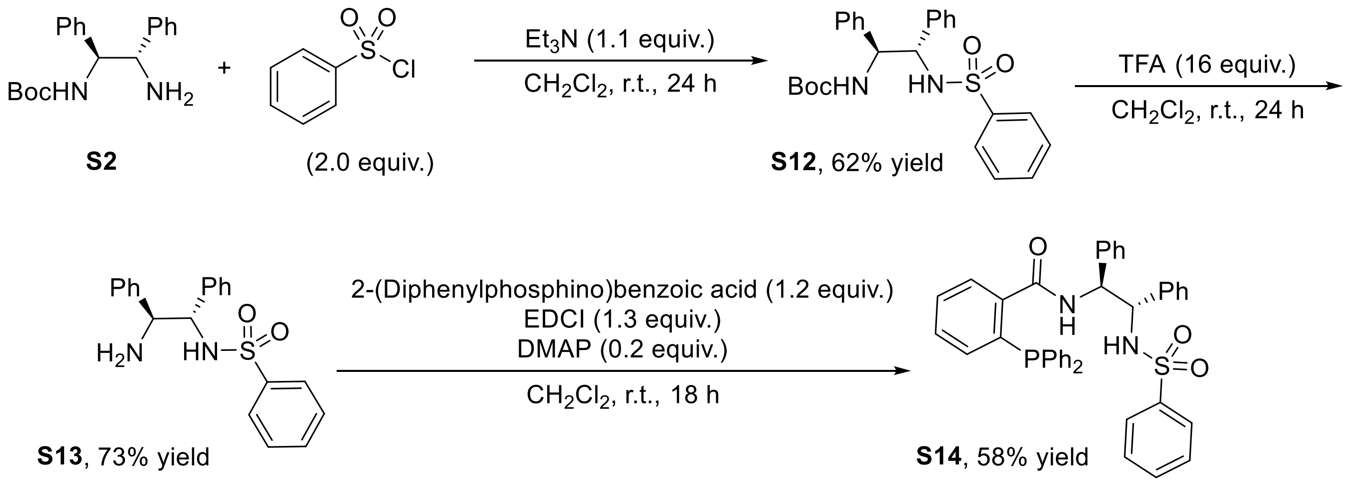

Step 12: Benzenesulfonyl chloride (0.36 g, 2.0 mmol) and Et_3N (0.15 mL, 1.1 mmol) were added to a solution of **S2** (0.30 g, 1.0 mmol) in CH_2Cl_2 (12 mL) at 0 °C. The mixture was stirred for 24 h at room temperature and then quenched by the addition of water. The mixture was extracted with CH_2Cl_2, and the combined organic layers were dried over Na_2SO_4, filtered, and concentrated under reduced pressure. The crude product was purified by silica gel column chromatography in two steps (first eluent: hexane: EtOAc = 7:3; second eluent: toluene: EtOAc = 8:2) to afford **S12** (0.45 g, 62% yield).

Step 13: **S12** (0.28 g, 0.62 mmol) was added to a solution of TFA (0.76 g, 9.9 mmol) in CH_2Cl_2 (12 mL) at room temperature, and the mixture was stirred for 24 h. The reaction was quenched by the addition of saturated aqueous $NaHCO_3$, and the mixture was extracted with CH_2Cl_2 (3 times). The combined organic layers were washed with brine, dried over Na_2SO_4, filtered, and concentrated under reduced pressure. The crude product was purified by silica gel column chromatography (EtOAc) to afford **S13** (0.22 g, 73% yield).

Step 14: 2-(Diphenylphosphino)benzoic acid (0.10 g, 0.34 mmol), EDCI (59 mg, 0.38 mmol), and DMAP (6.8 mg, 0.056 mmol) were added to a solution of **S13** (0.28 g, 0.10 mmol) in CH_2Cl_2 (1.0 mL) at room temperature. The mixture was stirred for 18 h at room temperature and then concentrated under reduced pressure. The crude product was purified by silica gel column chromatography in two steps (first eluent: hexane: EtOAc = 9:1; second eluent: toluene: EtOAc = 7:3) to afford **S14** (0.18 g, 58% yield).

***N*-[(1*S*,2*S*)-2-[(Benzenesulfonyl)amino]-1,2-diphenylethyl]-2-(diphenylphosphino)benzamide (S14)**

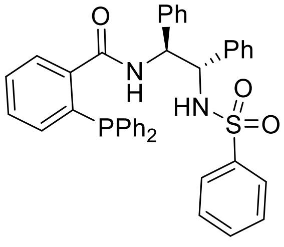

White solid, 58% yield, $[\alpha]_D^{25}$ +172.0 (*c* 0.05, $CHCl_3$); **m.p.** 128.0–129.0 ºC; **^{1}H NMR** (400 MHz, $CDCl_3$) δ 7.78–7.75 (m, 1H), 7.54–7.51 (m, 2H), 7.45–7.41 (m, 1H), 7.36–7.27 (m, 6H), 7.25–7.22 (m, 1H), 7.18–7.07 (m, 10H), 7.01–6.91 (m, 4H), 6.85–6.78 (m, 5H), 6.46–6.44 (m, 1H), 5.24–5.19 (m, 1H), 4.68–4.64 (m, 1H); **^{13}C NMR** (176 MHz, $CDCl_3$) δ 170.42, 141.04, 140.55, 140.41, 137.73, 137.22, 136.70, 136.42, 135.96, 135.85, 134.25, 134.04, 133.93, 133.83, 132.01, 130.77, 129.19, 128.96, 128.93, 128.75, 128.71, 128.56, 128.11, 128.04, 127.65, 127.59, 126.87, 64.23, 59.29; **^{31}P NMR** (283 MHz, $CDCl_3$) δ −10.36; **IR** (ATR) 3271, 3061, 1959, 1637, 1514, 1434, 1320, 1158, 1090, 927 cm^{-1}; **HRMS** (ESI): m/z calcd. for $C_{39}H_{34}N_2O_3SP$ $[M+H]^+$ 641.2023; found 641.2018.

2.5 Synthesis of Ligand E

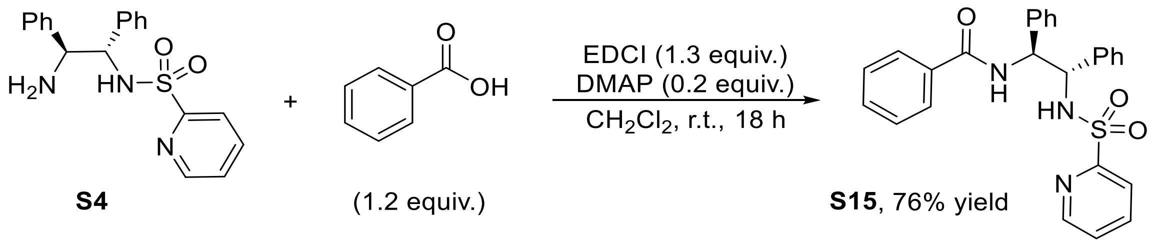

Step 15: Benzoic acid (46 mg, 0.38 mmol), EDCI (64 mg, 0.41 mmol), and DMAP (7.8 mg, 0.064 mmol) were added to a solution of **S4** (0.13 g, 0.32 mmol) in CH_2Cl_2 (1.1 mL) at room temperature. The mixture was stirred for 18 h at room temperature and then concentrated under reduced pressure. The crude product was purified by silica gel column chromatography (toluene: EtOAc = 7:3) to afford **S18** (0.11 g, 76% yield).

***N*-[(1*S*,2*S*)-2-[(2-Pyridinesulfonyl)amino]-1,2-diphenylethyl]-2-(diphenylphosphino)benzamide (S15)**

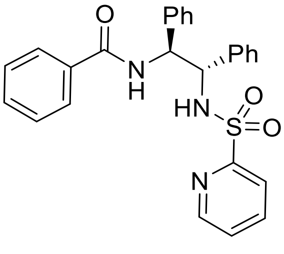

White solid, 76% yield, $[\alpha]_D^{25}$ −83.7 (*c* 0.06, $CHCl_3$); **m.p.** 254.0–255.0 ºC; **^{1}H NMR** (400 MHz,

$CDCl_3$) δ 8.40–8.38 (m, 1H), 7.77–7.73 (m, 3H), 7.66–7.61 (m, 1H), 7.49–7.45 (m, 2H), 7.38–7.34 (m, 2H), 7.25–7.23 (m, 1H), 7.21–7.16 (m, 3H), 7.12–7.09 (m, 2H), 7.05–6.99 (m, 3H), 6.96–6.94 (m, 2H), 6.74–6.72 (m, 1H), 5.47–5.43 (m, 1H), 4.93–4.90 (m, 1H); **^{13}C NMR** (125 MHz, $CDCl_3$) δ 168.12, 157.96, 149.61, 138.52, 137.97, 137.74, 133.72, 131.71, 128.52, 128.18, 127.90, 127.82, 127.70, 127.56, 127.54, 127.26, 126.30, 121.81, 63.56, 59.26; **IR** (ATR) 3112, 3014, 2784, 1709, 1577, 1447, 1337, 1225, 1154, 953 cm^{-1}; **HRMS** (ESI): m/z calcd. for $C_{26}H_{24}N_3O_3S$ $[M+H]^+$ 458.1533; found 458.1541.

2.6 Synthesis of Ligand F

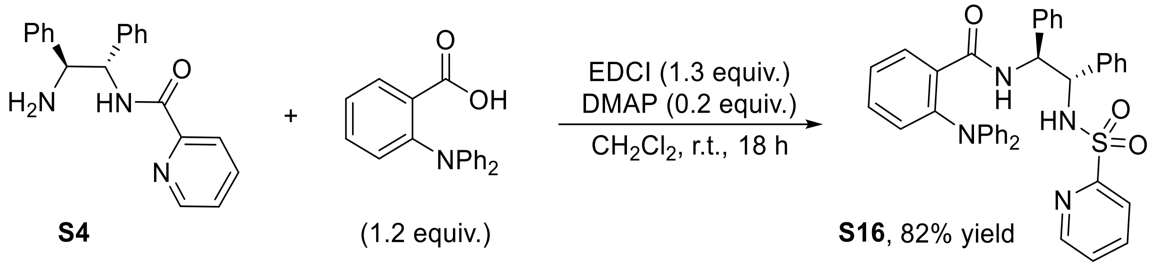

Step 16: 2-(Diphenylamino)benzoic acid (0.35 g, 1.20 mmol), EDCI (0.20 g, 1.30 mmol), and DMAP (24.0 mg, 0.20 mmol) were added to a solution of **S4** (0.33 g, 0.96 mmol) in CH_2Cl_2 (20 mL) at room temperature. The mixture was stirred for 18 h at room temperature and then concentrated under reduced pressure. The crude product was purified by silica gel column chromatography (hexane: EtOAc = 6:4) to afford **S16** (0.49 g, 82% yield).

***N*-[(1*S*,2*S*)-2-[(2-Pyridinesulfonyl)amino]-1,2-diphenylethyl]-2-(diphenylamino)benzamide (S16)**

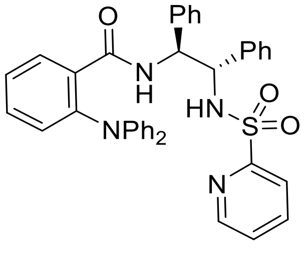

White solid, 82% yield, $[\alpha]_D^{25}$ −132.1 (*c* 0.04, $CHCl_3$); **m.p.** 99.0–100.0 ºC; **^{1}H NMR** (300 MHz, $CDCl_3$) δ 8.33–8.30 (m, 1H), 8.06–8.00 (m, 2H), 7.63–7.54 (m, 2H), 7.51–7.45 (m, 1H), 7.38–7.33 (m, 1H), 7.23–7.19 (m, 1H), 7.13–7.07 (m, 6H), 7.02–6.87 (m, 7H), 6.77–6.74 (m, 2H), 6.71–6.66 (m, 6H), 6.42–6.40 (m, 1H), 4.99–4.94 (m, 1H), 4.75–4.69 (m, 1H); **^{13}C NMR** (125 MHz, $CDCl_3$) δ 167.69, 158.06, 149.51, 147.19, 144.89, 137.82, 137.56, 137.44, 132.69, 132.18, 131.83, 130.14, 129.28, 128.46, 127.89, 127.85, 127.60, 127.53, 127.37, 126.24, 125.94, 122.70, 122.28, 121.81, 64.36, 59.28; **IR** (ATR) 3677, 3407, 3055, 2985, 2931, 2685, 2307, 1726, 1265, 733 cm^{-1}; **HRMS** (ESI): m/z calcd. for $C_{38}H_{33}N_4O_3S$ $[M+H]^+$ 625.2268; found 625.2274.

2.7 Synthesis of Ligand G[1a)]

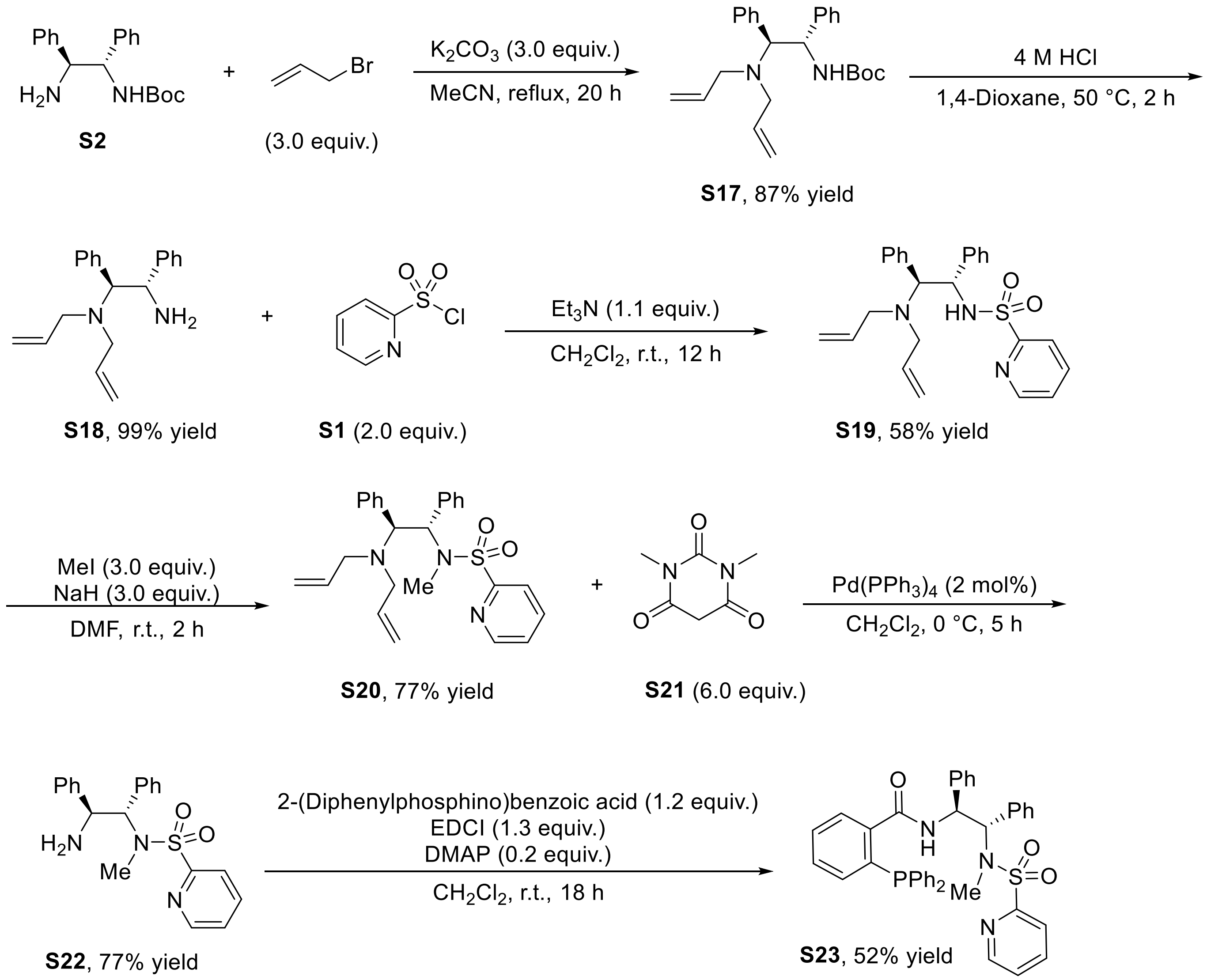

Step 17: A solution of **S2** (3.8 g, 12 mmol), K_2CO_3 (5.1 g, 37 mmol), and allyl bromide (3.1 mL, 37 mmol) in CH_3CN (123 mL) was stirred at 85 ºC for 20 h and filtered. The filtrate was concentrated, and the crude residue was purified by flash column chromatography on silica gel (hexane: EtOAc = 98:2) to give **S17** (4.2 g, 87% yield). The spectroscopic data were consistent with those reported in the literature.[1a)]

Step 18: To a solution of **S17** (3.7 g, 9.4 mmol) in 1,4-dioxane (75 mL) was added 4 M HCl (78 mL). The reaction mixture was stirred at room temperature for 2 h and then concentrated under reduced pressure. The crude residue was added with H_2O, and the pH value was adjusted to 10 using 25% aqueous NaOH. The resulting mixture was extracted with CH_2Cl_2, and the combined extracts were concentrated to afford **S18** (3.5 g, 9.2 mmol), which was used directly in the next step without further purification. **S1** (4.7 g, 26 mmol) and Et_3N (2.0 mL, 14 mmol) were added to a solution of **S18** (4.1 g, 13.1 mmol) in CH_2Cl_2 (26 mL) at 0 °C. The reaction mixture was stirred at room temperature for 12 h and then quenched with water. The resulting mixture was extracted with CH_2Cl_2, and the combined organic layers were dried over Na_2SO_4,

filtered, and concentrated under reduced pressure. The crude product was purified by silica gel column chromatography (hexane: EtOAc = 95:5) to afford **S19** (3.3 g, 58% yield).

Step 19: To a solution of **S19** (3.3 g, 7.6 mmol) in DMF (57 mL) was added NaH (0.55 g, 23 mmol, 60% dispersion in mineral oil) at room temperature. The reaction was stirred for 10 min before the addition of MeI (1.4 mL, 23 mmol). After being stirred at room temperature for 2 h, the reaction mixture was quenched with H_2O and extracted with EtOAc (3 times). The combined extract was dried over Na_2SO_4 and concentrated. The crude residue was purified by flash column chromatography on silica gel (hexane: EtOAc = 9:1 to 8:2) to afford **S20** (2.6 g, 77% yield).

Step 20: To a solution of **S20** (2.6 g, 5.8 mmol), **S21** (5.5 g, 35 mmol), and $Pd(PPh_4)_3$ (0.14 g, 0.12 mmol) in CH_2Cl_2 (63 mL) was stirred at 0 ºC for 5 h. The reaction mixture was quenched with a saturated solution of $NaHCO_3$ and extracted with CH_2Cl_2 (3 times). The combined extract was dried over Na_2SO_4 and concentrated. The crude residue was purified by flash column chromatography on silica gel (hexane: EtOAc = 1:1) to afford **S22** (1.9 g, 77% yield).

Step 21: 2-(Diphenylphosphino)benzoic acid (1.9 g, 6.1 mmol), EDCI (1.0 g, 6.6 mmol), and DMAP (0.12 g, 1.0 mmol) were added to a solution of **S22** (1.8 g, 5.1 mmol) in CH_2Cl_2 (18 mL) at room temperature. The mixture was stirred for 18 h at room temperature and then concentrated under reduced pressure. The crude product was purified by silica gel column chromatography in two steps (first eluent: EtOAc = 7:3) to afford **S23** (1.7 g, 52% yield).

***N*-[(1*S*,2*S*)-2-[Methyl(2-pyridinesulfonyl)amino]-1,2-diphenylethyl]-2-(diphenylphosphino) benzamide (S23)**

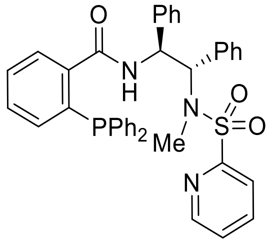

White solid, 52% yield, $[\alpha]_D^{25}$ +166.1 (*c* 0.07, $CHCl_3$); **m.p.** 233.0–234.0 ºC; **^{1}H NMR** (400 MHz, $CDCl_3$) δ 8.07–8.05 (m, 1H), 8.02–7.99 (m, 1H), 7.82–7.78 (m, 1H), 7.75–7.74 (m, 1H), 7.64–7.61 (m, 1H), 7.44–7.40 (m, 1H), 7.36–7.34 (m, 1H), 7.32–7.26 (m, 4H), 7.25–7.07 (m, 15H), 7.01–6.94 (m, 3H), 5.87–5.82 (m, 1H), 5.70–5.67 (m, 1H), 2.86 (s, 3H); **^{13}C NMR** (176 MHz, $CDCl_3$) δ 169.01, 157.81, 149.84, 142.45, 142.29, 139.34, 138.00, 137.88, 137.81, 137.31, 137.24, 136.73 (d, *J* = 19.9 Hz), 135.28, 134.54, 133.93 (d, *J* = 19.9 Hz), 133.55 (d, *J* = 19.9 Hz), 130.12, 129.12, 128.93, 128.60, 128.50, 128.45, 128.26, 128.14,

127.59, 127.54, 126.53, 122.95, 64.19, 53.09, 29.82; **^{31}P NMR** (283 MHz, $CDCl_3$) δ −10.32; **IR** (ATR) 3269, 1960, 1819, 1710, 1633, 1519, 1447, 1323, 1155, 952 cm^{-1}; **HRMS** (ESI): m/z calcd. for $C_{39}H_{35}N_3O_3SP$ $[M+H]^+$ 656.2132; found 656.2140.

2.8 Synthesis of Ligand H

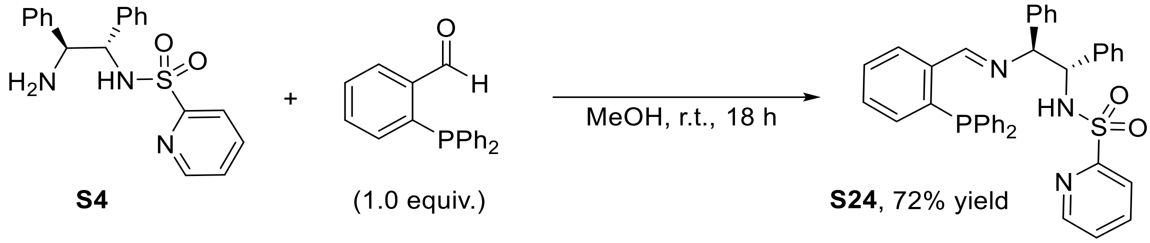

Step 21: 2-(Diphenylphosphanyl)benzaldehyde (0.15 g, 0.50 mmol) was added to a solution of **S4** (0.18 g, 0.50 mmol) in MeOH (3.7 mL) at room temperature for 18 h. The reaction mixture was filtered to afford **S24** (0.21 g, 72% yield).

***N*-[(1*S*,2*S*)-2-[[[2-(Diphenylphosphino)phenyl]methylene]amino]-1,2-diphenylethyl]-2-pyridinesulfonamide (S24)**

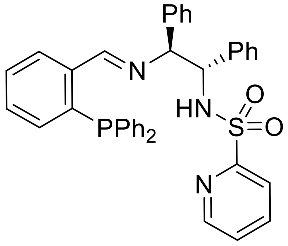

White solid, 72% yield, $[\alpha]_D^{25}$ +61.2 (*c* 0.19, $CHCl_3$); **m.p.** 152.0–153.0 ºC; **^{1}H NMR** (400 MHz, $CDCl_3$) δ 8.35–8.33 (m, 1H), 8.05–8.04 (m, 1H), 7.57–7.53 (m, 1H), 7.49–7.44 (m, 2H), 7.42–7.28 (m, 12H), 7.18–7.12 (m, 4H), 7.06–6.98 (m, 5H), 6.95–6.91 (m, 2H), 6.73–6.71 (m, 2H), 5.00 (dd, *J* = 8.7, 2.4 Hz, 1H), 4.41–4.40 (m, 1H); **^{13}C NMR** (176 MHz, $CDCl_3$) δ 162.49, 158.19, 149.56, 140.64, 139.97, 138.76, 138.66, 138.00, 137.94, 137.06, 135.09, 134.74, 134.63, 133.62, 133.51, 131.87, 130.52, 129.04, 128.81, 128.75, 128.72, 128.58, 128.15, 128.01, 127.16, 127.10, 125.67, 121.39, 80.36, 64.31; **^{31}P NMR** (283 MHz, $CDCl_3$) δ −8.67; **IR** (ATR) 3650, 3056, 2867, 2839, 2305, 1956, 1647, 1332, 1175, 742 cm^{-1}; **HRMS** (ESI): m/z calcd. for $C_{38}H_{32}N_3O_2SP$ $[M+H]^+$ 626.2026; found 626.2028.

2.9 Synthesis of Ligand I

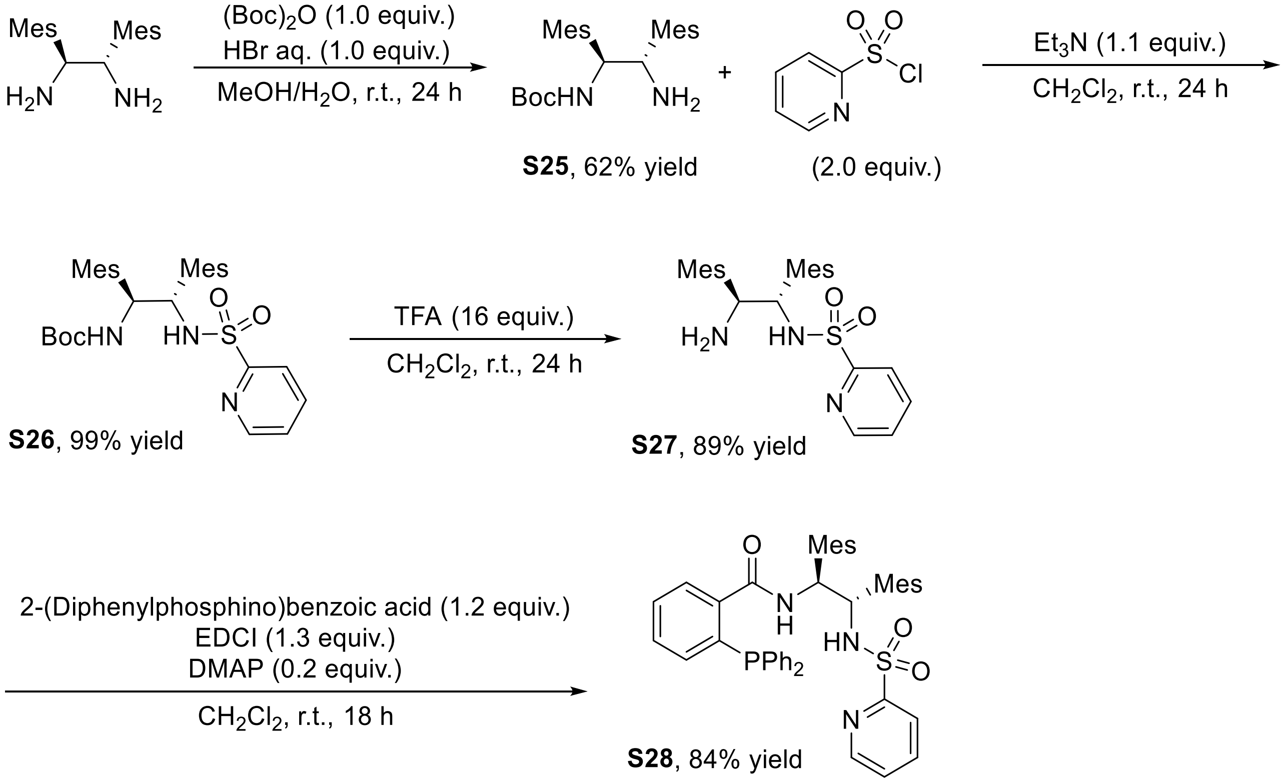

Step 22: Aqueous HBr (0.24 mL, 2.0 mmol) was added to a solution of (1*S*,2*S*)-1,2-bis(2,4,6-trimethylphenyl)-1,2-ethanediamine (0.59 g, 2.0 mmol) in methanol (5.2 mL) at room temperature. To the resulting mixture, H_2O (0.64 mL) and Boc_2O (0.48 g, 2.2 mmol) were subsequently added. After stirring for 24 h at room temperature, methanol was removed under reduced pressure. The residue was treated with 25% aqueous NaOH until the pH exceeded 14, followed by extraction with EtOAc (2 times). The combined organic extracts were dried over Na_2SO_4, filtered, and concentrated under reduced pressure. The crude product was purified by silica gel column chromatography (hexane: EtOAc: methanol = 3:1:0.1) to afford **S25** (0.49 g, 62% yield). The product was identified as a known compound, exhibiting spectroscopic data consistent with those reported in the literature.[4)]

Step 23: **S1** (0.44 g, 2.5 mmol) and Et_3N (2.5 mL, 1.4 mmol) were added to a solution of **S22** (0.49 g, 1.2 mmol) in CH_2Cl_2 (2.5 mL) at 0 °C. The reaction mixture was stirred at room temperature for 24 h and then quenched with water. The resulting mixture was extracted with CH_2Cl_2, and the combined organic layers were dried over Na_2SO_4, filtered, and concentrated under reduced pressure. The crude product was purified by silica gel column chromatography (toluene: EtOAc = 95:5) to afford **S26** (0.68 g, 99% yield).

Step 24: **S26** (0.68 g, 1.2 mmol) was added to a solution of TFA (1.5 mL, 20 mmol) in CH_2Cl_2 (19 mL) at room temperature, and the mixture was stirred for 24 h. The reaction was quenched by the addition of

saturated aqueous $NaHCO_3$, and the mixture was extracted with CH_2Cl_2 (3 times). The combined organic layers were washed with brine, dried over Na_2SO_4, filtered, and concentrated under reduced pressure. The crude product was purified by silica gel column chromatography (hexane: EtOAc = 5:5) to afford **S27** (0.49 g, 89% yield).

Step 25: 2-(Diphenylphosphino)benzoic acid (0.40 g, 1.3 mmol), EDCI (0.27 g, 1.4 mmol), and DMAP (24 mg, 0.2 mmol) were added to a solution of **S27** (0.49 g, 1.1 mmol) in CH_2Cl_2 (20 mL) at room temperature. The reaction mixture was stirred for 18 h at room temperature and then concentrated under reduced pressure. The crude product was purified by silica gel column chromatography in two steps (eluent: hexane: EtOAc = 6:4) to afford **S28** (0.92 g, 84% yield).

***N*-[(1*S*,2*S*)-2-[(2-Pyridinesulfonyl)amino]-1,2-bis(2,4,6-trimethylphenyl)ethyl]-2-(diphenylphosphino)benzamide (S28)**

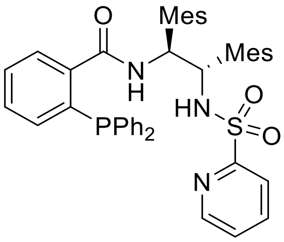

White solid, 84% yield, $[\alpha]_D^{25}$ −82.9 (*c* 0.07, $CHCl_3$); **m.p.** 132.0–133.0 ºC; **^{1}H NMR** (400 MHz, $CDCl_3$) δ 8.13–8.11 (m, 1H), 7.74–7.67 (m, 3H), 7.61–7.57 (m, 1H), 7.45–7.41 (m, 1H), 7.34–7.27 (m, 3H), 7.24–7.13 (m, 7H), 7.06–7.01 (m, 2H), 6.88–6.85 (m, 1H), 6.72–6.71 (m, 1H), 6.56–6.50 (m, 3H), 6.36 (s, 1H), 5.89–5.84 (m, 1H), 5.46–5.41 (m, 1H), 2.55 (s, 3H), 2.41 (s, 3H), 2.18 (s, 3H), 2.08 (s, 3H), 1.55–1.54 (m, 6H); **^{13}C NMR** (176 MHz, $CDCl_3$) δ 170.00, 158.31, 149.16, 141.38, 141.24, 138.32, 137.56, 137.10, 137.07, 136.89, 136.55, 136.22, 136.17, 136.01, 135.96, 135.64, 135.56, 135.47, 134.02 (d, *J* = 21.1 Hz), 133.92 (d, *J* = 21.1 Hz), 133.53, 131.65 (d, *J* = 21.1 Hz) , 130.90, 130.85, 130.21, 129.34, 129.00, 128.72, 128.60, 128.50, 126.09, 121.72, 56.51, 51.60, 21.16, 20.87, 20.74, 20.74, 20.22, 19.64; **^{31}P NMR** (283 MHz, $CDCl_3$) δ −10.03; **IR** (ATR) 3277, 3014, 2864, 2823, 1662, 1434, 1174, 1122, 744, 696 cm^{-1}; **HRMS** (ESI): m/z calcd. for $C_{44}H_{45}N_3O_3SP$ $[M+H]^+$ 726.2914; found 726.2932.

3. DFT Calculation

All density functional theory (DFT) calculations were performed using Gaussian 16 revision C.02.[6] B3LYP functional with def2-SV(P) basis set for all the atoms, and the CPCM solvation mode (THF) was employed for the geometry optimization. For reaction pathway calculations, the artificial force induced reaction (AFIR) method, implemented in the global reaction route mapping (GRRM) program[5] combined with Gaussian 16 program was utilized. Probable conformations of dinuclear catalysts and intermediate structures were initially obtained using the AFIR method. Automated reaction path searches using the single-component artificial force induced reaction (SC-AFIR) method and the multi-component artificial force induced reaction (MC-AFIR) method were also conducted at GFN2-xTB level using the ORCA 4.2.0 software[7] to obtain the initial structures for optimizations. All obtained equilibrium structures (EQ) were re-optimized at the above computational level. After optimization of structures, frequency calculations were performed at the same level of the theory to confirm that the obtained structures were either stationary points (no imaginary frequencies) or transition states (one imaginary frequency). IRC calculations were performed for each transition state structure to confirm that the transition state connects the reaction pathway between the starting materials and the products or intermediates. Thermal corrections to the Gibbs free energy at 273.15 K (0 °C) were calculated by frequency calculation. Single-point energy calculations for the optimized geometry were performed using M06-D3 functional with def2-TZVPP basis set for all atoms in CPCM solvation model (THF). 3D Models were visualized by CYLview[8] and GaussView. Natural bond orbital (NBO) analysis was carried out using NBO version 7.0.[9]

3.1 Preliminary conformational search of new design catalysts

3.1.1 SC-AFIR Search

The reaction path between **Ligand A** and **Int-1** was explored by the SC-AFIR method implemented in the GRRM program by dividing it into four cases (**Scheme S1**). In the initial search, electronic structure calculations were carried out by the GFN2-xTB method in the Orca program. The artificial force is applied between 16 selected (red) atoms. The collision energy parameter γ of the AFIR method was set to γ = 150.0 kJ/mol. The last integer in each Cartesian coordinate is for random distribution of each molecule; in the case (A), ligand A is part #1, two silver atoms are part #2 or 3, the acetate anion is part #4, and two THF are part #5 or 6. In the case (B), ligand A is part #1, two silver atoms are part #2 or 3, two acetate anions are part #4 or 5, and two THF are part #6 or 7. In the case (C), ligand A is part #1, two silver atoms are part #2 or 3, and two THF are part #4 or 5. In the case (D), ligand A is part #1, two silver atoms are part #2 or 3, the acetate anion is part #4, and two THF are part #5 or 6. A total of 26 equilibrium (EQ)

structures (A: 7EQ, B: 6EQ, C: 6EQ, D: 7EQ) were re-optimized at the B3LYP/def2-SV(P)/CPCM(THF) after removing the THF molecules.

(A) Deprotonation of sulfonamide protone

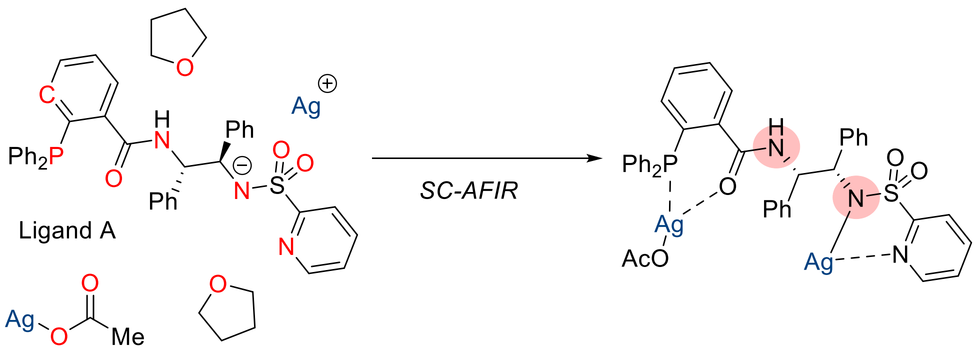

(B) Without deprotonation of amide protone

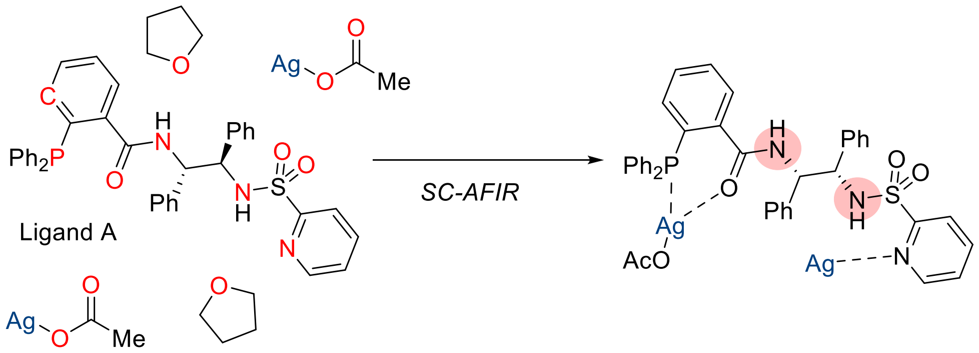

(C) Deprotonation of both amide protone

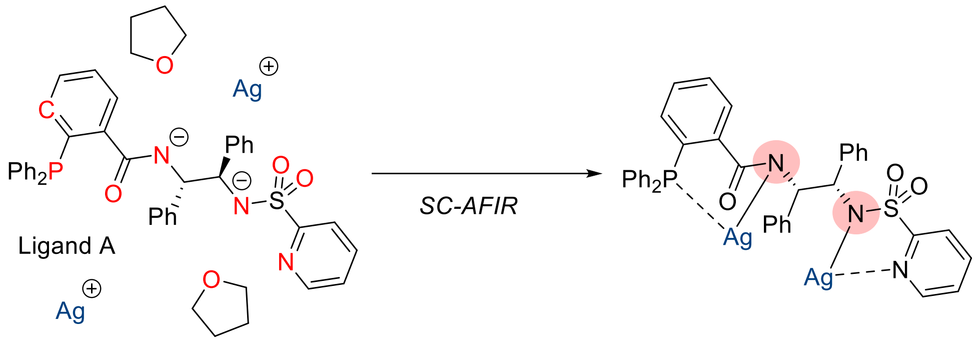

(D) Deprotonation of amide protone

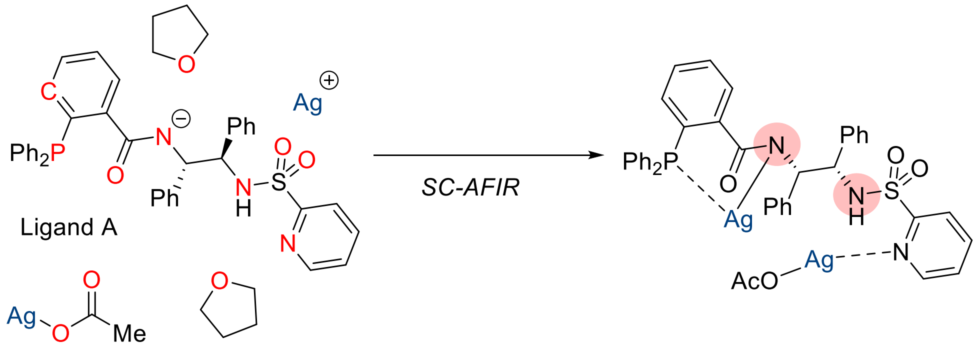

Scheme S1. SC-AFIR search divided into four cases.

3.1.2 Results (the GFN2-xTB method in the Orca program)

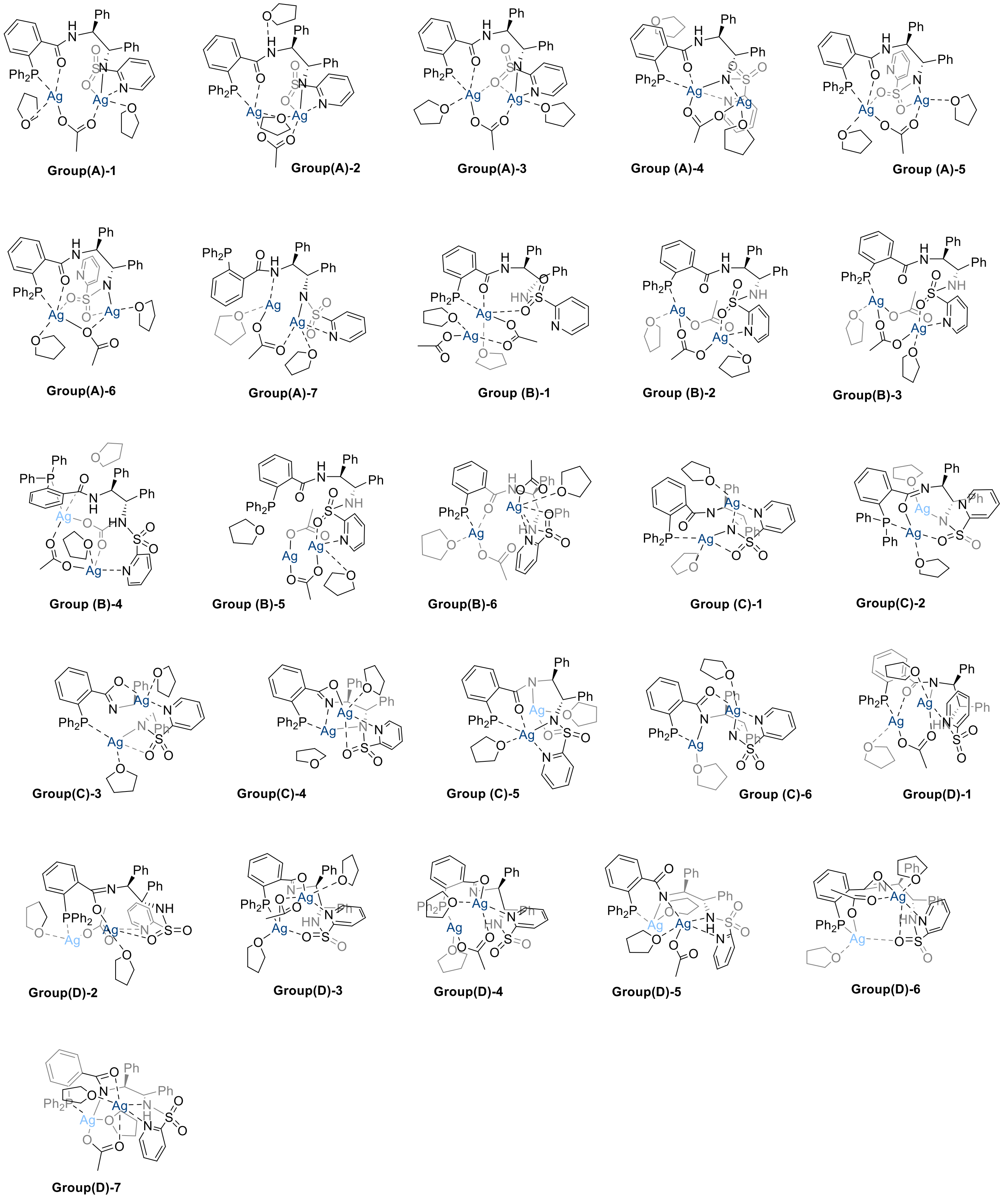

Figure S1. Result of SC-AFIR search divided into four cases by the GFN2-xTB method in the Orca program.

Calculated Structures

(A) Deprotonation of sulfonamide protone

Group(A)-1

C	1.6052	-0.10213	1.782323
C	2.539489	-1.31039	1.443036
C	4.629155	-0.79311	2.642167
C	5.913446	-0.07844	2.638241
O	4.23337	-1.2164	3.745367
C	6.377921	0.270591	3.920198
C	7.541561	1.034674	4.006133
C	8.240761	1.397732	2.865932
C	7.783076	1.024243	1.607177
C	6.613351	0.294071	1.492174
P	5.322927	-0.13592	5.369862
S	3.088569	2.03218	2.183471
C	2.060425	3.368972	1.540589
O	3.710363	2.606599	3.378755
C	2.400036	4.001362	0.359892
C	1.574152	5.016957	-0.09661
C	0.452451	5.347062	0.65223
C	0.19604	4.655686	1.824337
N	0.991626	3.677647	2.256427
O	3.988865	1.73372	1.066487
C	2.261607	-2.53925	2.289278
C	1.826295	-2.4629	3.608897
C	1.59193	-3.61527	4.342332
C	1.787971	-4.86222	3.764886
C	2.223894	-4.94889	2.450673
C	2.457887	-3.79367	1.717648
C	1.064927	0.609223	0.556414
C	1.794806	0.776824	-0.61761
C	1.260165	1.48493	-1.68517
C	-0.00993	2.036456	-1.59569
C	-0.74864	1.868454	-0.43226
C	-0.21564	1.156543	0.63121
C	5.769237	-1.85418	5.85193
C	6.41478	-2.74845	4.999735

C	6.690302	-4.04279	5.414616
C	6.319408	-4.46363	6.684417
C	5.672113	-3.58186	7.539849
C	5.398784	-2.28744	7.125763
C	6.27982	0.743551	6.713856
C	5.721711	1.910753	7.233615
C	6.362191	2.643408	8.220602
C	7.577309	2.202951	8.724855
C	8.140967	1.030134	8.237138
C	7.500805	0.308919	7.240379
H	0.738256	-0.52784	2.305885
H	2.349253	-1.58719	0.396609
H	7.914901	1.349511	4.973155
H	9.150391	1.980928	2.958205
H	8.33334	1.312784	0.718865
H	6.244011	0.015613	0.511518
H	3.286465	3.695633	-0.18018
H	1.798976	5.539426	-1.01912
H	-0.21972	6.132749	0.33026
H	-0.66954	4.883219	2.436845
H	1.702552	-1.49777	4.084745
H	1.262849	-3.53798	5.372658
H	1.606937	-5.76376	4.339363
H	2.384688	-5.91866	1.992631
H	2.8026	-3.86993	0.691203
H	2.796172	0.373141	-0.70646
H	1.843891	1.610504	-2.59047
H	-0.42283	2.593991	-2.4289
H	-1.7435	2.293081	-0.35259
H	-0.79896	1.026741	1.535885
H	6.703593	-2.43269	4.003612
H	7.195572	-4.72729	4.741654
H	6.532569	-5.47702	7.006255
H	5.376907	-3.90499	8.532218
H	4.890878	-1.60395	7.798306
H	4.763907	2.250263	6.854725
H	5.909983	3.557104	8.589078
H	8.084875	2.767613	9.499331
H	9.087394	0.677466	8.633392

H	7.953004	-0.60471	6.86796
N	2.192301	0.830432	2.728796
N	3.951333	-0.94659	1.498808
H	4.250977	-0.35934	0.731632
Ag	0.757452	2.23217	4.091082
C	-2.41015	1.203835	4.195691
O	-1.15261	0.571747	3.990321
C	-0.97234	-0.41688	5.005899
C	-1.8793	-0.02094	6.184061
C	-2.57719	1.259737	5.714631
H	-2.37722	2.190967	3.727693
H	-3.20983	0.606767	3.730479
H	-1.24443	-1.40477	4.608425
H	0.084142	-0.42322	5.287259
H	-2.60489	-0.81132	6.390526
H	-1.29366	0.151674	7.088073
H	-3.62702	1.295199	6.007857
H	-2.06593	2.138936	6.113625
C	5.253585	4.810164	5.127333
O	4.547639	4.681117	6.34843
C	3.176304	4.543362	6.043215
C	2.928296	5.434261	4.816838
C	4.309931	5.546515	4.157568
H	6.175141	5.368019	5.323943
H	5.51325	3.817073	4.728435
H	2.939881	3.492621	5.800023
H	2.592026	4.843253	6.918116
H	2.19219	4.984325	4.14887
H	2.561521	6.417342	5.122311
H	4.324362	5.073846	3.174129
H	4.604996	6.592101	4.041926
Ag	3.051727	0.808731	5.140633
O	1.599347	1.04828	6.926363
C	0.822697	1.998655	7.142773
O	0.340304	2.771663	6.285123
C	0.431201	2.255518	8.593712
H	-0.54088	2.739969	8.641029
H	0.425494	1.320409	9.148354
H	1.182265	2.91744	9.02279

Group(A)-2

C	2.284807	0.770141	2.397259
C	3.671277	0.619067	1.715861
C	4.905815	0.553362	3.884641
C	6.120406	0.971872	4.608557
O	4.032408	-0.04317	4.554549
C	6.002743	0.928967	6.008844
C	7.094068	1.346576	6.769511
C	8.273824	1.746664	6.160641
C	8.384176	1.758449	4.775374
C	7.304267	1.377458	3.996365
P	4.355515	0.516924	6.71714
S	2.620427	3.289814	3.092035
C	1.203812	4.173657	2.402021
O	2.852806	3.930119	4.386999
C	1.410675	5.23049	1.534922
C	0.298979	5.859348	0.996319
C	-0.96091	5.395269	1.350875
C	-1.06579	4.328478	2.227773
N	0.005908	3.725252	2.740589
O	3.673584	3.466643	2.088669
C	3.752903	-0.75757	1.090814
C	4.012935	-1.88904	1.860151
C	4.044547	-3.14328	1.268949
C	3.809194	-3.28219	-0.0922
C	3.539454	-2.15952	-0.8622
C	3.510845	-0.90359	-0.27289
C	1.206951	0.948107	1.341393
C	1.287674	1.932551	0.357555
C	0.261187	2.100717	-0.56026
C	-0.86141	1.285672	-0.51218
C	-0.94549	0.294498	0.455031
C	0.082709	0.126276	1.371536
C	4.33086	-1.31374	6.915685
C	5.113116	-2.18169	6.155359
C	5.001048	-3.55493	6.313649
C	4.101853	-4.08261	7.230258
C	3.319476	-3.22785	7.995523

C	3.434875	-1.85507	7.83937
C	4.68173	0.923863	8.516046
C	4.075557	2.062864	9.046916
C	4.246545	2.418693	10.3763
C	5.030874	1.632062	11.2078
C	5.638098	0.489998	10.70163
C	5.464	0.139786	9.371277
H	2.084005	-0.16424	2.935777
H	3.772386	1.368903	0.925008
H	7.032751	1.354702	7.851138
H	9.115547	2.051724	6.772531
H	9.310001	2.067993	4.304202
H	7.392408	1.384212	2.91632
H	2.419173	5.539697	1.292742
H	0.411468	6.692051	0.311635
H	-1.85564	5.854969	0.949553
H	-2.032	3.936533	2.525696
H	4.190002	-1.79392	2.924055
H	4.250503	-4.01782	1.876291
H	3.832843	-4.2639	-0.55179
H	3.349899	-2.26038	-1.92519
H	3.29064	-0.02934	-0.8771
H	2.149833	2.58623	0.303642
H	0.33886	2.875818	-1.31532
H	-1.66465	1.421196	-1.22785
H	-1.8152	-0.352	0.497201
H	0.008652	-0.65442	2.119063
H	5.815962	-1.78269	5.433144
H	5.617938	-4.21703	5.71555
H	4.011644	-5.15664	7.349149
H	2.616815	-3.63179	8.716248
H	2.824794	-1.19355	8.445376
H	3.449304	2.67639	8.4063
H	3.762801	3.307929	10.76521
H	5.165468	1.903659	12.24907
H	6.248858	-0.132	11.3473
H	5.93725	-0.76036	8.992525
N	2.215049	1.790852	3.420342
N	4.79399	0.867495	2.60542

H	5.507068	1.454155	2.192115
Ag	0.04861	2.038846	4.4368
C	-1.59368	-0.7566	5.117694
O	-0.34036	-0.47533	4.505326
C	0.663638	-1.18417	5.224061
C	0.211652	-1.16985	6.689336
C	-1.3066	-0.95732	6.614243
H	-2.26126	0.08701	4.926519
H	-2.02473	-1.66783	4.675701
H	0.735688	-2.21345	4.842146
H	1.61932	-0.67754	5.071382
H	0.472982	-2.10409	7.188937
H	0.687508	-0.34599	7.225212
H	-1.85689	-1.8168	7.002232
H	-1.60323	-0.07369	7.18157
C	6.376282	3.504404	0.52993
O	6.684463	2.126765	0.698558
C	6.596852	1.459981	-0.55157
C	5.709106	2.324719	-1.46688
C	5.37575	3.5636	-0.62799
H	5.952428	3.87344	1.467532
H	7.290189	4.071693	0.292537
H	7.601409	1.335852	-0.98379
H	6.164784	0.466846	-0.37827
H	6.248997	2.596628	-2.37691
H	4.803698	1.787502	-1.75704
H	5.475814	4.487895	-1.19893
H	4.355837	3.502074	-0.24254
Ag	2.52081	1.988964	5.93495
O	0.897093	2.21189	7.577619
C	-0.32845	2.417032	7.482111
O	-1.00472	2.409438	6.432093
C	-1.07729	2.707288	8.779718
H	-2.13762	2.501235	8.657153
H	-0.65857	2.109784	9.586069
H	-0.93841	3.761725	9.013658

Group(A)-3

C	2.135822	-0.11184	2.36283
C	2.953112	-1.04545	1.431075
C	5.061761	-0.65911	2.692465
C	6.427479	-0.1127	2.67519
O	4.595833	-0.94545	3.815721
C	6.911754	0.269095	3.939485
C	8.201319	0.795184	4.012442
C	8.981677	0.927723	2.874091
C	8.4853	0.555406	1.630179
C	7.205586	0.036612	1.529486
P	5.762475	0.16792	5.375573
S	3.325363	2.055566	1.601956
C	2.667634	3.64222	1.122922
O	4.097572	2.288876	2.854366
C	2.953645	4.179811	-0.11667
C	2.380016	5.3997	-0.44366
C	1.545307	6.009947	0.482877
C	1.327927	5.394604	1.704984
N	1.897414	4.233292	2.021392
O	4.173447	1.649399	0.473364
C	2.632279	-2.51175	1.643234
C	2.699403	-3.11377	2.897581
C	2.377553	-4.45397	3.050534
C	1.98108	-5.20995	1.956105
C	1.908169	-4.61712	0.703792
C	2.232498	-3.27654	0.549853
C	0.738882	-0.6496	2.558725
C	-0.13545	-0.73112	1.477436
C	-1.41542	-1.23799	1.649531
C	-1.83614	-1.65434	2.907526
C	-0.97385	-1.55647	3.990917
C	0.309653	-1.05814	3.817256
C	5.977591	-1.55266	5.9944
C	7.202089	-2.21749	5.93835
C	7.316099	-3.52027	6.401718
C	6.209109	-4.1737	6.926198
C	4.985857	-3.51969	6.9853

C	4.871332	-2.21823	6.521032
C	6.784806	1.085022	6.646184
C	6.965962	2.458985	6.448512
C	7.601688	3.24624	7.394381
C	8.059478	2.67713	8.576161
C	7.872813	1.320525	8.799313
C	7.239417	0.535166	7.845857
H	2.624878	-0.05066	3.341628
H	2.706596	-0.7789	0.395552
H	8.610086	1.099897	4.968547
H	9.986727	1.326551	2.956922
H	9.097555	0.667643	0.742679
H	6.818572	-0.2628	0.561671
H	3.601664	3.646381	-0.80006
H	2.572009	5.860546	-1.40578
H	1.065539	6.95517	0.260282
H	0.677305	5.834306	2.452852
H	2.997306	-2.53916	3.764105
H	2.43389	-4.90891	4.033378
H	1.727195	-6.25698	2.078699
H	1.596542	-5.19819	-0.15732
H	2.169735	-2.81982	-0.43268
H	0.191441	-0.39733	0.498377
H	-2.08628	-1.31233	0.800455
H	-2.83568	-2.05306	3.040921
H	-1.30083	-1.87231	4.9755
H	0.980137	-0.98597	4.666219
H	8.071448	-1.71669	5.525625
H	8.273291	-4.028	6.35065
H	6.299727	-5.19287	7.285617
H	4.117304	-4.02732	7.390546
H	3.911705	-1.714	6.558493
H	6.595877	2.916002	5.53644
H	7.73456	4.307767	7.214483
H	8.553401	3.290405	9.321907
H	8.220526	0.869334	9.72263
H	7.100555	-0.52231	8.042451
N	1.985463	1.230273	1.802055
N	4.393803	-0.79821	1.550684

H	4.786696	-0.33194	0.743536
Ag	1.225759	2.569459	3.579045
C	-1.89293	3.755988	3.887542
O	-1.01989	3.5703	2.779507
C	-1.63705	2.693558	1.839019
C	-2.75412	1.944542	2.587989
C	-2.62243	2.421718	4.03672
H	-1.28923	4.011616	4.760412
H	-2.60022	4.573266	3.6759
H	-2.04471	3.278014	1.001594
H	-0.86942	2.012531	1.455116
H	-3.73232	2.20829	2.177799
H	-2.62939	0.863682	2.506522
H	-3.58877	2.534556	4.52945
H	-2.00718	1.728199	4.615172
C	4.048236	4.7516	5.1941
O	4.097696	3.646789	6.083866
C	3.316601	3.952128	7.231042
C	2.203474	4.902731	6.757434
C	2.640619	5.338802	5.352467
H	4.815104	5.490946	5.471859
H	4.247531	4.382607	4.184891
H	2.927672	3.013121	7.63306
H	3.948049	4.436468	7.991269
H	1.242396	4.386788	6.718452
H	2.109974	5.755077	7.43345
H	1.971014	4.911414	4.601886
H	2.643243	6.423687	5.234195
Ag	3.619944	1.378693	5.109823
O	1.72847	0.834768	6.340347
C	0.650239	1.449958	6.417736
O	0.245319	2.343149	5.638928
C	-0.28908	1.069559	7.55898
H	-0.81407	1.952306	7.916389
H	-1.0169	0.361188	7.165271
H	0.270086	0.600675	8.364704

Group(A)-4

C	1.777525	0.02713	1.856681
C	2.944181	-0.46769	0.973103
C	4.562057	-0.27088	2.868497
C	5.930508	0.094425	3.272011
O	3.76168	-0.53252	3.79169
C	6.058032	0.450846	4.625485
C	7.314041	0.849275	5.08005
C	8.40887	0.854113	4.229328
C	8.272932	0.47541	2.899688
C	7.029538	0.102149	2.416649
P	4.519818	0.550332	5.630804
S	2.936615	2.421324	1.766962
C	2.265003	3.935025	2.410372
O	4.287238	2.350864	2.336539
C	1.669209	4.869881	1.587839
C	1.149343	6.01327	2.177357
C	1.247765	6.152482	3.555291
C	1.868634	5.158885	4.294853
N	2.379282	4.068333	3.724576
O	2.897315	2.48521	0.308141
C	2.714528	-1.93416	0.666996
C	2.9343	-2.90829	1.637923
C	2.674291	-4.24206	1.359651
C	2.186692	-4.61409	0.114222
C	1.960928	-3.6464	-0.85463
C	2.223869	-2.31201	-0.57941
C	0.483654	-0.08659	1.070479
C	0.130185	0.85449	0.106166
C	-1.0595	0.725026	-0.59554
C	-1.90784	-0.34467	-0.34427
C	-1.56202	-1.2863	0.615229
C	-0.37302	-1.15782	1.317599
C	4.295912	-1.10934	6.391269
C	4.810241	-2.28241	5.840986
C	4.554597	-3.51044	6.432512
C	3.777368	-3.58589	7.580254
C	3.261452	-2.42388	8.138927

C	3.519552	-1.19625	7.548535
C	5.197634	1.402365	7.153487
C	4.833446	2.730217	7.377278
C	5.289076	3.42938	8.484716
C	6.120513	2.803274	9.40247
C	6.485509	1.47776	9.206236
C	6.028553	0.785654	8.094778
H	1.699766	-0.65149	2.713664
H	2.955293	0.102644	0.037543
H	7.449068	1.16328	6.107801
H	9.376152	1.166614	4.605321
H	9.132494	0.481026	2.239465
H	6.918375	-0.1962	1.379553
H	1.620559	4.695997	0.520525
H	0.672445	6.777473	1.574704
H	0.845803	7.025531	4.054381
H	1.963118	5.228147	5.372848
H	3.307107	-2.62642	2.615102
H	2.849001	-4.99377	2.12158
H	1.98074	-5.65674	-0.1003
H	1.577158	-3.93003	-1.82834
H	2.040541	-1.55754	-1.33703
H	0.784761	1.692382	-0.09756
H	-1.32482	1.463742	-1.34402
H	-2.83738	-0.44361	-0.89385
H	-2.22085	-2.12338	0.818933
H	-0.10897	-1.89298	2.069848
H	5.414477	-2.23658	4.942339
H	4.962892	-4.41418	5.993192
H	3.574678	-4.54718	8.039095
H	2.656135	-2.47402	9.037456
H	3.115812	-0.29228	7.992394
H	4.169931	3.22125	6.670691
H	4.991228	4.461445	8.634103
H	6.478636	3.343608	10.27173
H	7.12936	0.980303	9.92352
H	6.31523	-0.25213	7.95985
N	1.909755	1.365328	2.420262
N	4.261133	-0.27302	1.57328

H	4.923914	0.181681	0.96264
Ag	0.045168	1.671493	3.666751
C	-1.62157	-0.7559	5.132253
O	-0.38071	-0.65872	4.442973
C	0.625002	-1.14698	5.326271
C	0.200768	-0.686	6.727738
C	-1.30619	-0.41792	6.596806
H	-2.32347	-0.05693	4.672215
H	-2.01439	-1.77977	5.041467
H	0.666305	-2.24429	5.265854
H	1.585817	-0.73406	5.013268
H	0.418539	-1.45303	7.472762
H	0.729754	0.227569	7.006064
H	-1.89444	-1.03618	7.277092
H	-1.5301	0.630897	6.801164
C	7.088193	3.837695	2.21016
O	7.439175	3.683606	3.574622
C	6.339892	4.092907	4.360487
C	5.713991	5.274075	3.607391
C	6.055817	4.980971	2.141057
H	8.001494	4.063924	1.648448
H	6.65251	2.904389	1.822299
H	5.607265	3.273711	4.458995
H	6.707222	4.364061	5.355514
H	4.638268	5.330996	3.778495
H	6.167495	6.214847	3.930033
H	5.171893	4.66129	1.586509
H	6.471803	5.861856	1.647332
Ag	2.685214	1.969952	4.806229
O	-0.8128	2.963734	5.316591
C	-0.07127	3.085314	6.317375
O	1.084535	2.635519	6.43859
C	-0.65682	3.842253	7.505524
H	-1.31915	4.628847	7.152066
H	-1.23328	3.135655	8.100902
H	0.142835	4.255654	8.115173

Group(A)⁻5

C	1.805113	0.405473	2.625301
C	2.523779	-0.40356	1.512087
C	4.734676	-0.40851	2.648371
C	6.133835	0.048791	2.620111
O	4.336971	-0.91569	3.717141
C	6.727323	0.165636	3.888978
C	8.039727	0.632018	3.955745
C	8.745173	0.938456	2.802266
C	8.1506	0.801277	1.553125
C	6.841124	0.36046	1.461423
P	5.683527	-0.14372	5.372183
S	2.859937	2.767478	2.345407
C	3.535407	3.122164	0.708646
O	2.344179	4.087324	2.742212
C	3.272343	4.355063	0.134143
C	3.769091	4.604977	-1.13514
C	4.499384	3.610494	-1.77163
C	4.700719	2.40623	-1.11657
N	4.222837	2.167782	0.103601
O	3.988786	2.270058	3.164438
C	2.172195	-1.87822	1.492725
C	2.20196	-2.67162	2.636651
C	1.865418	-4.01521	2.567738
C	1.492562	-4.58433	1.358147
C	1.457918	-3.80058	0.213786
C	1.796369	-2.45651	0.282288
C	0.422491	-0.14782	2.878849
C	-0.54309	-0.08791	1.875359
C	-1.81142	-0.60551	2.092036
C	-2.12973	-1.17793	3.318343
C	-1.17634	-1.22298	4.326632
C	0.094677	-0.7106	4.107971
C	5.849017	-1.93918	5.722937
C	6.324746	-2.859	4.789756
C	6.383395	-4.20947	5.099307
C	5.963073	-4.66029	6.343184
C	5.481649	-3.75322	7.277769

C	5.422711	-2.40306	6.96855
C	6.843748	0.515903	6.678437
C	6.682448	1.855342	7.039479
C	7.48075	2.44418	8.00686
C	8.460008	1.694166	8.643638
C	8.63488	0.35979	8.301326
C	7.836816	-0.22308	7.327507
H	2.385136	0.350096	3.557246
H	2.225097	0.033619	0.549585
H	8.524838	0.750725	4.917283
H	9.769555	1.286193	2.876737
H	8.708146	1.038831	0.65423
H	6.375378	0.243311	0.489127
H	2.701076	5.098353	0.674214
H	3.590832	5.558745	-1.61857
H	4.906491	3.766191	-2.76312
H	5.262497	1.601261	-1.58036
H	2.483491	-2.24537	3.589791
H	1.892922	-4.62027	3.467188
H	1.227865	-5.63456	1.307221
H	1.165541	-4.23466	-0.73611
H	1.764664	-1.85004	-0.61742
H	-0.29617	0.364649	0.920936
H	-2.55354	-0.56686	1.30197
H	-3.11775	-1.59304	3.483626
H	-1.4225	-1.6614	5.287337
H	0.834702	-0.74849	4.898927
H	6.648805	-2.51816	3.812961
H	6.757395	-4.91392	4.364177
H	6.007426	-5.71689	6.582864
H	5.147234	-4.09939	8.249617
H	5.039983	-1.69996	7.701096
H	5.916027	2.447563	6.55073
H	7.332836	3.486607	8.26562
H	9.08461	2.146089	9.406332
H	9.398684	-0.2317	8.794924
H	7.988656	-1.26605	7.070804
N	1.615013	1.794446	2.239869
N	3.975925	-0.21798	1.571928

H	4.316394	0.471428	0.901106
Ag	0.377318	2.984588	3.837949
C	-2.84919	2.819878	2.980052
O	-1.93786	3.726259	3.589178
C	-2.44266	4.063056	4.877295
C	-3.10675	2.785048	5.39807
C	-3.52103	2.038773	4.123376
H	-3.59482	3.382139	2.39848
H	-2.28478	2.170539	2.304457
H	-1.60407	4.385277	5.497098
H	-3.17273	4.8815	4.78678
H	-2.38428	2.19724	5.967966
H	-3.95894	3.010954	6.040699
H	-3.17036	1.004841	4.145227
H	-4.60564	2.028695	3.996216
C	4.824951	5.131575	5.157053
O	4.832219	4.578631	6.462817
C	3.511784	4.195486	6.782684
C	2.568469	5.11264	5.982945
C	3.502776	5.9009	5.057993
H	5.70422	5.774641	5.053705
H	4.867638	4.331055	4.401519
H	3.346632	3.138126	6.495104
H	3.378444	4.281751	7.866265
H	1.854581	4.516141	5.407352
H	2.002331	5.773619	6.642327
H	3.135192	5.937616	4.032956
H	3.630985	6.923594	5.422319
Ag	3.584652	1.14166	5.325318
O	1.708713	0.995222	6.582992
C	0.615439	1.563974	6.775554
O	0.056685	2.398219	6.03307
C	-0.13988	1.181597	8.048249
H	-0.60494	2.065976	8.47779
H	-0.91989	0.473234	7.772112
H	0.534563	0.716852	8.763013

Group(A)-6

C	1.705903	0.456392	2.779909
C	2.457642	-0.26228	1.62644
C	4.697702	-0.07056	2.662514
C	6.068894	0.459837	2.589531
O	4.372737	-0.60986	3.741593
C	6.72695	0.520621	3.831183
C	8.016397	1.05021	3.859328
C	8.640528	1.467371	2.693426
C	7.983845	1.383404	1.471159
C	6.693202	0.884161	1.419355
P	5.76139	0.090378	5.337712
S	2.535502	2.889614	2.394472
C	2.986819	3.239436	0.682164
O	1.962666	4.181315	2.812175
C	2.38907	4.316592	0.047101
C	2.704291	4.55187	-1.28118
C	3.598115	3.697641	-1.91387
C	4.136283	2.64215	-1.19571
N	3.831432	2.416444	0.081307
O	3.793351	2.51195	3.083488
C	2.282711	-1.76914	1.617399
C	2.381745	-2.54081	2.772263
C	2.225267	-3.9174	2.712083
C	1.965724	-4.54176	1.500043
C	1.862947	-3.78018	0.344772
C	2.021971	-2.40283	0.404588
C	0.389825	-0.2381	3.050371
C	-0.55359	-0.36428	2.031989
C	-1.74513	-1.03844	2.257954
C	-2.01492	-1.57415	3.511659
C	-1.09289	-1.42168	4.538357
C	0.105665	-0.76007	4.30795
C	5.942304	-1.72987	5.567537
C	6.358513	-2.59157	4.554031
C	6.433967	-3.95798	4.778243
C	6.089357	-4.48324	6.016212
C	5.670057	-3.63398	7.031482

C	5.596344	-2.26761	6.807658
C	7.016895	0.593955	6.64034
C	6.802988	1.782192	7.339681
C	7.67792	2.20651	8.328722
C	8.790378	1.439556	8.644575
C	9.019476	0.250336	7.964772
C	8.142472	-0.16666	6.974874
H	2.318893	0.438441	3.687144
H	2.07236	0.137415	0.678627
H	8.549072	1.135109	4.798938
H	9.64925	1.862981	2.737773
H	8.477273	1.710036	0.562809
H	6.176732	0.815752	0.468416
H	1.704775	4.956115	0.588914
H	2.26336	5.387014	-1.81356
H	3.873347	3.848022	-2.95058
H	4.836059	1.949881	-1.6539
H	2.577725	-2.07172	3.726935
H	2.305873	-4.50532	3.619744
H	1.842206	-5.61809	1.455538
H	1.658041	-4.25805	-0.60706
H	1.939938	-1.81414	-0.50374
H	-0.34819	0.057032	1.053551
H	-2.4626	-1.15205	1.452345
H	-2.9417	-2.10954	3.686013
H	-1.30215	-1.83094	5.520568
H	0.82367	-0.6497	5.111751
H	6.622988	-2.19279	3.581248
H	6.761101	-4.61652	3.980823
H	6.14534	-5.55227	6.189013
H	5.396376	-4.03783	8.000154
H	5.265538	-1.60962	7.6048
H	5.932261	2.384773	7.105049
H	7.488743	3.13497	8.856481
H	9.475715	1.764436	9.4197
H	9.885945	-0.35557	8.207404
H	8.333954	-1.0997	6.45512
N	1.36831	1.830257	2.434941
N	3.885392	0.067108	1.620989

H	4.137508	0.780589	0.936302
Ag	0.293567	2.99044	4.245688
C	-2.82974	2.804953	5.276287
O	-2.14743	3.028987	4.04518
C	-2.82566	2.374178	2.976168
C	-3.89618	1.472694	3.611049
C	-3.53106	1.460266	5.096931
H	-2.09253	2.802521	6.081399
H	-3.56011	3.610436	5.448034
H	-3.27462	3.125163	2.311541
H	-2.09177	1.791269	2.406394
H	-4.89244	1.897246	3.46248
H	-3.87893	0.471766	3.177351
H	-4.40485	1.363224	5.741914
H	-2.83837	0.641559	5.310575
C	4.779759	4.816617	5.160342
O	4.323533	3.949505	6.201246
C	3.2945	4.609351	6.930204
C	2.559518	5.457861	5.895418
C	3.669917	5.854225	4.919416
H	5.714481	5.303282	5.475431
H	4.977125	4.206944	4.274973
H	2.659403	3.851722	7.393238
H	3.739832	5.239079	7.716475
H	1.804701	4.851059	5.386834
H	2.068368	6.320035	6.347946
H	3.320261	5.824227	3.887188
H	4.036643	6.86186	5.131844
Ag	3.831447	1.632206	5.396794
O	1.598611	1.512857	5.929838
C	0.947397	2.052961	6.867417
O	0.113636	2.952853	6.69064
C	1.186929	1.537632	8.279318
H	0.7775	2.232759	9.008175
H	0.686394	0.574854	8.375955
H	2.252151	1.393603	8.44739

Group(A)-7

C	2.480154	0.130016	1.935666
C	3.826791	0.38917	1.219022
C	5.18452	-0.4342	3.118625
C	6.152781	-0.13925	4.189533
O	4.599508	-1.52723	3.142333
C	6.139817	-1.04904	5.264616
C	6.971082	-0.7692	6.349712
C	7.784585	0.353364	6.365633
C	7.815402	1.218041	5.278191
C	6.999186	0.970318	4.187943
P	5.102133	-2.58676	5.102455
S	1.593847	2.566496	2.238794
C	-0.17726	2.341715	2.247922
O	1.835936	3.611413	3.244669
C	-0.94721	2.54423	1.1195
C	-2.31055	2.308845	1.220264
C	-2.82592	1.895005	2.441278
C	-1.96759	1.722807	3.516641
N	-0.65878	1.933021	3.410801
O	1.974002	2.894789	0.868271
C	4.211874	-0.6599	0.195726
C	3.866628	-2.00142	0.3291
C	4.252167	-2.92218	-0.63432
C	4.987672	-2.51561	-1.73847
C	5.339125	-1.18005	-1.87637
C	4.952815	-0.25845	-0.91436
C	1.310834	-0.12047	1.008659
C	1.275872	0.298355	-0.31825
C	0.152996	0.062864	-1.0999
C	-0.94961	-0.58998	-0.56735
C	-0.92418	-1.01002	0.755869
C	0.198688	-0.77766	1.534446
C	5.503267	-3.34263	6.786973
C	6.769515	-3.93241	6.900691
C	7.149586	-4.62583	8.038844
C	6.256154	-4.76991	9.092664
C	4.985874	-4.2217	8.989232

C	4.614632	-3.52245	7.848624
C	3.450971	-1.85191	5.550377
C	3.280307	-0.90811	6.562507
C	2.013025	-0.44827	6.898486
C	0.894991	-0.89883	6.199706
C	1.056518	-1.82734	5.176026
C	2.321279	-2.30067	4.862528
H	2.585306	-0.7504	2.579442
H	3.783018	1.3663	0.72449
H	6.982974	-1.43281	7.206112
H	8.407583	0.551965	7.230859
H	8.469897	2.082092	5.282021
H	7.032306	1.63387	3.331452
H	-0.48273	2.869947	0.197886
H	-2.95873	2.446881	0.362624
H	-3.8858	1.706532	2.559822
H	-2.33109	1.411425	4.489827
H	3.298854	-2.3378	1.187011
H	3.976616	-3.96462	-0.51949
H	5.287146	-3.23739	-2.49009
H	5.91433	-0.85348	-2.73575
H	5.22848	0.785169	-1.02702
H	2.119121	0.820807	-0.75222
H	0.140125	0.395492	-2.132
H	-1.82588	-0.77025	-1.17997
H	-1.78128	-1.51942	1.182715
H	0.211826	-1.10255	2.569151
H	7.469856	-3.8407	6.074728
H	8.141342	-5.06113	8.103168
H	6.546536	-5.31394	9.984834
H	4.276619	-4.33961	9.801964
H	3.613204	-3.10967	7.793668
H	4.141037	-0.53352	7.105958
H	1.890523	0.24654	7.720415
H	-0.09709	-0.56845	6.4875
H	0.189413	-2.19495	4.637781
H	2.43918	-3.03421	4.07249
N	2.214375	1.226363	2.856221
N	4.882144	0.525424	2.231223

H	5.469098	1.340953	2.162762
Ag	1.126929	1.768712	5.096823
C	-0.55746	3.633145	7.380926
O	-0.71489	3.388009	5.985266
C	-0.5449	4.622647	5.299003
C	0.554339	5.348911	6.074528
C	0.313374	4.896303	7.51855
H	-1.54504	3.785262	7.84148
H	-0.08542	2.755084	7.829347
H	-0.26858	4.407724	4.265412
H	-1.48635	5.194231	5.315798
H	1.533871	5.010318	5.728379
H	0.491606	6.431541	5.957352
H	1.25217	4.668945	8.024736
H	-0.21212	5.664264	8.091729
C	4.841033	4.420487	2.0019
O	5.468673	3.584075	2.976449
C	5.974271	4.442809	3.998087
C	4.89269	5.511302	4.170862
C	4.27066	5.623739	2.772002
H	5.590907	4.744135	1.265463
H	4.070225	3.835221	1.497513
H	6.137183	3.846267	4.897506
H	6.925231	4.888308	3.670165
H	4.14727	5.1688	4.892826
H	5.310506	6.456525	4.52019
H	3.182508	5.570305	2.823753
H	4.548846	6.559678	2.282827
Ag	3.862996	2.116197	4.208055
O	4.434236	2.548196	6.337395
C	3.529787	2.723062	7.183738
O	2.303957	2.612332	6.993682
C	3.987449	3.128121	8.58113
H	4.081201	4.213035	8.599402
H	3.251734	2.819329	9.319533
H	4.957197	2.684915	8.794261

(B) Without deprotonation of amide protone

Group(B)-1

C	3.617451	0.772197	-0.0065
C	4.896723	0.430087	0.8048
C	4.421069	-0.83758	2.825257
C	4.29376	-2.12757	3.514094
O	4.227044	0.206538	3.466915
C	3.70036	-2.07961	4.788106
C	3.577049	-3.29049	5.474346
C	4.025015	-4.48408	4.931202
C	4.606891	-4.50984	3.669494
C	4.739431	-3.3293	2.961428
P	3.082938	-0.44573	5.471722
S	1.493739	-0.46338	1.046678
C	-0.06187	0.320197	1.389873
O	1.421252	-1.11827	-0.23647
C	-0.91226	0.642045	0.351489
C	-2.09982	1.286757	0.669717
C	-2.36388	1.562797	2.002925
C	-1.4439	1.189811	2.970237
N	-0.30084	0.575672	2.666273
O	1.836916	-1.26361	2.208222
C	6.08643	0.36498	-0.1242
C	6.216057	-0.67148	-1.0457
C	7.293636	-0.69955	-1.91913
C	8.245081	0.310525	-1.88502
C	8.117104	1.348661	-0.97294
C	7.042391	1.375941	-0.096
C	3.828133	2.099294	-0.70237
C	3.821844	3.283298	0.031138
C	4.059944	4.495177	-0.60002
C	4.311681	4.5333	-1.96478
C	4.323073	3.355016	-2.69812
C	4.082911	2.141258	-2.06927
C	2.157387	-1.17814	6.916652
C	0.99315	-1.88929	6.608074
C	0.197394	-2.43572	7.604441
C	0.543589	-2.26562	8.938903

C	1.690246	-1.55314	9.262906
C	2.487477	-1.01689	8.260998
C	4.672517	0.111321	6.210997
C	5.139111	1.396215	5.940684
C	6.346256	1.839126	6.458542
C	7.10886	0.997097	7.25642
C	6.661053	-0.29012	7.522447
C	5.454355	-0.73345	6.999323
H	3.418045	-0.01457	-0.74129
H	5.049444	1.210888	1.559755
H	3.131983	-3.31154	6.461453
H	3.92451	-5.40235	5.499335
H	4.959033	-5.44334	3.245733
H	5.208941	-3.34138	1.983174
H	-0.64597	0.392766	-0.66739
H	-2.7994	1.567653	-0.10865
H	-3.27462	2.069988	2.29547
H	-1.62202	1.390962	4.019336
H	5.472616	-1.46088	-1.08677
H	7.387052	-1.51126	-2.63195
H	9.084699	0.288941	-2.57038
H	8.856138	2.141337	-0.94357
H	6.942939	2.189964	0.613714
H	3.624061	3.260159	1.096467
H	4.052705	5.412603	-0.0222
H	4.500051	5.480801	-2.45694
H	4.520507	3.379156	-3.76407
H	4.096617	1.221274	-2.64413
H	0.713789	-2.02547	5.567268
H	-0.69596	-2.9915	7.340308
H	-0.07761	-2.68677	9.721589
H	1.967332	-1.41556	10.30259
H	3.381062	-0.46751	8.532409
H	4.554752	2.053671	5.311585
H	6.68674	2.843128	6.232585
H	8.053545	1.338408	7.665559
H	7.256524	-0.9571	8.136912
H	5.128849	-1.74833	7.201913
N	2.467164	0.872763	0.878024

H	2.682477	1.238358	1.799703
N	4.73709	-0.8222	1.519904
H	4.693733	-1.67212	0.980937
Ag	0.256035	3.037448	5.410954
O	2.115364	1.54901	6.586756
C	2.20741	2.512402	7.395228
O	1.507409	3.540254	7.319227
C	3.19513	2.421427	8.544935
H	2.809572	1.711501	9.275021
H	4.156832	2.066788	8.183221
H	3.304498	3.396174	9.014298
C	-2.25442	2.592636	7.439161
O	-1.6564	1.805002	6.415226
C	-1.14379	0.620748	7.02213
C	-0.78871	0.986305	8.473039
C	-1.35564	2.398686	8.663781
H	-2.29489	3.627705	7.092864
H	-3.2756	2.232029	7.635952
H	-1.90597	-0.17132	6.989336
H	-0.27143	0.301221	6.447473
H	-1.23843	0.274013	9.168248
H	0.29178	0.971517	8.62304
H	-1.91715	2.499116	9.593589
H	-0.54915	3.135232	8.657507
C	4.636271	5.416807	6.355797
O	4.864043	4.594488	5.221801
C	3.687804	4.507251	4.435869
C	2.912049	5.797653	4.705675
C	3.272542	6.102895	6.160918
H	4.642545	4.804847	7.270105
H	5.453833	6.148165	6.422814
H	3.977379	4.391726	3.386348
H	3.088887	3.633273	4.735964
H	3.256922	6.597911	4.044689
H	1.839042	5.662441	4.56363
H	3.331485	7.175479	6.354749
H	2.531903	5.668063	6.835762
Ag	1.675251	0.93038	3.9469
O	1.758724	2.921017	2.66127

C	0.789494	3.696784	2.506504
O	-0.12628	3.892882	3.3289
C	0.723381	4.468502	1.192978
H	0.976641	3.803823	0.369527
H	-0.26923	4.88964	1.054344
H	1.457161	5.271953	1.23284

Group(B)-2

C	2.707761	0.465461	0.162513
C	4.003016	0.62969	1.006871
C	4.503317	-0.57102	3.065453
C	4.555366	-1.82898	3.819695
O	4.70607	0.490229	3.673178
C	4.622627	-1.67761	5.216977
C	4.672961	-2.84122	5.987035
C	4.664925	-4.09558	5.398542
C	4.589161	-4.22641	4.016227
C	4.532151	-3.09179	3.226926
P	4.534922	0.031387	5.934836
S	1.222709	-0.72972	2.009537
C	-0.55212	-0.80017	1.9792
O	1.725713	-1.9391	1.390913
C	-1.19986	-1.94148	1.553283
C	-2.58845	-1.92281	1.552261
C	-3.23738	-0.76711	1.962905
C	-2.48844	0.327833	2.365854
N	-1.15689	0.30642	2.37246
O	1.623958	-0.43638	3.377285
C	5.191661	0.938001	0.123276
C	5.863878	-0.08031	-0.54694
C	6.93221	0.217513	-1.38055
C	7.334401	1.534515	-1.55444
C	6.6682	2.553357	-0.88742
C	5.602106	2.256174	-0.05163
C	2.634732	1.505998	-0.9334
C	2.155941	2.790683	-0.69531
C	2.15212	3.734068	-1.713
C	2.62975	3.406882	-2.97389

C	3.106493	2.126203	-3.21766
C	3.107163	1.180615	-2.20316
C	4.393313	-0.44708	7.734289
C	3.195939	-1.0557	8.128458
C	2.943631	-1.35602	9.457804
C	3.880383	-1.03482	10.43184
C	5.060129	-0.4035	10.06372
C	5.309934	-0.10863	8.730468
C	6.323674	0.482844	5.845949
C	6.714466	1.704127	5.301633
C	8.054518	2.053507	5.230776
C	9.027687	1.180058	5.696482
C	8.652264	-0.04764	6.224914
C	7.310904	-0.3941	6.297478
H	2.706869	-0.53231	-0.29217
H	3.862424	1.440188	1.733209
H	4.727966	-2.77355	7.066897
H	4.719336	-4.98114	6.022282
H	4.578227	-5.2093	3.559036
H	4.480566	-3.19099	2.14782
H	-0.63336	-2.80866	1.240202
H	-3.14873	-2.79406	1.234083
H	-4.31899	-0.71369	1.972072
H	-2.95268	1.253132	2.686126
H	5.555556	-1.11146	-0.41123
H	7.450962	-0.58235	-1.89726
H	8.168059	1.766362	-2.20753
H	6.980067	3.583626	-1.01727
H	5.082188	3.054235	0.467454
H	1.779968	3.063107	0.282673
H	1.776674	4.732037	-1.51579
H	2.630187	4.147194	-3.76589
H	3.480271	1.861434	-4.20046
H	3.485208	0.182213	-2.39696
H	2.444409	-1.28582	7.380148
H	2.00979	-1.83235	9.736845
H	3.685598	-1.26407	11.47395
H	5.789809	-0.13322	10.81987
H	6.228792	0.403602	8.468519

H	5.963545	2.380492	4.912041
H	8.341448	3.009316	4.805465
H	10.07647	1.450965	5.641149
H	9.40727	-0.74086	6.580254
H	7.033048	-1.36117	6.703294
N	1.52575	0.568062	1.010075
H	1.529549	1.406475	1.596732
N	4.223344	-0.59816	1.753826
H	3.91348	-1.46104	1.334012
Ag	-0.04093	1.693953	3.914156
O	0.919089	0.83197	7.009678
C	-0.31474	0.978451	6.979798
O	-1.00162	1.320781	5.992863
C	-1.07216	0.740651	8.286354
H	-1.12781	1.691036	8.815568
H	-2.07988	0.390554	8.075871
H	-0.53494	0.021998	8.900637
C	-1.31798	4.570842	3.069037
O	-1.97868	3.376824	3.47015
C	-2.58494	3.643107	4.72971
C	-1.59518	4.548459	5.477082
C	-0.74747	5.177873	4.360699
H	-0.54624	4.307057	2.343211
H	-2.04083	5.257113	2.601168
H	-3.54739	4.155847	4.577061
H	-2.75599	2.691544	5.236151
H	-2.11854	5.300593	6.070307
H	-0.97049	3.952319	6.145121
H	-0.82455	6.266785	4.350105
H	0.303593	4.907838	4.479716
C	3.222367	2.464197	8.454079
O	4.094352	2.406806	7.33309
C	4.381315	3.73963	6.932033
C	3.14187	4.579299	7.286318
C	2.309826	3.668908	8.198906
H	2.66794	1.525317	8.508035
H	3.805642	2.597984	9.378997
H	5.271337	4.108106	7.465896
H	4.581654	3.739779	5.85825

H	3.431307	5.503809	7.791199
H	2.585782	4.839048	6.38361
H	2.028614	4.161296	9.131636
H	1.400059	3.345416	7.688765
Ag	2.48041	1.288182	5.309714
O	2.726345	3.430565	4.343588
C	2.345123	3.650464	3.186615
O	1.461828	3.003757	2.562947
C	3.005259	4.802974	2.431878
H	3.455589	5.500668	3.133749
H	3.783841	4.383084	1.795839
H	2.272998	5.307754	1.805801

Group(B)-3

C	2.842512	0.641239	-0.16398
C	4.035299	0.608184	0.836327
C	3.993645	-0.47329	3.010817
C	3.602709	-1.60697	3.860681
O	4.354435	0.572532	3.5782
C	3.402569	-1.27256	5.210128
C	2.996794	-2.28377	6.08013
C	2.808074	-3.58031	5.625375
C	3.010361	-3.89647	4.286059
C	3.404644	-2.90761	3.400832
P	3.6837	0.488243	5.728176
S	0.712561	-0.07152	1.219109
C	-0.72174	0.844464	1.717373
O	0.36522	-1.03326	0.200424
C	-1.94747	0.619913	1.124181
C	-3.0256	1.351689	1.605186
C	-2.81159	2.25115	2.639963
C	-1.5337	2.401084	3.160797
N	-0.50302	1.704089	2.693144
O	1.321983	-0.60002	2.434706
C	5.374059	0.611269	0.133834
C	5.955402	-0.57412	-0.30764
C	7.175999	-0.55083	-0.96724
C	7.822836	0.656075	-1.19365

C	7.248437	1.840526	-0.75292
C	6.030352	1.818302	-0.09054
C	3.134139	1.537146	-1.34917
C	2.720828	2.864899	-1.36726
C	3.027667	3.680156	-2.44744
C	3.748768	3.175291	-3.51984
C	4.150359	1.846312	-3.51486
C	3.841557	1.0302	-2.43706
C	3.063452	0.397191	7.486505
C	1.710187	0.113837	7.696369
C	1.13815	0.214995	8.954405
C	1.907364	0.626418	10.03505
C	3.246584	0.93608	9.842572
C	3.81612	0.82699	8.581783
C	5.502141	0.391688	6.045573
C	6.058678	-0.6593	6.77559
C	7.424124	-0.70764	7.013931
C	8.253325	0.293355	6.525601
C	7.710231	1.340587	5.794486
C	6.345618	1.385698	5.555006
H	2.650719	-0.37521	-0.52934
H	3.97213	1.484607	1.493757
H	2.83223	-2.06571	7.128484
H	2.50459	-4.35362	6.32233
H	2.861724	-4.91167	3.936606
H	3.570306	-3.15024	2.356659
H	-2.04849	-0.10413	0.326316
H	-4.01362	1.214963	1.181021
H	-3.63148	2.832109	3.044046
H	-1.32293	3.081739	3.977767
H	5.455426	-1.52002	-0.12831
H	7.622123	-1.47921	-1.30628
H	8.775487	0.673188	-1.71079
H	7.751484	2.785646	-0.92373
H	5.58333	2.744789	0.253261
H	2.14462	3.260942	-0.54002
H	2.701651	4.714272	-2.4491
H	3.992201	3.813622	-4.3616
H	4.705588	1.442002	-4.35389

H	4.156313	-0.0079	-2.43894
H	1.096092	-0.18137	6.85332
H	0.087417	-0.01901	9.090961
H	1.462406	0.715027	11.02008
H	3.852233	1.269449	10.6786
H	4.861653	1.082861	8.450818
H	5.421803	-1.44766	7.162887
H	7.841865	-1.5306	7.583886
H	9.320577	0.255115	6.714348
H	8.351802	2.125213	5.40875
H	5.927363	2.200813	4.976408
N	1.635232	1.117818	0.507011
H	1.836753	1.827156	1.222162
N	3.908249	-0.57473	1.680515
H	3.421271	-1.36913	1.293196
Ag	1.444054	1.420465	4.219354
O	1.604663	3.587318	7.058062
C	0.440879	3.200303	6.837425
O	0.046755	2.539916	5.853828
C	-0.60189	3.547772	7.902405
H	-0.40657	4.540538	8.30117
H	-1.60469	3.491637	7.485452
H	-0.50327	2.819551	8.706819
C	-1.47013	-0.16873	5.600981
O	-0.29876	-0.45836	4.858524
C	-0.32272	-1.82683	4.499495
C	-0.97808	-2.54747	5.687112
C	-1.80076	-1.45181	6.383102
H	-1.25955	0.684311	6.249828
H	-2.29652	0.094797	4.920524
H	-0.91039	-1.97163	3.579173
H	0.70395	-2.15305	4.320433
H	-1.6004	-3.37894	5.35194
H	-0.21236	-2.93964	6.360931
H	-2.871	-1.66504	6.347703
H	-1.50661	-1.35306	7.430229
C	4.438963	4.451411	8.201329
O	5.091693	3.979045	7.026416
C	5.567324	5.119246	6.320049

C	4.483369	6.188	6.504189
C	3.791764	5.792126	7.817295
H	3.706227	3.70127	8.506498
H	5.179905	4.586612	9.003707
H	6.525769	5.448252	6.750065
H	5.712132	4.837145	5.275354
H	4.916038	7.189064	6.549711
H	3.778965	6.150159	5.670624
H	3.949615	6.537226	8.600041
H	2.71781	5.66754	7.669382
Ag	3.338181	3.011702	5.585578
O	3.588611	4.380886	3.734658
C	3.048901	4.034107	2.670256
O	2.35243	3.009499	2.480922
C	3.27598	4.950242	1.464689
H	2.32965	5.125334	0.957388
H	3.710733	5.893034	1.787953
H	3.959296	4.452259	0.778016

Group(B)-4

C	2.912902	1.217698	0.822128
C	3.737018	0.775383	2.047391
C	3.370554	0.043832	4.310915
C	2.861188	-1.00439	5.2038
O	4.167269	0.878596	4.748827
C	2.923636	-0.74845	6.584806
C	2.514037	-1.76301	7.448814
C	2.06184	-2.98197	6.966974
C	2.004991	-3.22139	5.59958
C	2.410359	-2.23334	4.719693
P	3.51598	0.894602	7.187317
S	0.763044	2.534129	0.061377
C	-0.73706	2.865734	0.952797
O	1.192815	3.767066	-0.55732
C	-1.38105	4.069367	0.75574
C	-2.5613	4.284303	1.454237
C	-3.01955	3.282862	2.296891
C	-2.29068	2.11091	2.420796

N	-1.15424	1.896928	1.752805
O	0.576306	1.366999	-0.76645
C	4.833837	-0.18359	1.633545
C	4.510681	-1.44319	1.131949
C	5.512654	-2.32787	0.762044
C	6.846099	-1.96293	0.889146
C	7.173506	-0.71111	1.389677
C	6.171294	0.173783	1.760999
C	3.832574	1.889524	-0.176
C	4.380585	1.136646	-1.21063
C	5.269095	1.715304	-2.10501
C	5.616606	3.052556	-1.97435
C	5.067279	3.810037	-0.94963
C	4.182194	3.230809	-0.05204
C	3.205777	0.85476	9.019011
C	1.997469	0.479775	9.613549
C	1.82345	0.543406	10.98826
C	2.84591	1.002718	11.806
C	4.043135	1.407092	11.23212
C	4.215883	1.335374	9.858518
C	5.344084	0.466538	7.261851
C	5.838853	-0.80963	7.531074
C	7.204219	-1.05705	7.544247
C	8.103646	-0.03034	7.290904
C	7.628816	1.247342	7.028856
C	6.263188	1.486754	7.014544
H	2.44052	0.340764	0.364829
H	4.166872	1.661762	2.527745
H	2.566926	-1.61632	8.520476
H	1.763329	-3.7568	7.6645
H	1.661527	-4.17833	5.22325
H	2.40747	-2.42815	3.652507
H	-0.96296	4.810903	0.088012
H	-3.10327	5.216101	1.345707
H	-3.93126	3.411821	2.866903
H	-2.61568	1.315392	3.08131
H	3.471148	-1.73773	1.030854
H	5.250452	-3.30538	0.372982
H	7.628512	-2.65513	0.599794

H	8.212894	-0.42119	1.494435
H	6.432161	1.149079	2.154411
H	4.110894	0.091339	-1.31684
H	5.689644	1.119148	-2.90712
H	6.310448	3.50509	-2.67366
H	5.327486	4.857476	-0.84631
H	3.746655	3.837945	0.733577
H	1.170362	0.141361	9.002952
H	0.877687	0.236332	11.42208
H	2.708621	1.053029	12.88053
H	4.847971	1.780405	11.85632
H	5.160251	1.66031	9.435627
H	5.152539	-1.62546	7.732628
H	7.567523	-2.05807	7.752015
H	9.170373	-0.22613	7.297769
H	8.322063	2.05776	6.83176
H	5.900747	2.488787	6.804162
N	1.844813	2.090041	1.297067
H	2.200089	2.951655	1.700844
N	2.885277	0.118057	3.036538
H	2.378135	-0.68943	2.69994
Ag	0.556231	1.389394	3.333707
O	1.288993	1.425973	7.074393
C	0.2418	1.279914	6.38994
O	0.114352	0.519406	5.406711
C	-0.95264	2.146971	6.776313
H	-0.9724	2.999936	6.097781
H	-1.87434	1.582282	6.652705
H	-0.85504	2.502025	7.79934
C	-0.54433	-1.87447	3.348051
O	0.265989	-1.14625	2.430681
C	-0.27783	-1.33015	1.129584
C	-1.79848	-1.45461	1.316561
C	-1.97722	-1.76906	2.809467
H	-0.21986	-2.92578	3.378899
H	-0.42292	-1.4289	4.337007
H	0.136817	-2.24471	0.678899
H	0.000963	-0.47023	0.517408
H	-2.29389	-0.51737	1.054494

H	-2.20442	-2.24549	0.683203
H	-2.51608	-0.96321	3.313011
H	-2.52598	-2.69876	2.969265
C	6.703606	3.410878	4.060899
O	6.473874	4.337016	5.107096
C	5.307729	5.067883	4.814822
C	5.123337	5.047086	3.285653
C	6.244295	4.128093	2.786101
H	7.76853	3.161305	4.053326
H	6.116163	2.493021	4.22353
H	4.427089	4.592688	5.30269
H	5.417379	6.078694	5.222177
H	4.140097	4.646671	3.028076
H	5.207358	6.049077	2.861196
H	5.895775	3.424623	2.028543
H	7.063665	4.714624	2.363058
Ag	3.171355	2.911948	5.800846
O	2.078467	3.528036	3.895037
C	1.025531	4.241827	3.918138
O	-0.1069	3.799837	3.708899
C	1.204886	5.728393	4.195823
H	1.886701	6.147707	3.45783
H	0.245374	6.237591	4.147658
H	1.64273	5.856326	5.184357

Group(B)-5

C	2.589038	-0.10409	1.052108
C	3.431505	0.552798	2.163818
C	4.048878	0.094071	4.435377
C	4.080057	-0.75783	5.627774
O	4.529963	1.234817	4.487211
C	4.739425	-0.20241	6.742128
C	4.767372	-0.97033	7.908511
C	4.184936	-2.22663	7.96485
C	3.537845	-2.75614	6.853627
C	3.484306	-2.0189	5.684872
P	5.408251	1.546801	6.60405
S	0.496862	-1.70697	0.774411

C	-0.4383	-0.7448	-0.4028
O	1.371268	-2.5754	0.033727
C	-0.13669	-0.82135	-1.74791
C	-0.85906	-0.01052	-2.61238
C	-1.82797	0.827764	-2.08026
C	-2.05408	0.825445	-0.71234
N	-1.36249	0.044555	0.115178
O	-0.45762	-2.28623	1.703365
C	4.806754	0.894779	1.634806
C	5.810746	-0.0686	1.613389
C	7.065498	0.243738	1.113026
C	7.322022	1.516851	0.622002
C	6.321142	2.478525	0.637347
C	5.06654	2.169491	1.142746
C	2.197276	0.919259	0.009753
C	1.323247	1.956135	0.329679
C	0.959771	2.885587	-0.63277
C	1.474122	2.794785	-1.91929
C	2.357045	1.772858	-2.2391
C	2.717266	0.837789	-1.27854
C	6.181001	1.603766	8.333495
C	5.297786	1.91478	9.37437
C	5.741152	2.109139	10.67418
C	7.09648	2.012448	10.9642
C	7.991632	1.713007	9.946557
C	7.536994	1.511394	8.649001
C	6.938369	1.107434	5.637568
C	7.372885	1.964339	4.626526
C	8.494149	1.664287	3.86753
C	9.196753	0.488919	4.096691
C	8.766838	-0.38233	5.088192
C	7.647791	-0.07533	5.850077
H	3.161176	-0.91816	0.591723
H	2.93104	1.469006	2.509164
H	5.259342	-0.58711	8.79469
H	4.233264	-2.8001	8.884384
H	3.076338	-3.7358	6.902485
H	2.967116	-2.4211	4.820288
H	0.63727	-1.49205	-2.09693

H	-0.66157	-0.03034	-3.67753
H	-2.40645	1.481982	-2.72046
H	-2.79643	1.469897	-0.25729
H	5.610952	-1.06016	2.005119
H	7.847209	-0.5075	1.110883
H	8.303385	1.760721	0.231195
H	6.518702	3.475378	0.25908
H	4.287347	2.924089	1.161224
H	0.920593	2.035816	1.332852
H	0.272627	3.684005	-0.37612
H	1.189184	3.521783	-2.67132
H	2.765875	1.70062	-3.24065
H	3.405562	0.038865	-1.53195
H	4.236841	2.003883	9.155574
H	5.031841	2.341536	11.46177
H	7.451657	2.170768	11.9767
H	9.051962	1.634324	10.16376
H	8.259563	1.273723	7.875735
H	6.816828	2.871287	4.42078
H	8.816178	2.345027	3.086203
H	10.07106	0.249184	3.500865
H	9.303648	-1.30805	5.267946
H	7.320735	-0.7745	6.612633
N	1.430052	-0.70387	1.701005
H	0.838607	-0.04469	2.207173
N	3.530851	-0.37545	3.283987
H	2.896772	-1.1611	3.292989
Ag	-1.75839	0.19165	2.452026
O	-2.88705	-2.12017	4.795621
C	-3.79427	-1.38159	4.366452
O	-3.67642	-0.47273	3.517474
C	-5.19055	-1.62529	4.928044
H	-5.12269	-1.83113	5.993648
H	-5.82805	-0.7647	4.741907
H	-5.6035	-2.49964	4.427382
C	-1.94221	3.471931	2.548889
O	-2.76632	2.497346	1.918823
C	-4.02989	2.528233	2.577348
C	-3.71731	2.79637	4.056085

C	-2.3207	3.435443	4.036581
H	-0.89833	3.202873	2.373921
H	-2.14188	4.463416	2.114458
H	-4.64794	3.333818	2.152545
H	-4.52216	1.567148	2.416875
H	-4.4641	3.45554	4.502314
H	-3.70511	1.858071	4.613762
H	-2.32155	4.438949	4.466017
H	-1.60752	2.822312	4.590799
C	7.569401	4.45266	7.046009
O	6.309407	3.962506	6.620741
C	5.286226	4.764029	7.182651
C	5.822945	5.253101	8.536918
C	7.344462	5.070638	8.435942
H	8.270189	3.612748	7.066347
H	7.938078	5.20872	6.335638
H	5.059316	5.612073	6.518475
H	4.393959	4.135098	7.27936
H	5.547757	6.294431	8.714132
H	5.418335	4.646821	9.350298
H	7.874122	6.02067	8.529319
H	7.70526	4.39907	9.218084
Ag	-0.73518	-1.96647	4.149499
O	0.819705	-0.41938	4.832051
C	0.746839	0.774299	4.480481
O	0.124077	1.195373	3.469447
C	1.476829	1.81302	5.315103
H	2.173758	2.361188	4.684243
H	0.740113	2.513096	5.705837
H	2.010336	1.338124	6.134295

Group(B)-6

C	2.729582	1.227528	1.938774
C	4.052366	0.681642	2.495818
C	4.710732	-0.14332	4.676946
C	4.324773	-0.96462	5.860302
O	5.82361	0.388392	4.574713
C	3.185855	-0.64633	6.612771

C	2.866838	-1.48361	7.681964
C	3.657319	-2.57848	8.002018
C	4.803883	-2.85486	7.269806
C	5.14116	-2.04011	6.200548
P	2.219997	0.871838	6.14243
S	0.803647	2.909124	2.48454
C	0.134989	3.426463	4.049441
O	1.036694	4.107096	1.710207
C	0.646183	4.569568	4.63189
C	0.083503	4.986448	5.830096
C	-0.95535	4.239486	6.367968
C	-1.38143	3.096415	5.710625
N	-0.83793	2.692111	4.562713
O	-0.06138	1.897487	1.89658
C	4.719455	-0.23285	1.491923
C	4.274336	-1.54263	1.336932
C	4.868395	-2.37573	0.400646
C	5.908707	-1.90536	-0.38927
C	6.354619	-0.59981	-0.23853
C	5.762283	0.234176	0.698774
C	2.916969	1.891266	0.591554
C	2.13351	1.482339	-0.48327
C	2.283278	2.076033	-1.72801
C	3.218768	3.084932	-1.90877
C	4.003453	3.49646	-0.84053
C	3.855399	2.902654	0.404067
C	0.941558	0.819398	7.499991
C	-0.38367	0.507339	7.194753
C	-1.37265	0.506072	8.168065
C	-1.05652	0.825959	9.480666
C	0.256059	1.138031	9.807682
C	1.240128	1.132697	8.830668
C	3.350492	2.119764	6.989083
C	4.718002	1.947897	7.233206
C	5.518264	3.012791	7.64865
C	4.965417	4.273331	7.836436
C	3.607288	4.458255	7.617864
C	2.821491	3.398467	7.19415
H	2.01326	0.401283	1.844047

H	4.723307	1.517633	2.739615
H	1.988563	-1.27862	8.282452
H	3.380986	-3.21306	8.83696
H	5.431115	-3.6998	7.529893
H	6.030475	-2.23748	5.612274
H	1.472988	5.093336	4.169583
H	0.447911	5.876095	6.329156
H	-1.42634	4.531318	7.298296
H	-2.17994	2.48279	6.110774
H	3.464616	-1.91349	1.956533
H	4.517106	-3.39531	0.287022
H	6.372265	-2.55709	-1.12121
H	7.167659	-0.228	-0.85196
H	6.110142	1.254841	0.810654
H	1.3966	0.698395	-0.34346
H	1.665521	1.750524	-2.55771
H	3.335053	3.550797	-2.88094
H	4.735821	4.284676	-0.9742
H	4.475362	3.233003	1.227287
H	-0.65724	0.257563	6.176313
H	-2.39302	0.254119	7.899382
H	-1.8267	0.830466	10.24399
H	0.516523	1.386012	10.83108
H	2.258349	1.379567	9.110376
H	5.170019	0.967567	7.142867
H	6.569558	2.842324	7.851904
H	5.588223	5.100828	8.156322
H	3.159787	5.435087	7.768612
H	1.76284	3.562002	7.020877
N	2.19761	2.140623	2.957048
H	2.860377	2.869244	3.255233
N	3.775503	-0.03389	3.729055
H	2.850067	-0.42043	3.874987
Ag	-1.10045	0.51813	3.65503
O	1.041296	-0.61006	4.67125
C	0.421566	-1.71283	4.601037
O	-0.7342	-1.81175	4.165183
C	1.136131	-2.96665	5.072066
H	0.592793	-3.85059	4.747654

H	2.152106	-2.98041	4.681837
H	1.183331	-2.94746	6.159989
C	-3.0061	-1.52922	2.037683
O	-2.91476	-0.10787	2.079689
C	-2.55891	0.312505	0.767198
C	-1.54812	-0.73193	0.272976
C	-1.87661	-1.9848	1.100504
H	-2.8969	-1.911	3.053563
H	-3.98898	-1.8204	1.638235
H	-2.14369	1.319436	0.827292
H	-3.45547	0.322421	0.129436
H	-1.64967	-0.90556	-0.79961
H	-0.5289	-0.39393	0.474181
H	-2.19995	-2.81699	0.47251
H	-1.00956	-2.30505	1.681466
C	8.743011	1.64044	4.616109
O	8.21608	2.94052	4.854887
C	8.613033	3.753218	3.754787
C	8.521395	2.841272	2.522648
C	8.6214	1.420381	3.100187
H	9.79611	1.606407	4.933733
H	8.163848	0.922268	5.199307
H	7.939944	4.610141	3.701378
H	9.64507	4.102126	3.909514
H	7.564474	2.987782	2.017351
H	9.323477	3.054033	1.813568
H	7.722192	0.844241	2.872037
H	9.48578	0.878831	2.711175
Ag	5.740554	2.73887	4.76876
O	5.646527	4.391514	2.983069
C	4.481204	4.739568	3.257683
O	3.766837	4.153175	4.113203
C	3.867532	5.908018	2.504816
H	3.485641	6.633274	3.221101
H	4.607363	6.372483	1.857589
H	3.034966	5.538031	1.90846

(C) Deprotonation of both amide protone

Group(C)-1

C	2.130692	1.650363	1.129529
C	3.445863	0.8687	1.395889
C	3.778318	-0.4432	3.323158
C	3.535766	-1.65987	4.132207
O	4.453518	0.454369	3.93556
C	3.577139	-1.43215	5.514421
C	3.339378	-2.51158	6.362574
C	3.108039	-3.77956	5.841027
C	3.073407	-3.98716	4.466974
C	3.278778	-2.92011	3.605865
P	3.793547	0.331901	6.017525
S	0.283849	2.740866	2.646809
C	-0.95016	1.751354	1.782173
O	-0.00308	2.579415	4.081386
C	-1.89778	2.367393	0.987092
C	-2.81197	1.567103	0.319994
C	-2.72389	0.190004	0.476431
C	-1.73065	-0.33626	1.284969
N	-0.85451	0.437415	1.926752
O	0.097118	4.083406	2.117403
C	4.103951	0.428628	0.112476
C	3.735691	-0.76424	-0.50446
C	4.328683	-1.1487	-1.69797
C	5.293609	-0.34346	-2.2882
C	5.667115	0.845701	-1.67686
C	5.075823	1.229393	-0.48203
C	2.298271	2.73422	0.089546
C	2.52195	4.065399	0.417828
C	2.693257	5.0197	-0.57443
C	2.641459	4.654077	-1.91169
C	2.398343	3.329676	-2.25105
C	2.22342	2.378527	-1.25761
C	3.128817	0.185726	7.782086
C	3.765638	-0.46391	8.84447
C	3.176075	-0.53333	10.09853
C	1.937448	0.052585	10.32319
C	1.295867	0.717627	9.287123

C	1.8927	0.779188	8.035863
C	5.577699	0.456488	6.459894
C	6.566683	-0.23071	5.756408
C	7.904626	-0.08155	6.087576
C	8.280161	0.763782	7.122763
C	7.30627	1.454134	7.831159
C	5.96727	1.299928	7.503713
H	1.395372	0.927598	0.741278
H	4.124257	1.532789	1.948788
H	3.346821	-2.38221	7.438413
H	2.94881	-4.61258	6.517041
H	2.886489	-4.9796	4.072341
H	3.254689	-3.05446	2.530017
H	-1.90718	3.446516	0.903193
H	-3.5758	2.00577	-0.31147
H	-3.41618	-0.47264	-0.02822
H	-1.62711	-1.40693	1.425104
H	2.987964	-1.39678	-0.03825
H	4.036242	-2.08042	-2.17039
H	5.755814	-0.64348	-3.22217
H	6.421992	1.477224	-2.13259
H	5.368264	2.160592	-0.00794
H	2.527739	4.365084	1.455995
H	2.86457	6.054887	-0.29988
H	2.778865	5.398746	-2.68788
H	2.342346	3.036022	-3.29375
H	2.026805	1.346977	-1.53145
H	4.743072	-0.91043	8.694676
H	3.688065	-1.0449	10.90647
H	1.478776	-0.00034	11.30435
H	0.338685	1.198612	9.457424
H	1.392651	1.312404	7.23089
H	6.289229	-0.88786	4.941316
H	8.659466	-0.62725	5.531936
H	9.327215	0.883272	7.378008
H	7.588739	2.114992	8.643501
H	5.21658	1.833865	8.07687
N	1.726942	2.103154	2.453363
N	3.127004	-0.32082	2.19299

Ag	1.044543	-0.20358	3.194833
C	-1.07616	0.203882	5.642697
O	-0.12903	-0.78638	5.227898
C	-0.11648	-1.9035	6.113297
C	-0.93547	-1.48514	7.334834
C	-1.88834	-0.43091	6.770761
H	-0.53444	1.092859	5.988988
H	-1.69211	0.482907	4.781375
H	-0.56346	-2.77285	5.608242
H	0.92206	-2.13866	6.372506
H	-1.46238	-2.33201	7.775909
H	-0.28318	-1.0427	8.092559
H	-2.79125	-0.90416	6.375648
H	-2.17878	0.307168	7.519144
C	3.546185	4.959804	3.96776
O	3.568638	4.180669	5.159641
C	4.935306	4.020453	5.512098
C	5.661373	3.78529	4.183629
C	4.784698	4.525313	3.162589
H	2.60271	4.763353	3.455735
H	3.60538	6.027091	4.22616
H	5.299843	4.933324	6.007133
H	5.017771	3.178618	6.201145
H	5.691378	2.716862	3.956847
H	6.682885	4.166941	4.211451
H	5.294793	5.392258	2.738268
H	4.506804	3.858095	2.344955
Ag	2.429865	2.015848	4.741211

Group(C)-2

C	1.90156	1.714105	1.079128
C	3.368519	1.255961	1.349857
C	3.425586	-0.21139	3.219046
C	3.436564	-1.58249	3.764895
O	3.4759	0.697394	4.17071
C	3.390981	-1.69863	5.161126
C	3.351736	-2.98009	5.710882
C	3.385926	-4.10268	4.895276

C	3.446192	-3.97082	3.512128
C	3.47001	-2.70726	2.946428
P	3.208901	-0.14748	6.13223
S	-0.10219	1.691464	2.734199
C	-0.16647	-0.10443	2.834744
O	-0.28513	2.172754	4.124673
C	-0.46302	-0.82962	1.693258
C	-0.39827	-2.21254	1.758153
C	-0.05443	-2.80266	2.967124
C	0.20725	-1.99437	4.060178
N	0.159244	-0.66381	3.990834
O	-1.15972	2.039076	1.801351
C	4.140629	1.164907	0.055013
C	4.091952	6.58E-05	-0.70656
C	4.765836	-0.0787	-1.91585
C	5.494921	1.008185	-2.37983
C	5.548554	2.172858	-1.62641
C	4.874031	2.249914	-0.41646
C	1.870288	2.858018	0.090793
C	2.070665	4.169219	0.513812
C	2.092663	5.209673	-0.40292
C	1.914992	4.949782	-1.75511
C	1.708228	3.64578	-2.18408
C	1.686501	2.605842	-1.26621
C	3.096799	-0.92676	7.857501
C	4.193285	-1.45825	8.544407
C	4.052362	-1.99546	9.815089
C	2.807796	-2.01543	10.43104
C	1.70678	-1.49312	9.767376
C	1.858915	-0.95515	8.498136
C	4.899912	0.546361	6.38696
C	5.99762	0.161193	5.618307
C	7.244451	0.728046	5.837408
C	7.410168	1.694699	6.820822
C	6.321815	2.088576	7.588874
C	5.076939	1.516209	7.373627
H	1.35	0.855533	0.674
H	3.833012	2.00172	2.0159
H	3.296446	-3.11439	6.784562

H	3.361733	-5.08968	5.344071
H	3.469225	-4.85308	2.882182
H	3.504429	-2.57103	1.870625
H	-0.74739	-0.31334	0.785783
H	-0.61145	-2.81875	0.885415
H	0.013034	-3.87917	3.06359
H	0.473568	-2.41931	5.022303
H	3.528128	-0.84967	-0.3366
H	4.72138	-0.99142	-2.50036
H	6.020848	0.947966	-3.32623
H	6.115858	3.025923	-1.98289
H	4.910482	3.167702	0.161219
H	2.197028	4.377739	1.57107
H	2.249275	6.2272	-0.06074
H	1.934219	5.762419	-2.47285
H	1.564674	3.437432	-3.23876
H	1.530538	1.587489	-1.60696
H	5.172995	-1.45005	8.0781
H	4.917985	-2.40079	10.32798
H	2.697907	-2.43403	11.42531
H	0.730439	-1.50086	10.23985
H	0.993496	-0.53977	7.988543
H	5.875879	-0.58525	4.842167
H	8.090773	0.415824	5.23531
H	8.384558	2.139666	6.988833
H	6.443639	2.843505	8.357862
H	4.231421	1.827032	7.978874
N	1.363585	2.125645	2.357582
N	3.339964	-0.06468	1.952101
Ag	0.973266	0.807128	5.715846
C	-1.82431	2.130398	6.805522
O	-1.05389	0.972045	7.126424
C	-1.91	-0.15735	7.013855
C	-2.82945	0.146491	5.825861
C	-2.88706	1.679504	5.78975
H	-2.29396	2.519632	7.720783
H	-1.14768	2.883611	6.397419
H	-1.2901	-1.04469	6.860823
H	-2.48999	-0.2764	7.941683

H	-2.39252	-0.24114	4.902815
H	-3.81421	-0.30351	5.960383
H	-2.65029	2.053026	4.792794
H	-3.87342	2.050953	6.074789
C	4.511175	5.533834	3.694416
O	4.352519	4.319127	4.415065
C	5.562798	3.570076	4.309221
C	6.223295	3.987865	2.985088
C	5.388324	5.176633	2.490583
H	3.520456	5.896673	3.410105
H	5.003818	6.282412	4.333272
H	6.21284	3.80095	5.165417
H	5.30464	2.508864	4.331191
H	7.26447	4.273055	3.149222
H	6.203679	3.168702	2.264096
H	6.008607	6.019706	2.183847
H	4.763135	4.879877	1.645056
Ag	2.4455	2.855979	4.178218

Group(C)-3

C	2.864679	1.294985	0.764311
C	3.084575	-0.2522	0.823541
C	4.178418	-0.53691	2.951568
C	5.453706	-0.69925	3.680952
O	3.192985	-0.26815	3.78045
C	5.463422	-0.22398	5.000006
C	6.661603	-0.28814	5.709581
C	7.798145	-0.83536	5.130326
C	7.771212	-1.30374	3.82161
C	6.596802	-1.22708	3.091504
P	3.924325	0.589095	5.607345
S	1.230557	2.963191	1.860746
C	-0.37402	2.312579	1.371905
O	0.986784	3.419111	3.24331
C	-1.07646	2.849951	0.310161
C	-2.31201	2.299337	0.003679
C	-2.78255	1.247427	0.778727
C	-1.9994	0.772725	1.819211

N	-0.80862	1.296134	2.098851
O	1.528793	3.992488	0.870465
C	1.815033	-1.04611	1.071508
C	1.784053	-2.13025	1.951154
C	0.643045	-2.91072	2.081754
C	-0.48778	-2.63274	1.326757
C	-0.4618	-1.57292	0.429543
C	0.676775	-0.79199	0.3015
C	4.190079	1.968178	0.472155
C	5.11469	2.212207	1.4829
C	6.346361	2.778668	1.193672
C	6.6635	3.124727	-0.11332
C	5.742843	2.895756	-1.12647
C	4.516634	2.313861	-0.83606
C	2.915126	-0.73204	6.406916
C	2.974121	-2.06916	6.017801
C	2.17605	-3.02238	6.633074
C	1.307785	-2.65525	7.653825
C	1.238888	-1.32525	8.0499
C	2.029781	-0.3726	7.425061
C	4.61469	1.293555	7.228932
C	4.832598	2.669281	7.293587
C	5.327911	3.275953	8.438387
C	5.612651	2.506852	9.557824
C	5.398869	1.135174	9.519542
C	4.907584	0.537168	8.368456
H	2.182657	1.495764	-0.07408
H	3.406834	-0.52209	-0.19606
H	6.721429	0.086572	6.724461
H	8.716689	-0.88784	5.704736
H	8.667446	-1.71906	3.374317
H	6.547591	-1.56705	2.062609
H	-0.65896	3.6775	-0.24875
H	-2.89968	2.684719	-0.82165
H	-3.74559	0.795163	0.576019
H	-2.32561	-0.05101	2.445506
H	2.668743	-2.39092	2.517805
H	0.646685	-3.7524	2.766212
H	-1.3761	-3.24756	1.420801

H	-1.33163	-1.35772	-0.18206
H	0.677276	0.019393	-0.41847
H	4.872767	1.952187	2.507749
H	7.066185	2.941344	1.989023
H	7.625424	3.570809	-0.34109
H	5.982277	3.167392	-2.14901
H	3.80557	2.130577	-1.63535
H	3.650728	-2.3688	5.226421
H	2.237241	-4.0592	6.320526
H	0.691951	-3.4022	8.142514
H	0.571794	-1.0309	8.853272
H	1.968097	0.663585	7.741894
H	4.600905	3.278876	6.424062
H	5.487491	4.348742	8.458637
H	5.996417	2.974048	10.45809
H	5.61692	0.527837	10.39157
H	4.743752	-0.53542	8.357731
N	2.285242	1.789084	2.000133
N	4.206324	-0.59185	1.674436
Ag	0.922639	0.426803	3.557812
C	-1.41213	0.134759	6.066286
O	-0.87987	-0.5525	4.936526
C	-1.10938	-1.95279	5.034263
C	-2.33688	-2.09587	5.932652
C	-2.21598	-0.89476	6.873254
H	-0.59031	0.556407	6.659911
H	-2.044	0.954468	5.701557
H	-1.27261	-2.34128	4.024073
H	-0.23304	-2.45081	5.475991
H	-3.25192	-2.02722	5.338765
H	-2.33604	-3.04479	6.469867
H	-3.19145	-0.50198	7.163348
H	-1.67247	-1.17378	7.779242
C	3.763695	5.212748	2.650042
O	4.288533	4.474022	3.760115
C	5.583555	4.936111	4.128374
C	5.998599	5.94858	3.056952
C	4.659425	6.440977	2.507791
H	3.804384	4.587103	1.749644

H	2.721499	5.46596	2.863625
H	5.52938	5.407395	5.121155
H	6.271117	4.08304	4.180057
H	6.60256	6.753982	3.476966
H	6.571774	5.45296	2.268896
H	4.279984	7.269435	3.11202
H	4.731855	6.767075	1.469745
Ag	3.012358	2.462155	4.265788

Group(C)-4

C	2.709518	1.067622	1.390553
C	3.749298	-0.06989	1.195655
C	3.526205	-0.9869	3.417565
C	4.275795	-1.58487	4.59427
O	2.261019	-1.09196	3.436135
C	4.706682	-0.85055	5.707438
C	5.362468	-1.52264	6.742659
C	5.586478	-2.88849	6.680878
C	5.150865	-3.61184	5.579248
C	4.49508	-2.95953	4.54688
P	4.396575	0.962052	5.748543
S	2.53666	3.06166	2.975009
C	0.876848	3.197766	2.306054
O	2.333395	2.413974	4.307519
C	0.58329	4.216399	1.417769
C	-0.67518	4.234685	0.837688
C	-1.57217	3.22733	1.169403
C	-1.18905	2.251651	2.074491
N	0.019894	2.242044	2.635245
O	3.069523	4.408781	3.061939
C	3.223879	-1.19582	0.326679
C	2.759109	-2.41721	0.804448
C	2.267052	-3.38017	-0.06709
C	2.237069	-3.14613	-1.43341
C	2.728273	-1.94488	-1.92649
C	3.221416	-0.98676	-1.05521
C	2.056707	1.492482	0.097073
C	2.623346	2.495456	-0.6871

C	2.007105	2.902962	-1.8604
C	0.816938	2.3127	-2.26536
C	0.248012	1.309733	-1.49211
C	0.864875	0.902298	-0.31847
C	2.810926	0.999562	6.678567
C	1.911958	-0.07168	6.634943
C	0.662111	0.031772	7.237529
C	0.294545	1.201423	7.890691
C	1.183745	2.265464	7.943301
C	2.426664	2.169563	7.335668
C	5.621243	1.496539	7.004946
C	6.872519	1.895602	6.52925
C	7.864108	2.327937	7.395298
C	7.612879	2.38289	8.760352
C	6.373168	1.992487	9.248523
C	5.386519	1.547727	8.380578
H	1.926884	0.703731	2.066009
H	4.592536	0.378256	0.651294
H	5.704697	-0.96939	7.610221
H	6.099702	-3.38768	7.495244
H	5.321655	-4.6812	5.525287
H	4.150869	-3.51973	3.684285
H	1.331236	4.966214	1.194468
H	-0.9491	5.012226	0.134109
H	-2.56094	3.196956	0.728602
H	-1.85855	1.44685	2.358573
H	2.784946	-2.64664	1.859551
H	1.905028	-4.32263	0.330511
H	1.846626	-3.8976	-2.1107
H	2.730549	-1.75371	-2.99418
H	3.612575	-0.05804	-1.4569
H	3.544409	2.965337	-0.36036
H	2.45483	3.687464	-2.46077
H	0.332556	2.634856	-3.18047
H	-0.68222	0.845536	-1.80169
H	0.413894	0.120958	0.283975
H	2.197398	-1.00475	6.164408
H	-0.01659	-0.81396	7.208851
H	-0.68103	1.279463	8.357394

H	0.905523	3.181516	8.451975
H	3.10288	3.014158	7.371802
H	7.065956	1.866536	5.45993
H	8.830329	2.628803	7.00608
H	8.381285	2.731004	9.441226
H	6.172559	2.032202	10.31338
H	4.42554	1.24256	8.778463
N	3.416642	2.209045	1.970395
N	4.301843	-0.49356	2.484281
Ag	0.767461	0.500924	4.126924
C	-1.90239	-1.58663	4.353756
O	-1.20123	-0.89046	3.333046
C	-0.63777	-1.87124	2.463406
C	-0.24523	-3.05156	3.362943
C	-1.0761	-2.85026	4.638674
H	-1.98961	-0.92733	5.221448
H	-2.9098	-1.85054	3.99757
H	-1.38653	-2.17375	1.716608
H	0.221518	-1.42634	1.958167
H	-0.46608	-4.00348	2.877052
H	0.821751	-3.0163	3.589634
H	-1.72437	-3.70301	4.847556
H	-0.42462	-2.69415	5.501526
C	3.824424	5.28735	5.851693
O	4.118129	5.070932	7.224473
C	5.471426	5.395766	7.486246
C	6.204637	5.44289	6.130525
C	5.138692	5.036646	5.110053
H	3.035099	4.592699	5.555931
H	3.476541	6.320227	5.691673
H	5.536299	6.367781	7.999673
H	5.888582	4.626207	8.148964
H	6.573312	6.451981	5.929918
H	7.057184	4.760441	6.118414
H	5.197332	5.610459	4.185392
H	5.231801	3.974425	4.861289
Ag	5.082226	1.480295	3.408398

Group(C)-5

C	2.274614	0.851037	1.41226
C	3.791797	0.596462	1.55928
C	3.552343	-0.78135	3.532154
C	4.278524	-1.29788	4.760157
O	2.423318	-1.25969	3.284426
C	3.774465	-1.11116	6.053045
C	4.450361	-1.69089	7.132042
C	5.594072	-2.44727	6.928587
C	6.097507	-2.61563	5.645002
C	5.440928	-2.03985	4.568337
P	2.350301	0.016386	6.284269
S	2.17075	2.982565	2.908758
C	0.686869	3.617237	3.672584
O	3.181664	3.095766	4.006963
C	0.270862	4.902931	3.373213
C	-0.89531	5.369214	3.957478
C	-1.59293	4.522037	4.80921
C	-1.10219	3.247686	5.041008
N	0.023679	2.803594	4.483483
O	2.47206	3.86121	1.779471
C	4.313836	-0.41861	0.562252
C	3.697595	-1.65542	0.382959
C	4.22563	-2.58258	-0.50331
C	5.381296	-2.29396	-1.21644
C	6.013268	-1.07247	-1.0305
C	5.483991	-0.14512	-0.14445
C	1.880673	1.598949	0.149531
C	2.758829	1.951335	-0.86903
C	2.303002	2.632502	-1.99118
C	0.96416	2.969654	-2.11358
C	0.077465	2.619927	-1.1027
C	0.533429	1.941054	0.014498
C	1.671363	-0.55234	7.888999
C	1.281711	-1.89255	7.993301
C	0.662013	-2.36531	9.138383
C	0.409026	-1.50414	10.19869
C	0.782437	-0.17079	10.10482

C	1.405115	0.302806	8.959251
C	3.215065	1.56973	6.741127
C	4.604599	1.676091	6.874807
C	5.20065	2.910739	7.109503
C	4.42157	4.055306	7.215064
C	3.042755	3.961527	7.087286
C	2.448451	2.732824	6.84365
H	1.755381	-0.11427	1.377785
H	4.309037	1.549966	1.393909
H	4.077806	-1.54916	8.140646
H	6.099199	-2.89664	7.776347
H	6.998586	-3.19743	5.486163
H	5.822438	-2.16631	3.560904
H	0.854195	5.509245	2.69192
H	-1.25676	6.370468	3.752645
H	-2.51079	4.844142	5.285606
H	-1.62316	2.555999	5.695032
H	2.804429	-1.90665	0.942021
H	3.732546	-3.5398	-0.63585
H	5.79135	-3.02029	-1.90959
H	6.92256	-0.84111	-1.57511
H	5.991373	0.803755	0.002164
H	3.810916	1.703245	-0.80821
H	3.004819	2.901795	-2.77364
H	0.611746	3.502735	-2.98987
H	-0.97285	2.879009	-1.18669
H	-0.16047	1.678823	0.80666
H	1.479649	-2.57131	7.170144
H	0.373651	-3.40864	9.20394
H	-0.0779	-1.87198	11.09471
H	0.588831	0.506757	10.92923
H	1.693242	1.346759	8.903052
H	5.224859	0.787891	6.840873
H	6.277768	2.972252	7.216558
H	4.889453	5.016846	7.392969
H	2.427731	4.85169	7.158291
H	1.374496	2.677542	6.702364
N	1.717432	1.519109	2.574632
N	4.237185	0.133558	2.884041

Ag	0.808918	0.429745	4.411755
C	-1.33143	-2.09896	5.128272
O	-0.95075	-1.24521	4.05662
C	-0.5989	-2.05064	2.933193
C	0.044343	-3.30439	3.52669
C	-0.65692	-3.46151	4.880443
H	-1.00502	-1.63756	6.066458
H	-2.42673	-2.20145	5.146806
H	-1.50412	-2.30018	2.358871
H	0.088485	-1.47913	2.308066
H	-0.09886	-4.17328	2.883131
H	1.114812	-3.14087	3.666941
H	-1.40545	-4.25692	4.851571
H	0.057433	-3.69629	5.671982
C	5.933481	4.811219	3.674977
O	6.503814	3.548341	3.324823
C	6.779465	3.55937	1.926701
C	5.657503	4.387697	1.297166
C	5.248524	5.35689	2.412142
H	5.228167	4.646895	4.493605
H	6.731578	5.486925	4.016691
H	7.762321	4.022801	1.750131
H	6.800362	2.525348	1.573618
H	5.999881	4.90571	0.400184
H	4.813872	3.74747	1.030394
H	5.585534	6.374782	2.203277
H	4.164541	5.367712	2.531554
Ag	5.129322	1.725881	4.128992

Group(C)-6

C	1.72996	2.07998	1.895442
C	3.016717	1.283819	1.538915
C	3.289423	-0.48243	3.114391
C	4.030212	-1.16984	4.256124
O	2.469488	-1.23375	2.531299
C	4.296264	-0.7323	5.567817
C	4.870329	-1.64557	6.464403
C	5.200395	-2.93845	6.09975

C	4.967646	-3.35802	4.79884
C	4.375817	-2.48204	3.908089
P	4.08113	1.009629	6.125136
S	-0.65474	1.726326	2.730739
C	-1.39372	1.277632	1.13765
O	-1.35625	0.850719	3.674513
C	-2.00786	2.2195	0.333734
C	-2.52216	1.805518	-0.88539
C	-2.39868	0.467397	-1.23689
C	-1.76358	-0.40372	-0.36637
N	-1.26257	0.002583	0.798748
O	-0.89824	3.159475	2.850678
C	2.903182	0.263726	0.422118
C	1.79066	0.138281	-0.4024
C	1.779067	-0.78197	-1.44334
C	2.884792	-1.58175	-1.68558
C	4.011575	-1.44674	-0.88368
C	4.021576	-0.52893	0.152817
C	2.130578	3.378141	2.571318
C	1.996877	3.549403	3.952931
C	2.376171	4.747324	4.555378
C	2.87997	5.786721	3.785601
C	2.996814	5.629151	2.408953
C	2.634145	4.431981	1.808669
C	2.397115	1.148401	6.810023
C	1.302968	0.745311	6.036534
C	0.008707	1.001504	6.463407
C	-0.21819	1.667526	7.659431
C	0.858768	2.102376	8.418285
C	2.154897	1.86113	7.989602
C	5.174743	1.109816	7.59485
C	6.391492	1.775592	7.435051
C	7.280075	1.902866	8.492044
C	6.961677	1.369422	9.73297
C	5.753837	0.707358	9.90922
C	4.867543	0.575638	8.851122
H	1.235894	2.352147	0.949406
H	3.727905	2.038855	1.16343
H	5.072497	-1.33988	7.483289

H	5.642095	-3.60822	6.828915
H	5.231751	-4.36162	4.485708
H	4.166836	-2.81461	2.897267
H	-2.07641	3.246361	0.669754
H	-3.0103	2.509258	-1.55019
H	-2.78838	0.101534	-2.17907
H	-1.64612	-1.45494	-0.60829
H	0.915394	0.755479	-0.24913
H	0.895517	-0.86909	-2.06759
H	2.873068	-2.30214	-2.49601
H	4.886962	-2.06011	-1.06963
H	4.90485	-0.43209	0.77612
H	1.506519	2.775818	4.532414
H	2.25075	4.872541	5.625354
H	3.159417	6.725231	4.251308
H	3.360048	6.45032	1.79974
H	2.72184	4.323803	0.732047
H	1.461521	0.254343	5.082727
H	-0.82708	0.710143	5.840917
H	-1.23183	1.866776	7.987578
H	0.693942	2.646659	9.341838
H	2.982669	2.24377	8.575961
H	6.641931	2.200705	6.467789
H	8.220766	2.42233	8.347368
H	7.65221	1.470485	10.5626
H	5.500169	0.288749	10.87681
H	3.928917	0.055181	9.004581
N	0.861849	1.274237	2.729908
N	3.645937	0.728824	2.746953
Ag	0.15126	-0.98405	2.429737
C	-1.17456	-2.3514	5.191467
O	-0.31181	-2.70292	4.113352
C	0.845856	-3.36787	4.599571
C	1.05816	-2.83622	6.01784
C	-0.35976	-2.49864	6.488209
H	-1.51624	-1.32398	5.030099
H	-2.04668	-3.02054	5.195389
H	0.676581	-4.45589	4.608076
H	1.679477	-3.13436	3.931967

H	1.544228	-3.57416	6.657702
H	1.676993	-1.93577	5.995029
H	-0.76612	-3.30347	7.105901
H	-0.37703	-1.57664	7.072486
C	6.426207	4.807341	3.039009
O	6.380087	3.398137	3.256003
C	6.858706	2.729105	2.093161
C	6.426092	3.613406	0.924164
C	6.479631	5.025068	1.516495
H	5.530983	5.249782	3.487886
H	7.316396	5.224009	3.530785
H	7.954363	2.640481	2.13994
H	6.411795	1.732626	2.067246
H	7.08318	3.497581	0.06192
H	5.404845	3.36506	0.624054
H	7.405631	5.532069	1.235903
H	5.639606	5.632473	1.176086
Ag	4.396911	2.329131	4.141064

(D) Deprotonation of amide protone

Group(D)-1

C	1.65125	1.568734	0.850512
C	2.749248	1.137861	1.84167
C	3.124209	-0.29389	3.599988
C	2.912653	-1.47989	4.449636
O	4.041526	0.488489	4.020767
C	3.420237	-1.34588	5.75101
C	3.27197	-2.42036	6.626037
C	2.678576	-3.60113	6.199536
C	2.191611	-3.72265	4.901695
C	2.300554	-2.65359	4.024156
P	4.106185	0.310256	6.194915
S	-0.95872	1.986129	0.728387
C	-1.59816	0.327491	0.860464
O	-1.84569	2.873335	1.436769
C	-2.16195	-0.25557	-0.25754
C	-2.71527	-1.52027	-0.11594
C	-2.68072	-2.12036	1.134741

C	-2.08591	-1.45141	2.192359
N	-1.54455	-0.24088	2.053258
O	-0.70214	2.215523	-0.67365
C	4.015183	0.817244	1.080237
C	4.143297	-0.41418	0.443191
C	5.292678	-0.71694	-0.27089
C	6.323838	0.209245	-0.35609
C	6.201077	1.437992	0.277494
C	5.050821	1.740282	0.991837
C	2.138237	2.742356	0.031098
C	2.343008	3.982875	0.631129
C	2.835442	5.044763	-0.11269
C	3.132186	4.875689	-1.45821
C	2.930781	3.641174	-2.05944
C	2.436276	2.578149	-1.3175
C	4.089125	0.099555	8.071663
C	3.037098	0.710378	8.756345
C	2.912654	0.608944	10.13383
C	3.855077	-0.10566	10.8606
C	4.916513	-0.711	10.20107
C	5.030797	-0.60983	8.821973
C	5.920356	0.173144	5.949635
C	6.750226	1.057846	6.641141
C	8.122483	1.043952	6.441481
C	8.685407	0.145673	5.544622
C	7.8682	-0.73495	4.848455
C	6.496003	-0.71867	5.045776
H	1.389442	0.722588	0.204913
H	2.928217	1.971604	2.53861
H	3.634525	-2.3524	7.64514
H	2.594464	-4.43615	6.886448
H	1.734426	-4.65078	4.577261
H	1.930865	-2.72682	3.007072
H	-2.163	0.270368	-1.20336
H	-3.16702	-2.02198	-0.96347
H	-3.10927	-3.10259	1.290858
H	-2.03929	-1.88474	3.18455
H	3.341273	-1.14076	0.521521
H	5.385459	-1.67962	-0.76204

H	7.22326	-0.02797	-0.91347
H	7.004586	2.163941	0.216818
H	4.958004	2.701471	1.485284
H	2.118163	4.125604	1.682768
H	2.991329	6.007043	0.362469
H	3.519619	5.706004	-2.03802
H	3.159591	3.504249	-3.11055
H	2.279129	1.614095	-1.78931
H	2.304766	1.283779	8.193232
H	2.084099	1.091001	10.64157
H	3.765855	-0.18653	11.93836
H	5.658917	-1.26636	10.76429
H	5.868225	-1.08518	8.322013
H	6.317548	1.762639	7.343866
H	8.754118	1.738143	6.985305
H	9.758186	0.134653	5.38649
H	8.302454	-1.43619	4.144022
H	5.864764	-1.40063	4.48815
N	0.463624	1.922884	1.629581
N	2.326689	-0.05026	2.586941
Ag	0.272945	0.212344	3.531601
O	0.390771	2.133143	4.982526
C	0.398821	3.267268	4.418473
O	1.442099	3.844347	4.067899
C	-0.93561	3.94906	4.180581
H	-1.71912	3.209916	4.033975
H	-1.16752	4.541696	5.064892
H	-0.87304	4.608352	3.31832
C	-1.76152	0.111035	6.056413
O	-1.18207	-0.93127	5.273352
C	-0.60701	-1.86173	6.181216
C	-0.03116	-1.01897	7.327343
C	-0.83876	0.285704	7.271738
H	-2.77425	-0.18449	6.368888
H	-1.81788	1.008971	5.438784
H	-1.38587	-2.546	6.551793
H	0.151405	-2.43918	5.647545
H	-0.1379	-1.53139	8.284859
H	1.030321	-0.82342	7.159669

H	-1.41911	0.444998	8.182288
H	-0.17954	1.143123	7.126483
C	3.566929	5.574734	5.533453
O	4.260524	4.37641	5.868443
C	5.441545	4.328531	5.079836
C	5.03279	4.900758	3.718132
C	3.880394	5.857077	4.054043
H	2.502327	5.410463	5.711654
H	3.922201	6.395995	6.17368
H	6.226803	4.943033	5.546188
H	5.77715	3.289896	5.027953
H	5.86581	5.406123	3.22736
H	4.680094	4.097593	3.067159
H	4.168155	6.900821	3.911316
H	3.008322	5.650547	3.431817
Ag	2.90248	2.332404	5.393928
H	0.564234	2.831403	2.073327

Group(D)-2

C	1.154152	0.997908	2.151217
C	2.469699	0.195961	1.900482
C	2.426427	-1.13511	3.808437
C	2.283459	-2.4121	4.525506
O	2.643039	-0.12618	4.614709
C	2.394192	-2.35468	5.923088
C	2.304482	-3.55139	6.632904
C	2.077867	-4.75196	5.974442
C	1.946005	-4.78785	4.59087
C	2.052579	-3.61427	3.86381
P	2.760748	-0.72344	6.698432
S	0.216662	3.245639	3.168971
C	0.793145	3.922554	4.720941
O	-0.04437	4.383373	2.314414
C	-0.01568	4.878461	5.307651
C	0.389782	5.406464	6.524395
C	1.575655	4.949793	7.082388
C	2.314267	3.988556	6.412219
N	1.920953	3.483709	5.242046

O	-0.86676	2.349331	3.481519
C	2.793791	0.044868	0.436038
C	2.459609	-1.12338	-0.24312
C	2.731907	-1.24966	-1.5968
C	3.340077	-0.20887	-2.28575
C	3.679241	0.957159	-1.61328
C	3.408559	1.082975	-0.25839
C	0.221465	0.923203	0.967487
C	0.183457	1.896077	-0.02561
C	-0.65756	1.745411	-1.11886
C	-1.4599	0.619321	-1.23438
C	-1.42057	-0.35844	-0.24939
C	-0.58382	-0.2073	0.845491
C	1.161819	0.187345	6.801261
C	0.079354	-0.04627	5.954618
C	-1.0629	0.735592	6.034818
C	-1.14511	1.760238	6.967286
C	-0.07874	1.99526	7.824877
C	1.066454	1.218179	7.737701
C	2.780252	-1.26872	8.506522
C	4.004024	-1.2743	9.175128
C	4.098996	-1.63133	10.51218
C	2.955919	-1.98897	11.21302
C	1.725619	-1.98683	10.56845
C	1.640361	-1.63136	9.230872
H	0.663242	0.555427	3.023871
H	3.281912	0.774473	2.385456
H	2.407167	-3.55742	7.711482
H	2.003497	-5.66915	6.548345
H	1.76698	-5.73006	4.085266
H	1.965198	-3.61014	2.782535
H	-0.92905	5.193023	4.820023
H	-0.20956	6.15756	7.025104
H	1.927	5.334356	8.031601
H	3.245003	3.605736	6.814334
H	1.985343	-1.93388	0.299182
H	2.465994	-2.16326	-2.11739
H	3.55046	-0.30663	-3.34504
H	4.155959	1.773329	-2.14498

H	3.673748	1.997396	0.258966
H	0.808736	2.778891	0.0446
H	-0.68152	2.511228	-1.88632
H	-2.11411	0.502728	-2.09118
H	-2.04331	-1.24218	-0.33455
H	-0.54774	-0.97347	1.612803
H	0.129225	-0.8406	5.21941
H	-1.88733	0.55326	5.355437
H	-2.03514	2.376959	7.023543
H	-0.13382	2.793818	8.557469
H	1.900243	1.417318	8.403809
H	4.901375	-0.98594	8.631856
H	5.063375	-1.62646	11.0085
H	3.022218	-2.26541	12.2595
H	0.828258	-2.26278	11.11176
H	0.672363	-1.63009	8.740502
N	1.519732	2.366269	2.533229
N	2.351962	-1.10354	2.525991
Ag	3.465574	2.0281	4.10828
O	5.410813	3.280039	4.396048
C	5.844796	3.419848	5.560426
O	5.537989	2.716742	6.546622
C	6.841534	4.546867	5.797924
H	7.842375	4.137319	5.668603
H	6.685443	5.343446	5.074596
H	6.741576	4.921898	6.813541
C	5.853291	-1.74456	3.991075
O	6.154616	-0.45939	4.521855
C	6.884888	0.275289	3.545794
C	6.346662	-0.19056	2.186924
C	5.734809	-1.56981	2.468997
H	6.659795	-2.44935	4.244944
H	4.922329	-2.09134	4.447933
H	7.958897	0.053129	3.639987
H	6.719168	1.339942	3.723746
H	5.583953	0.500851	1.821291
H	7.145196	-0.24067	1.444878
H	4.688358	-1.60337	2.157408
H	6.272754	-2.36361	1.947335

C	4.351717	4.52464	1.602044
O	3.158798	4.281901	0.872633
C	2.454202	5.509513	0.76482
C	2.709262	6.246851	2.088318
C	4.024736	5.64582	2.603619
H	4.643826	3.594119	2.096644
H	5.15867	4.831799	0.918634
H	2.835332	6.093618	-0.0878
H	1.397718	5.282982	0.603522
H	2.78157	7.324676	1.93359
H	1.895546	6.052985	2.791037
H	4.826208	6.386499	2.631756
H	3.903329	5.238278	3.609174
Ag	4.792691	0.582866	6.280051
H	1.865997	2.917302	1.749801

Group(D)-3

C	2.830069	0.845004	1.548082
C	4.050527	0.410145	2.418631
C	2.85954	-0.62134	4.163343
C	2.728606	-1.05768	5.574644
O	2.154386	-1.2372	3.306621
C	2.961326	-0.37424	6.753005
C	3.289934	-1.0899	7.913942
C	3.688999	-2.45222	7.817585
C	2.993698	-3.12296	6.892795
C	2.355306	-2.49998	5.724539
P	2.895728	1.458563	6.917744
S	1.20288	2.995096	2.287183
C	-0.54396	2.660699	2.164438
O	1.432937	3.664027	3.564045
C	-1.43533	3.665882	2.479111
C	-2.78748	3.384587	2.343152
C	-3.15837	2.126279	1.889444
C	-2.17856	1.190507	1.601737
N	-0.8766	1.452779	1.74271
O	1.593587	3.701762	1.090063
C	4.653576	-0.89651	1.929007

C	4.624888	-1.25872	0.583822
C	5.192649	-2.45319	0.159466
C	5.80784	-3.29877	1.070262
C	5.865813	-2.93265	2.408118
C	5.298668	-1.74112	2.831373
C	3.226373	1.67764	0.351244
C	4.180747	2.689911	0.422808
C	4.528514	3.41228	-0.70823
C	3.927946	3.134849	-1.92832
C	2.972042	2.132565	-2.00954
C	2.625331	1.409589	-0.87755
C	1.736945	1.71725	8.321292
C	0.776604	0.77724	8.69485
C	-0.10198	1.041802	9.735283
C	-0.04238	2.25386	10.41027
C	0.89626	3.20547	10.03409
C	1.776059	2.939839	8.995415
C	4.524188	1.613066	7.845079
C	4.689811	1.685569	9.229485
C	5.951892	1.797898	9.796482
C	7.081496	1.833748	8.991729
C	6.939176	1.760354	7.612107
C	5.675431	1.656534	7.054527
H	2.340754	-0.06286	1.17415
H	4.832512	1.176063	2.334429
H	3.234395	-0.61886	8.889851
H	4.265723	-2.92269	8.602415
H	1.273428	-2.62453	5.852279
H	2.604804	-3.05259	4.813158
H	-1.07786	4.624738	2.830638
H	-3.53293	4.131727	2.588479
H	-4.20314	1.867614	1.768997
H	-2.4285	0.193706	1.255925
H	4.168472	-0.60903	-0.15376
H	5.152003	-2.72046	-0.89117
H	6.245786	-4.23363	0.738695
H	6.355499	-3.57975	3.127736
H	5.353958	-1.46631	3.878515
H	4.648453	2.934188	1.369007

H	5.271602	4.199308	-0.63426
H	4.201416	3.700825	-2.81174
H	2.493032	1.91227	-2.95724
H	1.871893	0.631356	-0.9474
H	0.709913	-0.1649	8.163562
H	-0.83935	0.298648	10.01849
H	-0.73012	2.459367	11.22302
H	0.941937	4.15836	10.55013
H	2.501835	3.691671	8.702032
H	3.824414	1.654653	9.882203
H	6.052172	1.854798	10.87516
H	8.067105	1.919881	9.435578
H	7.814068	1.788011	6.971266
H	5.574322	1.605017	5.972907
N	1.846478	1.477658	2.438354
N	3.739929	0.324672	3.844639
Ag	0.054341	-0.23529	3.198278
O	-1.02988	1.131534	4.748783
C	-0.21734	1.609978	5.559167
O	0.97162	1.208128	5.687034
C	-0.70997	2.75767	6.428557
H	0.098086	3.458161	6.625917
H	-1.53505	3.26116	5.929466
H	-1.0648	2.348046	7.373225
C	-0.76998	-3.2285	2.154789
O	-0.83889	-1.92694	1.581361
C	-0.00912	-1.9314	0.424418
C	1.166912	-2.86606	0.746532
C	0.67221	-3.70405	1.933418
H	-1.03786	-3.14914	3.210866
H	-1.48583	-3.89291	1.647164
H	-0.58256	-2.30272	-0.43842
H	0.303939	-0.90231	0.229046
H	1.420037	-3.48469	-0.11655
H	2.050486	-2.29153	1.031229
H	0.705546	-4.7749	1.72562
H	1.280661	-3.50047	2.816601
C	4.033317	5.290951	3.142107
O	4.68579	4.226433	3.83959

C	6.099588	4.274392	3.676351
C	6.371674	5.359589	2.631462
C	5.141031	6.261763	2.734714
H	3.296866	5.745092	3.813054
H	3.514224	4.887218	2.26349
H	6.564498	4.522449	4.641901
H	6.457049	3.289114	3.353936
H	6.43813	4.918899	1.633226
H	7.299263	5.894306	2.839411
H	5.289739	7.022034	3.506072
H	4.912094	6.758858	1.791497
Ag	3.346962	2.42614	4.735045
H	2.18856	1.422166	3.399478

Group(D)-4

C	2.560976	1.548418	1.933112
C	3.751926	0.868973	2.660884
C	2.397387	-0.76456	3.884358
C	2.519499	-1.59916	5.106859
O	1.395144	-0.8448	3.118449
C	3.3455	-1.11122	6.130893
C	3.498004	-1.88314	7.281447
C	2.86846	-3.11476	7.395512
C	2.062444	-3.59278	6.369319
C	1.885105	-2.83072	5.224899
P	3.99737	0.587916	5.914509
S	0.581829	3.324861	2.301738
C	-0.23045	2.251489	1.15028
O	-0.28269	3.532833	3.435346
C	-0.13309	2.484216	-0.20687
C	-0.80048	1.606416	-1.05073
C	-1.51638	0.561025	-0.48552
C	-1.556	0.426148	0.894391
N	-0.91942	1.270192	1.703154
O	1.058724	4.492009	1.589894
C	4.538145	0.031393	1.669866
C	3.999157	-1.1345	1.132507
C	4.729967	-1.8863	0.224318

C	6.003234	-1.48285	-0.15482
C	6.54449	-0.32118	0.377486
C	5.813581	0.433055	1.284417
C	3.087225	2.266169	0.712587
C	3.794299	3.461044	0.818091
C	4.311568	4.074383	-0.31273
C	4.138598	3.494363	-1.56182
C	3.445357	2.297312	-1.67492
C	2.923183	1.686456	-0.54396
C	4.528472	0.898464	7.699967
C	3.624612	1.558321	8.5351
C	3.931851	1.835341	9.858351
C	5.164126	1.461091	10.37648
C	6.078678	0.805706	9.563213
C	5.762471	0.526549	8.241443
C	5.693145	0.430032	5.20679
C	6.141268	-0.73607	4.586819
C	7.398088	-0.78902	4.00451
C	8.224378	0.327122	4.023479
C	7.78919	1.494831	4.635809
C	6.533844	1.543719	5.224175
H	1.828242	0.794724	1.637007
H	4.405171	1.676619	3.027389
H	4.117085	-1.52875	8.097382
H	3.008605	-3.70652	8.293572
H	1.575711	-4.55733	6.463264
H	1.275666	-3.19333	4.404654
H	0.443254	3.32047	-0.57949
H	-0.7576	1.738637	-2.12528
H	-2.04639	-0.14693	-1.11057
H	-2.1113	-0.37178	1.372264
H	3.007995	-1.45877	1.425275
H	4.302915	-2.79322	-0.18966
H	6.573183	-2.07322	-0.86363
H	7.538923	-0.00023	0.087216
H	6.240952	1.341205	1.697295
H	3.936903	3.923866	1.788504
H	4.8548	5.007881	-0.21683
H	4.545214	3.973191	-2.44527

H	3.310696	1.835377	-2.64668
H	2.38899	0.746639	-0.635
H	2.65931	1.860385	8.14029
H	3.210726	2.347961	10.48577
H	5.411893	1.680102	11.4094
H	7.043497	0.508511	9.960524
H	6.489195	0.011182	7.622453
H	5.492893	-1.60412	4.549476
H	7.731473	-1.70258	3.524439
H	9.203834	0.287988	3.55992
H	8.428227	2.371202	4.652732
H	6.201652	2.458364	5.705305
N	1.930247	2.446504	2.9015
N	3.414106	0.064032	3.812972
Ag	-0.20953	0.707765	3.895936
O	-1.10589	0.948031	5.984841
C	-0.65974	1.632722	6.929966
O	0.423433	2.243924	6.958202
C	-1.52964	1.702761	8.182545
H	-2.55897	1.906575	7.895927
H	-1.4887	0.733342	8.676842
H	-1.16063	2.473062	8.854924
C	-3.17864	-0.7951	4.557255
O	-2.16368	-1.0319	3.584008
C	-1.56908	-2.29443	3.857521
C	-1.54592	-2.38918	5.384227
C	-2.80234	-1.61786	5.803021
H	-3.20645	0.277934	4.763178
H	-4.15394	-1.11133	4.157668
H	-2.17796	-3.10156	3.420515
H	-0.57352	-2.30421	3.408509
H	-1.55352	-3.42397	5.729425
H	-0.65302	-1.894	5.773597
H	-3.61379	-2.2974	6.074976
H	-2.59746	-0.96454	6.652027
C	2.106401	5.494999	4.881027
O	2.995138	4.380969	4.929263
C	4.330796	4.838749	4.754796
C	4.239341	6.187668	4.017475

C	2.734656	6.43904	3.856343
H	1.122192	5.128609	4.584002
H	2.040043	5.966996	5.872597
H	4.819738	4.953727	5.733119
H	4.874807	4.083438	4.175795
H	4.708683	6.976419	4.6098
H	4.742018	6.14261	3.049624
H	2.466432	7.478941	4.044967
H	2.407534	6.167232	2.850129
Ag	2.100496	2.108286	5.41345
H	2.572306	3.195202	3.147012

Group(D)-5

C	2.3909	2.008033	0.775443
C	3.436964	1.075388	1.451326
C	4.053784	0.148413	3.502767
C	3.674869	-0.48152	4.832738
O	5.155641	-0.13059	3.031873
C	3.184158	0.113142	6.00642
C	2.962314	-0.70808	7.119511
C	3.217466	-2.07059	7.095377
C	3.720759	-2.65124	5.939621
C	3.946344	-1.85505	4.830092
P	3.030903	1.940414	6.195657
S	0.105603	1.162491	-0.24027
C	-1.25898	0.320157	0.532052
O	-0.42573	2.344893	-0.88227
C	-2.52445	0.523766	0.015679
C	-3.57811	-0.16017	0.604098
C	-3.30064	-1.00516	1.668526
C	-1.9936	-1.13089	2.112954
N	-0.97976	-0.47391	1.551291
O	0.864949	0.236118	-1.03687
C	3.52393	-0.21621	0.661026
C	2.88393	-1.38746	1.044833
C	2.964357	-2.52958	0.262874
C	3.692199	-2.51525	-0.91799
C	4.341219	-1.35183	-1.30879

C	4.257467	-0.21172	-0.52457
C	2.615814	3.453165	1.141265
C	3.556102	4.178822	0.412203
C	3.825489	5.505359	0.71886
C	3.167763	6.127197	1.773623
C	2.230334	5.418178	2.508699
C	1.949546	4.087563	2.19756
C	2.047086	2.234744	7.73513
C	0.89647	1.513852	8.065433
C	0.167857	1.814518	9.206586
C	0.561899	2.854913	10.03642
C	1.690492	3.594136	9.711262
C	2.42217	3.286351	8.574404
C	4.722326	2.198329	6.997752
C	5.325448	3.443948	6.802756
C	6.570765	3.742903	7.335094
C	7.250023	2.789592	8.080725
C	6.669425	1.546217	8.288507
C	5.421035	1.258011	7.756421
H	2.532306	1.900317	-0.3068
H	4.4101	1.582145	1.375263
H	2.602103	-0.27	8.042852
H	3.054182	-2.66583	7.987137
H	3.953248	-3.70989	5.910706
H	4.368106	-2.29142	3.930853
H	-2.67074	1.20095	-0.81566
H	-4.59061	-0.03384	0.239141
H	-4.09192	-1.55972	2.157805
H	-1.74601	-1.7784	2.945025
H	2.316691	-1.41802	1.969438
H	2.45514	-3.43411	0.579076
H	3.75584	-3.40757	-1.53088
H	4.915866	-1.33274	-2.22854
H	4.770763	0.693409	-0.83537
H	4.073248	3.703599	-0.4145
H	4.543655	6.059361	0.124794
H	3.378011	7.163367	2.011945
H	1.691571	5.899008	3.317234
H	1.142519	3.594172	2.724323

H	0.556344	0.710236	7.424368
H	-0.71648	1.233452	9.445192
H	-0.00937	3.091052	10.92715
H	2.00507	4.415222	10.34623
H	3.301142	3.877204	8.341577
H	4.812967	4.192785	6.205511
H	7.012431	4.718569	7.163481
H	8.227084	3.013115	8.494484
H	7.192425	0.793212	8.868249
H	4.993622	0.277689	7.934287
N	1.034605	1.565236	1.104805
N	3.101156	0.869432	2.859257
Ag	0.878904	0.090151	3.158543
O	-0.5149	2.084984	3.727925
C	0.060937	2.309379	4.798443
O	1.015493	1.592265	5.236201
C	-0.36008	3.505555	5.634133
H	-0.74528	3.151939	6.589272
H	-1.12683	4.07427	5.113885
H	0.508396	4.134437	5.825916
C	-0.14186	-1.87764	5.62409
O	0.210633	-2.05603	4.25265
C	0.782727	-3.33953	4.040537
C	0.196747	-4.22516	5.140099
C	-0.00305	-3.24794	6.301368
H	0.52389	-1.13461	6.081006
H	-1.17144	-1.50119	5.663852
H	0.511776	-3.67288	3.033749
H	1.878482	-3.28354	4.120289
H	-0.7617	-4.64254	4.820569
H	0.868136	-5.04463	5.398936
H	-0.88539	-3.49331	6.894311
H	0.868501	-3.25567	6.960414
C	6.739109	2.380143	3.971565
O	5.826658	3.464467	3.782243
C	6.37982	4.29759	2.772607
C	7.020672	3.339235	1.760685
C	7.325508	2.080437	2.585232
H	7.524371	2.687979	4.678021

H	6.188327	1.536794	4.389585
H	5.577022	4.901895	2.343644
H	7.135643	4.964024	3.216244
H	7.917931	3.774807	1.318037
H	6.314931	3.111285	0.957842
H	6.83846	1.205649	2.151834
H	8.397515	1.88654	2.650475
Ag	3.42993	2.930109	3.989742
H	0.498851	2.267745	1.601278

Group(D)-6

C	2.231582	1.711611	1.518498
C	3.585663	1.078858	1.934503
C	2.832789	-0.18599	3.800348
C	3.192151	-0.88161	5.091702
O	1.809835	-0.62442	3.197428
C	3.349843	-0.31518	6.363878
C	3.62486	-1.16166	7.4395
C	3.759707	-2.53097	7.272945
C	3.617067	-3.08772	6.010381
C	3.326699	-2.26395	4.936452
P	3.168289	1.503055	6.605229
S	0.623393	3.575623	2.610925
C	-0.6179	2.817833	1.591346
O	0.406201	3.103192	3.958851
C	-1.02743	3.457646	0.437038
C	-2.00135	2.833096	-0.32868
C	-2.49846	1.611117	0.103158
C	-2.02001	1.057243	1.281221
N	-1.08697	1.657252	2.017891
O	0.626308	4.995291	2.361598
C	3.983185	-0.13363	1.119101
C	3.075953	-1.08049	0.65304
C	3.516357	-2.19191	-0.05138
C	4.869348	-2.37712	-0.29639
C	5.783092	-1.44237	0.170861
C	5.341356	-0.33116	0.872988
C	2.117548	1.867265	0.022154

C	2.99728	2.694227	-0.67505
C	2.893567	2.830659	-2.05134
C	1.91212	2.140122	-2.74923
C	1.035662	1.310718	-2.06375
C	1.13776	1.175784	-0.68683
C	2.70266	1.881907	8.352485
C	3.495168	1.547826	9.452663
C	3.127636	1.928558	10.73537
C	1.975686	2.674417	10.94125
C	1.199172	3.048438	9.852978
C	1.562191	2.657516	8.572843
C	5.009972	1.858119	6.792501
C	5.955162	1.137335	6.055994
C	7.301671	1.471553	6.081189
C	7.739345	2.552703	6.836246
C	6.813314	3.30159	7.551801
C	5.46849	2.961196	7.520912
H	1.405015	1.110997	1.898581
H	4.350464	1.851135	1.768798
H	3.734347	-0.75016	8.434961
H	3.973273	-3.15966	8.130404
H	3.72146	-4.15726	5.865623
H	3.205323	-2.68936	3.945995
H	-0.59998	4.412513	0.160629
H	-2.36009	3.291373	-1.24259
H	-3.25654	1.089633	-0.46776
H	-2.38549	0.106744	1.650303
H	2.016987	-0.963	0.840621
H	2.795394	-2.91955	-0.40853
H	5.211299	-3.24653	-0.84695
H	6.843509	-1.57865	-0.01233
H	6.062629	0.39359	1.237807
H	3.774984	3.232413	-0.14248
H	3.584465	3.476753	-2.58141
H	1.832285	2.246772	-3.825
H	0.267069	0.766856	-2.60179
H	0.447381	0.527739	-0.15852
H	4.420912	1.001564	9.314582
H	3.751456	1.647899	11.57715

H	1.691587	2.97365	11.94408
H	0.308677	3.649441	10.00099
H	0.951249	2.959643	7.730076
H	5.634009	0.294277	5.453718
H	8.013813	0.883373	5.512172
H	8.792974	2.807148	6.870625
H	7.140931	4.153488	8.138429
H	4.762259	3.559715	8.087567
N	2.160686	3.027305	2.166242
N	3.659459	0.744161	3.35857
Ag	-0.13762	0.453959	3.906679
O	-0.86382	-0.18358	6.081323
C	0.099268	0.187369	6.765169
O	1.037686	0.906907	6.306154
C	0.153621	-0.24868	8.2189
H	1.148077	-0.6232	8.453709
H	-0.0543	0.610551	8.854315
H	-0.59095	-1.02073	8.398291
C	-2.2656	-2.11233	4.081012
O	-1.5392	-1.55424	2.988564
C	-0.59539	-2.5297	2.55958
C	-0.11453	-3.21437	3.84248
C	-1.29462	-3.05639	4.810132
H	-2.60895	-1.2922	4.714865
H	-3.13738	-2.66574	3.699023
H	-1.08377	-3.24981	1.884065
H	0.207021	-2.01769	2.025293
H	0.142671	-4.26014	3.665925
H	0.766588	-2.69748	4.22799
H	-1.77455	-4.01415	5.0236
H	-0.96845	-2.61573	5.753519
C	5.190804	5.329318	4.788527
O	4.830428	4.226141	3.965094
C	5.985197	3.608268	3.396917
C	7.195481	4.436437	3.842546
C	6.581284	5.753868	4.317932
H	4.438884	6.114656	4.659126
H	5.210819	5.017397	5.844016
H	6.051193	2.570637	3.7468

H	5.873952	3.608921	2.303838
H	7.710616	3.938124	4.667971
H	7.905038	4.58143	3.026873
H	6.50368	6.462868	3.489583
H	7.156218	6.213583	5.122553
Ag	2.967352	2.685686	4.504024
H	2.565923	3.762832	1.598199

Group(D)-7

C	2.20659	1.645232	0.904858
C	3.53938	0.951568	1.301006
C	3.087241	-0.26998	3.301244
C	3.558258	-0.79123	4.641483
O	2.177647	-0.95618	2.757029
C	3.675563	-0.13762	5.874338
C	4.070161	-0.88945	6.990278
C	4.342666	-2.24263	6.909707
C	4.243403	-2.88496	5.682962
C	3.845853	-2.16202	4.573495
P	3.537957	1.683971	6.116224
S	-0.0771	0.868283	-0.22879
C	-1.60281	0.943229	0.689327
O	0.092761	2.143015	-0.8875
C	-2.56503	1.85776	0.310343
C	-3.76266	1.857372	1.01197
C	-3.9273	0.9364	2.036437
C	-2.89553	0.062061	2.337707
N	-1.73816	0.069115	1.673164
O	-0.09955	-0.33751	-1.0219
C	3.860764	-0.28459	0.488327
C	3.412657	-0.4475	-0.93258
C	3.44358	-1.83918	-1.41062
C	3.852104	-2.87606	-0.64516
C	4.756216	-2.53228	0.378425
C	4.658663	-1.27228	0.990252
C	1.91994	2.890873	1.71367
C	2.900135	3.890462	1.810693
C	2.646565	5.06359	2.519583

C	1.412716	5.263804	3.124549
C	0.432825	4.290129	3.009376
C	0.687518	3.116213	2.314397
C	2.638708	1.924082	7.70567
C	1.819663	0.952078	8.278039
C	1.137829	1.206136	9.459002
C	1.255694	2.440732	10.08325
C	2.049902	3.42583	9.511871
C	2.73078	3.170323	8.330389
C	5.302273	1.776535	6.792791
C	6.314277	1.464607	5.87924
C	7.653685	1.514332	6.225158
C	8.022	1.884255	7.51269
C	7.036373	2.189855	8.438872
C	5.694719	2.13248	8.083161
H	2.332046	1.968855	-0.13894
H	4.309961	1.689363	1.022443
H	4.180461	-0.39244	7.948205
H	4.642467	-2.78963	7.796582
H	4.468373	-3.94179	5.593794
H	3.760993	-2.65973	3.613376
H	-2.37337	2.545482	-0.50285
H	-4.54834	2.559863	0.760117
H	-4.84802	0.899056	2.605361
H	-2.98482	-0.6684	3.132812
H	2.398406	-0.06914	-1.09277
H	4.041927	0.140656	-1.61159
H	3.728564	-3.90677	-0.95394
H	5.535341	-3.2208	0.691092
H	5.180826	-1.12475	1.929773
H	3.834444	3.79037	1.270744
H	3.410401	5.831803	2.570631
H	1.216379	6.179545	3.670205
H	-0.5413	4.438795	3.460962
H	-0.09595	2.374656	2.238635
H	1.703005	-0.00895	7.791647
H	0.507818	0.436654	9.891905
H	0.722735	2.638115	11.00661
H	2.136091	4.397939	9.984965

H	3.332428	3.953673	7.880399
H	6.037443	1.16422	4.873073
H	8.412847	1.263698	5.491455
H	9.069051	1.928881	7.791169
H	7.31024	2.472065	9.450009
H	4.950087	2.365201	8.83603
N	1.095737	0.693884	0.972796
N	3.738399	0.729648	2.735581
Ag	0.051317	-0.17265	3.213348
O	-0.64253	1.568879	4.650495
C	0.324223	1.944077	5.333779
O	1.460373	1.393998	5.306423
C	0.097536	3.140496	6.246784
H	0.953843	3.810487	6.211822
H	-0.02907	2.781203	7.266699
H	-0.80465	3.665266	5.941292
C	-1.1376	-1.64982	5.741788
O	-1.33248	-1.97128	4.368531
C	-0.67561	-3.21274	4.140521
C	0.592945	-3.17068	5.005964
C	0.303108	-2.07868	6.047259
H	-1.30428	-0.5783	5.866006
H	-1.85631	-2.20912	6.359577
H	-1.33362	-4.04074	4.444546
H	-0.46224	-3.29379	3.072354
H	0.787318	-4.13968	5.469121
H	1.45661	-2.89698	4.397301
H	0.981967	-1.23426	5.909
H	0.404076	-2.44298	7.070857
C	6.560547	4.734221	2.888137
O	6.140315	3.431082	3.264065
C	6.881443	2.488052	2.499409
C	7.06981	3.138818	1.124045
C	6.962278	4.644855	1.405262
H	5.730141	5.424834	3.060271
H	7.416161	5.041851	3.507824
H	7.8523	2.298511	2.98106
H	6.308399	1.558836	2.460726
H	8.032158	2.869252	0.686983

H	6.278917	2.819523	0.441345
H	7.91106	5.15591	1.231875
H	6.204188	5.105896	0.769026
Ag	3.882716	2.65402	3.916674
H	1.441498	-0.25884	0.904877

3.1.3 Result (B3LYP/def2-SV(P)/CPCM(THF) after removing the THF molecules)

In order to compare the free energies of these four types of conformational states, the systems were balanced so that the number of atoms matched between the conformations, divided in each case. Upon deprotonation of the amide proton of the ligand, the acetate anion of silver acetate abstracts the proton, generating the salt states of KOAc and $KHCO_3$. The most stable conformation was the **(A)** structure, in which one amide proton on the sulfonamide side was abstracted, and the resulting structure featured one silver atom coordinated to the phosphine and sulfonyl oxygen atoms, while the other silver atom was coordinated to the sulfonamide and picolinamide moieties. Furthermore, the acetate anion was predicted to bridge the two silver centers (**Figure S2**).

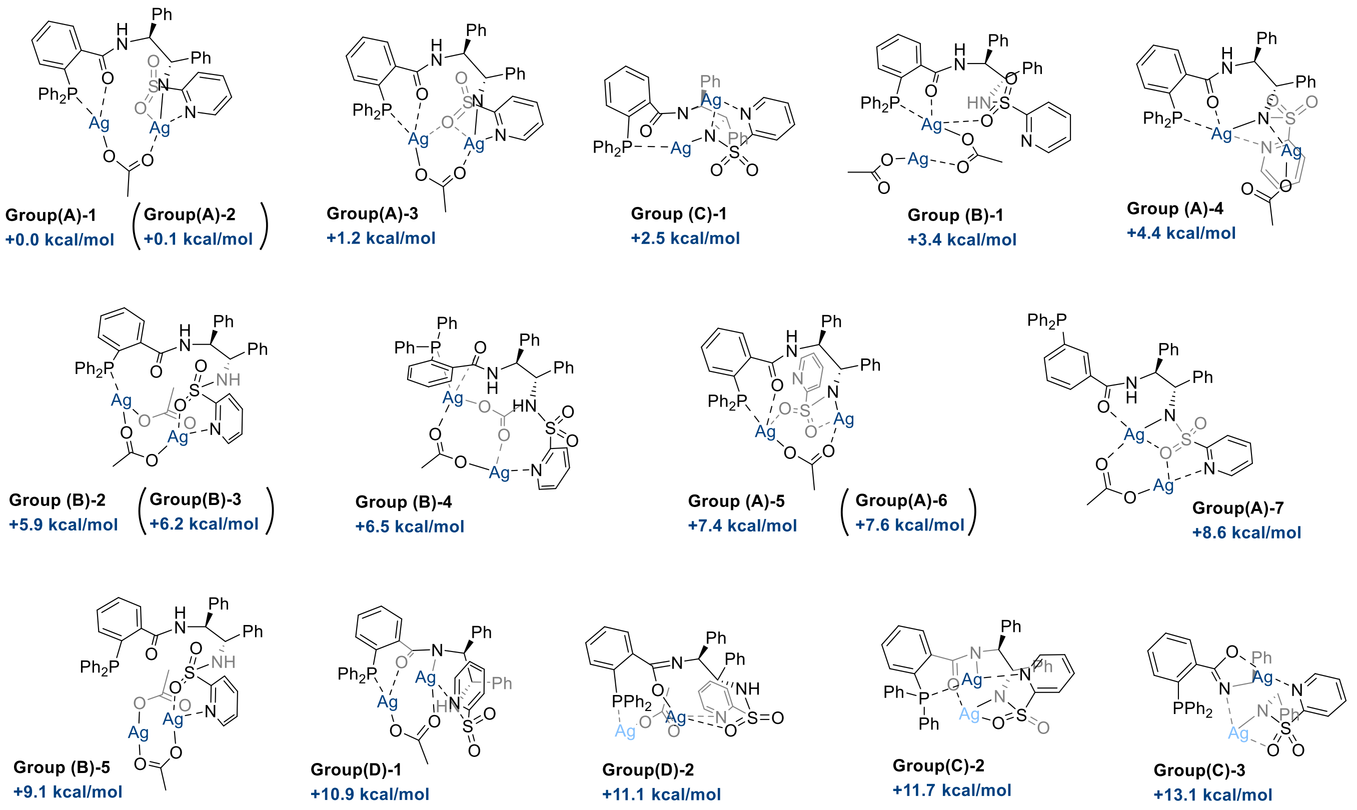

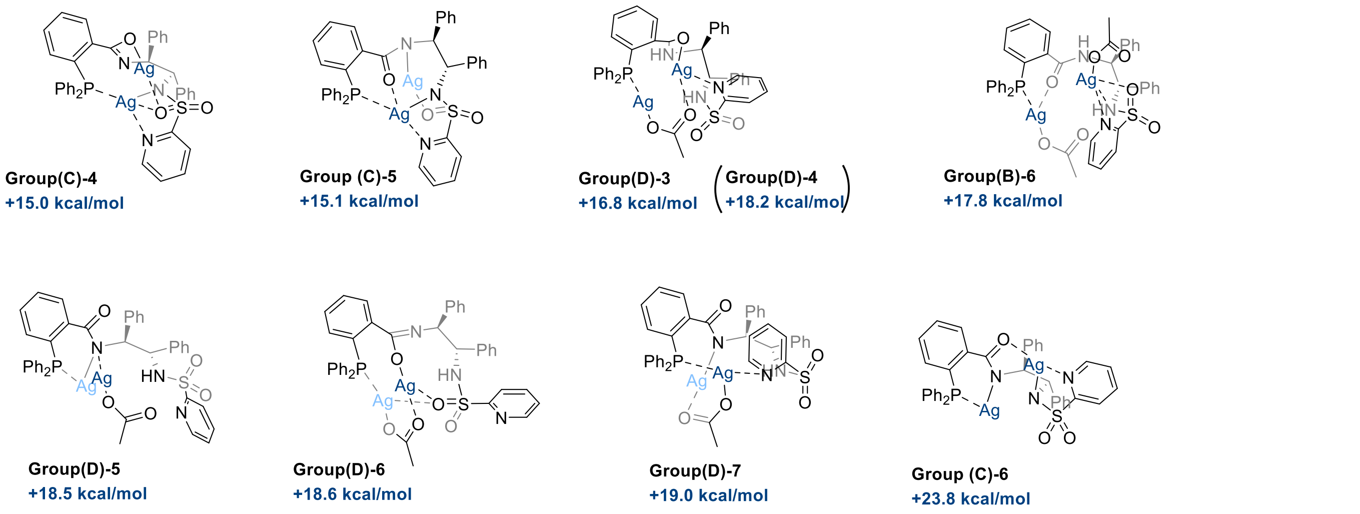

Figure S2. Result of SC-AFIR search divided into four cases by the B3LYP/def2-SV(P)/CPCM(THF) after removing the THF molecules

Calculated Structures

(A) Deprotonation of sulfonamide proton

Group(A)-1

B3LYP/def2-SV(P)/CPCM(THF)

B3LYP/def2-SV(P)/CPCM(THF) free energy: -3116.40744099167 (a.u.)

C	2.400938	0.113002	-0.30571
C	2.052586	1.564002	-0.76914
C	-0.45271	1.386952	-0.81313
C	-1.67526	1.468785	-1.68903
O	-0.56748	1.109635	0.384382
C	-2.89159	0.866895	-1.27213
C	-4.00623	0.950459	-2.12461
C	-3.94496	1.629327	-3.34513
C	-2.75745	2.247611	-3.73717
C	-1.63664	2.163948	-2.90995
P	-3.06054	-0.04657	0.338851
S	2.251015	-1.00754	-2.78752
C	3.855229	-1.84879	-3.06601
O	1.264687	-1.91114	-3.4231
C	4.769495	-1.31014	-3.97531
C	5.948885	-2.02513	-4.20479
C	6.155028	-3.22633	-3.52171

C	5.165614	-3.66843	-2.63535
N	4.034226	-2.99282	-2.41531
O	2.420529	0.33922	-3.40649
C	2.248504	2.636995	0.303412
C	2.778637	3.877674	-0.09203
C	2.969115	4.913381	0.827976
C	2.63635	4.721975	2.173225
C	2.112908	3.490796	2.582527
C	1.918627	2.4594	1.656794
C	3.874978	-0.03256	0.075838
C	4.920582	0.429902	-0.74188
C	6.257411	0.243764	-0.37249
C	6.576062	-0.40893	0.823558
C	5.54576	-0.87381	1.648125
C	4.211057	-0.68358	1.275001
C	-3.23645	1.235407	1.648704
C	-3.52591	2.577389	1.356061
C	-3.67676	3.508371	2.389837
C	-3.54467	3.10818	3.723891
C	-3.25854	1.771044	4.02207
C	-3.10185	0.840286	2.990234
C	-4.76505	-0.77372	0.240009
C	-4.91167	-2.04834	-0.33824
C	-6.16959	-2.65106	-0.43007
C	-7.29978	-1.99318	0.069228
C	-7.16469	-0.72976	0.654155
C	-5.90685	-0.12161	0.737283
H	1.821874	-0.07372	0.606386
H	2.732525	1.820309	-1.58798
H	-4.94902	0.48457	-1.83462
H	-4.83423	1.680324	-3.98006
H	-2.69995	2.798836	-4.67996
H	-0.72039	2.674639	-3.21453
H	4.556708	-0.36517	-4.47694
H	6.696767	-1.64599	-4.90781
H	7.064131	-3.81523	-3.66963
H	5.291571	-4.60668	-2.08234
H	3.048783	4.035168	-1.14171
H	3.384209	5.869282	0.493133

H	2.786547	5.526999	2.899508
H	1.849597	3.329531	3.632755
H	1.493561	1.514417	1.997892
H	4.699352	0.937466	-1.68454
H	7.05501	0.61299	-1.02499
H	7.622029	-0.55207	1.11233
H	5.780624	-1.38304	2.588207
H	3.411074	-1.0466	1.929336
H	-3.63154	2.905791	0.31859
H	-3.89848	4.552501	2.148628
H	-3.66139	3.838276	4.530586
H	-3.14961	1.449965	5.062428
H	-2.86785	-0.20122	3.235777
H	-4.03357	-2.58029	-0.72068
H	-6.26466	-3.64176	-0.88471
H	-8.28388	-2.46765	0.006842
H	-8.04306	-0.21088	1.050102
H	-5.82034	0.867184	1.194798
N	1.907829	-0.9388	-1.21924
N	0.734478	1.642942	-1.41057
H	0.785663	1.554343	-2.42167
Ag	1.108313	-2.76219	-0.46879
Ag	-1.57545	-1.88534	0.900436
O	-1.02357	-3.76763	1.841576
C	-0.30474	-4.69775	1.382606
O	0.488749	-4.60614	0.40382
C	-0.36864	-6.04027	2.09559
H	-1.38081	-6.22604	2.48825
H	-0.06031	-6.8576	1.425724
H	0.327962	-6.01676	2.954096

Group(A)-2

B3LYP/def2-SV(P)/CPCM(THF)

B3LYP/def2-SV(P)/CPCM(THF) free energy: -3116.40730416245 (a.u.)

C	2.156537	0.919968	2.430639
C	3.538477	0.643005	1.742959
C	4.871811	0.049925	3.812518

C	6.129105	0.413976	4.565639
O	4.101654	-0.81236	4.239243
C	6.114644	0.59606	5.971056
C	7.320582	0.916145	6.616966
C	8.520011	1.029062	5.905892
C	8.530355	0.837388	4.522969
C	7.336752	0.539566	3.860437
P	4.525051	0.51329	6.930208
S	2.733711	3.438515	3.246944
C	1.377367	4.49754	2.606147
O	3.009679	4.03686	4.577535
C	1.660564	5.499919	1.678675
C	0.617432	6.341913	1.284148
C	-0.65427	6.146706	1.828873
C	-0.83707	5.115842	2.752585
N	0.165225	4.307794	3.133173
O	3.804365	3.563495	2.221279
C	3.542603	-0.64285	0.916121
C	3.197436	-1.89667	1.451251
C	3.220685	-3.04458	0.651766
C	3.586806	-2.96322	-0.69661
C	3.931741	-1.72206	-1.24115
C	3.907815	-0.57591	-0.43927
C	1.04871	1.127962	1.395589
C	1.166588	2.035325	0.328219
C	0.112607	2.225929	-0.57223
C	-1.08117	1.510492	-0.42519
C	-1.21176	0.601804	0.630516
C	-0.15494	0.415071	1.528606
C	4.415085	-1.22417	7.535972
C	5.522866	-2.08444	7.61505
C	5.371021	-3.39034	8.094762
C	4.113536	-3.84822	8.503039
C	3.005158	-2.99711	8.423457
C	3.150327	-1.69368	7.936644
C	4.886403	1.503805	8.447943
C	4.703602	2.897727	8.36987
C	4.938439	3.709326	9.483185
C	5.342083	3.13753	10.69608

C	5.512203	1.752289	10.7874
C	5.287778	0.938757	9.670212
H	1.892458	0.012177	2.987863
H	3.723496	1.464383	1.043616
H	7.328831	1.084012	7.696585
H	9.445097	1.268099	6.438847
H	9.463225	0.919415	3.957617
H	7.34301	0.383314	2.778179
H	2.671635	5.604251	1.282883
H	0.796987	7.140186	0.558158
H	-1.49844	6.780373	1.547063
H	-1.81528	4.92793	3.203795
H	2.930859	-1.97841	2.506215
H	2.950952	-4.01162	1.088281
H	3.602584	-3.86307	-1.3196
H	4.218725	-1.64272	-2.29448
H	4.175986	0.391346	-0.87717
H	2.086142	2.611359	0.194459
H	0.227426	2.937764	-1.3958
H	-1.90361	1.65826	-1.13198
H	-2.13772	0.03149	0.754677
H	-0.26479	-0.30242	2.348603
H	6.511596	-1.74139	7.298491
H	6.241198	-4.05195	8.148431
H	3.996354	-4.87006	8.877126
H	2.017103	-3.35214	8.732611
H	2.27161	-1.04394	7.854008
H	4.369646	3.352395	7.430825
H	4.79519	4.791435	9.405719
H	5.517275	3.771458	11.57076
H	5.822061	1.298024	11.73366
H	5.425884	-0.14222	9.756634
N	2.180727	1.955649	3.486726
N	4.682536	0.739313	2.661936
H	5.252905	1.568384	2.528279
Ag	-0.14604	2.557276	4.552371
Ag	2.488942	1.256076	5.692022
O	0.640015	0.499116	6.953093
C	-0.60367	0.619128	6.838072

O	-1.21013	1.350094	5.995873
C	-1.48114	-0.21099	7.771653
H	-2.5129	0.171956	7.801924
H	-1.50402	-1.25417	7.405663
H	-1.05212	-0.22692	8.787133

Group(A)⁻3

B3LYP/def2-SV(P)/CPCM(THF)

B3LYP/def2-SV(P)/CPCM(THF) free energy: -3116.40557250981 (a.u.)

C	2.006683	-0.61316	2.345334
C	3.10331	-1.15574	1.36902
C	5.078637	-1.12338	2.970963
C	6.557597	-0.8148	3.015272
O	4.505763	-1.60435	3.948814
C	7.165301	-0.26801	4.17324
C	8.554828	-0.06128	4.167306
C	9.338675	-0.40322	3.060036
C	8.738537	-0.95241	1.92655
C	7.35554	-1.15114	1.90962
P	6.151648	0.273818	5.631356
S	2.785246	1.996806	2.149258
C	1.347176	3.073558	1.782046
O	3.583742	2.822376	3.113205
C	1.272752	3.737528	0.556367
C	0.176594	4.581066	0.348416
C	-0.77706	4.716676	1.360619
C	-0.59676	4.001854	2.550312
N	0.450321	3.196631	2.753771
O	3.420437	1.733914	0.8336
C	2.896969	-2.63527	1.044877
C	2.950354	-3.63582	2.032428
C	2.762499	-4.98029	1.695261
C	2.513922	-5.35057	0.368292
C	2.458234	-4.36466	-0.6219
C	2.649685	-3.02051	-0.28286
C	0.617883	-0.7018	1.710267
C	0.306235	-0.04449	0.506914

C	-0.98051	-0.11456	-0.03615
C	-1.98224	-0.84592	0.613768
C	-1.68568	-1.50489	1.811108
C	-0.39595	-1.43148	2.350997
C	5.955998	-1.2122	6.69982
C	6.757283	-2.35766	6.571636
C	6.570624	-3.44837	7.428105
C	5.586426	-3.40385	8.421802
C	4.783384	-2.26508	8.553974
C	4.96304	-1.17717	7.694024
C	7.315705	1.348906	6.588287
C	7.38911	2.711658	6.243406
C	8.240344	3.577345	6.935768
C	9.019932	3.096373	7.99459
C	8.94599	1.746384	8.352615
C	8.101275	0.876159	7.653382
H	2.00568	-1.28483	3.213778
H	3.00317	-0.60642	0.427035
H	9.042064	0.375845	5.041277
H	10.4198	-0.23887	3.091198
H	9.340903	-1.23061	1.056934
H	6.888126	-1.59832	1.028354
H	2.04448	3.591337	-0.20083
H	0.070177	5.123971	-0.59544
H	-1.64912	5.363671	1.235751
H	-1.32114	4.080065	3.368666
H	3.157832	-3.35998	3.06857
H	2.810786	-5.74504	2.477098
H	2.364991	-6.40324	0.107929
H	2.265294	-4.64021	-1.66353
H	2.603241	-2.25631	-1.06568
H	1.072931	0.536882	-0.01331
H	-1.20173	0.405421	-0.97364
H	-2.98872	-0.90168	0.187476
H	-2.45945	-2.08122	2.328119
H	-0.17179	-1.95349	3.28734
H	7.528741	-2.4065	5.798315
H	7.198617	-4.33768	7.316472
H	5.441568	-4.25846	9.089804

H	4.006969	-2.22515	9.324029
H	4.319598	-0.29628	7.792434
H	6.772512	3.103883	5.427307
H	8.28656	4.633453	6.653287
H	9.679715	3.775289	8.54349
H	9.548807	1.36419	9.182166
H	8.05662	-0.17642	7.944591
N	2.294321	0.700048	2.947101
N	4.481904	-0.85944	1.783454
H	4.999629	-0.28045	1.131728
Ag	1.184395	1.336346	4.728853
Ag	4.073418	1.539814	5.219152
O	2.913016	2.551455	6.963356
C	1.726035	2.489161	7.371347
O	0.746852	1.983885	6.738806
C	1.418213	3.100652	8.733876
H	1.435902	4.202265	8.643357
H	0.427073	2.792235	9.100029
H	2.196392	2.819113	9.462677

Group(A)-4

B3LYP/def2-SV(P)/CPCM(THF)

B3LYP/def2-SV(P)/CPCM(THF) free energy: -3116.40040562872 (a.u.)

C	1.533834	0.120523	1.723274
C	2.809998	-0.31626	0.937392
C	4.385482	-0.49998	2.898918
C	5.785151	-0.16707	3.357353
O	3.642499	-1.17371	3.61477
C	6.038637	0.247258	4.688168
C	7.369386	0.448202	5.087919
C	8.432617	0.235311	4.202721
C	8.177535	-0.16636	2.890667
C	6.857343	-0.36175	2.473813
P	4.634057	0.669377	5.823988
S	2.053144	2.744519	1.312397
C	2.02297	4.175448	2.455708
O	3.473226	2.627702	0.873373

C	1.574741	5.387078	1.927046
C	1.676394	6.528396	2.727776
C	2.217057	6.409118	4.009134
C	2.630888	5.147991	4.4454
N	2.537965	4.051006	3.679596
O	1.120625	3.11151	0.217214
C	2.758175	-1.75894	0.430564
C	2.453761	-2.85179	1.260883
C	2.44398	-4.15411	0.749839
C	2.737395	-4.39015	-0.59808
C	3.042012	-3.31112	-1.43405
C	3.050252	-2.0098	-0.92063
C	0.249361	-0.19362	0.950266
C	0.029661	0.228158	-0.37188
C	-1.16791	-0.07092	-1.02914
C	-2.1721	-0.79737	-0.37773
C	-1.96738	-1.22367	0.938223
C	-0.76523	-0.92489	1.590319
C	4.169756	-0.87643	6.704005
C	5.016124	-1.99362	6.782215
C	4.611444	-3.1359	7.481397
C	3.361976	-3.17031	8.110933
C	2.514001	-2.05932	8.035383
C	2.911835	-0.91834	7.330758
C	5.43702	1.701435	7.132743
C	5.652434	3.065331	6.858715
C	6.244565	3.89916	7.810699
C	6.615508	3.385192	9.059531
C	6.395831	2.034338	9.345669
C	5.812655	1.195218	8.388153
H	1.512135	-0.49242	2.632773
H	2.891509	0.321844	0.0484
H	7.587041	0.783256	6.104561
H	9.460288	0.389806	4.544689
H	9.001542	-0.33264	2.190684
H	6.653269	-0.69221	1.451809
H	1.158924	5.420023	0.91955
H	1.334641	7.497001	2.351916
H	2.317086	7.274008	4.669124

H	3.049063	5.009461	5.446825
H	2.239965	-2.68663	2.317896
H	2.204604	-4.99181	1.412689
H	2.727265	-5.41025	-0.99476
H	3.271292	-3.47992	-2.49094
H	3.285674	-1.17149	-1.5849
H	0.785678	0.81894	-0.89307
H	-1.3184	0.268904	-2.05886
H	-3.10862	-1.02956	-0.89451
H	-2.74191	-1.79489	1.459724
H	-0.61066	-1.27066	2.618407
H	5.993518	-1.97963	6.291859
H	5.276596	-4.00343	7.532854
H	3.047052	-4.06535	8.656272
H	1.533149	-2.08236	8.520079
H	2.236659	-0.05823	7.261973
H	5.355167	3.480817	5.889857
H	6.408581	4.956093	7.579702
H	7.070831	4.039191	9.809482
H	6.67985	1.625871	10.32038
H	5.650744	0.141116	8.626874
N	1.576042	1.512324	2.254547
N	4.059489	-0.04848	1.665233
H	4.603512	0.725173	1.293441
Ag	0.025709	1.886258	3.718282
Ag	2.803112	1.938175	4.763163
O	-0.97432	2.180612	5.584597
C	-0.18439	2.295057	6.582602
O	1.065064	2.250482	6.532243
C	-0.86776	2.479443	7.933982
H	-1.63186	3.272855	7.870342
H	-1.39127	1.545118	8.206698
H	-0.13705	2.724041	8.719792

Group(A)⁻5

B3LYP/def2-SV(P)/CPCM(THF)

B3LYP/def2-SV(P)/CPCM(THF) free energy: -3116.39572646885 (a.u.)

C	1.629744	-0.03372	2.602345
C	2.534668	-0.50578	1.415613
C	4.664836	-0.68368	2.766551
C	6.122007	-0.30324	2.777416
O	4.196164	-1.33147	3.708158
C	6.795269	-0.06191	4.002572
C	8.163796	0.253728	3.962111
C	8.86626	0.305521	2.753302
C	8.203733	0.049531	1.552401
C	6.83925	-0.24911	1.57178
P	5.896895	-0.05626	5.629956
S	2.708823	2.545994	2.755062
C	3.048042	3.002892	1.007118
O	2.132856	3.791725	3.321189
C	2.432005	4.15041	0.49675
C	2.715096	4.50514	-0.82499
C	3.593712	3.713078	-1.56889
C	4.152451	2.586774	-0.95813
N	3.882654	2.242301	0.305956
O	4.032165	2.1543	3.334038
C	2.25648	-1.92932	0.930345
C	2.478194	-3.06265	1.733784
C	2.225785	-4.34674	1.240343
C	1.745859	-4.52601	-0.0625
C	1.522759	-3.40738	-0.87101
C	1.777848	-2.12347	-0.3757
C	0.188088	-0.50627	2.428031
C	-0.64554	0.061547	1.450184
C	-1.96365	-0.37518	1.292452
C	-2.47578	-1.38369	2.11974
C	-1.6586	-1.94852	3.103517
C	-0.33627	-1.51109	3.25393
C	5.842506	-1.80553	6.201797
C	6.645617	-2.81525	5.64862
C	6.57059	-4.12418	6.137729
C	5.697276	-4.43655	7.185503
C	4.893943	-3.43436	7.741552
C	4.962604	-2.12733	7.248938
C	7.115855	0.725918	6.787437

C	7.092065	2.12675	6.920913
C	7.976642	2.777361	7.785866
C	8.889831	2.034272	8.543279
C	8.915129	0.640559	8.426782
C	8.036136	-0.01047	7.55333
H	2.034759	-0.52408	3.499118
H	2.314785	0.155573	0.565162
H	8.701384	0.461993	4.889066
H	9.934361	0.542235	2.758704
H	8.744122	0.076286	0.601687
H	6.324514	-0.46934	0.633306
H	1.767084	4.743587	1.124713
H	2.256683	5.395597	-1.26517
H	3.844739	3.959469	-2.60371
H	4.844049	1.936097	-1.50498
H	2.862033	-2.93478	2.747431
H	2.405803	-5.2158	1.881384
H	1.548531	-5.5324	-0.44504
H	1.150182	-3.53107	-1.89284
H	1.599928	-1.2557	-1.01951
H	-0.25951	0.865151	0.816306
H	-2.59813	0.077147	0.523447
H	-3.50966	-1.72253	1.999983
H	-2.04983	-2.73147	3.760849
H	0.297292	-1.95947	4.025975
H	7.330564	-2.58587	4.827761
H	7.198855	-4.90366	5.695603
H	5.639392	-5.46113	7.565551
H	4.204021	-3.67101	8.557317
H	4.319832	-1.35231	7.680454
H	6.370132	2.717421	6.346104
H	7.944792	3.867422	7.875594
H	9.576538	2.541157	9.228155
H	9.622873	0.052583	9.01929
H	8.070385	-1.1001	7.473122
N	1.544207	1.43499	2.829471
N	3.966898	-0.29031	1.674918
H	4.354099	0.486476	1.129339
Ag	0.727426	1.760727	4.846901

Ag	3.787713	1.234797	5.802939
O	2.506861	2.118377	7.411781
C	1.287637	2.206653	7.71299
O	0.309024	2.026532	6.928509
C	0.959471	2.55893	9.158608
H	1.491017	3.482958	9.44464
H	-0.12269	2.691151	9.306825
H	1.324749	1.754889	9.822113

Group(A)-6

B3LYP/def2-SV(P)/CPCM(THF)

B3LYP/def2-SV(P)/CPCM(THF) free energy: -3116.39538444192 (a.u.)

C	1.614424	0.033726	2.762443
C	2.442122	-0.41781	1.512274
C	4.666364	-0.54563	2.70862
C	6.104525	-0.10637	2.627281
O	4.287457	-1.2206	3.671419
C	6.843555	0.161609	3.807582
C	8.191771	0.536622	3.681759
C	8.812488	0.621497	2.430873
C	8.085981	0.339404	1.273483
C	6.739698	-0.01818	1.3784
P	6.056418	0.110849	5.490707
S	2.637452	2.634736	2.859034
C	2.870598	3.117429	1.100165
O	2.070372	3.864029	3.468896
C	2.218042	4.266469	0.641048
C	2.419993	4.639186	-0.69062
C	3.257935	3.8626	-1.49528
C	3.859185	2.733171	-0.93274
N	3.667088	2.371829	0.340747
O	3.995271	2.258848	3.360879
C	2.163384	-1.84529	1.039506
C	2.472137	-2.97742	1.815384
C	2.2152	-4.26454	1.332297
C	1.643838	-4.44832	0.06753
C	1.33323	-3.33093	-0.71336

C	1.593046	-2.04397	-0.22857
C	0.180187	-0.48696	2.701525
C	-0.74571	0.049927	1.791421
C	-2.05684	-0.43076	1.737074
C	-2.46898	-1.45361	2.601649
C	-1.55894	-1.98884	3.518195
C	-0.24432	-1.50716	3.565231
C	6.151915	-1.64214	6.046432
C	6.986493	-2.59059	5.434288
C	7.028777	-3.90587	5.91021
C	6.242563	-4.28576	7.003582
C	5.408819	-3.34497	7.618962
C	5.360097	-2.03209	7.139763
C	7.301909	0.96665	6.565105
C	7.232745	2.369342	6.663138
C	8.132841	3.074224	7.467071
C	9.107405	2.385357	8.199231
C	9.178181	0.990976	8.117982
C	8.283689	0.285112	7.304475
H	2.101681	-0.43506	3.629707
H	2.14639	0.241458	0.683324
H	8.77787	0.765589	4.573838
H	9.867522	0.904318	2.369034
H	8.562802	0.391782	0.29037
H	6.175515	-0.25769	0.473644
H	1.587338	4.84694	1.314448
H	1.930519	5.531558	-1.09179
H	3.445353	4.123199	-2.54005
H	4.521314	2.093943	-1.52747
H	2.927356	-2.84545	2.798454
H	2.463986	-5.13251	1.951462
H	1.443527	-5.45712	-0.30702
H	0.888371	-3.45793	-1.70544
H	1.346206	-1.17724	-0.85068
H	-0.43705	0.864072	1.129339
H	-2.76407	-0.0017	1.019959
H	-3.49727	-1.82672	2.563133
H	-1.87125	-2.78303	4.203658
H	0.461937	-1.93249	4.285513

H	7.605219	-2.30832	4.578191
H	7.680486	-4.63705	5.421954
H	6.276184	-5.31522	7.373229
H	4.786493	-3.63474	8.471123
H	4.69419	-1.30631	7.618849
H	6.465248	2.91977	6.107614
H	8.065849	4.164633	7.529176
H	9.806686	2.93528	8.836514
H	9.934258	0.444926	8.69058
H	8.354954	-0.80424	7.250407
N	1.497562	1.501673	2.980865
N	3.883284	-0.1672	1.67097
H	4.20857	0.626798	1.109086
Ag	0.764086	1.812292	5.034487
Ag	3.8862	1.253103	5.844895
O	2.642948	1.970578	7.555906
C	1.439178	2.106351	7.898642
O	0.430384	2.028782	7.136288
C	1.172463	2.386467	9.372516
H	0.101704	2.549545	9.565907
H	1.529206	1.533684	9.977035
H	1.747849	3.273194	9.690351

Group(A)-7

B3LYP/def2-SV(P)/CPCM(THF)

B3LYP/def2-SV(P)/CPCM(THF) free energy: -3116.39373430375 (a.u.)

C	1.962348	0.20715	1.695508
C	3.417612	-0.18345	1.273686
C	4.25254	-0.43063	3.659851
C	5.366329	0.010806	4.576677
O	3.293792	-1.08024	4.089359
C	5.940255	-0.88687	5.511187
C	6.950772	-0.40074	6.358112
C	7.367304	0.934331	6.30945
C	6.781817	1.816093	5.398377
C	5.788682	1.350297	4.53256
P	5.433226	-2.68514	5.504206

S	2.383192	2.826377	2.205185
C	0.954278	3.801223	1.575857
O	2.762828	3.561834	3.440665
C	0.915134	4.167508	0.230902
C	-0.17632	4.911756	-0.2254
C	-1.18199	5.260076	0.677249
C	-1.05311	4.860813	2.008267
N	-0.00149	4.149776	2.450458
O	3.351283	2.843134	1.08334
C	3.486319	-1.56107	0.616375
C	3.10625	-2.73132	1.298138
C	3.185795	-3.9788	0.670939
C	3.645302	-4.08106	-0.64767
C	4.030252	-2.92493	-1.33377
C	3.950873	-1.6779	-0.70353
C	0.999692	0.304645	0.507875
C	1.346339	0.852419	-0.73835
C	0.407397	0.933929	-1.77398
C	-0.89937	0.474521	-1.58278
C	-1.26045	-0.07184	-0.34571
C	-0.31747	-0.15735	0.68258
C	6.876215	-3.48153	6.363825
C	7.962702	-3.86314	5.55299
C	9.081908	-4.49648	6.101303
C	9.125844	-4.77898	7.472102
C	8.046903	-4.41933	8.286721
C	6.932043	-3.77361	7.737953
C	4.115726	-2.76455	6.804059
C	3.783975	-1.70961	7.670074
C	2.745025	-1.84845	8.598641
C	2.027232	-3.04651	8.680738
C	2.349843	-4.10568	7.823811
C	3.379018	-3.96105	6.888502
H	1.59273	-0.61969	2.312629
H	3.758321	0.552853	0.537806
H	7.424419	-1.07603	7.074774
H	8.149158	1.283115	6.991156
H	7.092097	2.864448	5.360386
H	5.310702	2.043551	3.834324

H	1.731964	3.87826	-0.42921
H	-0.23603	5.216124	-1.27423
H	-2.05438	5.839604	0.366672
H	-1.81336	5.122113	2.749016
H	2.764876	-2.66385	2.333729
H	2.888099	-4.87893	1.218259
H	3.705343	-5.05851	-1.13661
H	4.394948	-2.99116	-2.3637
H	4.256477	-0.77929	-1.24944
H	2.356949	1.22678	-0.91392
H	0.703264	1.359921	-2.73799
H	-1.63126	0.536998	-2.39418
H	-2.27781	-0.44152	-0.18297
H	-0.606	-0.59712	1.643348
H	7.930751	-3.66416	4.475979
H	9.917938	-4.78051	5.454256
H	9.996633	-5.28334	7.902537
H	8.071834	-4.63937	9.358854
H	6.09969	-3.49504	8.389997
H	4.33566	-0.76692	7.621219
H	2.496497	-1.01321	9.261187
H	1.215557	-3.15351	9.407076
H	1.790905	-5.04543	7.876047
H	3.610348	-4.79053	6.211174
N	1.895039	1.358732	2.618261
N	4.383152	-0.03328	2.372989
H	5.24815	0.434759	2.126537
Ag	0.157033	3.705868	4.619275
Ag	1.421479	1.074424	4.714064
O	0.797824	1.180192	6.775198
C	0.284205	2.201895	7.309744
O	-0.0131	3.285175	6.728146
C	-0.01973	2.13632	8.799839
H	0.464013	2.984314	9.314581
H	-1.10828	2.239143	8.955584
H	0.325264	1.188097	9.23819

(B) Without deprotonation of amide protone

Group(B)-1

B3LYP/def2-SV(P)/CPCM(THF)

B3LYP/def2-SV(P)/CPCM(THF) free energy: -3345.29866943522 (a.u.)

C	3.300812	0.937198	-0.0117
C	4.605359	0.523254	0.752236
C	4.68002	-0.7822	2.845014
C	4.309764	-2.07248	3.522511
O	5.228047	0.126252	3.47338
C	3.890238	-2.0802	4.877539
C	3.567217	-3.31185	5.470584
C	3.677498	-4.51255	4.761667
C	4.106071	-4.50119	3.433507
C	4.413323	-3.28428	2.820998
P	3.691186	-0.51304	5.85718
S	1.071631	-0.29205	0.982861
C	-0.30537	0.107234	-0.13321
O	1.712123	-1.50426	0.428091
C	-0.65731	-0.78563	-1.14535
C	-1.75101	-0.43616	-1.94625
C	-2.41745	0.764234	-1.69382
C	-1.96537	1.577841	-0.64588
N	-0.92473	1.251606	0.123623
O	0.523375	-0.30554	2.345442
C	5.795298	0.373344	-0.18983
C	5.864017	-0.69291	-1.10251
C	6.952679	-0.82107	-1.97031
C	7.992412	0.116324	-1.93754
C	7.935559	1.179146	-1.03028
C	6.84414	1.304065	-0.1629
C	3.456579	2.272103	-0.72796
C	3.640947	3.462345	-0.0039
C	3.781408	4.685132	-0.66753
C	3.743197	4.736042	-2.06632
C	3.560817	3.556841	-2.79539
C	3.417772	2.334497	-2.12857
C	2.622255	-1.00625	7.277912

C	1.227606	-0.90852	7.12381
C	0.370317	-1.27351	8.165996
C	0.89748	-1.72826	9.380301
C	2.283784	-1.81981	9.546151
C	3.143316	-1.46288	8.501077
C	5.331394	-0.17564	6.618449
C	5.481245	1.004879	7.369308
C	6.71153	1.301618	7.96513
C	7.800198	0.43488	7.812805
C	7.653604	-0.73707	7.063436
C	6.424517	-1.04314	6.468493
H	3.106795	0.165196	-0.77079
H	4.841068	1.308578	1.484146
H	3.224302	-3.34326	6.506662
H	3.429399	-5.4563	5.256006
H	4.20498	-5.43504	2.872868
H	4.765493	-3.27873	1.786188
H	-0.09828	-1.71024	-1.29525
H	-2.07408	-1.09661	-2.75609
H	-3.27511	1.073027	-2.29706
H	-2.46275	2.528372	-0.42185
H	5.064968	-1.44083	-1.13539
H	6.99037	-1.65847	-2.67403
H	8.845589	0.015567	-2.61551
H	8.745445	1.914351	-0.99312
H	6.808471	2.137056	0.546283
H	3.662557	3.441992	1.090107
H	3.921893	5.602919	-0.08822
H	3.854792	5.693085	-2.58518
H	3.528451	3.585562	-3.88893
H	3.275855	1.416215	-2.7073
H	0.806086	-0.53843	6.183091
H	-0.71241	-1.19223	8.031671
H	0.227457	-2.00542	10.19982
H	2.701714	-2.17105	10.49449
H	4.224379	-1.54023	8.64367
H	4.642439	1.698612	7.506267
H	6.817819	2.221136	8.549104
H	8.762447	0.673633	8.276309

H	8.499631	-1.41985	6.938028
H	6.325715	-1.96024	5.882448
N	2.140737	0.947708	0.882827
H	2.033735	1.672851	1.617007
N	4.360586	-0.68775	1.528648
H	3.724819	-1.38008	1.133904
Ag	1.963082	3.771274	6.233536
O	1.164877	3.775598	9.015046
C	2.34657	3.413478	9.033668
O	3.094621	3.30123	7.986815
C	3.028678	3.042976	10.34926
H	2.370817	3.258447	11.20525
H	3.278419	1.966512	10.34561
H	3.977786	3.595833	10.45958
Ag	2.736438	1.312015	4.583276
O	1.924104	2.715932	3.104388
C	1.13305	3.694407	3.302028
O	0.933506	4.2484	4.411294
C	0.39095	4.224221	2.088824
H	-0.09445	3.387041	1.556988
H	-0.35537	4.978037	2.380149
H	1.115035	4.680558	1.390252

Group(B)-2

B3LYP/def2-SV(P)/CPCM(THF)

B3LYP/def2-SV(P)/CPCM(THF) free energy: -3345.29468546148 (a.u.)

C	2.760604	0.448552	0.153506
C	4.175164	0.456267	0.831426
C	4.616563	-0.46029	3.099839
C	4.736887	-1.69987	3.944779
O	4.847736	0.651176	3.573827
C	4.749046	-1.60901	5.364851
C	4.825954	-2.80318	6.103303
C	4.929631	-4.05094	5.481609
C	4.967579	-4.13166	4.090091
C	4.870937	-2.96094	3.337466
P	4.590422	0.005782	6.285371

S	1.013472	-0.80464	1.833507
C	-0.75731	-0.57365	1.508347
O	1.462211	-1.97917	1.068096
C	-1.40836	-1.40159	0.596748
C	-2.77379	-1.17028	0.39245
C	-3.39944	-0.13989	1.096682
C	-2.63935	0.62659	1.988784
N	-1.33624	0.408524	2.191798
O	1.16587	-0.78929	3.299073
C	5.324006	0.40358	-0.16916
C	5.605506	-0.76758	-0.89255
C	6.658781	-0.80409	-1.81125
C	7.448932	0.332706	-2.02083
C	7.178857	1.50284	-1.30405
C	6.124226	1.535062	-0.3845
C	2.590731	1.527137	-0.90912
C	2.241654	2.84444	-0.57041
C	2.109933	3.823417	-1.56115
C	2.322537	3.500443	-2.90604
C	2.661452	2.188659	-3.25416
C	2.791693	1.211078	-2.26198
C	4.602468	-0.51142	8.070052
C	3.376928	-0.57022	8.758433
C	3.33704	-0.94471	10.10709
C	4.518607	-1.25369	10.78826
C	5.7445	-1.18585	10.11537
C	5.786529	-0.81718	8.767407
C	6.228875	0.841745	6.134487
C	6.304191	2.196885	6.496879
C	7.52287	2.880871	6.44019
C	8.679748	2.219663	6.012385
C	8.610871	0.871298	5.64475
C	7.393169	0.184366	5.707095
H	2.634352	-0.52724	-0.33795
H	4.264133	1.383729	1.414312
H	4.812827	-2.76726	7.193232
H	4.994899	-4.95547	6.093425
H	5.074714	-5.09691	3.587371
H	4.928949	-3.04141	2.249641

H	-0.86699	-2.19244	0.07551
H	-3.33784	-1.7911	-0.30934
H	-4.46315	0.072321	0.964066
H	-3.09395	1.441652	2.56137
H	5.002528	-1.66896	-0.74077
H	6.864109	-1.72538	-2.3652
H	8.274459	0.304191	-2.73872
H	7.793491	2.395425	-1.45674
H	5.921086	2.453788	0.174949
H	2.049069	3.111367	0.472326
H	1.834396	4.84417	-1.27811
H	2.217614	4.266729	-3.68036
H	2.820492	1.921131	-4.30344
H	3.053229	0.186927	-2.54555
H	2.444119	-0.30255	8.250618
H	2.375552	-0.98373	10.62841
H	4.48673	-1.53948	11.84429
H	6.674867	-1.41855	10.64278
H	6.752759	-0.76776	8.258136
H	5.402196	2.7273	6.820613
H	7.565448	3.936738	6.724673
H	9.632705	2.755361	5.962018
H	9.51046	0.34763	5.306431
H	7.35563	-0.86857	5.414176
N	1.717169	0.536869	1.177959
H	1.712389	1.355878	1.810183
N	4.245349	-0.63356	1.799477
H	3.922861	-1.54671	1.498811
Ag	-0.03632	1.593626	4.182629
O	0.719103	1.02884	7.262137
C	-0.50952	0.842212	7.074697
O	-1.12182	0.948048	5.970401
C	-1.34375	0.409084	8.278358
H	-0.98191	0.899993	9.196481
H	-2.41226	0.627453	8.125442
H	-1.2294	-0.68238	8.41592
Ag	2.563222	1.380791	5.869021
O	2.517143	3.421591	4.771108
C	2.183619	3.577142	3.57661

O	1.372376	2.823172	2.934662
C	2.799224	4.734449	2.799978
H	3.227869	5.481718	3.485315
H	3.610539	4.344378	2.158219
H	2.052027	5.20725	2.141875

Group(B)⁻3

B3LYP/def2-SV(P)/CPCM(THF)

B3LYP/def2-SV(P)/CPCM(THF) free energy: -3345.29409158866 (a.u.)

C	2.791344	0.873636	0.023486
C	4.077418	0.515951	0.847563
C	4.024996	-0.24548	3.212213
C	3.685104	-1.36453	4.158999
O	4.554192	0.787144	3.621211
C	3.627915	-1.13164	5.561757
C	3.266935	-2.2046	6.395905
C	3.007339	-3.48071	5.887753
C	3.11318	-3.71656	4.517515
C	3.449469	-2.66134	3.669167
P	3.931753	0.539986	6.332522
S	0.607076	0.400694	1.588446
C	-0.96398	1.151199	1.076375
O	0.708379	-0.92024	0.947056
C	-1.77568	0.499798	0.150489
C	-2.97294	1.134722	-0.2006
C	-3.28376	2.365103	0.381497
C	-2.38738	2.921949	1.302102
N	-1.24468	2.31925	1.64489
O	0.646378	0.499842	3.058176
C	5.228079	0.010207	-0.01516
C	5.190822	-1.26418	-0.60561
C	6.24946	-1.71502	-1.39988
C	7.364993	-0.89682	-1.61573
C	7.413594	0.372292	-1.03014
C	6.352235	0.819623	-0.23502
C	3.056031	1.835811	-1.12798
C	3.099268	3.225336	-0.93038

C	3.363896	4.088769	-1.99896
C	3.586634	3.577035	-3.2822
C	3.536098	2.194685	-3.49078
C	3.270438	1.333257	-2.42108
C	3.66055	0.234266	8.145238
C	2.437631	0.633526	8.714672
C	2.189903	0.432841	10.07815
C	3.162147	-0.15722	10.89186
C	4.387385	-0.54628	10.33709
C	4.635186	-0.35176	8.974923
C	5.757502	0.804248	6.273585
C	6.677444	-0.22295	6.010878
C	8.051755	0.04138	6.011041
C	8.521797	1.332173	6.277332
C	7.61001	2.360788	6.540444
C	6.236115	2.098913	6.534511
H	2.401381	-0.06113	-0.4053
H	4.407519	1.426672	1.366538
H	3.191458	-2.05092	7.47294
H	2.736631	-4.28999	6.572261
H	2.93934	-4.71452	4.105519
H	3.553325	-2.87118	2.60219
H	-1.48064	-0.46217	-0.27121
H	-3.65209	0.669194	-0.9204
H	-4.20809	2.892247	0.132749
H	-2.59606	3.883636	1.782226
H	4.33065	-1.92257	-0.44581
H	6.203272	-2.71108	-1.85102
H	8.194635	-1.24993	-2.23596
H	8.283022	1.017962	-1.18833
H	6.399186	1.813164	0.222081
H	2.90591	3.645912	0.060175
H	3.391088	5.169184	-1.82598
H	3.792002	4.253537	-4.11763
H	3.69847	1.782639	-4.49162
H	3.229114	0.253734	-2.59574
H	1.676143	1.124708	8.099278
H	1.23394	0.75065	10.50586
H	2.969992	-0.30725	11.95885

H	5.156737	-1.00192	10.96829
H	5.597986	-0.66021	8.558589
H	6.326203	-1.23663	5.798784
H	8.75813	-0.76799	5.801505
H	9.596811	1.537038	6.27604
H	7.967568	3.374798	6.744286
H	5.530317	2.913806	6.72848
N	1.752739	1.384754	0.921267
H	1.962002	2.221504	1.492711
N	3.722595	-0.43481	1.896243
H	3.153015	-1.23136	1.632785
Ag	0.23061	3.208684	3.681124
O	0.527698	2.798572	6.859007
C	-0.68195	2.946026	6.55184
O	-1.14234	3.093596	5.380461
C	-1.7033	2.905335	7.686286
H	-2.64198	3.404622	7.399332
H	-1.93117	1.848855	7.921152
H	-1.28881	3.367379	8.597004
Ag	2.476643	2.427806	5.620109
O	3.16072	4.249798	4.360239
C	2.978709	4.363186	3.128984
O	2.019829	3.823442	2.474576
C	3.977675	5.184027	2.323142
H	3.462306	5.790273	1.560334
H	4.574283	5.832515	2.982947
H	4.66434	4.496931	1.795385

Group(B)-4

B3LYP/def2-SV(P)/CPCM(THF)

B3LYP/def2-SV(P)/CPCM(THF) free energy: -3345.29363842597 (a.u.)

C	2.934454	1.606705	0.635304
C	3.830037	1.054603	1.788186
C	3.305934	0.210861	4.053
C	2.50975	-0.76128	4.882444
O	4.19404	0.893426	4.562593
C	2.47225	-0.63875	6.299995

C	1.71146	-1.57184	7.025939
C	1.030112	-2.618	6.396492
C	1.099515	-2.75681	5.010774
C	1.836017	-1.83198	4.26904
P	3.300748	0.756904	7.219279
S	0.387557	2.612297	0.641371
C	-0.6557	1.548021	1.69077
O	-0.05704	3.990751	0.877601
C	-1.35842	0.495596	1.105543
C	-2.18807	-0.26526	1.937851
C	-2.27485	0.065274	3.290801
C	-1.52356	1.144833	3.771419
N	-0.72441	1.870592	2.980302
O	0.342143	2.022279	-0.70321
C	5.015577	0.233356	1.287193
C	4.828306	-1.03401	0.711201
C	5.919907	-1.78007	0.25741
C	7.218556	-1.26978	0.376372
C	7.415937	-0.0117	0.954177
C	6.32026	0.731992	1.407592
C	3.742399	2.307214	-0.45477
C	3.997246	1.663053	-1.67399
C	4.74629	2.29286	-2.67406
C	5.250953	3.580728	-2.46631
C	4.996537	4.234936	-1.25513
C	4.246165	3.602763	-0.25886
C	2.794989	0.475581	8.983092
C	1.719833	1.225075	9.494737
C	1.304739	1.05865	10.8211
C	1.964519	0.151466	11.65663
C	3.043033	-0.59028	11.1604
C	3.455862	-0.42996	9.833936
C	5.099385	0.357318	7.270213
C	5.609713	-0.9225	7.002307
C	6.985047	-1.16636	7.087343
C	7.863017	-0.13673	7.444055
C	7.360244	1.141588	7.711919
C	5.986467	1.388064	7.621361
H	2.414601	0.756221	0.166429

H	4.226999	1.914761	2.346805
H	1.648443	-1.4907	8.111857
H	0.456255	-3.32858	6.998568
H	0.591075	-3.58206	4.504488
H	1.903241	-1.97545	3.187991
H	-1.25907	0.290528	0.038949
H	-2.76108	-1.10176	1.527551
H	-2.91548	-0.49911	3.972905
H	-1.56492	1.452956	4.820957
H	3.821146	-1.45329	0.617099
H	5.755939	-2.76543	-0.18983
H	8.073717	-1.85416	0.022956
H	8.427408	0.393079	1.058209
H	6.482578	1.713485	1.864181
H	3.601608	0.657393	-1.8478
H	4.930952	1.775887	-3.62087
H	5.835029	4.076473	-3.24785
H	5.380078	5.245778	-1.08535
H	4.040899	4.134325	0.674817
H	1.211202	1.958848	8.860301
H	0.467791	1.650772	11.2039
H	1.644498	0.027342	12.69586
H	3.569195	-1.29698	11.80965
H	4.300956	-1.01641	9.463702
H	4.936287	-1.73639	6.719116
H	7.37123	-2.16771	6.872786
H	8.938329	-0.32924	7.508856
H	8.04003	1.954416	7.985273
H	5.603655	2.395477	7.817721
N	1.915925	2.47306	1.226941
H	2.206146	3.216566	1.883421
N	3.007173	0.296063	2.727246
H	2.214624	-0.21635	2.360251
Ag	0.668227	3.566869	4.301589
O	0.663707	3.901257	7.532193
C	-0.51411	3.804687	7.105841
O	-0.85249	3.54174	5.911298
C	-1.6491	3.969424	8.113665
H	-1.34555	4.631488	8.9401

H	-2.56023	4.356033	7.629824
H	-1.8871	2.977865	8.542583
Ag	2.592773	3.013254	6.516843
O	3.575011	4.679506	5.274399
C	3.466097	4.791665	4.033483
O	2.532226	4.278507	3.32836
C	4.536168	5.572417	3.281929
H	5.263131	4.85892	2.851862
H	4.093866	6.144209	2.450343
H	5.078167	6.247474	3.96201

Group(B)‑5

B3LYP/def2-SV(P)/CPCM(THF)

B3LYP/def2-SV(P)/CPCM(THF) free energy: -3345.28947712636 (a.u.)

C	3.011857	0.024856	0.648902
C	4.514702	0.366282	0.945171
C	5.463648	-0.04894	3.200812
C	5.897393	-1.11477	4.171956
O	5.528214	1.144217	3.485231
C	5.559622	-1.01651	5.545318
C	6.00896	-2.03004	6.409048
C	6.789825	-3.09473	5.94592
C	7.128147	-3.17592	4.593312
C	6.674884	-2.1906	3.712758
P	4.438451	0.35866	6.132849
S	1.447616	-1.23261	2.493924
C	-0.21396	-1.2078	1.749447
O	2.102306	-2.45584	2.011443
C	-0.57174	-2.17894	0.816945
C	-1.85824	-2.1008	0.270726
C	-2.70551	-1.06988	0.679299
C	-2.24123	-0.15067	1.627192
N	-1.01343	-0.22173	2.154062
O	1.252608	-1.00437	3.935302
C	5.393167	0.314977	-0.29938
C	5.699781	-0.90651	-0.92281
C	6.509918	-0.94418	-2.06168

C	7.026732	0.242103	-2.5967
C	6.729171	1.463047	-1.98316
C	5.919213	1.496406	-0.84215
C	2.39991	0.900537	-0.43923
C	1.906492	2.184803	-0.15475
C	1.370391	2.983404	-1.17065
C	1.316602	2.510116	-2.48664
C	1.796875	1.229177	-2.77837
C	2.332212	0.432068	-1.76081
C	3.763503	-0.37053	7.702349
C	2.668386	-1.24746	7.563737
C	2.057497	-1.81425	8.685892
C	2.516588	-1.49718	9.970882
C	3.591365	-0.61528	10.12223
C	4.21246	-0.05803	8.996788
C	5.621441	1.641238	6.754445
C	5.11933	2.94933	6.888812
C	5.935968	3.984527	7.356386
C	7.27358	3.730995	7.681488
C	7.787203	2.437151	7.538178
C	6.966685	1.39924	7.080815
H	2.986812	-1.01854	0.299684
H	4.555751	1.383597	1.358131
H	5.744652	-1.99057	7.468657
H	7.133956	-3.86028	6.648161
H	7.743217	-4.00087	4.221931
H	6.947722	-2.24633	2.654955
H	0.130679	-2.96554	0.538408
H	-2.1901	-2.83981	-0.46392
H	-3.71652	-0.97275	0.276498
H	-2.8773	0.668508	1.976535
H	5.30978	-1.84692	-0.51918
H	6.739455	-1.90497	-2.53281
H	7.661736	0.213087	-3.48753
H	7.131546	2.395707	-2.39062
H	5.69359	2.455924	-0.36615
H	1.916578	2.56275	0.871627
H	0.988591	3.98037	-0.92958
H	0.895835	3.135198	-3.28033

H	1.751693	0.844357	-3.80193
H	2.703181	-0.56948	-1.99956
H	2.289169	-1.48612	6.563458
H	1.212176	-2.49782	8.557414
H	2.033096	-1.93245	10.85108
H	3.953889	-0.359	11.12296
H	5.054842	0.625513	9.133438
H	4.082165	3.164326	6.608294
H	5.527877	4.995364	7.456028
H	7.916144	4.541375	8.039883
H	8.833784	2.23177	7.785204
H	7.383649	0.39354	6.977523
N	2.263593	0.059197	1.904715
H	2.120924	0.947047	2.420851
N	5.011023	-0.50808	1.999377
H	4.843943	-1.50552	1.908422
Ag	-0.40477	1.22672	4.081602
O	-1.61039	2.269482	7.221619
C	-2.32018	1.384409	6.650608
O	-2.11057	0.877495	5.520378
C	-3.55522	0.931299	7.419666
H	-3.30834	0.773607	8.482654
H	-4.31895	1.729199	7.374049
H	-3.97696	0.011219	6.987686
Ag	0.113663	3.067829	6.251393
O	1.801605	3.78081	5.145182
C	2.126671	3.292713	4.025195
O	1.504261	2.36916	3.419943
C	3.375631	3.849246	3.363555
H	3.168418	4.103313	2.310086
H	3.741241	4.740505	3.895054
H	4.159595	3.070104	3.372628

Group(B)-6

B3LYP/def2-SV(P)/CPCM(THF)

B3LYP/def2-SV(P)/CPCM(THF) free energy: -3345.27570799048 (a.u.)

C	2.548291	1.2109	1.419855

C	3.797666	0.796681	2.264455
C	3.867587	0.349359	4.713099
C	3.526305	-0.66361	5.769339
O	4.628876	1.297091	5.021879
C	3.076385	-0.26624	7.052857
C	2.851708	-1.2669	8.013211
C	3.079481	-2.61748	7.7274
C	3.52141	-2.99841	6.458314
C	3.736406	-2.02201	5.482645
P	2.666001	1.524832	7.373307
S	0.221695	2.523211	1.798285
C	-0.37803	3.269273	3.347562
O	0.037301	3.545607	0.762207
C	-0.54827	4.649498	3.390042
C	-1.02524	5.209352	4.58021
C	-1.30964	4.366357	5.653949
C	-1.10347	2.991403	5.509691
N	-0.63973	2.44987	4.371978
O	-0.42772	1.212588	1.614209
C	4.778642	-0.08902	1.498804
C	4.473887	-1.43228	1.216288
C	5.382645	-2.2298	0.513491
C	6.606451	-1.70018	0.086129
C	6.91857	-0.36686	0.369621
C	6.009342	0.43152	1.073286
C	2.898712	1.644088	-0.00088
C	2.669544	0.767144	-1.07179
C	3.001017	1.131456	-2.38143
C	3.566975	2.384477	-2.63735
C	3.790058	3.270765	-1.57687
C	3.456887	2.905484	-0.2688
C	1.444858	1.378058	8.763673
C	0.186483	0.814136	8.469069
C	-0.81522	0.744406	9.440294
C	-0.58585	1.256945	10.72421
C	0.65325	1.828983	11.02555
C	1.662392	1.88684	10.05472
C	4.178757	2.182753	8.215063
C	5.141436	1.384821	8.856369

C	6.267597	1.966788	9.449262
C	6.445242	3.354759	9.415496
C	5.492553	4.160033	8.78129
C	4.372252	3.576142	8.179317
H	1.894017	0.328472	1.362078
H	4.328452	1.718109	2.546804
H	2.487037	-0.99023	9.005392
H	2.907779	-3.3716	8.501472
H	3.700597	-4.05207	6.225341
H	4.086383	-2.31476	4.489856
H	-0.30881	5.255719	2.516466
H	-1.1722	6.289763	4.660808
H	-1.68656	4.757995	6.601496
H	-1.31091	2.304301	6.33293
H	3.525877	-1.86271	1.554937
H	5.133185	-3.27394	0.299945
H	7.316263	-2.32726	-0.46227
H	7.875025	0.055549	0.04629
H	6.262538	1.473454	1.294202
H	2.224432	-0.21426	-0.88012
H	2.812122	0.434146	-3.20356
H	3.826261	2.673572	-3.66062
H	4.221987	4.257917	-1.76919
H	3.610754	3.624533	0.541676
H	-0.00892	0.409774	7.468464
H	-1.78088	0.29241	9.192645
H	-1.37149	1.209749	11.48456
H	0.843362	2.230927	12.02585
H	2.626755	2.332067	10.31288
H	5.018656	0.29888	8.892423
H	7.010529	1.330662	9.940714
H	7.32698	3.80765	9.879326
H	5.624584	5.245919	8.746241
H	3.639381	4.213529	7.672819
N	1.815251	2.238296	2.185192
H	2.306104	3.144967	2.330443
N	3.365058	0.131108	3.494065
H	2.661621	-0.62666	3.380991
Ag	-0.36739	0.247934	4.211874

O	1.537895	-1.89905	2.917779
C	0.604808	-2.47303	3.520152
O	-0.29396	-1.89059	4.213848
C	0.530267	-3.99558	3.474109
H	-0.4867	-4.35853	3.690208
H	0.867555	-4.36781	2.493074
H	1.214684	-4.40496	4.240619
Ag	4.584376	3.396092	4.494156
O	4.4794	5.44295	3.915303
C	3.586664	5.709386	3.038091
O	2.803348	4.882348	2.530775
C	3.537679	7.164199	2.581368
H	3.680063	7.847884	3.434513
H	4.366633	7.345033	1.872248
H	2.587648	7.384901	2.070739

(C) Deprotonation of both amide protone

Group(C)-1

B3LYP/def2-SV(P)/CPCM(THF)

B3LYP/def2-SV(P)/CPCM(THF) free energy: -2887.50684480927 (a.u.)

C	2.26059	1.732494	1.011571
C	3.477603	0.744807	1.195027
C	4.117372	-0.68237	2.985612
C	3.776888	-1.79188	3.965255
O	5.225219	-0.127	3.127943
C	3.664274	-1.52454	5.35295
C	3.432411	-2.58865	6.240418
C	3.329978	-3.90388	5.774626
C	3.43987	-4.16634	4.406321
C	3.65592	-3.1134	3.511268
P	3.692747	0.228317	5.95336
S	0.196139	2.633676	2.455299
C	-1.12762	1.429041	1.946657
O	-0.06263	2.810042	3.90907
C	-2.29587	1.971216	1.405605
C	-3.37399	1.119354	1.155113
C	-3.24611	-0.23933	1.451894

C	-2.0365	-0.6966	1.974236
N	-0.99694	0.12244	2.215819
O	-0.06706	3.793973	1.577252
C	4.005521	0.25587	-0.15125
C	3.220318	-0.55012	-0.99507
C	3.721754	-1.01907	-2.21313
C	5.024545	-0.69277	-2.61131
C	5.818168	0.10305	-1.77911
C	5.310666	0.569998	-0.56041
C	2.726131	2.999448	0.296076
C	3.54097	3.936828	0.952579
C	3.988948	5.084612	0.292454
C	3.628857	5.316431	-1.0414
C	2.816555	4.391301	-1.70465
C	2.370527	3.243712	-1.03798
C	2.87034	0.171814	7.602488
C	3.581155	-0.00719	8.801553
C	2.903819	-0.04316	10.02567
C	1.51236	0.096413	10.06529
C	0.797009	0.27779	8.875666
C	1.472566	0.321869	7.652754
C	5.44259	0.646237	6.324443
C	6.452633	-0.32493	6.403109
C	7.767676	0.050469	6.6987
C	8.084401	1.395413	6.918884
C	7.081729	2.369044	6.837298
C	5.768314	1.997336	6.535774
H	1.525464	1.232432	0.351941
H	4.281783	1.332821	1.665632
H	3.323765	-2.39372	7.310302
H	3.158638	-4.71922	6.483812
H	3.355773	-5.19166	4.032946
H	3.741333	-3.3147	2.439277
H	-2.34222	3.039287	1.191111
H	-4.30271	1.514723	0.733546
H	-4.06368	-0.94249	1.276829
H	-1.88759	-1.75505	2.204999
H	2.203634	-0.82224	-0.69355
H	3.092705	-1.64394	-2.8555

H	5.418244	-1.05977	-3.5645
H	6.839376	0.361773	-2.07724
H	5.940511	1.187309	0.08777
H	3.817752	3.770914	1.998382
H	4.621428	5.804226	0.82234
H	3.977889	6.215764	-1.55869
H	2.524485	4.563295	-2.74567
H	1.731421	2.527265	-1.56455
H	4.668816	-0.11699	8.785302
H	3.468872	-0.18047	10.95266
H	0.985316	0.070461	11.02394
H	-0.29047	0.39581	8.899488
H	0.907324	0.481153	6.728041
H	6.217553	-1.37738	6.224642
H	8.54888	-0.71385	6.754275
H	9.114205	1.686327	7.147733
H	7.323656	3.423669	7.000248
H	4.992781	2.766935	6.458207
N	1.653587	1.996487	2.324751
N	3.176248	-0.38863	2.077358
Ag	1.069964	-0.68926	2.488825
Ag	2.531234	1.568339	4.303678

Group(C)-2

B3LYP/def2-SV(P)/CPCM(THF)

B3LYP/def2-SV(P)/CPCM(THF) free energy: -2887.49215278382 (a.u.)

C	2.250162	1.51554	1.382342
C	3.659255	0.840037	1.235317
C	4.822858	-0.12496	3.065932
C	5.311629	-1.40145	3.73807
O	5.430879	0.939743	3.447223
C	4.991846	-1.7576	5.069942
C	5.559859	-2.91114	5.637672
C	6.447128	-3.70648	4.906151
C	6.758032	-3.36222	3.587304
C	6.188051	-2.22247	3.012237
P	3.720053	-0.76544	5.960552

S	0.692558	2.566975	3.282351
C	-0.53377	1.21153	3.126107
O	0.827924	2.817852	4.740856
C	-1.79496	1.46473	2.590259
C	-2.70604	0.404692	2.536785
C	-2.31598	-0.84934	3.012343
C	-1.02476	-1.00219	3.524933
N	-0.15197	0.013555	3.57834
O	0.126815	3.67238	2.480885
C	3.86947	0.327268	-0.19002
C	3.169222	-0.7958	-0.66432
C	3.365247	-1.26866	-1.96512
C	4.276142	-0.63116	-2.81792
C	4.987484	0.479984	-2.3549
C	4.783326	0.951833	-1.05182
C	2.078584	2.633342	0.354685
C	2.843979	3.809803	0.418438
C	2.70102	4.813672	-0.54339
C	1.785813	4.659919	-1.59278
C	1.017545	3.493977	-1.66768
C	1.165824	2.492678	-0.70049
C	3.177279	-1.81803	7.370174
C	3.692411	-1.68802	8.670511
C	3.228243	-2.51557	9.699947
C	2.251069	-3.48199	9.441081
C	1.73103	-3.61641	8.147747
C	2.184751	-2.78408	7.121355
C	4.612109	0.65807	6.71749
C	5.971367	0.599414	7.063068
C	6.612374	1.720963	7.601441
C	5.904515	2.91185	7.797999
C	4.547628	2.979027	7.459246
C	3.903002	1.858519	6.919095
H	1.497966	0.734887	1.155375
H	4.396227	1.645911	1.40572
H	5.300933	-3.19995	6.659807
H	6.88465	-4.59816	5.365183
H	7.440168	-3.98586	3.001017
H	6.421507	-1.9582	1.976426

H	-2.03997	2.464184	2.22882
H	-3.70796	0.558443	2.125709
H	-2.99607	-1.70406	2.98684
H	-0.67646	-1.96775	3.903244
H	2.471806	-1.31251	0.001013
H	2.807633	-2.14352	-2.31561
H	4.432562	-1.0028	-3.83568
H	5.707151	0.983477	-3.00867
H	5.344406	1.823475	-0.69944
H	3.556343	3.950571	1.237544
H	3.306357	5.723232	-0.47323
H	1.672132	5.446379	-2.34564
H	0.296734	3.361838	-2.48109
H	0.558039	1.58415	-0.76715
H	4.457955	-0.93868	8.888031
H	3.635413	-2.40224	10.70931
H	1.889677	-4.12726	10.24759
H	0.961554	-4.36587	7.939248
H	1.761474	-2.88325	6.115897
H	6.54059	-0.31982	6.898859
H	7.673512	1.664157	7.862461
H	6.410011	3.788493	8.214067
H	3.985048	3.904438	7.615195
H	2.839197	1.918084	6.666093
N	2.095187	1.951897	2.783431
N	3.889466	-0.27115	2.155296
Ag	2.055267	-0.11998	4.307695
Ag	3.914184	2.602692	3.98108

Group(C)‑3

B3LYP/def2-SV(P)/CPCM(THF)

B3LYP/def2-SV(P)/CPCM(THF) free energy: -2887.48993658408 (a.u.)

C	2.934519	1.459553	0.794049
C	3.366373	-0.05469	0.791196
C	3.949045	-0.9117	3.021571
C	5.133506	-1.23933	3.919746
O	2.807787	-1.24227	3.543387

C	5.243112	-0.75887	5.248072
C	6.361124	-1.11945	6.02282
C	7.350343	-1.96382	5.511799
C	7.244852	-2.43573	4.199879
C	6.151909	-2.06274	3.414367
P	3.995222	0.426594	5.923947
S	1.116857	2.958713	2.023279
C	-0.54754	2.185618	1.818705
O	1.035656	3.506719	3.406887
C	-1.51732	2.894364	1.108727
C	-2.81294	2.372779	1.055918
C	-3.08669	1.172393	1.714773
C	-2.04907	0.528311	2.390646
N	-0.80162	1.025652	2.438008
O	1.205015	3.915471	0.89952
C	2.24906	-1.0737	0.48169
C	2.53096	-2.44653	0.634009
C	1.613186	-3.42801	0.255467
C	0.37637	-3.06658	-0.29433
C	0.082493	-1.71246	-0.46703
C	1.00969	-0.73065	-0.08826
C	4.136193	2.351746	0.448871
C	4.873952	3.065799	1.402625
C	5.979967	3.839832	1.030642
C	6.36889	3.918975	-0.31027
C	5.636908	3.218756	-1.27626
C	4.533953	2.447991	-0.89652
C	2.753299	-0.57307	6.843573
C	3.010451	-1.88007	7.288083
C	2.030837	-2.59279	7.988815
C	0.788429	-2.00719	8.2553
C	0.524611	-0.70488	7.81447
C	1.499208	0.005739	7.106989
C	4.899889	1.366822	7.227551
C	5.666955	2.474994	6.822597
C	6.372583	3.231714	7.761689
C	6.308409	2.900054	9.12075
C	5.539178	1.807446	9.533441
C	4.839094	1.042202	8.592988

H	2.245134	1.579788	-0.0571
H	3.999291	-0.11138	-0.11646
H	6.468177	-0.7313	7.039086
H	8.204949	-2.24136	6.136149
H	8.0193	-3.08798	3.784243
H	6.07493	-2.40591	2.379314
H	-1.24905	3.828877	0.614487
H	-3.5981	2.901135	0.507449
H	-4.08561	0.73023	1.704688
H	-2.21658	-0.42022	2.908032
H	3.494113	-2.7477	1.05308
H	1.866538	-4.48489	0.388197
H	-0.34546	-3.8341	-0.59053
H	-0.87409	-1.4072	-0.90308
H	0.744103	0.314732	-0.25725
H	4.582397	3.02568	2.45556
H	6.538941	4.38603	1.797319
H	7.231203	4.526734	-0.60201
H	5.920205	3.27846	-2.33205
H	3.96446	1.914022	-1.66571
H	3.975402	-2.35095	7.082036
H	2.241356	-3.61225	8.32624
H	0.023637	-2.56697	8.802323
H	-0.44633	-0.2417	8.014759
H	1.278677	1.018155	6.752437
H	5.709776	2.751256	5.763423
H	6.966723	4.08935	7.431935
H	6.853577	3.497648	9.857724
H	5.481035	1.545718	10.59439
H	4.242397	0.190697	8.930503
N	2.249542	1.829922	2.036438
N	4.273651	-0.38517	1.880216
Ag	0.932048	-0.25322	3.072104
Ag	3.033253	1.720866	4.130116

Group(C)-4

B3LYP/def2-SV(P)/CPCM(THF)

B3LYP/def2-SV(P)/CPCM(THF) free energy: -2887.48696080509 (a.u.)

C	1.499206	0.317651	2.491059
C	2.935331	-0.18265	2.907302
C	2.364247	-1.65352	4.788906
C	2.807551	-2.07211	6.174403
O	1.330046	-2.26962	4.298258
C	3.566431	-1.24162	7.041713
C	3.953474	-1.74773	8.296832
C	3.61615	-3.04399	8.697222
C	2.858617	-3.85485	7.848543
C	2.454739	-3.36166	6.606745
P	3.954946	0.519599	6.611469
S	-0.50988	1.645212	3.613561
C	-0.43321	3.26906	4.432785
O	-1.22752	1.827062	2.331609
C	-1.32575	4.260508	4.028178
C	-1.28492	5.48439	4.703401
C	-0.36421	5.657306	5.739482
C	0.489182	4.598268	6.062451
N	0.452443	3.423841	5.418386
O	-1.34286	0.836582	4.61753
C	3.548206	-1.10807	1.85263
C	3.008978	-2.37899	1.57928
C	3.592529	-3.21219	0.619501
C	4.72885	-2.79559	-0.08554
C	5.278682	-1.53814	0.18255
C	4.691479	-0.70606	1.143534
C	1.564588	1.011748	1.131338
C	2.138634	2.286529	0.995212
C	2.225397	2.90584	-0.2556
C	1.733794	2.260804	-1.39739
C	1.155111	0.99309	-1.27387
C	1.073313	0.377767	-0.01951
C	4.467673	1.253401	8.242356
C	3.458155	1.784214	9.066612
C	3.769239	2.363911	10.29999
C	5.102036	2.438303	10.72272
C	6.116041	1.924922	9.907569
C	5.80186	1.33391	8.677611
C	5.531151	0.526338	5.660026

C	6.397567	-0.57774	5.622942
C	7.597422	-0.51113	4.906531
C	7.946997	0.659107	4.222661
C	7.088668	1.763929	4.25606
C	5.886154	1.696371	4.968202
H	0.86048	-0.57468	2.383755
H	3.551089	0.735187	2.904184
H	4.528416	-1.12156	8.982052
H	3.942245	-3.41166	9.674959
H	2.582587	-4.86955	8.15213
H	1.85968	-3.97911	5.930441
H	-2.01854	4.071173	3.206782
H	-1.96442	6.293097	4.419445
H	-0.29965	6.597793	6.291872
H	1.229079	4.693139	6.863004
H	2.135253	-2.71615	2.142034
H	3.15702	-4.19725	0.421244
H	5.183897	-3.44933	-0.83657
H	6.169587	-1.20073	-0.3572
H	5.130609	0.276429	1.345539
H	2.51054	2.806092	1.88345
H	2.676169	3.900222	-0.33972
H	1.797508	2.746221	-2.37651
H	0.76062	0.480761	-2.15743
H	0.615587	-0.61302	0.067472
H	2.413518	1.747205	8.737163
H	2.969287	2.767766	10.9283
H	5.34939	2.900699	11.68331
H	7.160761	1.98268	10.22882
H	6.607216	0.934275	8.055758
H	6.13389	-1.49934	6.149243
H	8.262007	-1.38039	4.882074
H	8.885461	0.708606	3.661908
H	7.351944	2.681747	3.721274
H	5.21612	2.562809	4.980425
N	1.001795	1.208138	3.554777
N	3.104676	-0.73697	4.24429
Ag	-0.52637	-1.22924	4.768721
Ag	2.110108	1.699555	5.566694

Group(C)-5

B3LYP/def2-SV(P)/CPCM(THF)

B3LYP/def2-SV(P)/CPCM(THF) free energy: -2887.48672272531 (a.u.)

C	2.35724	1.001095	1.565111
C	3.795387	0.402726	1.407473
C	4.1413	-0.24433	3.733962
C	5.088084	-0.26249	4.925366
O	3.043354	-0.82487	3.885518
C	4.642365	0.075408	6.227125
C	5.534441	-0.03765	7.307948
C	6.840593	-0.50094	7.120412
C	7.278624	-0.83818	5.836699
C	6.407429	-0.70984	4.751462
P	2.942753	0.769068	6.483212
S	2.740333	3.70339	1.899802
C	1.530288	4.735876	2.789648
O	4.051141	4.177868	2.526055
C	0.946305	5.820653	2.137507
C	0.049362	6.607878	2.867358
C	-0.21596	6.278221	4.198587
C	0.426066	5.166785	4.754689
N	1.284116	4.41092	4.059667
O	2.645574	4.053683	0.465205
C	3.81785	-0.96657	0.72171
C	2.944265	-2.01736	1.055501
C	3.033364	-3.25641	0.410934
C	3.998826	-3.47676	-0.57807
C	4.876618	-2.4423	-0.91923
C	4.781182	-1.204	-0.27541
C	1.566095	1.076071	0.256399
C	2.133205	1.413399	-0.983
C	1.351185	1.468565	-2.14156
C	-0.01968	1.192469	-2.08482
C	-0.60083	0.856397	-0.85735
C	0.189225	0.79609	0.295507
C	1.834985	-0.65262	6.851197
C	0.446796	-0.43404	6.821857
C	-0.44126	-1.47521	7.10849

C	0.049132	-2.7496	7.416697
C	1.429766	-2.97587	7.438575
C	2.319998	-1.93319	7.158582
C	3.070127	1.662003	8.09786
C	3.524545	2.993598	8.079741
C	3.643695	3.722376	9.266366
C	3.29492	3.134122	10.48828
C	2.83168	1.81472	10.51582
C	2.721667	1.080677	9.328714
H	1.810657	0.304847	2.210959
H	4.345211	1.07714	0.731951
H	5.21118	0.24087	8.313867
H	7.51273	-0.5929	7.978892
H	8.299353	-1.20017	5.678754
H	6.74213	-0.96622	3.742857
H	1.188212	6.027064	1.093748
H	-0.4367	7.468188	2.397991
H	-0.91055	6.867203	4.802523
H	0.244718	4.871605	5.793262
H	2.201946	-1.8719	1.840693
H	2.34063	-4.0585	0.686692
H	4.065255	-4.44727	-1.08023
H	5.636033	-2.59646	-1.69279
H	5.469192	-0.39894	-0.55626
H	3.197788	1.646232	-1.05544
H	1.817548	1.732156	-3.09646
H	-0.63004	1.23597	-2.99253
H	-1.67079	0.632121	-0.79726
H	-0.2716	0.521922	1.250928
H	0.053874	0.556259	6.566874
H	-1.51974	-1.29176	7.08214
H	-0.64497	-3.56723	7.63402
H	1.819253	-3.97135	7.673096
H	3.396359	-2.12357	7.172716
H	3.783931	3.467685	7.126639
H	4.001272	4.756113	9.23538
H	3.379114	3.70642	11.4172
H	2.553752	1.349834	11.46675
H	2.360606	0.049523	9.366796

N	2.320572	2.253801	2.35163
N	4.600195	0.389597	2.65058
Ag	2.240671	2.194961	4.652543
Ag	5.394673	2.40011	3.064937

Group(C)-6

B3LYP/def2-SV(P)/CPCM(THF)

B3LYP/def2-SV(P)/CPCM(THF) free energy: -2887.47296431722 (a.u.)

C	2.304026	2.50007	1.542262
C	3.286624	1.29322	1.371151
C	3.43815	-0.37233	3.193267
C	4.396241	-1.00941	4.207313
O	2.431086	-1.11087	2.937362
C	4.607313	-0.62521	5.554745
C	5.493192	-1.36951	6.356432
C	6.169528	-2.48168	5.851156
C	5.955209	-2.87131	4.526205
C	5.070313	-2.14672	3.725894
P	3.766098	0.872296	6.234115
S	-0.19954	2.956207	2.188677
C	-1.49652	1.87249	1.443003
O	-0.79361	3.225389	3.525517
C	-2.48933	2.467076	0.66103
C	-3.5307	1.664413	0.190116
C	-3.54289	0.305993	0.519872
C	-2.50284	-0.20106	1.29963
N	-1.49879	0.570263	1.749126
O	-0.11427	4.111493	1.256147
C	2.858639	0.246748	0.331282
C	1.68267	0.333932	-0.43138
C	1.396804	-0.60586	-1.4324
C	2.285004	-1.65087	-1.6973
C	3.470454	-1.74172	-0.95633
C	3.750354	-0.80297	0.038621
C	3.055819	3.720162	2.122025
C	2.656451	4.347092	3.324021
C	3.334232	5.486797	3.80592

C	4.404273	6.025622	3.092827
C	4.792016	5.427924	1.883933
C	4.130326	4.295021	1.409509
C	2.051525	0.319162	6.614377
C	1.735594	-1.02123	6.898398
C	0.41401	-1.39244	7.167376
C	-0.60392	-0.4315	7.153856
C	-0.29779	0.904235	6.869212
C	1.022121	1.276865	6.596619
C	4.56409	1.207854	7.856484
C	5.717195	2.014019	7.865106
C	6.366375	2.310228	9.066703
C	5.860396	1.818807	10.27626
C	4.706574	1.027808	10.27762
C	4.060905	0.720575	9.074548
H	2.031657	2.81241	0.515634
H	4.173684	1.755572	0.901182
H	5.658353	-1.07415	7.395523
H	6.856272	-3.04131	6.493175
H	6.474395	-3.74182	4.113493
H	4.893157	-2.4652	2.694493
H	-2.42993	3.53239	0.432802
H	-4.32442	2.095509	-0.42721
H	-4.33963	-0.35764	0.17547
H	-2.46595	-1.25939	1.572625
H	0.967526	1.138521	-0.25437
H	0.470137	-0.51231	-2.0078
H	2.061858	-2.38423	-2.47851
H	4.184349	-2.54681	-1.15872
H	4.684335	-0.88036	0.603536
H	1.75085	4.003225	3.82967
H	2.994523	5.957809	4.733426
H	4.924424	6.914593	3.461647
H	5.61587	5.854657	1.30326
H	4.440787	3.861109	0.453841
H	2.520897	-1.78216	6.902647
H	0.17903	-2.43872	7.385349
H	-1.63782	-0.72533	7.359341
H	-1.08925	1.658966	6.843339

H	1.249155	2.319914	6.354538
H	6.108025	2.416966	6.924483
H	7.264147	2.935648	9.059179
H	6.362671	2.058145	11.21852
H	4.303755	0.645255	11.22039
H	3.160866	0.100204	9.090164
N	1.13845	2.116726	2.319745
N	3.824581	0.759052	2.637546
Ag	0.468608	-0.21866	2.547482
Ag	3.879156	2.409017	4.327411

(D) Deprotonation of amide protone

Group(D)-1

B3LYP/def2-SV(P)/CPCM(THF)

B3LYP/def2-SV(P)/CPCM(THF) free energy: -3116.39001412008 (a.u.)

C	1.803564	1.741004	0.501548
C	2.935216	1.32983	1.500053
C	3.319779	0.059818	3.45188
C	3.024528	-1.1824	4.271736
O	4.374512	0.672774	3.725176
C	3.443881	-1.2743	5.622461
C	3.266649	-2.48325	6.315715
C	2.70015	-3.59741	5.686977
C	2.281152	-3.50543	4.357438
C	2.441554	-2.30341	3.662021
P	4.042485	0.236591	6.506142
S	-0.89399	2.221887	0.596767
C	-1.4262	0.518457	0.963522
O	-1.69143	3.156946	1.398763
C	-1.96556	-0.24291	-0.07257
C	-2.46044	-1.51007	0.258153
C	-2.39008	-1.93788	1.584624
C	-1.81897	-1.08577	2.538663
N	-1.34538	0.124706	2.229041
O	-0.89091	2.336483	-0.86729
C	4.117852	0.757935	0.713856
C	4.001529	-0.47317	0.046903

C	5.074969	-1.00209	-0.67752
C	6.289127	-0.30812	-0.74364
C	6.418718	0.91516	-0.07679
C	5.341465	1.439121	0.646468
C	2.29535	2.686349	-0.59006
C	2.723393	3.987882	-0.27964
C	3.177398	4.85183	-1.2815
C	3.214159	4.425125	-2.61384
C	2.790874	3.130879	-2.93454
C	2.334682	2.271866	-1.92923
C	3.910912	-0.22162	8.29809
C	2.684217	-0.0196	8.957233
C	2.53315	-0.356	10.30557
C	3.612163	-0.88925	11.02039
C	4.838738	-1.08668	10.37798
C	4.987985	-0.75708	9.025786
C	5.852709	0.409952	6.256643
C	6.458145	1.62735	6.607211
C	7.839361	1.796587	6.464773
C	8.625787	0.752027	5.966063
C	8.025364	-0.46151	5.61059
C	6.644778	-0.63373	5.754824
H	1.43966	0.814962	0.030367
H	3.288958	2.254422	1.997641
H	3.568681	-2.563	7.362287
H	2.585132	-4.53308	6.242442
H	1.834224	-4.37082	3.858332
H	2.128615	-2.21918	2.618965
H	-1.9949	0.145638	-1.09116
H	-2.8942	-2.15042	-0.515
H	-2.76733	-2.91869	1.884608
H	-1.73713	-1.3895	3.587622
H	3.061529	-1.03072	0.10133
H	4.963008	-1.962	-1.19214
H	7.13089	-0.72094	-1.30873
H	7.365725	1.463268	-0.11449
H	5.45431	2.391852	1.172715
H	2.708054	4.344035	0.755989
H	3.505462	5.862243	-1.01833

H	3.569261	5.099927	-3.39889
H	2.811723	2.787604	-3.97363
H	1.99961	1.263531	-2.19082
H	1.834972	0.409432	8.414004
H	1.57137	-0.1923	10.80102
H	3.497917	-1.14521	12.0782
H	5.688302	-1.49962	10.93053
H	5.953406	-0.91913	8.539657
H	5.848173	2.45361	6.987966
H	8.300255	2.750445	6.739013
H	9.705783	0.884944	5.849925
H	8.634497	-1.28024	5.215174
H	6.183945	-1.58223	5.465731
N	0.655687	2.297254	1.248126
N	2.440916	0.35902	2.47698
Ag	0.666974	1.113357	3.65175
O	-0.58511	2.304366	5.036254
C	-0.20706	3.312184	5.691178
O	0.983415	3.568596	6.042733
C	-1.27663	4.328779	6.068955
H	-2.19047	3.811466	6.404834
H	-0.92141	5.018611	6.849432
H	-1.54248	4.914724	5.170025
Ag	2.542474	2.072588	6.062216
H	0.791203	3.23235	1.639112

Group(D)-2

B3LYP/def2-SV(P)/CPCM(THF)

B3LYP/def2-SV(P)/CPCM(THF) free energy: -3116.38968132574 (a.u.)

C	0.749002	1.376004	2.344183
C	2.237296	0.889448	2.129602
C	2.444635	-0.59604	3.944894
C	2.599171	-2.01641	4.451821
O	2.345463	0.306629	4.866913
C	2.581232	-2.34717	5.832456
C	2.734018	-3.69662	6.209222
C	2.899405	-4.7069	5.259844

C	2.915147	-4.38071	3.901201
C	2.763542	-3.05055	3.513288
P	2.437251	-1.07043	7.171765
S	0.080544	3.889867	3.240971
C	1.432368	5.09667	3.419779
O	-1.0543	4.644349	2.696787
C	1.234592	6.385366	2.926473
C	2.280226	7.302901	3.076357
C	3.451389	6.887982	3.709022
C	3.541888	5.570316	4.173481
N	2.544719	4.684944	4.028006
O	-0.04741	3.165774	4.512592
C	2.702428	0.996243	0.682205
C	2.638193	-0.10178	-0.19134
C	3.045311	0.016528	-1.52445
C	3.528153	1.237581	-2.01054
C	3.607112	2.336888	-1.14853
C	3.201749	2.213023	0.185846
C	-0.30636	0.571237	1.601701
C	-0.63101	0.81288	0.255842
C	-1.60249	0.04791	-0.39851
C	-2.2638	-0.98147	0.280321
C	-1.94849	-1.23517	1.619474
C	-0.9816	-0.46267	2.271643
C	0.70597	-0.44575	7.160231
C	-0.3354	-1.13185	6.515416
C	-1.64377	-0.63697	6.564736
C	-1.92519	0.543951	7.260504
C	-0.89173	1.230463	7.90796
C	0.416909	0.739566	7.856145
C	2.484823	-2.06235	8.743112
C	3.719452	-2.21371	9.399535
C	3.814679	-2.94227	10.5897
C	2.67023	-3.521	11.14925
C	1.434285	-3.37051	10.51032
C	1.341717	-2.64848	9.315598
H	0.576345	1.243981	3.418845
H	2.829192	1.633119	2.710397
H	2.724637	-3.97163	7.264904

H	3.01477	-5.74465	5.587125
H	3.046073	-5.1608	3.14479
H	2.77298	-2.76886	2.459161
H	0.291605	6.652917	2.448931
H	2.17343	8.325864	2.704454
H	4.293005	7.570607	3.850078
H	4.432339	5.200098	4.689812
H	2.272299	-1.05613	0.192876
H	2.98779	-0.8521	-2.18878
H	3.849552	1.32922	-3.05301
H	3.996755	3.293365	-1.51193
H	3.284973	3.074081	0.856612
H	-0.12434	1.604944	-0.3037
H	-1.84066	0.257403	-1.44607
H	-3.02402	-1.58005	-0.23126
H	-2.46308	-2.03334	2.163814
H	-0.75174	-0.65999	3.323038
H	-0.1289	-2.05454	5.965893
H	-2.4465	-1.17872	6.055113
H	-2.94817	0.930817	7.295255
H	-1.1021	2.157179	8.450483
H	1.220648	1.289917	8.356779
H	4.620625	-1.75452	8.977986
H	4.784703	-3.04994	11.08456
H	2.74034	-4.08414	12.08498
H	0.534001	-3.81655	10.94422
H	0.367902	-2.54196	8.830351
N	0.635725	2.821451	2.101585
N	2.406019	-0.4386	2.652734
Ag	3.105926	2.426492	4.997512
O	5.002143	3.437645	5.83983
C	5.847251	3.061779	6.690859
O	5.779216	2.007652	7.391697
C	7.057453	3.96165	6.913285
H	7.783395	3.503156	7.601342
H	7.54349	4.177328	5.946266
H	6.720031	4.927053	7.33123
Ag	4.164225	0.602525	7.231342
H	0.474133	3.148715	1.151399

Group(D)-3

B3LYP/def2-SV(P)/CPCM(THF)

B3LYP/def2-SV(P)/CPCM(THF) free energy: -3116.38071455746 (a.u.)

C	2.545868	1.713273	1.676378
C	3.929285	0.973872	1.834671
C	3.310716	-0.66837	3.532294
C	3.875394	-1.52965	4.651238
O	2.114521	-0.94944	3.112724
C	4.259125	-1.06892	5.935047
C	4.855323	-1.97275	6.835149
C	5.067763	-3.30971	6.489213
C	4.663246	-3.77068	5.233968
C	4.069536	-2.88448	4.332749
P	3.826032	0.636198	6.511422
S	0.935905	3.261498	3.338336
C	-0.53322	2.509582	2.56275
O	0.79299	3.160208	4.80774
C	-1.23471	3.243076	1.605154
C	-2.39211	2.657862	1.079402
C	-2.77679	1.395132	1.533587
C	-1.98598	0.755388	2.496973
N	-0.87826	1.30637	3.001052
O	1.069611	4.590192	2.736661
C	4.264071	0.15913	0.584685
C	3.488258	-0.94818	0.193837
C	3.822091	-1.68708	-0.94573
C	4.938002	-1.33667	-1.71615
C	5.719212	-0.24152	-1.33457
C	5.382075	0.497184	-0.19396
C	2.599867	2.728812	0.547889
C	3.377429	3.89486	0.649241
C	3.466174	4.787845	-0.42152
C	2.776693	4.530879	-1.61371
C	1.996368	3.375858	-1.72394
C	1.910063	2.483382	-0.64873
C	4.226073	0.635003	8.318098
C	3.254862	0.171423	9.225045
C	3.51617	0.148	10.59779

C	4.747931	0.600469	11.08533
C	5.716048	1.07144	10.19277
C	5.459176	1.087799	8.816982
C	5.036176	1.818596	5.798015
C	4.680075	3.175892	5.735011
C	5.591515	4.123329	5.256003
C	6.86107	3.719797	4.828425
C	7.216702	2.366143	4.879681
C	6.309458	1.418017	5.361448
H	1.772428	0.963059	1.450092
H	4.670517	1.789012	1.884691
H	5.153148	-1.63326	7.829273
H	5.539818	-3.98772	7.206435
H	4.811644	-4.81805	4.953572
H	3.758174	-3.23949	3.34583
H	-0.88754	4.228892	1.294412
H	-2.98356	3.187163	0.326965
H	-3.67648	0.90589	1.151837
H	-2.25673	-0.23443	2.880089
H	2.62544	-1.23615	0.799375
H	3.205828	-2.54491	-1.23433
H	5.197382	-1.9163	-2.6078
H	6.596037	0.041655	-1.92581
H	5.999918	1.353456	0.09585
H	3.900719	4.114517	1.583768
H	4.073608	5.693271	-0.32429
H	2.845506	5.231855	-2.45152
H	1.450331	3.16648	-2.64915
H	1.298763	1.580244	-0.7423
H	2.282451	-0.17428	8.858265
H	2.750556	-0.21704	11.28889
H	4.950132	0.590497	12.16069
H	6.679917	1.430505	10.56614
H	6.227374	1.458391	8.133918
H	3.681759	3.496749	6.049799
H	5.304144	5.178101	5.211281
H	7.572881	4.459314	4.448957
H	8.205999	2.045362	4.539284
H	6.591954	0.362388	5.388746

N	2.270816	2.33621	2.984442
N	4.147277	0.192719	3.047339
Ag	0.450272	-0.98764	4.448039
O	-0.66915	0.845469	6.929658
C	-1.45906	-0.08723	6.621892
O	-1.24906	-0.99101	5.759725
C	-2.78107	-0.16635	7.37336
H	-3.59349	-0.47583	6.696087
H	-2.69528	-0.93524	8.163319
H	-3.02597	0.795964	7.848109
Ag	1.442342	1.102762	6.34144
H	2.474916	1.70686	3.762263

Group(D)-4

B3LYP/def2-SV(P)/CPCM(THF)

B3LYP/def2-SV(P)/CPCM(THF) free energy: -3116.37836145992 (a.u.)

C	3.159759	1.174295	1.468614
C	4.27685	0.140861	1.882885
C	3.113147	-0.95541	3.728535
C	3.339857	-1.7475	5.006083
O	1.906126	-0.93155	3.24953
C	3.812382	-1.21366	6.230828
C	4.061457	-2.08811	7.305777
C	3.846953	-3.46395	7.188404
C	3.354707	-3.98643	5.989956
C	3.101364	-3.12926	4.916908
P	3.916863	0.615144	6.501863
S	1.875016	3.30556	2.707034
C	0.326436	2.806476	1.881977
O	1.598726	3.485223	4.149421
C	-0.08084	3.50684	0.745858
C	-1.30075	3.13275	0.171051
C	-2.03208	2.096431	0.753734
C	-1.51576	1.464714	1.892454
N	-0.35185	1.815833	2.446598
O	2.386485	4.444024	1.940699
C	4.458747	-0.92721	0.803932

C	3.445324	-1.86012	0.516075
C	3.635378	-2.84172	-0.46168
C	4.841999	-2.91211	-1.16952
C	5.857656	-1.993	-0.88877
C	5.664226	-1.0107	0.090198
C	3.575757	1.953556	0.232849
C	4.626418	2.885687	0.275994
C	5.039834	3.54804	-0.8825
C	4.408284	3.290208	-2.10621
C	3.358408	2.36825	-2.15973
C	2.946925	1.706623	-0.99653
C	4.260476	0.800795	8.31083
C	3.174679	0.847414	9.204723
C	3.388694	0.980419	10.57957
C	4.6921	1.081594	11.07971
C	5.777689	1.04499	10.19858
C	5.565308	0.903952	8.822479
C	5.446629	1.243298	5.705458
C	6.539434	0.411364	5.413188
C	7.704344	0.952132	4.861108
C	7.787111	2.32461	4.594296
C	6.698224	3.156556	4.876889
C	5.530083	2.6172	5.426785
H	2.234065	0.618639	1.252677
H	5.207128	0.733234	1.89991
H	4.420781	-1.6916	8.257527
H	4.056042	-4.12038	8.03831
H	3.169994	-5.06021	5.887598
H	2.72215	-3.53466	3.974178
H	0.532755	4.310876	0.338561
H	-1.67129	3.648639	-0.71926
H	-2.9906	1.775481	0.338132
H	-2.06146	0.64747	2.376428
H	2.506778	-1.81906	1.074809
H	2.835101	-3.55882	-0.67189
H	4.989233	-3.68131	-1.9343
H	6.806757	-2.03875	-1.43253
H	6.46566	-0.29564	0.302925
H	5.11066	3.108611	1.230641

H	5.857672	4.273751	-0.83035
H	4.732127	3.810307	-3.01322
H	2.855106	2.161557	-3.10945
H	2.12575	0.984593	-1.0459
H	2.149474	0.781848	8.824824
H	2.532879	1.013887	11.26037
H	4.86067	1.194363	12.15501
H	6.799418	1.127601	10.58147
H	6.4252	0.876825	8.148635
H	6.478574	-0.66245	5.607937
H	8.551288	0.297162	4.63476
H	8.699757	2.744269	4.160089
H	6.753284	4.228366	4.66397
H	4.675332	3.270406	5.631446
N	2.95708	2.044	2.6424
N	4.191805	-0.46738	3.204872
Ag	0.267885	-0.24669	4.447503
O	-1.37275	0.400899	5.680821
C	-1.33208	1.434446	6.409604
O	-0.31819	2.153114	6.625413
C	-2.6206	1.83806	7.114069
H	-3.4902	1.306401	6.699314
H	-2.53274	1.595018	8.188762
H	-2.76983	2.927802	7.035639
Ag	1.782688	1.716675	6.097626
H	2.910047	1.507047	3.509633

Group(D)-5

B3LYP/def2-SV(P)/CPCM(THF)

B3LYP/def2-SV(P)/CPCM(THF) free energy: -3116.37791262969 (a.u.)

C	2.313938	1.882888	1.274048
C	3.171646	0.773482	1.983196
C	3.851818	0.145458	4.171407
C	3.492097	-0.42221	5.541466
O	5.052021	0.179964	3.870352
C	3.502049	0.325363	6.742784
C	3.270566	-0.3283	7.966194

C	3.040668	-1.70564	8.012585
C	3.033459	-2.44651	6.826835
C	3.259747	-1.80641	5.605717
P	3.722553	2.153781	6.671228
S	0.143286	2.001055	-0.31435
C	-1.11851	0.708826	-0.51642
O	-0.5772	3.273432	-0.14167
C	-2.45204	1.020885	-0.25126
C	-3.38991	0.000903	-0.44653
C	-2.94702	-1.24785	-0.88787
C	-1.57667	-1.43515	-1.11603
N	-0.67449	-0.46876	-0.93119
O	1.127689	1.892344	-1.40067
C	3.216248	-0.49221	1.11949
C	2.436672	-1.6253	1.393348
C	2.494337	-2.75464	0.567507
C	3.337063	-2.77105	-0.54871
C	4.124286	-1.64842	-0.8306
C	4.062281	-0.52385	-0.0021
C	2.533383	3.2744	1.869548
C	3.72654	3.960081	1.58144
C	3.963529	5.246044	2.078545
C	3.019385	5.874202	2.898044
C	1.824953	5.210994	3.202083
C	1.571406	3.91972	2.686378
C	3.092145	2.79251	8.276171
C	1.71812	3.072819	8.387789
C	1.188641	3.548905	9.590342
C	2.028466	3.763301	10.68991
C	3.397514	3.496294	10.58322
C	3.92941	3.011217	9.38315
C	5.533469	2.463768	6.695592
C	5.990088	3.754182	6.370857
C	7.357215	4.043109	6.382268
C	8.282523	3.042421	6.703592
C	7.835121	1.754176	7.014622
C	6.466306	1.463396	7.012322
H	2.729879	1.917294	0.257159
H	4.197162	1.177891	1.975432

H	3.264048	0.245288	8.896456
H	2.864179	-2.19585	8.974486
H	2.849652	-3.5249	6.85063
H	3.258254	-2.38766	4.678574
H	-2.73173	2.014861	0.098218
H	-4.45143	0.18319	-0.25547
H	-3.64807	-2.07019	-1.05358
H	-1.19526	-2.40379	-1.45981
H	1.780955	-1.62679	2.267609
H	1.876137	-3.62753	0.80088
H	3.384523	-3.65465	-1.19298
H	4.795169	-1.64992	-1.69559
H	4.692003	0.344059	-0.22842
H	4.474581	3.488062	0.936754
H	4.893254	5.761515	1.820058
H	3.203323	6.880179	3.285366
H	1.057931	5.704826	3.805683
H	0.579054	3.479493	2.827164
H	1.056556	2.920408	7.528306
H	0.11805	3.761281	9.665415
H	1.615573	4.143902	11.62905
H	4.058725	3.66518	11.43851
H	5.000772	2.805151	9.312852
H	5.274094	4.538641	6.103589
H	7.701274	5.050732	6.130372
H	9.353625	3.266168	6.70469
H	8.554446	0.966943	7.259691
H	6.128584	0.451737	7.251042
N	0.90831	1.511805	1.098806
N	2.816072	0.501536	3.374875
Ag	0.821457	0.382424	4.108232
O	-1.34796	1.929884	2.899365
C	-1.86179	1.331019	3.865826
O	-1.23628	0.535427	4.652291
C	-3.35195	1.504192	4.138521
H	-3.91172	0.770499	3.528822
H	-3.68296	2.511439	3.838609
H	-3.59387	1.322141	5.197615
Ag	2.534266	2.91062	4.666792

H	0.25333	1.624061	1.893368

Group(D)-6

B3LYP/def2-SV(P)/CPCM(THF)

B3LYP/def2-SV(P)/CPCM(THF) free energy: -3116.37786800535 (a.u.)

C	2.360864	1.01425	1.859165
C	3.759771	0.401523	2.249847
C	3.13293	-0.85131	4.228298
C	3.631437	-1.35983	5.57162
O	2.038379	-1.39025	3.770425
C	3.810079	-0.56655	6.730386
C	4.349195	-1.15557	7.889461
C	4.707728	-2.50595	7.916148
C	4.510567	-3.29538	6.779839
C	3.971477	-2.72254	5.625892
P	3.188388	1.175133	6.772205
S	0.770115	2.905497	3.143928
C	-0.18075	3.503034	1.713543
O	-0.03165	1.819934	3.744726
C	-0.15926	4.866211	1.41198
C	-0.91851	5.280121	0.31253
C	-1.64035	4.325728	-0.40817
C	-1.57707	2.98594	-0.00364
N	-0.85686	2.581286	1.045611
O	1.124981	4.088419	3.941333
C	4.241979	-0.68826	1.286117
C	3.548024	-1.89934	1.105135
C	4.040541	-2.88356	0.24233
C	5.237204	-2.68209	-0.45688
C	5.938277	-1.48511	-0.28314
C	5.441514	-0.50116	0.580293
C	2.237936	1.257876	0.358748
C	2.931803	2.303317	-0.27273
C	2.837199	2.496543	-1.6551
C	2.044639	1.643805	-2.43172
C	1.34614	0.600609	-1.8135
C	1.442706	0.413413	-0.4309

C	3.250043	1.641732	8.563241
C	4.341322	2.316755	9.13632
C	4.335898	2.648906	10.49622
C	3.244322	2.307909	11.30112
C	2.152034	1.63602	10.73959
C	2.152292	1.312275	9.380012
C	4.48086	2.235348	6.006887
C	5.823822	1.832449	5.925434
C	6.784857	2.690732	5.382427
C	6.413965	3.957048	4.913877
C	5.075916	4.359901	4.985401
C	4.111284	3.502017	5.526248
H	1.579238	0.318213	2.187169
H	4.467606	1.240099	2.113003
H	4.48458	-0.55511	8.791783
H	5.13069	-2.93832	8.827903
H	4.77646	-4.35698	6.790058
H	3.818038	-3.33667	4.733636
H	0.416321	5.567857	2.017446
H	-0.94627	6.335933	0.028288
H	-2.24736	4.609864	-1.27172
H	-2.12961	2.211469	-0.54782
H	2.623313	-2.07015	1.659542
H	3.483624	-3.81771	0.114757
H	5.620151	-3.45443	-1.13156
H	6.876596	-1.31314	-0.82041
H	6.000202	0.432139	0.708266
H	3.555393	2.986194	0.313412
H	3.38601	3.317845	-2.12668
H	1.969114	1.794091	-3.51319
H	0.718182	-0.06908	-2.40958
H	0.88773	-0.4008	0.044746
H	5.203717	2.589554	8.523136
H	5.191997	3.177835	10.92619
H	3.241286	2.569881	12.3636
H	1.290575	1.370224	11.35963
H	1.284865	0.799536	8.950473
H	6.121931	0.841719	6.278861
H	7.828439	2.366685	5.322196

H	7.167429	4.62652	4.487425
H	4.776372	5.344028	4.611846
H	3.063785	3.815514	5.54844
N	2.202314	2.24773	2.651576
N	3.927126	-0.02246	3.633943
Ag	0.282377	-1.4841	4.957931
O	-1.53699	-1.34397	6.056584
C	-1.92426	-0.26276	6.598258
O	-1.27269	0.810202	6.666253
C	-3.33002	-0.27766	7.184535
H	-3.55635	-1.26048	7.628354
H	-3.45435	0.518531	7.934485
H	-4.05601	-0.1043	6.368655
Ag	0.84878	1.216698	6.074561
H	2.936635	2.951635	2.585373

Group(D)-7

B3LYP/def2-SV(P)/CPCM(THF)

B3LYP/def2-SV(P)/CPCM(THF) free energy: -3116.37713516223 (a.u.)

C	2.114912	1.447335	0.80681
C	3.680353	1.365112	0.932057
C	3.975607	-0.20264	2.781934
C	4.998256	-0.72173	3.793595
O	3.048314	-0.97365	2.47149
C	4.740418	-0.99535	5.154606
C	5.690132	-1.71249	5.907508
C	6.898896	-2.12302	5.343787
C	7.176418	-1.81179	4.008072
C	6.232896	-1.12077	3.246919
P	3.176649	-0.42745	5.953857
S	0.010717	2.679675	2.140579
C	-0.70674	1.264108	3.031636
O	0.021219	3.830209	3.053043
C	-1.81118	0.62562	2.470805
C	-2.43028	-0.37793	3.224565
C	-1.92231	-0.68026	4.487471
C	-0.80061	0.018091	4.947981

N	-0.19942	0.974707	4.228881
O	-0.73443	2.757665	0.879998
C	4.310422	0.537167	-0.19273
C	3.932354	-0.78761	-0.47907
C	4.574197	-1.51132	-1.49026
C	5.604637	-0.92923	-2.23723
C	5.990076	0.387088	-1.96327
C	5.34679	1.108182	-0.95157
C	1.735065	1.971429	-0.57674
C	1.996349	3.299836	-0.94885
C	1.680385	3.76012	-2.23039
C	1.095138	2.896227	-3.16395
C	0.827605	1.571549	-2.80284
C	1.14599	1.116543	-1.51864
C	2.294332	-2.03023	6.241632
C	1.746777	-2.65615	5.10442
C	1.081773	-3.87976	5.22414
C	0.946881	-4.49082	6.476947
C	1.49359	-3.87684	7.607987
C	2.167519	-2.65452	7.493403
C	3.6414	0.217758	7.621456
C	2.609382	0.435046	8.557422
C	2.872312	1.051042	9.783458
C	4.16782	1.484278	10.09062
C	5.193042	1.300451	9.15862
C	4.934071	0.673841	7.933236
H	1.697939	0.441438	0.947425
H	4.024329	2.397095	0.760588
H	5.484857	-1.95079	6.954983
H	7.622144	-2.67943	5.947388
H	8.125013	-2.11687	3.555553
H	6.438802	-0.89439	2.196832
H	-2.17017	0.915631	1.483107
H	-3.29951	-0.9089	2.826692
H	-2.37677	-1.45037	5.115099
H	-0.36766	-0.20369	5.927319
H	3.143425	-1.26227	0.105801
H	4.263573	-2.5408	-1.69633
H	6.102653	-1.49771	-3.02904

H	6.792474	0.858046	-2.54012
H	5.654858	2.139488	-0.7488
H	2.438041	3.996347	-0.22862
H	1.888931	4.800249	-2.50021
H	0.845647	3.256122	-4.16701
H	0.365367	0.888265	-3.52231
H	0.930441	0.079323	-1.24339
H	1.876018	-2.1958	4.118723
H	0.666879	-4.35891	4.331818
H	0.421277	-5.44621	6.569884
H	1.40272	-4.35183	8.589849
H	2.597303	-2.19851	8.388288
H	1.583697	0.129817	8.327689
H	2.057468	1.200568	10.49835
H	4.373469	1.970583	11.04893
H	6.207271	1.645918	9.381101
H	5.751217	0.552315	7.218896
N	1.617786	2.285053	1.918701
N	4.26547	0.988252	2.231469
Ag	2.025856	1.607707	4.98746
O	4.680676	4.062349	5.131219
C	3.6482	4.314133	5.8311
O	2.56078	3.687994	5.799891
C	3.778199	5.465659	6.820873
H	4.459155	6.242106	6.437674
H	2.791849	5.899159	7.048275
H	4.206458	5.07386	7.762242
Ag	4.637899	2.512449	3.671679
H	2.195549	3.079742	2.195835

3.1.4. Computational study (3D structure, Steric map)

The computational results for the dinuclear catalyst were analyzed using 3D structural models and steric maps. It was found that the phosphine and pyridinesulfonyl groups of the ligand formed an appropriate asymmetric reaction site by adopting a conformation that covered the metal center (**Figure S3**). Based on these preliminary conformational searches, the 2-pyridinesulfonylated diamine–phosphine ligand designed in this study is expected to create an excellent asymmetric environment, as the reactive substrate can be properly accommodated within the resulting chiral pocket.

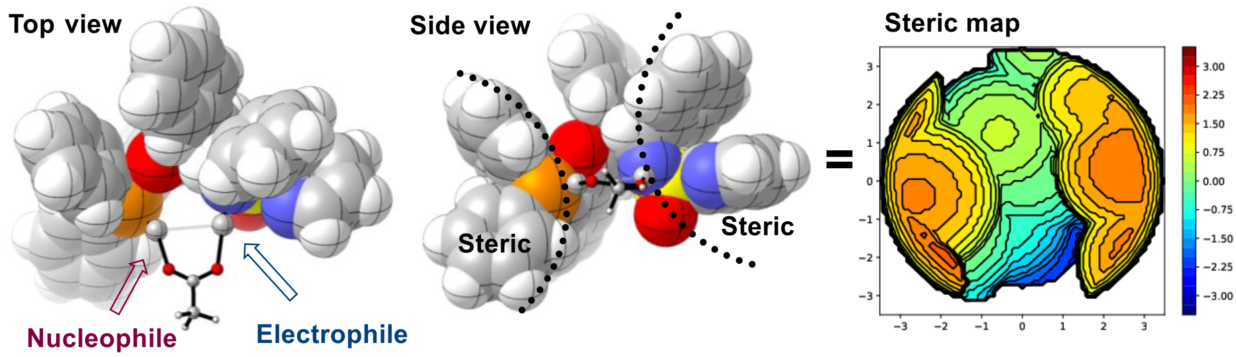

Detailed information of Steric map

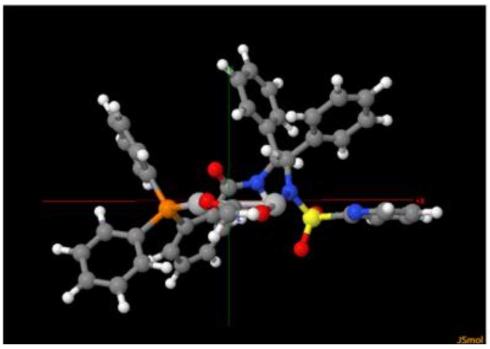

%V Free	%V Buried	% V Tot/V Ex
37.8	62.2	99.9

Quadrant	V f	V b	V t	%V f	%V b
SW	16.8	28.1	44.9	37.4	62.6
NW	16.6	28.2	44.9	37.1	62.9
NE	11.8	33.0	44.9	26.4	73.6
SE	22.5	22.3	44.9	50.3	49.7

Figure S3. Computational study of the dinuclear catalyst.

3.1.5. Energy diagram of the catalyst between 3 and Int-1

ESI-MS analysis of a THF solution of ligand **A** with 2 equivalents of AgOAc detected **Int-1** (**Int-1**−OAc$^+$ calcd. for $C_{38}H_{31}N_3O_3PSAg_2^+$: 853.9921; found 853.9916). Here, the dinuclear complex (**Int-1**) was found to be more stable than the mononuclear complex (**Int-1'** −OAc$^+$).

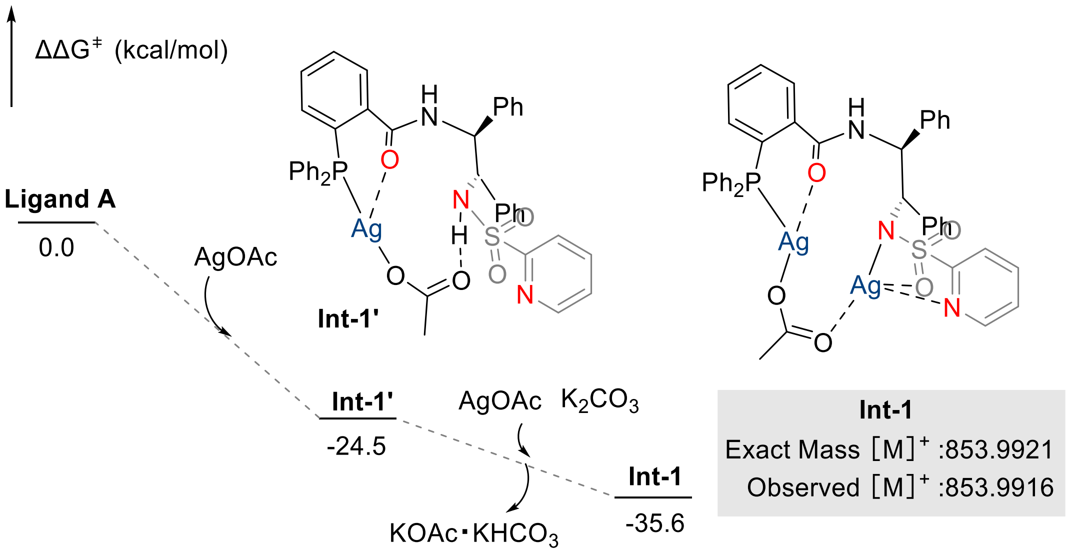

Figure S4. Energy profiles for the formation of dinuclear catalyst.

3.1.6. Calculated Structures

Ligand A

M06-D3/def2-TZVPP/CPCM(THF)//B3LYP/def2-SV(P)/CPCM(THF)

B3LYP/def2-SV(P)/CPCM(THF) free energy: -2595.11945936 (a.u.)

M06-D3/def2-TZVPP/CPCM(THF) single point energy: -2596.14945063 (a.u.)

Thermal correction to Gibbs Free Energy: 0.544338

C	-2.64475	-0.62915	-0.25566
C	-1.20839	-1.16056	-0.56149
C	0.01552	0.50528	0.89195
C	1.17192	1.49504	0.9553
O	-0.74976	0.4037	1.85015
C	2.4521	1.37514	0.34525
C	3.38257	2.4113	0.54768
C	3.08126	3.53674	1.31976
C	1.83073	3.64159	1.9307
C	0.8982	2.61967	1.75381
P	2.90893	-0.08437	-0.72933
S	-3.99182	1.74587	-0.36757
C	-3.09105	3.25206	0.09578
O	-4.82668	2.09215	-1.52292
C	-3.1652	3.71982	1.40746
C	-2.49413	4.91515	1.69026
C	-1.79761	5.55789	0.66462
C	-1.79517	4.9807	-0.61275
N	-2.43782	3.84499	-0.89433
O	-4.58623	1.19376	0.85745
C	-0.9452	-2.53594	0.05218
C	-0.94087	-2.74425	1.44329
C	-0.69387	-4.01602	1.96966
C	-0.45161	-5.10143	1.11896
C	-0.45367	-4.90491	-0.26537
C	-0.69743	-3.63081	-0.7909
C	-3.71574	-1.62323	-0.69874

C	-3.96318	-1.87065	-2.05915
C	-4.94707	-2.78225	-2.45439
C	-5.70171	-3.46269	-1.49155
C	-5.46712	-3.22085	-0.13393
C	-4.48232	-2.30687	0.25635
C	4.5631	0.37664	-1.4153
C	4.57578	1.05266	-2.65002
C	5.78356	1.41198	-3.25579
C	6.99894	1.08248	-2.64425
C	6.99855	0.3975	-1.42398
C	5.7895	0.04851	-0.81148
C	3.30977	-1.39594	0.51338
C	3.36837	-2.72226	0.045
C	3.66995	-3.77458	0.91445
C	3.90109	-3.51842	2.27129
C	3.83374	-2.20576	2.75031
C	3.54273	-1.15045	1.87773
H	-2.72901	-0.4717	0.82502
H	-1.1507	-1.28946	-1.65352
H	4.37425	2.33644	0.09685
H	3.83205	4.3224	1.44894
H	1.5806	4.51165	2.54492
H	-0.07927	2.67704	2.23681
H	-3.72335	3.16986	2.16656
H	-2.51816	5.33546	2.69985
H	-1.26107	6.49309	0.84499
H	-1.25485	5.45776	-1.43887
H	-1.11955	-1.90109	2.11376
H	-0.69224	-4.16016	3.05475
H	-0.26185	-6.09601	1.53458
H	-0.26514	-5.74458	-0.94167
H	-0.69798	-3.48813	-1.87655
H	-3.39203	-1.3432	-2.83071
H	-5.12593	-2.95998	-3.5194
H	-6.47322	-4.17517	-1.7996
H	-6.05615	-3.74234	0.62698
H	-4.30974	-2.11669	1.32003
H	3.62982	1.29765	-3.14479
H	5.77532	1.94118	-4.21377

H	7.94557	1.35424	-3.1215
H	7.9455	0.13352	-0.9427
H	5.80457	-0.4829	0.14398
H	3.17379	-2.93507	-1.01153
H	3.71383	-4.79928	0.53276
H	4.12862	-4.34204	2.95506
H	4.01094	-1.99843	3.81032
H	3.4985	-0.12972	2.26693
N	-2.79358	0.69542	-0.87479
H	-2.66257	0.75424	-1.88406
N	-0.15618	-0.18025	-0.26601
H	0.54144	-0.0536	-0.9973

Int-1’

M06-D3/def2-TZVPP/CPCM(THF)//B3LYP/def2-SV(P)/CPCM(THF)

B3LYP/def2-SV(P)/CPCM(THF) free energy: -2970.51989634 (a.u.)

M06-D3/def2-TZVPP/CPCM(THF) single point energy: -2971.73008616 (a.u.)

Thermal correction to Gibbs Free Energy: 0.590262

C	2.24597	0.83796	0.25467
C	1.85014	1.93594	-0.77235
C	-0.61341	1.44954	-0.99879
C	-1.71949	1.25406	-2.00513
O	-0.85513	1.47002	0.20888
C	-2.9055	0.55438	-1.66145
C	-3.91367	0.42776	-2.63213
C	-3.78014	0.99307	-3.90426
C	-2.62381	1.70109	-4.23133
C	-1.60594	1.82594	-3.28381
P	-3.14756	-0.24455	-0.00141
S	2.4381	-1.27499	-1.56351
C	4.09215	-1.96765	-1.25109
O	1.54992	-2.42794	-1.76466
C	5.15609	-1.6319	-2.08854
C	6.38694	-2.24459	-1.82743
C	6.48305	-3.13928	-0.75962
C	5.34092	-3.39066	0.01253

N	4.1614	-2.81523	-0.23261
O	2.60113	-0.27385	-2.63623
C	1.80562	3.34811	-0.18339
C	1.28124	3.63833	1.08628
C	1.24119	4.95543	1.55816
C	1.71993	6.00599	0.76849
C	2.24109	5.73048	-0.50057
C	2.28263	4.41326	-0.96699
C	3.68321	0.95007	0.78109
C	4.69581	1.69594	0.15936
C	5.99028	1.73492	0.69534
C	6.29284	1.02847	1.86189
C	5.29138	0.27606	2.48914
C	4.00209	0.2385	1.95377
C	-3.6964	1.08727	1.14285
C	-4.18067	2.32353	0.68717
C	-4.61109	3.29185	1.60088
C	-4.56588	3.03369	2.97533
C	-4.08498	1.80249	3.43531
C	-3.64888	0.83555	2.52417
C	-4.67399	-1.26893	-0.23227
C	-4.52625	-2.58067	-0.72059
C	-5.64178	-3.40162	-0.90752
C	-6.92136	-2.92781	-0.59432
C	-7.07821	-1.62929	-0.09893
C	-5.96274	-0.80269	0.07919
H	1.58016	0.93865	1.12199
H	2.6157	1.94052	-1.55621
H	-4.82858	-0.11852	-2.39711
H	-4.58916	0.88275	-4.6322
H	-2.51196	2.16247	-5.2166
H	-0.71492	2.40537	-3.53659
H	5.01998	-0.9234	-2.90674
H	7.25719	-2.02242	-2.45188
H	7.42649	-3.63782	-0.5221
H	5.38191	-4.08432	0.86025
H	0.88453	2.83796	1.71307
H	0.82927	5.15982	2.55142
H	1.68831	7.03475	1.1407

H	2.62152	6.5423	-1.1284
H	2.69595	4.20739	-1.96033
H	4.49453	2.26741	-0.74926
H	6.76311	2.32644	0.19447
H	7.30311	1.06258	2.28159
H	5.51622	-0.28398	3.40245
H	3.22812	-0.37022	2.43268
H	-4.21967	2.54013	-0.38382
H	-4.98291	4.25364	1.23443
H	-4.90148	3.79319	3.68807
H	-4.04148	1.59438	4.50866
H	-3.26332	-0.12019	2.89453
H	-3.52877	-2.96816	-0.95517
H	-5.50913	-4.41817	-1.28991
H	-7.79457	-3.57294	-0.73128
H	-8.07481	-1.25358	0.15235
H	-6.10328	0.21021	0.46494
N	0.62671	1.62297	-1.51962
H	0.78701	1.30544	-2.4697
Ag	-1.42912	-1.70993	0.84027
O	-0.32492	-3.36337	1.70449
C	0.79025	-2.98835	2.19146
O	1.21557	-1.81007	2.14594
C	1.66576	-4.05856	2.82976
H	2.52532	-4.24365	2.15968
H	2.07087	-3.69446	3.78903
H	1.11854	-5.00109	2.984
N	1.90051	-0.51429	-0.21321
H	1.60968	-1.15634	0.55047

Int-1

M06-D3/def2-TZVPP/CPCM(THF)//B3LYP/def2-SV(P)/CPCM(THF)

B3LYP/def2-SV(P)/CPCM(THF) free energy: -3116.96936552 (a.u.)

M06-D3/def2-TZVPP/CPCM(THF) single point energy: -3118.20244440 (a.u.)

Thermal correction to Gibbs Free Energy: 0.575642

C	-2.15513	0.96382	-0.29197
C	-1.76338	2.23233	0.53258

C	0.62535	1.6453	1.05479
C	1.61585	1.40217	2.1634
O	0.98015	1.54279	-0.12298
C	2.82138	0.69972	1.90068
C	3.71651	0.48654	2.96347
C	3.45483	0.96901	4.24922
C	2.28418	1.68596	4.49712
C	1.3782	1.89757	3.45698
P	3.25063	0.0317	0.21856
S	-2.69905	-0.60939	1.86716
C	-4.40076	-1.24606	1.62572
O	-1.98244	-1.74843	2.48527
C	-5.43654	-0.77444	2.4368
C	-6.70243	-1.34166	2.26207
C	-6.86971	-2.33749	1.29637
C	-5.75686	-2.73088	0.54368
N	-4.54343	-2.19679	0.70933
O	-2.86988	0.60981	2.70948
C	-1.59088	3.49796	-0.30885
C	-2.12489	4.70124	0.18461
C	-1.99413	5.89951	-0.52451
C	-1.32567	5.91409	-1.75321
C	-0.7924	4.72352	-2.25893
C	-0.92206	3.52764	-1.54388
C	-3.51079	1.12375	-0.98182
C	-4.6608	1.56202	-0.30264
C	-5.89088	1.66037	-0.9623
C	-5.99593	1.32271	-2.3163
C	-4.85956	0.88566	-3.00546
C	-3.63189	0.79038	-2.34146
C	3.84432	1.46359	-0.77518
C	4.20294	2.68836	-0.19049
C	4.67284	3.73933	-0.98581
C	4.79303	3.57673	-2.37023
C	4.439	2.35734	-2.95866
C	3.96404	1.30754	-2.16611
C	4.80744	-0.92987	0.53061
C	4.68977	-2.27892	0.91418
C	5.82691	-3.05584	1.15312

C	7.10166	-2.49864	0.99709
C	7.23073	-1.1616	0.60734
C	6.09217	-0.38029	0.37816
H	-1.40898	0.86423	-1.08916
H	-2.58139	2.43684	1.23058
H	4.64446	-0.06065	2.79195
H	4.17767	0.79044	5.05056
H	2.07516	2.08677	5.49296
H	0.47935	2.4854	3.65557
H	-5.24813	0.0092	3.17192
H	-7.54671	-1.00745	2.87264
H	-7.84284	-2.80534	1.12527
H	-5.84896	-3.50969	-0.22242
H	-2.6559	4.69937	1.14252
H	-2.4207	6.82187	-0.11775
H	-1.22282	6.84776	-2.31517
H	-0.2673	4.72324	-3.21947
H	-0.4826	2.61542	-1.94951
H	-4.60706	1.82486	0.75715
H	-6.773	2.00435	-0.41293
H	-6.9582	1.40205	-2.83174
H	-4.92668	0.62121	-4.06555
H	-2.74656	0.45153	-2.89033
H	4.1133	2.83167	0.88968
H	4.94551	4.6905	-0.51841
H	5.15899	4.40059	-2.99052
H	4.52567	2.22246	-4.04106
H	3.68033	0.3618	-2.63984
H	3.69833	-2.73095	1.02837
H	5.71604	-4.10219	1.45325
H	7.99306	-3.10802	1.17457
H	8.22378	-0.71988	0.47978
H	6.21187	0.66366	0.07783
N	-2.00112	-0.30261	0.45283
N	-0.6284	1.98921	1.43185
H	-0.92039	1.74757	2.37459
Ag	-1.30463	-2.05987	-0.52348
Ag	1.76443	-1.49919	-0.94964
O	1.27893	-3.14392	-2.28582

C	0.29395	-3.933	-2.29794
O	-0.77662	-3.79376	-1.64369
C	0.42418	-5.1744	-3.16837
H	1.1581	-5.86036	-2.70783
H	-0.53931	-5.69564	-3.26998
H	0.81296	-4.89892	-4.16295

3.2 Energy diagram for the reaction from Int-1 to Int-5

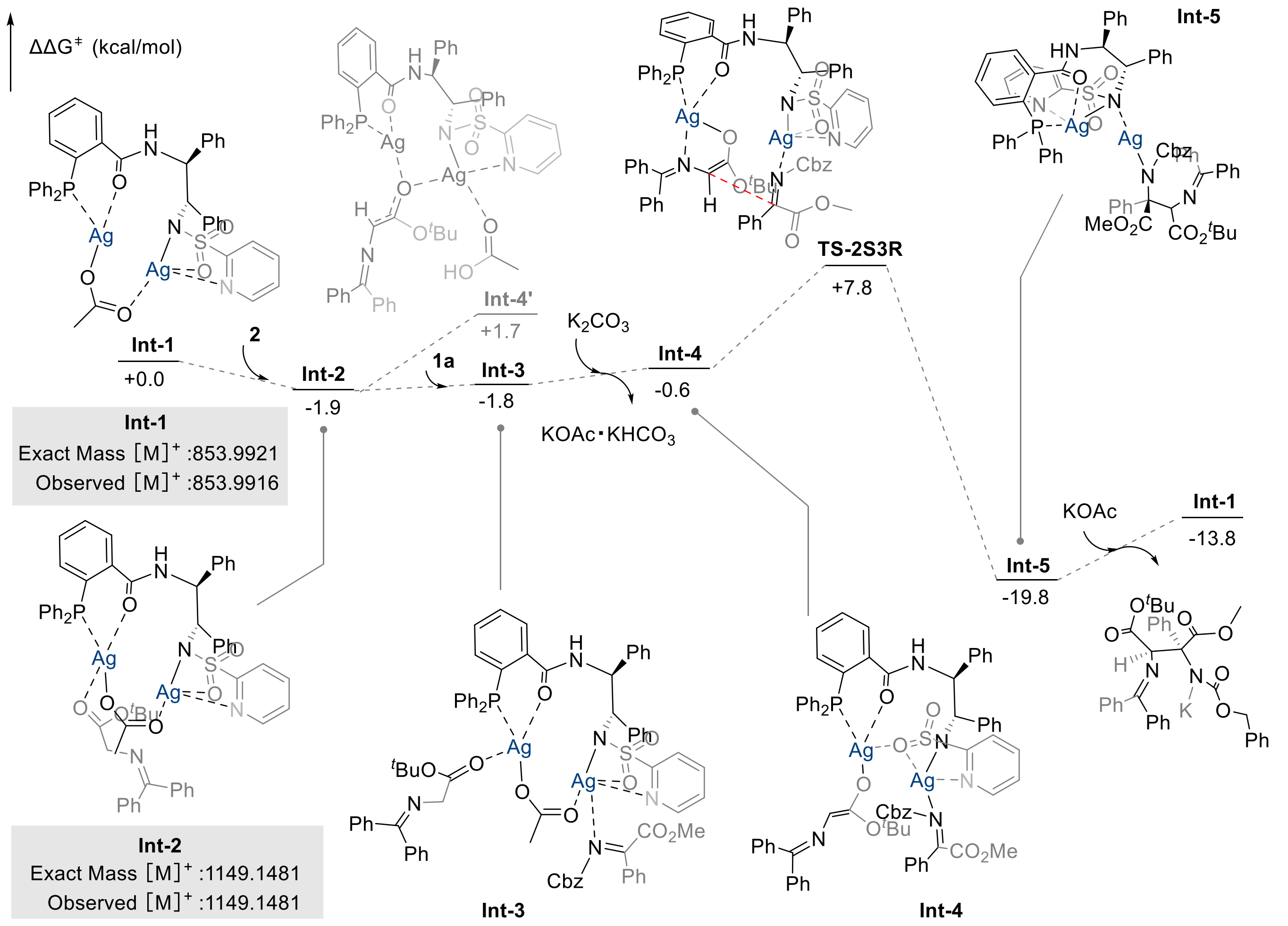

Figure S5. Energy profiles for the addition of glycinate Schiff base **2** to acyclic ketimine **1a** using ligand **A**.

3.2.1. Intermidiate-2 (Int-2)

The reaction path between **Int-1** and **Int-2** was explored by the MC-AFIR method implemented in the GRRM program. In the initial search, electronic structure calculations were carried out at the GFN2-xTB method using the Orca program. The artificial force is applied between the Ag(79) atom and N(103), O(88) atoms of the Schiff base **2** moiety. The collision energy parameter (γ) of the AFIR method was set to 200.0 kJ/mol. In this case, the last integer in each Cartesian coordinate is for the random distribution of each molecule; the catalyst (ligand + 2Ag + acetate anion) was defined as part #1, and the Schiff base is part #2. All obtained 10 equilibrium (EQ) structures were re-optimized at the B3LYP/def2-SV(P)/CPCM(THF). After that, single-point energy calculations for the optimized geometry were performed using M06-D3 functional with def2-TZVPP basis set for all the atoms in CPCM solvation model (THF).

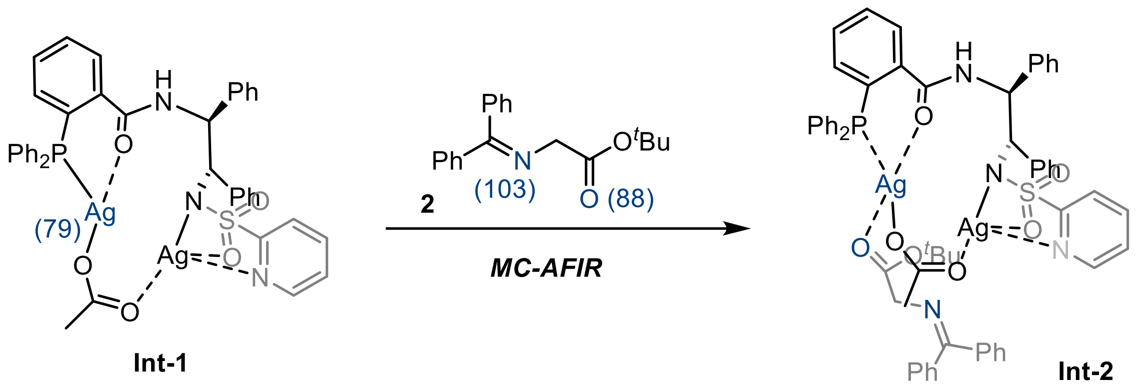

Scheme S2. MC-AFIR Search for **Int-2**.

Calculated Structures

Int-2

M06-D3/def2-TZVPP/CPCM(THF)//B3LYP/def2-SV(P)/CPCM(THF)

B3LYP/def2-SV(P)/CPCM(THF) free energy: -4058.13404679 (a.u.)

M06-D3/def2-TZVPP/CPCM(THF) single point energy: -4059.78405532 (a.u.)

Thermal correction to Gibbs Free Energy: 0.904772

C	-1.64845	-2.78446	-0.1779
C	-2.63287	-2.82548	1.02946
C	-3.05252	-0.37155	1.33015
C	-2.94151	0.78761	2.28469
O	-3.53171	-0.20423	0.20493
C	-2.94884	2.11956	1.79529
C	-2.85261	3.17348	2.72027
C	-2.77697	2.93399	4.09578
C	-2.79584	1.62381	4.57547
C	-2.87721	0.56408	3.67056
P	-3.06094	2.51913	-0.01699
S	0.59719	-2.51162	1.34607
C	1.54012	-4.03978	0.97944
O	1.63812	-1.46015	1.45609
C	1.60987	-5.05528	1.93672
C	2.39305	-6.17354	1.63382
C	3.05622	-6.2213	0.40517
C	2.91293	-5.14056	-0.47393
N	2.1709	-4.06724	-0.18889

O	-0.15109	-2.83129	2.59688
C	-4.05412	-3.27817	0.68873
C	-4.68758	-3.01831	-0.53635
C	-5.99702	-3.45268	-0.77179
C	-6.70087	-4.14997	0.21575
C	-6.08225	-4.41474	1.44254
C	-4.77158	-3.98448	1.66995
C	-1.48254	-4.14519	-0.85839
C	-1.39219	-5.35491	-0.15021
C	-1.19596	-6.56797	-0.82104
C	-1.08722	-6.59524	-2.21528
C	-1.17915	-5.39827	-2.93478
C	-1.37698	-4.19008	-2.25954
C	-4.84411	2.38877	-0.45816
C	-5.86008	2.3749	0.51064
C	-7.20314	2.30292	0.12457
C	-7.54516	2.249	-1.23097
C	-6.5372	2.26429	-2.2017
C	-5.1942	2.32977	-1.81749
C	-2.74173	4.34492	-0.07236
C	-1.40238	4.77718	-0.12203
C	-1.09989	6.141	-0.18214
C	-2.12859	7.09043	-0.20913
C	-3.4615	6.66898	-0.16945
C	-3.76798	5.30471	-0.09864
H	-2.0968	-2.11485	-0.92197
H	-2.24192	-3.5481	1.75412
H	-2.8444	4.2063	2.36847
H	-2.71387	3.77828	4.78848
H	-2.75521	1.42243	5.64968
H	-2.91997	-0.45745	4.05497
H	1.06646	-4.96405	2.87793
H	2.48109	-6.99663	2.3494
H	3.67596	-7.07795	0.12724
H	3.41999	-5.14201	-1.44592
H	-4.16548	-2.46693	-1.31996
H	-6.4705	-3.24053	-1.73565
H	-7.72513	-4.48784	0.02914
H	-6.61825	-4.96375	2.22323

H	-4.29293	-4.20199	2.63086
H	-1.47584	-5.36797	0.93974
H	-1.13045	-7.49797	-0.24745
H	-0.93613	-7.54448	-2.73875
H	-1.10179	-5.40519	-4.02664
H	-1.45192	-3.25837	-2.83099
H	-5.60921	2.4165	1.57395
H	-7.98534	2.28884	0.88977
H	-8.59603	2.1915	-1.53069
H	-6.79498	2.21793	-3.26411
H	-4.41425	2.32946	-2.58642
H	-0.58425	4.04881	-0.10731
H	-0.054	6.46122	-0.21665
H	-1.891	8.15723	-0.26547
H	-4.27163	7.40438	-0.19352
H	-4.81495	4.99313	-0.06493
N	-0.3614	-2.1136	0.11912
N	-2.62299	-1.57017	1.78956
H	-1.96466	-1.58262	2.5648
Ag	0.54049	-0.84988	-1.3468
Ag	-1.54467	1.52332	-1.6362
O	1.37079	0.21228	-3.00763
C	0.69884	0.97832	-3.75609
O	-0.46643	1.40216	-3.52328
C	1.36401	1.45923	-5.03678
H	0.63459	1.48101	-5.86274
H	1.72115	2.49409	-4.88254
H	2.22356	0.82594	-5.30314
C	2.55639	2.37706	0.41431
O	1.44257	2.82213	0.22882
O	3.14615	2.22416	1.59527
C	2.5084	2.54661	2.88864
C	2.26873	4.05734	2.97183
H	1.52704	4.3873	2.22811
H	1.89325	4.31648	3.97731
H	3.21091	4.60858	2.8048
C	1.22034	1.73601	3.06379
H	1.4041	0.66575	2.86675
H	0.86265	1.84577	4.10271

H	0.42714	2.0851	2.3855
C	3.56698	2.10497	3.90205
H	3.22136	2.31585	4.9284
H	3.7603	1.02212	3.81307
H	4.51564	2.64391	3.73401
N	4.23806	0.78044	-0.41632
C	5.51353	0.76542	-0.30448
C	6.17487	-0.5136	0.10394
C	7.55729	-0.7103	-0.07006
C	5.41053	-1.55395	0.67126
C	8.16046	-1.91696	0.30073
H	8.16894	0.08267	-0.50756
C	6.01567	-2.75344	1.04792
H	4.33826	-1.40535	0.82058
C	7.39248	-2.94165	0.86192
H	9.23578	-2.05426	0.15067
H	5.41003	-3.54876	1.49354
H	7.86421	-3.88392	1.15803
C	6.39901	1.96185	-0.54241
C	6.9612	2.651	0.54536
C	6.66355	2.41335	-1.84601
C	7.76666	3.77448	0.3333
H	6.76244	2.30673	1.56501
C	7.47686	3.53233	-2.05785
H	6.23315	1.88222	-2.70063
C	8.02847	4.21624	-0.96901
H	8.19349	4.3059	1.18956
H	7.67862	3.87092	-3.07883
H	8.66249	5.0927	-1.1348
C	3.49505	1.97679	-0.72683
H	2.85648	1.78407	-1.60378
H	4.12086	2.86728	-0.94329

3.2.2. Intermidiate-3 (Int-3)

The reaction path from **Int-2** to **Int-3** was explored by the MC-AFIR method implemented in the GRRM program. In the initial search, electronic structure calculations were carried out at the GFN2-xTB method in the Orca program. The artificial force is applied between the Ag(78) atom and N(145), O(147), O(130)

atoms of the ketimine **1a** moiety. The collision energy parameter γ of the AFIR method was set as γ = 400.0 kJ/mol. In this case, the last integer in each Cartesian coordinate represents a random distribution of each molecule; the catalyst (ligand + 2Ag + acetate anion) was defined as part #1, the Schiff base is part #2, and ketiminoester is part #3. All obtained 10 equilibrium (EQ) structures were re-optimized at the B3LYP/def2-SV(P)/CPCM(THF). Subsequently, single-point energy calculations for the optimized geometry were performed using M06-D3 functional with def2-TZVPP basis set for all atoms in CPCM solvation model (THF).

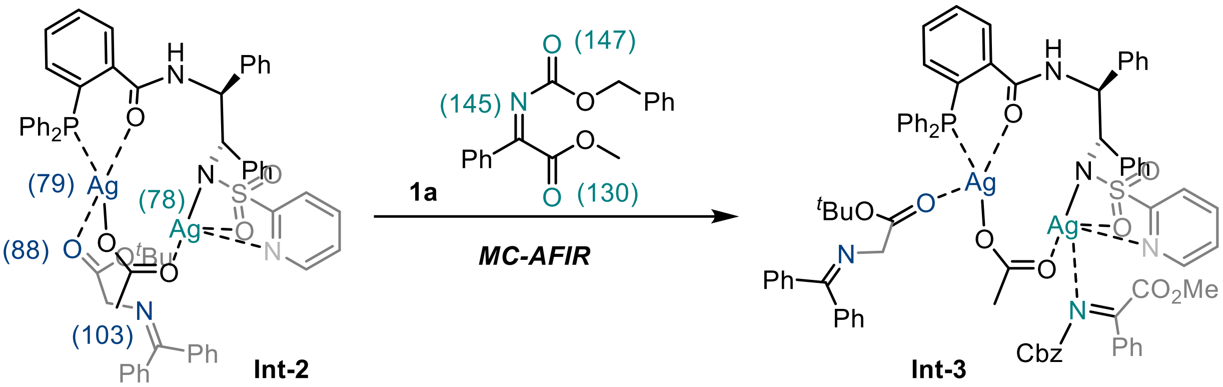

Scheme S3. MC-AFIR search of **Int-3**.

Calculated Structures

Int-3

M06-D3/def2-TZVPP/CPCM(THF)//B3LYP/def2-SV(P)/CPCM(THF)

B3LYP/def2-SV(P)/CPCM(THF) free energy: -5069.87217338 (a.u.)

M06-D3/def2-TZVPP/CPCM(THF) single point energy: -5072.02077269 (a.u.)

Thermal correction to Gibbs Free Energy: 1.165897

C	0.29519	3.44922	0.90535
C	0.11669	4.54437	-0.18943
C	-0.41806	3.04053	-2.13563
C	-0.019	2.6707	-3.54016
O	-1.38067	2.48391	-1.60078
C	-0.39177	1.4144	-4.08335
C	-0.02404	1.12269	-5.40805
C	0.67533	2.04459	-6.19316
C	1.02387	3.28689	-5.6619
C	0.67459	3.59056	-4.34485

P	-1.31394	0.13247	-3.10048
S	2.94396	3.03293	0.35109
C	3.81152	3.53261	1.8872
O	3.70902	1.85369	-0.11985
C	4.53432	4.72815	1.91792
C	5.22759	5.03277	3.0935
C	5.16356	4.1404	4.16645
C	4.40853	2.97058	4.01767
N	3.7477	2.67136	2.89617
O	3.00713	4.24363	-0.52109
C	-1.17127	5.35955	-0.07077
C	-1.09388	6.75299	-0.23984
C	-2.23427	7.55892	-0.1631
C	-3.48194	6.97988	0.09095
C	-3.57339	5.59454	0.26462
C	-2.43041	4.79126	0.18375
C	0.24515	4.01809	2.32368
C	0.95671	5.16505	2.71329
C	0.91338	5.62317	4.035
C	0.1543	4.94251	4.99356
C	-0.56449	3.80221	4.6179
C	-0.51958	3.34874	3.29494
C	-3.09024	0.60911	-3.17433
C	-3.59376	1.47293	-4.16048
C	-4.9564	1.78898	-4.19448
C	-5.83027	1.24295	-3.24783
C	-5.33465	0.3802	-2.26367
C	-3.97172	0.06732	-2.22402
C	-1.22233	-1.37117	-4.18449
C	-0.03746	-2.13305	-4.15332
C	0.08031	-3.29418	-4.92167
C	-0.98818	-3.72131	-5.72032
C	-2.1704	-2.97589	-5.75134
C	-2.2873	-1.80539	-4.99098
H	-0.56364	2.77372	0.81146
H	0.94248	5.25587	-0.08504
H	-0.29048	0.15983	-5.8467
H	0.936	1.78815	-7.2242
H	1.55668	4.0239	-6.26951

H	0.92223	4.5766	-3.94515
H	4.54545	5.38804	1.04954
H	5.80885	5.95681	3.16846
H	5.68819	4.34103	5.10437
H	4.3363	2.24604	4.83733
H	-0.12068	7.21679	-0.43313
H	-2.14559	8.64164	-0.2974
H	-4.37833	7.60471	0.15548
H	-4.5444	5.13093	0.46482
H	-2.52611	3.71094	0.30338
H	1.55641	5.71801	1.98499
H	1.47535	6.51968	4.31565
H	0.11975	5.30225	6.02674
H	-1.16867	3.26467	5.35582
H	-1.09568	2.46239	3.00644
H	-2.92358	1.90783	-4.90702
H	-5.33508	2.46662	-4.96586
H	-6.89544	1.49231	-3.27491
H	-6.00617	-0.05155	-1.51556
H	-3.60352	-0.59897	-1.43651
H	0.80516	-1.81151	-3.53119
H	1.00861	-3.87277	-4.88977
H	-0.89901	-4.63604	-6.3144
H	-3.01089	-3.30343	-6.37103
H	-3.21739	-1.23317	-5.0302
N	1.44577	2.54698	0.66786
N	0.31727	4.01151	-1.54365
H	1.25465	4.17839	-1.89934
Ag	1.33977	0.51317	1.31276
Ag	-0.54117	-0.65415	-0.92764
O	1.1498	-1.47141	2.08962
C	0.38674	-2.3536	1.60422
O	-0.33385	-2.22575	0.57578
C	0.36084	-3.70581	2.2995
H	-0.63068	-4.17625	2.20503
H	1.10118	-4.3567	1.80019
H	0.64141	-3.61029	3.35984
C	-3.8286	-0.19068	2.14181
O	-2.96455	0.66066	2.16241

O	-5.11687	0.00358	2.42542
C	-5.69083	1.31505	2.78959
C	-5.06645	1.80998	4.09823
H	-3.99838	2.04416	3.97332
H	-5.58856	2.72424	4.43064
H	-5.17459	1.04744	4.88948
C	-5.49519	2.30371	1.6365
H	-5.8946	1.88463	0.69644
H	-6.04259	3.23669	1.85692
H	-4.43203	2.54726	1.49133
C	-7.17499	0.99553	2.9824
H	-7.7282	1.91262	3.2473
H	-7.60691	0.5818	2.05495
H	-7.31504	0.2583	3.79198
N	-4.519	-2.23623	0.93322
C	-5.24181	-3.24807	1.24575
C	-6.1607	-3.80789	0.20311
C	-7.257	-4.61844	0.55149
C	-5.9506	-3.51107	-1.15808
C	-8.12396	-5.10995	-0.43048
H	-7.44395	-4.85967	1.60073
C	-6.80967	-4.00884	-2.13817
H	-5.09721	-2.88793	-1.43356
C	-7.90252	-4.80932	-1.77788
H	-8.976	-5.73164	-0.13873
H	-6.62564	-3.77463	-3.19135
H	-8.57639	-5.1994	-2.54704
C	-5.22667	-3.93281	2.58722
C	-5.67162	-3.2684	3.74296
C	-4.76012	-5.25453	2.69877
C	-5.65185	-3.91408	4.98418
H	-6.03445	-2.23977	3.6654
C	-4.72764	-5.89376	3.94183
H	-4.41708	-5.78542	1.80545
C	-5.17715	-5.22611	5.08728
H	-6.0073	-3.38713	5.87505
H	-4.35353	-6.91959	4.01511
H	-5.15836	-5.72905	6.05905
C	-3.54797	-1.66128	1.83463

H	-2.55677	-1.69738	1.353
H	-3.46373	-2.17933	2.81179
O	2.73927	-0.29224	-2.6492
O	4.88561	0.39482	-2.6898
C	5.66431	-2.2759	-1.9046
C	5.91312	-2.47578	-3.27669
C	6.58632	-2.77187	-0.96356
C	7.04416	-3.17829	-3.69578
H	5.21096	-2.09499	-4.02372
C	7.72609	-3.45833	-1.38759
H	6.42874	-2.59682	0.10246
C	7.95444	-3.6691	-2.75224
H	7.21802	-3.33921	-4.76368
H	8.44072	-3.8275	-0.64635
H	8.84615	-4.21094	-3.08159
C	3.90106	-0.42635	-2.34625
C	4.42662	-1.57198	-1.47943
N	3.71627	-1.80074	-0.44736
C	3.84357	-2.80079	0.50372
O	3.33571	-3.89586	0.3943
O	4.47334	-2.34276	1.59439
C	4.4957	-3.1886	2.77412
H	4.52677	-2.47345	3.6081
H	3.55575	-3.75609	2.81441
C	5.69842	-4.10151	2.80053
C	6.92518	-3.63679	3.30302
C	5.61844	-5.41667	2.31375
C	8.05107	-4.46647	3.31617
H	6.99739	-2.61608	3.69309
C	6.74201	-6.24955	2.32924
H	4.66919	-5.78401	1.91406
C	7.96091	-5.77584	2.82881
H	8.99968	-4.09223	3.71349
H	6.66544	-7.27346	1.95035
H	8.83965	-6.42823	2.84237
C	4.53185	1.60752	-3.37325
H	4.03436	2.28503	-2.66174
H	5.47999	2.04167	-3.72
H	3.86855	1.39244	-4.22552

3.2.2. Intermidiate-4’ (Int-4’)

The reaction path from **Int-2** to **Int-4’** was explored by the MC-AFIR method implemented in the GRRM program. In the initial search, electronic structure calculations were carried out by the GFN2-xTB method using the Orca program. The artificial force is applied between the H(128) atom of the Schiff base moiety and O(82) atoms of the acetate moiety, the Ag(78,79) atoms and O(88)･N(103) of the Schiff base moiety. The collision energy parameter (γ) of the AFIR method was set to 400.0 kJ/mol. The last integer in each Cartesian coordinate is for random distribution of each molecule; in this case, the catalyst (ligand + 2Ag) was defined as part #1, the acetate anion is part #2, and the Schiff base (except H(80)) is part #3. All obtained 10 equilibrium (EQ) structures were re-optimized at the B3LYP/def2-SV(P)/CPCM(THF). Subsequently, single-point energy calculations for the optimized geometry were performed using M06-D3 functional with def2-TZVPP basis set for all atoms in CPCM solvation model (THF).

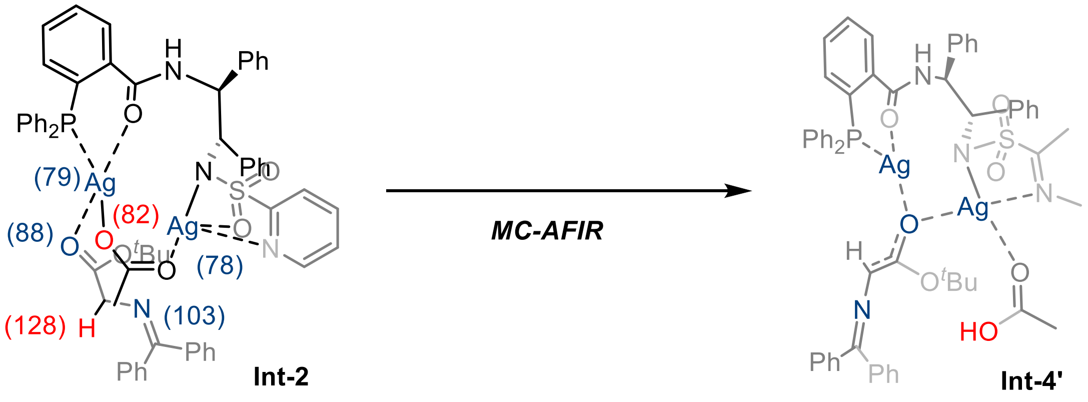

Scheme S4. MC-AFIR search of **Int-4’**.

Calculated Structures

Int-4’

M06-D3/def2-TZVPP/CPCM(THF)//B3LYP/def2-SV(P)/CPCM(THF)

B3LYP/def2-SV(P)/CPCM(THF) free energy: -4058.10283803 (a.u.)

M06-D3/def2-TZVPP/CPCM(THF) single point energy: -4059.77530297 (a.u.)

Thermal correction to Gibbs Free Energy: 0.901709

C	2.59293	-0.2985	0.97785
C	3.30935	0.79109	1.84592
C	1.46885	2.5325	1.88748

C	1.22119	4.01043	1.69498
O	0.55345	1.77448	2.21218
C	0.11911	4.48005	0.93947
C	-0.08398	5.86641	0.83941
C	0.76394	6.77357	1.48364
C	1.84672	6.30524	2.23014
C	2.0738	4.9297	2.32626
P	-0.97975	3.30472	0.01167
S	3.26945	0.73898	-1.43974
C	4.08022	-0.61119	-2.38311
O	2.47185	1.44129	-2.48285
C	5.41541	-0.47377	-2.76418
C	5.97274	-1.48087	-3.55727
C	5.17971	-2.57005	-3.92741
C	3.8527	-2.61125	-3.49175
N	3.31586	-1.64378	-2.73565
O	4.39774	1.49963	-0.84131
C	3.44376	0.38796	3.31486
C	2.34278	0.01875	4.10813
C	2.51738	-0.33044	5.4517
C	3.79268	-0.31996	6.02823
C	4.89569	0.04475	5.24949
C	4.71833	0.39415	3.90645
C	3.36132	-1.62118	1.01379
C	4.73131	-1.70955	0.70997
C	5.39057	-2.9435	0.71765
C	4.69356	-4.1154	1.03392
C	3.33174	-4.04162	1.3445
C	2.67614	-2.80509	1.33473
C	-2.39945	2.96319	1.13247
C	-2.64334	3.70134	2.30193
C	-3.74059	3.39464	3.11476
C	-4.60623	2.35254	2.7661
C	-4.36841	1.61154	1.60218
C	-3.26775	1.90987	0.79303
C	-1.69256	4.3572	-1.32746
C	-0.92331	4.5471	-2.4906
C	-1.40696	5.33041	-3.54184
C	-2.67406	5.92013	-3.4523

C	-3.45073	5.72599	-2.30557
C	-2.96307	4.95119	-1.24609
H	1.61937	-0.48944	1.44659
H	4.32528	0.90113	1.45374
H	-0.9163	6.2516	0.24575
H	0.57445	7.84746	1.39623
H	2.51537	7.00598	2.7385
H	2.91607	4.55975	2.91754
H	5.99023	0.3953	-2.44152
H	7.01597	-1.41488	-3.87965
H	5.57724	-3.38002	-4.5437
H	3.19673	-3.44676	-3.75487
H	1.33961	0.02192	3.67826
H	1.64702	-0.6127	6.05289
H	3.92565	-0.59537	7.07919
H	5.89931	0.05602	5.68623
H	5.58933	0.67457	3.30483
H	5.298	-0.80959	0.45684
H	6.45747	-2.98787	0.47704
H	5.21103	-5.07975	1.04254
H	2.7748	-4.94837	1.60084
H	1.612	-2.75927	1.58951
H	-1.97536	4.51784	2.58788
H	-3.91747	3.97486	4.02562
H	-5.46328	2.11373	3.40321
H	-5.03449	0.7891	1.32523
H	-3.08732	1.30916	-0.104
H	0.06137	4.07534	-2.57805
H	-0.79616	5.47203	-4.43859
H	-3.05723	6.52603	-4.27908
H	-4.44338	6.18052	-2.23059
H	-3.5799	4.81102	-0.35463
N	2.21466	0.12837	-0.39015
N	2.74919	2.14192	1.68584
H	3.37412	2.81515	1.25514
Ag	1.14383	-1.73855	-1.76999
Ag	0.22913	1.33055	-0.78507
O	0.37106	-4.01642	-1.83621
C	-0.64007	-4.70316	-1.91313

O	-1.85487	-4.26863	-1.62944
C	-0.62028	-6.15114	-2.33337
H	-1.35866	-6.32202	-3.13495
H	0.38515	-6.43599	-2.67433
H	-0.91357	-6.78482	-1.47745
O	-0.85406	-0.51865	-2.07262
C	-1.98864	-0.91314	-1.66023
O	-3.1017	-0.66959	-2.38323
C	-3.09582	-0.26203	-3.78741
N	-3.42408	-1.97238	-0.01332
C	-3.68956	-2.51555	1.13578
C	-2.61797	-2.82751	2.14731
C	-2.30782	-1.92063	3.17612
C	-1.87935	-4.02104	2.06128
C	-1.29046	-2.2001	4.09624
H	-2.87139	-0.98544	3.25575
C	-0.8627	-4.30412	2.98077
H	-2.10614	-4.73415	1.26246
C	-0.5653	-3.39369	4.0018
H	-1.06428	-1.48253	4.89162
H	-0.30152	-5.24086	2.90067
H	0.23015	-3.61263	4.72095
C	-5.09959	-2.84189	1.46096
C	-5.46263	-3.45882	2.67727
C	-6.13461	-2.54087	0.54463
C	-6.79858	-3.75934	2.96609
H	-4.68947	-3.70881	3.40854
C	-7.46444	-2.84076	0.83404
H	-5.861	-2.06663	-0.40071
C	-7.80889	-3.45296	2.04924
H	-7.04916	-4.23852	3.91837
H	-8.24493	-2.59584	0.10561
H	-8.85377	-3.68765	2.27546
C	-2.55915	1.16658	-3.93781
H	-3.1141	1.85634	-3.27803
H	-1.49112	1.22069	-3.68063
H	-2.69281	1.50855	-4.97979
C	-2.30455	-1.26799	-4.63381
H	-2.66027	-2.29522	-4.43773

H	-2.45287	-1.05184	-5.70658
H	-1.22729	-1.21925	-4.41262
C	-4.58261	-0.31155	-4.15861
H	-4.98122	-1.33267	-4.0284
H	-5.16389	0.3714	-3.51493
H	-4.72733	-0.00914	-5.21001
C	-2.17853	-1.62613	-0.44976
H	-1.86216	-3.30833	-1.29651
H	-1.29856	-1.68259	0.21023

3.2.3. Intermidiate-4 (Int-4)

The reaction path from **Int-3** to **Int-4** was explored by the MC-AFIR method implemented in the GRRM program. In the initial search, electronic structure calculations were carried out by the GFN2-xTB method using the Orca program. The artificial force is applied between the H(128) atom of the Schiff base moiety and O(82) atoms of the acetate moiety, the Ag(79) atom and O(88)•N(103) of the Schiff base moiety, the Ag(78) atoms and the N(145)•O(147) of the ketimine moiety. The collision energy parameter (γ) of the AFIR method was set to 600.0 kJ/mol. The last integer in each Cartesian coordinate is for random distribution of each molecule; in this case, the catalyst (ligand + 2Ag) was defined as part #1, the acetate anion is part #2, the Schiff base (except H(128)) is part #3, and the ketiminoester is part #4. All obtained 10 equilibrium (EQ) structures were re-optimized at the B3LYP/def2-SV(P)/CPCM(THF). Subsequently, single-point energy calculations for the optimized geometry were performed using M06-D3 functional with def2-TZVPP basis set for all atoms in CPCM solvation model (THF).

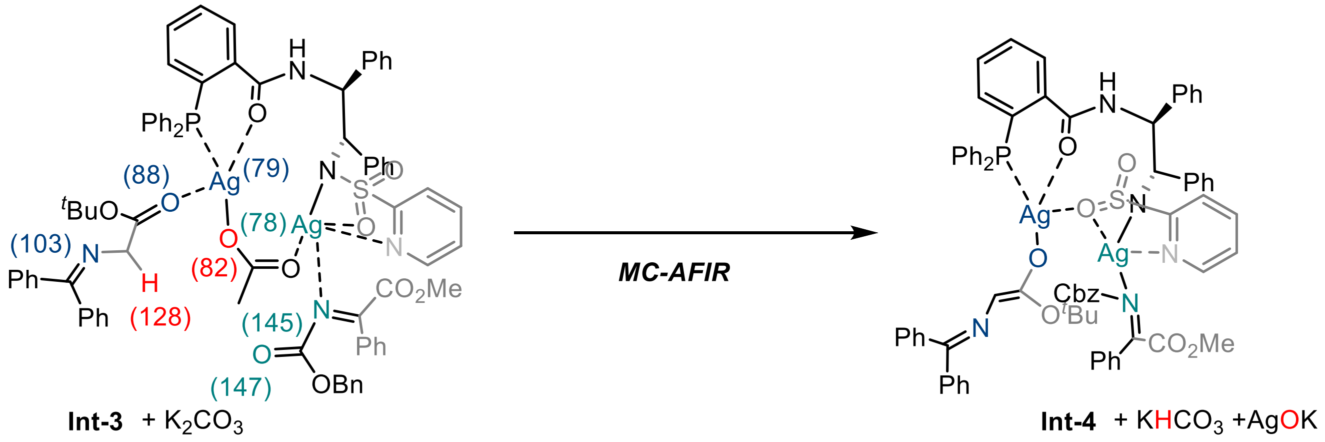

Scheme S5. MC-AFIR search of **Int-4**.

Calculated Structures

Int-4

M06-D3/def2-TZVPP/CPCM(THF)//B3LYP/def2-SV(P)/CPCM(THF)

B3LYP/def2-SV(P)/CPCM(THF) free energy: -4840.91761147 (a.u.)

M06-D3/def2-TZVPP/CPCM(THF) single point energy: -4842.94104079 (a.u.)

Thermal correction to Gibbs Free Energy: 1.114452

C	-3.57528	-0.95129	-0.07003
C	-4.8901	-0.12453	-0.29019
C	-3.85472	2.14457	-0.65496
C	-3.89794	3.58012	-0.19347
O	-3.06541	1.78763	-1.53031
C	-2.70191	4.32361	-0.03375
C	-2.79565	5.66691	0.36455
C	-4.03704	6.27632	0.57944
C	-5.21248	5.54285	0.41075
C	-5.13697	4.19991	0.03125
P	-1.0435	3.51138	-0.24902
S	-3.23364	-0.27198	2.52084
C	-3.01448	-1.83494	3.4607
O	-2.31302	0.68064	3.19882
C	-3.90091	-2.14443	4.49407
C	-3.65399	-3.30548	5.23196
C	-2.54692	-4.09487	4.90703
C	-1.72292	-3.69186	3.8524
N	-1.95959	-2.57973	3.1456
O	-4.68693	0.02719	2.64078
C	-5.56593	-0.43298	-1.62749
C	-6.91572	-0.82443	-1.62823
C	-7.58685	-1.11411	-2.82112
C	-6.91335	-1.01796	-4.04303
C	-5.56886	-0.62951	-4.05773
C	-4.90041	-0.3405	-2.86293
C	-3.88971	-2.44911	0.01286
C	-4.87137	-2.97001	0.87511
C	-5.10653	-4.34721	0.94912
C	-4.36618	-5.23508	0.16029
C	-3.38649	-4.73197	-0.70183

C	-3.15446	-3.35327	-0.77197
C	-0.68399	3.63374	-2.05091
C	-1.20547	4.66607	-2.84928
C	-0.90496	4.72545	-4.21408
C	-0.08148	3.75456	-4.79536
C	0.43848	2.72313	-4.00588
C	0.13729	2.65936	-2.64085
C	0.12371	4.70998	0.54026
C	0.28377	4.63988	1.9372
C	1.16024	5.50342	2.59938
C	1.90554	6.43978	1.87206
C	1.76362	6.5092	0.48263
C	0.87658	5.65193	-0.18063
H	-2.95302	-0.80731	-0.96218
H	-5.59617	-0.42393	0.48963
H	-1.88676	6.25428	0.51243
H	-4.07862	7.32787	0.87848
H	-6.18784	6.01134	0.57107
H	-6.05675	3.62679	-0.11387
H	-4.7535	-1.49616	4.70185
H	-4.32108	-3.59067	6.05081
H	-2.32107	-5.01051	5.45919
H	-0.84099	-4.27134	3.5621
H	-7.452	-0.90765	-0.67706
H	-8.63799	-1.41828	-2.79343
H	-7.43291	-1.24468	-4.97939
H	-5.03318	-0.54814	-5.00903
H	-3.8584	-0.01813	-2.89202
H	-5.45659	-2.30038	1.51042
H	-5.87579	-4.72786	1.62852
H	-4.5536	-6.31192	0.21655
H	-2.80018	-5.413	-1.32685
H	-2.38172	-2.97447	-1.44768
H	-1.85674	5.42748	-2.41092
H	-1.3198	5.53284	-4.82545
H	0.15028	3.79923	-5.86396
H	1.07808	1.9569	-4.45391
H	0.53191	1.83222	-2.04259
H	-0.27952	3.89893	2.51486

H	1.26947	5.43687	3.68604
H	2.59974	7.10996	2.38818
H	2.34552	7.23505	-0.09368
H	0.77758	5.72138	-1.26664
N	-2.69506	-0.46359	1.01848
N	-4.73527	1.31545	-0.04783
H	-5.1681	1.64546	0.80885
Ag	-0.66715	-1.82614	1.18315
Ag	-0.93312	1.30633	0.83523
C	2.01293	0.051	2.0873
O	0.74867	0.10982	1.97198
O	2.55056	-0.45613	3.2381
C	2.09325	-0.03588	4.55952
C	1.93392	1.48932	4.60112
H	1.11841	1.82224	3.93827
H	1.69741	1.81678	5.62875
H	2.86878	1.98473	4.28551
C	0.79035	-0.74658	4.94677
H	0.91105	-1.83981	4.8527
H	0.53607	-0.51857	5.99827
H	-0.04214	-0.43013	4.30171
C	3.23416	-0.47548	5.48428
H	3.00724	-0.21306	6.53228
H	3.37926	-1.56881	5.42537
H	4.17976	0.01491	5.19484
N	4.26548	0.4366	1.31417
C	5.17962	0.8969	0.50534
C	6.60098	0.75756	0.90223
C	7.65526	1.31116	0.14106
C	6.95077	0.06228	2.08565
C	8.98942	1.17298	0.53944
H	7.42915	1.86272	-0.77492
C	8.27994	-0.07272	2.48125
H	6.14422	-0.36528	2.68552
C	9.31439	0.48028	1.71017
H	9.78199	1.61524	-0.07359
H	8.51666	-0.61675	3.40218
H	10.35831	0.37169	2.02079
C	4.85734	1.60757	-0.78215

C	4.34565	2.91832	-0.75365
C	5.06465	1.00303	-2.03483
C	4.06214	3.60729	-1.93757
H	4.17783	3.40681	0.21145
C	4.77897	1.68785	-3.22185
H	5.45889	-0.01645	-2.08006
C	4.27986	2.99435	-3.17729
H	3.67035	4.62838	-1.89064
H	4.95017	1.19808	-4.18605
H	4.05943	3.53281	-4.10428
C	2.93012	0.44349	1.09331
H	2.49929	0.78688	0.14181
O	1.22433	-3.42187	2.01398
O	3.38191	-3.85069	1.5845
C	3.23596	-3.14405	-1.10658
C	4.53013	-2.69042	-0.78688
C	3.02458	-3.8303	-2.31818
C	5.58641	-2.9129	-1.672
H	4.70281	-2.14398	0.14189
C	4.0902	-4.06364	-3.19109
H	2.02871	-4.20403	-2.56798
C	5.37182	-3.60374	-2.87133
H	6.58554	-2.54516	-1.421
H	3.91576	-4.60901	-4.12293
H	6.20594	-3.78509	-3.55581
C	2.17665	-3.41448	1.26365
C	2.08967	-2.92926	-0.18497
N	0.94879	-2.44125	-0.5205
C	0.72127	-1.82084	-1.76787
O	1.13824	-0.72171	-2.046
O	-0.09612	-2.55941	-2.51827
C	-0.55622	-1.99914	-3.7855
H	-1.56654	-2.41453	-3.90194
H	-0.6167	-0.9076	-3.67571
C	0.33146	-2.39803	-4.93784
C	0.09008	-3.59485	-5.63214
C	1.41241	-1.59034	-5.32889
C	0.91291	-3.97989	-6.69545
H	-0.75473	-4.22787	-5.34126

C	2.23481	-1.97227	-6.39349
H	1.61249	-0.66004	-4.78933
C	1.98724	-3.16804	-7.07813
H	0.71156	-4.91305	-7.23046
H	3.07145	-1.33272	-6.69095
H	2.62919	-3.46536	-7.91319
C	3.58608	-4.28958	2.93749
H	2.89908	-5.11648	3.17702
H	4.63032	-4.62569	2.98507
H	3.41579	-3.44763	3.62705

3.2.4. Transition states

TS models (normal mode transition states and reverse mode transition states, in which the nucleophile and electrophile exchange their coordination positions) were manually investigated by the B3LYP/def2-SV(P)/CPCM(THF). Subsequently, single-point energy calculations were performed on the optimized geometries using M06-D3 functional with def2-TZVPP basis set for all atoms in CPCM solvation model (THF). The calculation results for the reaction between **1a** and **2a** using ligand **A** are shown in **Figure S6**, where the relative energies of the optimized structures are depicted. As a result, **TS-2S3R** was found to be more stable than **TS-2R3S**, **TS-2R3R**, and **TS-2S3S**.

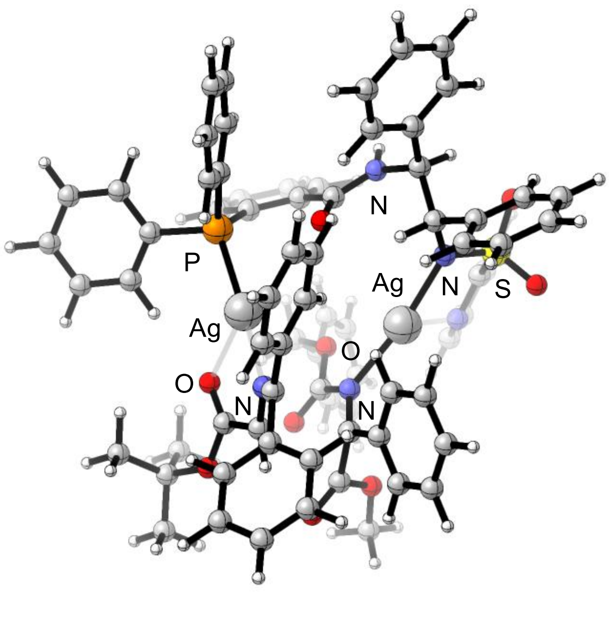

TS-2S3R

ΔΔG‡ +0.00 kcal/mol

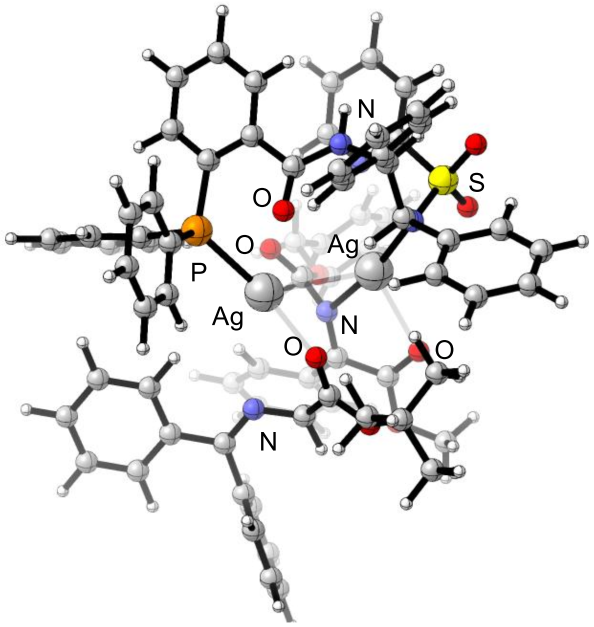

TS-2R3S

+2.29 kcal/mol

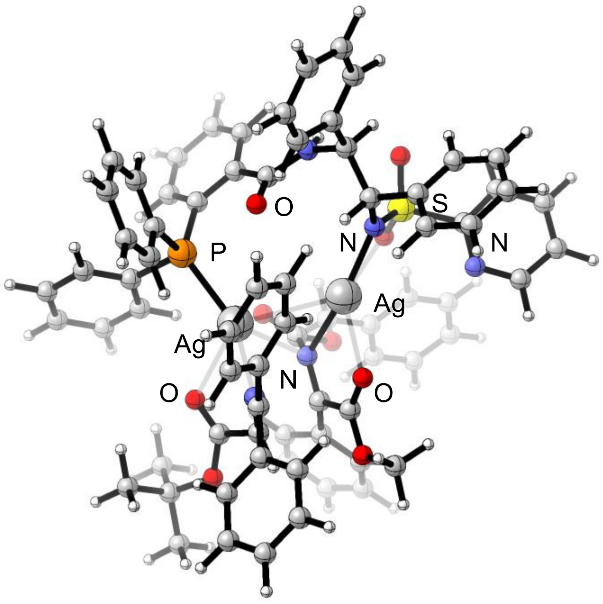

TS-2R3R

+3.33 kcal/mol

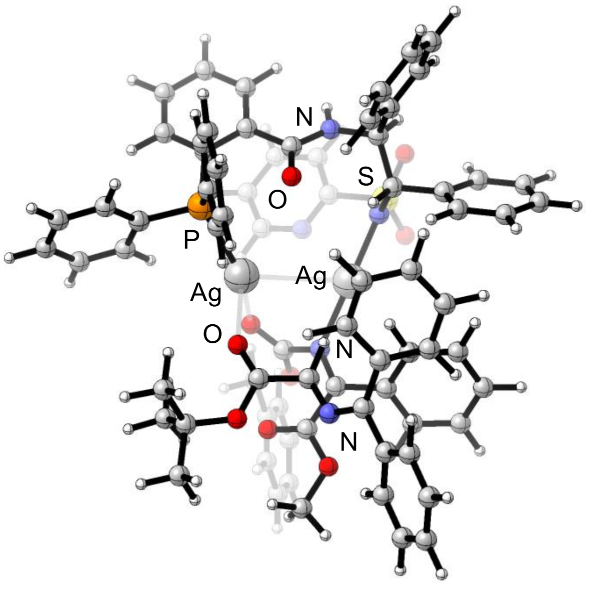

TS-2S3S

+8.98 kcal/mol

Figure S6. DFT calculation of transition states between **1a** and **2a** using Ligand **A** by Gaussian 16 M06-D3/def2-TZVPP/CPCM(THF)//B3LYP/def2-SV(P)/CPCM(THF).

Furthermore, the normal-mode transition state shown in **Figure S7** was found to be more stable than the reverse-mode transition state, in which the nucleophile and electrophile exchange their coordination positions.

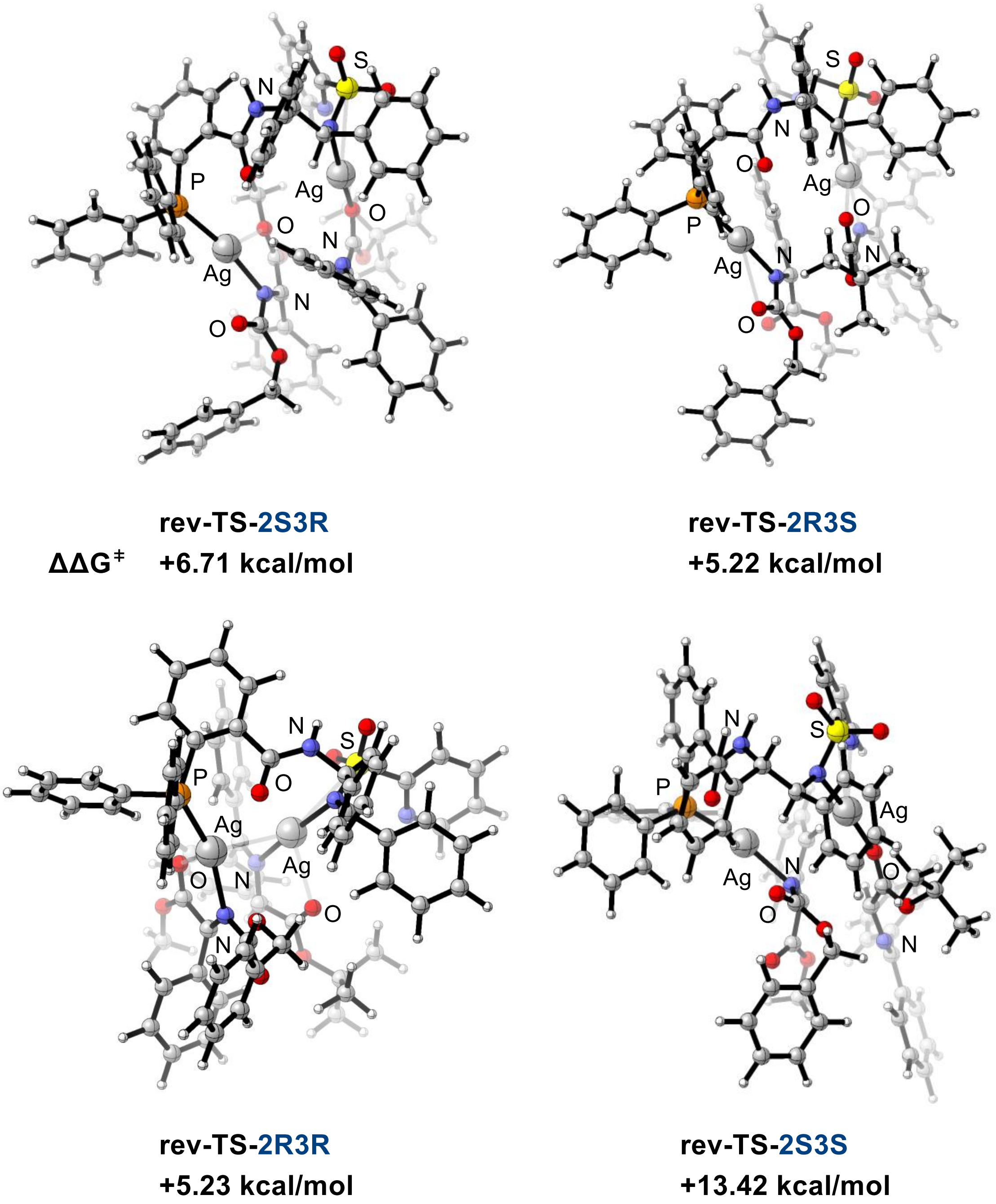

Figure S7. DFT calculation of transition states between **1a** and **2a** using **Ligand A** by Gaussian 16 M06-D3/def2-TZVPP/CPCM(THF)//B3LYP/def2-SV(P)/CPCM(THF).

3.2.5. Computational study (3D structure)

A computational analysis of the transition state **TS-2S3R** was performed using 3D structural data to evaluate the suitability of the designed ligand (Figure S8). The analysis revealed that the nucleophile and electrophile were properly accommodated within the asymmetric pocket created by the steric influence of

the phosphine moiety and the pyridinesulfonyl group of the ligand, each being coordinated to one of the two silver centers.

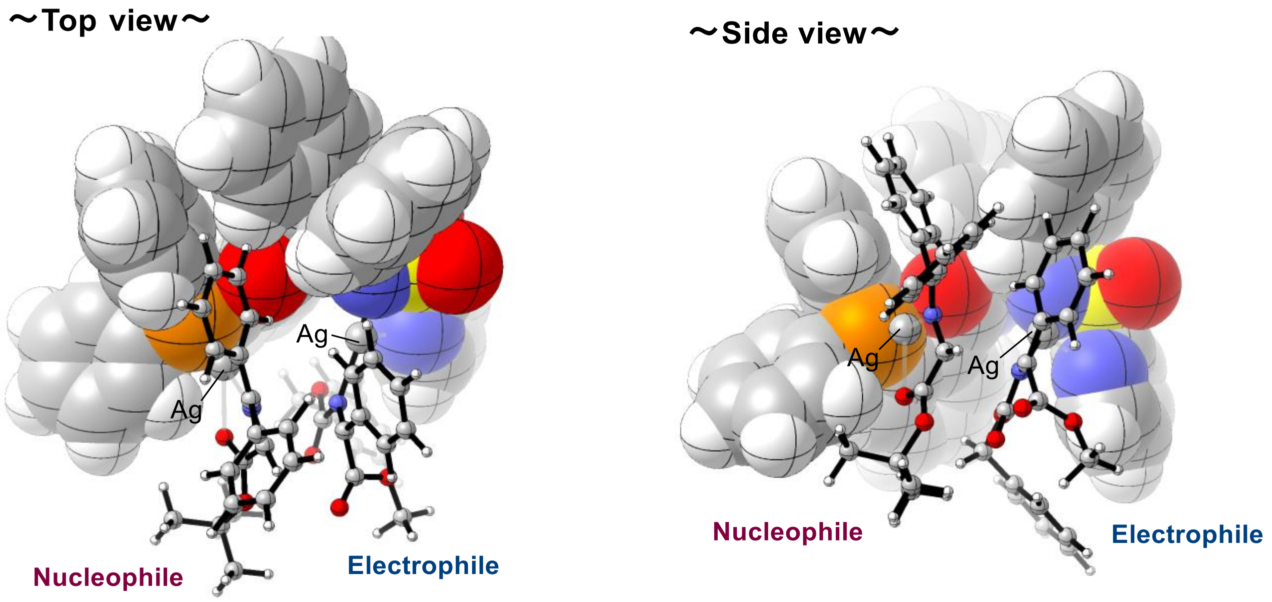

Figure S8. 3D structure of TS-2S3R

3.2.6. Distortion-Interaction analysis and NBO analysis

The results revealed that the interaction between the catalyst and the substrate plays a crucial role in the reaction. Moreover, this interaction provides greater stabilization in the transition state leading to the major isomer than in that leading to the minor isomer (**Table S1**).

	TS(kcal/mol)	ΔEcat(kcal/mol)	ΔEsub(kcal/mol)	ΔEint(kcal/mol)
ΔΔE(2R3S)-(2S3R)	**+2.58**	**-2.73**	**-0.08**	**+5.40**
ΔΔE(2R3R)-(2S3R)	**+3.37**	**-0.63**	**+6.33**	**-2.37**
ΔΔE(2S3S)-(2S3R)	**+11.2**	**-5.32**	**+0.74**	**+15.83**

Table S1. Distortion-Interaction analysis

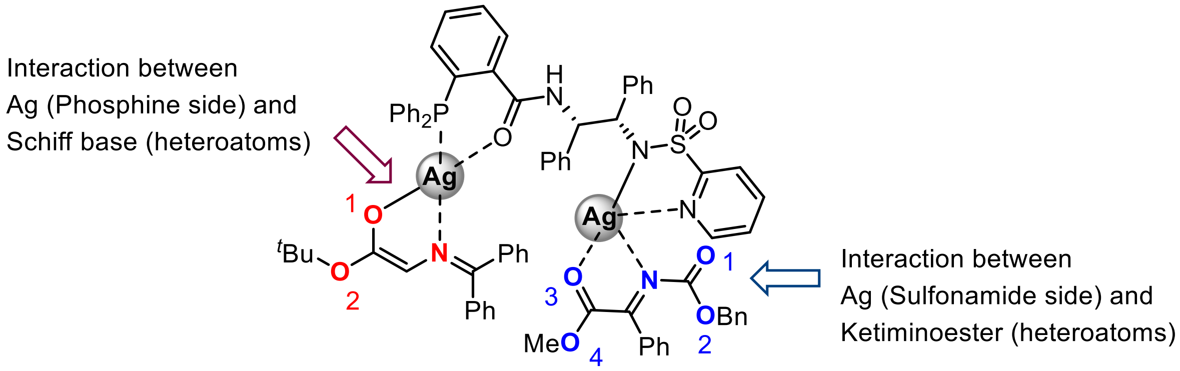

	TS-2S3R	TS-2R3S	TS-2R3R	TS-2S3S
**Ag-N** (Å)	**2.33 (57.45 kcal/mol)**	**3.33 (2.34kcal/mol)**	**2.39 (39.09 kcal/mol)**	**5.42 (0.00 kcal/mol)**
**Ag-O1** (Å)	**2.48 (14.6 kcal/mol)**	**2.31 (27.49 kcal/mol)**	**2.68 (7.06 kcal/mol)**	**2.37 (19.30 kcal/mol)**
**Ag-O2** (Å)	**4.45 0.60 kcal/mol**	**4.47 (0.89 kcal/mol)**	**4.59 (0.65 kcal/mol)**	**4.61 (0.51 kcal/mol)**
**Summary**	**72.65 kcal/mol**	**30.72 kcal/mol**	**50.74 kcal/mol**	**19.81 kcal/mol**

Table S2. NBO analysis; Interaction between Ag (Phosphine side) and Schiff base (heteroatoms)

	TS-2S3R	TS-2R3S	TS-2R3R	TS-2S3S
**Ag-N** (Å)	**2.24 (85.33 kcal/mol)**	**2.26 (74.31 kcal/mol)**	**2.29 (58.89 kcal/mol)**	**2.19 (94.49 kcal/mol)**
**Ag-O1** (Å)	**4.27 (0.74 kcal/mol)**	**3.41 (2.49 kcal/mol)**	**3.54 (2.94 kcal/mol)**	**3.29 (3.92 kcal/mol)**
**Ag-O2** (Å)	**2.91 (3.79 kcal/mol)**	**3.87 (0.77 kcal/mol)**	**3.53 (1.42 kcal/mol)**	**3.83 (0.42 kcal/mol)**
**Ag-O3** (Å)	**5.63 (0.00 kcal/mol)**	**2.68 (9.16 kcal/mol)**	**2.56 (11.58 kcal/mol)**	**4.98 (0.00 kcal/mol)**
**Ag-O4** (Å)	**4.57 (0.08 kcal/mol)**	**4.60 (0.58 kcal/mol)**	**4.52 (0.49 kcal/mol)**	**5.56 (0.00 kcal/mol)**
**Summary**	**102.71 kcal/mol**	**87.31 kcal/mol**	**75.32 kcal/mol**	**98.83 kcal/mol**

Table S3. NBO analysis; Interaction between Ag (Sulfonamide side) and Ketiminoester (heteroatoms)

3.2.8. IGMH analysis

The calculations of the Independent Gradient Model based on Hirshfeld (IGMH) analysis were performed by using Multiwfn (version 3.8) program.[10)] The graphics were depicted by using VMD (version 1.9.4a) program.[11)] The surfaces were colored on a blue-green-red (BGR) scale using VMD program. The green region indicates strong attractive interactions. The transition state structures were defined as two fragments, catalysts and substrates. Isosurface grids were generated with a dimension of 40×40×40 and an extension of 10 Bohr in all directions.

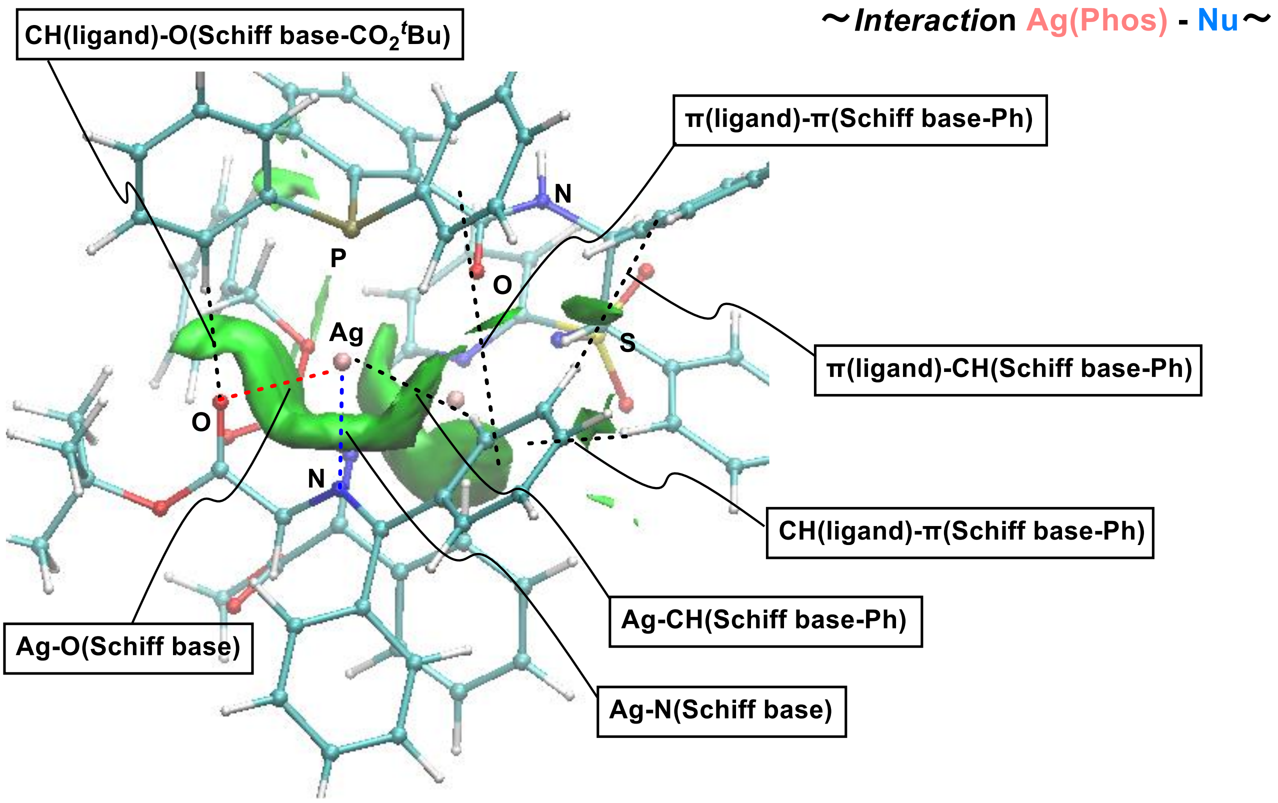

Figure S9. IGMH analysis of transition states between **1a** and **2** using ligand **A** and silver acetates on the phosphine side.

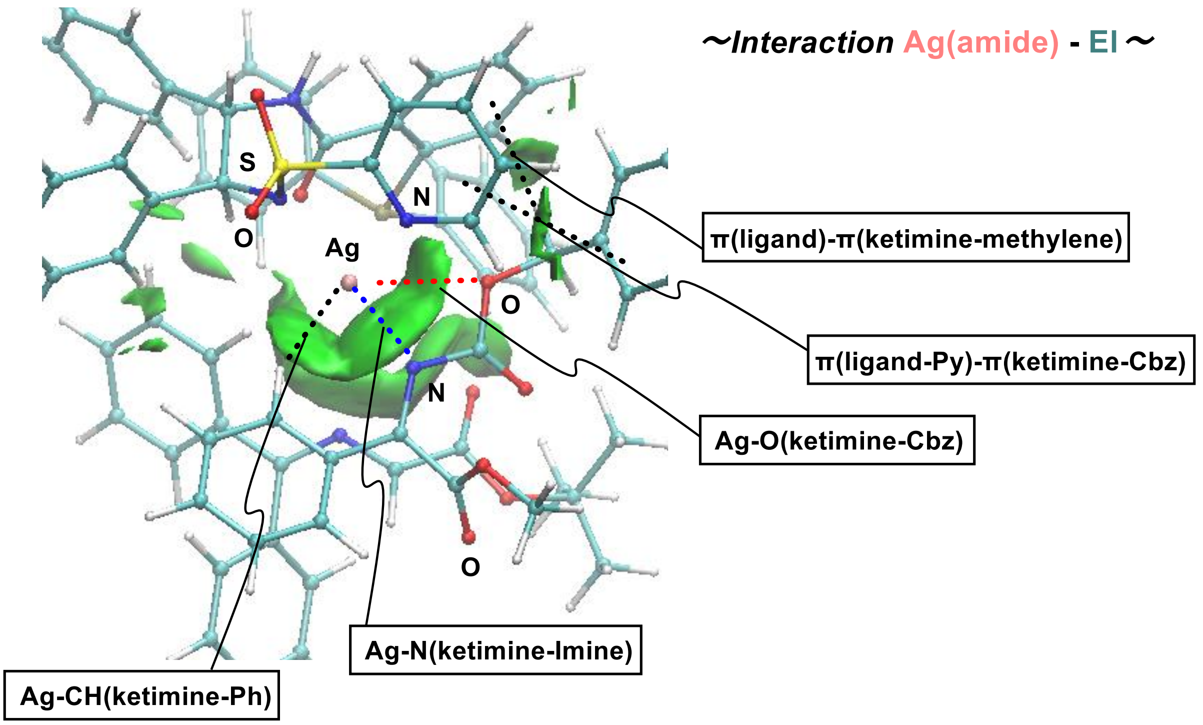

Figure S10. IGMH analysis of transition states between **1a** and **2** using ligand **A** and silver acetates on the heteroarenesulfonamide side.

Calculated Structures

TS-2S3R

M06-D3/def2-TZVPP/CPCM(THF)//B3LYP/def2-SV(P)/CPCM(THF)

Number of imaginary frequencies: 1 (91.568i)

B3LYP/def2-SV(P)/CPCM(THF) free energy: -4840.90227663 (a.u.)

M06-D3/def2-TZVPP/CPCM(THF) single point energy: -4842.93720828 (a.u.)

Thermal correction to Gibbs Free Energy: 1.124104

C	3.5994	0.43925	-1.0765
C	4.49511	0.3981	0.21158
C	2.48304	0.10522	1.75057
C	1.81436	0.66293	2.98643
O	1.91911	-0.77924	1.09778
C	0.80434	-0.07797	3.66162

C	0.20475	0.49736	4.79575
C	0.56838	1.76527	5.26027
C	1.54163	2.49839	4.58266
C	2.14907	1.9457	3.45342
P	0.16378	-1.72976	3.07123
S	3.60662	3.05244	-1.65543
C	2.6266	4.3899	-0.896
O	4.97871	3.1963	-1.0821
C	3.30306	5.4196	-0.23859
C	2.54328	6.49815	0.22559
C	1.16216	6.49425	0.01713
C	0.58917	5.40244	-0.64454
N	1.31206	4.37398	-1.09837
O	3.51886	3.32116	-3.11357
C	5.38674	-0.83295	0.33433
C	4.86339	-2.11987	0.5496
C	5.71097	-3.22831	0.64874
C	7.0978	-3.07193	0.53664
C	7.63076	-1.79558	0.3304
C	6.77993	-0.68872	0.23212
C	4.33693	-0.00479	-2.34537
C	5.51046	0.60506	-2.82563
C	6.12881	0.15033	-3.99486
C	5.59227	-0.92453	-4.71321
C	4.42854	-1.54362	-4.24774
C	3.81443	-1.08571	-3.07616
C	1.56794	-2.90912	3.24771
C	2.66628	-2.66415	4.08885
C	3.66232	-3.63363	4.25087
C	3.56975	-4.85747	3.57912
C	2.48053	-5.10616	2.73621
C	1.48789	-4.13552	2.56732
C	-0.96573	-2.2684	4.44398
C	-2.34912	-2.07277	4.27675
C	-3.24915	-2.47007	5.2725
C	-2.78076	-3.07746	6.44203
C	-1.40677	-3.28324	6.61414
C	-0.50524	-2.88126	5.62364
H	2.8179	-0.31005	-0.91578

H	5.14929	1.27685	0.15618
H	-0.56788	-0.05047	5.3368
H	0.0794	2.17624	6.14824
H	1.82675	3.49853	4.92087
H	2.88177	2.54926	2.91238
H	4.38411	5.36646	-0.10413
H	3.02757	7.33074	0.74471
H	0.53061	7.31484	0.36568
H	-0.4918	5.35371	-0.81201
H	3.7831	-2.24871	0.65096
H	5.28285	-4.2212	0.81797
H	7.75922	-3.94065	0.61383
H	8.71348	-1.65758	0.2472
H	7.20776	0.30596	0.07036
H	5.93778	1.45593	-2.29259
H	7.04066	0.64296	-4.34795
H	6.07949	-1.27714	-5.62783
H	3.99612	-2.38863	-4.79318
H	2.91029	-1.58725	-2.71609
H	2.74992	-1.71585	4.62709
H	4.51319	-3.43028	4.90821
H	4.34849	-5.61515	3.70878
H	2.40427	-6.05711	2.20042
H	0.64585	-4.33429	1.89695
H	-2.73199	-1.60853	3.36148
H	-4.32179	-2.30988	5.12601
H	-3.48492	-3.39521	7.21722
H	-1.03271	-3.76223	7.52429
H	0.56375	-3.05219	5.77505
N	2.85394	1.71247	-1.17682
N	3.6892	0.62681	1.42787
H	4.11384	1.2538	2.09982
Ag	-1.11964	-1.71091	0.97603
O	-3.396	-0.75397	1.22537
C	-3.9734	-0.76952	0.13004
O	-5.26777	-0.45149	-0.02662
C	-6.20366	-0.21028	1.07389
N	-2.27164	-2.02378	-1.02473
C	-2.00891	-2.97946	-1.89398

C	-2.9998	-3.41292	-2.93495
C	-2.63409	-3.50336	-4.28927
C	-4.29837	-3.81658	-2.56403
C	-3.54304	-3.95996	-5.24936
H	-1.62785	-3.20601	-4.59449
C	-5.20223	-4.28602	-3.52007
H	-4.59916	-3.77243	-1.51347
C	-4.83011	-4.35362	-4.86873
H	-3.24085	-4.01102	-6.3001
H	-6.20239	-4.60426	-3.20974
H	-5.53998	-4.71757	-5.61796
C	-0.76311	-3.77905	-1.78876
C	-0.76907	-5.1442	-2.15792
C	0.45337	-3.23588	-1.3217
C	0.37848	-5.93149	-2.04772
H	-1.6935	-5.59952	-2.52018
C	1.60344	-4.02325	-1.21886
H	0.51652	-2.17707	-1.05864
C	1.57458	-5.37501	-1.57991
H	0.33661	-6.98822	-2.32971
H	2.53442	-3.57067	-0.86722
H	2.47855	-5.98678	-1.50371
Ag	0.662	1.78237	-1.25398
C	-5.79223	1.0008	1.91828
H	-4.86569	0.80397	2.4767
H	-5.62469	1.87943	1.27605
H	-6.59961	1.23291	2.6359
C	-6.33395	-1.48203	1.92257
H	-7.13126	-1.34844	2.67483
H	-6.6032	-2.34422	1.28692
H	-5.3939	-1.71075	2.44877
C	-7.51647	0.07786	0.33722
H	-7.80372	-0.7806	-0.29445
H	-8.32847	0.26965	1.05942
H	-7.40597	0.96296	-0.31259
C	-3.37107	-1.21211	-1.11931
H	-4.04101	-1.28936	-1.97486
C	-2.36239	1.0549	-2.14353
C	-1.81269	0.39171	-3.3395

C	-2.48128	0.47303	-4.58532
C	-0.57029	-0.2779	-3.30433
C	-1.90289	-0.03903	-5.74543
H	-3.45692	0.95586	-4.65919
C	0.0066	-0.79018	-4.46728
H	-0.04599	-0.38784	-2.35397
C	-0.65085	-0.66415	-5.69569
H	-2.43223	0.0571	-6.6979
H	0.97783	-1.28919	-4.41184
H	-0.19375	-1.05743	-6.60854
C	-3.72515	1.72359	-2.37216
O	-4.77866	1.16815	-2.55526
O	-3.53681	3.04305	-2.53182
C	-4.70289	3.8299	-2.79321
H	-5.40285	3.76655	-1.94352
H	-5.20999	3.48436	-3.70902
N	-1.55547	1.49287	-1.17006
C	-2.17109	2.08995	-0.07942
O	-3.37236	2.18739	0.09883
O	-1.25809	2.54912	0.80011
C	-1.73883	3.04553	2.07068
H	-0.98141	2.72378	2.79907
H	-2.69149	2.54148	2.29181
C	-1.89977	4.54915	2.11099
C	-1.10903	5.31382	2.98206
C	-2.8583	5.20511	1.31704
C	-1.2694	6.70206	3.0658
H	-0.35896	4.81702	3.60562
C	-3.01221	6.59244	1.39077
H	-3.48744	4.61952	0.64176
C	-2.22017	7.34555	2.2676
H	-0.64601	7.28122	3.75414
H	-3.76169	7.08908	0.76652
H	-2.34744	8.4309	2.32916
H	-4.34764	4.86229	-2.91875

TS-2R3S

M06-D3/def2-TZVPP/CPCM(THF)//B3LYP/def2-SV(P)/CPCM(THF)

Number of imaginary frequencies: 1 (124.1452i)

B3LYP/def2-SV(P)/CPCM(THF) free energy: -4840.89948101 (a.u.)

M06-D3/def2-TZVPP/CPCM(THF) single point energy: -4842.93309409 (a.u.)

Thermal correction to Gibbs Free Energy: 1.123642

C	3.77385	-1.35703	0.80362
C	4.91127	-0.6326	-0.0015
C	3.51831	1.32633	-0.84966
C	3.27668	2.19526	-2.05812
O	2.95992	1.5907	0.21981
C	2.12409	3.02319	-2.13404
C	1.94372	3.80066	-3.29229
C	2.87658	3.80488	-4.33315
C	4.03014	3.02846	-4.23279
C	4.2191	2.23443	-3.10157
P	0.85155	3.14584	-0.76935
S	3.2321	-3.06468	-1.26049
C	3.00288	-2.15321	-2.83623
O	4.66688	-3.46726	-1.28294
C	3.99087	-2.21298	-3.82403
C	3.74466	-1.54328	-5.02766
C	2.54235	-0.84933	-5.18328
C	1.6263	-0.85706	-4.1242
N	1.85158	-1.50496	-2.97815
O	2.23634	-4.16001	-1.3585
C	5.99849	0.03067	0.84253
C	5.76806	1.1731	1.63063
C	6.80394	1.7485	2.37338
C	8.08859	1.19251	2.34981
C	8.33005	0.05619	1.57174
C	7.29321	-0.51393	0.82437
C	4.33817	-2.15775	1.98493
C	4.82606	-3.46933	1.86392
C	5.34115	-4.14849	2.97459
C	5.38089	-3.53169	4.22991
C	4.89314	-2.22733	4.36548
C	4.37504	-1.55482	3.25406
C	1.59696	4.29206	0.46956

C	2.71899	5.0907	0.19643
C	3.22791	5.95368	1.17372
C	2.61856	6.03381	2.43049
C	1.49716	5.24395	2.70866
C	0.99318	4.37483	1.73598
C	-0.45811	4.19015	-1.56711
C	-1.42591	3.54222	-2.35842
C	-2.39801	4.28464	-3.03661
C	-2.42895	5.67935	-2.92135
C	-1.48545	6.32881	-2.11814
C	-0.50436	5.59004	-1.4461
H	3.16839	-0.55514	1.24356
H	5.41061	-1.41737	-0.58216
H	1.06188	4.43405	-3.3893
H	2.70195	4.43236	-5.21203
H	4.78524	3.04096	-5.02373
H	5.14318	1.6566	-3.02651
H	4.91273	-2.76886	-3.64798
H	4.4864	-1.56416	-5.83195
H	2.31353	-0.30766	-6.10478
H	0.67598	-0.31622	-4.19223
H	4.77097	1.61629	1.65933
H	6.60372	2.63849	2.97869
H	8.89659	1.64384	2.93423
H	9.32973	-0.3887	1.5411
H	7.49521	-1.40205	0.21655
H	4.80969	-3.9612	0.89001
H	5.71526	-5.17064	2.85476
H	5.78413	-4.06499	5.09676
H	3.98473	-0.53995	3.37823
H	3.20631	5.04206	-0.78089
H	4.10611	6.56677	0.94876
H	3.01872	6.7086	3.19341
H	1.01564	5.29691	3.68979
H	0.12211	3.75283	1.96868
H	-1.4142	2.45187	-2.45266
H	-3.13941	3.76851	-3.65443
H	-3.1922	6.25845	-3.45023
H	-1.50721	7.41819	-2.0155

H	0.23349	6.11447	-0.83375
N	2.8276	-2.10576	-0.04379
N	4.36439	0.28079	-1.02204
H	4.68798	0.10314	-1.96455
Ag	-0.12021	1.11997	0.29135
O	-0.298	-0.07138	2.25939
C	-1.27174	-0.70498	2.69272
O	-1.17685	-1.63983	3.64534
C	0.0314	-1.95153	4.43317
N	-3.02759	0.71327	1.86398
C	-4.20297	1.2035	2.14381
C	-5.18029	0.48956	3.04344
C	-4.9694	0.49319	4.43391
C	-6.30415	-0.17865	2.53158
C	-5.86221	-0.1558	5.29262
H	-4.09841	1.01211	4.84625
C	-7.1954	-0.83195	3.39074
H	-6.48048	-0.19207	1.45272
C	-6.9784	-0.82155	4.77277
H	-5.68473	-0.14084	6.37256
H	-8.06403	-1.3523	2.97511
H	-7.67715	-1.33069	5.44382
C	-4.56389	2.5596	1.66684
C	-5.79632	3.15381	2.015
C	-3.66872	3.31136	0.87467
C	-6.11997	4.44512	1.58874
H	-6.5098	2.60292	2.63119
C	-3.99014	4.60097	0.45478
H	-2.70898	2.86887	0.59918
C	-5.21927	5.17621	0.80824
H	-7.08227	4.88205	1.87336
H	-3.27731	5.1623	-0.1553
H	-5.4702	6.18846	0.47636
Ag	0.65571	-1.80395	-0.14636
C	0.66545	-0.67253	4.99263
H	1.12922	-0.06443	4.20284
H	-0.09166	-0.0609	5.51477
H	1.44368	-0.94954	5.72483
C	1.00213	-2.76164	3.57551

H	1.37799	-2.17002	2.72781
H	1.86807	-3.07157	4.18474
H	0.50627	-3.66083	3.17613
C	-0.52521	-2.80378	5.57833
H	-1.02169	-3.70909	5.18939
H	0.29441	-3.11956	6.24581
H	-1.25947	-2.23077	6.17061
C	-2.65516	-0.53666	2.26463
H	-3.37118	-1.18818	2.77461
C	-2.54034	-1.79549	0.18361
C	-3.94767	-1.54835	-0.24857
C	-4.30613	-0.26886	-0.72889
C	-4.92481	-2.56566	-0.30213
C	-5.58041	-0.01728	-1.24232
H	-3.57616	0.5408	-0.7051
C	-6.19702	-2.31244	-0.81908
H	-4.68712	-3.57177	0.03941
C	-6.53511	-1.03839	-1.29012
H	-5.82645	0.9856	-1.60382
H	-6.92856	-3.12521	-0.86186
H	-7.53385	-0.84441	-1.69293
C	-2.17866	-3.0035	1.01149
O	-1.06828	-3.50378	1.00237
O	-3.17946	-3.48679	1.75141
C	-2.91711	-4.66477	2.52207
H	-2.17182	-4.45016	3.30322
H	-2.549	-5.47696	1.87496
N	-1.51262	-1.27393	-0.52412
C	-1.64375	-0.82515	-1.8232
O	-1.12499	0.19327	-2.25956
O	-2.32756	-1.67847	-2.60983
C	-2.55416	-1.28755	-3.98117
H	-1.5833	-1.06977	-4.45216
H	-3.1587	-0.36568	-3.98839
C	-3.25981	-2.4179	-4.68363
C	-2.52248	-3.46468	-5.26033
C	-4.66119	-2.45773	-4.74992
C	-3.172	-4.53045	-5.89075
H	-1.42856	-3.44276	-5.21613

C	-5.31428	-3.52131	-5.38182
H	-5.24591	-1.64825	-4.30109
C	-4.57039	-4.56022	-5.95285
H	-2.58517	-5.33879	-6.33786
H	-6.40755	-3.5386	-5.42902
H	-5.08004	-5.39214	-6.44895
H	-3.87803	-4.94139	2.97779
H	4.90814	-1.73198	5.3418

TS-2R3R

M06-D3/def2-TZVPP/CPCM(THF)//B3LYP/def2-SV(P)/CPCM(THF)

Number of imaginary frequencies: 1 (108.8661i)

B3LYP/def2-SV(P)/CPCM(THF) free energy: -4840.90454234 (a.u.)

M06-D3/def2-TZVPP/CPCM(THF) single point energy: -4842.93189181 (a.u.)

Thermal correction to Gibbs Free Energy: 1.124101

C	-3.8503	-0.12997	0.63161
C	-4.63599	-0.88983	-0.49107
C	-2.65785	-1.66955	-1.87982
C	-2.09241	-1.69324	-3.27885
O	-2.05	-2.22486	-0.96116
C	-0.7559	-2.11962	-3.51303
C	-0.28866	-2.12576	-4.84014
C	-1.09794	-1.74583	-5.91407
C	-2.41709	-1.35906	-5.6837
C	-2.90094	-1.34278	-4.37597
P	0.41513	-2.66765	-2.15802
S	-3.54485	2.17856	-0.75359
C	-4.11718	3.54537	0.32739
O	-2.39109	2.77106	-1.47971
C	-5.31372	4.20349	0.02885
C	-5.67556	5.28476	0.83776
C	-4.83622	5.64999	1.89339
C	-3.66049	4.91727	2.09455
N	-3.30607	3.88632	1.32234
O	-4.73782	1.83632	-1.58133
C	-5.29424	-2.18379	-0.01494

C	-4.56909	-3.2718	0.50268
C	-5.22502	-4.43216	0.92874
C	-6.61889	-4.52888	0.85051
C	-7.35285	-3.45467	0.33736
C	-6.69343	-2.29718	-0.0901
C	-4.76007	0.29129	1.78853
C	-5.98205	0.95835	1.59138
C	-6.76679	1.35826	2.67869
C	-6.34615	1.10006	3.98806
C	-5.13189	0.43879	4.20026
C	-4.35164	0.04038	3.10946
C	-0.16148	-4.34729	-1.66049
C	-1.01299	-5.12599	-2.46089
C	-1.38812	-6.41089	-2.05372
C	-0.91158	-6.93524	-0.84681
C	-0.06043	-6.16601	-0.04577
C	0.30823	-4.87776	-0.44813
C	1.96044	-3.05638	-3.11943
C	2.80966	-1.98605	-3.46049
C	3.98543	-2.21367	-4.18135
C	4.34457	-3.51503	-4.55317
C	3.51549	-4.58616	-4.20598
C	2.32863	-4.35882	-3.49854
H	-3.12909	-0.84603	1.04475
H	-5.44996	-0.23291	-0.81106
H	0.73149	-2.44334	-5.05439
H	-0.69323	-1.7705	-6.93013
H	-3.07378	-1.08065	-6.51269
H	-3.94778	-1.07545	-4.21928
H	-5.93136	3.87147	-0.80663
H	-6.60278	5.83294	0.645
H	-5.08254	6.48737	2.55169
H	-2.97633	5.17539	2.91162
H	-3.48077	-3.21735	0.54705
H	-4.63876	-5.26835	1.32332
H	-7.12974	-5.43691	1.18617
H	-8.44363	-3.51521	0.26841
H	-7.28004	-1.46302	-0.48878
H	-6.32965	1.18002	0.57934

H	-7.71497	1.87509	2.4994
H	-6.96225	1.4112	4.83752
H	-4.79008	0.22839	5.21868
H	-3.40341	-0.47679	3.28861
H	-1.39078	-4.73267	-3.40859
H	-2.05564	-7.00516	-2.68547
H	-1.20572	-7.9406	-0.52993
H	0.31299	-6.5639	0.90259
H	0.9615	-4.27818	0.19468
H	2.54734	-0.96643	-3.16301
H	4.63024	-1.36948	-4.44456
H	5.27029	-3.69308	-5.10926
H	3.7885	-5.60756	-4.48924
H	1.68933	-5.20774	-3.24536
N	-2.99212	0.9543	0.1206
N	-3.8526	-1.04608	-1.72503
H	-4.11835	-0.37786	-2.4399
Ag	1.25405	-1.18517	-0.32564
O	3.88925	-0.78598	-0.62155
C	4.26373	-0.2079	0.39493
O	5.50952	0.26797	0.57773
C	6.66955	-0.14979	-0.22887
N	2.41266	-0.91136	1.74679
C	2.22719	-1.54708	2.87105
C	3.1852	-1.49333	4.02403
C	2.70329	-1.27481	5.32902
C	4.56365	-1.72293	3.84747
C	3.57643	-1.25346	6.42081
H	1.63243	-1.12115	5.48953
C	5.43279	-1.72159	4.94104
H	4.95785	-1.92256	2.84794
C	4.94409	-1.47749	6.23039
H	3.18383	-1.06842	7.42527
H	6.49839	-1.91519	4.78514
H	5.62726	-1.47081	7.08514
C	1.05354	-2.44426	3.05074
C	1.22626	-3.6956	3.68013
C	-0.23793	-2.07847	2.6296
C	0.14696	-4.5619	3.86291

H	2.22127	-3.99688	4.01789
C	-1.32041	-2.94029	2.83108
H	-0.39686	-1.09641	2.17622
C	-1.1341	-4.18395	3.44341
H	0.30609	-5.53362	4.34025
H	-2.32095	-2.63621	2.51409
H	-1.98583	-4.85312	3.59827
Ag	-0.84699	1.063	0.41167
C	7.83068	0.58662	0.44468
H	7.67596	1.67838	0.3978
H	7.91472	0.29252	1.5051
H	8.78035	0.34565	-0.06287
C	6.53545	0.28274	-1.69301
H	6.39868	1.37368	-1.76658
H	7.46282	0.0208	-2.23255
H	5.6886	-0.21923	-2.18297
C	6.84437	-1.66819	-0.10404
H	6.00333	-2.20804	-0.56675
H	7.77483	-1.97751	-0.61123
H	6.91873	-1.96413	0.95739
C	3.42185	0.02418	1.56878
H	3.94064	0.41782	2.44553
C	2.19353	1.94539	0.85481
C	3.24897	2.95387	0.5058
C	3.39628	4.1572	1.22582
C	4.03474	2.78236	-0.65084
C	4.30033	5.13891	0.81045
H	2.7899	4.34627	2.11042
C	4.93693	3.76312	-1.06557
H	3.93489	1.87089	-1.24251
C	5.07855	4.94792	-0.33457
H	4.3867	6.06537	1.38644
H	5.53144	3.60328	-1.97002
H	5.78519	5.71747	-0.65971
C	1.65306	1.87618	2.25857
O	0.54885	1.43072	2.52731
O	2.47821	2.34279	3.19844
C	1.9888	2.39053	4.54347
H	1.81231	1.37419	4.92858

H	1.05347	2.97099	4.59354
N	1.35075	1.46844	-0.08079
C	1.3294	1.88503	-1.40967
O	1.35201	1.13066	-2.36573
O	1.1589	3.21299	-1.53512
C	0.99603	3.72712	-2.87581
H	0.19517	3.15338	-3.36765
H	1.93273	3.56339	-3.43349
C	0.65367	5.19142	-2.78705
C	-0.64781	5.59111	-2.43654
C	1.62467	6.17226	-3.0379
C	-0.96667	6.949	-2.34032
H	-1.40809	4.82883	-2.23479
C	1.30486	7.53191	-2.946
H	2.64138	5.8694	-3.30939
C	0.00795	7.92234	-2.59599
H	-1.98302	7.25079	-2.06779
H	2.07091	8.28717	-3.14794
H	-0.24488	8.985	-2.52418
H	2.77813	2.88389	5.12755

TS-2S3S

M06-D3/def2-TZVPP/CPCM(THF)//B3LYP/def2-SV(P)/CPCM(THF)

Number of imaginary frequencies: 1 (124.5811i)

B3LYP/def2-SV(P)/CPCM(THF) free energy: -4840.89927118 (a.u.)

M06-D3/def2-TZVPP/CPCM(THF) single point energy: -4842.919282 (a.u.)

Thermal correction to Gibbs Free Energy: 1.120482

C	-2.5431	2.17902	-1.71289
C	-4.0841	2.02475	-1.49553
C	-4.03646	0.00924	0.0515
C	-4.76954	-1.25676	0.42116
O	-3.09235	0.39312	0.74816
C	-4.20826	-2.15337	1.37171
C	-4.92293	-3.32221	1.68828
C	-6.16749	-3.60262	1.11731
C	-6.73363	-2.70314	0.21475

C	-6.03506	-1.54296	-0.12103
P	-2.55525	-1.87238	2.18677
S	-2.46311	0.41442	-3.80157
C	-2.85063	-1.32975	-3.40758
O	-3.7503	0.99595	-4.27611
C	-4.07111	-1.87914	-3.80968
C	-4.29274	-3.23235	-3.53309
C	-3.29836	-3.96269	-2.87726
C	-2.11269	-3.3072	-2.52374
N	-1.89472	-2.01629	-2.79043
O	-1.38228	0.30407	-4.80957
C	-4.69028	3.12053	-0.61998
C	-4.29699	3.33411	0.71393
C	-4.89183	4.34173	1.48039
C	-5.88596	5.15914	0.92944
C	-6.28359	4.95859	-0.39609
C	-5.68995	3.94692	-1.15934
C	-2.20433	3.56166	-2.28174
C	-2.51335	3.92465	-3.60495
C	-2.18662	5.19347	-4.09411
C	-1.54225	6.1264	-3.27237
C	-1.22489	5.77584	-1.95647
C	-1.55401	4.50458	-1.46986
C	-2.82378	-0.58002	3.47217
C	-4.09966	-0.22627	3.93877
C	-4.24252	0.74002	4.9409
C	-3.11358	1.35742	5.49081
C	-1.8388	1.00743	5.03074
C	-1.69418	0.04769	4.02311
C	-2.33024	-3.40707	3.21311
C	-1.6685	-4.50418	2.63064
C	-1.45442	-5.68011	3.356
C	-1.88673	-5.77215	4.68399
C	-2.53695	-4.68504	5.2775
C	-2.75977	-3.51161	4.54794
H	-2.08964	2.1292	-0.71542
H	-4.54945	2.1073	-2.48314
H	-4.50987	-4.03282	2.40505
H	-6.69567	-4.51944	1.39492

H	-7.7173	-2.89333	-0.22349
H	-6.51418	-0.8381	-0.80413
H	-4.81144	-1.26238	-4.32124
H	-5.23246	-3.70883	-3.82842
H	-3.43324	-5.02158	-2.64173
H	-1.30389	-3.83631	-2.00704
H	-3.52823	2.69965	1.159
H	-4.57425	4.48913	2.51767
H	-6.34765	5.94852	1.53096
H	-7.05915	5.59048	-0.84041
H	-6.00834	3.79879	-2.19656
H	-3.00377	3.20203	-4.26169
H	-2.43556	5.45446	-5.12791
H	-1.28602	7.11848	-3.6575
H	-0.71709	6.49217	-1.30271
H	-1.29882	4.24351	-0.43752
H	-4.99159	-0.70052	3.52006
H	-5.24283	1.00962	5.29352
H	-3.22709	2.11207	6.27506
H	-0.95035	1.48639	5.4537
H	-0.69312	-0.20674	3.65873
H	-1.3129	-4.44049	1.59639
H	-0.93982	-6.5227	2.8843
H	-1.71199	-6.68806	5.2568
H	-2.87411	-4.74735	6.31677
H	-3.27156	-2.67396	5.0283
N	-1.90775	1.07694	-2.4515
N	-4.46989	0.67497	-1.04615
H	-5.17766	0.23089	-1.6168
Ag	-0.48161	-1.68774	0.89519
O	1.55802	-1.24784	2.02671
C	2.61331	-0.59924	2.07048
O	3.68827	-1.01737	2.75462
C	3.68388	-2.16833	3.66665
N	3.84746	1.47008	1.70307
C	3.82969	2.7643	1.83501
C	2.57761	3.5855	1.93411
C	1.54493	3.20532	2.81372
C	2.41606	4.77123	1.19232

C	0.38808	3.97919	2.94221
H	1.65792	2.30125	3.41825
C	1.25765	5.54392	1.31578
H	3.20567	5.0885	0.50604
C	0.23796	5.15072	2.19059
H	-0.39819	3.66677	3.63642
H	1.15202	6.46002	0.7262
H	-0.66754	5.75707	2.29001
C	5.14112	3.45555	1.9828
C	5.26864	4.69448	2.64458
C	6.31295	2.84776	1.48343
C	6.51958	5.29818	2.80569
H	4.38321	5.18547	3.05557
C	7.56005	3.45287	1.63994
H	6.21672	1.89406	0.96092
C	7.67114	4.68288	2.30297
H	6.5937	6.25524	3.33181
H	8.45395	2.96608	1.2366
H	8.64916	5.15916	2.42376
Ag	-0.04619	0.19706	-1.84143
C	3.40367	-3.47672	2.91784
H	2.36062	-3.52717	2.57233
H	4.06448	-3.5614	2.04029
H	3.59567	-4.33071	3.59185
C	2.67847	-1.92462	4.79922
H	1.64432	-1.90849	4.42218
H	2.76303	-2.73027	5.5496
H	2.88917	-0.96415	5.30219

rev-TS-2S3R (Reverse mode)

M06-D3/def2-TZVPP/CPCM(THF)//B3LYP/def2-SV(P)/CPCM(THF)

Number of imaginary frequencies: 1 (126.6729i)

B3LYP/def2-SV(P)/CPCM(THF) free energy: -4840.89860666 (a.u.)

M06-D3/def2-TZVPP/CPCM(THF) single point energy: -4842.92801091 (a.u.)

Thermal correction to Gibbs Free Energy: 1.123222

C	3.17107	-1.35399	1.31877

C	4.32269	-0.35954	0.98474
C	3.02227	1.2104	-0.53337
C	3.02834	1.87086	-1.89544
O	2.0914	1.40942	0.25151
C	2.28509	3.05929	-2.13918
C	2.31564	3.61369	-3.4308
C	3.03985	3.02236	-4.47014
C	3.74939	1.846	-4.23566
C	3.73712	1.28361	-2.95784
P	1.16773	3.85263	-0.87965
S	3.74522	-3.27258	-0.47948
C	4.07496	-2.75863	-2.2102
O	5.09261	-3.27844	0.15129
C	5.39957	-2.54527	-2.60937
C	5.63348	-2.22877	-3.95142
C	4.54669	-2.14463	-4.82524
C	3.26434	-2.37567	-4.31279
N	3.03283	-2.67784	-3.03234
O	3.07676	-4.58615	-0.63262
C	4.71831	0.55839	2.14376
C	3.78101	1.16023	2.99973
C	4.19991	1.98503	4.0493
C	5.56164	2.23055	4.25898
C	6.50465	1.64456	3.40816
C	6.08214	0.81525	2.36399
C	3.42305	-2.16402	2.6018
C	4.69034	-2.57252	3.04839
C	4.8317	-3.33638	4.21273
C	3.70775	-3.71591	4.95439
C	2.43822	-3.31846	4.52087
C	2.30344	-2.54823	3.36096
C	2.23754	4.46349	0.48527
C	3.61515	4.68669	0.32856
C	4.36235	5.24069	1.37419
C	3.74098	5.57983	2.58066
C	2.36945	5.35397	2.7447
C	1.62204	4.79367	1.70466
C	0.61842	5.42632	-1.68906
C	-0.6636	5.47048	-2.26459

C	-1.13109	6.63741	-2.87912
C	-0.32484	7.77951	-2.91646
C	0.9504	7.74933	-2.33927
C	1.41987	6.58193	-1.73005
H	2.28123	-0.74385	1.51539
H	5.20463	-0.96228	0.736
H	1.75867	4.52769	-3.64141
H	3.03696	3.48361	-5.46196
H	4.30595	1.35714	-5.03991
H	4.27262	0.34418	-2.8031
H	6.21029	-2.6386	-1.88606
H	6.65357	-2.05813	-4.30892
H	4.68308	-1.90768	-5.88369
H	2.38698	-2.31818	-4.96821
H	2.71355	0.98592	2.85118
H	3.45391	2.43967	4.70847
H	5.88583	2.87415	5.08285
H	7.57335	1.82499	3.56174
H	6.82895	0.34898	1.71207
H	5.58446	-2.30805	2.48309
H	5.83171	-3.63957	4.54001
H	3.82115	-4.31517	5.86354
H	1.54749	-3.60223	5.09097
H	1.30989	-2.23289	3.02823
H	4.11357	4.43303	-0.61136
H	5.43547	5.40864	1.24223
H	4.32704	6.01403	3.3962
H	1.87862	5.60905	3.68871
H	0.55121	4.61189	1.84369
H	-1.30838	4.58569	-2.23048
H	-2.13134	6.65374	-3.32215
H	-0.69125	8.69542	-3.39013
H	1.58408	8.6412	-2.36038
H	2.41682	6.57749	-1.28148
N	2.74224	-2.19833	0.17967
N	4.07546	0.40666	-0.25323
H	4.84597	0.4114	-0.90993
Ag	-0.72978	2.47567	-0.27164
O	-0.36525	-0.99726	1.84018

C	-1.59239	-1.09287	1.81486
O	-2.39333	-0.45907	2.69937
C	-1.90378	0.07328	3.98155
N	-1.6961	-2.84954	0.14893
C	-2.28419	-3.93737	-0.29568
C	-3.67715	-4.33851	0.08435
C	-4.04161	-4.49567	1.43536
C	-4.62645	-4.64569	-0.90734
C	-5.32001	-4.94108	1.78249
H	-3.31089	-4.28316	2.22091
C	-5.90891	-5.081	-0.56053
H	-4.35523	-4.54109	-1.96191
C	-6.26015	-5.23115	0.78596
H	-5.58112	-5.06905	2.83756
H	-6.63589	-5.30809	-1.34647
H	-7.26086	-5.58028	1.05867
C	-1.5401	-4.89153	-1.15649
C	-0.61811	-4.45034	-2.12717
C	-1.74464	-6.28237	-1.01922
C	0.08836	-5.36347	-2.9147
H	-0.48348	-3.38002	-2.29596
C	-1.03156	-7.19346	-1.80027
H	-2.45621	-6.65314	-0.27723
C	-0.11037	-6.73841	-2.75226
H	0.79857	-4.99243	-3.65935
H	-1.19392	-8.267	-1.66254
H	0.44499	-7.45293	-3.36752
Ag	0.58886	-2.5843	0.02529
C	-0.93563	1.24389	3.77097
H	0.00509	0.90754	3.31101
H	-1.39428	2.01106	3.1244
H	-0.70791	1.70786	4.74742
C	-1.27016	-1.06002	4.79782
H	-1.02529	-0.69306	5.80982
H	-1.97638	-1.90287	4.90036
H	-0.34296	-1.42656	4.33327
C	-3.18347	0.55638	4.67012
H	-3.91251	-0.26764	4.75707
H	-2.95097	0.92647	5.68317

H	-3.64985	1.37735	4.10247
C	-2.36146	-1.8717	0.85834
H	-3.41884	-1.99567	1.08027
C	-2.95594	0.06517	-0.66465
C	-2.15842	-0.46168	-1.79796
C	-2.78876	-1.17694	-2.84062
C	-0.7712	-0.22875	-1.91254
C	-2.0835	-1.56456	-3.98183
H	-3.85364	-1.41136	-2.78993
C	-0.06758	-0.61351	-3.05441
H	-0.22513	0.25215	-1.09768
C	-0.72318	-1.26839	-4.10449
H	-2.60658	-2.09626	-4.78202
H	1.00442	-0.41051	-3.11619
H	-0.17342	-1.55968	-5.00434
C	-4.47121	-0.10664	-0.87103
O	-5.05448	0.66841	-1.59403
O	-5.03278	-1.16426	-0.30271
C	-6.44223	-1.33226	-0.5179
H	-6.99121	-0.46514	-0.11709
H	-6.71598	-2.25078	0.01751
N	-2.54343	1.17328	-0.02199
C	-3.32945	1.8838	0.85949
O	-2.96688	2.96791	1.30476
O	-4.50497	1.32061	1.18307
C	-5.43133	2.05701	2.01636
H	-4.85106	2.76607	2.62254
H	-5.88525	1.29786	2.66867
C	-6.487	2.76839	1.2031
C	-6.18633	3.96187	0.52346
C	-7.7881	2.24952	1.11728
C	-7.16409	4.61324	-0.23361
H	-5.17782	4.37991	0.5928
C	-8.77044	2.90305	0.36408
H	-8.03847	1.32715	1.65187
C	-8.45883	4.0851	-0.31553
H	-6.91697	5.54169	-0.75781
H	-9.78127	2.48726	0.30927
H	-9.22482	4.59788	-0.90556

H	-6.65863	-1.43536	-1.59334

rev-TS-2R3S (Reverse mode)

M06-D3/def2-TZVPP/CPCM(THF)//B3LYP/def2-SV(P)/CPCM(THF)

Number of imaginary frequencies: 1 (84.4185i)

B3LYP/def2-SV(P)/CPCM(THF) free energy: -4840.89532576 (a.u.)

M06-D3/def2-TZVPP/CPCM(THF) single point energy: -4842.92421101 (a.u.)

Thermal correction to Gibbs Free Energy: 1.121795

C	3.73397	-0.87692	1.08504
C	4.69589	0.34462	0.88573
C	3.02367	1.59637	-0.57916
C	2.88231	2.23974	-1.94079
O	2.03284	1.45613	0.14374
C	1.83376	3.15956	-2.21596
C	1.73644	3.69142	-3.51346
C	2.62627	3.32453	-4.52818
C	3.63758	2.40253	-4.26297
C	3.75646	1.87043	-2.97734
P	0.50435	3.59354	-0.98747
S	4.60903	-2.44304	-0.92314
C	4.22885	-2.22932	-2.69718
O	5.96809	-1.85391	-0.74704
C	5.23198	-1.74848	-3.54378
C	4.94602	-1.67595	-4.9108
C	3.68698	-2.08012	-5.36159
C	2.76063	-2.54409	-4.42065
N	3.02925	-2.62168	-3.1141
O	4.52428	-3.9076	-0.70131
C	4.95171	1.13735	2.16328
C	3.94805	1.90018	2.78372
C	4.21357	2.60641	3.96171
C	5.48766	2.56481	4.54051
C	6.497	1.81521	3.92773
C	6.2279	1.11024	2.74929
C	4.15082	-1.77932	2.25195
C	5.42641	-2.36316	2.36064

C	5.74101	-3.2038	3.43328
C	4.78869	-3.48305	4.42056
C	3.51593	-2.9127	4.32363
C	3.20548	-2.0693	3.25026
C	1.33938	4.42917	0.42297
C	2.59316	5.05081	0.30084
C	3.14396	5.74663	1.38291
C	2.44781	5.83103	2.59338
C	1.201	5.20831	2.72345
C	0.6517	4.50606	1.64602
C	-0.40852	4.98877	-1.79854
C	-1.64082	4.70951	-2.41528
C	-2.37648	5.72496	-3.03625
C	-1.8929	7.03718	-3.03883
C	-0.67074	7.32844	-2.42097
C	0.06705	6.31269	-1.80575
H	2.76215	-0.45063	1.35891
H	5.65693	-0.06266	0.55325
H	0.94605	4.40574	-3.74885
H	2.51563	3.75815	-5.52635
H	4.3287	2.08732	-5.04964
H	4.52918	1.12081	-2.79118
H	6.1981	-1.45057	-3.13463
H	5.70055	-1.31	-5.61367
H	3.42273	-2.04253	-6.4216
H	1.76354	-2.87395	-4.73491
H	2.95535	1.9454	2.33031
H	3.41862	3.19656	4.42819
H	5.69322	3.11749	5.46273
H	7.49931	1.7784	4.36623
H	7.02436	0.52535	2.27809
H	6.17651	-2.17925	1.58915
H	6.74051	-3.64644	3.49628
H	5.0379	-4.14189	5.25852
H	2.75853	-3.11997	5.0863
H	2.20767	-1.62341	3.18969
H	3.14767	4.99793	-0.64044
H	4.12179	6.226	1.27644
H	2.87948	6.37724	3.43748

H	0.65395	5.26368	3.66939
H	-0.31976	4.01387	1.75887
H	-2.03383	3.687	-2.40868
H	-3.33332	5.48895	-3.51145
H	-2.46993	7.83453	-3.51712
H	-0.28915	8.35402	-2.41484
H	1.01737	6.55995	-1.32546
N	3.45209	-1.60067	-0.17276
N	4.2721	1.19742	-0.2406
H	5.02269	1.47278	-0.86239
Ag	-1.02687	1.77911	-0.47825
O	-0.29408	-3.34958	-1.4539
C	-1.49649	-3.38413	-1.14129
O	-2.37753	-4.12925	-1.81381
C	-2.03325	-5.09225	-2.87253
N	-1.34927	-2.11674	0.93082
C	-1.68913	-2.1369	2.18961
C	-0.93256	-1.31276	3.16895
C	-0.83966	-1.69072	4.52571
C	-0.30582	-0.11496	2.77008
C	-0.14839	-0.8997	5.4471
H	-1.30448	-2.62037	4.86256
C	0.36789	0.68426	3.69483
H	-0.37156	0.19167	1.72426
C	0.45091	0.29696	5.03798
H	-0.08164	-1.21911	6.49174
H	0.8238	1.62192	3.36545
H	0.97997	0.92512	5.76114
C	-2.79332	-2.99885	2.73387
C	-2.80577	-4.38501	2.48369
C	-3.81476	-2.4545	3.53349
C	-3.81353	-5.1982	3.00895
H	-2.0081	-4.83161	1.88345
C	-4.83189	-3.26483	4.04787
H	-3.81003	-1.38521	3.75791
C	-4.83492	-4.63953	3.78682
H	-3.79917	-6.27459	2.81214
H	-5.62203	-2.81992	4.66068
H	-5.62626	-5.27578	4.19495

Ag	1.47701	-2.38339	-0.59805
C	-1.17312	-6.21146	-2.27482
H	-1.00328	-6.99386	-3.03512
H	-0.19352	-5.83176	-1.94426
H	-1.68502	-6.67514	-1.41323
C	-1.34598	-4.40338	-4.05694
H	-1.29487	-5.10896	-4.90507
H	-1.91995	-3.51543	-4.36633
H	-0.32332	-4.08836	-3.80088
C	-3.40736	-5.62461	-3.29
H	-4.03451	-4.80474	-3.6805
H	-3.2973	-6.38951	-4.07735
H	-3.92617	-6.07942	-2.42856
C	-2.13168	-2.68118	-0.04143
H	-3.10679	-3.09911	0.21072
C	-3.15861	-0.63434	-0.96747
C	-4.6127	-1.0098	-1.17716
C	-5.27987	-0.35877	-2.23355
C	-5.34883	-1.90804	-0.3895
C	-6.63093	-0.60173	-2.4969
H	-4.73646	0.3512	-2.86223
C	-6.69664	-2.16451	-0.65974
H	-4.88305	-2.40203	0.4624
C	-7.34527	-1.51473	-1.71485
H	-7.12243	-0.07806	-3.32276
H	-7.24305	-2.87545	-0.03226
H	-8.40029	-1.71674	-1.92439
C	-2.32513	-0.83678	-2.20788
O	-2.71612	-1.4207	-3.19823
O	-1.1062	-0.26908	-2.14538
C	-0.2907	-0.36266	-3.3199
H	-0.10345	-1.41663	-3.57029
H	0.65103	0.14364	-3.06856
N	-2.74307	0.32933	-0.13186
C	-3.41605	0.84696	0.95387
O	-3.02242	1.86406	1.51341
O	-4.50167	0.17702	1.37249
C	-5.2861	0.77111	2.42942
H	-4.61259	1.11843	3.22625

H	-5.89075	-0.06841	2.80311
C	-6.17064	1.89443	1.93811
C	-7.21617	1.62729	1.03792
C	-5.96765	3.21324	2.37049
C	-8.04151	2.65875	0.58096
H	-7.38255	0.60241	0.69051
C	-6.79797	4.24644	1.9211
H	-5.14895	3.43249	3.06233
C	-7.8355	3.97154	1.02387
H	-8.85216	2.4371	-0.12027
H	-6.63001	5.27026	2.26969
H	-8.48407	4.77889	0.66945
H	-0.7844	0.13895	-4.16829

rev-TS-2R3R (Reverse mode)

M06-D3/def2-TZVPP/CPCM(THF)//B3LYP/def2-SV(P)/CPCM(THF)

Number of imaginary frequencies: 1 (77.6166i)

B3LYP/def2-SV(P)/CPCM(THF) free energy: -4840.90454234 (a.u.)

M06-D3/def2-TZVPP/CPCM(THF) single point energy: -4842.9270287 (a.u.)

Thermal correction to Gibbs Free Energy: 1.122258

C	-1.55443	2.8324	0.48877
C	-2.98127	3.07336	-0.09603
C	-3.2298	0.99652	-1.46142
C	-3.33422	0.42144	-2.84836
O	-3.20702	0.243	-0.48305
C	-3.09237	-0.9626	-3.05944
C	-3.16613	-1.45879	-4.37298
C	-3.49551	-0.63257	-5.45173
C	-3.76713	0.71884	-5.23512
C	-3.68514	1.23341	-3.94069
P	-2.6409	-2.12629	-1.67609
S	-0.25082	4.0238	-1.5728
C	0.57141	5.44882	-0.76387
O	0.74179	3.56779	-2.57484
C	0.06478	6.73833	-0.94612
C	0.77106	7.79742	-0.36753

C	1.9317	7.51901	0.35842
C	2.34374	6.18549	0.47199
N	1.67808	5.16987	-0.08376
O	-1.51685	4.59749	-2.12449
C	-4.14169	2.86571	0.87811
C	-4.11566	1.95854	1.94764
C	-5.21702	1.8205	2.79964
C	-6.37214	2.58214	2.59238
C	-6.41455	3.4882	1.52702
C	-5.30699	3.62778	0.68489
C	-1.24191	3.73721	1.685
C	-0.44725	3.22413	2.72546
C	-0.09952	4.00945	3.82845
C	-0.54586	5.33341	3.91585
C	-1.33895	5.85763	2.89038
C	-1.68294	5.0669	1.78687
C	-4.20747	-2.47337	-0.76788
C	-5.47283	-2.15007	-1.28254
C	-6.63084	-2.47224	-0.56588
C	-6.53764	-3.12432	0.66848
C	-5.27898	-3.44922	1.18695
C	-4.12046	-3.12184	0.47485
C	-2.34962	-3.73617	-2.55906
C	-1.03185	-4.07903	-2.91359
C	-0.76318	-5.28093	-3.57833
C	-1.80417	-6.16277	-3.88727
C	-3.11721	-5.8363	-3.52951
C	-3.38865	-4.63231	-2.87139
H	-1.53409	1.80205	0.86581
H	-3.03139	4.11951	-0.41694
H	-2.96988	-2.51397	-4.56706
H	-3.54998	-1.05569	-6.45905
H	-4.04607	1.37277	-6.06611
H	-3.92281	2.28742	-3.781
H	-0.84883	6.894	-1.52109
H	0.41656	8.82629	-0.48236
H	2.51243	8.31661	0.82945
H	3.24966	5.92795	1.0337
H	-3.23204	1.342	2.1199

H	-5.17073	1.10885	3.63034
H	-7.23323	2.4721	3.25907
H	-7.30944	4.09449	1.35433
H	-5.34658	4.34544	-0.14169
H	-0.08461	2.19327	2.66182
H	0.52057	3.58348	4.62371
H	-0.27982	5.95126	4.77931
H	-1.69762	6.89033	2.94726
H	-2.30809	5.50708	1.00575
H	-5.56292	-1.64085	-2.24591
H	-7.61098	-2.21141	-0.97704
H	-7.44425	-3.37528	1.22756
H	-5.19421	-3.95245	2.1548
H	-3.14022	-3.36776	0.89683
H	-0.20116	-3.41399	-2.65968
H	0.26795	-5.53114	-3.84629
H	-1.59266	-7.10595	-4.40056
H	-3.93733	-6.52258	-3.76239
H	-4.42056	-4.39348	-2.60139
N	-0.48883	2.81983	-0.53924
N	-3.17502	2.34395	-1.35465
H	-2.89242	2.88955	-2.1653
Ag	-0.66919	-1.63354	-0.30261
O	2.2475	1.0803	1.60285
C	3.27856	0.44087	1.41705
O	4.29846	0.39184	2.30057
C	4.41675	1.28454	3.46491
N	2.9616	0.03803	-0.9534
C	3.51085	-0.121	-2.13627
C	4.93963	-0.52924	-2.33112
C	5.26096	-1.62328	-3.15548
C	5.98921	0.213	-1.75574
C	6.5935	-1.98048	-3.38001
H	4.45609	-2.20511	-3.61251
C	7.32196	-0.13595	-1.99253
H	5.75957	1.08054	-1.13039
C	7.62851	-1.23691	-2.80077
H	6.82455	-2.84121	-4.01519
H	8.12492	0.4588	-1.54641

H	8.67202	-1.51143	-2.98319
C	2.74274	0.18603	-3.37043
C	3.37677	0.84053	-4.44823
C	1.3839	-0.16398	-3.50959
C	2.66841	1.1649	-5.60701
H	4.43106	1.11763	-4.36742
C	0.68005	0.15008	-4.67529
H	0.89502	-0.72548	-2.71186
C	1.31645	0.82079	-5.72583
H	3.17559	1.69039	-6.42201
H	-0.37056	-0.14214	-4.76637
H	0.76364	1.06661	-6.63771
Ag	1.06262	1.27846	-0.6953
C	5.7378	0.83903	4.10001
H	5.6719	-0.20691	4.44728
H	6.56413	0.91434	3.37226
H	5.97619	1.4776	4.96765
C	3.25831	1.09164	4.44911
H	3.11853	0.02439	4.68434
H	3.48794	1.63167	5.38508
H	2.31416	1.4784	4.03935
C	4.5177	2.73364	2.97324
H	3.58619	3.05603	2.48169
H	4.70713	3.40377	3.83006
H	5.35341	2.84301	2.25965
C	3.57752	-0.34196	0.21979
H	4.58191	-0.76721	0.17382
C	2.15034	-2.40772	1.01844
C	2.88005	-3.22	2.06215
C	2.35286	-4.50344	2.2986
C	4.01485	-2.81253	2.77564
C	2.95758	-5.36524	3.2199
H	1.46466	-4.83632	1.75235
C	4.61648	-3.67433	3.69681
H	4.42418	-1.8141	2.62103
C	4.09441	-4.95345	3.92208
H	2.53537	-6.36106	3.3863
H	5.50134	-3.33907	4.24695
H	4.57039	-5.62499	4.6432

C	2.36293	-2.76756	-0.43362
O	1.50738	-2.52512	-1.27002
O	3.45401	-3.39772	-0.88532
C	4.66943	-3.75109	-0.22212
H	5.09855	-2.90114	0.32538
H	5.3548	-4.04405	-1.0316
N	0.96787	-1.85302	1.27552
C	0.60169	-1.52936	2.57637
O	1.29354	-1.53075	3.57424
O	-0.70924	-1.18022	2.59172
C	-1.28578	-0.79803	3.86085
H	-2.03997	-0.04106	3.60385
H	-0.49884	-0.33604	4.47346
C	-1.92664	-1.96401	4.57868
C	-1.15958	-2.83444	5.37301
C	-3.3064	-2.19545	4.45985
C	-1.76057	-3.91324	6.02884
H	-0.08444	-2.66175	5.46981
C	-3.91034	-3.2727	5.11829
H	-3.91654	-1.52294	3.84777
C	-3.13747	-4.13515	5.90348
H	-1.15176	-4.58185	6.64557
H	-4.98823	-3.43564	5.02052
H	-3.60789	-4.97716	6.42087
H	4.51603	-4.59962	0.46107

rev-TS-2S3S (Reverse mode)

M06-D3/def2-TZVPP/CPCM(THF)//B3LYP/def2-SV(P)/CPCM(THF)

Number of imaginary frequencies: 1 (114.7051i)

B3LYP/def2-SV(P)/CPCM(THF) free energy: -4840.89927118 (a.u.)

M06-D3/def2-TZVPP/CPCM(THF) single point energy: -4842.91104708 (a.u.)

Thermal correction to Gibbs Free Energy: 1.119324

C	2.93389	-2.52944	-0.41833
C	4.44585	-2.2201	-0.15353
C	4.3976	0.27989	-0.03041
C	4.84369	1.56668	-0.67253

O	3.68205	0.31399	0.97627
C	4.21371	2.78934	-0.31572
C	4.6336	3.96866	-0.95543
C	5.66426	3.96254	-1.89991
C	6.30523	2.76569	-2.2215
C	5.89238	1.58217	-1.60814
P	2.83896	2.8825	0.93642
S	3.07686	-2.30166	-3.11448
C	2.62048	-0.83816	-4.10897
O	4.57326	-2.31808	-3.09152
C	3.60416	-0.22241	-4.88583
C	3.21107	0.84414	-5.70059
C	1.87134	1.24015	-5.70187
C	0.97102	0.54987	-4.88306
N	1.34133	-0.47272	-4.10567
O	2.47181	-3.44978	-3.83742
C	4.92909	-2.5288	1.26502
C	4.1502	-2.34373	2.41848
C	4.66568	-2.64033	3.68519
C	5.97187	-3.12192	3.82514
C	6.75975	-3.31025	2.68469
C	6.23744	-3.01898	1.42052
C	2.64968	-4.02057	-0.22891
C	3.29349	-5.00701	-0.99681
C	3.01624	-6.3631	-0.79869
C	2.09062	-6.76122	0.17387
C	1.44148	-5.79003	0.9428
C	1.72132	-4.43326	0.74012
C	3.63588	2.73588	2.59138
C	5.01638	2.90327	2.78295
C	5.56758	2.81138	4.06595
C	4.74635	2.55574	5.16939
C	3.36909	2.38926	4.98495
C	2.81712	2.47575	3.70291
C	2.33583	4.66707	0.89209
C	1.27097	5.03951	0.05245
C	0.84934	6.37135	-0.01696
C	1.4775	7.34804	0.76344
C	2.53074	6.98671	1.611

C	2.95861	5.65641	1.67457
H	2.35579	-1.99261	0.34372
H	5.02714	-2.86684	-0.82007
H	4.15685	4.91893	-0.71102
H	5.97005	4.90085	-2.37189
H	7.12749	2.74877	-2.94239
H	6.41948	0.65661	-1.85003
H	4.63633	-0.57255	-4.84607
H	3.94603	1.35891	-6.32649
H	1.52367	2.06789	-6.32515
H	-0.08872	0.82675	-4.85389
H	3.13329	-1.95686	2.3385
H	4.03963	-2.48968	4.57043
H	6.37222	-3.35211	4.81759
H	7.78166	-3.69163	2.77686
H	6.85936	-3.17705	0.53282
H	4.00553	-4.71964	-1.77501
H	3.52695	-7.11452	-1.40945
H	1.87639	-7.82338	0.32909
H	0.71444	-6.08705	1.70548
H	1.21047	-3.67617	1.34433
H	5.67194	3.10223	1.93076
H	6.64585	2.9402	4.20145
H	5.17957	2.48243	6.17158
H	2.71968	2.18368	5.84128
H	1.73972	2.33126	3.56983
H	0.75789	4.28407	-0.55213
H	0.01897	6.64088	-0.67621
H	1.14256	8.38882	0.71621
H	3.02351	7.74366	2.22893
H	3.78364	5.39203	2.341
N	2.42456	-1.95971	-1.68482
N	4.8236	-0.87251	-0.59387
H	5.14082	-0.85718	-1.55992
Ag	0.89542	1.44435	0.63588
O	-1.87993	-1.16136	-1.90948
C	-2.94958	-1.33154	-1.29829
O	-3.44124	-2.53538	-0.99099
C	-3.08016	-3.80352	-1.64632

N	-5.01734	-0.475	-0.37667
C	-6.10157	0.21347	-0.56967
C	-6.21441	1.31418	-1.58741
C	-6.50593	2.63832	-1.21482
C	-6.03532	1.02636	-2.95326
C	-6.60954	3.64708	-2.17811
H	-6.64868	2.88315	-0.15866
C	-6.1461	2.03218	-3.91852
H	-5.81786	-0.00063	-3.26188
C	-6.43167	3.34699	-3.5335
H	-6.83189	4.67281	-1.86759
H	-6.01053	1.78657	-4.97649
H	-6.51739	4.13533	-4.2878
C	-7.30746	-0.15831	0.21616
C	-7.19736	-1.0821	1.28039
C	-8.58602	0.36098	-0.07647
C	-8.31471	-1.46402	2.02046
H	-6.2104	-1.49279	1.50472
C	-9.70685	-0.02308	0.66709
H	-8.71123	1.06636	-0.90106
C	-9.57856	-0.93452	1.71985
H	-8.20291	-2.1797	2.8414
H	-10.6883	0.39234	0.41718
H	-10.4555	-1.23279	2.30282
Ag	0.28761	-1.5318	-1.8912
C	-1.75853	-4.34392	-1.09599
H	-0.90504	-3.69398	-1.35175
H	-1.80049	-4.44442	0.00047
H	-1.56072	-5.34267	-1.52279
C	-3.04443	-3.63761	-3.17063
H	-2.95816	-4.63307	-3.63965
H	-3.97795	-3.17038	-3.53085
H	-2.19138	-3.02563	-3.50036
C	-4.23777	-4.72342	-1.24194
H	-5.19676	-4.32416	-1.61421
H	-4.08865	-5.7328	-1.66163
H	-4.30062	-4.80789	-0.14328
C	-3.80518	-0.20491	-0.94113
H	-3.6719	0.6517	-1.60914

C	-2.2407	1.10231	0.52218
C	-2.1825	2.27602	-0.4023
C	-2.78073	3.51728	-0.10602
C	-1.44529	2.17027	-1.60271
C	-2.65435	4.60336	-0.97831
H	-3.34908	3.65012	0.81409
C	-1.329	3.25201	-2.47621
H	-0.98794	1.21371	-1.85981
C	-1.93441	4.4776	-2.16977
H	-3.12424	5.55663	-0.71777
H	-0.76303	3.13652	-3.40569
H	-1.84402	5.32634	-2.85449
C	-3.14194	1.18744	1.74117
O	-2.75609	0.9172	2.85851
O	-4.36581	1.66108	1.49249
C	-5.21837	1.84303	2.62871
H	-5.41759	0.87647	3.11816
H	-6.15307	2.26952	2.24018
N	-1.07885	0.42368	0.67332
C	-0.96657	-0.72485	1.42809
O	0.10008	-1.09435	1.90653
O	-2.0989	-1.44095	1.51745
C	-2.09947	-2.65461	2.2959
H	-1.12984	-3.15755	2.17177
H	-2.89006	-3.25995	1.83135
C	-2.4064	-2.4178	3.75688
C	-3.73879	-2.35549	4.19624
C	-1.37549	-2.26076	4.69723
C	-4.0377	-2.13606	5.54456
H	-4.55136	-2.485	3.47319
C	-1.67086	-2.04248	6.04713
H	-0.33507	-2.3052	4.36389
C	-3.00237	-1.97817	6.47365
H	-5.08113	-2.09281	5.8722
H	-0.85738	-1.9244	6.7699
H	-3.23351	-1.80962	7.53021
H	-4.7526	2.52967	3.35437

3.2.5. Intermidiate-5 (Int-5)

The reaction path from **TS-2S3R** to **Int-5** was explored by the MC-AFIR method implemented in the GRRM program. In the initial search, electronic structure calculations were carried out at the GFN2-xTB method using the Orca program. The artificial force was applied between the C(120) atom of the Schiff base moiety and C(122) atoms of the ketimine moiety, the Ag(78) atoms, and N(140) atom of the ketimine moiety. The collision energy parameter (γ) of the AFIR method was set to 400.0 kJ/mol. The last integer in each Cartesian coordinate represents a random distribution of each molecule; in this case, the catalyst (ligand + 2Ag) was defined as part #1, the Schiff base is part #2, and the ketiminoester is part #3. All obtained 10 equilibrium (EQ) structures were re-optimized at the B3LYP/def2-SV(P)/CPCM(THF). Subsequently, single-point energy calculations for the optimized geometry were performed using M06-D3 functional with def2-TZVPP basis set for all atoms in CPCM solvation model (THF).

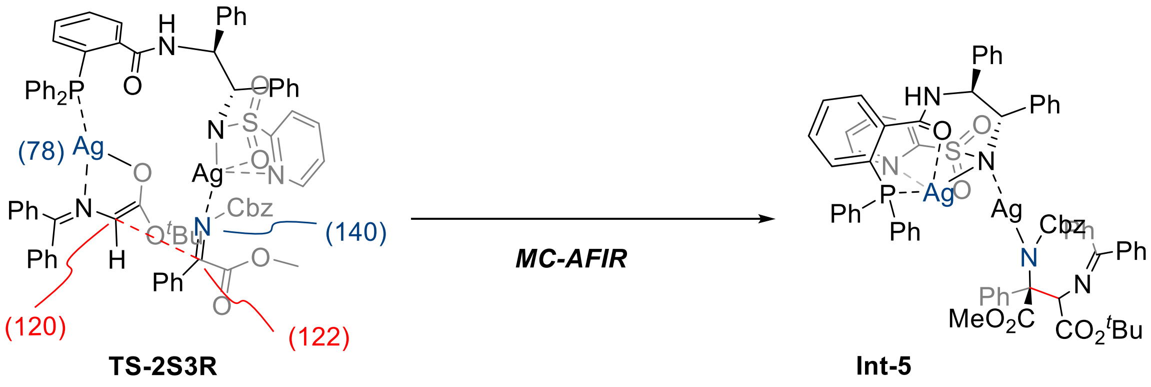

Scheme S6. MC-AFIR search of **Int-5**.

Calculated Structures

Int-5

M06-D3/def2-TZVPP/CPCM(THF)//B3LYP/def2-SV(P)/CPCM(THF)

B3LYP/def2-SV(P)/CPCM(THF) free energy: -4840.93439240 (a.u.)

M06-D3/def2-TZVPP/CPCM(THF) single point energy: -4842.98072578 (a.u.)

Thermal correction to Gibbs Free Energy: 1.123631

C	1.83362	2.2163	0.87232
C	3.32282	2.36092	1.31829
C	4.28078	0.52046	-0.13797
C	5.32637	-0.57253	-0.16996
O	3.63899	0.7956	-1.14926

C	5.00307	-1.88362	-0.60116
C	6.03268	-2.83309	-0.6919
C	7.35609	-2.49796	-0.38149
C	7.66737	-1.20621	0.04559
C	6.65132	-0.2511	0.15581
P	3.23021	-2.36302	-0.88285
S	0.8365	0.92527	3.06088
C	2.21862	-0.00728	3.81964
O	0.82942	2.22283	3.76748
C	2.74213	0.40861	5.04354
C	3.72211	-0.39606	5.63517
C	4.12513	-1.56403	4.98401
C	3.53691	-1.88458	3.75613
N	2.59882	-1.11679	3.18893
O	-0.32369	0.02719	3.25107
C	4.04674	3.57954	0.74011
C	3.94584	3.95041	-0.61166
C	4.66792	5.04244	-1.10432
C	5.50425	5.78066	-0.25921
C	5.61369	5.41901	1.08751
C	4.88945	4.32775	1.57881
C	1.03305	3.52112	0.98247
C	1.24786	4.49852	1.96729
C	0.47208	5.66145	2.00811
C	-0.54147	5.87095	1.06616
C	-0.76602	4.9078	0.07745
C	0.01998	3.7506	0.03626
C	2.86472	-2.02647	-2.65177
C	3.86468	-1.78008	-3.60561
C	3.52021	-1.55106	-4.94269
C	2.17812	-1.56953	-5.33795
C	1.1767	-1.8104	-4.38971
C	1.51683	-2.03126	-3.05195
C	3.22737	-4.20347	-0.76528
C	2.98232	-4.79515	0.48676
C	2.98046	-6.18612	0.6282
C	3.20942	-7.00366	-0.48439
C	3.44111	-6.42452	-1.73688
C	3.45088	-5.03271	-1.87862

H	1.86567	1.96506	-0.19391
H	3.33843	2.4661	2.41277
H	5.80427	-3.85533	-1.00237
H	8.14044	-3.25533	-0.47001
H	8.69818	-0.93735	0.29387
H	6.89453	0.7649	0.48095
H	2.38702	1.32932	5.50835
H	4.16293	-0.11137	6.59492
H	4.88633	-2.22043	5.4124
H	3.83184	-2.78462	3.20773
H	3.30425	3.38656	-1.29139
H	4.57132	5.31852	-2.15908
H	6.0656	6.63585	-0.64823
H	6.26083	5.9897	1.76092
H	4.97749	4.05613	2.63637
H	2.01987	4.35874	2.72511
H	0.6607	6.40827	2.786
H	-1.14874	6.78096	1.10086
H	-1.54859	5.05805	-0.6728
H	-0.16062	3.01347	-0.75296
H	4.91678	-1.76027	-3.30907
H	4.30689	-1.35762	-5.6783
H	1.91159	-1.38925	-6.38374
H	0.12358	-1.81562	-4.68617
H	0.72281	-2.20166	-2.31738
H	2.7795	-4.16515	1.35878
H	2.78845	-6.63154	1.60886
H	3.19984	-8.09251	-0.37659
H	3.61434	-7.05829	-2.61184
H	3.63131	-4.59478	-2.86391
N	1.12325	1.03086	1.45193
N	4.11927	1.14116	1.06091
H	4.71506	0.82977	1.82068
Ag	-0.60352	0.32058	0.23875
O	-6.6707	-1.25723	0.32251
C	-5.54239	-1.69661	0.33541
O	-5.20993	-2.94386	0.6703
C	-6.21302	-3.97612	0.99251
N	-4.74536	0.43886	-0.46669

C	-4.89621	1.50215	0.22743
C	-4.65351	1.59894	1.71338
C	-3.48015	2.20067	2.19956
C	-5.58751	1.08704	2.6295
C	-3.23714	2.27634	3.57511
H	-2.74458	2.60419	1.49636
C	-5.3518	1.1789	4.00612
H	-6.50076	0.6145	2.25772
C	-4.17566	1.76973	4.48158
H	-2.30402	2.71967	3.93489
H	-6.08967	0.78113	4.70999
H	-3.98864	1.83233	5.55817
C	-5.33391	2.74801	-0.48142
C	-5.77449	3.88242	0.22385
C	-5.32391	2.78996	-1.89108
C	-6.20169	5.02741	-0.45858
H	-5.79319	3.87156	1.31637
C	-5.74402	3.93471	-2.56915
H	-4.96483	1.91242	-2.43422
C	-6.18733	5.05816	-1.8561
H	-6.54717	5.89827	0.10723
H	-5.72481	3.95497	-3.66359
H	-6.51814	5.95439	-2.3905
Ag	1.92004	-1.1861	0.8213
C	-7.1349	-4.20663	-0.21016
H	-7.77063	-3.32961	-0.40183
H	-6.53873	-4.41122	-1.11548
H	-7.7837	-5.07837	-0.0134
C	-6.98766	-3.5748	2.25319
H	-7.63987	-4.40929	2.56602
H	-6.28909	-3.35764	3.08058
H	-7.61325	-2.68776	2.07497
C	-5.35982	-5.21794	1.26166
H	-4.65917	-5.0366	2.09471
H	-6.00816	-6.07027	1.52828
H	-4.77097	-5.48826	0.36916
C	-4.30183	-0.80534	0.1083
H	-3.88228	-0.66104	1.11966
C	-3.12467	-1.42984	-0.76613

C	-2.39655	-2.53602	0.05197
C	-2.1702	-3.83737	-0.42889
C	-1.90928	-2.23597	1.33754
C	-1.48345	-4.78973	0.33394
H	-2.52584	-4.13411	-1.41428
C	-1.21971	-3.18172	2.10409
H	-2.05969	-1.24832	1.77756
C	-1.0016	-4.46993	1.60583
H	-1.32894	-5.79251	-0.07696
H	-0.8587	-2.89801	3.09688
H	-0.46632	-5.21545	2.20211
C	-3.73967	-2.04784	-2.05125
O	-4.92245	-2.23676	-2.2211
O	-2.80538	-2.45782	-2.92162
C	-3.2782	-2.93132	-4.1821
H	-3.8223	-2.12666	-4.70342
H	-3.94431	-3.80073	-4.0546
N	-2.15207	-0.37507	-1.09201
C	-2.35216	0.33933	-2.21024
O	-3.22254	0.21335	-3.07243
O	-1.38448	1.32256	-2.35908
C	-1.5272	2.1756	-3.49506
H	-2.33217	2.90971	-3.31088
H	-1.84127	1.56489	-4.35882
C	-0.22653	2.88463	-3.78819
C	-0.22277	4.25548	-4.08809
C	0.99009	2.18316	-3.8263
C	0.96569	4.91275	-4.42839
H	-1.16207	4.81758	-4.05469
C	2.18022	2.83843	-4.15692
H	1.00509	1.11461	-3.59336
C	2.17191	4.20565	-4.46306
H	0.94821	5.98248	-4.65958
H	3.11917	2.27661	-4.17507
H	3.10342	4.71688	-4.72614
H	-2.38416	-3.21842	-4.75435

4. Optimization of the reaction conditions

4.1 Table S4: Screening of the protecting group

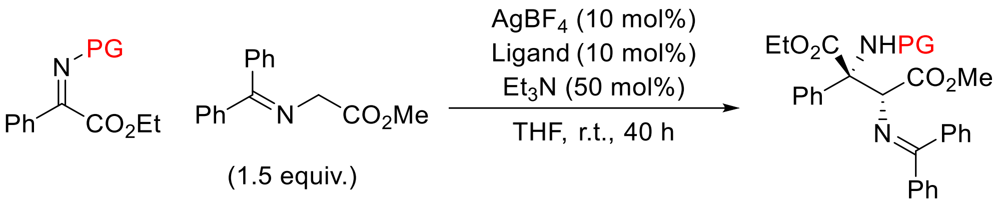

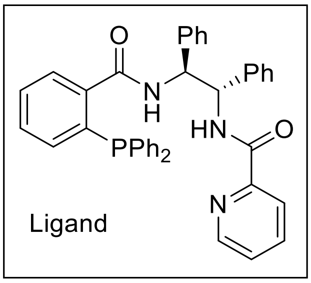

Entry[a]	PG	Yield (%)	Dr	Er (major)
1	Cbz	95	58:42	61:39
2	Boc	trace	-	53:47
3	Ts	34	64:36	52:48
4	Dpp	49	57:43	51:49

a) Determined by ^{1}H NMR.

4.2 Table S5: Screening of the metal salts

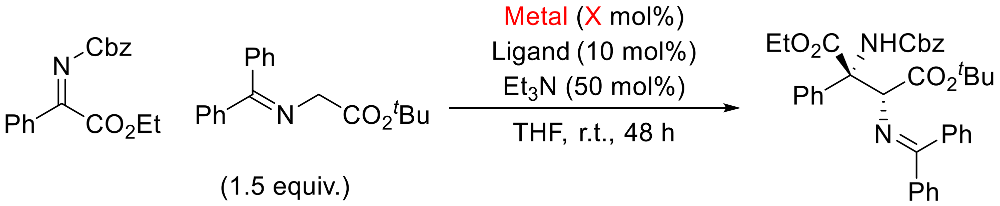

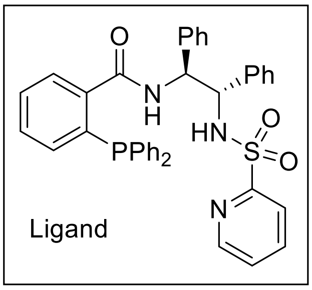

Entry[a]	Metal	X	Yield (%)	Dr	Er (major)
1	$AgBF_4$	10	37	99:1	85:15
2	Ag(acac)	10	trace	-	-
3	Ag_2CO_3	10	trace	-	-
4	AgOAc	10	32	99:1	58:42
5	$AgOCOCF_3$	10	trace	-	-
6	CuBr	10	15	83:17	52:48
7	$AgBF_4$	20	26	85:15	88:12
8	AgOAc	20	35	87:13	89:11

a) Determined by ^{1}H NMR.

4.3 Table S6: Screening of the bases

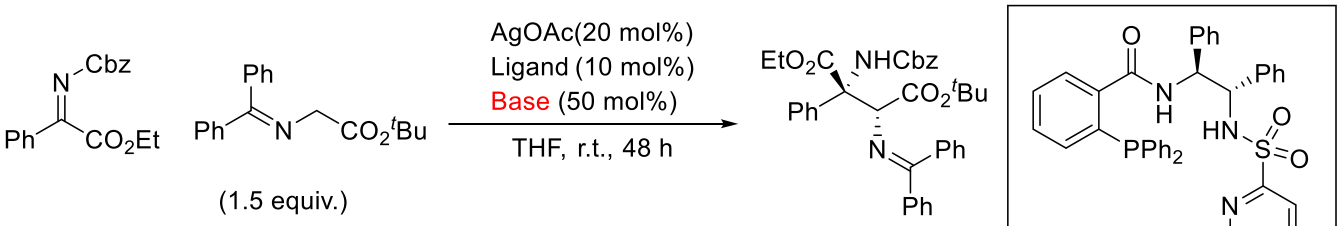

Entry[a]	Base	Yield (%)	Dr	Er (major)
1	Et_3N	35	87:13	89:11
2	DABCO	38	75:25	47:53
3	DBU	64	77:23	67:33
4	Cs_2CO_3	84	99:1	87:13
5	K_2CO_3	67	88:12	96:4
6[b]	Cs_2CO_3	81	99:1	97:3
7[b]	K_2CO_3	76	99:1	97:3

a) Determined by ^{1}H NMR.
b) Ketimine **1a** (1.1 equiv.) and Nucleophile (1.0 equiv.) were used at 0 °C.

4.4 Table S7: Reaction temperature

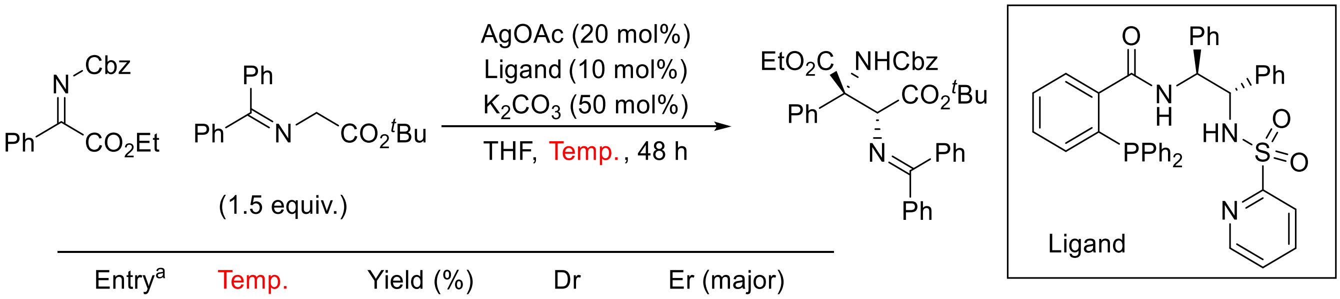

Entry[a]	Temp.	Yield (%)	Dr	Er (major)
1	0 °C	91	97:3	97:3
2	r.t.	67	88:12	96:4
3	-20 °C	61	91:9	86:14

a) Determined by ^{1}H NMR.

4.5 Table S8: Screening of the base equivalent

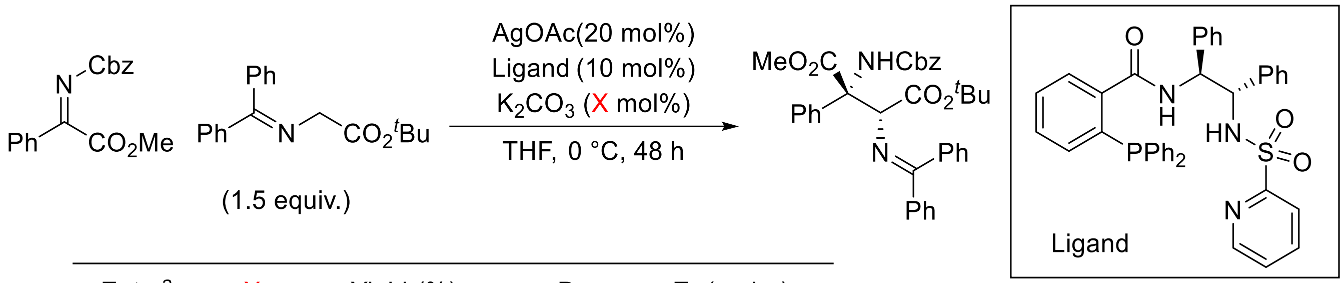

Entry[a]	X	Yield (%)	Dr	Er (major)
1	50	91	97:3	97:3
2	100	69	98:2	97:3
3	20	60	97:3	97:3

a) Determined by ^{1}H NMR.

4.6 Table S9: Screening of the additives

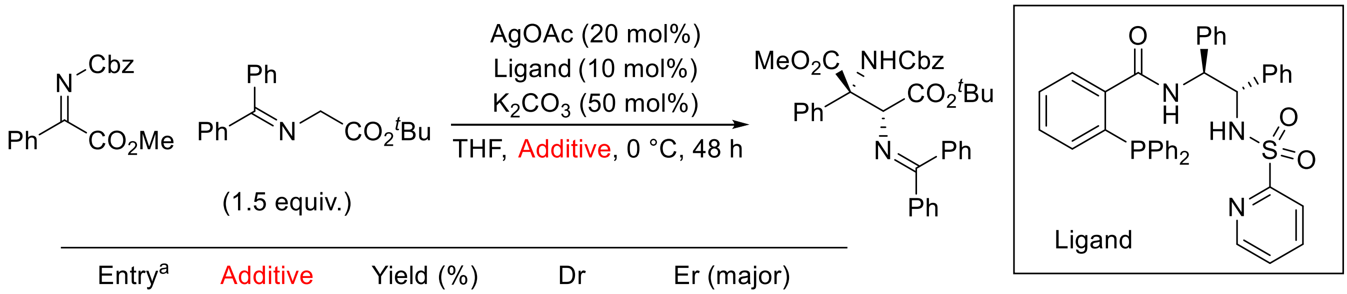

Entry[a]	Additive	Yield (%)	Dr	Er (major)
1	-	91	97:3	97:3
2	MS4A	95	99:1	97:3
3	MS3A	70	99:1	97:3
4	MS5A	65	99:1	97:3

a) Determined by ^{1}H NMR.

4.7 Table S10: Screening of the solvent

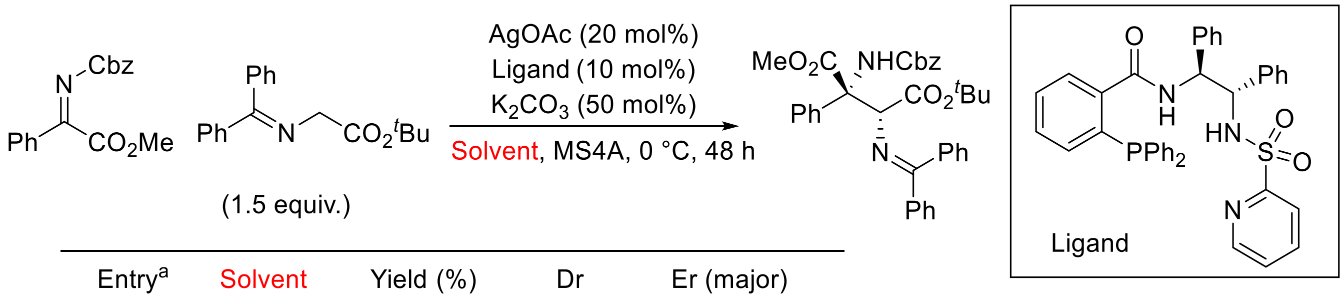

Entry[a]	Solvent	Yield (%)	Dr	Er (major)
1	THF	95	99:1	97:3
2	Toluene	99	99:1	79:21
3	CH_2Cl_2	64	99:1	81:19
4	Et_2O	99	99:1	89:11

a) Determined by ^{1}H NMR.

4.8 Table S11: Screening of equivalent of nucleophile

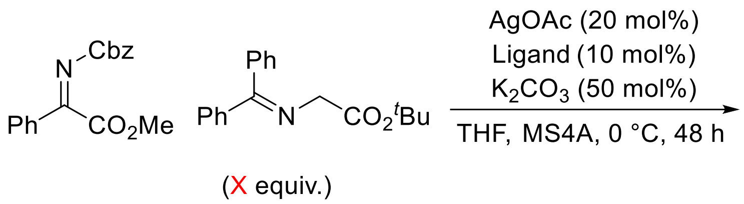

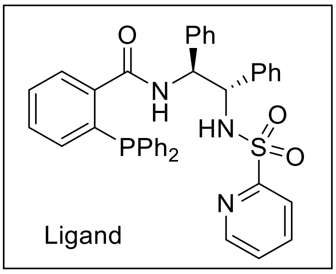

Entry[a]	X	Yield (%)	Dr	Er (major)
1	1.5	95	99:1	97:3
2	1.2	99	99:1	97:3
3	1.0	99	98:2	97:3

a) Determined by ^{1}H NMR.

4.9 Table S12: Screening of catalyst loading

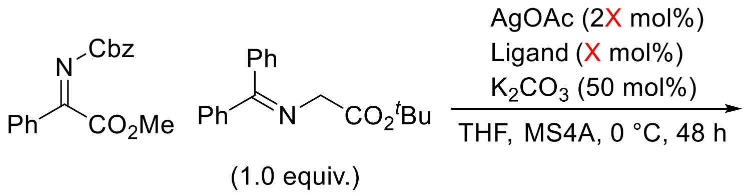

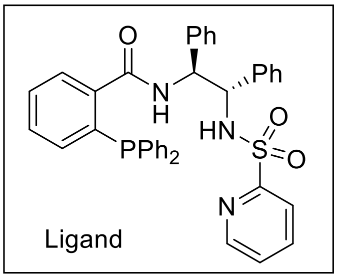

Entry	X	Yield (%)	Dr	Er (major)
1	10	99	98:2	97:3
2	5	99	99:1	98:2
3	1	36	99:1	95:5

a) Determined by ^{1}H NMR.

4.10 Scheme S13: Retro reaction

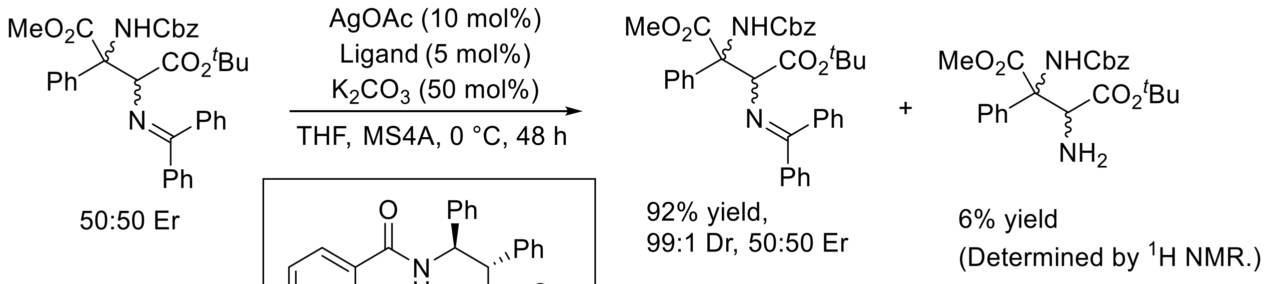

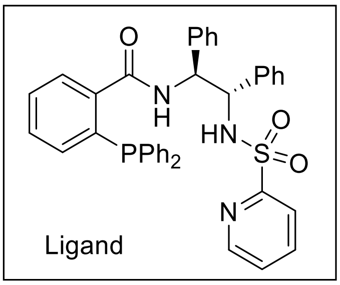

5 Limitations of substrates

5.1. Scheme S14: Limitations of nucleophiles

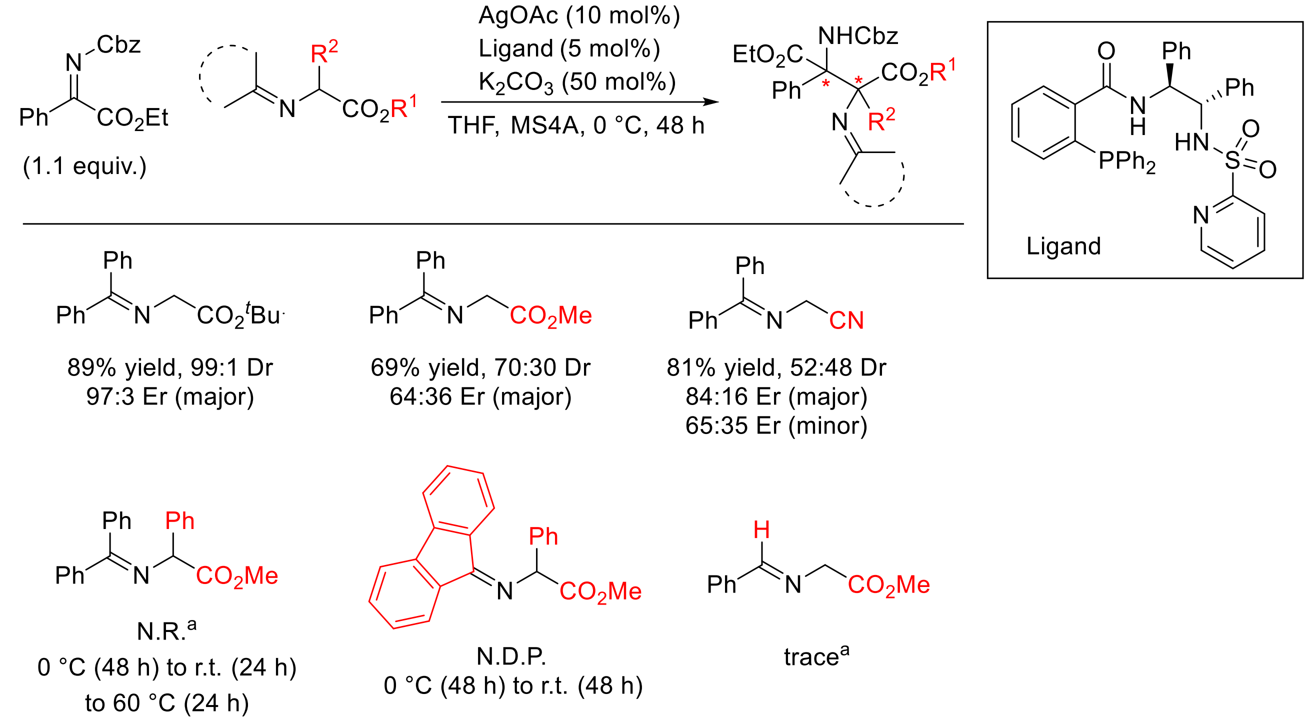

a) AgOAc (20 mol%) and Ligand (10 mol%) were used.

When the substrate was replaced from the glycinate-derived Schiff base to imines derived from benzaldehyde, neither the corresponding addition products nor the cyclized products were obtained.

5.2. Scheme S15: Limitations of electrophiles

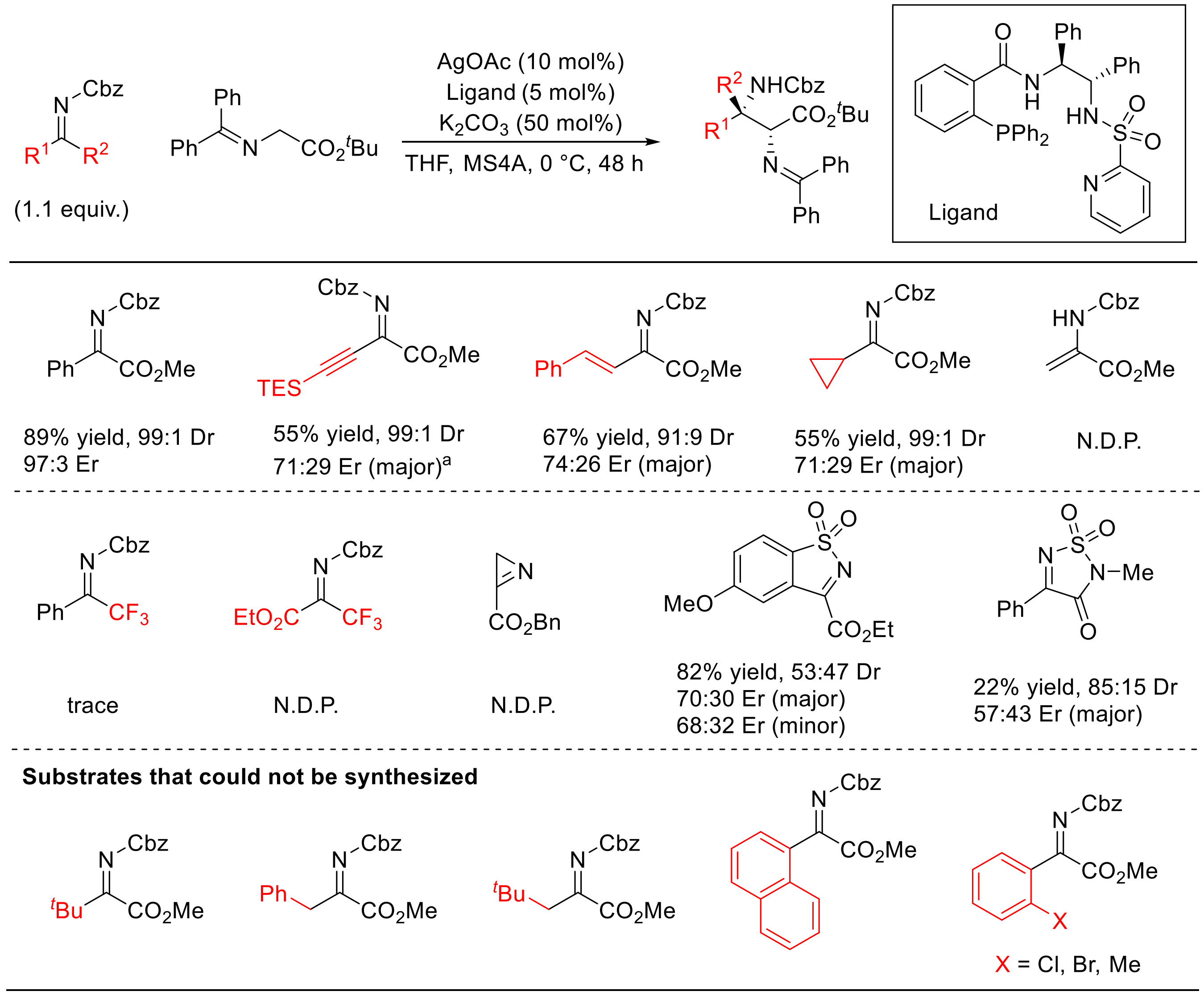

a) -20 °C.

Attempts were made to prepare the α-ketiminoesters shown in the lower part of Scheme S15 according to the reported procedure (ref. 1b); however, the synthesis was unsuccessful.

6 General procedure for preparation of Cbz-ketimines

α-Ketiminoesters were prepared by the reported methods.[1b] The spectroscopic data were consistent with literature values.[12] *α*-Iminonitrile **1s** was synthesized by the published procedure.[13]

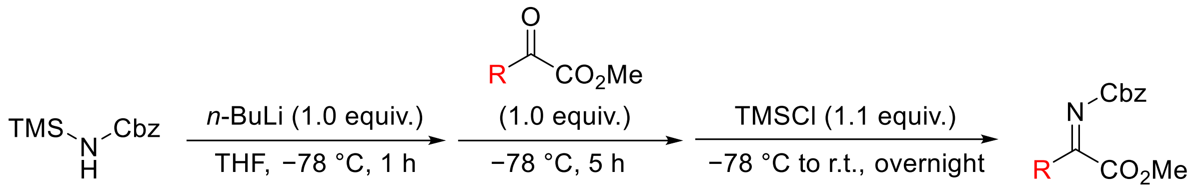

A 1.3 M solution of butyllithium in hexane (1.0 equiv.) was added to a stirred solution of *tert*-butyl(trimethylsilyl)benzamide (1.0 equiv.) in THF (0.1 M) at −78 °C. The mixture was stirred for 1 h, and a solution of α-ketoester (1.0 equiv.) in THF (1.0 M) was added dropwise. After stirring for 5 h, trimethylsilyl chloride (1.1 equiv.) was added. The reaction mixture was gradually warmed to room temperature and stirred for overnight, then poured into aqueous $NaHCO_3$ and extracted with EtOAc. The combined organic layers were dried over Na_2SO_4, filtered, and concentrated under reduced pressure. The residue was purified by silica gel column chromatography to afford the corresponding *N*-Cbz ketiminoesters.

Methyl 2-[(benzoyloxycarbonyl)imino]-2-(4-idophenyl)acetate (1e)

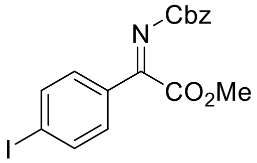

According to the typical procedure, the reaction with methyl 4-ido-1-oxobenzeneacetate (0.87 g, 3.0 mmol) gave **1e** (0.30 g, 24% yield) as a yellow oil. **^{1}H NMR** (400 MHz, $CDCl_3$) δ 7.80–7.78 (m, 2H), 7.59–7.57 (m, 2H), 7.44–7.33 (m, 5H), 5.28 (s, 2H), 3.67 (s, 3H); **^{13}C NMR** (176 MHz, $CDCl_3$) δ 162.32, 161.88, 161.41, 138.13, 135.18, 131.51, 130.73, 129.00, 128.78, 128.74, 128.72, 68.99, 53.07; **IR** (ATR) 3026, 2954, 1739, 1579, 1557, 1201, 1174, 1001, 844, 696 cm^{-1}; **HRMS** (ESI): m/z calcd. for $C_{17}H_{14}NO_4NaI$ $[M+Na]^+$: 445.9860; found 445.9876.

Methyl 2-[(benzoyloxycarbonyl)imino]-2-(2-fluorophenyl)acetate (1g)

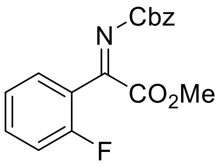

According to the typical procedure, the reaction with methyl 2-fluoro-1-oxobenzeneacetate (1.4 g, 7.5 mmol) gave **1g** (0.98 g, 41% yield) as a white solid. **^{1}H NMR** (300 MHz, $CDCl_3$) δ 7.81–7.78 (m, 1H), 7.54–7.51 (m, 1H), 7.38 (s, 4H), 7.26–7.22 (m, 2H), 7.14–7.08 (m, 1H), 5.29 (s, 2H), 3.72 (s, 3H); **^{13}C NMR** (125 MHz, $CDCl_3$) δ 162.06, 161.02, 159.00, 135.01, 134.69, 130.48, 128.83, 128.66, 128.63, 124.73, 121.65, 116.23, 116.06, 68.95, 53.24; **^{19}F NMR** (282 MHz, $CDCl_3$) δ −111.54; **IR** (ATR) 3069, 3035, 1747, 1631, 1436, 1266, 1213, 1025, 762, 698 cm^{-1}; **HRMS** (ESI): m/z calcd. for $C_{17}H_{14}NO_4NaF$ $[M+Na]^+$: 338.0800; found

338.0803.

Methyl 2-{[(benzyloxy)carbonyl]imino}-2-naphthylacetate (1j)

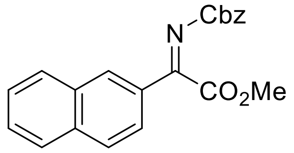

According to the typical procedure, the reaction with methyl 2-(naphthalen-2-yl)-2-oxoacetate (2.6 g, 12 mmol) gave **1j** (1.8 g, 50% yield) as a white solid. **^{1}H NMR** (300 MHz, $CDCl_3$) δ 8.33–8.30 (m, 1H), 8.03–8.00 (m, 1H), 7.91–7.85 (m, 3H), 7.63–7.52 (m, 2H), 7.45–7.37 (m, 5H), 5.31 (s, 2H), 3.73 (s, 3H); **^{13}C NMR** (125 MHz, $CDCl_3$) δ 163.24, 163.09, 161.70, 135.64, 135.26, 132.46, 132.18, 129.55, 129.29, 128.91, 128.87, 128.76, 128.64, 128.28, 127.86, 126.99, 124.12, 68.87, 52.93; **IR** (ATR) 3066, 2962, 1713, 1619, 1247, 1183, 1119, 1013, 742, 698 cm^{-1}; **HRMS** (ESI): m/z calcd. for $C_{21}H_{17}NO_4Na$ $[M+Na]^+$: 370.1050; found 370.1056.

Methyl 2-[(benzoyloxycarbonyl)imino]-2-furanate (1k)

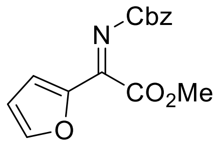

According to the typical procedure, the reaction with methyl α-oxo-2-furanate (1.1 g, 6.4 mmol) gave **1k** (0.92 g, 50% yield) as an orange oil. **^{1}H NMR** (300 MHz, $CDCl_3$) δ 7.46–7.43 (m, 2H), 7.40–7.35 (m, 4H), 7.28–7.26 (m, 1H), 6.54 (s, 1H), 5.31(s, 2H), 3.78 (s, 3H); **^{13}C NMR** (125 MHz, $CDCl_3$) δ 161.21, 148.25, 135.31, 129.03, 128.56, 128.53, 128.28, 122.04, 112.98, 111.13, 109.18, 68.63, 53.36; **IR** (ATR) 3421, 3143, 3033, 2955, 1721, 1619, 1247, 1191, 1007, 746 cm^{-1}; **HRMS** (ESI): m/z calcd. for $C_{15}H_{13}NO_5Na$ $[M+Na]^+$: 310.0686; found 310.0689.

Methyl 2-[(benzoyloxycarbonyl)imino]-2-thopheneacetate (1l)

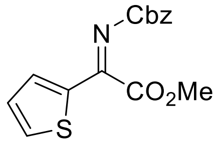

According to the typical procedure, the reaction with methyl α-oxo-2-thiopheneacetate (2.2 g, 13 mmol) gave **1l** (2.7 g, 69% yield) as a colorless oil. **^{1}H NMR** (300 MHz, $CDCl_3$) δ 7.81–7.80 (m, 1H), 7.68–7.65 (m, 1H), 7.47–7.43 (m, 2H), 7.42–7.33 (m, 3H), 7.15–7.11 (m, 1H), 5.28 (s, 2H), 3.70 (s, 3H); **^{13}C NMR** (125 MHz, $CDCl_3$) δ 161.45, 161.03, 155.91, 137.77, 135.62, 135.29, 134.73, 128.92, 128.62, 128.60, 128.55, 68.77, 53.15; **IR** (ATR) 3103, 3033, 2954, 1747, 1601, 1498, 1423, 1209, 976, 732 cm^{-1}; **HRMS** (ESI): m/z calcd. for $C_{15}H_{13}NO_4NaS$ $[M+Na]^+$: 326.0458; found 326.0462.

7 General procedure for the addition of glycinate Schiff base to acyclic ketimine

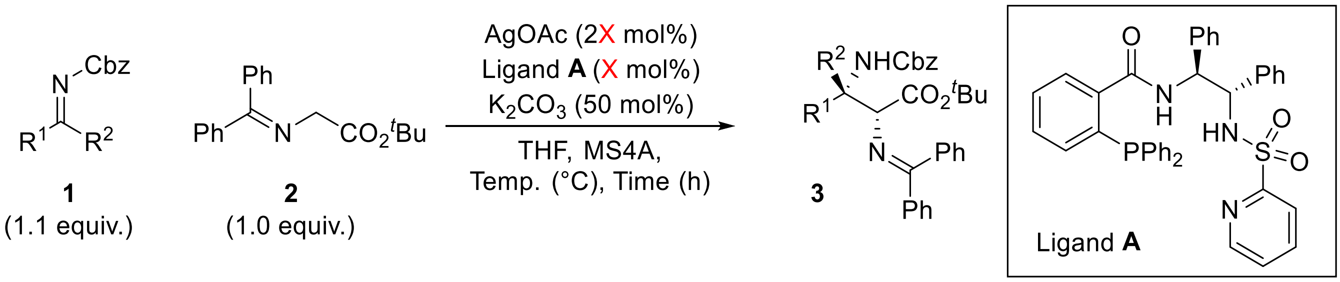

(2*S*,3*R*)-Methyl 2-[(benzyloxycarbonyl)amino]-3-(*tert*-butoxycarbonyl)-3-[(diphenylmethylene)amino]-2-phenylpropanate (3a)

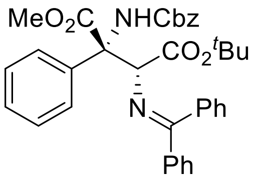

Ligand **A** (0.005 mmol, 5 mol%), silver acetate (0.01 mmol, 10 mol%), and MS4A (10 mg) were added to THF (1.0 mL). After stirring for 1 h at room temperature, ketiminoester **1a** (32.7 mg, 0.11 mmol), Schiff base **2** (29.5 mg, 0.10 mmol), and potassium carbonate (6.9 mg, 0.05 mmol) were added. Then, the reaction mixture was cooled to 0 °C. After stirring for 48 h at 0 °C, the reaction mixture was purified by silica gel column chromatography (hexane: EtOAc = 85/15) to afford the product **3a** (52.7 mg, 89% yield, 99:1 dr, 98:2 er) as a colorless oil. **Optical Rotation**: $[\alpha]_D^{25}$ +78.9 (*c* 0.17, $CHCl_3$); **^{1}H NMR** (300 MHz, $CDCl_3$) δ 7.62–7.56 (m, 4H), 7.44–7.26 (m, 14H), 6.90 (s, 1H), 6.79–6.77 (m, 2H), 5.15 (s, 2H), 4.56 (s, 1H), 3.72 (s, 3H), 1.27 (s, 9H); **^{13}C NMR** (125 MHz, $CDCl_3$) δ 172.61, 170.24, 168.32, 155.18, 139.10, 136.73, 136.48, 135.58, 130.76, 129.28, 129.07, 128.88, 128.72, 128.46, 128.30, 128.18, 128.10, 127.91, 127.71, 127.60, 82.75, 72.67, 68.16, 66.80, 52.82, 27.67; **IR** (ATR) 3417, 3061, 2979, 1729, 1659, 1625, 1598, 1495, 1258, 1146 cm^{-1}; **HRMS** (ESI): m/z calcd. for $C_{36}H_{36}N_2O_6Na$ $[M+Na]^+$: 615.2471; found 615.2465; **HPLC** (CHIRALPAK IG-3, hexane/^{i}PrOH = 95:5, 1.0 mL/min, 254 nm) 98:2 er, t_R 46.2 (major), t_R 58.0 (minor) min.

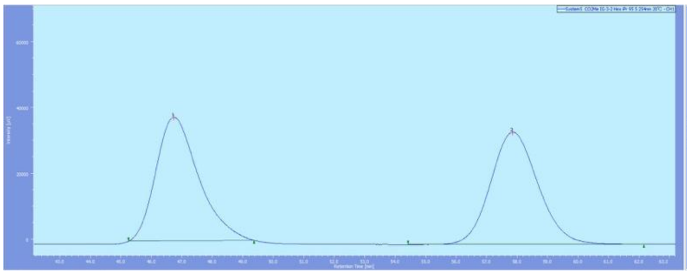

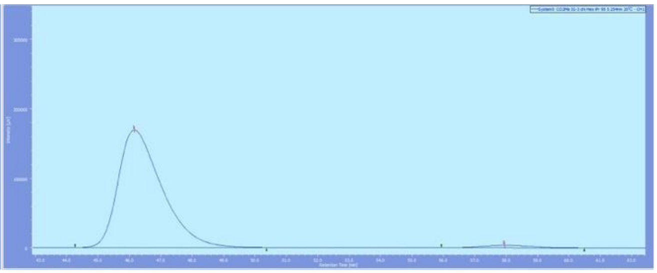

racemic-**3a**

Peak	tR (min)	Area (%)
1	46.7	49.9
2	57.9	50.1

(2*S*,3*R*)-**3a**

Peak	tR (min)	Area (%)
1	46.2	97.8
2	58.0	2.2

(2S,3R)-Methyl 2-[(benzyloxycarbonyl)amino]-3-(*tert*-butoxycarbonyl)-3-[(diphenylmethylene)amino]-2-(4-fluorophenyl)propanate (3b)

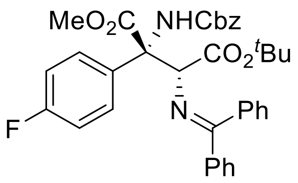

Ligand **A** (0.005 mmol, 5 mol%), silver acetate (0.01 mmol, 10 mol%), and MS4A (10 mg) were added to THF (1.0 mL). After stirring for 1 h at room temperature, ketiminoester **1b** (34.7 mg, 0.11 mmol), Schiff base **2** (29.5 mg, 0.10 mmol), and potassium carbonate (6.9 mg, 0.05 mmol) were added. Then, the reaction mixture was cooled to 0 °C. After stirring for 48 h at 0 °C, the reaction mixture was purified by silica gel column chromatography (hexane: EtOAc = 95/5) to afford the product **3b** (63.8 mg, 99% yield, 99:1 dr, 97:3 er) as a colorless oil. **Optical Rotation**: $[\alpha]_D^{25}$ +74.6 (*c* 0.07, $CHCl_3$); **^{1}H NMR** (400 MHz, $CDCl_3$) δ 7.63–7.60 (m, 2H), 7.56–7.54 (m, 2H), 7.43–7.31 (m, 11H), 6.97–6.93 (m, 2H), 6.87–6.82 (m, 3H), 5.13 (s, 2H), 4.51 (s, 1H), 3.69 (s, 3H), 1.25 (s, 9H); **^{13}C NMR** (176 MHz, $CDCl_3$) δ 172.86, 170.09, 168.28, 162.50 (d, J = 246.4 Hz), 155.21, 139.08, 136.66, 135.61, 132.33, 130.97, 130.05, 130.00, 129.11, 128.57, 128.50, 128.30, 128.25, 127.58, 114.44, 114.32, 83.06, 72.72, 67.75, 67.01, 52.96, 27.74; **IR** (ATR) 3407, 3066, 2983, 1726, 1628, 1567, 1537, 1494, 1276, 1216 cm^{-1}; **^{19}F NMR** (282 MHz, $CDCl_3$) δ −115.35; **HRMS** (ESI): m/z calcd. for $C_{36}H_{35}N_2O_6NaF$ $[M+Na]^+$: 633.2372; found 633.2380; **HPLC** (CHIRALPAK IBN-3, hexane/ ^{i}PrOH = 98:2, 1.0 mL/min, 254 nm) 97:3 er, t_R 19.7 (major), t_R 17.4 (minor) min.

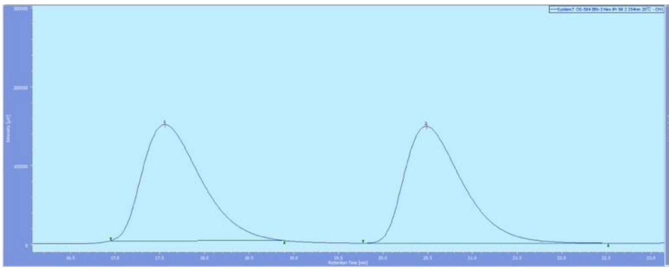

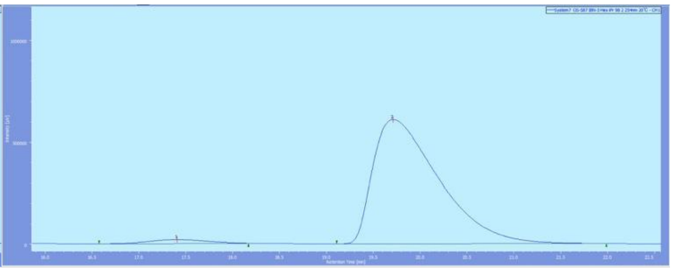

racemic-**3b**

Peak	tR (min)	Area (%)
1	17.6	50.0
2	20.5	50.0

(*2S,3R*)-**3b**

Peak	tR (min)	Area (%)
1	17.4	2.7
2	19.7	97.3

(*2S,3R*)-Methyl 2-[(benzyloxycarbonyl)amino]-3-(*tert*-butoxycarbonyl)-3-[(diphenylmethylene)amino]-2-(4-bromophenyl)propanate (3c)

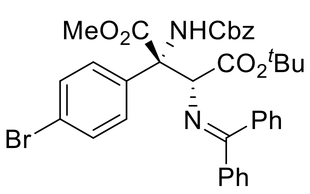

Ligand **A** (0.005 mmol, 5 mol%), silver acetate (0.01 mmol, 10 mol%), and MS4A (10 mg) were added to THF (1.0 mL). After stirring for 1 h at room temperature, ketiminoester **1c** (41.4 mg, 0.11 mmol), Schiff base **2** (29.5 mg, 0.10 mmol), and potassium carbonate (6.9 mg, 0.05 mmol) were added. Then, the reaction mixture was cooled to 0 °C. After stirring for 48 h at 0 °C, the reaction mixture was purified by silica gel column chromatography (hexane: EtOAc = 95/5) to afford the product **3c** (61.1 mg, 91% yield, 99:1 dr, 96:4 er) as a colorless oil. **Optical Rotation**: $[\alpha]_D^{25}$ +127.2 (*c* 0.09, $CHCl_3$); **^{1}H NMR** (300 MHz, $CDCl_3$) δ 7.56–7.48 (m, 4H), 7.45–7.30 (m, 13H), 7.25 (s, 1H), 6.86–6.80 (m, 2H), 5.12 (s, 2H), 4.50 (s, 1H), 3.70 (s, 3H), 1.26 (s, 9H); **^{13}C NMR** (125 MHz, $CDCl_3$) δ 172.86, 169.80, 168.13, 155.13, 138.94, 136.54, 135.65, 135.42, 130.93, 130.59, 129.88, 129.05, 129.03, 128.50, 128.43, 128.22, 128.18, 127.49, 127.04, 122.03, 83.06, 72.44, 67.80, 66.96, 52.94, 27.68; **IR** (ATR) 3425, 2983, 2855, 1727, 1621, 1488, 1265, 1009, 734, 702 cm^{-1}; **HRMS** (ESI): m/z calcd. for $C_{36}H_{35}N_2O_6NaBr$ $[M+Na]^+$: 693.1571; found 693.1570; **HPLC** (CHIRALPAK IM, hexane/ ^{i}PrOH = 95:5, 1.0 mL/min, 254 nm) 96:4 er, t_R 7.8 (major), t_R 10.2 (minor) min.

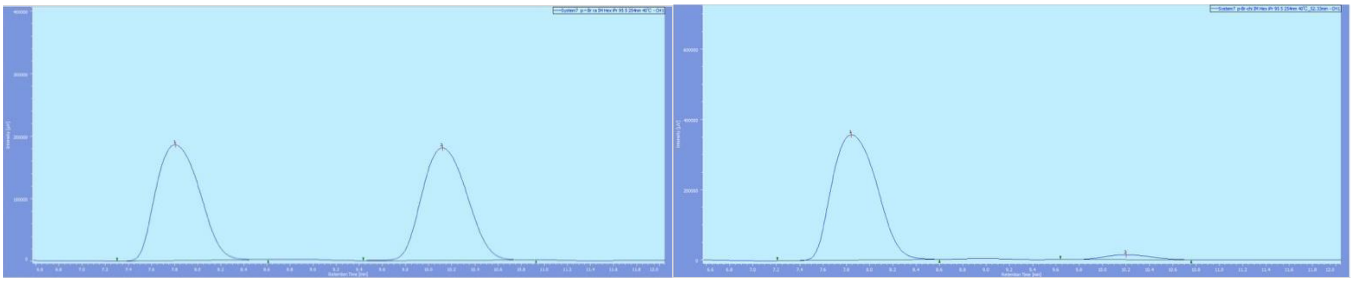

racemic-**3c**

Peak	tR (min)	Area (%)
1	7.8	50.1
2	10.1	49.9

(*2S,3R*)-**3c**

Peak	tR (min)	Area (%)
1	7.8	96.2
2	10.2	3.8

(2*S*,3*R*)-Methyl 2-[(benzyloxycarbonyl)amino]-3-(*tert*-butoxycarbonyl)-3-[(diphenylmethylene)amino]-2-(4-chlorophenyl)propanate (3d)

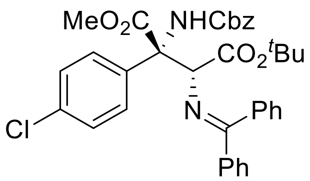

Ligand **A** (0.005 mmol, 5 mol%), silver acetate (0.01 mmol, 10 mol%), and MS4A (10 mg) were added to THF (1.0 mL). After stirring for 1 h at room temperature, ketiminoester **1d** (36.5 mg, 0.11 mmol), Schiff base **2** (29.5 mg, 0.10 mmol), and potassium carbonate (6.9 mg, 0.05 mmol) were added. Then, the reaction mixture was cooled to 0 °C. After stirring for 48 h at 0 °C, the reaction mixture was purified by silica gel column chromatography (hexane: EtOAc = 95/5) to afford the product **3d** (62.2 mg, 99% yield, 99:1 dr, 97:3 er) as a colorless oil. **Optical Rotation**: $[\alpha]_D^{25}$ +17.5 (*c* 1.20, $CHCl_3$); **^{1}H NMR** (400 MHz, $CDCl_3$) δ 7.63–7.60 (m, 2H), 7.56–7.54 (m, 2H), 7.43–7.31 (m, 11H), 6.97–6.93 (m, 2H), 6.87–6.82 (m, 3H), 5.16–5.09 (m, 2H), 4.51 (s, 1H), 3.69 (s, 3H), 1.25 (s, 9H); **^{13}C NMR** (125 MHz, $CDCl_3$) δ 172.90, 169.88, 168.17, 155.17, 138.95, 136.54, 135.74, 135.45, 135.10, 133.74, 130.96, 130.12, 129.56, 129.07, 128.52, 128.45, 128.20, 127.65, 127.50, 127.23, 83.07, 72.49, 67.72, 66.97, 52.96, 27.66; **IR** (ATR) 3425, 2979, 2884, 1728, 1626, 1496, 1220, 1145, 1012, 908 cm^{-1}; **HRMS** (ESI): m/z calcd. for $C_{36}H_{35}N_2O_6NaCl$ $[M+Na]^+$: 649.2076; found 649.2086; **HPLC** (CHIRALPAK IBN-3, hexane/ ^{i}PrOH = 98:2, 1.0 mL/min, 254 nm) 97:3 er, t_R 18.6 (major), t_R 15.6 (minor) min.

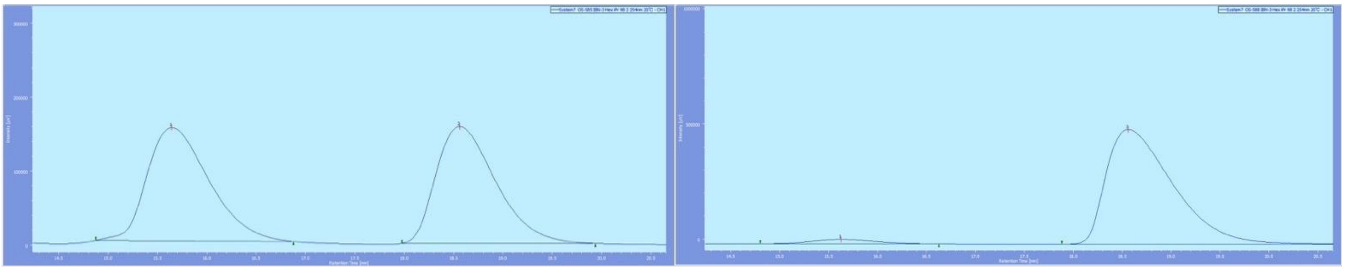

racemic-**3d**

Peak	tR (min)	Area (%)
1	15.6	50.1
2	18.6	49.9

(2*S*,3*R*)-**3d**

Peak	tR (min)	Area (%)
1	15.6	3.4
2	18.6	96.6

(*2S,3R*)-Methyl 2-[(benzyloxycarbonyl)amino]-3-(*tert*-butoxycarbonyl)-3-[(diphenylmethylene)amino]-2-(4-iodophenyl)propanate (3e)

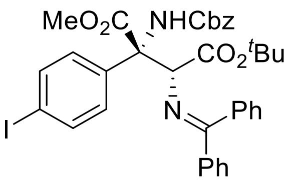

Ligand **A** (0.005 mmol, 5 mol%), silver acetate (0.01 mmol, 10 mol%), and MS4A (10 mg) were added to THF (1.0 mL). After stirring for 1 h at room temperature, ketiminoester **1e** (46.6 mg, 0.11 mmol), Schiff base **2** (29.5 mg, 0.10 mmol), and potassium carbonate (6.9 mg, 0.05 mmol) were added. Then, the reaction mixture was cooled to 0 °C. After stirring for 48 h at 0 °C, the reaction mixture was purified by silica gel column chromatography (hexane: EtOAc = 95/5) to afford the product **3e** (71.1 mg, 99% yield, 99:1 dr, 96:4 er) as a colorless oil. **Optical Rotation**: $[\alpha]_D^{25}$ +155.6 (*c* 0.05, $CHCl_3$); **^{1}H NMR** (700 MHz, $CDCl_3$) δ 7.58–7.57 (m, 2H), 7.55–7.53 (m, 2H), 7.43–7.32 (m, 14H), 6.84–6.79 (m, 2H), 5.14–5.09 (m, 2H), 4.50 (s, 1H), 3.69 (s, 3H), 1.25 (s, 9H); **^{13}C NMR** (176 MHz, $CDCl_3$) δ 172.95, 169.88, 168.23, 155.21, 139.03, 136.66, 136.49, 135.48, 131.00, 130.16, 129.12, 129.09, 128.57, 128.53, 128.48, 128.28, 128.26, 128.23, 127.57, 93.93, 83.13, 72.48, 67.96, 67.01, 53.01, 27.75; **IR** (ATR) 3424, 3067, 3031, 2978, 1727, 1626, 1492, 1221, 1145, 1003 cm^{-1}; **HRMS** (ESI): m/z calcd. for $C_{36}H_{35}N_2O_6NaI$ $[M+Na]^+$: 741.1432; found 741.1437; **HPLC** (CHIRALPAK IBN-3, hexane/ ^{i}PrOH = 98:2, 1.0 mL/min, 254 nm) 96:4 er, t_R 19.4 (major), t_R 17.0 (minor) min.

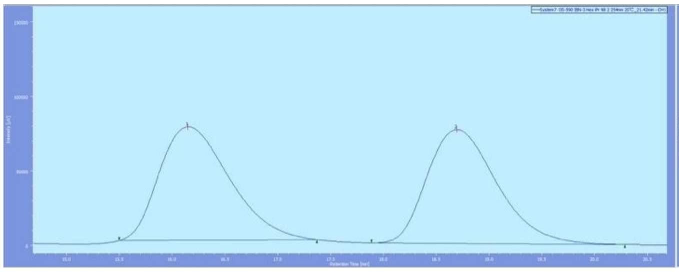

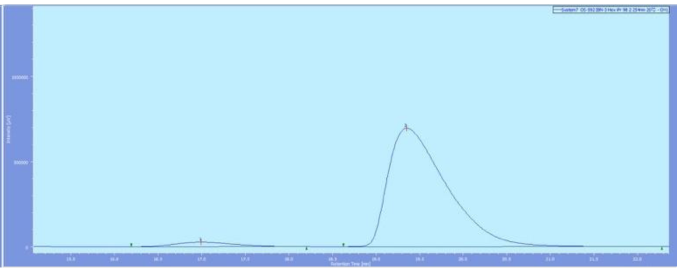

racemic-**3e**

Peak	tR (min)	Area (%)
1	16.2	50.1
2	18.7	49.9

(*2S,3R*)-**3e**

Peak	tR (min)	Area (%)
1	17.0	3.8
2	19.4	96.3

(2*S*,3*R*)-Methyl 2-[(benzyloxycarbonyl)amino]-3-(*tert*-butoxycarbonyl)-3-[(diphenylmethylene)amino]-2-(3-bromophenyl)propanate (3f)

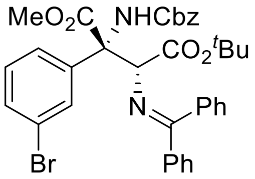

Ligand **A** (0.005 mmol, 5 mol%), silver acetate (0.01 mmol, 10 mol%), and MS4A (10 mg) were added to THF (1.0 mL). After stirring for 1 h at room temperature, ketiminoester **1f** (41.4 mg, 0.11 mmol), Schiff base **2** (29.5 mg, 0.10 mmol), and potassium carbonate (6.9 mg, 0.05 mmol) were added. Then, the reaction mixture was cooled to 0 °C. After stirring for 48 h at 0 °C, the reaction mixture was purified by silica gel column chromatography (hexane: EtOAc = 90/10) to afford the product **3f** (66.5 mg, 99% yield, 99:1 dr, 96:4 er) as a colorless oil. **Optical Rotation**: $[\alpha]_D^{25}$ +42.3 (*c* 0.11, $CHCl_3$); **^{1}H NMR** (300 MHz, $CDCl_3$) δ 7.80–7.79 (m, 1H), 7.60–7.55 (m, 3H), 7.43–7.36 (m, 9H), 7.34–7.31 (m, 3H), 7.18–7.12 (m, 1H), 6.94 (s, 1H), 6.83–6.81 (m, 2H), 5.20 (d, J = 12.5 Hz, 1H), 5.12 (d, J = 12.5 Hz, 1H), 4.50 (s, 1H), 3.78–3.67 (m, 3H), 1.30 (s, 9H); **^{13}C NMR** (125 MHz, $CDCl_3$) δ 172.85, 169.67, 168.05, 155.19, 138.91, 136.58, 135.46, 131.91, 131.34, 130.91, 130.79, 130.61, 129.27, 129.07, 128.54, 128.49, 128.16, 127.49, 126.85, 126.28, 124.64, 121.80, 83.70, 72.47, 67.75, 69.98, 52.97, 27.10; **IR** (ATR) 3424, 3113, 2984, 1728, 1620, 1597, 1567, 1500, 1265, 1224 cm^{-1}; **HRMS** (ESI): m/z calcd. for $C_{36}H_{35}N_2O_6NaBr$ $[M+Na]^+$: 693.1571; found 693.1569; **HPLC** (CHIRALPAK IM, hexane/^{i}PrOH = 95:5, 1.0 mL/min, 254 nm) 96:4 er, t_R 9.0 (major), t_R 10.7 (minor) min.

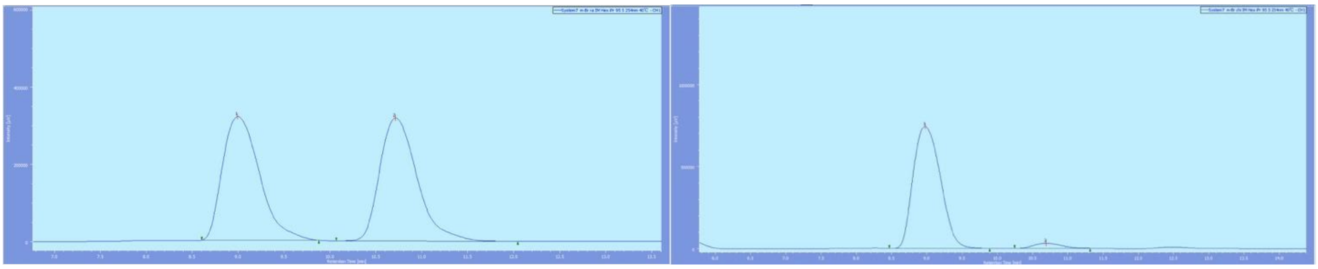

racemic-**3f**

Peak	tR (min)	Area (%)
1	9.0	50.0
2	10.7	50.0

(2S,3*R*)-**3f**

Peak	tR (min)	Area (%)
1	9.0	96.2
2	10.7	3.8

(*2S,3R*)-Methyl 2-[(benzyloxycarbonyl)amino]-3-(*tert*-butoxycarbonyl)-3-[(diphenylmethylene)amino]-2-(2-fluorophenyl)propanate (3g)

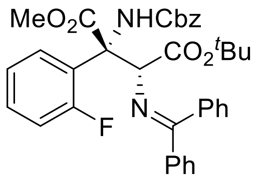

Ligand **A** (0.005 mmol, 5 mol%), silver acetate (0.01 mmol, 10 mol%), and MS4A (10 mg) were added to THF (1.0 mL). After stirring for 1 h at room temperature, ketiminoester **1g** (34.7 mg, 0.11 mmol), Schiff base **2** (29.5 mg, 0.10 mmol), and potassium carbonate (6.9 mg, 0.05 mmol) were added. Then, the reaction mixture was cooled to 0 °C. After stirring for 48 h at 0 °C, the reaction mixture was purified by silica gel column chromatography (hexane: EtOAc = 90/10) to afford the product **3g** (60.5 mg, 99% yield, 99:1dr, 96:4 er) as a colorless oil. **Optical Rotation**: $[\alpha]_D^{25}$ +75.6 (*c* 0.10, $CHCl_3$); **^{1}H NMR** (300 MHz, $CDCl_3$) δ 7.52–7.45 (m, 4H), 7.41–7.30 (m, 7H), 7.26–7.19 (m, 5H), 7.05–7.00 (m, 1H), 6.93–6.86 (m, 1H), 6.76–6.74 (m, 2H), 5.14 (d, *J* = 12.6 Hz, 1H), 5.03 (d, *J* = 12.6 Hz, 2H), 4.89 (s, 1H), 3.72 (s, 3H), 1.37 (s, 9H); **^{13}C NMR** (125 MHz, $CDCl_3$) δ 172.58, 169.53, 168.20, 160.00 (d, *J* = 246.3 Hz), 154.87, 139.02, 136.71, 135.27, 130.66, 130.28, 129.70, 129.63, 129.07, 128.92, 128.40, 128.10, 127.96, 127.89, 123.12, 123.10, 115.92, 115.74, 82.10, 68.04, 66.86, 66.63, 52.87, 27.19, 1.06; **^{19}F NMR** (282 MHz, $CDCl_3$) δ −110.01; **IR** (ATR) 3403, 3102, 2983, 1728, 1620, 1578, 1487, 1446, 1265, 734 cm^{-1}; **HRMS** (ESI): m/z calcd. for $C_{36}H_{35}N_2O_6NaF$ $[M+Na]^+$: 633.2372; found 633.2386; **HPLC** (CHIRALPAK IM, hexane/^{i}PrOH = 95:5, 1.0 mL/min, 254 nm) 96:4 er, t_R 9.5 (major), t_R 12.8 (minor) min.

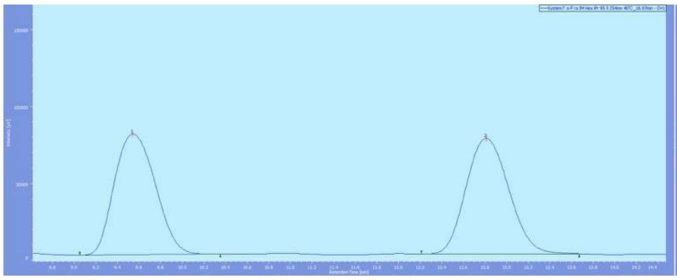

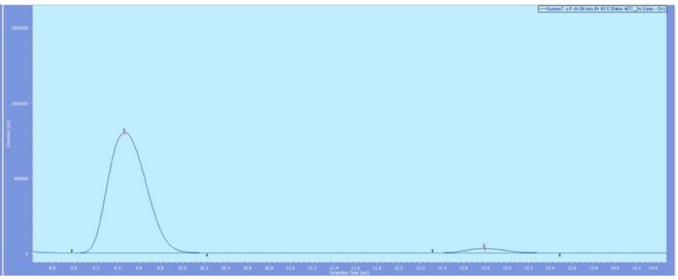

racemic-**3g**

Peak	tR (min)	Area (%)
1	9.5	50.0
2	12.8	50.0

(2S,3*R*)-**3g**

Peak	tR (min)	Area (%)
1	9.5	96.3
2	12.8	3.7

(*2S,3R*)-Methyl 2-[(benzyloxycarbonyl)amino]-3-(*tert*-butoxycarbonyl)-3-[(diphenylmethylene)amino]-2-(4-methylphenyl)propanate (3h)

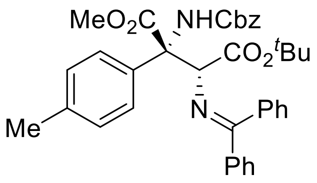

Ligand **A** (0.005 mmol, 5 mol%), silver acetate (0.01 mmol, 10 mol%), and MS4A (10 mg) were added to THF (1.0 mL). After stirring for 1 h at room temperature, ketiminoester **1h** (34.2 mg, 0.11 mmol), Schiff base **2** (29.5 mg, 0.10 mmol), and potassium carbonate (6.9 mg, 0.05 mmol) were added. Then, the reaction mixture was cooled to 0 °C. After stirring for 48 h at 0 °C, the reaction mixture was purified by silica gel column chromatography (hexane: EtOAc = 90/10) to afford the product **3h** (51.0 mg, 84% yield, 99:1 dr, 98:2 er) as a colorless oil. **Optical Rotation**: $[\alpha]_D^{25}$ −17.1 (*c* 0.07, $CHCl_3$); **^{1}H NMR** (300 MHz, $CDCl_3$) δ 7.59–7.56 (m, 2H), 7.47–7.31 (m, 13H), 7.17–7.06 (m, 2H), 6.88 (s, 1H), 6.79–6.77 (m, 2H), 5.18–5.09 (m, 2H), 4.55 (s, 1H), 3.72 (s, 3H), 2.31 (s, 2H), 1.28 (s, 9H); **^{13}C NMR** (125 MHz, $CDCl_3$) δ 172.49, 170.41, 168.38, 155.15, 139.16, 137.26, 136.80, 135.61, 133.48, 130.71, 129.07, 128.83, 128.44, 128.30, 128.27, 128.14, 128.08, 128.02, 127.71, 127.61, 82.65, 72.61, 68.03, 66.72, 52.77, 27.69, 21.04; **IR** (ATR) 3406, 3000, 2917, 1725, 1634, 1575, 1491, 1446, 1251, 1142 cm^{-1}; **HRMS** (ESI): m/z calcd. for $C_{37}H_{38}N_2O_6Na$ $[M+Na]^+$: 629.2623; found 629.2628; **HPLC** (CHIRALPAK IM, hexane/^{i}PrOH = 98:2, 1.0 mL/min, 254 nm) 98:2 er, t_R 22.9 (major), t_R 40.3 (minor) min.

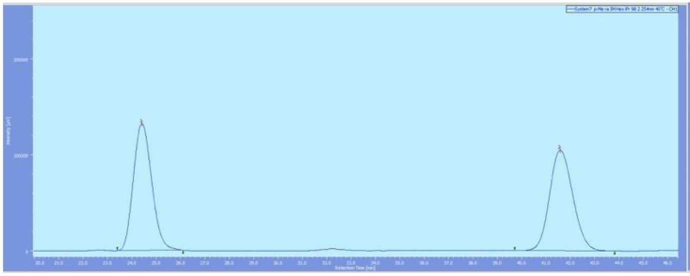

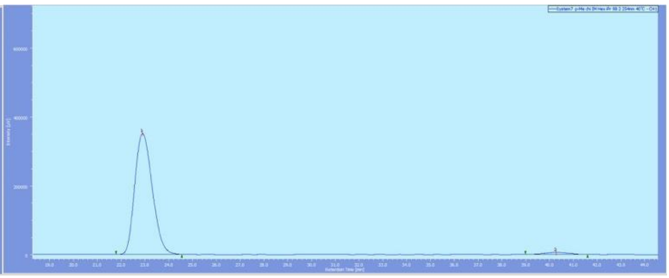

racemic-**3h**

Peak	tR (min)	Area (%)
1	24.4	50.0
2	41.6	50.0

(2S,3*R*)-**3h**

Peak	tR (min)	Area (%)
1	22.9	97.8
2	40.3	2.2

(2*S*,3*R*)-Methyl 2-[(benzyloxycarbonyl)amino]-3-(*tert*-butoxycarbonyl)-3-[(diphenylmethylene)amino]-2-(4-methoxyphenyl)propanate (3i)

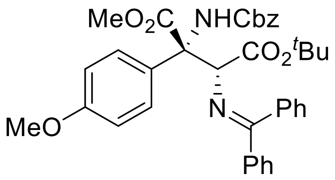

Ligand **A** (0.005 mmol, 5 mol%), silver acetate (0.01 mmol, 10 mol%), and MS4A (10 mg) were added to THF (1.0 mL). After stirring for 1 h at room temperature, ketiminoester **1i** (36.0 mg, 0.11 mmol), Schiff base **2** (29.5 mg, 0.10 mmol), and potassium carbonate (6.9 mg, 0.05 mmol) were added. Then, the reaction mixture was cooled to 0 °C. After stirring for 48 h at 0 °C, the reaction mixture was purified by silica gel column chromatography (hexane: EtOAc = 90/10) to afford the product **3i** (47.9 mg, 77% yield, 99:1 dr, 98:2 er) as a colorless oil. **Optical Rotation**: $[\alpha]_D^{25}$ +86.0 (*c* 0.09, $CHCl_3$); **^{1}H NMR** (300 MHz, $CDCl_3$) δ 7.59–7.51 (m, 5H), 7.44–7.31 (m, 11H), 6.86–6.79 (m, 4H), 5.18–5.08 (m, 2H), 4.52 (s, 1H), 3.78 (s, 3H), 3.71 (s, 3H), 1.27 (s, 9H); **^{13}C NMR** (125 MHz, $CDCl_3$) δ 172.52, 170.38, 168.35, 159.07, 155.13, 139.13, 136.73, 135.65, 130.75, 129.18, 129.06, 128.89, 128.59, 128.46, 128.34, 128.17, 128.10, 128.05, 127.58, 112.95, 82.72, 72.73, 67.74, 66.7, 55.30, 52.78, 27.71; **IR** (ATR) 3421, 3060, 2951, 1723, 1623, 1612, 1577, 1496, 1254, 1251 cm^{-1}; **HRMS** (ESI): m/z calcd. for $C_{37}H_{38}N_2O_7Na$ $[M+Na]^+$: 645.2572; found 645.2581; **HPLC** (CHIRALPAK IK, hexane/ ^{i}PrOH = 95:5, 1.0 mL/min, 254 nm) 98:2 er, t_R 26.3 (major), t_R 55.3 (minor) min.

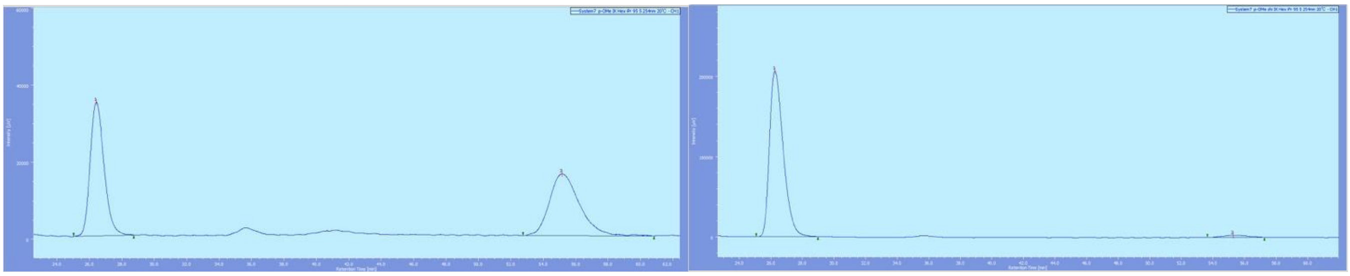

racemic-**3i**

Peak	tR (min)	Area (%)
1	26.4	50.1
2	55.1	49.9

(2S,3*R*)-**3i**

Peak	tR (min)	Area (%)
1	26.3	97.9
2	55.3	2.1

(2*S*,3*R*)-Methyl 2-[(benzyloxycarbonyl)amino]-3-(*tert*-butoxycarbonyl)-3-[(diphenylmethylene)amino]-2-(2-naphthyl)propanate (3j)

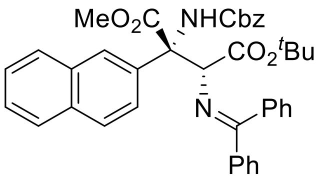

Ligand **A** (0.005 mmol, 5 mol%), silver acetate (0.01 mmol, 10 mol%), and MS4A (10 mg) were added to THF (1.0 mL). After stirring for 1 h at room temperature, ketiminoester **1j** (38.2 mg, 0.11 mmol), Schiff base **2** (29.5 mg, 0.10 mmol), and potassium carbonate (6.9 mg, 0.05 mmol) were added. Then, the reaction mixture was cooled to 0 °C. After stirring for 48 h at 0 °C, the reaction mixture was purified by silica gel column chromatography (hexane: EtOAc = 90/10) to afford the product **3j** (62.3 mg, 97% yield, 99:1 dr, 98:2 er) as a colorless oil. **Optical Rotation**: $[\alpha]_D^{25}$ +65.9 (*c* 0.06, $CHCl_3$); **^{1}H NMR** (300 MHz, $CDCl_3$) δ 7.89–7.68 (m, 6H), 7.59–7.56 (m, 2H), 7.48–7.43 (m, 4H), 7.41–7.24 (m, 9H), 7.05 (s, 1H), 6.67 (s, 1H), 5.24 (d, *J* = 12.4 Hz, 1H), 5.15 (d, *J* = 12.4 Hz, 1H), 4.68 (s, 1H), 3.76 (s, 3H), 1.25 (s, 9H); **^{13}C NMR** (125 MHz, $CDCl_3$) δ 172.67, 170.37, 168.39, 155.31, 139.08, 136.84, 135.46, 134.25, 132.83, 132.48, 130.77, 130.11, 129.29, 129.07, 128.86, 128.53, 128.39, 128.23, 128.10, 127.55, 127.28, 126.85, 126.66, 126.36, 126.06, 125.65, 82.78, 72.74, 68.37, 66.83, 52.89, 27.68; **IR** (ATR) 3410, 3060, 2962, 1725, 1619, 1597, 1575, 1492, 1258, 1142 cm^{-1}; **HRMS** (ESI): m/z calcd. for $C_{40}H_{38}N_2O_6Na$ $[M+Na]^+$: 665.2623; found 665.2628; **HPLC** (CHIRALPAK IF, hexane/ ^{i}PrOH = 95:5, 1.0 mL/min, 254 nm) 98:2 er, t_R 73.7 (major), t_R 62.2 (minor) min.

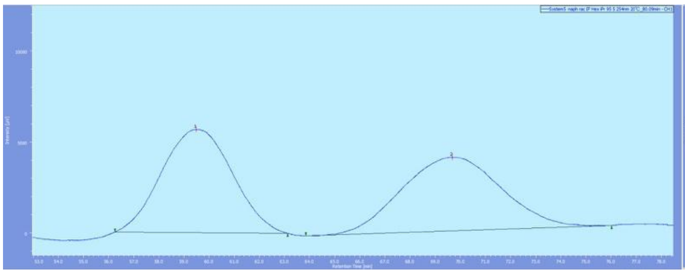

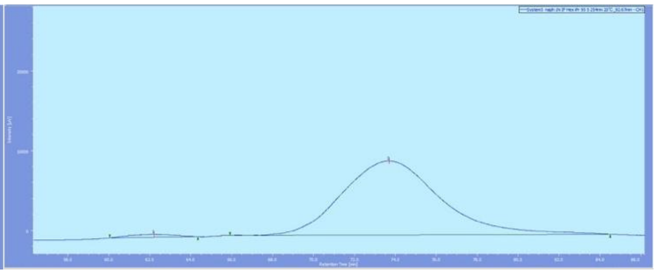

racemic-**3j**

Peak	tR (min)	Area (%)
1	59.5	49.9
2	69.7	50.1

(2S,3*R*)-**3j**

Peak	tR (min)	Area (%)
1	62.2	1.8
2	73.7	98.2

(2*S*,3*R*)-Methyl 2-[(benzyloxycarbonyl)amino]-3-(*tert*-butoxycarbonyl)-3-[(diphenylmethylene)amino]-2-(2-furyl)propanate (3k)

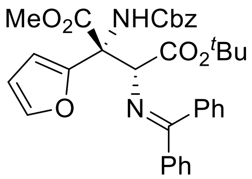

Ligand **A** (0.005 mmol, 5 mol%), silver acetate (0.01 mmol, 10 mol%), and MS4A (10 mg) were added to THF (1.0 mL). After stirring for 1 h at room temperature, ketiminoester **1k** (31.6 mg, 0.11 mmol), Schiff base **2** (29.5 mg, 0.10 mmol), and potassium carbonate (6.9 mg, 0.05 mmol) were added. Then, the reaction mixture was cooled to 0 °C. After stirring for 48 h at 0 °C, the reaction mixture was purified by silica gel column chromatography (hexane: EtOAc = 90/10) to afford the product **3k** (53.0 mg, 91% yield, 99:1 dr, 98:2 er) as a yellow oil. **Optical Rotation**: $[\alpha]_D^{25}$ +27.8 (*c* 0.11, $CHCl_3$); **^{1}H NMR** (300 MHz, $CDCl_3$) δ 7.58–7.55 (m, 2H), 7.44–7.34 (m, 9H), 7.32–7.26 (m, 3H), 7.00–6.97 (m, 2H), 6.92 (s, 1H), 6.60 (s, 1H), 6.34 (s, 1H), 5.20-5.07 (m, 2H), 4.62 (s, 1H), 3.72 (s, 3H), 1.37 (s, 9H); **^{13}C NMR** (125 MHz, $CDCl_3$) δ 172.77, 168.76, 167.65, 155.37, 150.07, 141.68, 139.13, 136.59, 135.68, 130.79, 130.10, 129.12, 129.05, 128.42, 128.30, 128.07, 127.99, 127.67, 110.68, 109.74, 82.86, 70.50, 66.85, 65.19, 52.97, 27.80; **IR** (ATR) 3417, 3060, 2974, 1732, 1657, 1623, 1601, 1492, 1254, 1146 cm^{-1}; **HRMS** (ESI): m/z calcd. for $C_{34}H_{34}N_2O_7Na$ $[M+Na]^+$: 605.2259; found 605.2271; **HPLC** (CHIRALPAK IM, hexane/^{i}PrOH = 95:5, 1.0 mL/min, 254 nm) 98:2 er, t_R 45.8 (major), t_R 50.8 (minor) min.

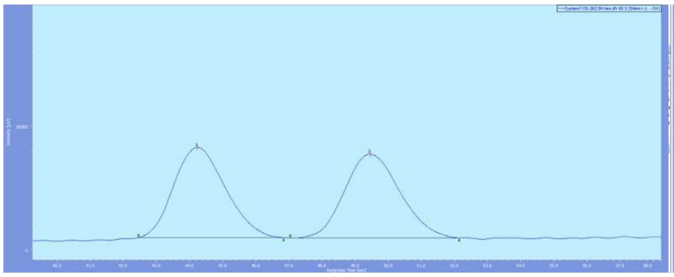

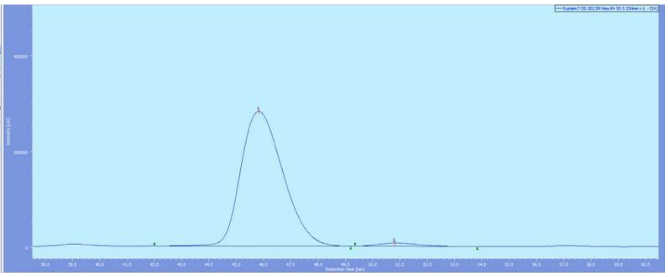

racemic-**3k**

Peak	tR (min)	Area (%)
1	44.2	50.1
2	49.5	49.9

(2S,3*R*)-**3k**

Peak	tR (min)	Area (%)
1	45.8	97.8
2	50.8	2.2

(*2S,3R*)-Methyl 2-[(benzyloxycarbonyl)amino]-3-(*tert*-butoxycarbonyl)-3-[(diphenylmethylene)amino]-2-(2-thienyl)propanate (3l)

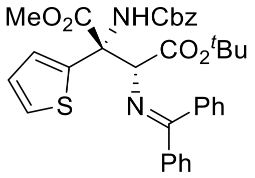

Ligand **A** (0.005 mmol, 5 mol%), silver acetate (0.01 mmol, 10 mol%), and MS4A (10 mg) were added to THF (1.0 mL). After stirring for 1 h at room temperature, ketiminoester **1l** (33.4 mg, 0.11 mmol), Schiff base **2** (29.5 mg, 0.10 mmol), and potassium carbonate (6.9 mg, 0.05 mmol) were added. Then, the reaction mixture was cooled to 0 °C. After stirring for 48 h at 0 °C, the reaction mixture was purified by silica gel column chromatography (hexane: EtOAc = 90/10) to afford the product **3l** (59.3mg, 99% yield, 99:1 dr, 95:5 er) as a yellow oil. **Optical Rotation**: $[\alpha]_D^{25}$ +61.8 (*c* 0.17, $CHCl_3$); **^{1}H NMR** (300 MHz, $CDCl_3$) δ 7.68–7.66 (m, 2H), 7.43–7.32 (m, 12H), 7.27–7.26 (m, 1H), 7.17–7.16 (m, 1H), 7.12 (s, 1H), 6.95–6.89 (m, 3H), 5.18–5.08 (m, 2H), 4.47 (s, 1H), 3.71 (s, 3H), 1.26 (s, 9H); **^{13}C NMR** (125 MHz, $CDCl_3$) δ 172.66, 169.57, 167.85, 155.00, 140.20, 138.94, 126.49, 125.67, 132.43, 131.98, 130.89, 129.19, 128.98, 128.50, 128.15, 127.67, 127.41, 126.72, 126.46, 125.50, 83.12, 72.42, 67.01, 53.05, 29.74, 27.68, ; **IR** (ATR) 3421, 3060, 2925, 1728, 1623, 1597, 1575, 1492, 1225, 1142 cm^{-1}; **HRMS** (ESI): m/z calcd. for $C_{34}H_{34}N_2O_6NaS$ $[M+Na]^+$: 621.2030; found 621.2045; **HPLC** (CHIRALPAK IK, hexane/ ^{i}PrOH = 95:5, 1.0 mL/min, 254 nm) 95:5 er, t_R 38.4 (major), t_R 67.2 (minor) min.

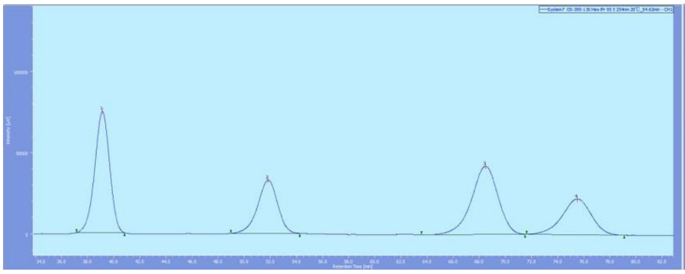

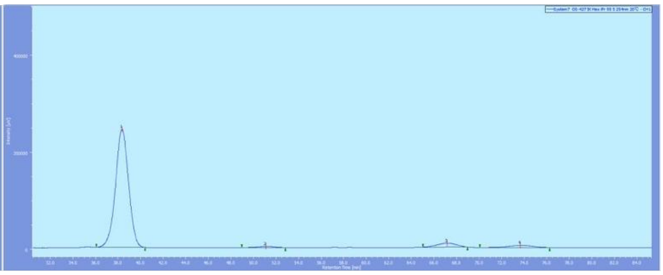

racemic-**3l**

Peak	tR (min)	Area (%)
1	39.1	50.0
2	68.5	50.0

(2S,3*R*)-**3l**

Peak	tR (min)	Area (%)
1	38.4	95.4
2	67.2	4.6

(2*S*,3*R*)-Methyl 2-[(benzyloxycarbonyl)amino]-3-(*tert*-butoxycarbonyl)-3-[(diphenylmethylene)amino]-4-phenylbut-3-ynoate (3m)

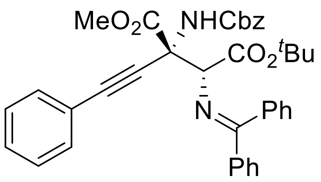

Ligand **A** (0.01 mmol, 10 mol%), silver acetate (0.02 mmol, 20 mol%), and MS4A (10 mg) were added to THF (1.0 mL). After stirring for 1 h at room temperature, ketiminoester **1m** (35.3 mg, 0.11 mmol), Schiff base **2** (29.5 mg, 0.10 mmol), and potassium carbonate (6.9 mg, 0.05 mmol) were added. Then, the reaction mixture was cooled to −20 °C. After stirring for 48 h at −20 °C, the reaction mixture was purified by silica gel column chromatography (hexane: EtOAc = 90/10, 1% TEA) to afford the product **3m** (43.8 mg, 71% yield, 99:1 dr, 90:10 er) as a yellow oil. **Optical Rotation**: $[\alpha]_D^{25}$ +140.0 (*c* 0.05, $CHCl_3$); **^{1}H NMR** (300 MHz, $CDCl_3$) δ 7.63–7.65 (m, 2H), 7.36–7.47 (m, 11H), 7.31–7.34 (m, 4H), 7.25–7.26 (m, 1H), 7.11–7.16 (m, 2H), 6.71 (s, 1H), 5.19 (s, 2H), 4.58 (s, 1H), 3.70 (s, 3H), 1.41 (s, 9H); **^{13}C NMR** (125 MHz, $CDCl_3$) δ 172.57, 167.35, 165.68, 154.27, 137.90, 135.41, 134.57, 130.98, 130.00, 128.28, 128.17, 127.51, 127.38, 127.25, 127.09, 127.02, 126.93, 126.79, 126.60, 121.40, 84.70, 81.65, 69.54, 65.91, 59.48, 52.33, 28.67, 26.86; **IR** (ATR) 3406, 3060, 2925, 1732, 1619, 1597, 1575, 1488, 1225, 1146 cm^{-1}; **HRMS** (ESI): m/z calcd. for $C_{38}H_{36}N_2O_6Na$ $[M+Na]^+$: 639.2466; found 639.2471; **HPLC** (CHIRALPAK IF, hexane/^{i}PrOH = 98:2, 1.0 mL/min, 254 nm) 90:10 er, t_R 82.1 (major), t_R 70.5 (minor) min.

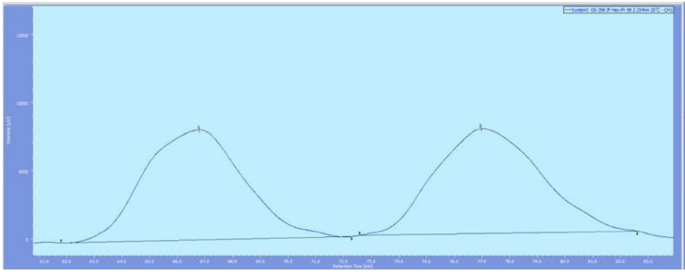

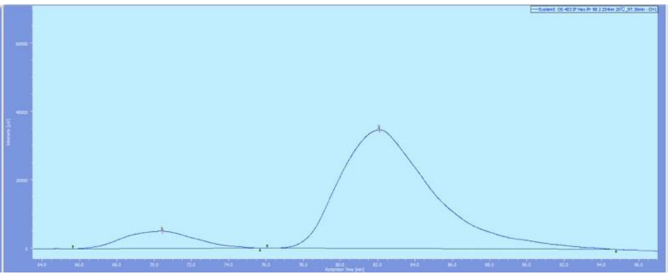

racemic-**3m**

Peak	tR (min)	Area (%)
1	66.8	49.9
2	77.0	50.1

(2S,3*R*)-**3m**

Peak	tR (min)	Area (%)
1	70.5	10.1
2	82.1	89.9

(*2S,3R*)-Methyl 2-[(benzyloxycarbonyl)amino]-3-(*tert*-butoxycarbonyl)-3-[(diphenylmethylene)amino]-4-(4-bromophenyl)but-3-ynoate (3n)

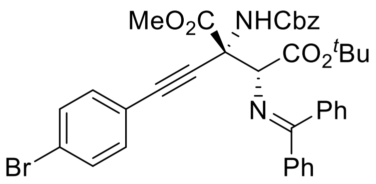

Ligand **A** (0.01 mmol, 10 mol%), silver acetate (0.02 mmol, 20 mol%), and MS4A (10 mg) were added to THF (1.0 mL). After stirring for 1 h at room temperature, ketiminoester **1n** (44.0 mg, 0.11 mmol), Schiff base **2** (29.5 mg, 0.10 mmol), and potassium carbonate (6.9 mg, 0.05 mmol) were added. Then, the reaction mixture was cooled to −20 °C. After stirring for 48 h at −20 °C, the reaction mixture was purified by silica gel column chromatography (hexane: EtOAc = 90/10, 1% TEA) to afford the product **3n** (30.3 mg, 44% yield, 99:1 dr, 88:12 er) as a yellow oil. **Optical Rotation**: $[\alpha]_D^{25}$ +96.9 (*c* 0.08, $CHCl_3$); **^{1}H NMR** (700 MHz, $CDCl_3$) δ 7.63–7.62 (m, 2H), 7.46–7.32 (m, 15H), 7.12–7.10 (m, 2H), 6.69 (br, 1H), 5.19–5.15 (m, 2H), 4.55 (s, 1H), 3.71 (s, 3H), 1.38 (s, 9H); **^{13}C NMR** (176 MHz, $CDCl_3$) δ 173.88, 168.33, 166.75, 155.40, 138.97, 136.49, 135.67, 133.55, 131.47, 131.21, 131.11, 129.40, 129.35, 128.68, 128.55, 128.22, 128.13, 127.90, 122.88, 121.51, 84.81, 82.87, 70.60, 67.11, 60.63, 53.54, 27.98, 27.75; **IR** (ATR) 3410, 3026, 2977, 1732, 1619, 1597, 1484, 1454, 1217, 1149 cm^{-1}; **HRMS** (ESI): m/z calcd. for $C_{38}H_{35}N_2O_6NaBr$ $[M+Na]^+$: 717.1571; found 717.1568; **HPLC** (CHIRALPAK IK, hexane/^{i}PrOH = 90:10, 1.0 mL/min, 254 nm) 88:12 er, t_R 12.0 (major), t_R 13.3 (minor) min.

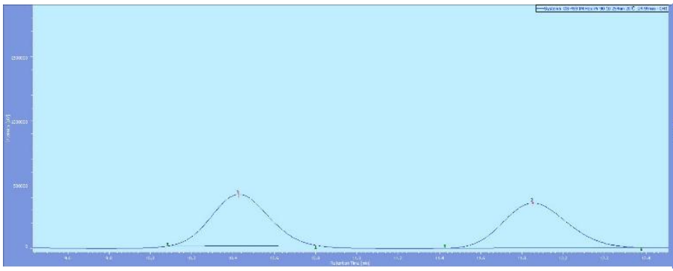

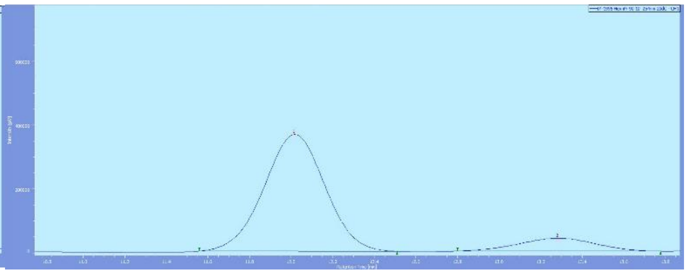

racemic-**3n**

Peak	tR (min)	Area (%)
1	10.4	50.2
2	11.9	49.8

(2S,3*R*)-**3n**

Peak	tR (min)	Area (%)
1	12.0	87.9
2	13.3	12.1

(*2S,3R*)-Methyl 2-[(benzyloxycarbonyl)amino]-3-(*tert*-butoxycarbonyl)-3-[(diphenylmethylene)amino]-4-(4-methoxyphenyl)but-3-ynoate (3o)

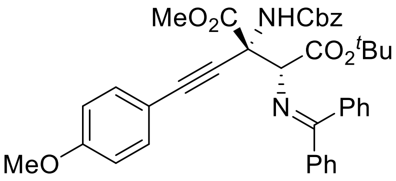

Ligand **A** (0.01 mmol, 10 mol%), silver acetate (0.02 mmol, 20 mol%), and MS4A (10 mg) were added to THF (1.0 mL). After stirring for 1 h at room temperature, ketiminoester **1o** (38.6 mg, 0.11 mmol), Schiff base **2** (29.5 mg, 0.10 mmol), and potassium carbonate (6.9 mg, 0.05 mmol) were added. Then, the reaction mixture was cooled to −20 °C. After stirring for 48 h at −20 °C, the reaction mixture was purified by silica gel column chromatography (hexane: EtOAc = 80/20) to afford the product **3o** (29.7 mg, 46% yield, 99:1 dr, 92:8 er) as a yellow oil. **Optical Rotation**: $[\alpha]_D^{25}$ +37.9 (*c* 0.19, $CHCl_3$); **^{1}H NMR** (500 MHz, $CDCl_3$) δ 7.68–7.63 (m, 2H), 7.46–7.44 (m, 5H), 7.42–7.29 (m, 8H), 7.17–7.11 (m, 2H), 6.79–6.72 (m, 3H), 5.18 (s, 2H), 4.57 (s, 1H), 3.82–3.78 (m, 3H), 3.71 (s, 3H), 1.40 (s, 9H); **^{13}C NMR** (125 MHz, $CDCl_3$) δ 173.60, 168.59, 166.85, 159.73, 155.40, 139.00, 136.51, 135.65, 133.54, 131.06, 130.12, 129.36, 129.23, 128.57, 128.44, 128.15, 127.99, 127.88, 114. 57, 113.70, 85.75, 82.93, 82.66, 70.66, 86.93, 60.53, 55.26, 53.57, 27.90; **IR** (ATR) 3407, 2979, 2925, 1730, 1626, 1572, 1489, 1369, 1220, 1146 cm^{-1}; **HRMS** (ESI): m/z calcd. for $C_{39}H_{38}N_2O_7Na$ $[M+Na]^+$: 669.2572; found 669.2585; **HPLC** (CHIRALPAK IM, hexane/ ^{i}PrOH = 90:10, 1.0 mL/min, 254 nm) 92:8 er, t_R 17.8 (major), t_R 20.1 (minor) min.

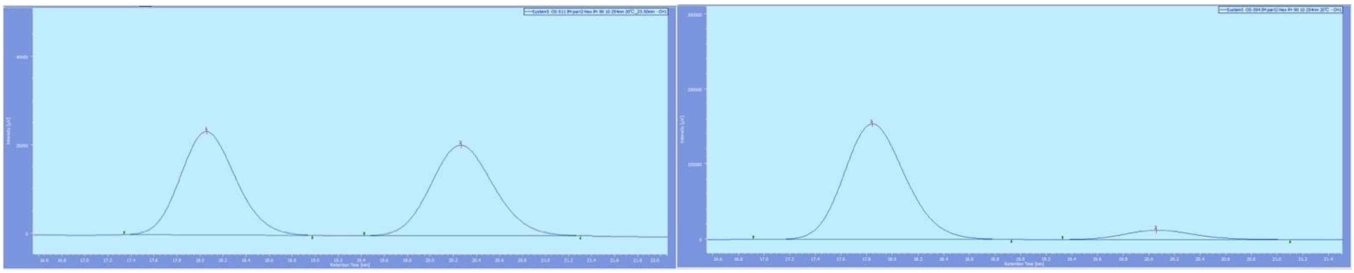

racemic-**3o**

Peak	tR (min)	Area (%)
1	18.1	50.1
2	20.3	49.9

(2S,3*R*)-**3o**

Peak	tR (min)	Area (%)
1	17.8	91.7
2	20.1	8.3

(*2S,3R*)-Ethyl 2-[(benzyloxycarbonyl)amino]-3-(*tert*-butoxycarbonyl)-3-[(diphenylmethylene)amino]-2-phenylpropanate (3p)

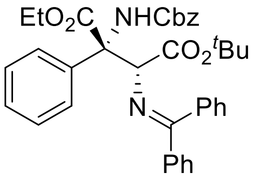

Ligand **A** (0.005 mmol, 5 mol%), silver acetate (0.01 mmol, 10 mol%), and MS4A (10 mg) were added to THF (1.0 mL). After stirring for 1 h at room temperature, ketiminoester **1p** (34.2 mg, 0.11 mmol), Schiff base **2** (29.5 mg, 0.10 mmol), and potassium carbonate (6.9 mg, 0.05 mmol) were added. Then, the reaction mixture was cooled to 0 °C. After stirring for 48 h at 0 °C, the reaction mixture was purified by silica gel column chromatography (hexane: EtOAc = 90/10) to afford the product **3p** (57.0 mg, 94% yield, 99:1 dr, 98:2 er) as a colorless oil. **Optical Rotation**: $[\alpha]_D^{25}$ +83.7 (*c* 0.13, $CHCl_3$); **^{1}H NMR** (300 MHz, $CDCl_3$) δ 7.64–7.58 (m, 5H), 7.43–7.33 (m, 10H), 7.28–7.25 (m, 3H), 6.86–6.80 (m, 3H), 5.14 (s, 2H), 4.57 (s, 1H), 4.25–4.15 (m, 2H), 1.25 (s, 9H), 1.18 (t, J = 7.0 Hz, 3H); **^{13}C NMR** (125 MHz, $CDCl_3$) δ 172.46, 169.65, 168.30, 155.02, 139.12, 136.77, 136.45, 135.56, 132.43, 130.68, 130.07, 129.27, 129.03, 128.79, 128.40, 128.24, 128.04, 127.94, 127.57, 127.50, 82.65, 72.53, 68.05, 66.68, 61.93, 27.63, 13.97, ; **IR** (ATR) 3421, 3060, 2974, 1725, 1623, 1597, 1575, 1496, 1254, 1149 cm^{-1}; **HRMS** (ESI): m/z calcd. for $C_{37}H_{38}N_2O_6Na$ $[M+Na]^+$: 629.2623; found 629.2628; **HPLC** (CHIRALPAK IM, hexane/^{i}PrOH = 95:5, 1.0 mL/min, 254 nm) 98:2 er, t_R 13.2 (major), t_R 20.4 (minor) min.

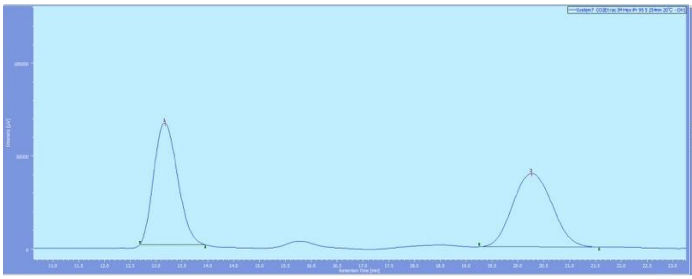

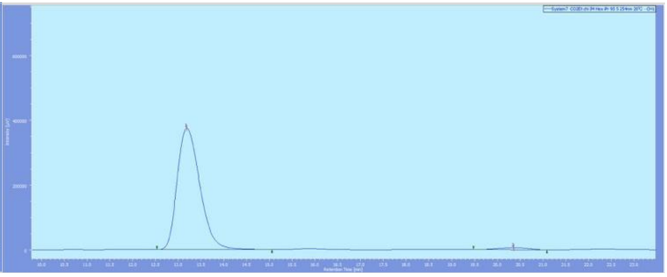

racemic-**3p**

Peak	tR (min)	Area (%)
1	13.2	50.0
2	20.3	50.0

(2S,3*R*)-**3p**

Peak	tR (min)	Area (%)
1	13.2	97.8
2	20.4	2.2

(*2S,3R*)-Isopropyl 2-[(benzyloxycarbonyl)amino]-3-(*tert*-butoxycarbonyl)-3-[(diphenylmethylene)amino]-2-phenylpropanate (3q)

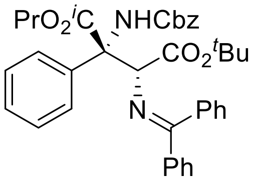

Ligand **A** (0.005 mmol, 5 mol%), silver acetate (0.01 mmol, 10 mol%), and MS4A (10 mg) were added to THF (1.0 mL). After stirring for 1 h at room temperature, ketiminoester **1q** (35.8 mg, 0.11 mmol), Schiff base **2** (29.5 mg, 0.10 mmol), and potassium carbonate (6.9 mg, 0.05 mmol) were added. Then, the reaction mixture was cooled to 0 °C. After stirring for 48 h at 0 °C, the reaction mixture was purified by silica gel column chromatography (hexane: EtOAc = 90/10) to afford the product **3q** (55.9 mg, 90% yield, 99:1 dr, 98:2 er) as a colorless oil. **Optical Rotation**: $[\alpha]_D^{25}$ +71.7 (*c* 0.13, $CHCl_3$); **^{1}H NMR** (300 MHz, $CDCl_3$) δ 7.63–7.60 (m, 5H), 7.42–7.28 (m, 13H), 6.82–6.80 (m, 3H), 5.18–5.97 (m, 2H), 4.59 (s, 1H), 1.21 (s, 9H), 1.17 (d, J = 5.7 Hz, 6H); **^{13}C NMR** (125 MHz, $CDCl_3$) δ 172.36, 169.15, 168.34, 154.92, 139.17, 136.84, 136.57, 135.62, 130.69, 129.30, 129.04, 128.84, 128.75, 128.59, 128.45, 128.26, 128.06, 128.03, 127.60, 127.47, 82.63, 72.42, 69.84, 68.07, 66.66, 27.66, 21.56, ; **IR** (ATR) 3421, 3060, 2974, 1721, 1657, 1623, 1575, 1492, 1258, 1146 cm^{-1}; **HRMS** (ESI): m/z calcd. for $C_{38}H_{40}N_2O_6Na$ $[M+Na]^+$: 643.2779; found 643.2783; **HPLC** (CHIRALPAK IM, hexane/^{i}PrOH = 95:15, 1.0 mL/min, 254 nm) 98:2 er, t_R 9.4 (major), t_R 12.7 (minor) min.

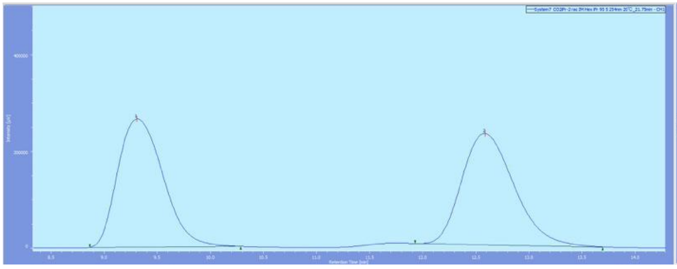

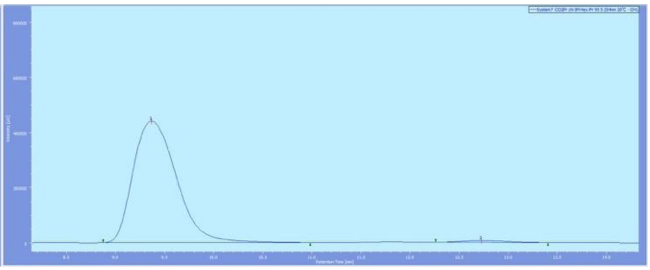

racemic-**3q**

Peak	tR (min)	Area (%)
1	9.3	49.9
2	12.6	50.1

(2S,3*R*)-**3q**

Peak	tR (min)	Area (%)
1	9.4	98.4
2	12.7	1.6

(*2S,3R*)-Benzyl 2-[(benzyloxycarbonyl)amino]-3-(*tert*-butoxycarbonyl)-3-[(diphenylmethylene)amino]-2-phenylpropanate (3r)

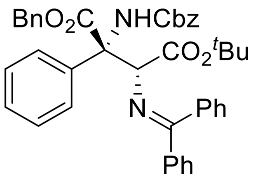

Ligand **A** (0.005 mmol, 5 mol%), silver acetate (0.01 mmol, 10 mol%), and MS4A (10 mg) were added to THF (1.0 mL). After stirring for 1 h at room temperature, ketiminoester **1r** (41.1 mg, 0.11 mmol), Schiff base **2** (29.5 mg, 0.10 mmol), and potassium carbonate (6.9 mg, 0.05 mmol) were added. Then, the reaction mixture was cooled to 0 °C. After stirring for 48 h at 0 °C, the reaction mixture was purified by silica gel column chromatography (hexane: EtOAc = 90/10) to afford the product **3r** (63.5 mg, 95% yield, 99:1 dr, 98:2 er) as a colorless oil. **Optical Rotation**: $[\alpha]_D^{25}$ −19.8 (*c* 0.12, $CHCl_3$); **^{1}H NMR** (400 MHz, $CDCl_3$) δ 7.70–7.68 (m, 2H), 7.57–7.56 (m, 2H), 7.43–7.30 (m, 9H), 7.27–7.23 (m, 5H), 7.22–7.20 (m, 3H), 7.16–7.13 (m, 2H), 6.82 (s, 1H), 6.61 (s, 2H), 5.21–5.12 (m, 4H), 4.55(s, 1H), 1.13 (s, 9H); **^{13}C NMR** (125 MHz, $CDCl_3$) δ 172.71, 169.44, 168.24, 155.13, 139.08, 136.62, 136.30, 135.58, 132.50, 130.78, 130.12, 129.32, 129.11, 128.92, 128.85, 128.73, 128.52, 128.36, 128.28, 128.12, 128.03, 127.83, 127.57, 127.45, 82.86, 72.59, 68.05, 67.57, 66.88, 29.76, 27.58; **IR** (ATR) 3425, 3060, 2974, 1728, 1623, 1597, 1577, 1488, 1251, 1142 cm^{-1}; **HRMS** (ESI): m/z calcd. for $C_{42}H_{40}N_2O_6Na$ $[M+Na]^+$: 691.2779; found 691.2787; **HPLC** (CHIRALPAK IM, hexane/^{i}PrOH = 90:10, 1.0 mL/min, 254 nm) 98:2 er, t_R 14.3 (major), t_R 16.6 (minor) min.

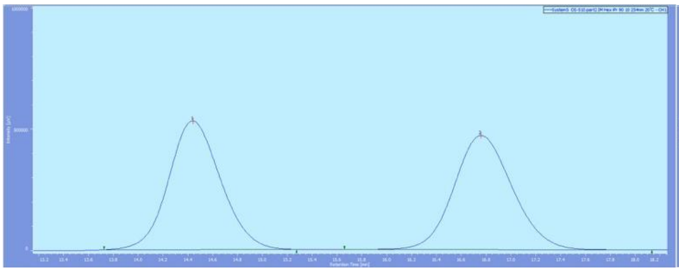

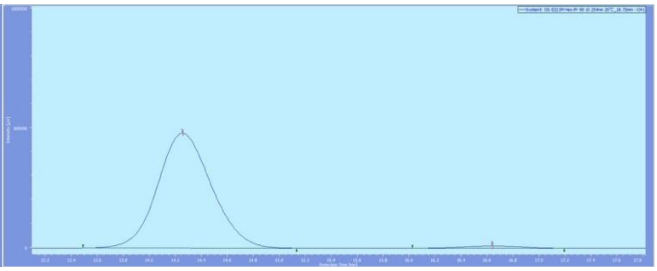

racemic-**3r**

Peak	tR (min)	Area (%)
1	14.4	50.1
2	16.8	49.9

(2S,3*R*)-**3r**

Peak	tR (min)	Area (%)
1	14.3	98.1
2	16.6	1.9

(*2S,3R*)-2-[(benzyloxycarbonyl)amino]-3-(*tert*-butoxycarbonyl)-3-[(diphenylmethylene)amino]-2-phenylacetonitrile (3s)

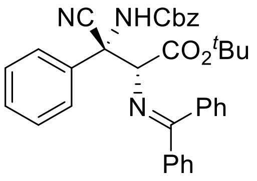

Ligand **A** (0.005 mmol, 5 mol%), silver acetate (0.01 mmol, 10 mol%), and MS4A (10 mg) were added to THF (1.0 mL). After stirring for 1 h at room temperature, ketiminoester **1s** (29.1 mg, 0.11 mmol), Schiff base **2** (29.5 mg, 0.10 mmol), and potassium carbonate (6.9 mg, 0.05 mmol) were added. Then, the reaction mixture was cooled to 0 °C. After stirring for 48 h at 0 °C, the reaction mixture was purified by silica gel column chromatography (hexane: EtOAc = 90/10) to afford the product **3s** (45.3 mg, 81% yield, 84:16 dr, 94:6 er) as a colorless oil. **Optical Rotation**: $[\alpha]_D^{25}$ +181.2 (*c* 0.08, $CHCl_3$); **^{1}H NMR** (400 MHz, $CDCl_3$, diastereomeric mixture) δ 7.67–7.53 (m, 2H), 7.47–7.39 (m, 3H), 7.38–7.22 (m, 14H), 7.01–6.99 (m, 1H), 6.37–6.35 (m, 1H), 5.18–5.13 (m, 2H), 4.21 (s, 1H), 1.48 (s, 9H); **^{13}C NMR** (125 MHz, $CDCl_3$, diastereomeric mixture) δ 174.28, 173.89, 166.82, 166.58, 154.57, 154.51, 138.36, 138.07, 135.83, 135.19, 134.74, 132.46, 131.28, 131.22, 130.91, 130.07, 129.32, 129.16, 129.12, 128.98, 128.84, 128.79, 128.68, 128.63, 128.54, 128.50, 128.45, 128.24, 128.18, 128.14, 128.07, 127.79, 127.67, 127.25, 127.09, 126.90, 125.98, 125.92, 118.11, 117.67, 83.86, 83.51, 72.37, 67.44, 64.47, 60.42, 27.83, 27.60, 25.30, 20.57; **IR** (ATR) 3409, 2917, 2849, 2371, 2311, 1776, 1735, 1474, 757, 698 cm^{-1}; **HRMS** (ESI): m/z calcd. for $C_{35}H_{33}N_3O_4Na$ $[M+Na]^+$: 582.2364; found 582.2376; **HPLC** (CHIRALPAK IG-3, hexane/^{i}PrOH = 95:5, 1.0 mL/min, 254 nm) 94:6 er, t_R 36.8 (major), t_R 46.3 (minor) min.

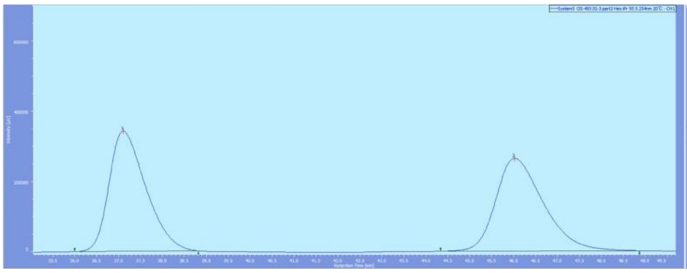

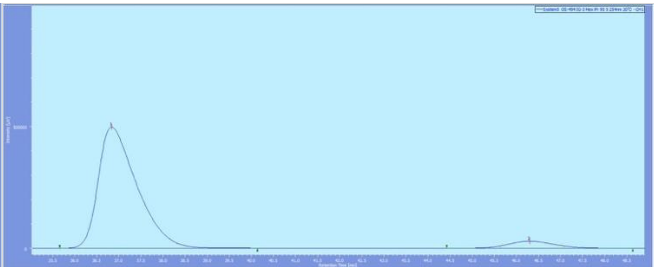

racemic-**3s**

Peak	tR (min)	Area (%)
1	37.1	50.1
2	46.0	49.9

(2S,3*R*)-**3s**

Peak	tR (min)	Area (%)
1	36.8	93.5
2	46.3	6.5

***tert*-Buthyl (*R*)-2-[(*S*)-3-((benzyloxycarbonyl)amino)-1-methyl-2-oxoindolin-3-yl]-2-[(diphenylmethylene)aminoacetate (3t)**

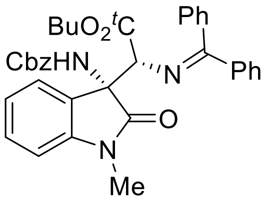

Ligand **A** (0.005 mmol, 5 mol%), silver acetate (0.01 mmol, 10 mol%), and MS4A (10 mg) were added to THF (1.0 mL). After stirring for 1 h at room temperature, ketiminoester **1t** (29.4 mg, 0.11 mmol), Schiff base **2** (29.5 mg, 0.10 mmol), and potassium carbonate (6.9 mg, 0.05 mmol) were added. Then, the reaction mixture was cooled to 0 °C. After stirring for 48 h at 0 °C, the reaction mixture was purified by silica gel column chromatography (hexane: EtOAc = 85/15) to afford the product **3t** (36.8 mg, 62% yield, 57:43 dr, 67:33 er (major), 97:3 er (minor)) as a colorless oil. **Optical Rotation**: $[\alpha]_D^{25}$ +214.9 (*c* 0.05, $CHCl_3$); **^{1}H NMR** (400 MHz, $CDCl_3$) δ 7.63–7.55 (m, 3H), 7.45–7.31 (m, 8H), 7.29–7.26 (m, 3H), 7.12–7.03 (m, 4H), 6.78 (s, 1H), 6.42 (s, 1H), 4.94–4.90 (m, 2H), 4.45 (s, 1H), 3.25 (s, 3H), 1.11 (s, 9H); **^{13}C NMR** (125 MHz, $CDCl_3$) δ 174.48, 174.35, 166.40, 154.47, 144.70, 144.62, 138.98, 135.88, 135.70, 131.01, 129.52, 128.98, 128.72, 128.63, 128.45, 128.30, 128.14, 127.81, 127.58, 124.45, 122.55, 107.88, 82.48, 68.91, 67.13, 62.66, 29.73, 27.48; **IR** (ATR) 3650, 3175, 2919, 2370, 2340, 1793, 1588, 1487, 1336, 1174 cm^{-1}; **HRMS** : m/z calcd. for $C_{36}H_{35}N_3O_5Na$ $[M+Na]^+$: 612.2469; found 612.2482; **HPLC** (CHIRALPAK AD-3, hexane/^{i}PrOH = 95:5, 1.0 mL/min, 254 nm) 97:3 er, t_R 42.8 (major), t_R 89.4 (minor) min.

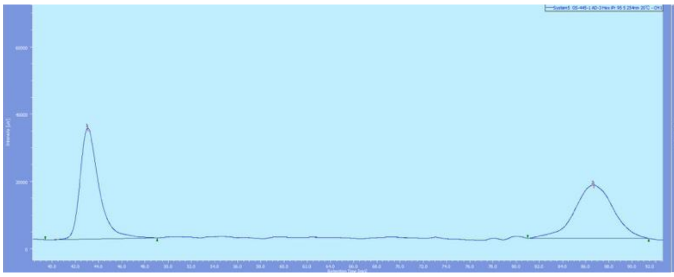

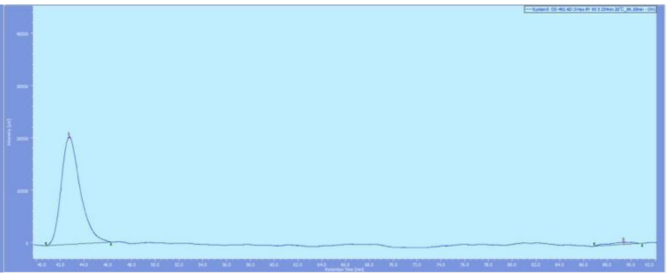

racemic-**3t**

Peak	tR (min)	Area (%)
1	43.1	50.0
2	86.7	50.0

(2S,3*R*)-**3t**

Peak	tR (min)	Area (%)
1	42.8	97.3
2	89.4	2.7

***tert*-Buthyl-(*2S,3R*)-3-(benzyloxycarbonylamino)-2-[(diphenylmethylene)amino]-3-phenylpropanate (3u)**

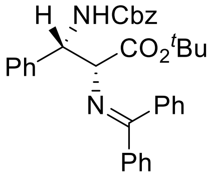

Ligand **A** (0.005 mmol, 5 mol%), silver acetate (0.01 mmol, 10 mol%), and MS4A (10 mg) were added to THF (1.0 mL). After stirring for 1 h at room temperature, ketiminoester **1u** (26.3 mg, 0.11 mmol), Schiff base **2** (29.5 mg, 0.10 mmol), and potassium carbonate (6.9 mg, 0.05 mmol) were added. Then, the reaction mixture was cooled to 0 °C. After stirring for 48 h at 0 °C, the reaction mixture was purified by silica gel column chromatography (hexane: EtOAc = 95/5) to afford the product **3u** (44.4 mg, 83% yield, 99:1 dr, 89:11 er) as a colorless oil. **Optical Rotation**: $[\alpha]_D^{25}$ +214.9 (*c* 0.05, $CHCl_3$); **^{1}H NMR** (500 MHz, $CDCl_3$) δ 7.55–7.53 (m, 2H), 7.40–7.28 (m, 10H), 7.24–7.18 (m, 5H), 7.16–7.14 (m, 2H), 6.64–6.62 (m, 1H), 6.48–6.47 (m, 1H), 5.49–5.47 (m, 1H), 5.18 (d, *J* = 12.3 Hz, 1H), 5.07 (d, *J* = 12.3 Hz, 1H), 4.15–4.14 (m, 1H), 1.42 (s, 9H); **^{13}C NMR** (125 MHz, $CDCl_3$) δ 174.24, 168.85, 155.80, 140.31, 138.84, 136.62, 135.98, 130.66, 130.58, 128.83, 128.52, 128.38, 128.32, 128.26, 128.12, 128.04, 127.17, 126.65, 126.58, 82.08, 70.04, 66.84, 57.24, 27.92; **IR** (ATR) 3650, 3175, 2919, 2370, 2340, 1793, 1588, 1487, 1336, 1174 cm^{-1}; **HRMS** (ESI) calcd. for $C_{34}H_{34}N_2O_4Na$ $[M+Na]^+$: 557.2411; found 557.2432; **HPLC** (CHIRALPAK IG-3, hexane/ ^{i}PrOH = 90:10, 1.0 mL/min, 254 nm) 89:11 er, t_R 36.6 (major), t_R 92.7 (minor) min.

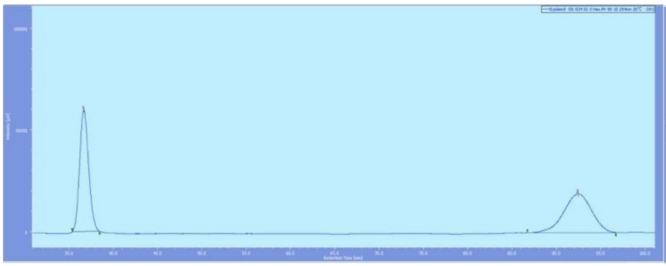

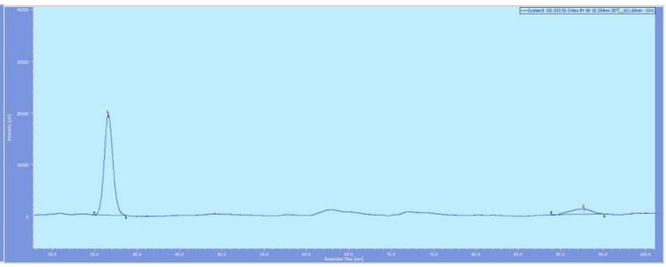

racemic-**3u**

Peak	tR (min)	Area (%)
1	36.7	50.1
2	92.5	49.9

(2S,3*R*)-**3u**

Peak	tR (min)	Area (%)
1	36.6	88.8
2	92.7	11.2

1-Benzyloxy-4-(*tert*-butoxy)-5-methyl-(*2S,3R*)-5-phenyl-2-oxo-1,5-imidazolidinetricarboxylate (4)

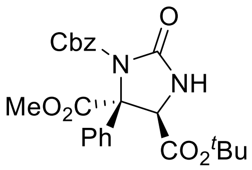

Compound **3a** was dissolved in 1 M HCl/ THF and stirred at 0 °C. After 1 h, the mixture was neutralized by adding saturated aqueous $NaHCO_3$ and then extracted by EtOAc. The organic phase was separated, dried over Na_2SO_4, and concentrated to give the primary amine compounds **3a'**. To a solution of **3a'** (42.8 mg, 0.10 mmol) in CH_2Cl_2 (1.2 mL) was added slowly a solution of triphosgene (29.9 mg, 0.10 mmol) at 0 °C. The mixture was allowed to reach room temperature and stirred for 2 h. After removal of the solvent to afford the product **4** (45.4 mg, 99% yield, 99:1 dr, 98:2 er) as a colorless oil. **Optical Rotation**: $[\alpha]_D^{25}$ +32.7 (*c* 0.10, $CHCl_3$); **^{1}H NMR** (400 MHz, $CDCl_3$) δ 8.61–8.59 (m, 1H), 7.39–7.35 (m, 7H), 7.33–7.31 (m, 2H), 6.86–6.85 (m, 1H), 5.83–5.81 (m, 1H), 5.17–5.11 (m, 2H), 3.76–3.75 (m, 3H), 1.42–1.41 (m, 9H); **^{13}C NMR** (125 MHz, $CDCl_3$) δ 170.06, 166.29, 156.19, 148.84, 135.61, 134.24, 129.70, 128.90, 128.70, 128.41, 126.57, 125.61, 83.97, 67.91, 67.40, 61.16, 54.32, 27.79; **IR** (ATR) 3390, 2982, 2881, 1743, 1710, 1499, 1488, 1371, 1336, 1270 cm^{-1}; **HRMS** (ESI): m/z calcd. for $C_{24}H_{26}N_2O_7Na$ $[M+Na]^+$: 477.1633; found 477.1639; **HPLC** (CHIRALPAK IM, hexane/^{i}PrOH = 85:15, 1.0 mL/min, 245 nm) 98:2 er, t_R 8.3 (major), t_R 9.9 (minor) min.

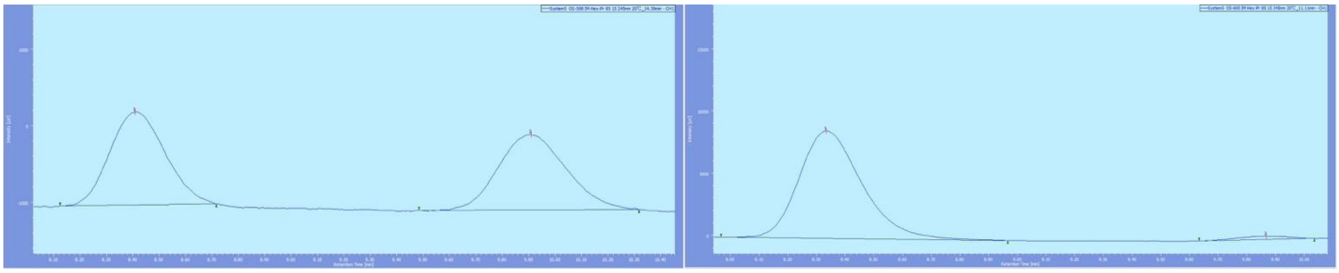

racemic-**4**

Peak	tR (min)	Area (%)
1	8.4	50.1
2	9.9	49.9

(2S,3*R*)-**4**

Peak	tR (min)	Area (%)
1	8.3	97.5
2	9.9	2.5

(*5S,6R,9S*)-5-Methyl-6-(*tert*-butoxy)-9benzyl-13,13-dimethyl-3,11-dioxo-2,12-dioxa-1,5-phenyl-4,7,10-triazatetradecane-dicarboxylate (5)

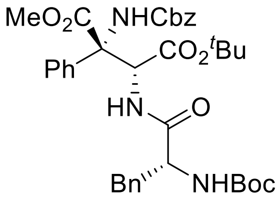

Compound **3a** was dissolved in 1 M HCl/ THF and stirred at 0 °C. After 1 h, the mixture was neutralized by adding saturated aqueous $NaHCO_3$ and then extracted by EtOAc. The organic phase was separated, dried over Na_2SO_4, and concentrated to give the primary amine compounds **3a'**. (*tert*-Butoxycarbonyl)-L-phenylalanine (26.5 mg, 0.10 mmol), EDCI·HCl (31.1 mg, 0.20 mmol), and HOBT·H_2O (30.6 mg, 0.20 mmol) were added to a solution of **3a'** (64.3 mg, 0.15 mmol) in CH_2Cl_2 (1.0 mL) at room temperature. The reaction mixture was stirred for 18 h at room temperature and then concentrated under reduced pressure. After concentration, dilute the mixture with EtOAc and wash with an aqueous solution of 1M HCl, a saturated $NaHCO_3$ aqueous solution, water, and brine, sequentially. Dry the organic phase over Na_2SO_4, filter, and concentrate under reduced pressure. The crude product was purified by silica gel column chromatography (eluent: hexane: EtOAc = 8:2) to afford **5** (44.3 mg, 66% yield) as a colorless oil. **Optical Rotation**: $[\alpha]_D^{25}$ −9.8 (*c* 0.44, $CHCl_3$); **^{1}H NMR** (400 MHz, $CDCl_3$) δ 8.76–8.73 (m, 1H), 7.39–7.34 (m, 5H), 7.33–7.27 (m, 5H), 7.25–7.16 (m, 3H), 7.10–7.08 (m, 2H), 6.84 (s, 1H), 6.11–6.09 (m, 1H), 5.12 (s, 2H), 4.87–4.85 (m, 1H), 4.39–4.36 (m, 1H), 3.75 (s, 3H), 2.94–2.89 (m, 1H), 2.70–2.64 (m, 1H), 1.40 (m, 9H), 1.21–1.14 (m, 9H); **^{13}C NMR** (176 MHz, $CDCl_3$) δ 172.35, 170.87, 167.52, 155.86, 154.74, 137.04, 136.04, 134.71, 129.43, 128.70, 128.62, 128.58, 128.52, 128.41, 128.38, 126.86, 126.58, 83.17, 79.37, 67.68, 67.57, 58.01, 55.24, 54.17, 38.56, 28.22, 27.91; **IR** (ATR) 3337, 2979, 1718, 1676, 1528, 1494, 1455, 1368, 1273, 1150, 910, 727 cm^{-1}; **HRMS** (ESI): m/z calcd. for $C_{37}H_{45}N_3O_9Na$ $[M+Na]^+$: 698.3048; found 698.3055; **HPLC** (CHIRALPAK IA, hexane/ ^{i}PrOH = 80:20, 1.0 mL/min, 254 nm) 98:2 er, t_R 6.7 (major), t_R 14.0 (minor) min.

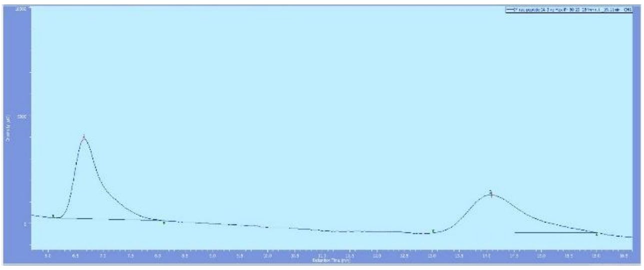

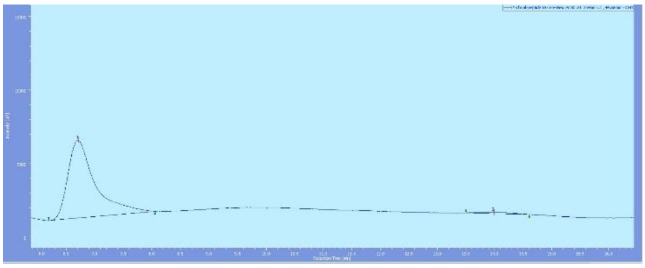

racemic-**5**

Peak	tR (min)	Area (%)
1	6.7	50.0
2	14.1	50.0

(2S,3*R*)-**5**

Peak	tR (min)	Area (%)
1	6.7	98.0
2	14.0	2.0

(*2S,3R*)-Methyl 2-amino-3-(*tert*-butoxycarbonyl)-3-[(diphenylmethylene)amino]-2-phenylpropionic acid (6)

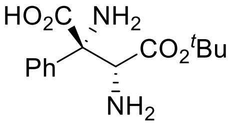

Compound **3r** was dissolved in 1 M HCl/ THF and stirred at 0 °C. After 1 h, the mixture was neutralized by adding saturated aqueous $NaHCO_3$ and then extracted by EtOAc. The organic phase was separated, dried over Na_2SO_4, and concentrated to give the primary amine compounds **3r'**. A solution of **3r'** (35.3 mg, 0.07 mmol), Pd/C (14.0 mg, 10 wt%, 0.007 mmol, 10 mol% in MeOH (2.0 mL) under H_2 atmosphere was stirred for 1 h at room temperature and filtered through a celite pad. The filtrate was concentrated in vacuo to give **6** (15.8 mg, 81% yield, 99:1 dr, 98:2 er) as a yellow solid. Er was determined by methyl esterification and cyclization of **6** using trimethysilyl diazomethane and triphogene. **Optical Rotation**: $[\alpha]_D^{25}$ +9.7 (*c* 0.28, MeOH); **m.p.** 112.0–113.0 ºC; **^{1}H NMR** (400 MHz, CD_3OD) δ 7.62–7.60 (m, 2H), 7.41–7.31 (m, 3H), 4.20 (s, 1H), 1.34 (s, 9H); **^{13}C NMR** (176 MHz, CD_3OD) δ 172.10, 171.38, 137.68, 128.30, 127.96, 126.48, 82.01, 68.65, 58.87, 26.71; **IR** (ATR) 3380, 2974, 2879, 1725, 1632, 1530, 1494, 1365, 1232, 1150 cm^{-1}; **HRMS** (ESI): m/z calcd. for $C_{14}H_{20}N_2O_4Na$ $[M+Na]^+$: 303.1316; found 303.1322; **HPLC** (CHIRALPAK IBN-3, hexane/ ^{i}PrOH = 70:30, 1.0 mL/min, 254 nm) 98:2 er, t_R 9.6 (major), t_R 10.9 (minor) min.

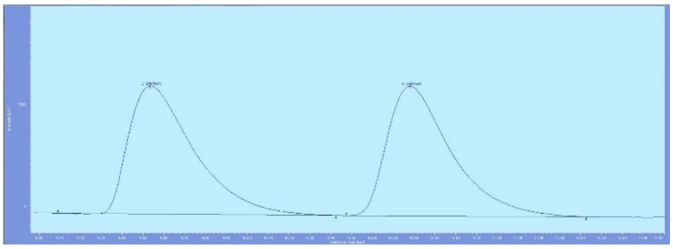

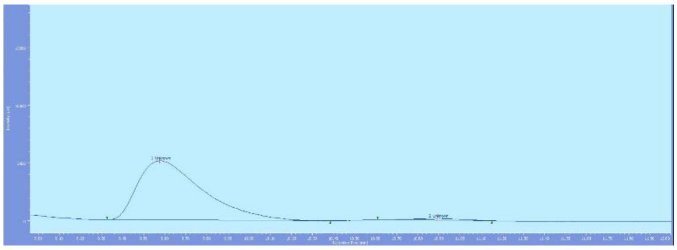

racemic-**6**

Peak	tR (min)	Area (%)
1	9.5	49.9
2	10.8	50.1

(2S,3*R*)-**6**

Peak	tR (min)	Area (%)
1	9.6	98.0
2	10.9	2.0

8 X-ray crystallographic structure

The obtained compound **3c** was smoothly converted to the α,β-diamino acid derivative **7** in nearly quantitative yield by treatment with 1 M HCl, and subsequent *N*-benzoylation of **7** with 4-bromobenzoyl chloride provided the corresponding product **8** in 99% yield, 99:1 dr, 96:4 er without loss of enantiopurity. X-ray crystallographic analysis of product **7** unambiguously established its absolute configuration (2*S*,3*R*), and the configurations of the other products were tentatively assumed by analogy.

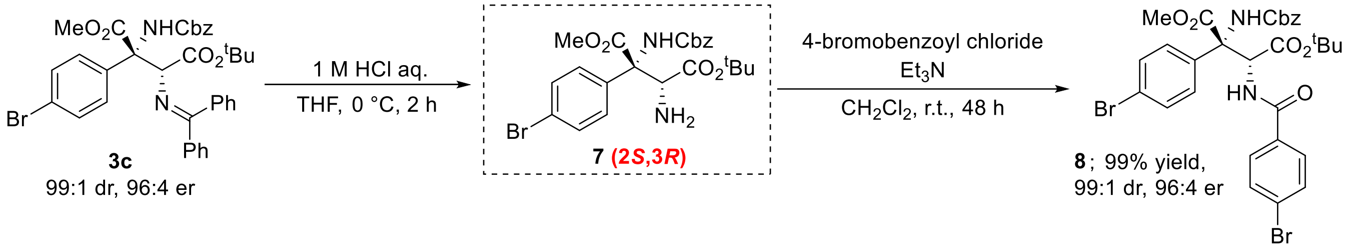

Compound **3b** (2.83 g, 4.2 mmol) was dissolved in 1 M HCl/ THF and stirred at 0 °C. After 2 h, the mixture was neutralized by adding saturated aqueous $NaHCO_3$ and then extracted by EtOAc. The organic phase was separated, dried over Na_2SO_4, and concentrated to give **7** (2.13 g, 99% yield, 99:1 dr) as a white solid. To a solution of **7** (25.7 mg, 0.06 mmol) in CH_2Cl_2 (0.6 mL) were added triethylamine (12.5 µL, 0.09 mmol) and 4-bromobenzoyl chloride (26.3 mg, 0.12 mmol) at 0 °C. After stirring at room temperature for 48 hours, the reaction mixture was extracted with CH_2Cl_2. The combined organic layers were dried over Na_2SO_4 and concentrated under reduced pressure. The residue was purified by silica-gel column chromatography (hexane: EtOAc = 75/25) to afford the product **8** (41.0 mg, 99% yield, 99:1 dr, 96:4 er) as a white solid.

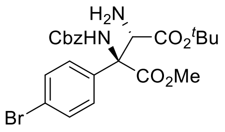

7
92% ee

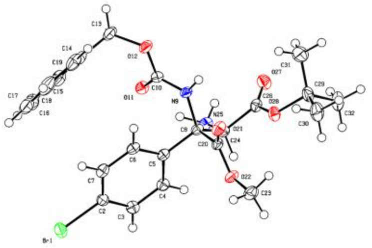

CCDC 2405070

Empirical Formula	$C_{23}H_{27}N_2O_6Br$
Formula weight	507.37
Temperature/K	103.15
Crystal system	monoclinic
Space group	$P2_1$

a/Å	5.9824(2)
b/Å	17.4162(5)
c/Å	11.7109(4)
α/º	90
β/º	104.150
γ/º	90
Volume/Å^3	1183.15(7)
Z	2
ρcalcg/cm^3	1.424
μ/mm^{-1}	2.714
F(000)	524.0
Crystal size/mm^3	0.079 × 0.03 × 0.022
Radiation	CuKα (λ = 1.54184)
2Θ range for data collection/°	7.786 to 152.058
Index ranges	-7 ≤ h ≤ 7, -15 ≤ k ≤ 21, -13 ≤ l ≤ 14
Reflections collected	11608
Independent reflections	3698 [R_{int} = 0.0408, R_{sigma} = 0.0450]
Data/restraints/parameters	3698/1/299
Goodness-of-fit on F2	1.067
Final R indexes [I>=2σ (I)]	R_1 = 0.0280, wR_2 = 0.0700
Final R indexes [all data]	R_1 = 0.0296, wR_2 = 0.0707
Largest diff. peak/hole / e Å-3	0.40/-0.73
Flack parameter	-0.025(11)

9 ESC-Mass analysis

9.1 Conformation of dinuclear catalysts

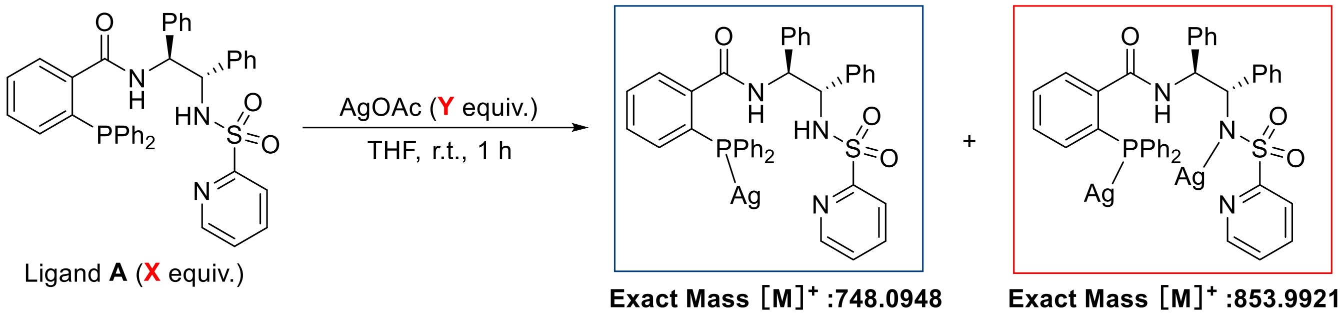

We confirmed that the dinuclear complex was formed during the reaction progression by ESI-Mass analysis (mobile phase; THF). The experiment was performed by mixing ligand **A** and silver acetate in a **1:1** or **1:2** ratio in THF. It was confirmed that the peak of the dinuclear complex was small in the ligand:silver=1:1, and the peak of the mononuclear complex was mainly obtained, whereas when ligand: silver = 1:2, it was confirmed that the peak of the dinuclear complex was observed to the same extent as the peak of the mononuclear complex.

A) Ligand (1.0 equiv.) : AgOAc (1.0 equiv.)

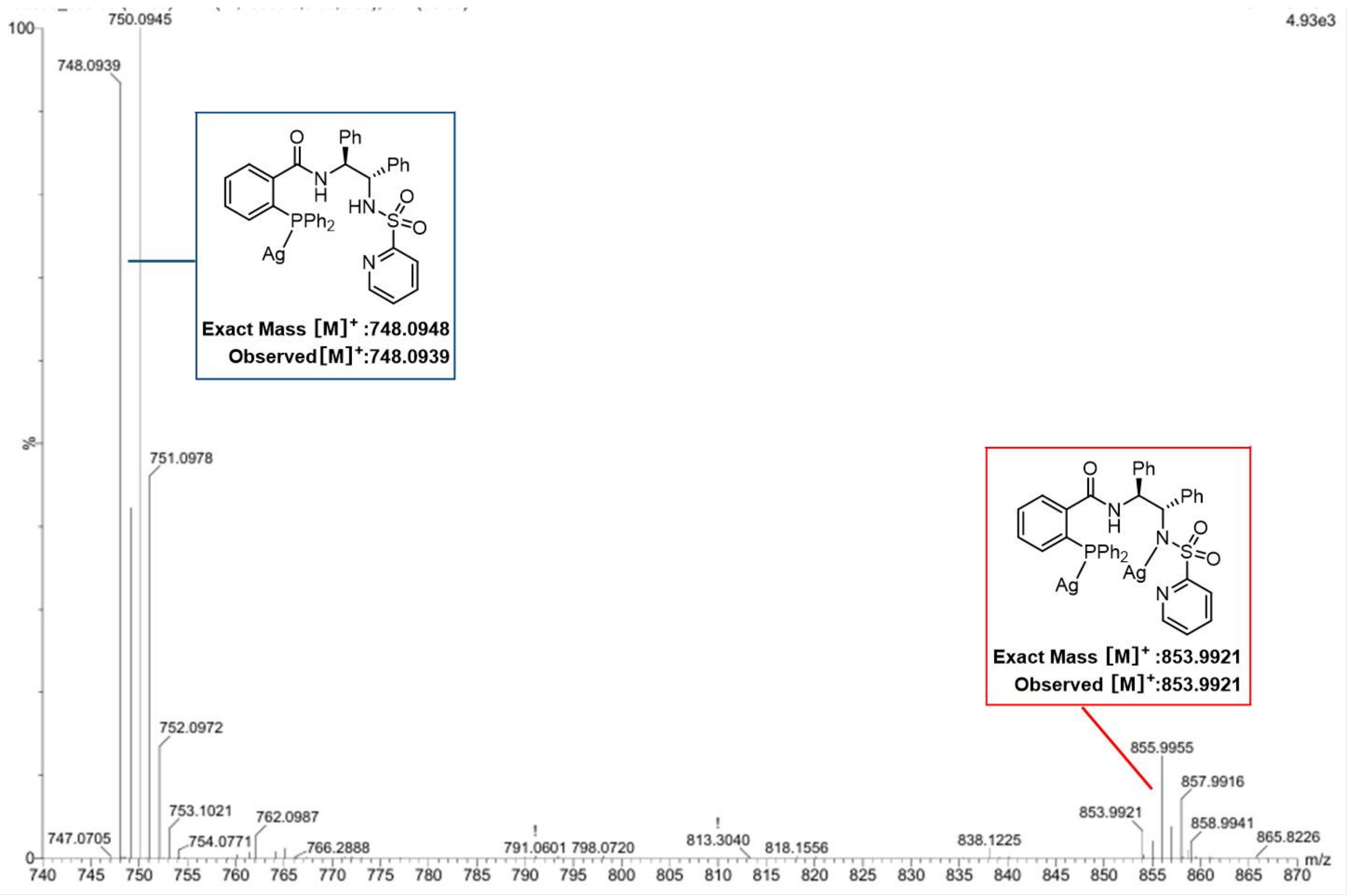

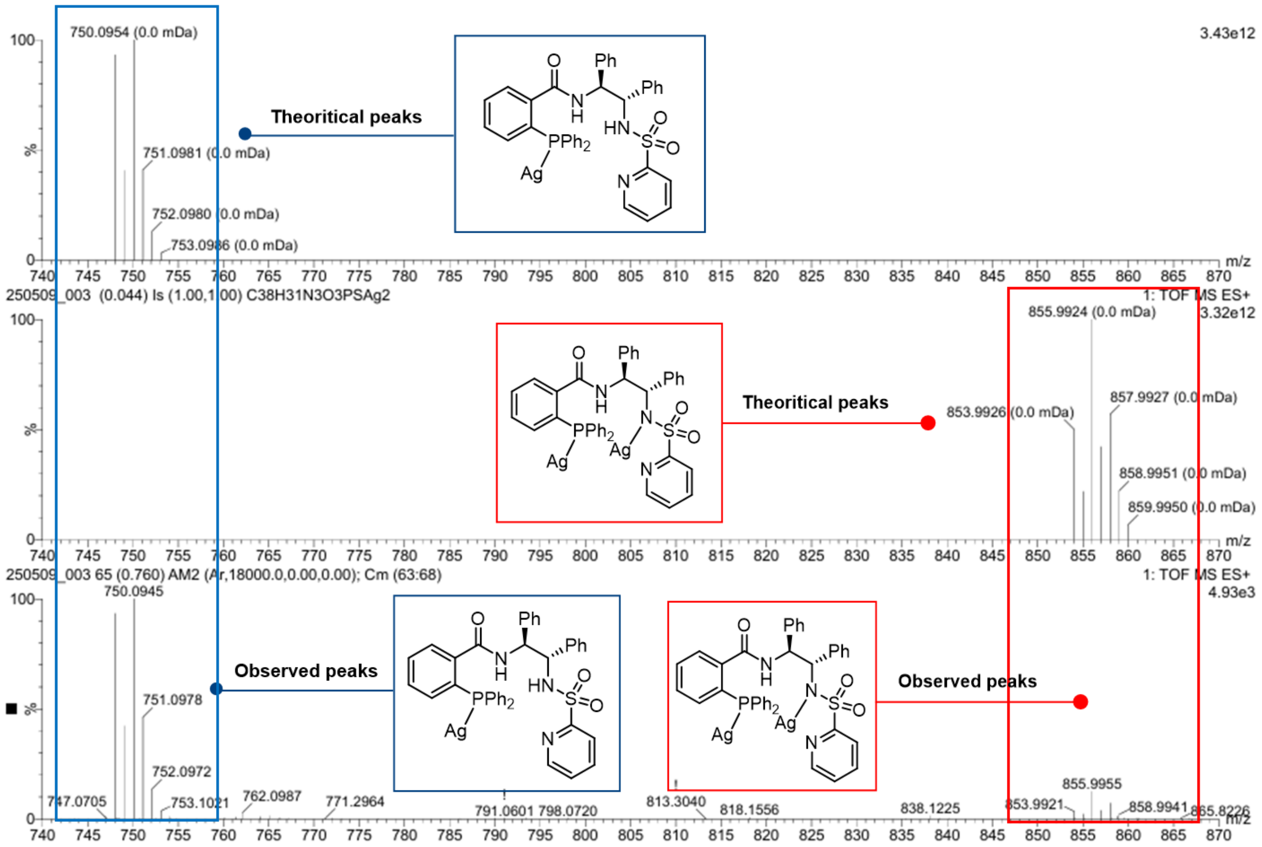

B) Ligand (1.0 equiv.) : AgOAc (2.0 equiv.)

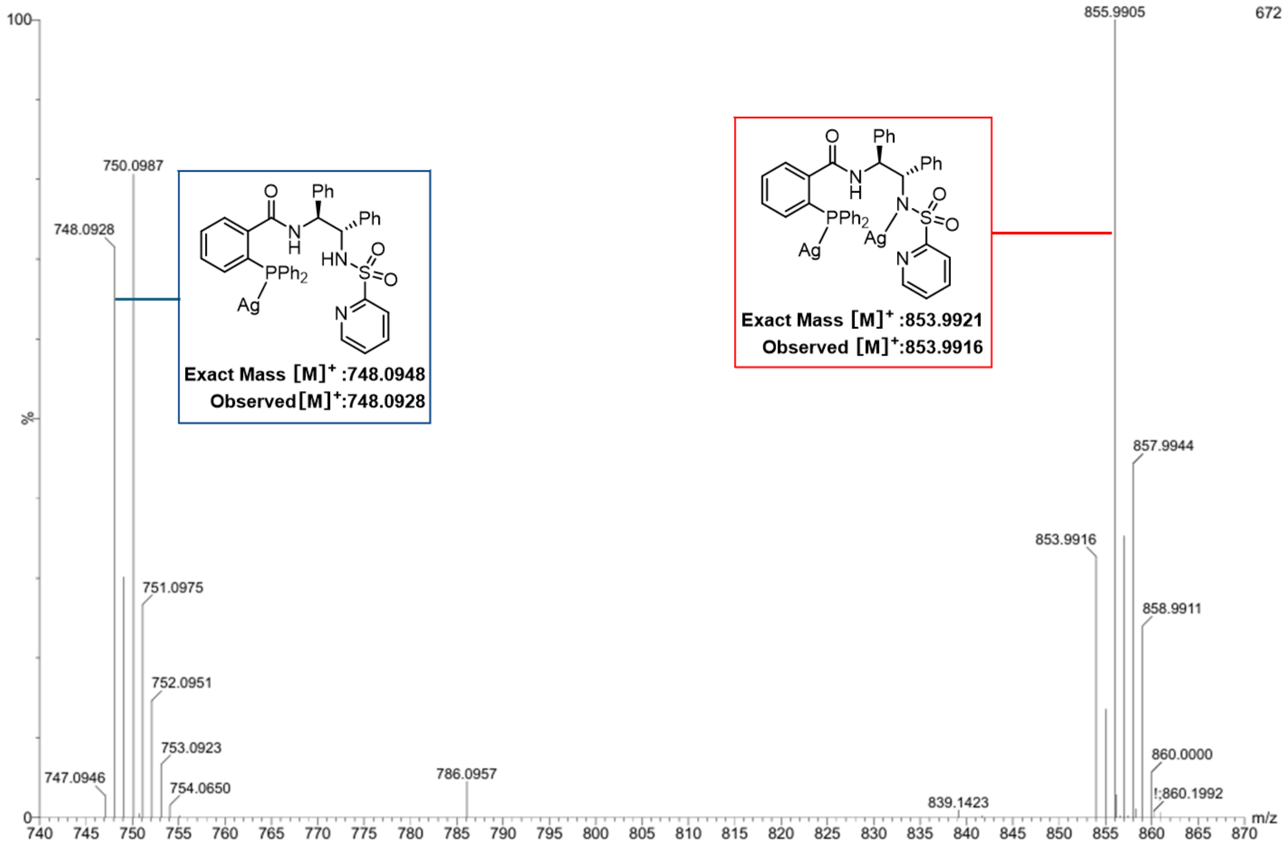

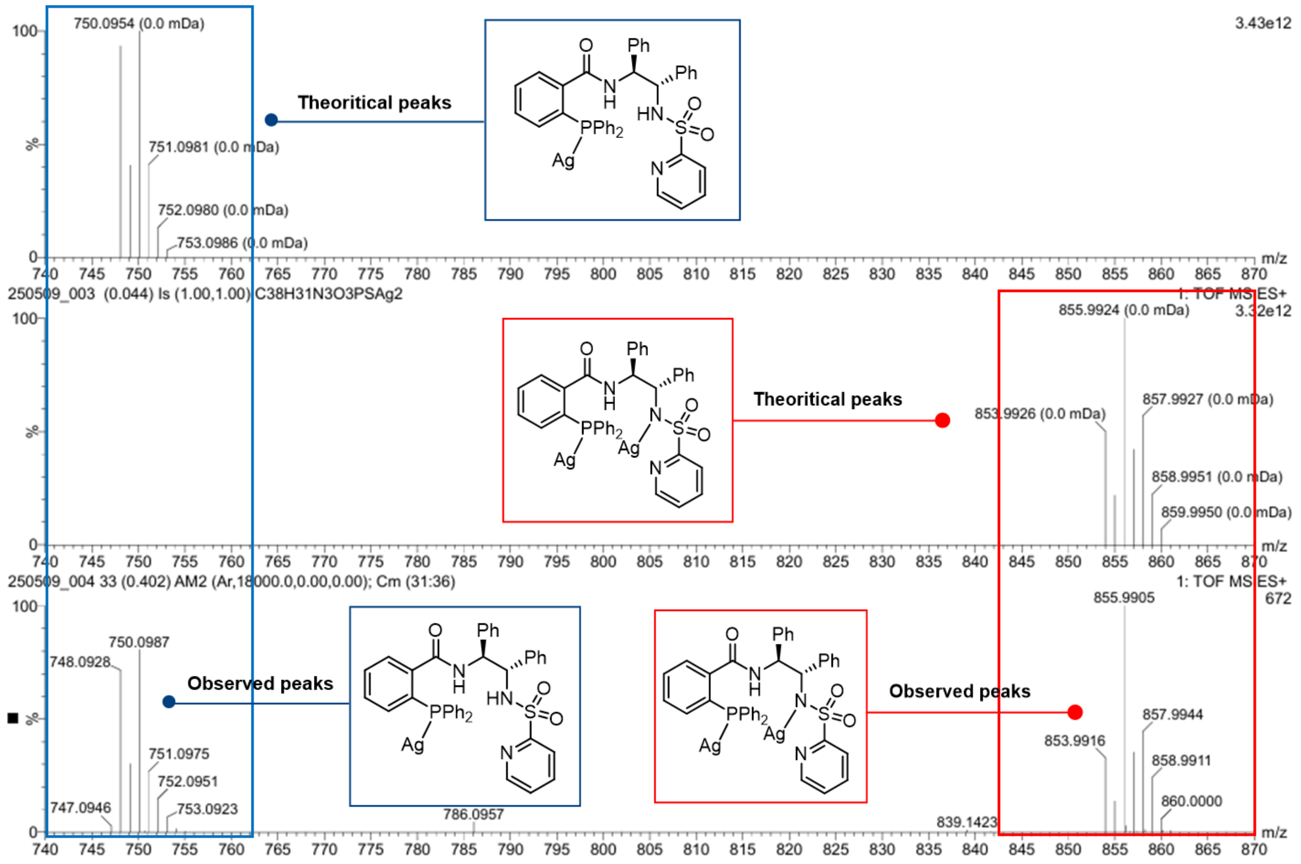

9.2 Complex of dinuclear catalysts and shiff base 2

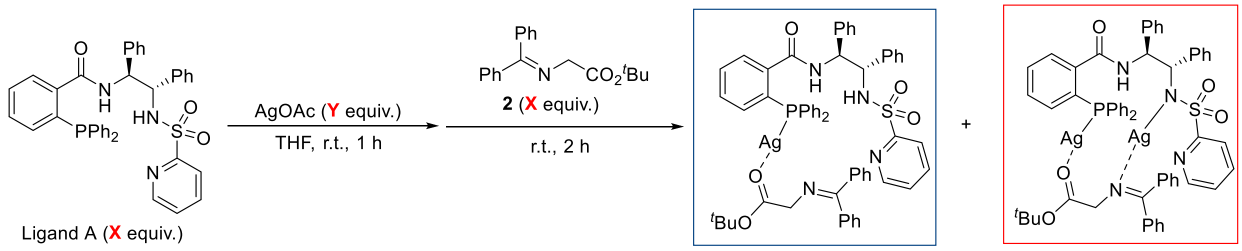

Exact Mass [M]$^+$:1043.2508 Exact Mass [M]$^+$:1149.1481

We also confirmed that the complex of dinuclear complexes and glycinate Schiff base was formed during the reaction progression by ESI-Mass analysis (mobile phase; THF). The experiment was performed by mixing ligand **A** and silver acetate in a **1:1** or **1:2** ratio in THF. After stirring for 1 h at room temperature, Schiff base **2** (1.0 equiv. to ligand) was added. Then, the reaction mixture was stirred for 2 h at room temperature. It was confirmed that the peak of the dinuclear complex was small in the (ligand:silver=1:1) + Schiff base **2**, and the peak of the complex of mononuclear and Schiff base **2** was obtained to the same extent as the peak of the complex of (ligand:silver=1:2) + Schiff base **2**, whereas when (ligand:silver=1:2) + Schiff base **2**, the peak of the (ligand:silver=1:2) + Schiff base **2** was mainly obtained.

A) Ligand (1.0 equiv.) : AgOAc (1.0 equiv.)

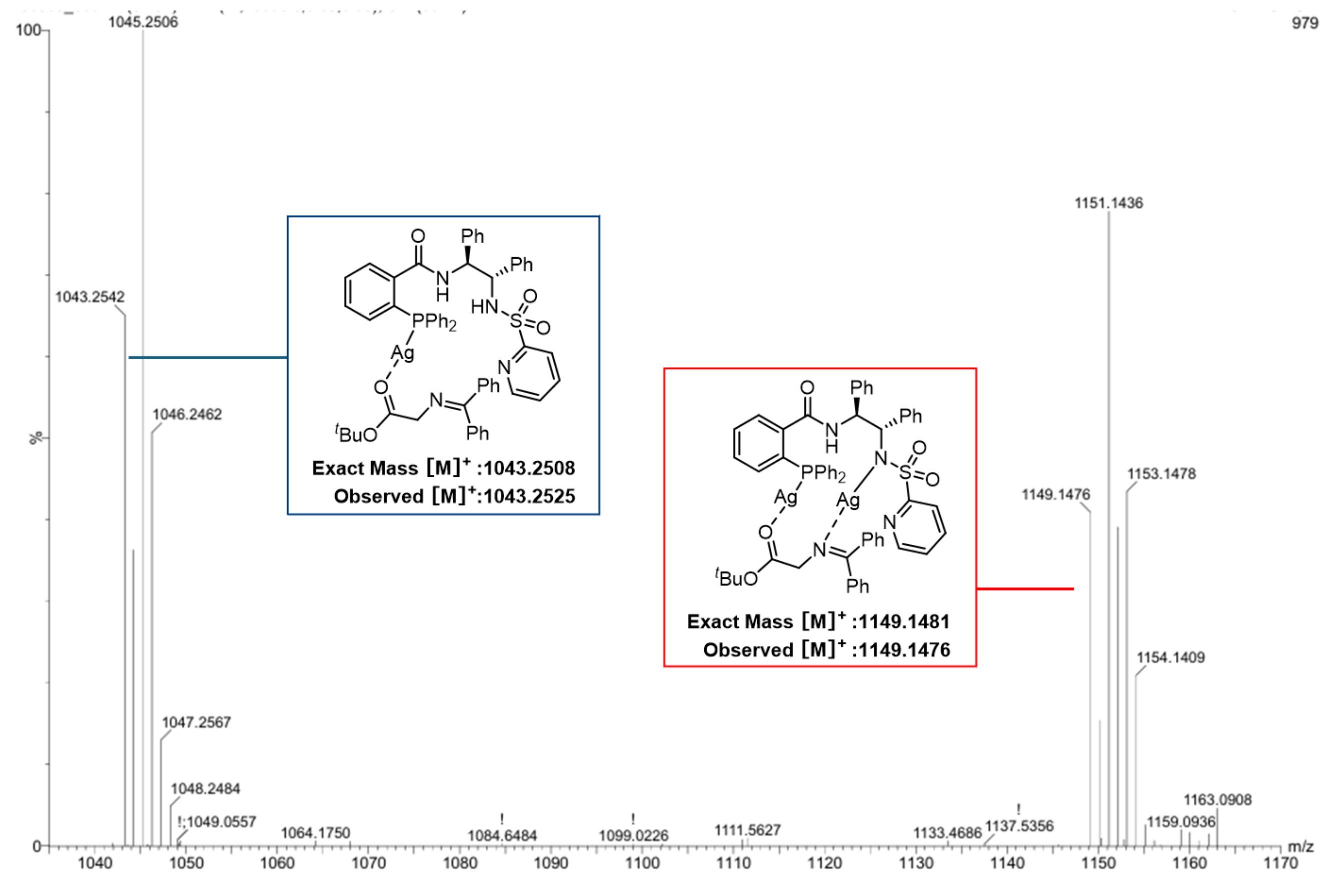

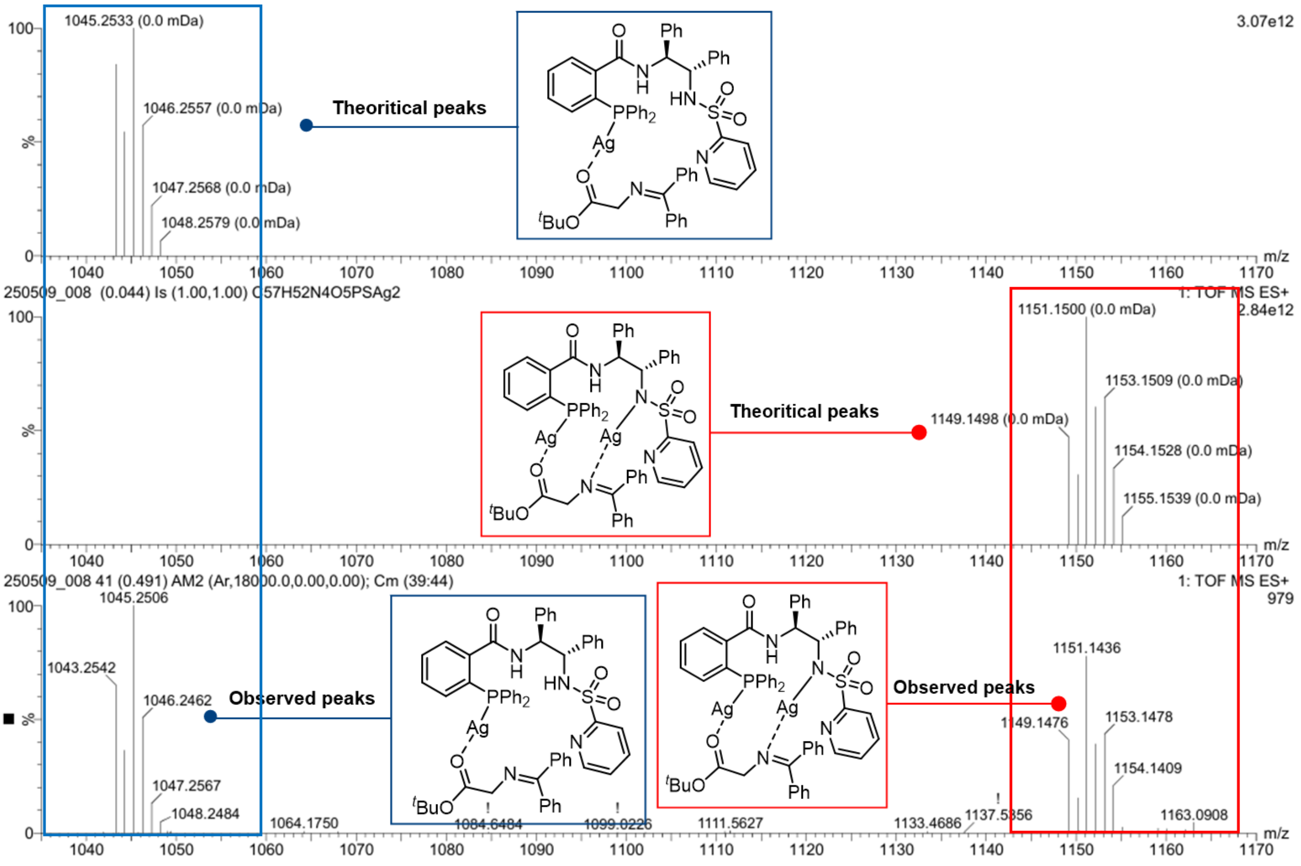

B) Ligand (1.0 equiv.) : AgOAc (2.0 equiv.)

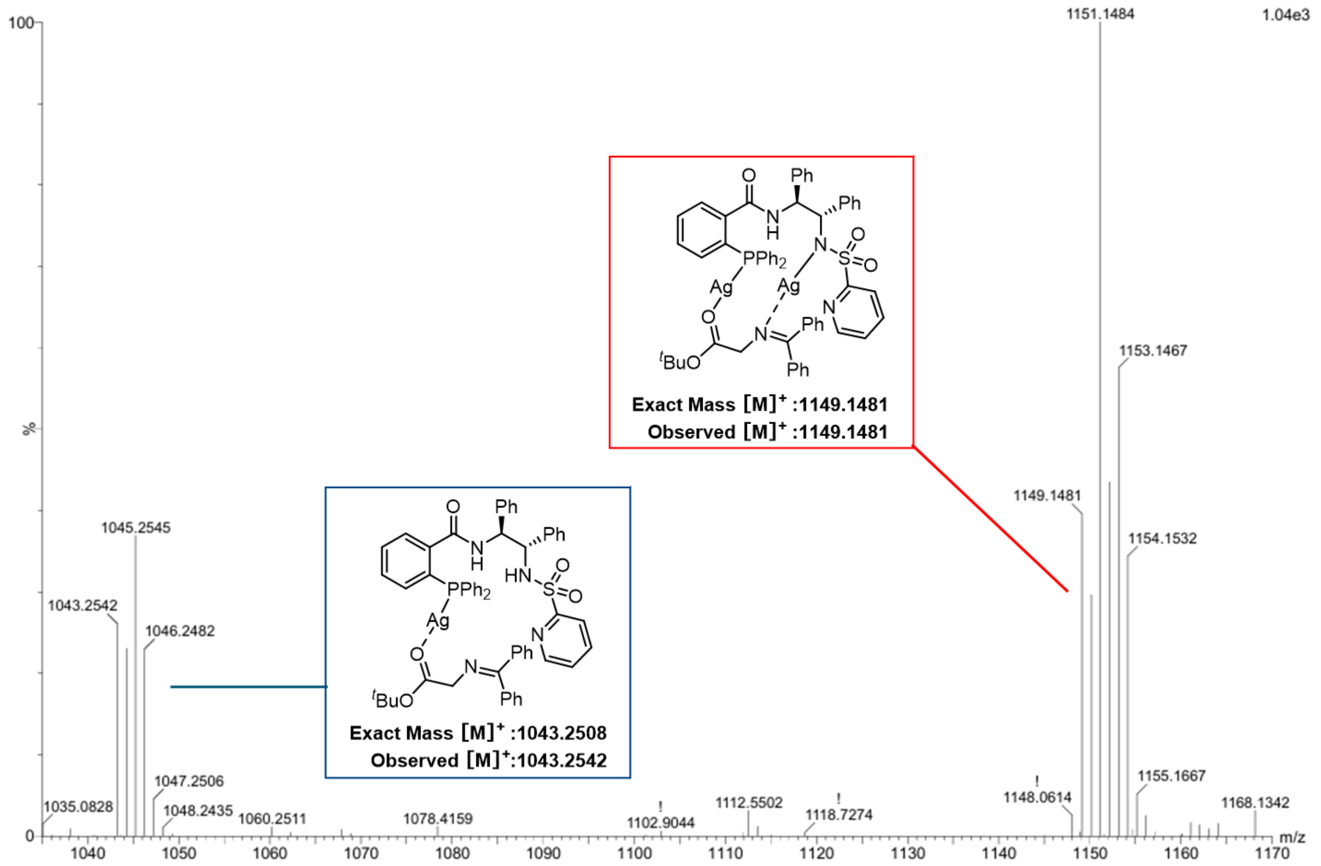

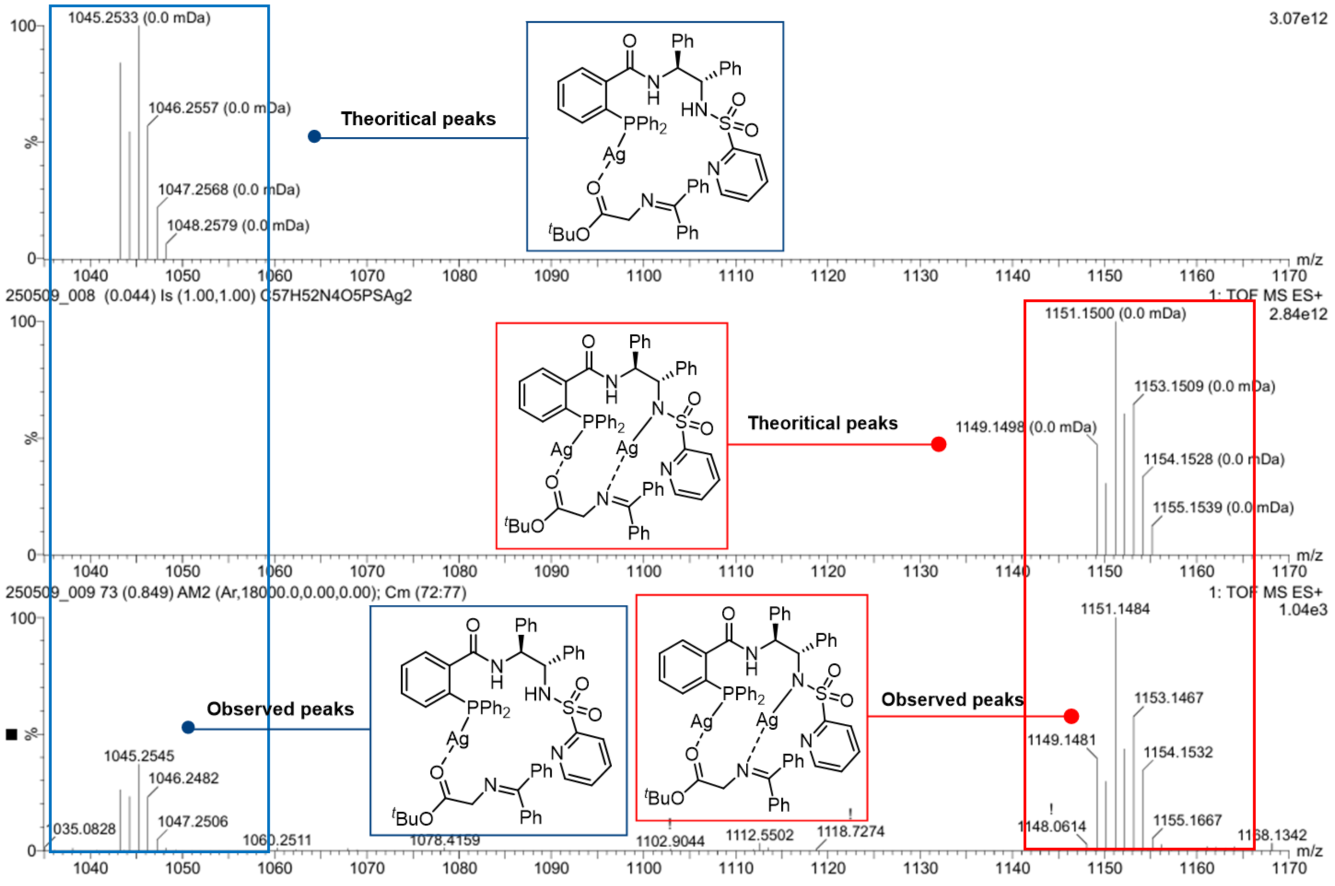

9.3 **Reaction mixture**

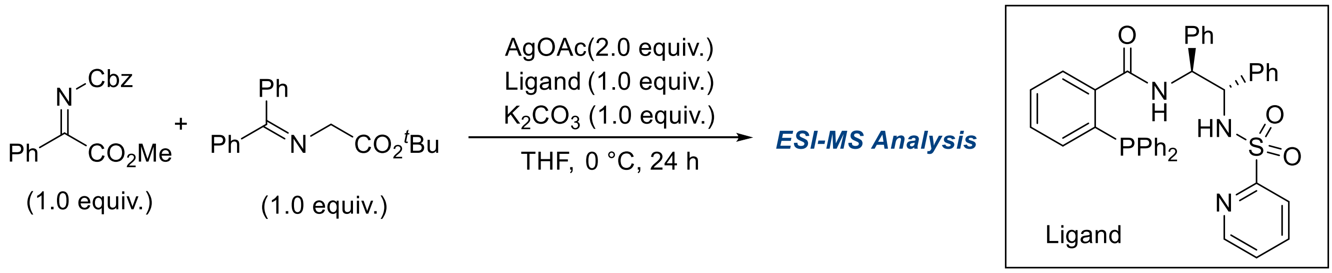

Observed ESI-MS

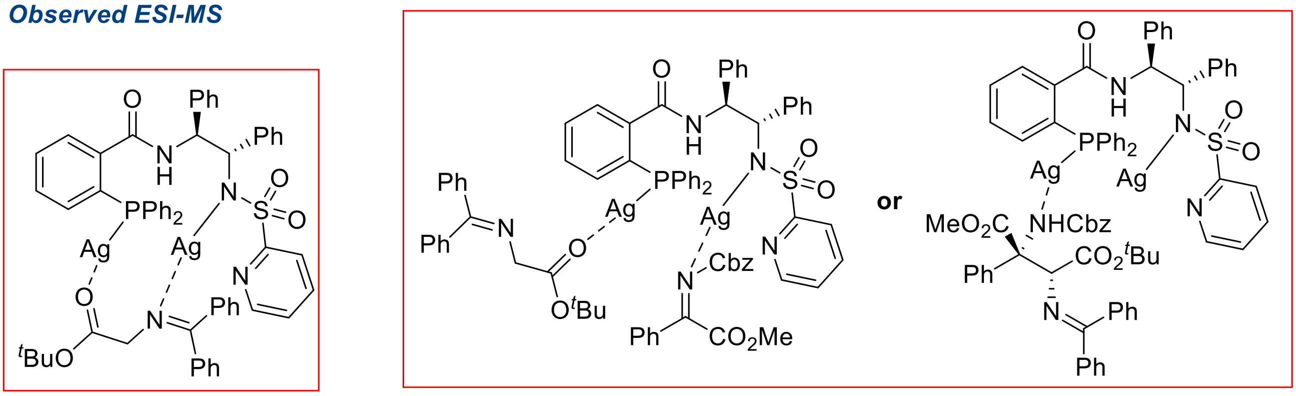

Exact Mass [M]⁺ :1149.1481

Exact Mass [M]⁺ :1446.2494

We performed the reaction mixture by ESI-Mass analysis (mobile phase; MeOH). The experiment was performed by mixing ligand **A** and silver acetate in a 1:2 ratio in THF. After stirring for 1 h at room temperature, ketimine **1a** (1.0 equiv. to ligand), Schiff base **2** (1.0 equiv. to ligand), and K_2CO_3 (1.0 equiv. to ligand) were added. Then, the reaction mixture was stirred for 24 h at 0 °C. It was confirmed that the peak of the (ligand:silver=1:2) + Schiff base **2** was mainly obtained. Also, the peak of the (ligand:silver=1:2) + ketimine **1a** + Schiff base **2** (or (ligand:silver=1:2) + product **3a**)) was observed.

A) Full-size mass spectrum for the reaction mixture

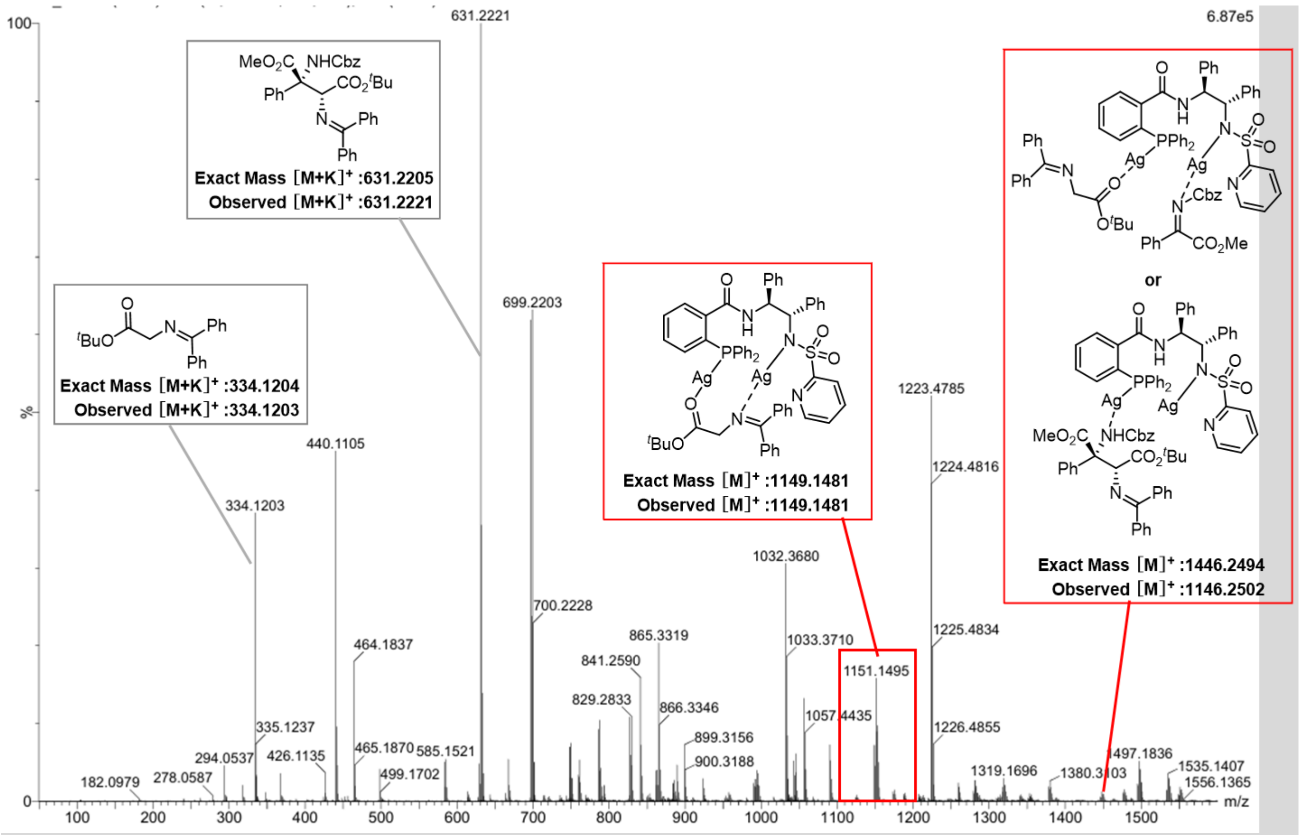

B) Bimetallic catalyst + Schiff base 2

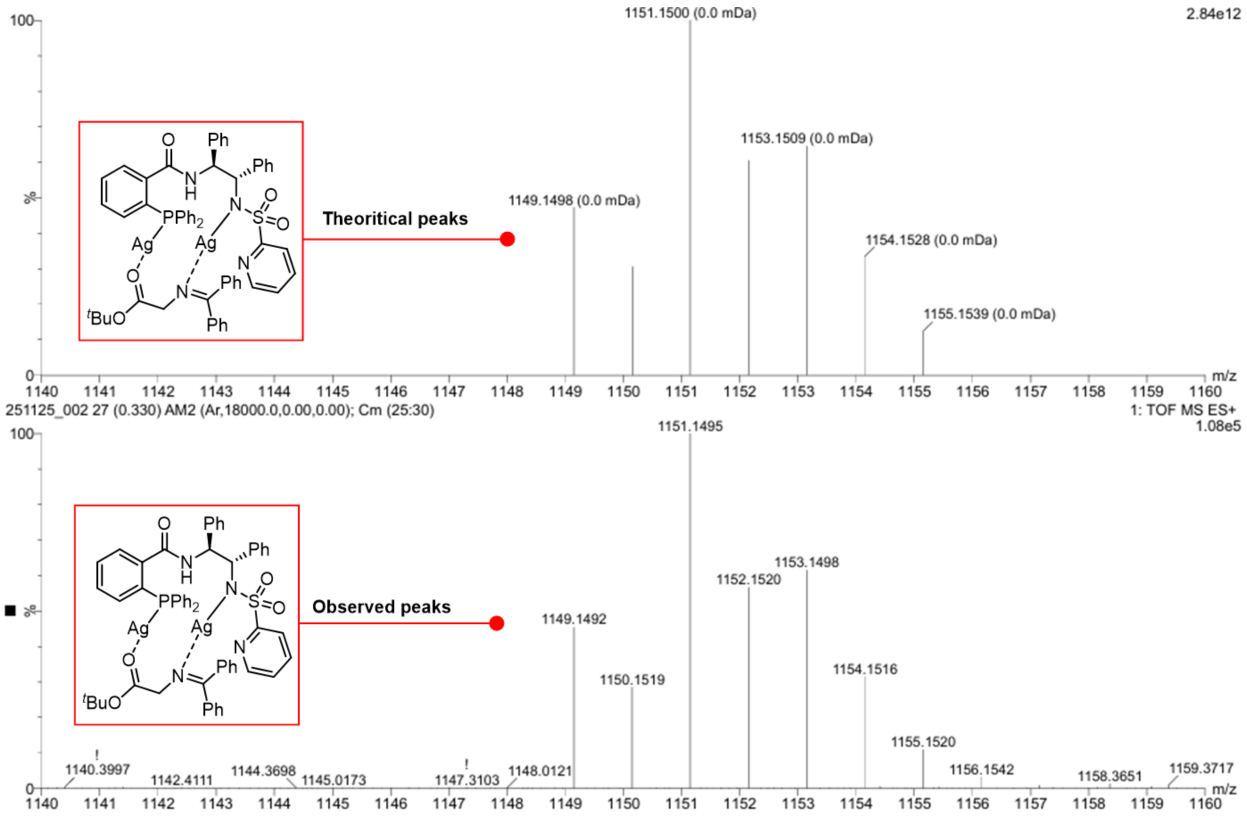

C) Bimetallic catalyst + ketimine 1a + Schiff base 2 (or Bimetallic catalyst + product 3a)

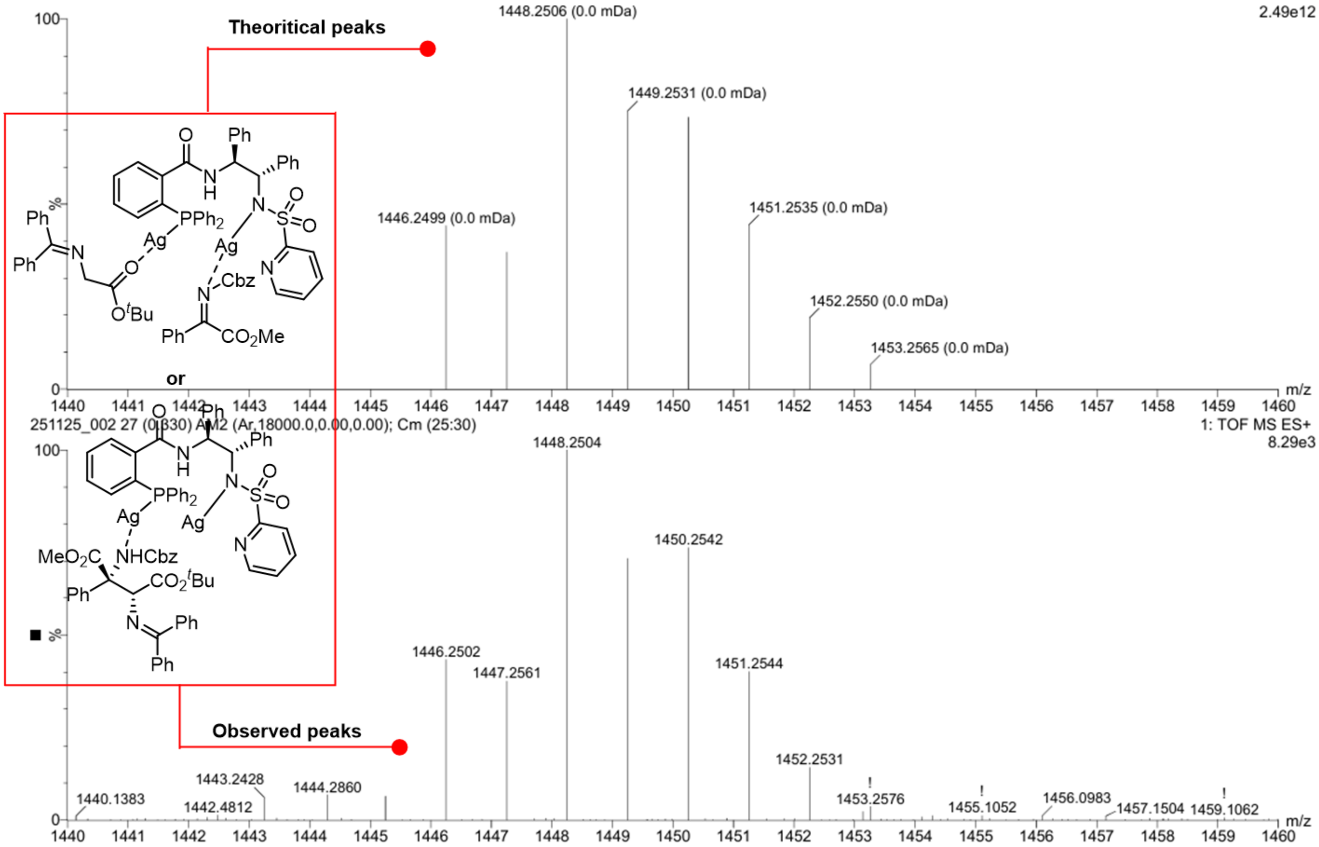

10. References

1) (a) Ligand **J**; Y. Xiong, Z. Du, H. Chen, Z. Yang, Q. Tan, C. Zhang, L. Zhu, Y. Lan, M. Zhang, *J. Am. Chem. Soc.* **2019**, *141*, 961. (b) Ligand **K**; Y. Iizuka, K. Obata, T. Takehara, T. Suzuki, S. Nakamura, *Adv. Synth. Catal*. **2024**, *366*, 4410.

2) F. Cuccu, A. Porcheddu, *Green Chem.* **2024**, *26*, 2684.

3) T. Takeda, M. Terada, *J. Am. Chem. Soc.* **2013**, *135*, 15306.

4) T. Takeda, M. Terada, *Aust. J. Chem.* **2014**, *67*, 1124.

5) S. Maeda, K. Ohno, K. Morokuma, *Phys. Chem. Chem. Phys*. **2013**, *15*, 3683.

6) Gaussian 16, Revision C.02, M. J. Frisch, G. W. Trucks, H. B. Schlegel, G. E. Scuseria, M. A. Robb, J. R. Cheeseman, G. Scalmani, V. Barone, G. A. Petersson, H. Nakatsuji, X. Li, M. Caricato, A. V. Marenich, J. Bloino, B. G. Janesko, R. Gomperts, B. Mennucci, H. P. Hratchian, J. V. Ortiz, A. F. Izmaylov, J. L. Sonnenberg, D. Williams-Young, F. Ding, F. Lipparini, F. Egidi, J. Goings, B. Peng, A. Petrone, T. Henderson, D. Ranasinghe, V. G. Zakrzewski, J. Gao, N. Rega, G. Zheng, W. Liang, M. Hada, M. Ehara, K. Toyota, R. Fukuda, J. Hasegawa, M. Ishida, T. Nakajima, Y. Honda, O. Kitao, H. Nakai, T. Vreven, K. Throssell, J. A. Montgomery, Jr., J. E. Peralta, F. Ogliaro, M. J. Bearpark, J. J. Heyd, E. N. Brothers, K. N. Kudin, V. N. Staroverov, T. A. Keith, R. Kobayashi, J. Normand, K. Raghavachari, A. P. Rendell, J. C. Burant, S. S. Iyengar, J. Tomasi, M. Cossi, J. M. Millam, M. Klene, C. Adamo, R. Cammi, J. W. Ochterski, R. L. Martin, K. Morokuma, O. Farkas, J. B. Foresman, D. J. Fox, Gaussian, Inc., Wallingford CT, **2016**

7) C. Bannwarth, S. Ehlert, S. Grimme, *J. Chem. Theory Comput.* **2019**, *15*, 1652.

8) CYLview, 1.0b; Legault, C. Y. Université de Sherbrooke, 2009 http://www.cylview.org

9) NBO 7.0: E. D. Glendening, J. K. Badenhoop, A. E. Reed, J. E. Carpenter, J. A. Bohmann, C. M. Morales, P. Karafiloglou, C. R. Landis, and F. Weinhold, Theoretical Chemistry Institute, University of Wisconsin, Madison, WI (2018)

10) (a) E. R. Johnson, S. Keinan, P. Mori-Sánchez, J. Contreras-García, A. J. Cohen, W. Yang, *J. Am. Chem. Soc*. **2010**, *132*, 6498. (b) T. Lu, F. Chen, *J. Comput. Chem.* **2012**, *33*, 580.

11) W. Humphrey, A. Dalke, K. Schulten, *J. Mol. Graph.* **1996**, *14*, 33.

12) (a) Y. Qian, C. Jing, C. Zhai, W. H. Hu, *Adv. Synth. Catal.* **2012**, *354*, 301. (b) W. Yan, D. Wang, J. Feng, P. Li, D. Zhao, R. Wang, *Org. Lett.* **2012**, *14*, 2512. (c) M. Hatano, H. Okamoto, T. Kawakami, K. Toh, H. Nakatsuji, A. Sakakura, K. Ishihara, *Chem. Sci.* **2018**, *9*, 6361. (d) K. Ogura, T. Takehara, T. Suzuki, S. Nakamura, *Adv. Synth. Catal.* **2021**, *363*, 4544. (e) R. A. Kovalevsky, K. V. Vasechkin, A. S. Kucherenko, S. G. Zlotin, *Adv. Synth. Catal.* **2023**, *365*, 3162.

13) Y. Oyamada, M. Fujii, T. Takehara, T. Suzuki, S. Nakamura, *ACS Catal.* **2024**, *14*, 3411.

11. NMR spectrum

A ^{1}H NMR (CDCl$_3$, 300 MHz)

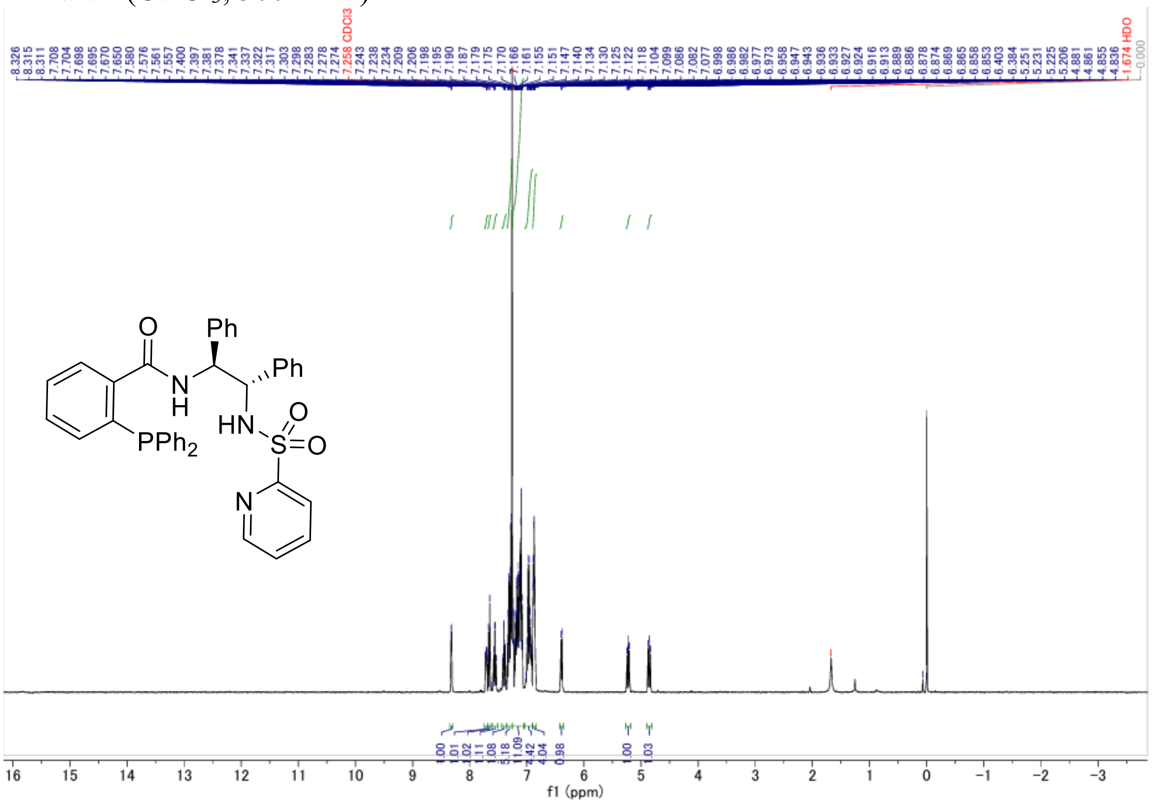

A ^{13}C NMR (CDCl$_3$, 176 MHz)

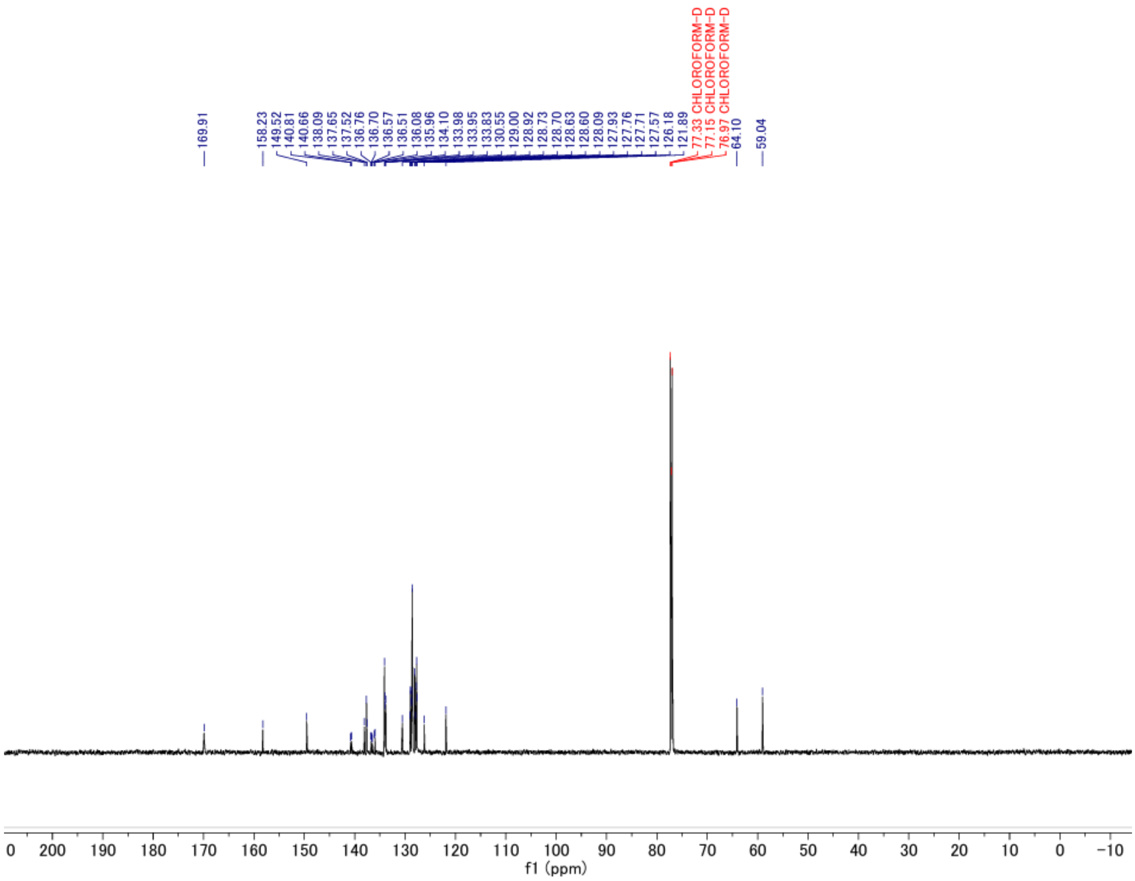

A ^{31}P NMR (CDCl$_3$, 121 MHz)

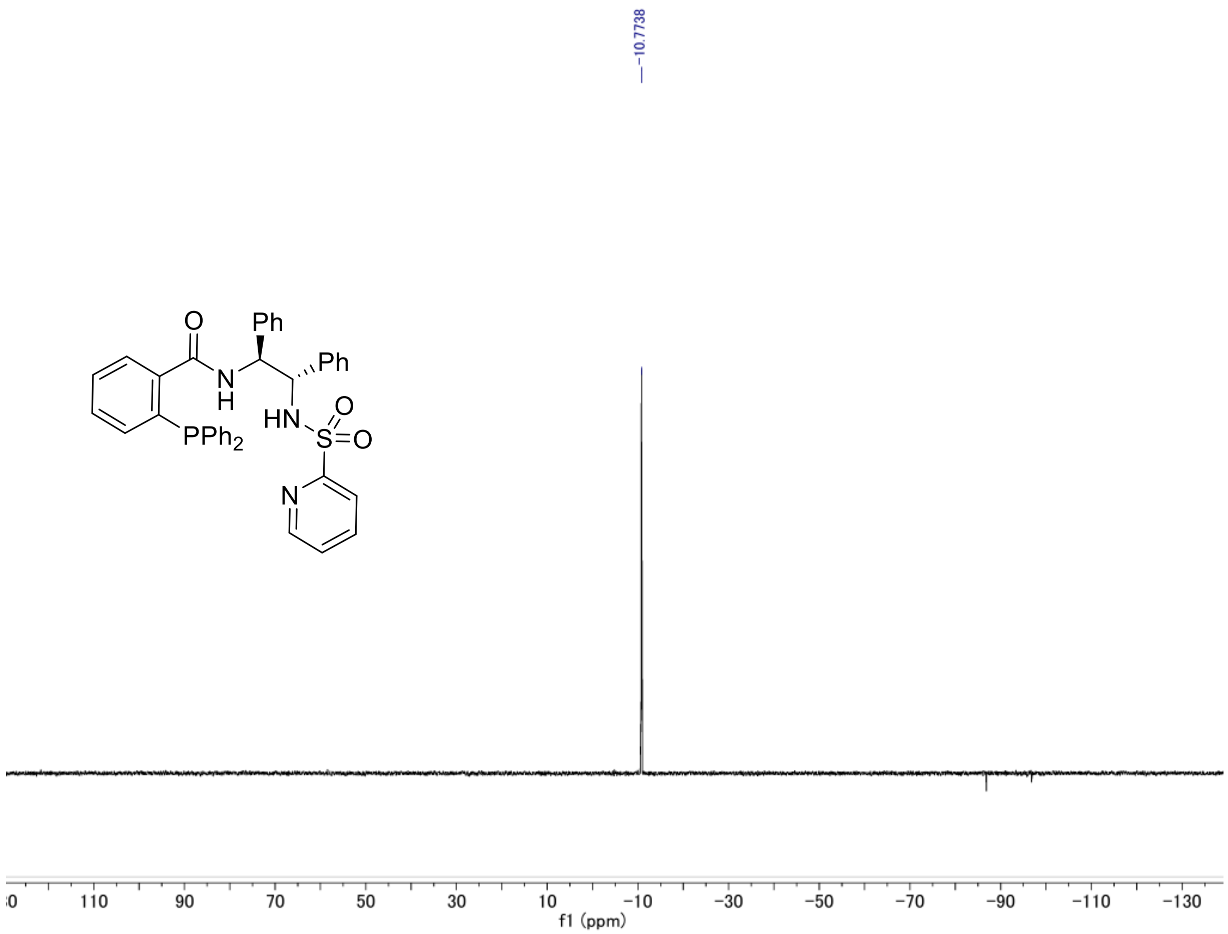

B ^{1}H NMR ($CDCl_3$, 300 MHz)

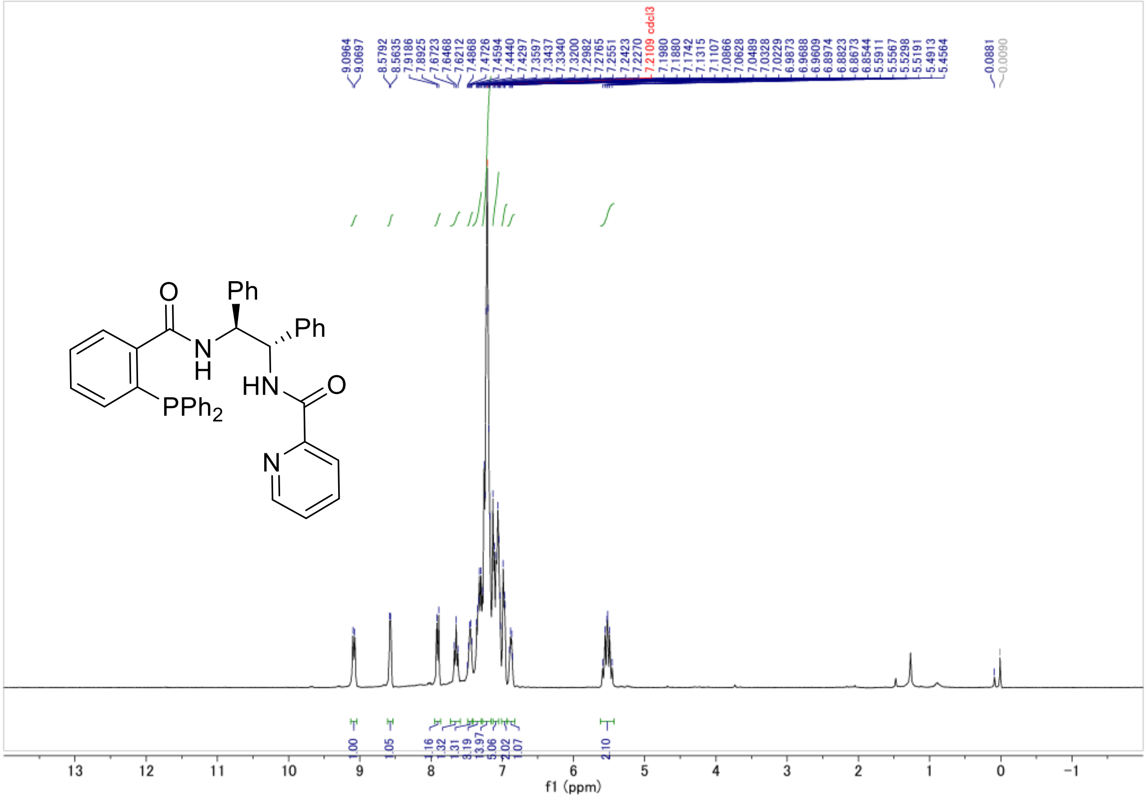

B ^{13}C NMR ($CDCl_3$, 125 MHz)

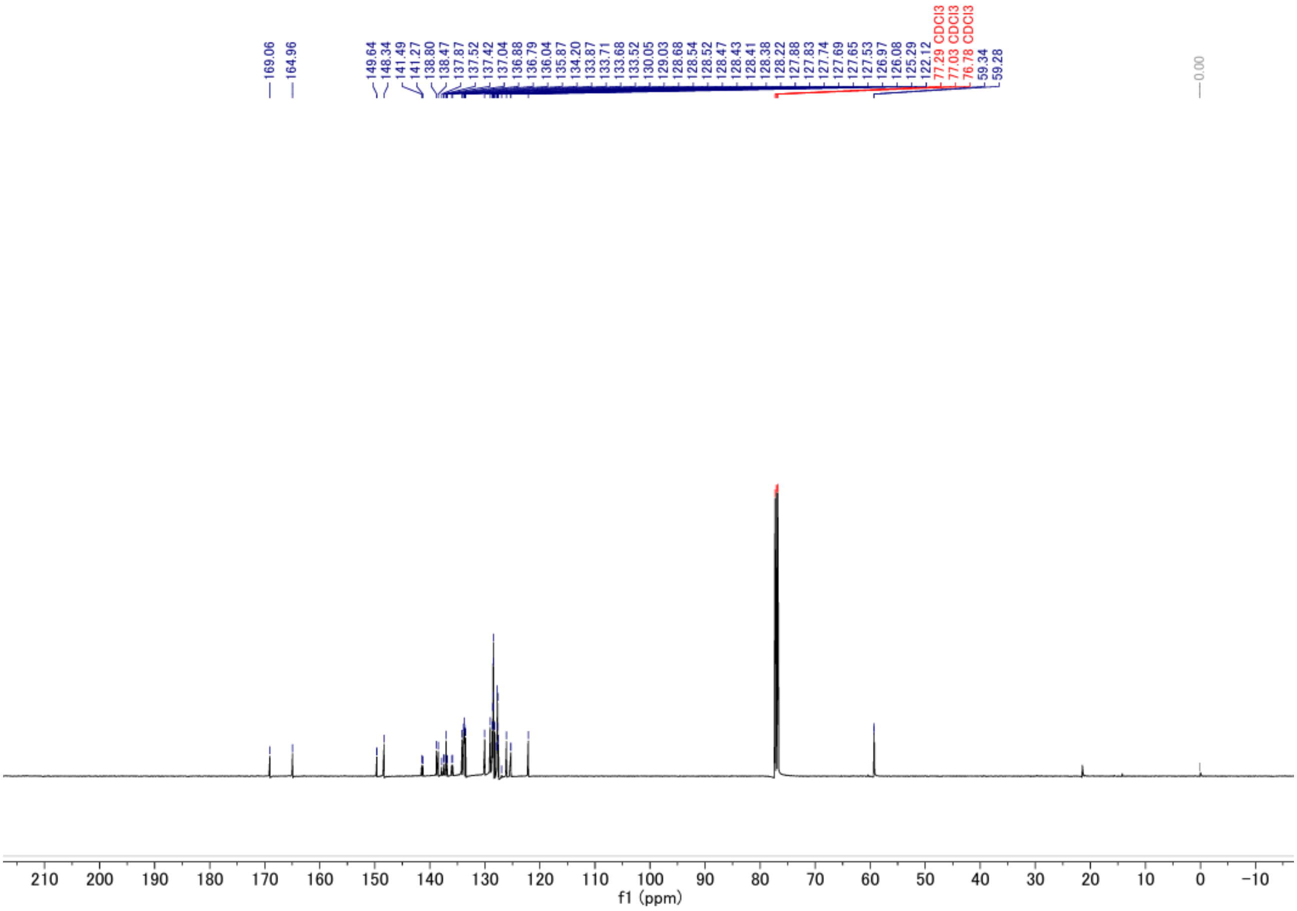

B ^{31}P NMR (CDCl$_3$, 121 MHz)

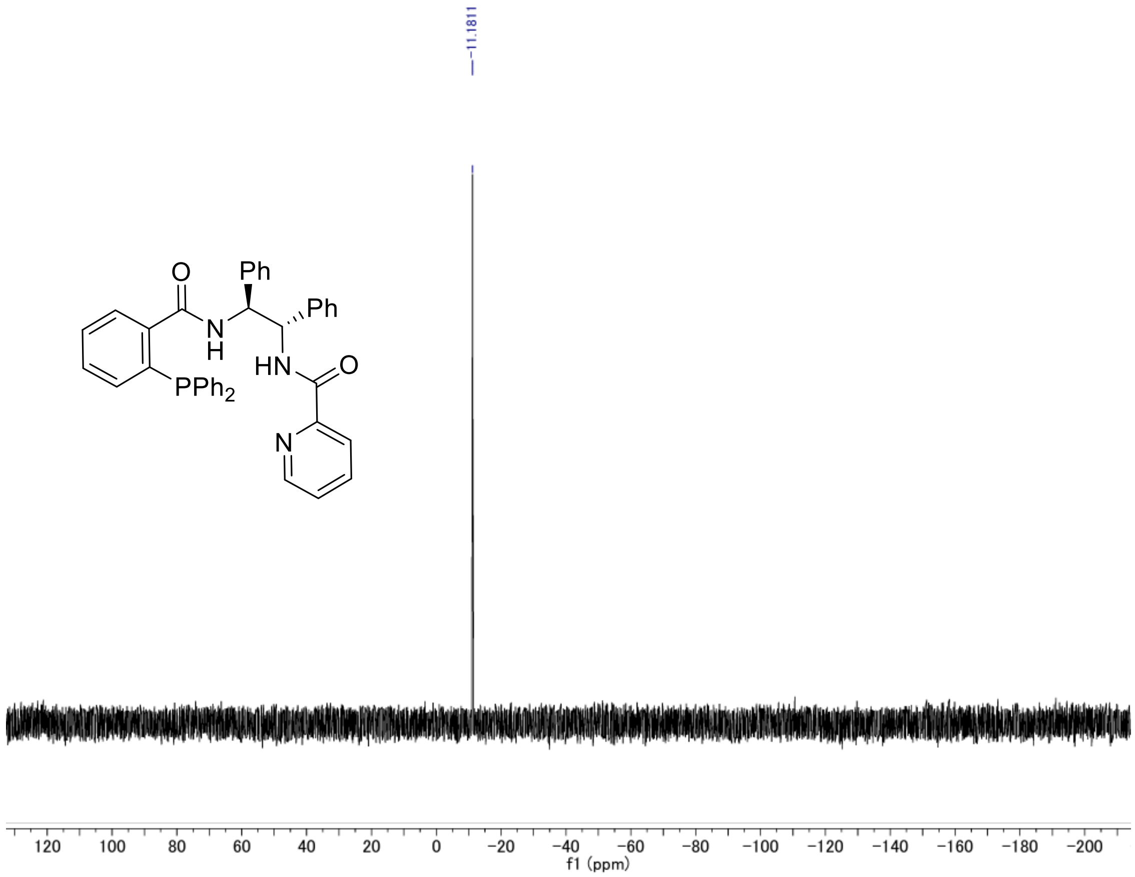

C ^{1}H NMR (CDCl$_3$, 400 MHz)

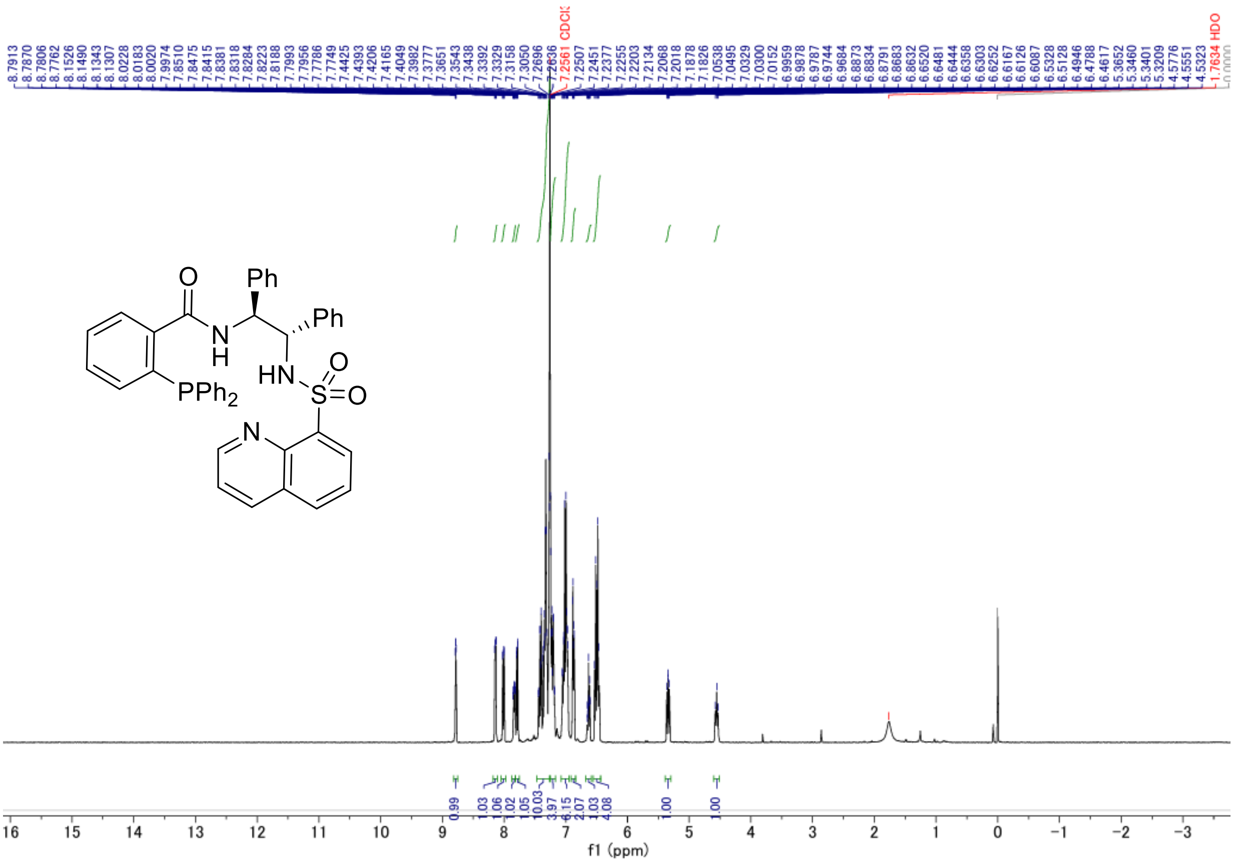

C ^{13}C NMR (CDCl$_3$, 176 MHz)

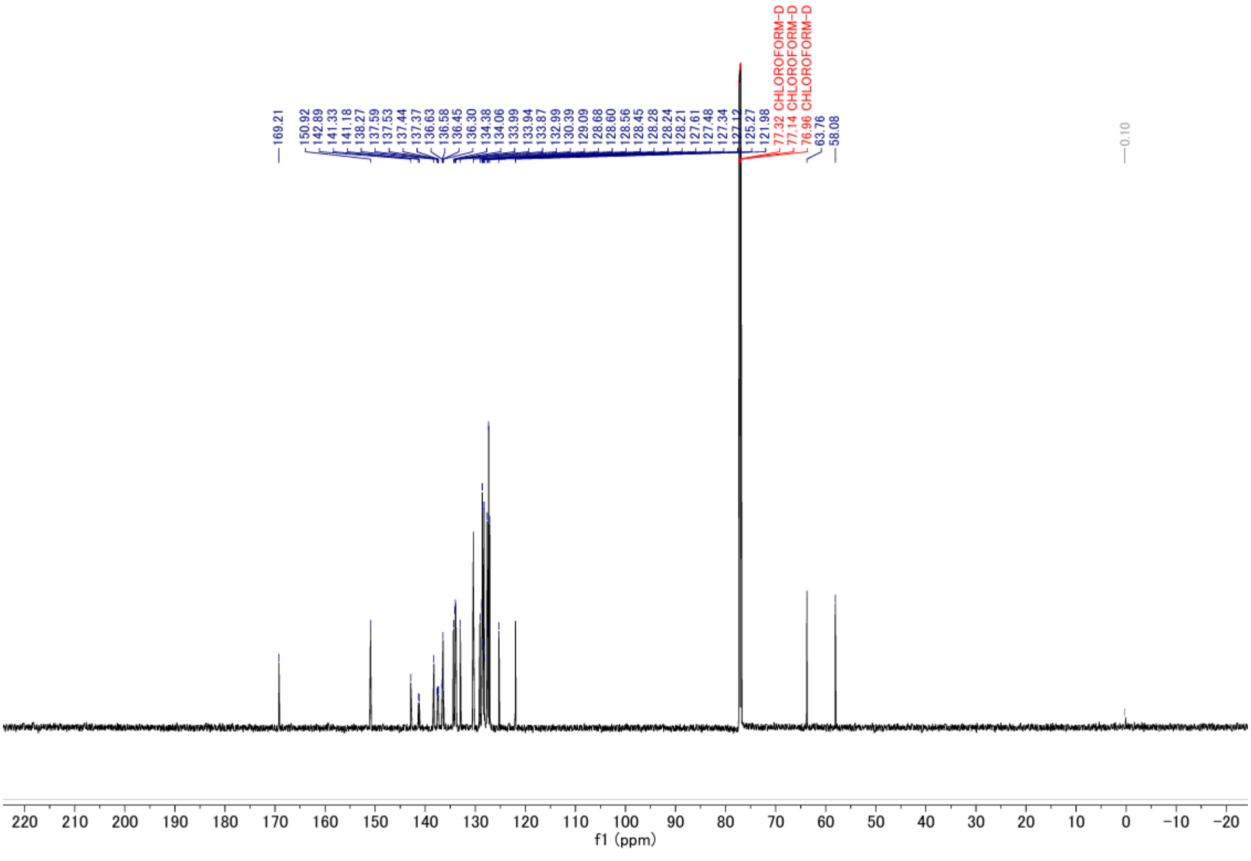

C ^{31}P NMR ($CDCl_3$, 283 MHz)

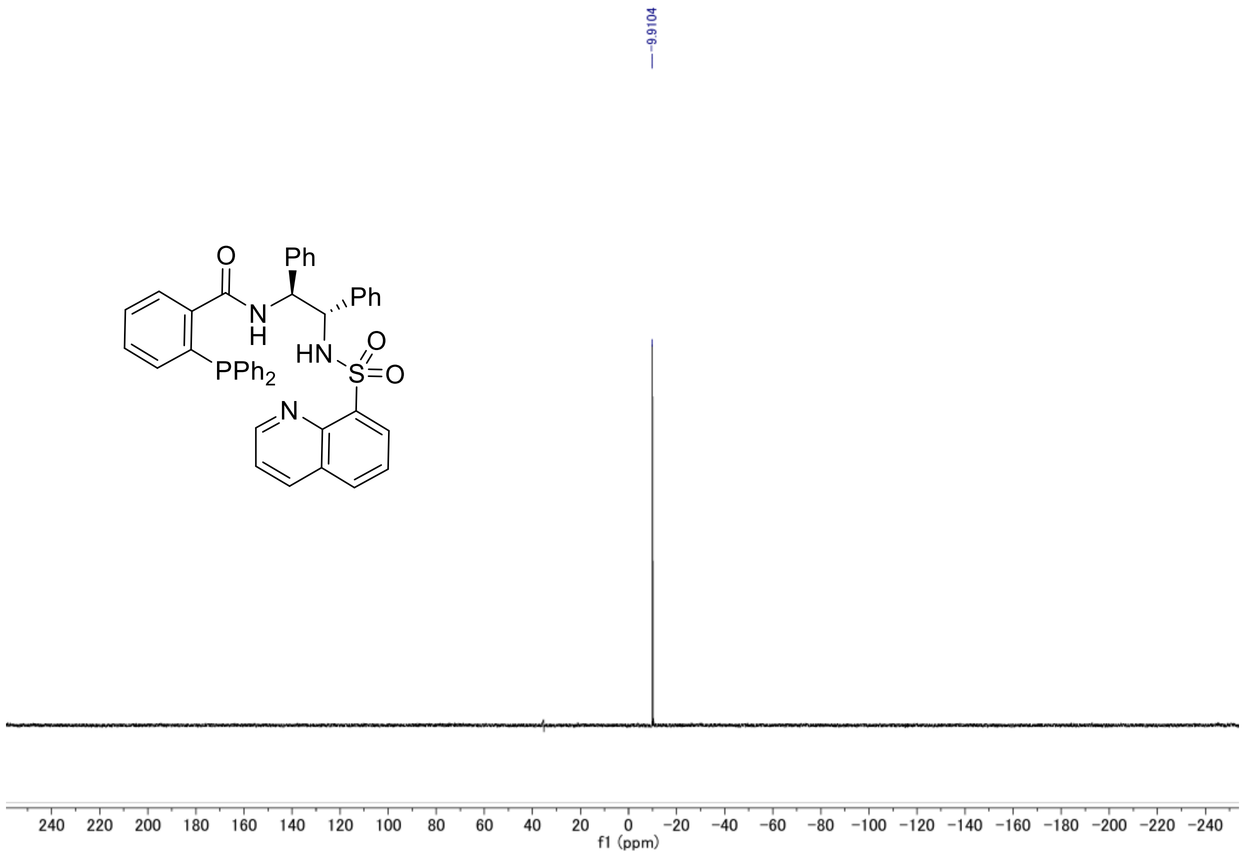

D ^{1}H NMR ($CDCl_3$, 400 MHz)

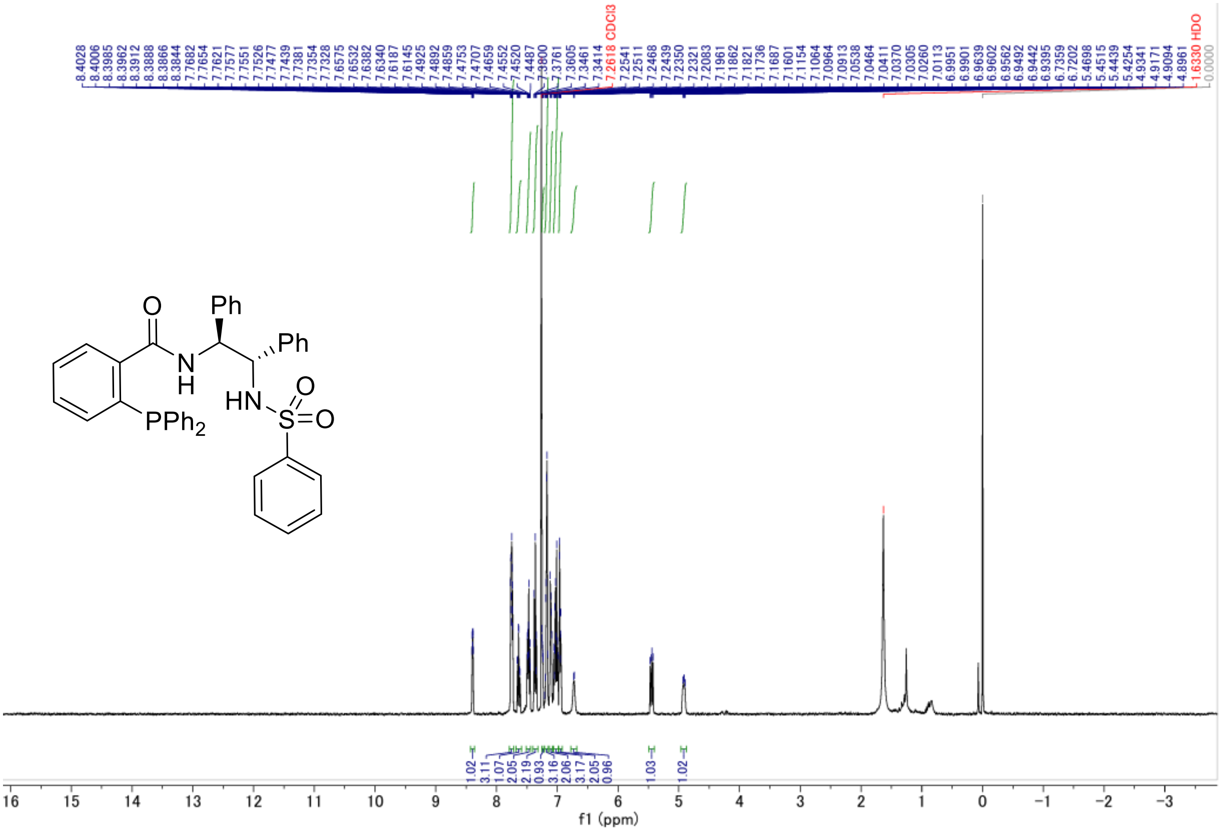

D ^{13}C NMR ($CDCl_3$, 176 MHz)

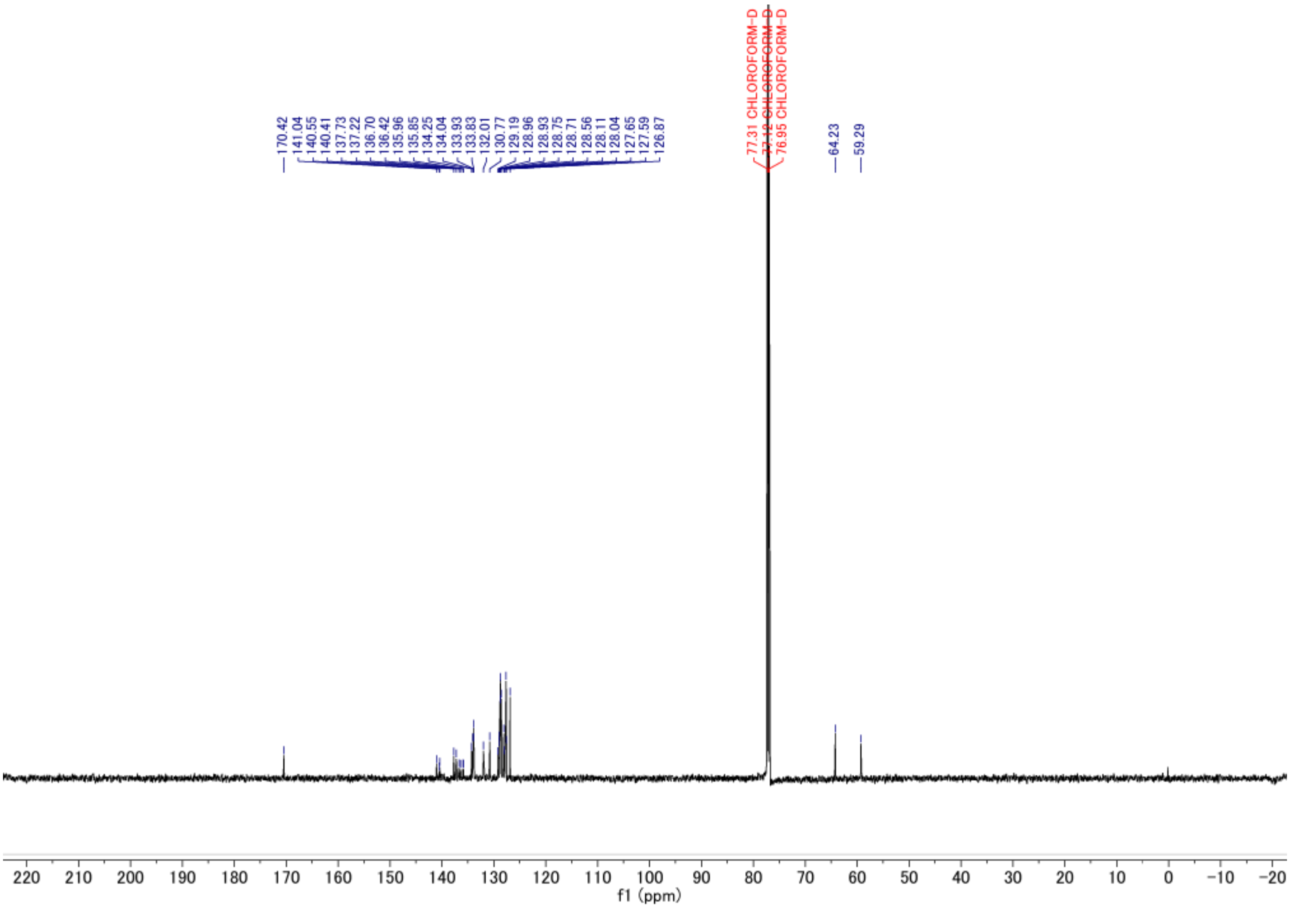

D ^{31}P NMR ($CDCl_3$, 283 MHz)

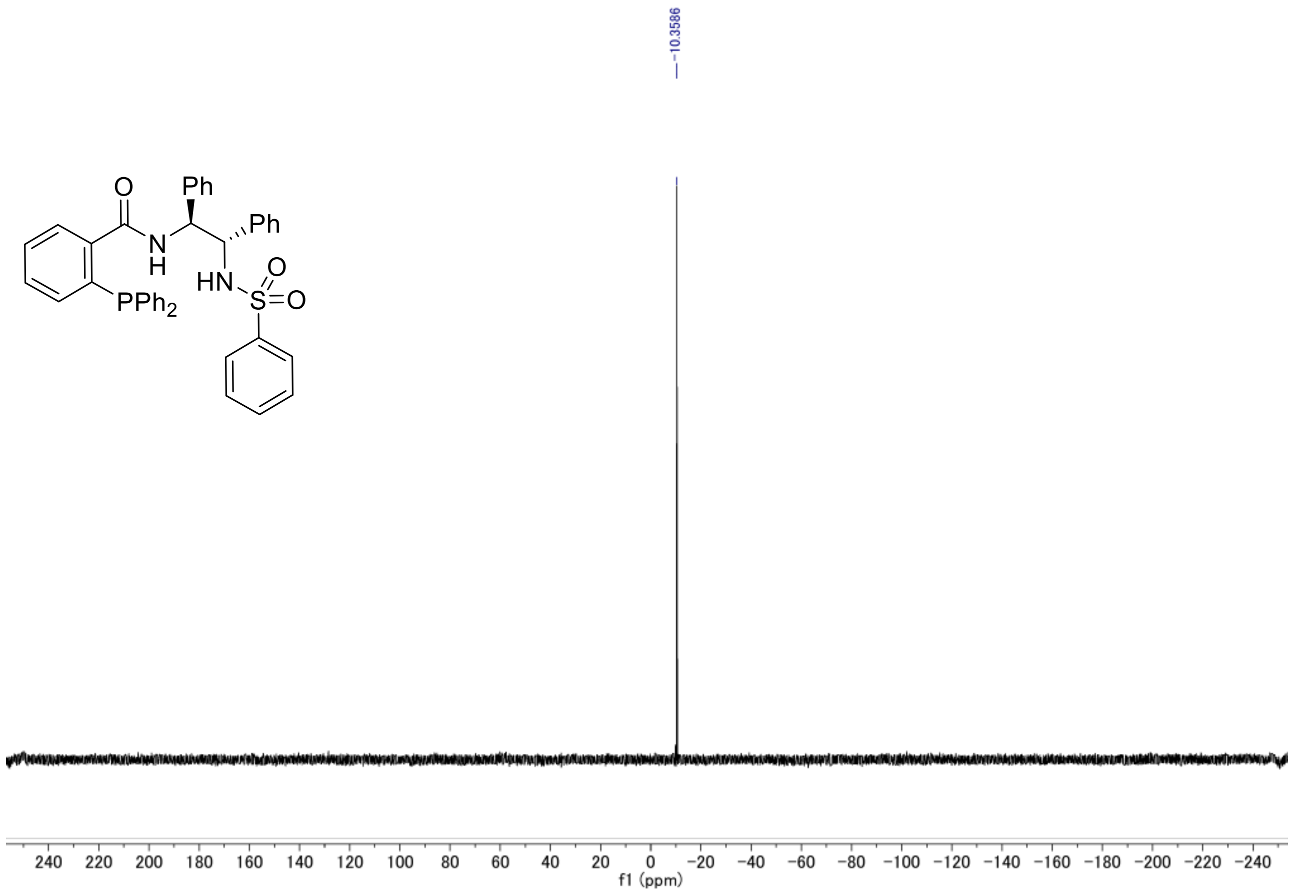

E ^{1}H NMR ($CDCl_3$, 400 MHz)

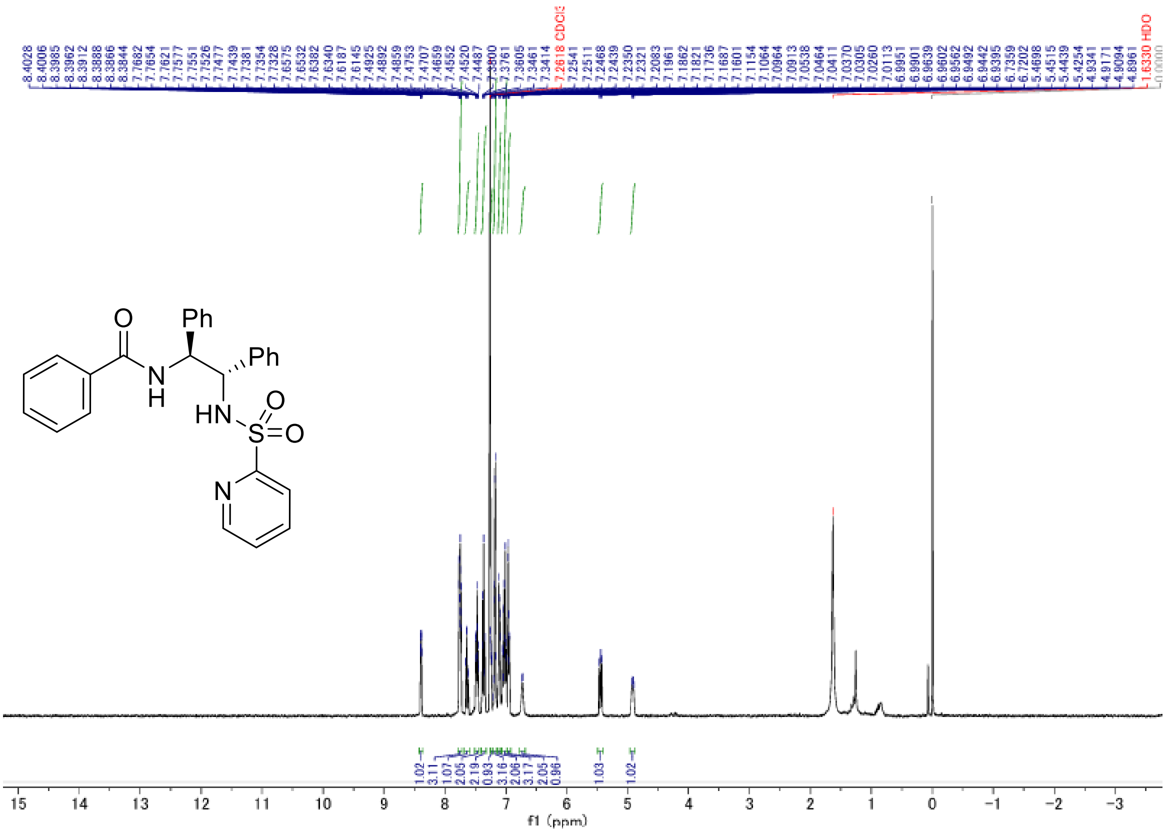

E ^{13}C NMR ($CDCl_3$, 125 MHz)

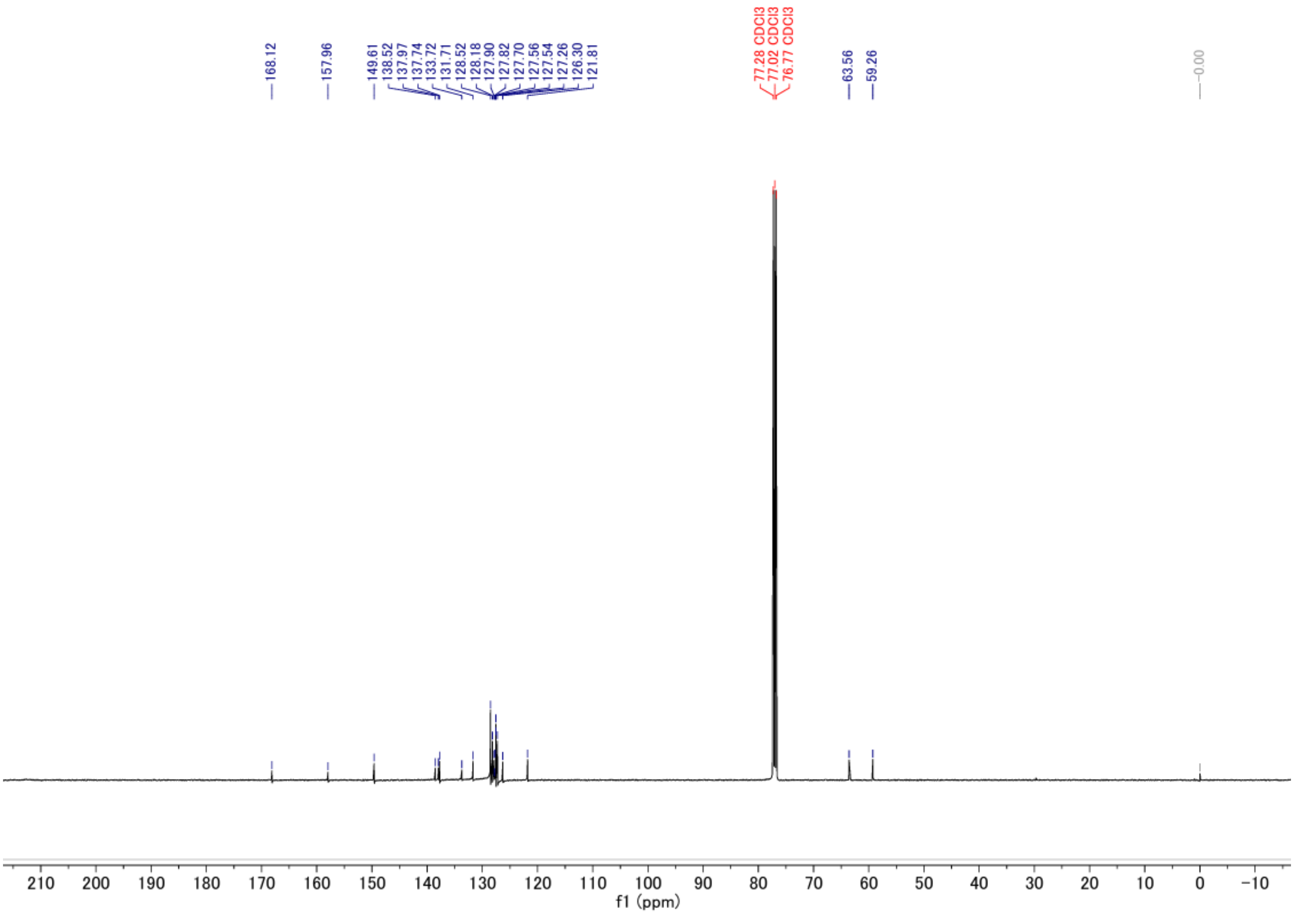

F ^{1}H NMR ($CDCl_3$, 300 MHz)

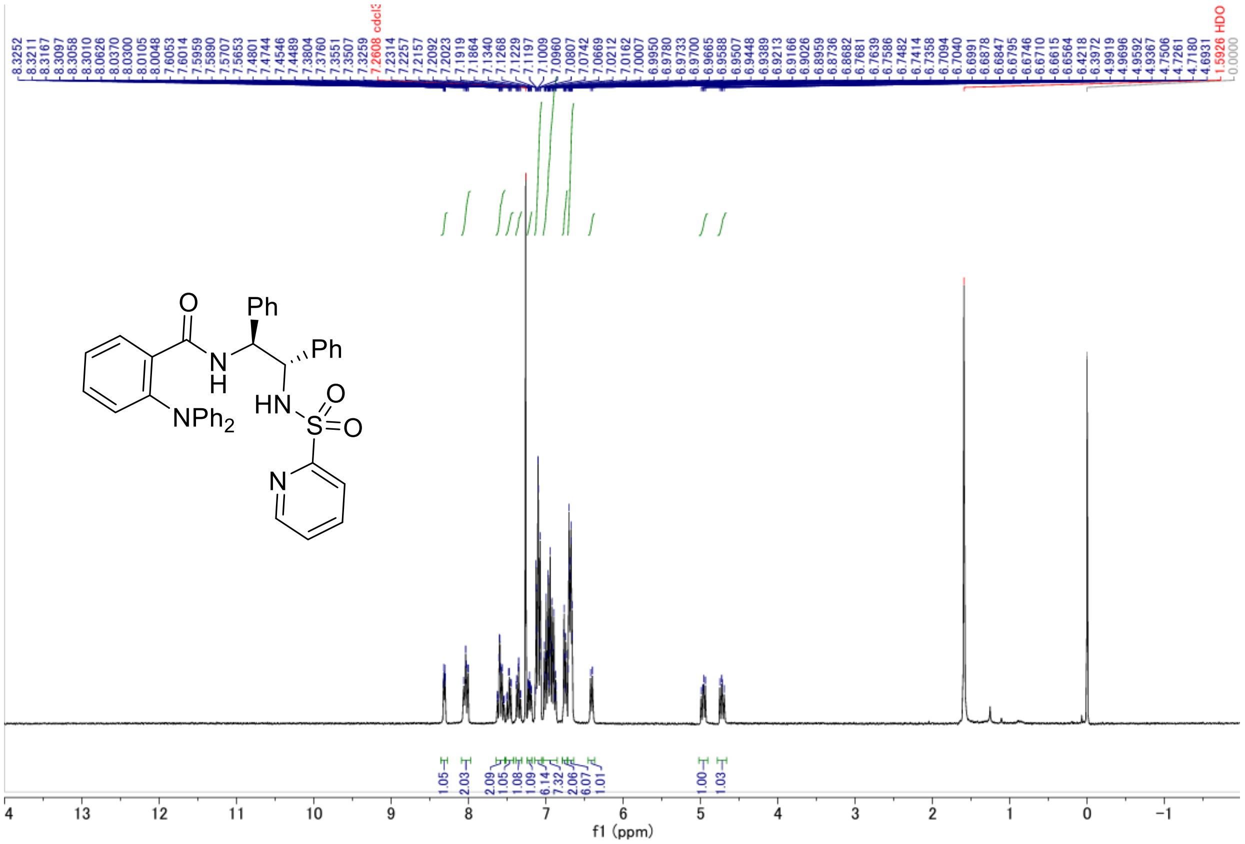

F ^{13}C NMR ($CDCl_3$, 125 MHz)

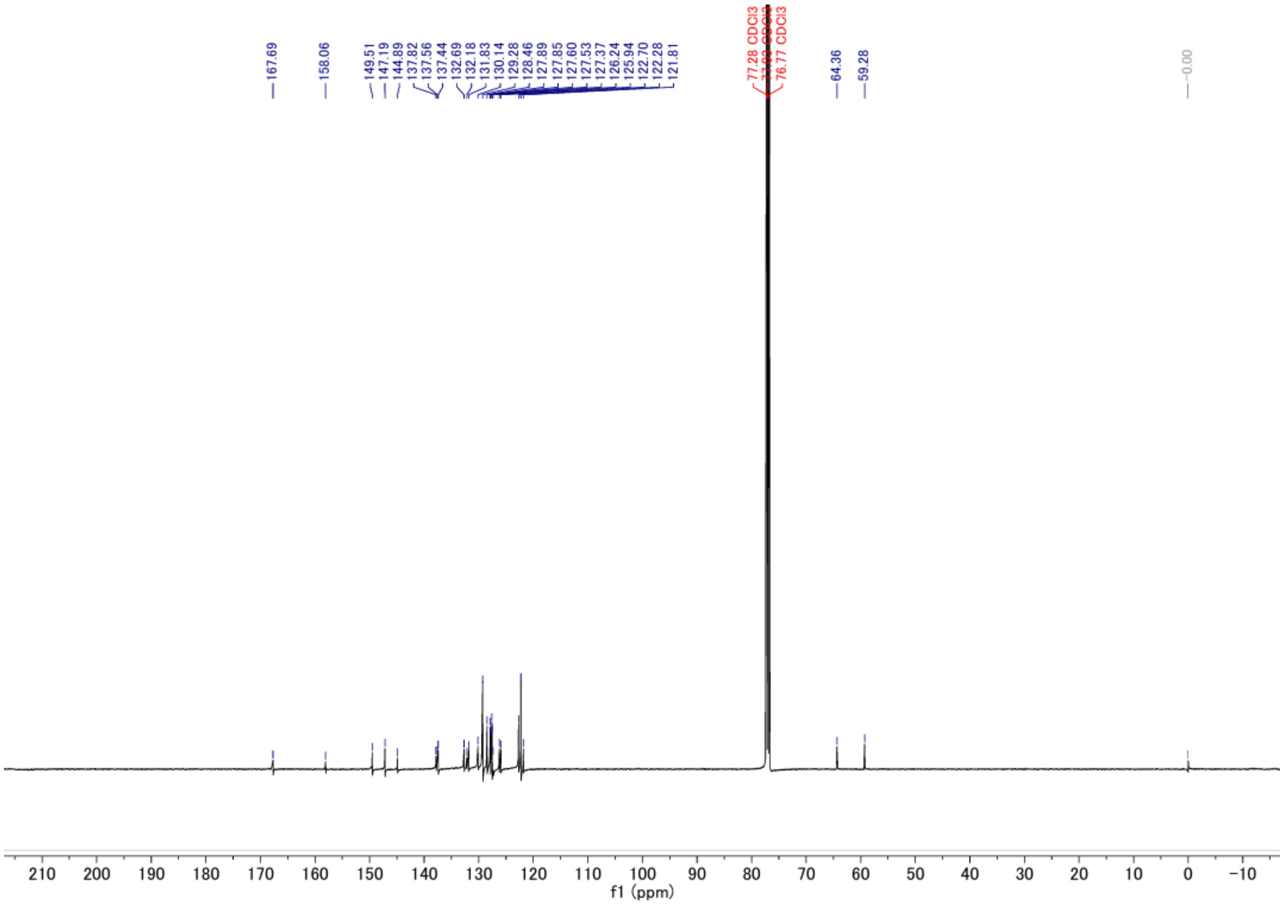

G ^{1}H NMR ($CDCl_3$, 400 MHz)

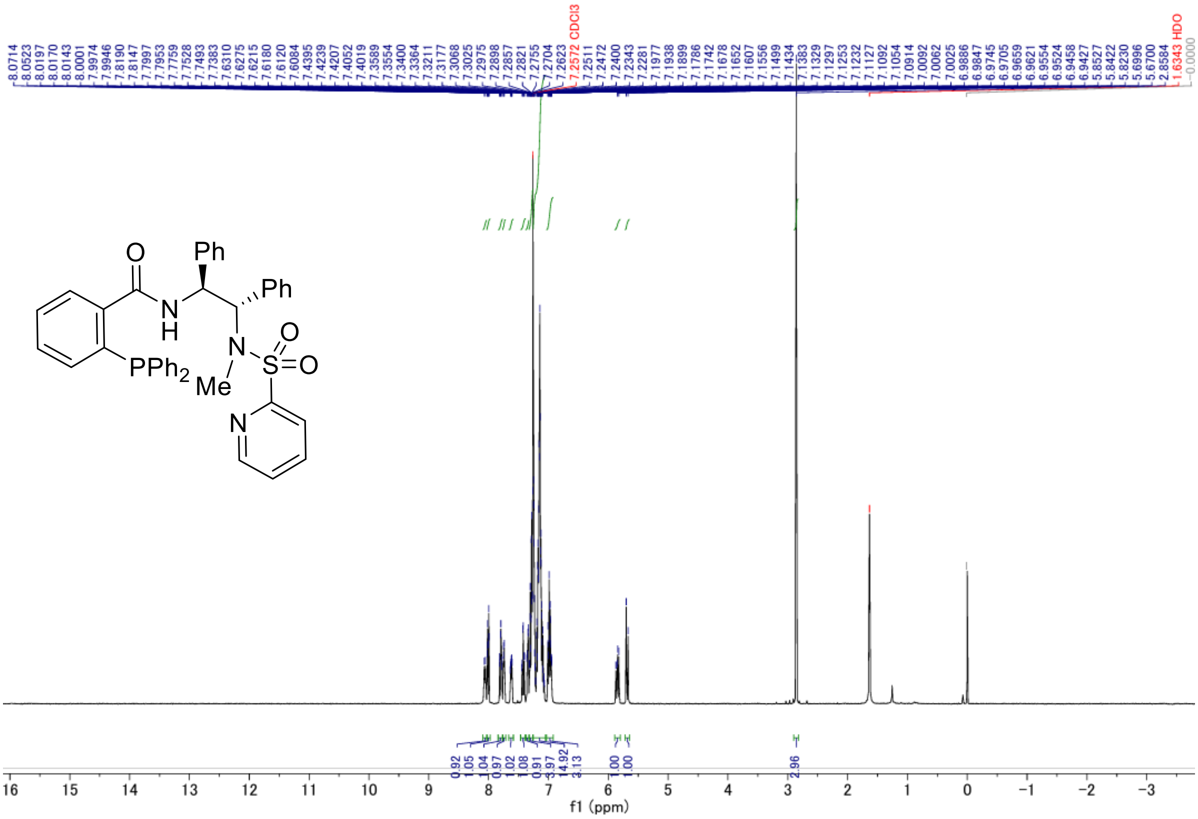

G ^{13}C NMR ($CDCl_3$, 176 MHz)

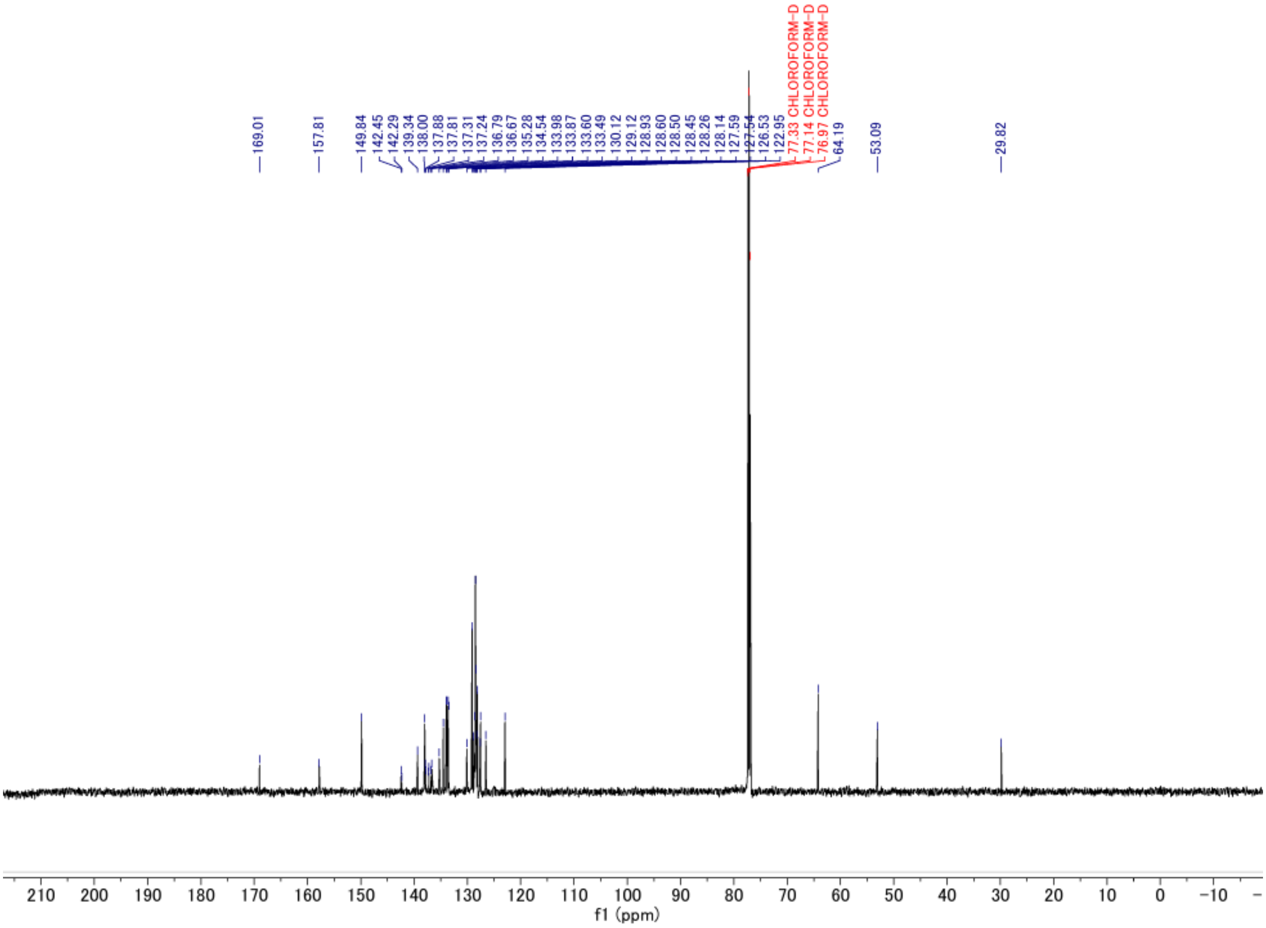

G ^{31}P NMR (CDCl$_3$, 283 MHz)

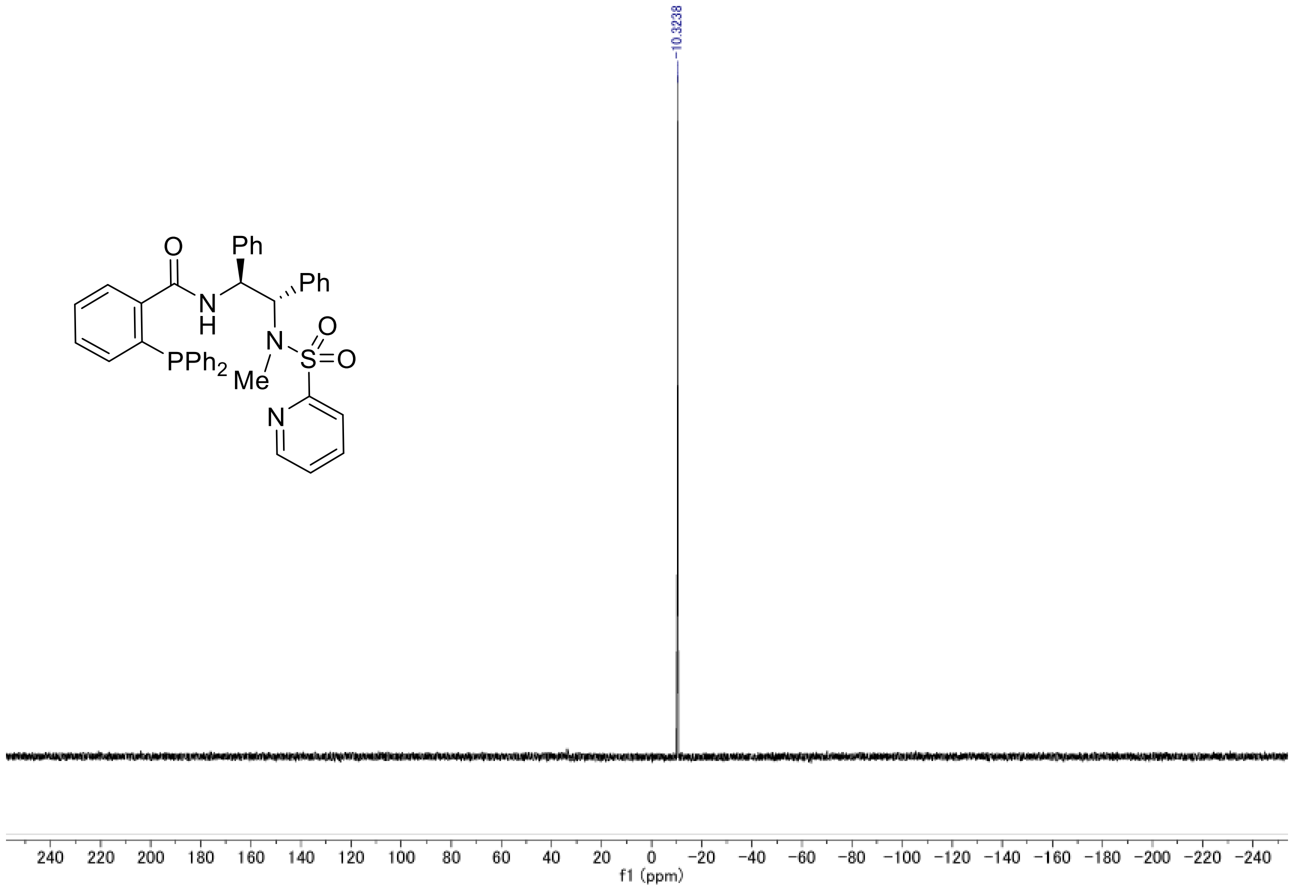

H ^{1}H NMR ($CDCl_3$, 400 MHz)

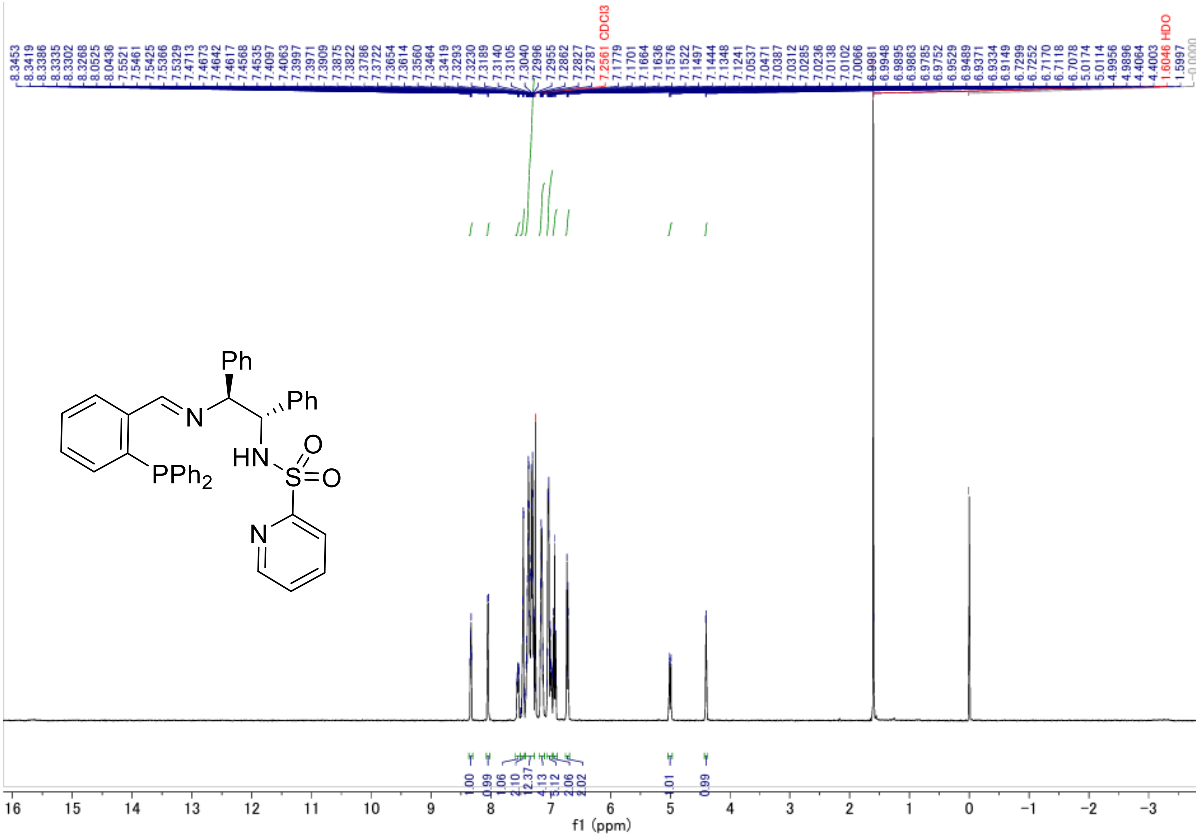

H ^{13}C NMR ($CDCl_3$, 176 MHz)

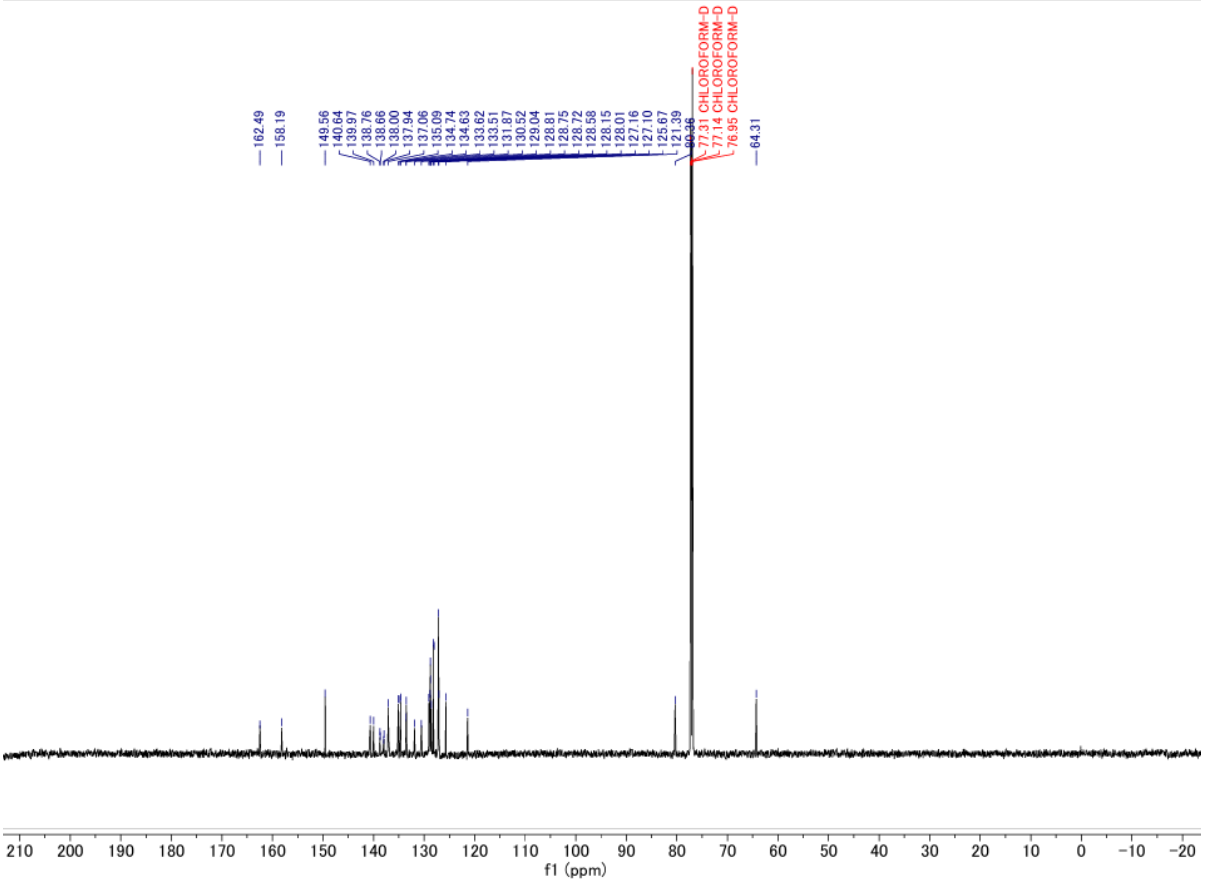

H ^{31}P NMR ($CDCl_3$, 283 MHz)

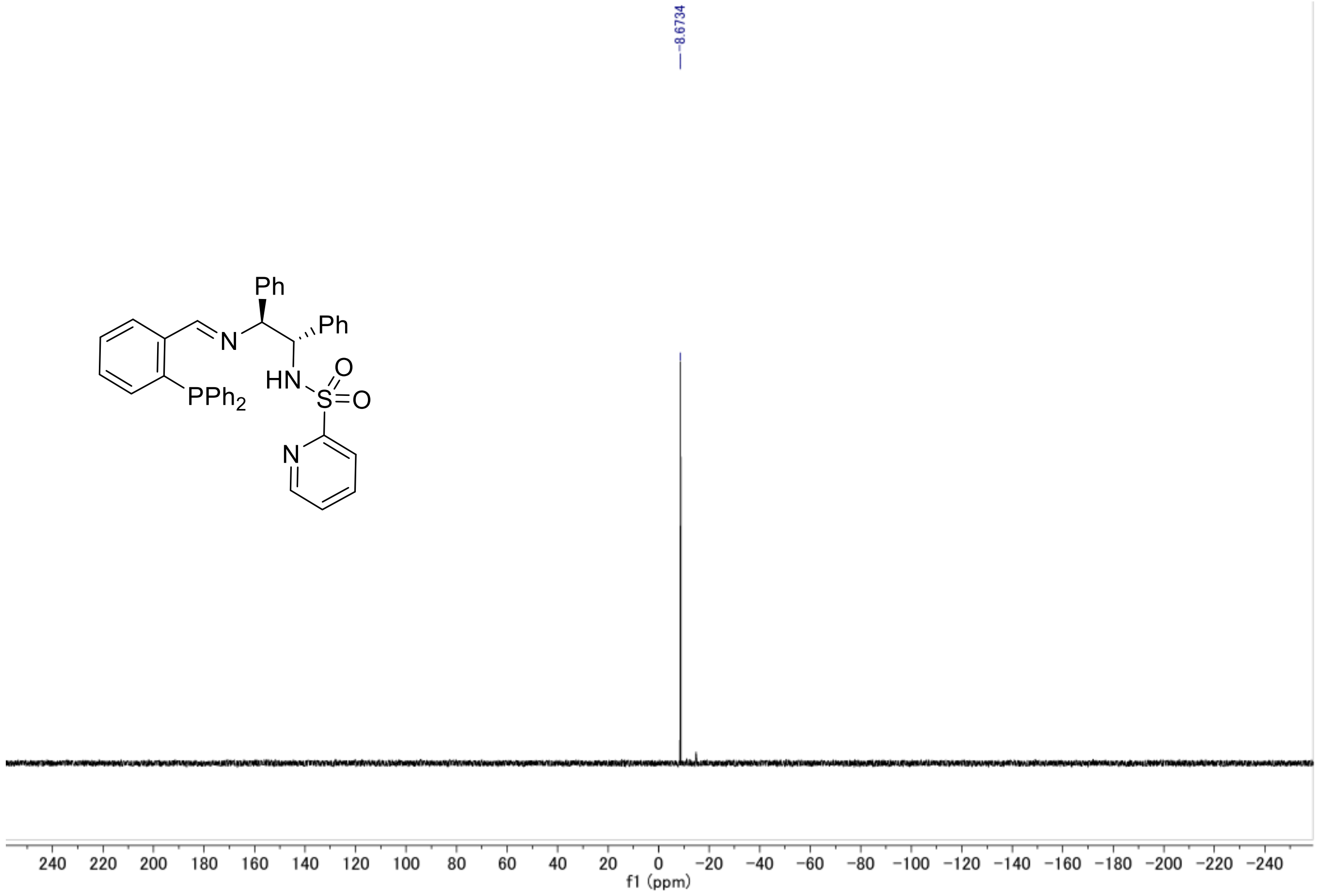

I ^{1}H NMR ($CDCl_3$, 400 MHz)

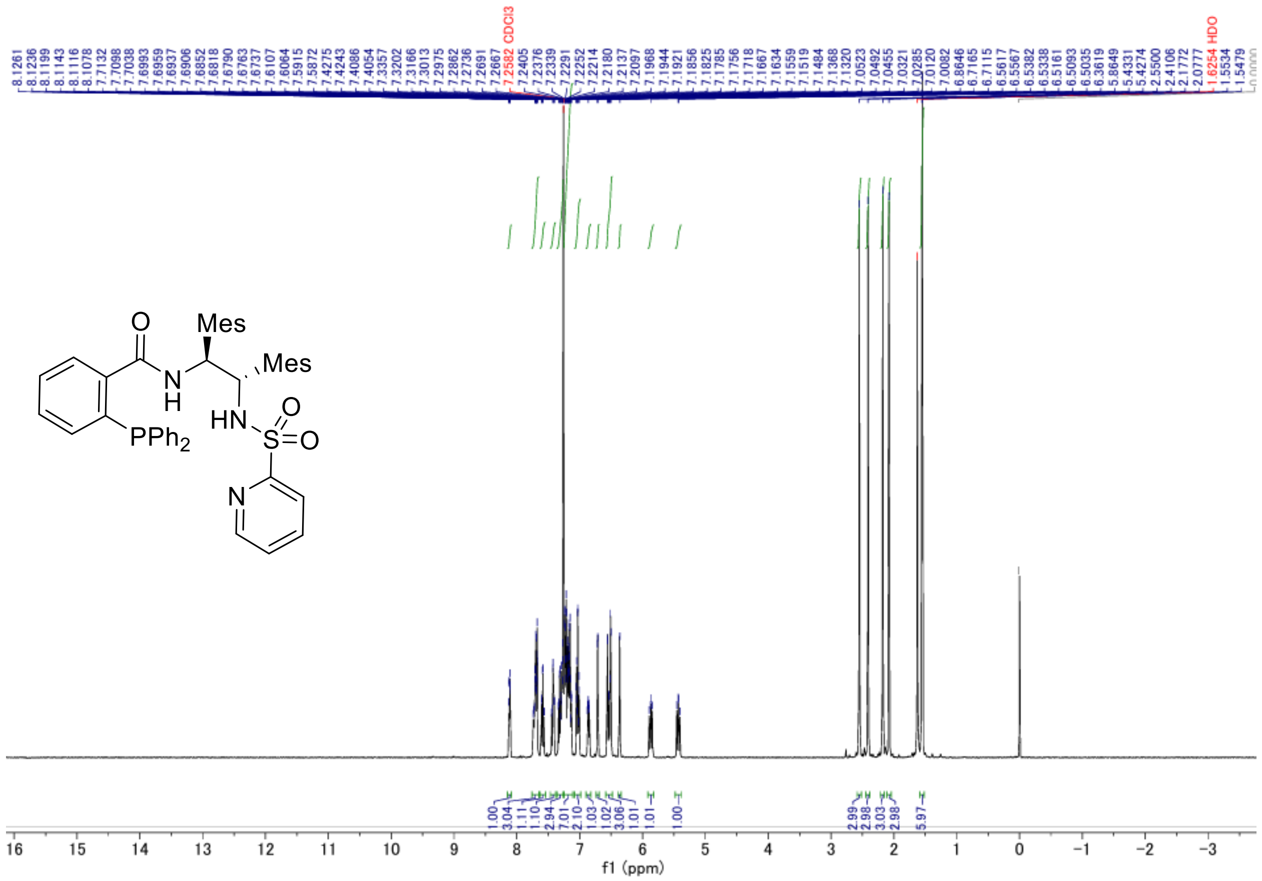

I ^{13}C NMR ($CDCl_3$, 176 MHz)

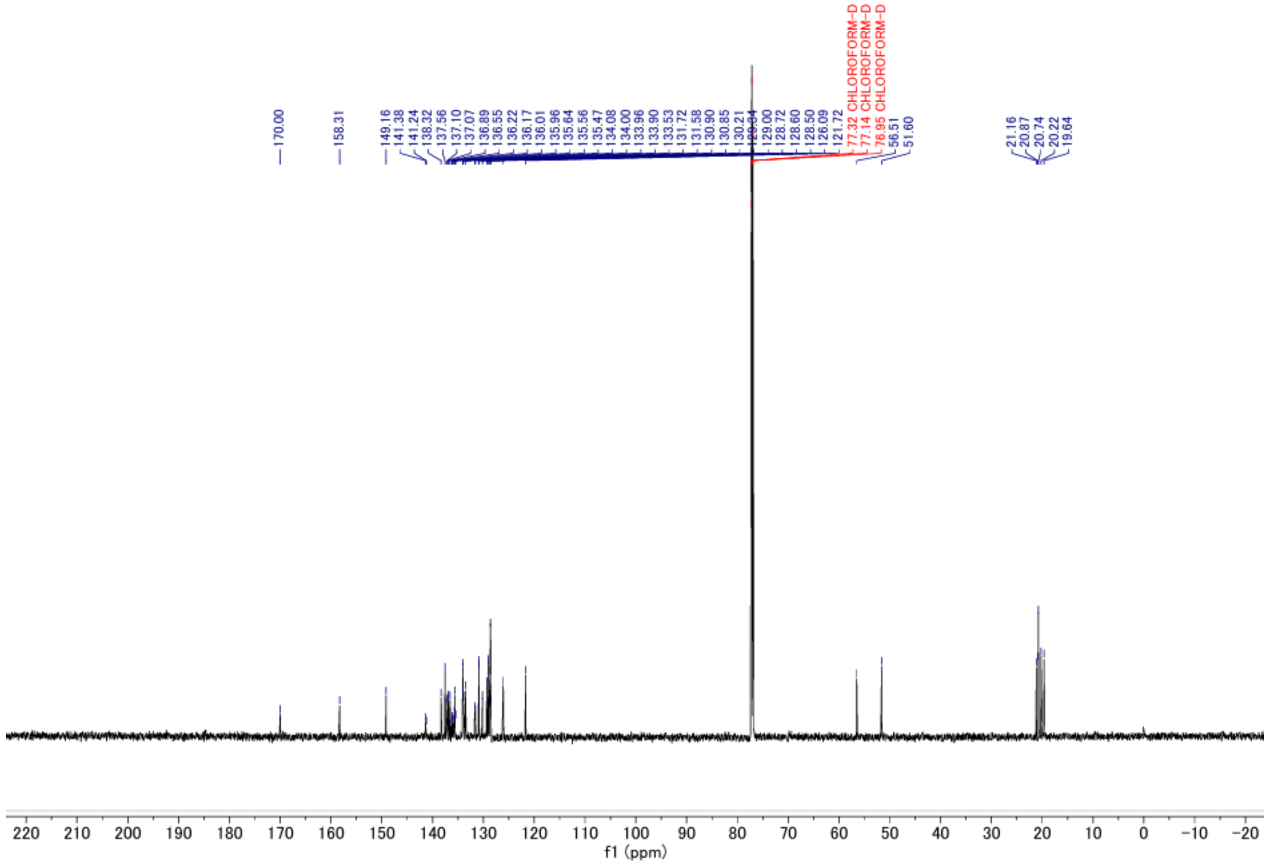

I ^{31}P NMR (CDCl$_3$, 283 MHz)

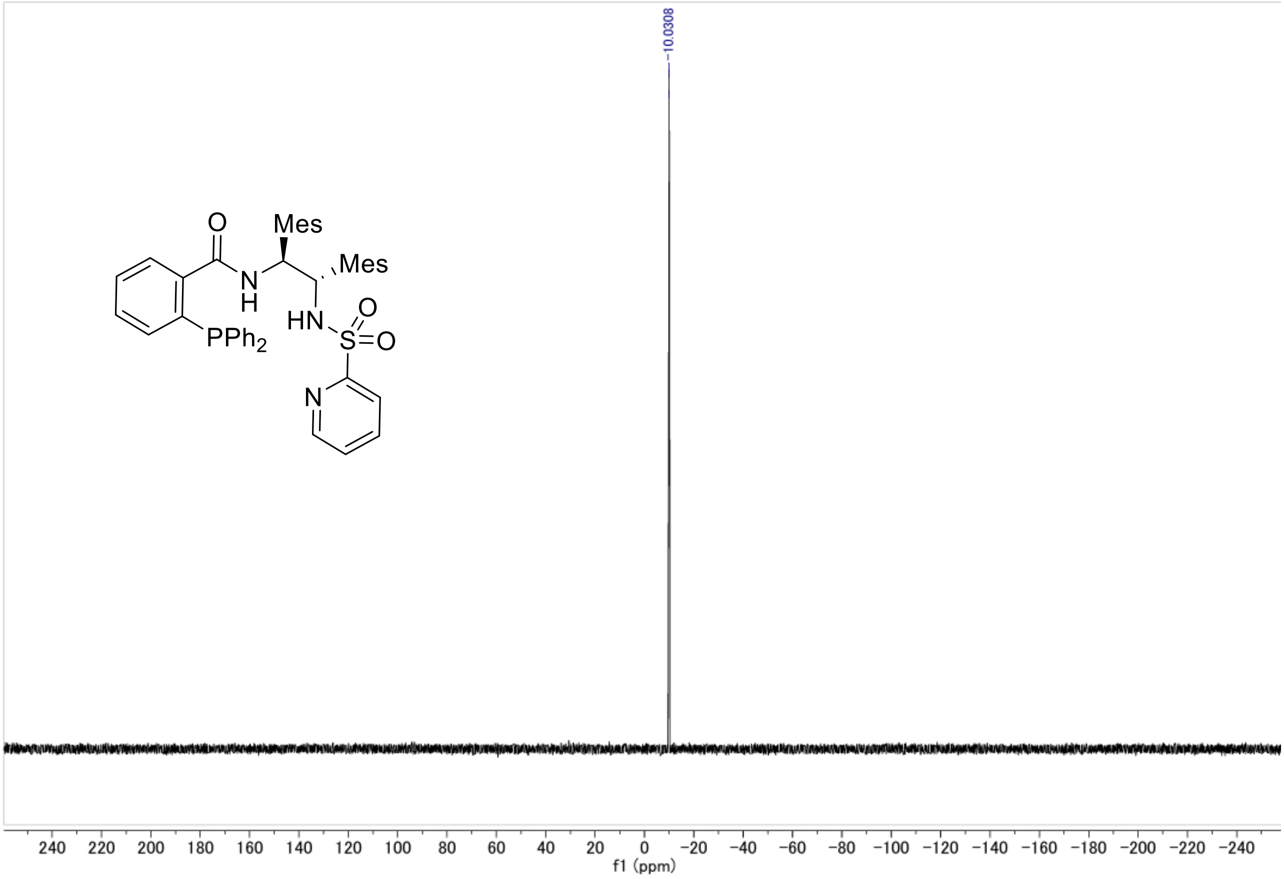

1e ^{1}H NMR ($CDCl_3$, 400 MHz)

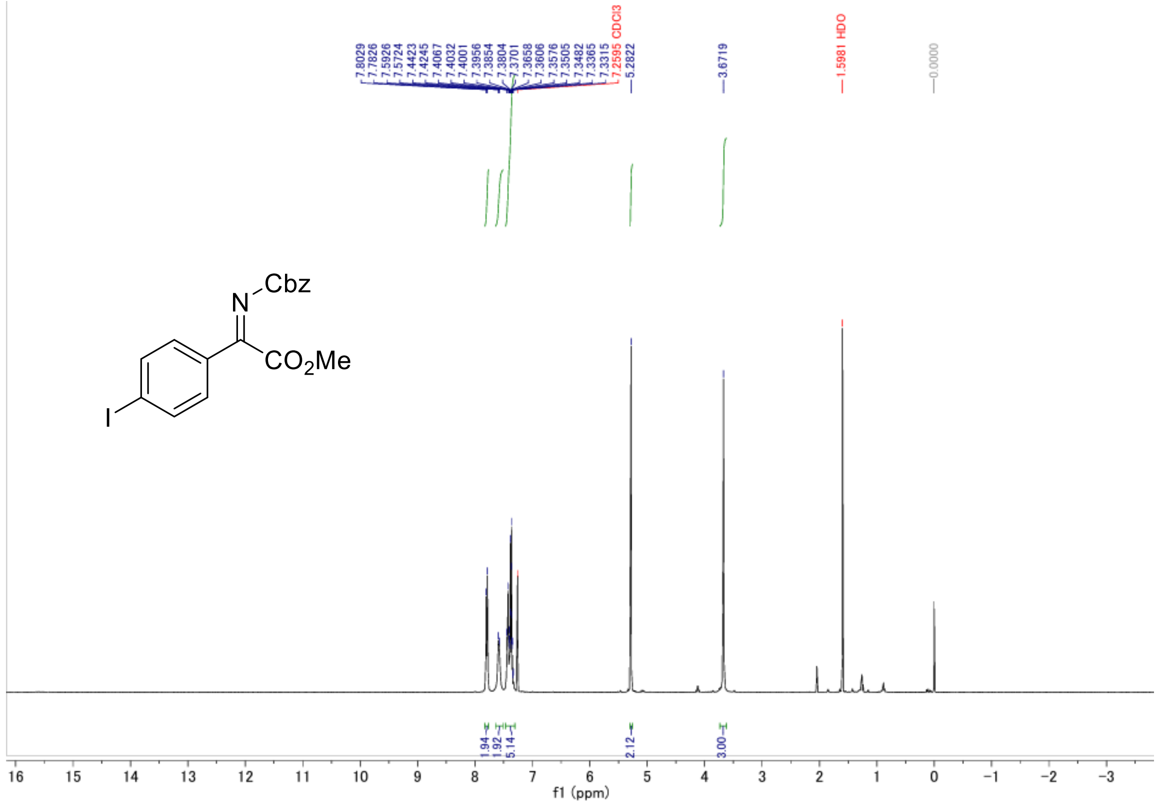

1e ^{13}C NMR ($CDCl_3$, 176 MHz)

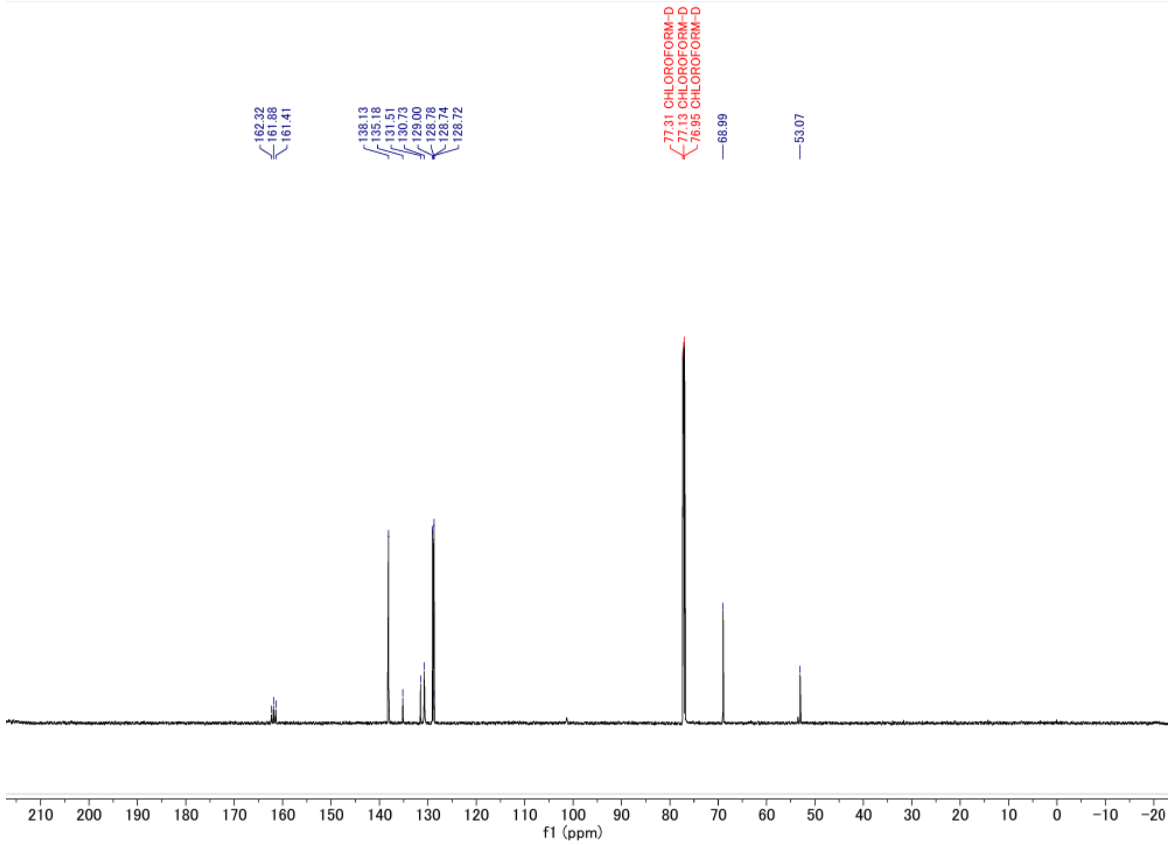

1g ^{1}H NMR ($CDCl_3$, 300 MHz)

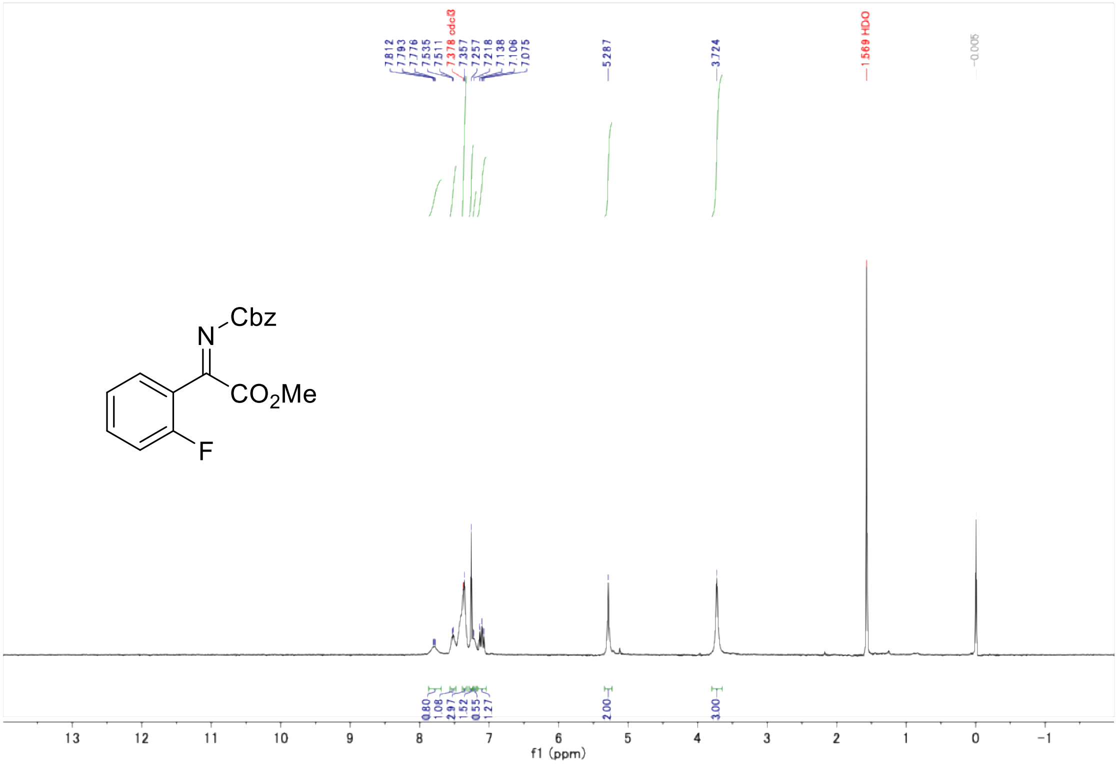

1g ^{13}C NMR ($CDCl_3$, 125 MHz)

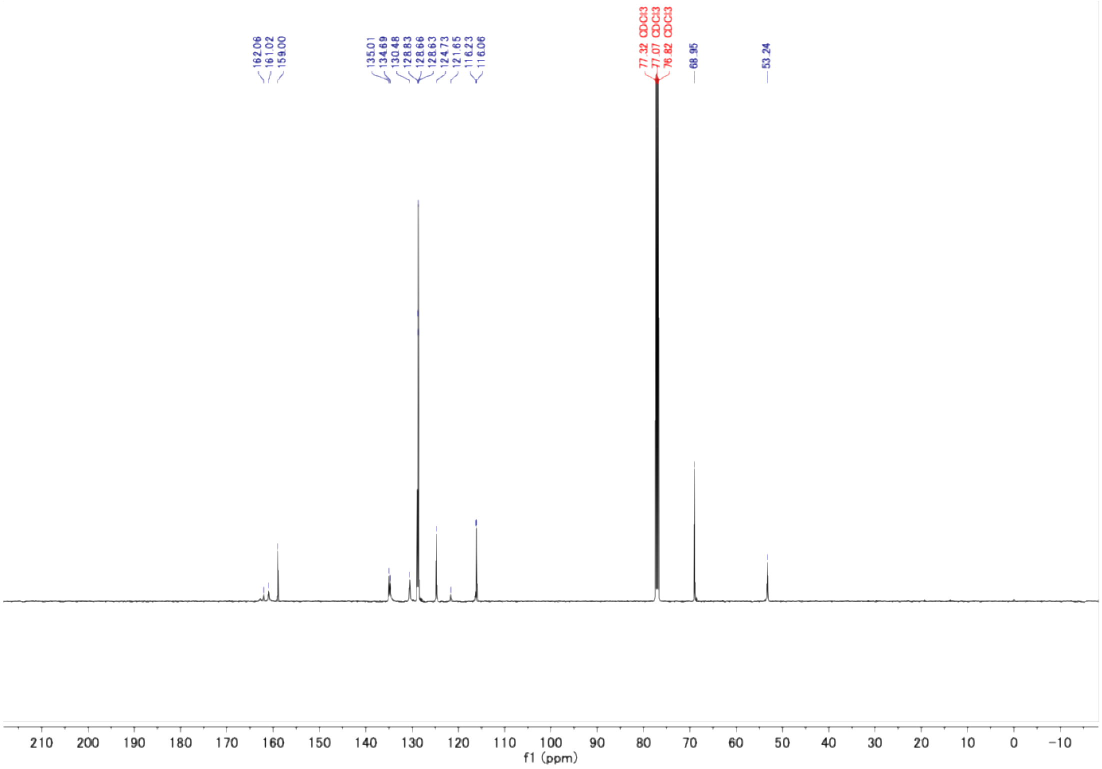

1g ^{19}F NMR ($CDCl_3$, 282 MHz)

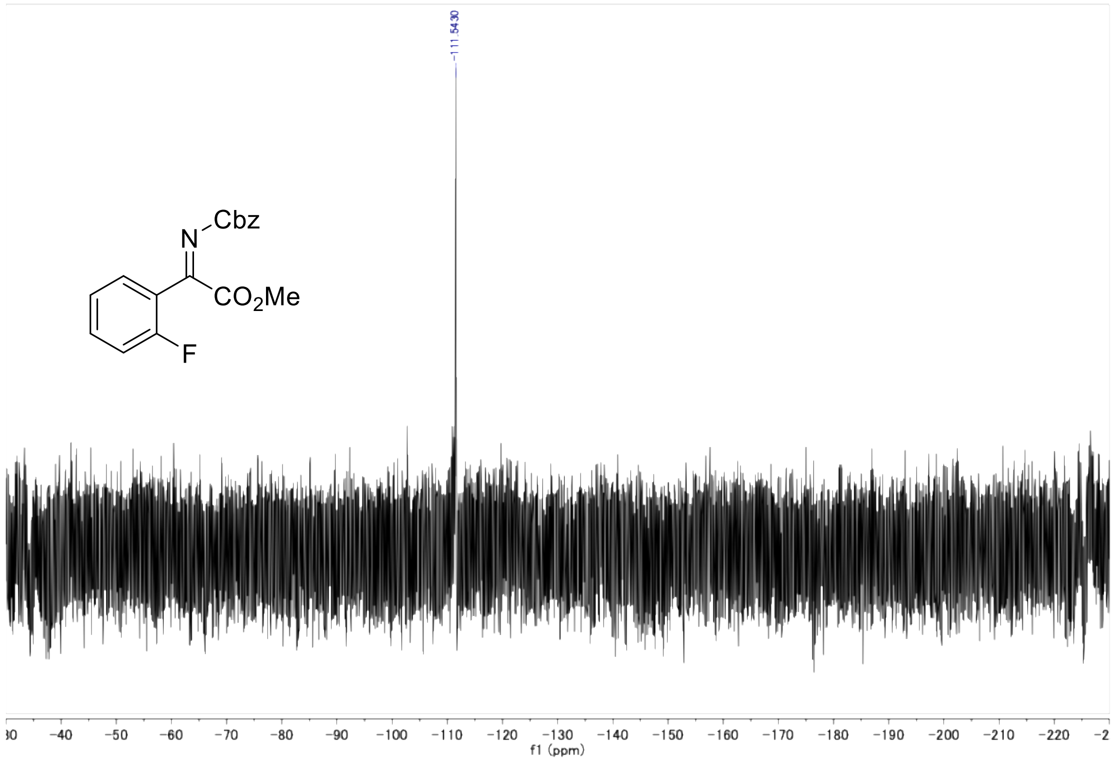

1j ^{1}H NMR ($CDCl_3$, 300 MHz)

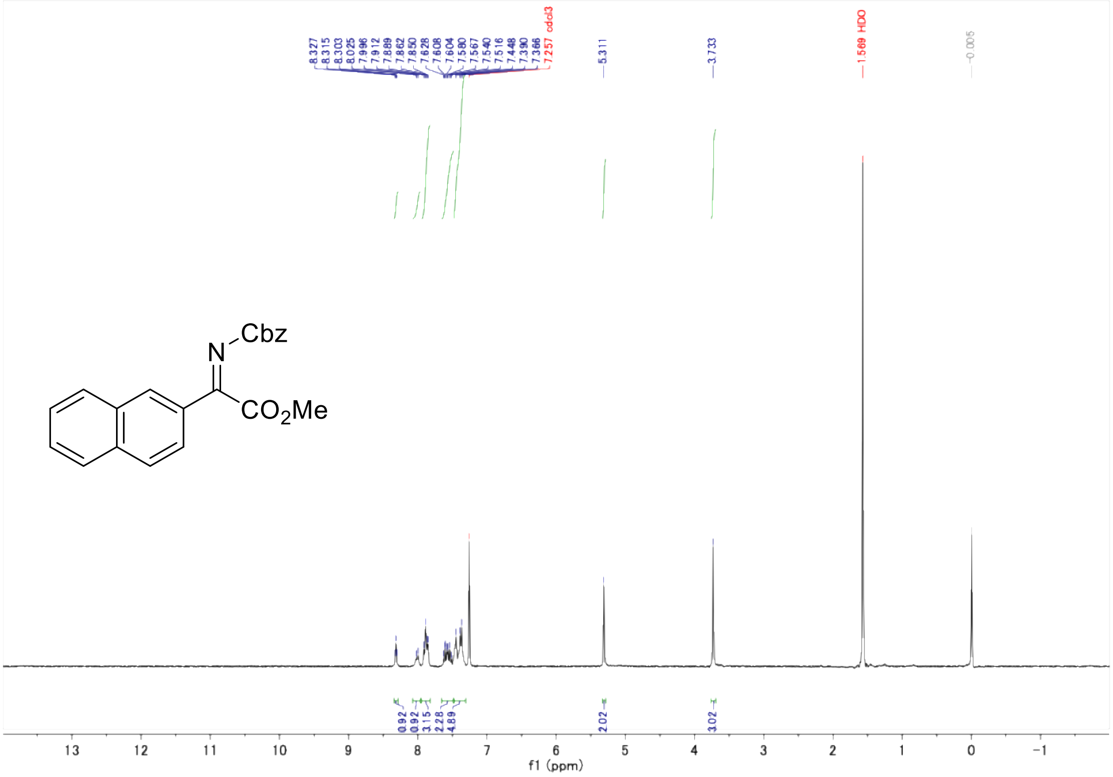

1j ^{13}C NMR ($CDCl_3$, 125 MHz)

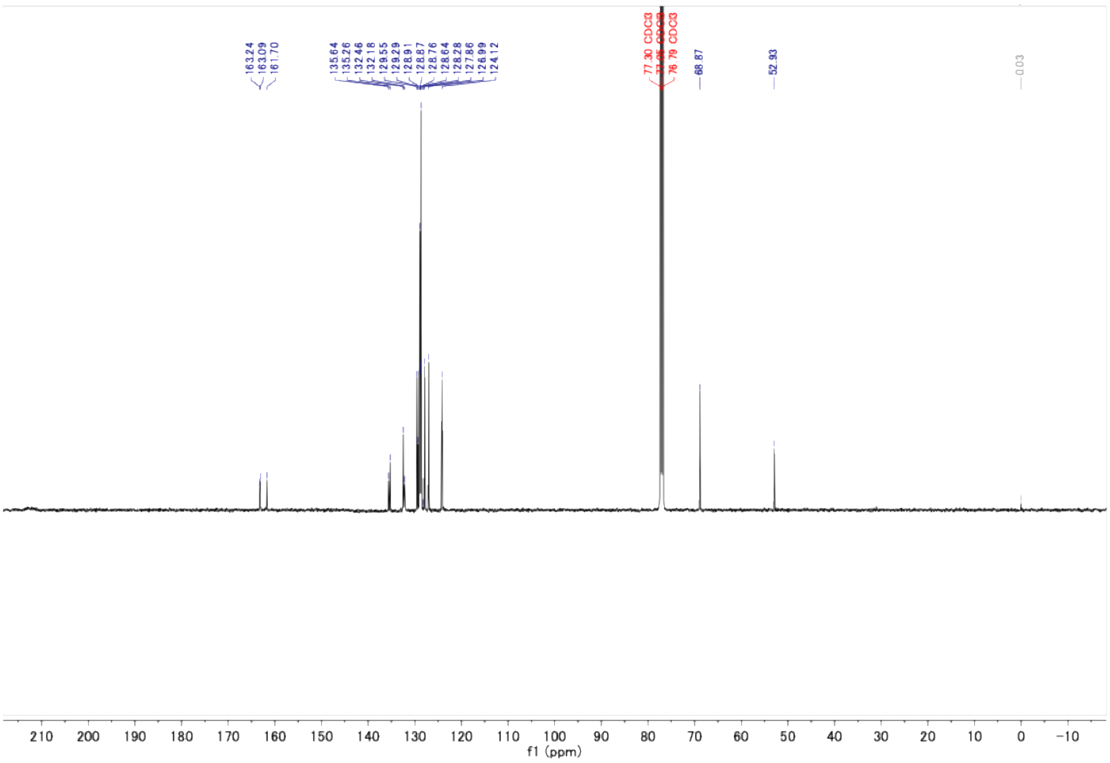

1k ^{1}H NMR ($CDCl_3$, 300 MHz)

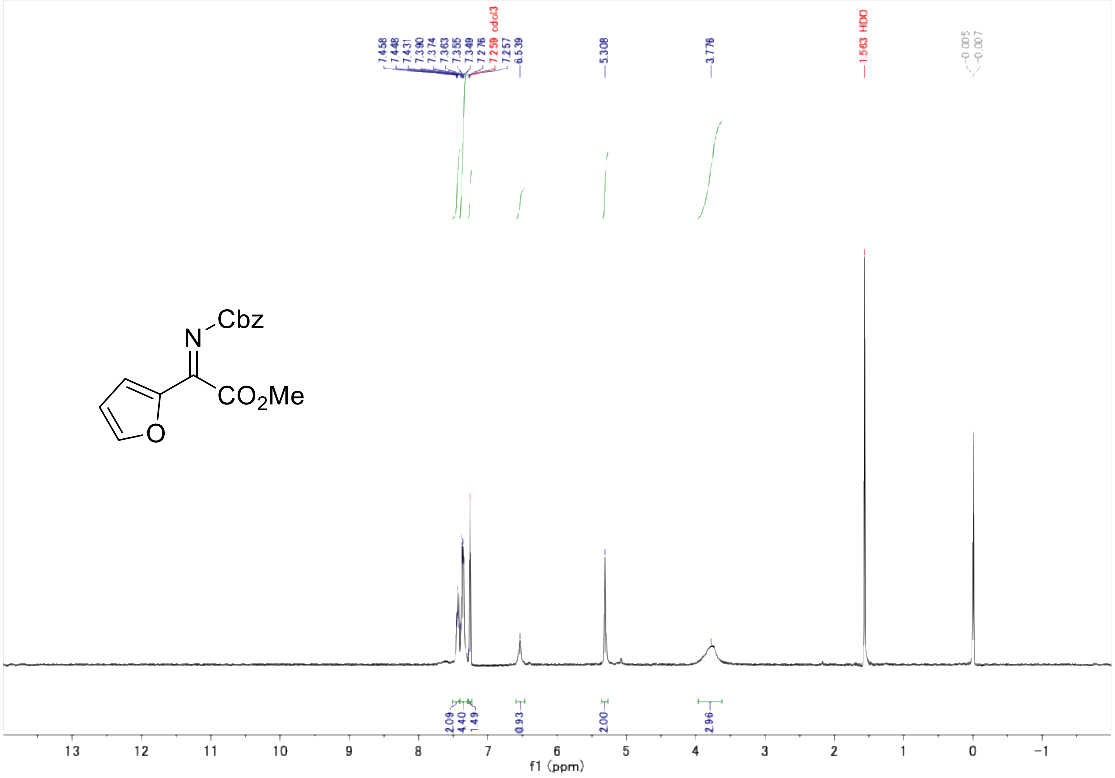

1k ^{13}C NMR ($CDCl_3$, 125 MHz)

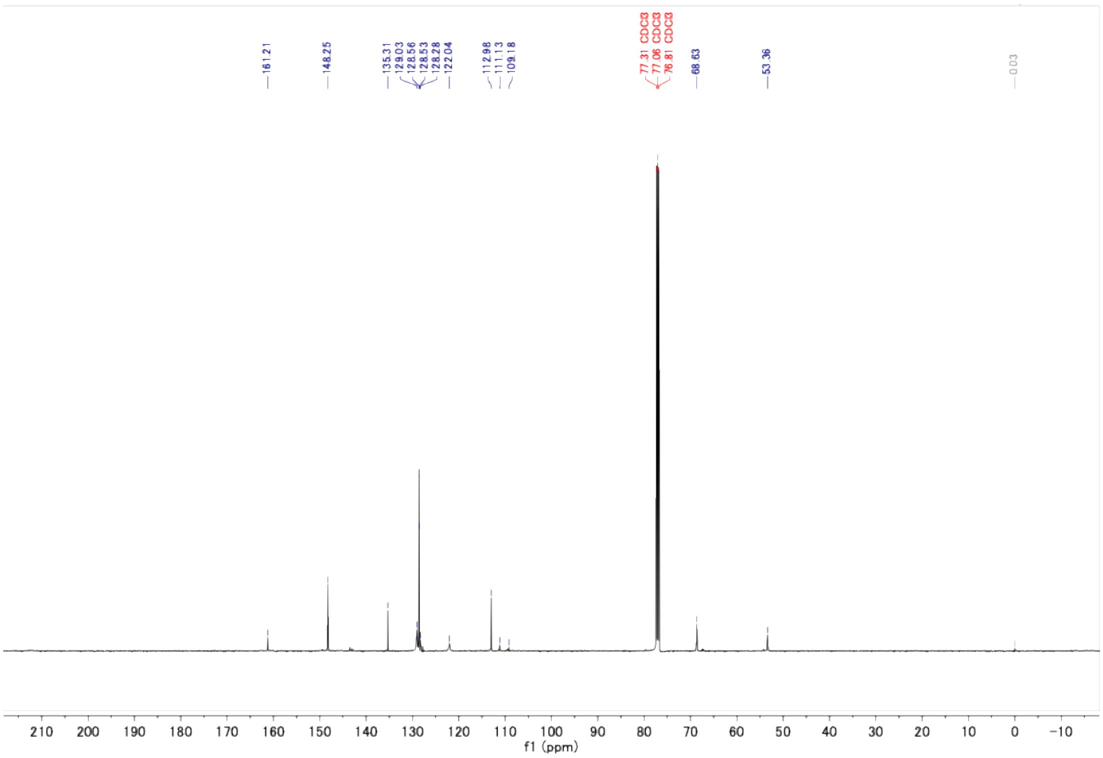

1l ^{1}H NMR ($CDCl_3$, 300 MHz)

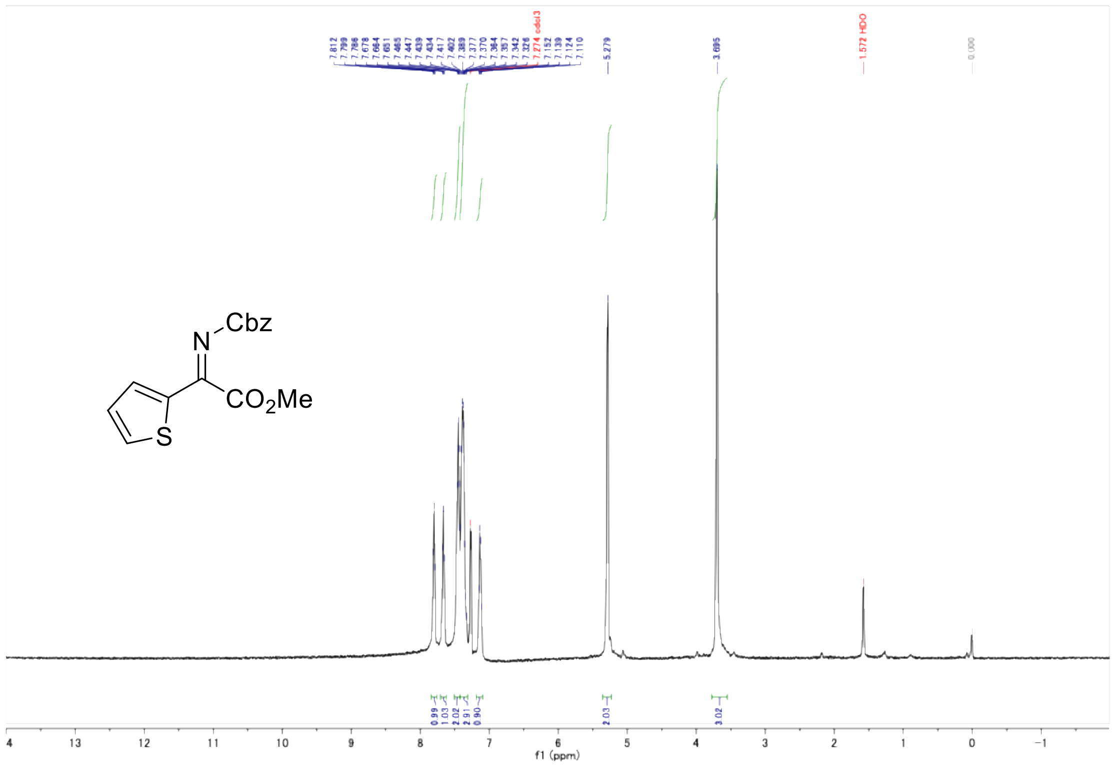

1l ^{13}C NMR ($CDCl_3$, 125 MHz)

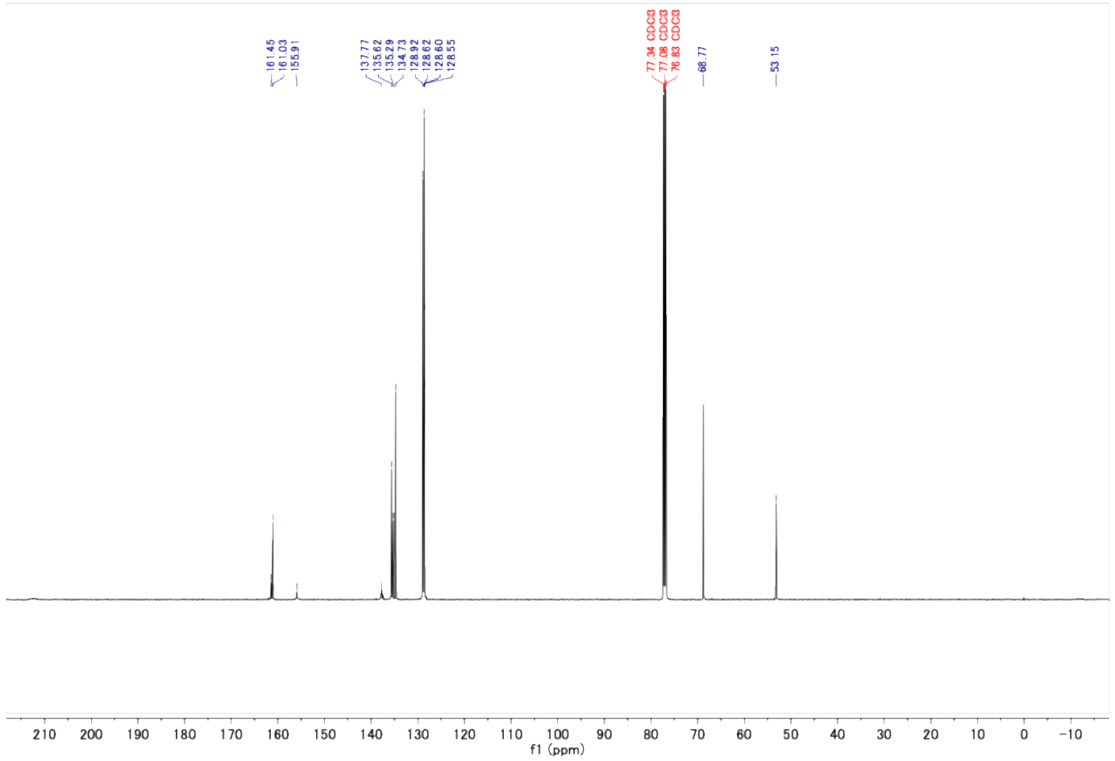

3a ^{1}H NMR ($CDCl_3$, 300 MHz)

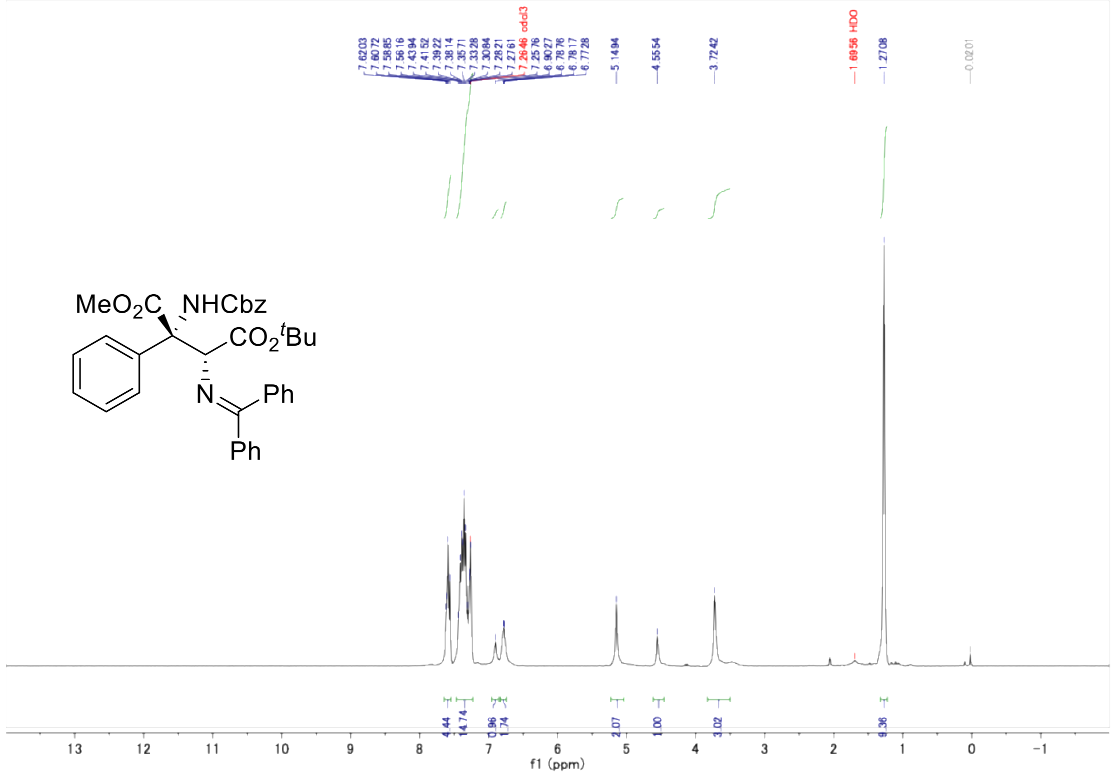

3a ^{13}C NMR ($CDCl_3$, 125 MHz)

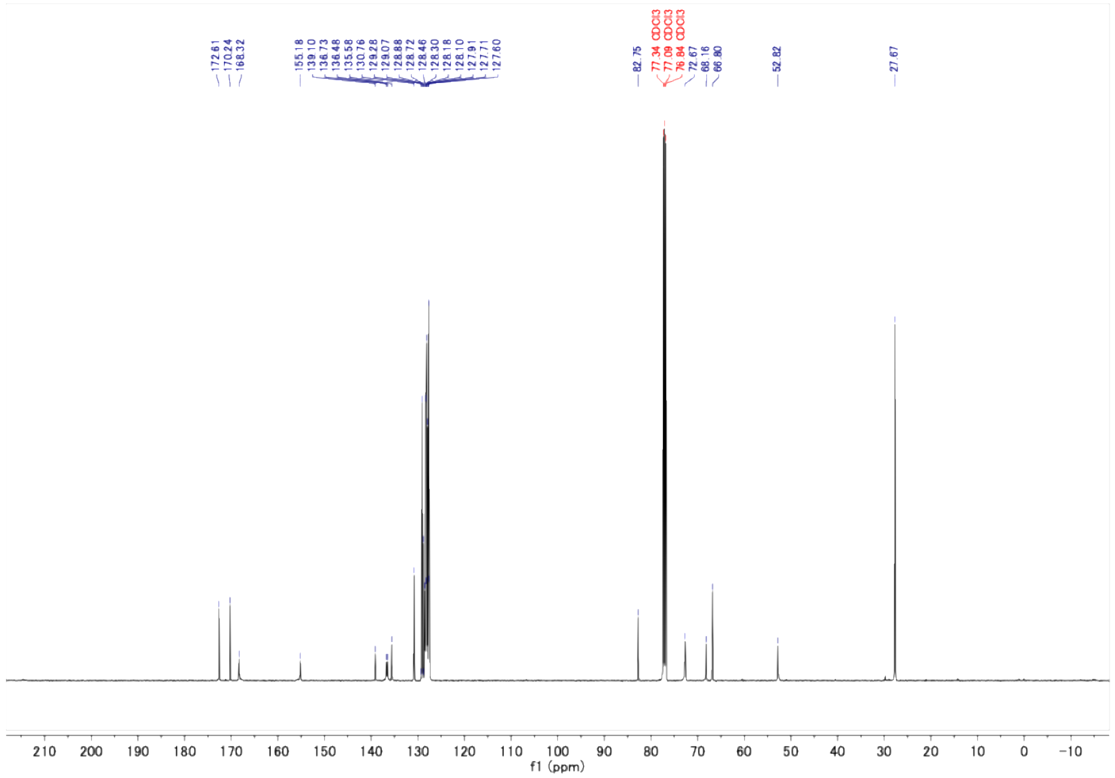

3b ^{1}H NMR ($CDCl_3$, 400 MHz)

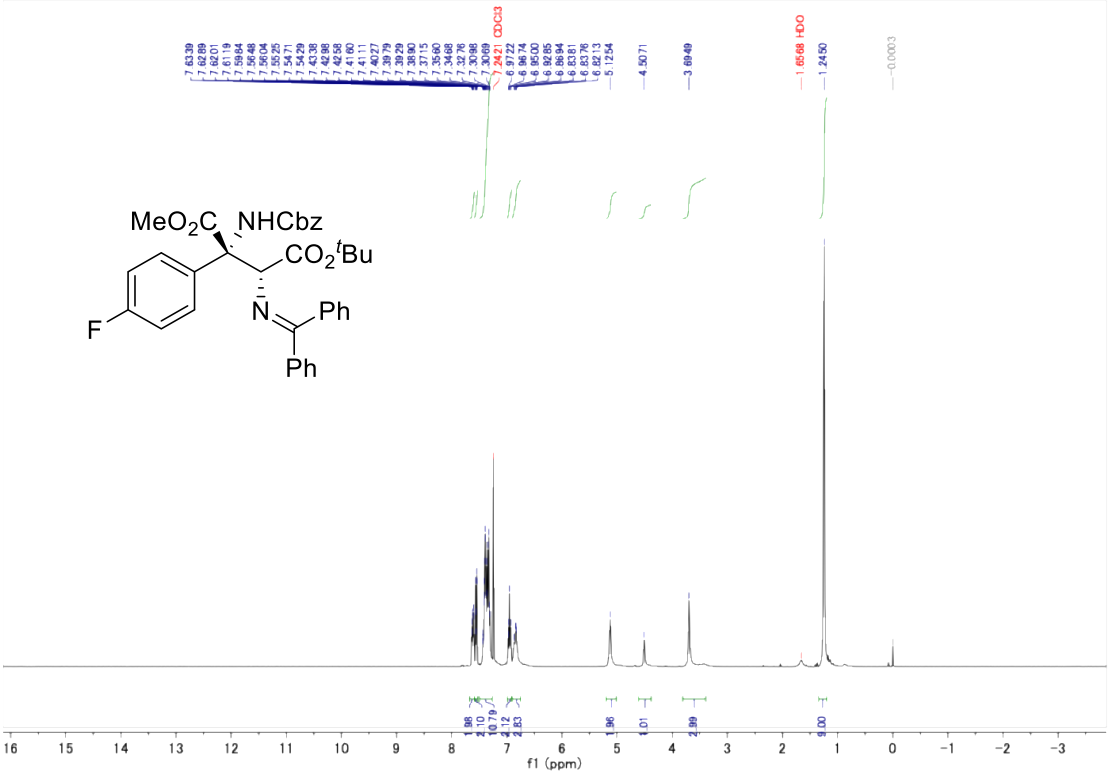

3b ^{13}C NMR ($CDCl_3$, 176 MHz)

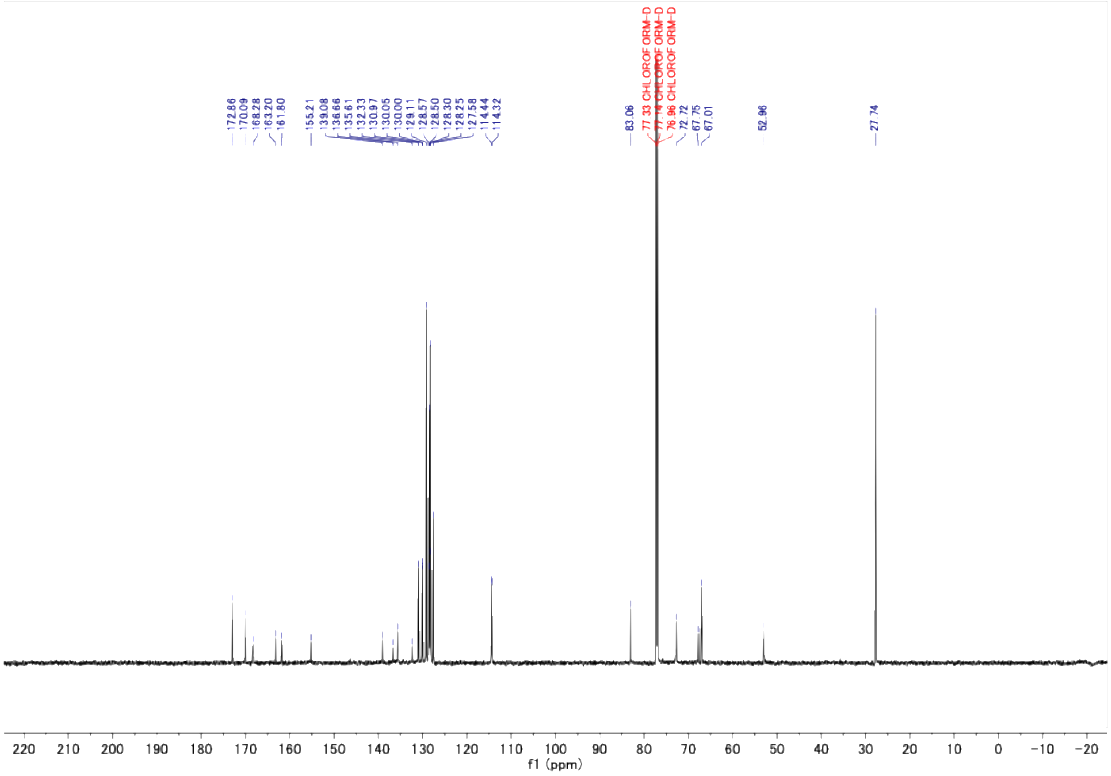

3b ^{19}F NMR ($CDCl_3$, 282 MHz)

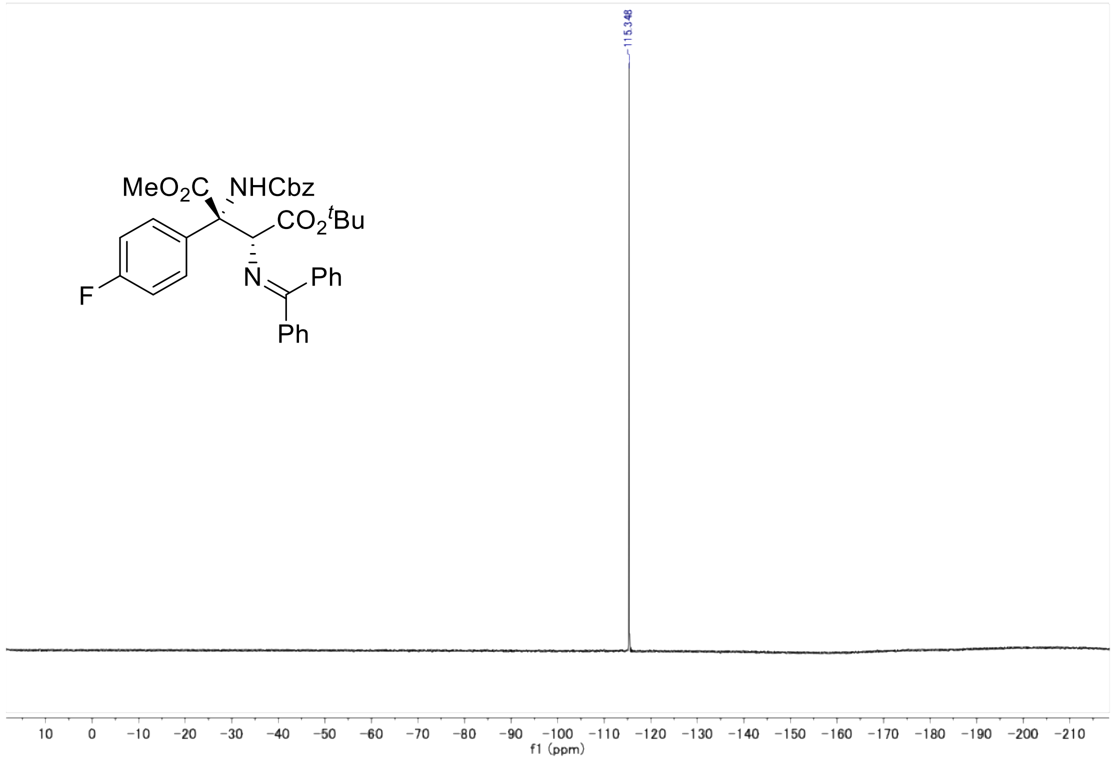

3c ^{1}H NMR ($CDCl_3$, 300 MHz)

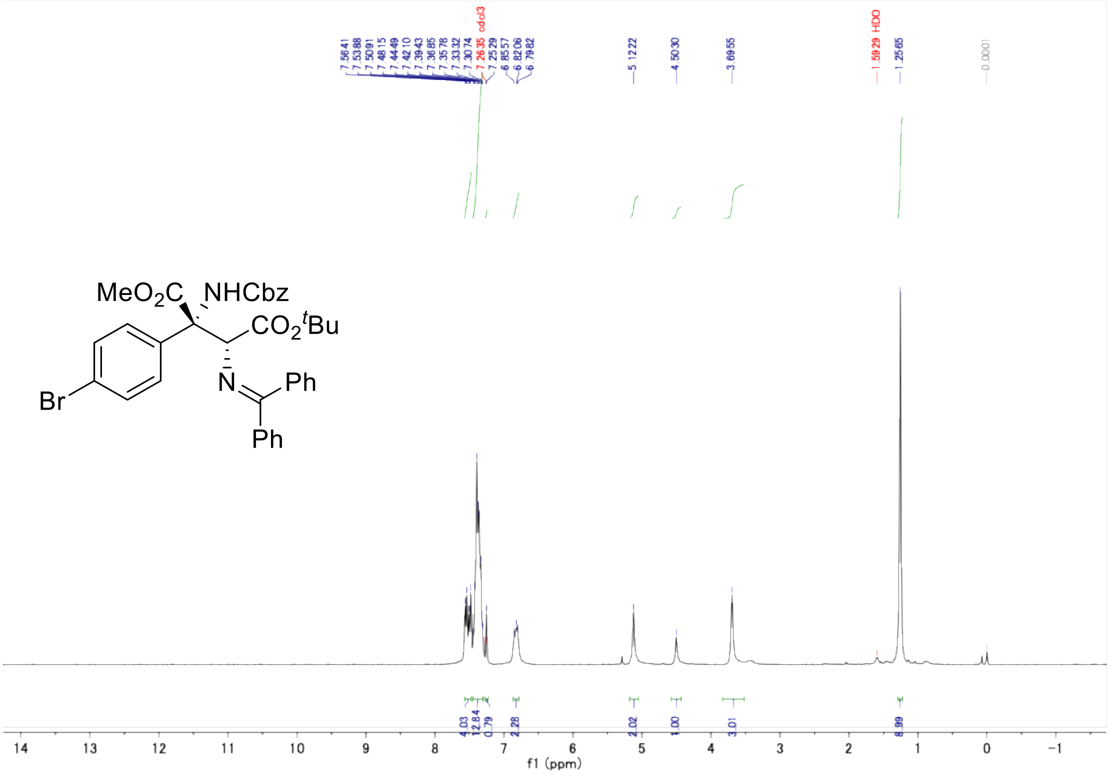

3c ^{13}C NMR ($CDCl_3$, 125 MHz)

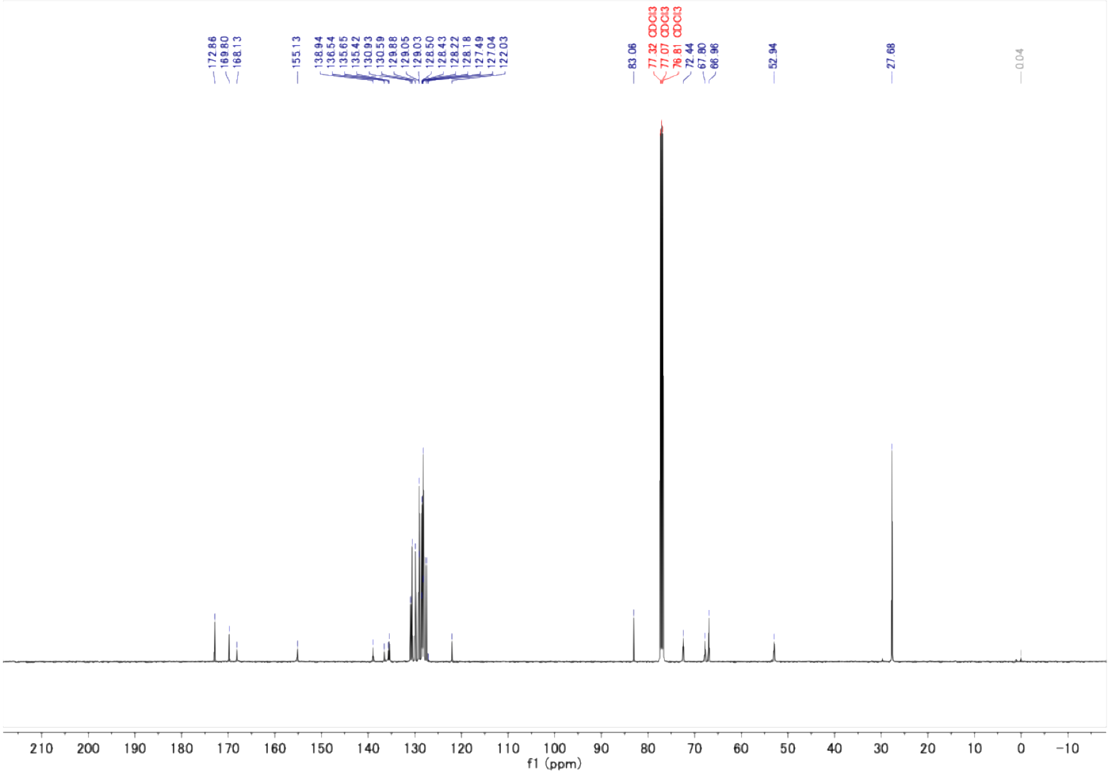

3d ^{1}H NMR (CDCl$_3$, 400 MHz)

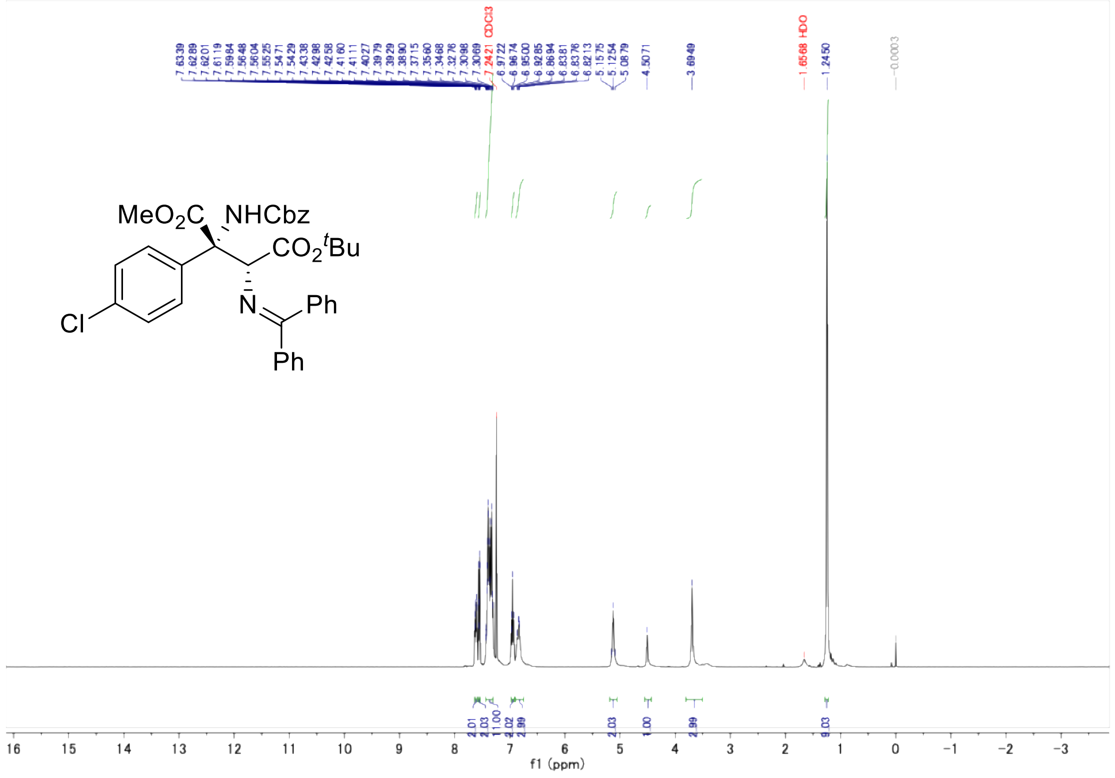

3d ^{13}C NMR (CDCl$_3$, 125 MHz)

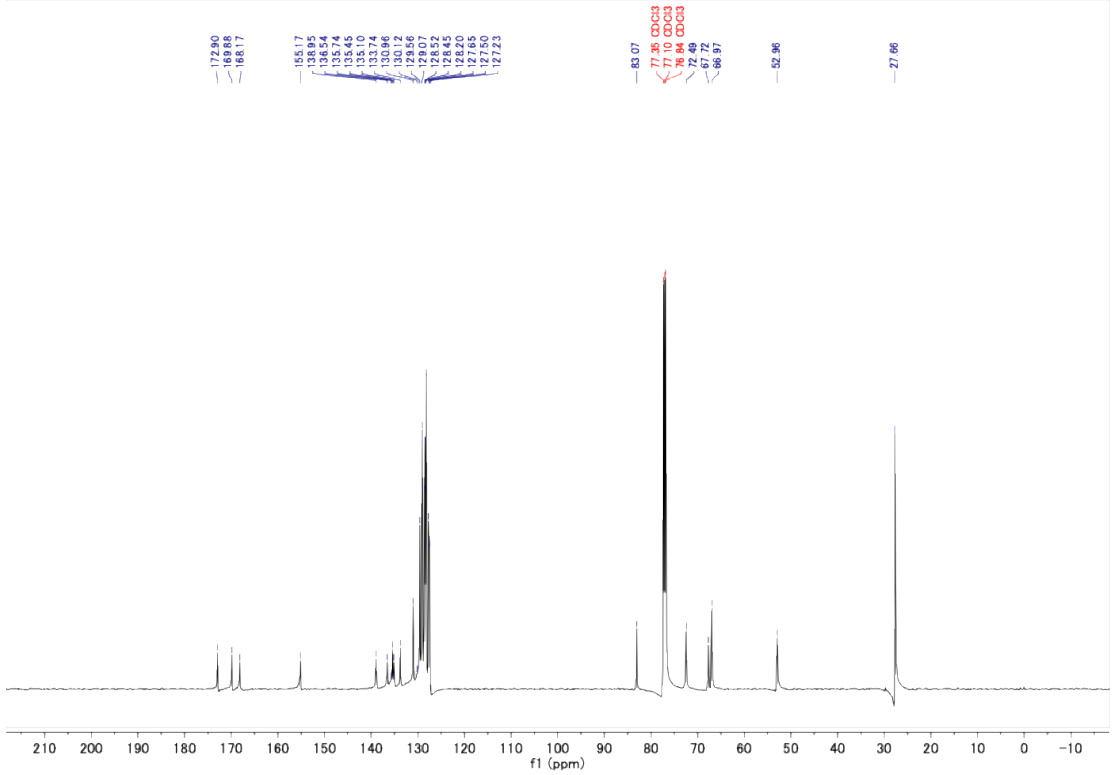

3e ^{1}H NMR ($CDCl_3$, 700 MHz)

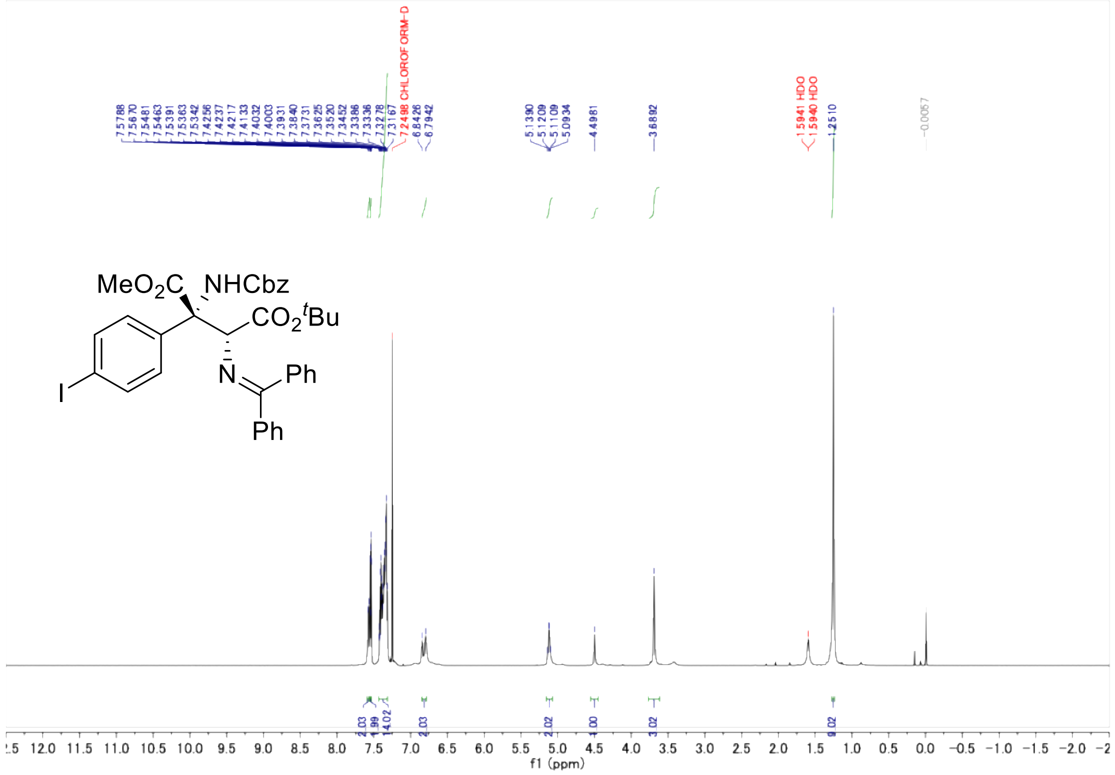

3e ^{13}C NMR ($CDCl_3$, 176 MHz)

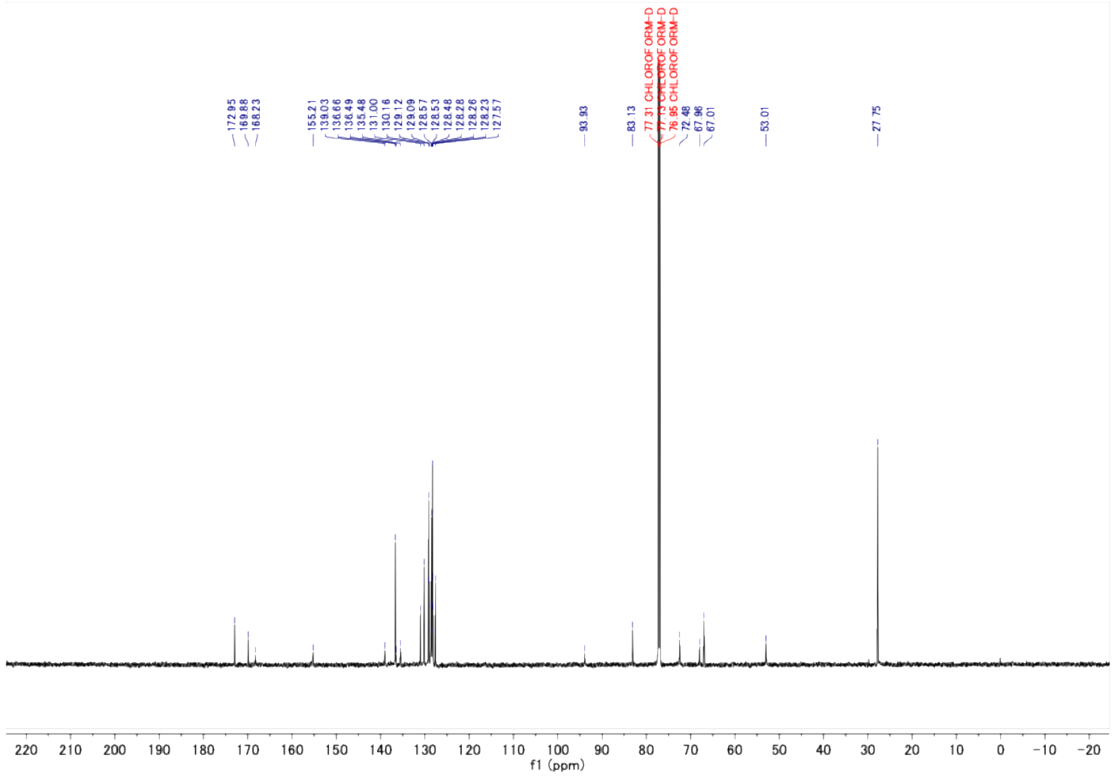

3f ^{1}H NMR ($CDCl_3$, 300 MHz)

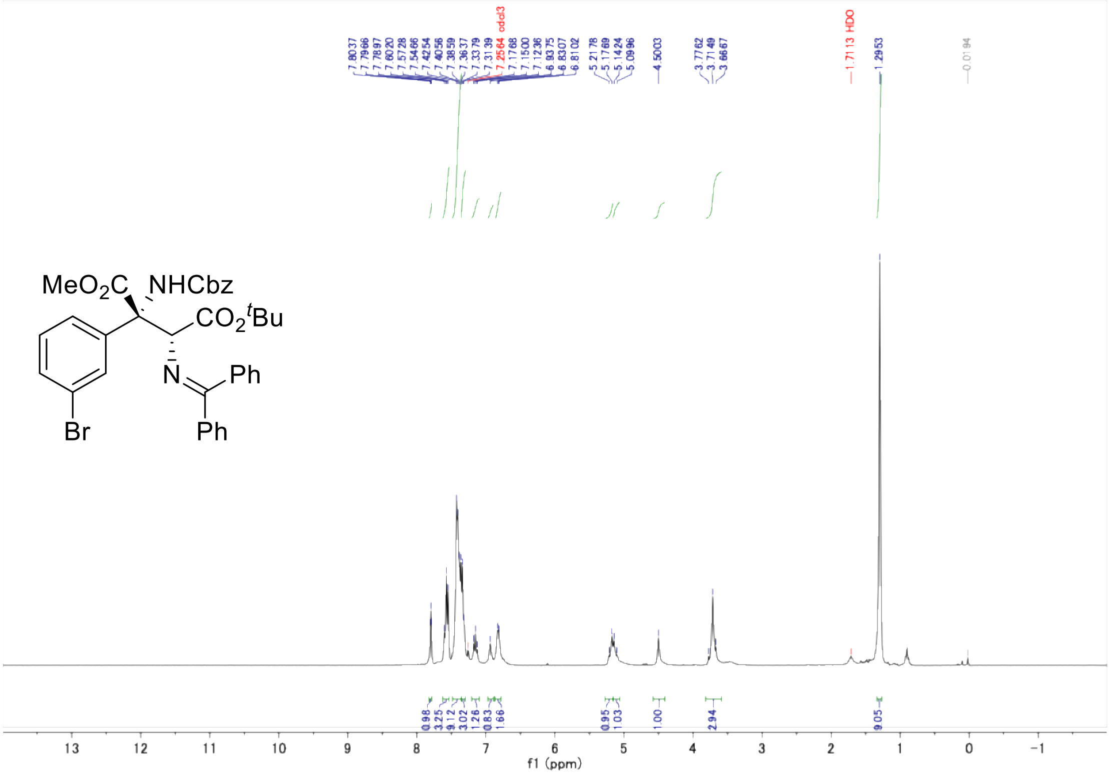

3f ^{13}C NMR ($CDCl_3$, 125 MHz)

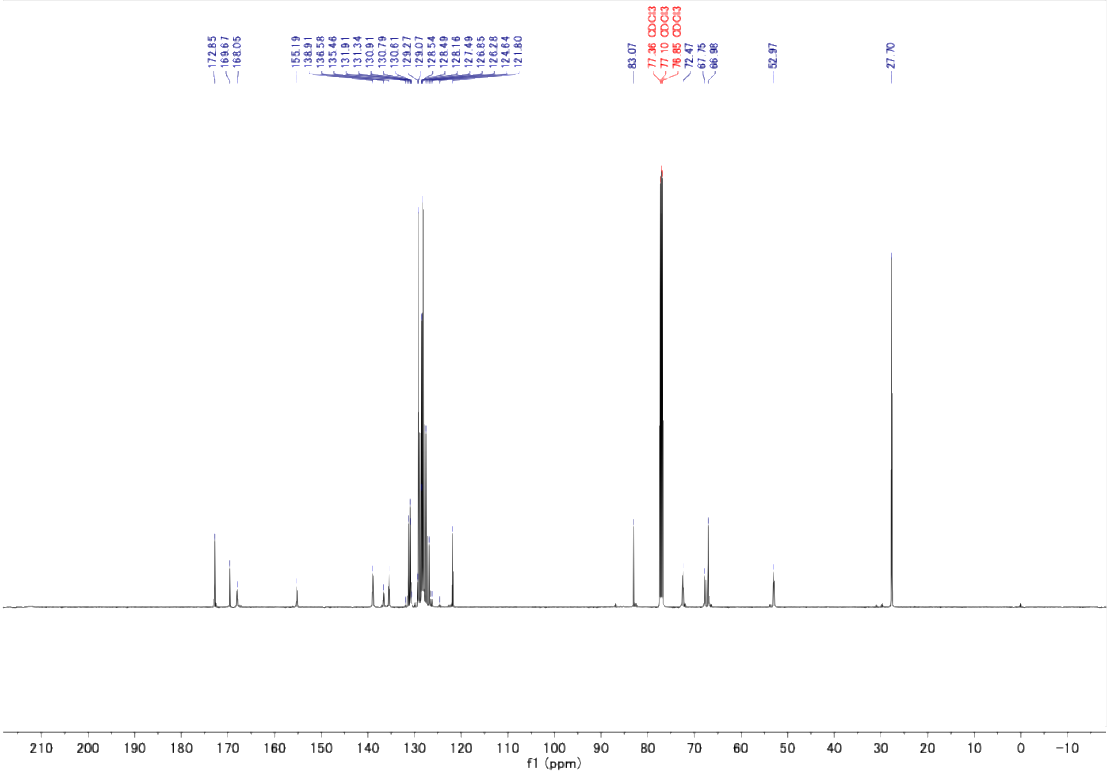

3g ^{1}H NMR ($CDCl_3$, 300 MHz)

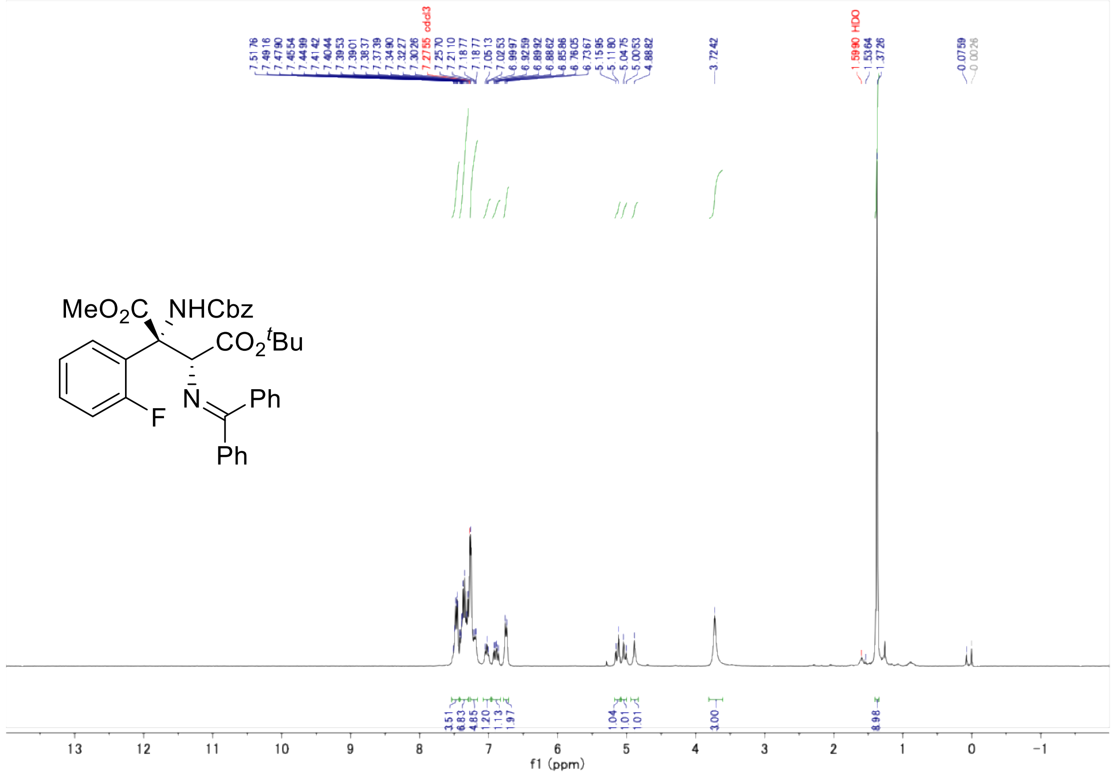

3g ^{13}C NMR ($CDCl_3$, 125 MHz)

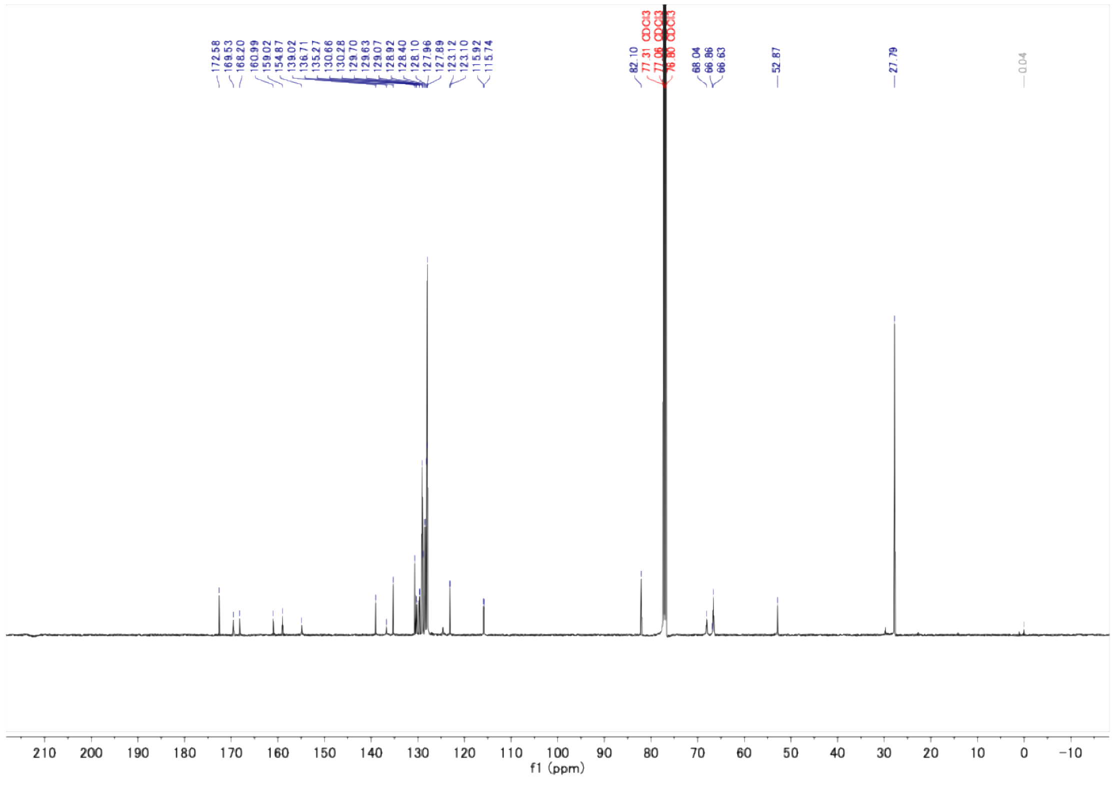

3g ^{19}F NMR ($CDCl_3$, 282 MHz)

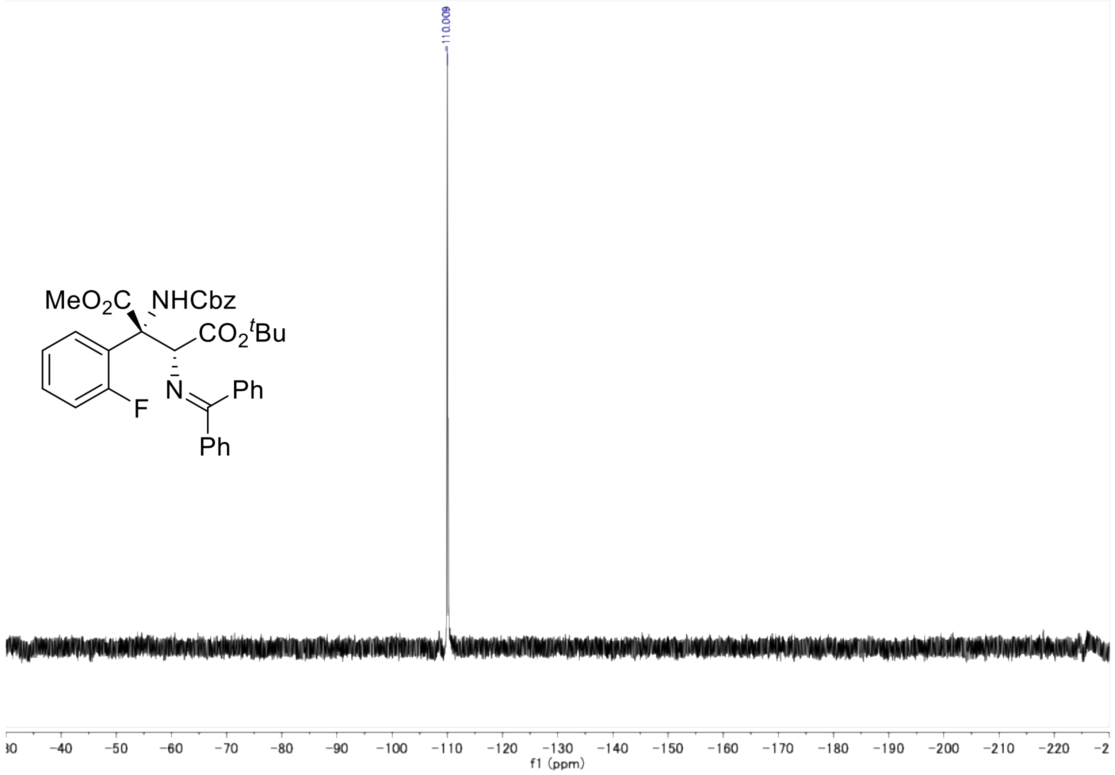

3h ^{1}H NMR ($CDCl_3$, 300 MHz)

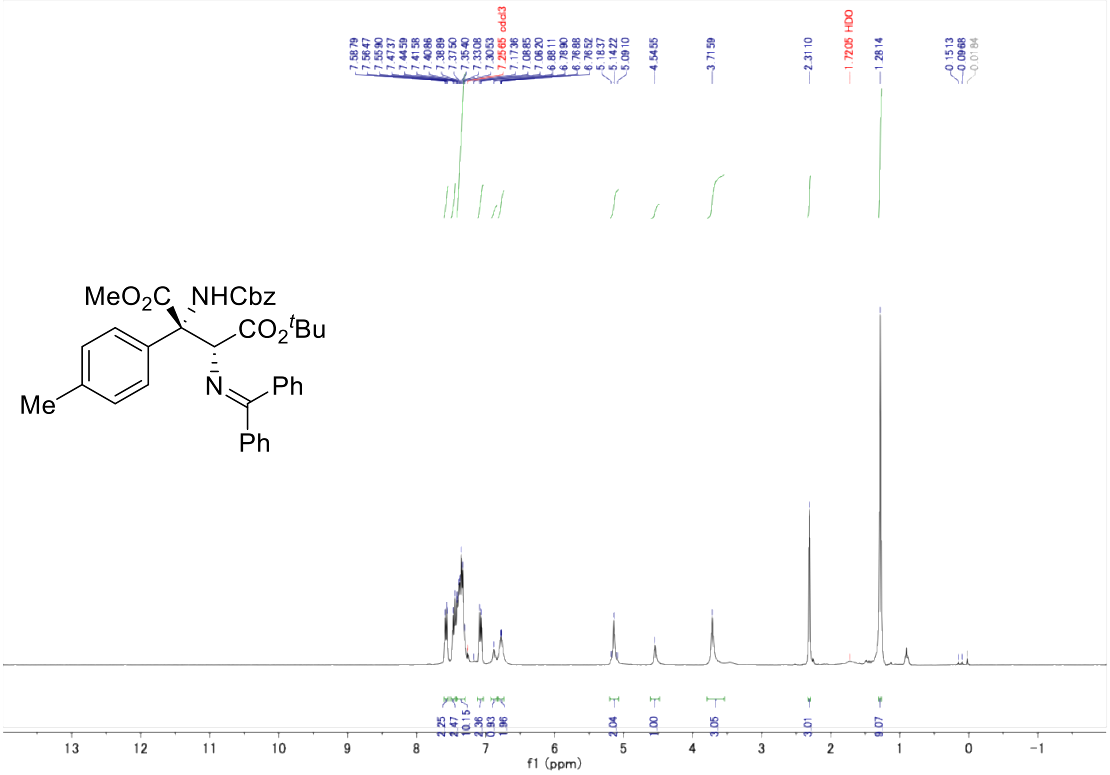

3h ^{13}C NMR ($CDCl_3$, 125 MHz)

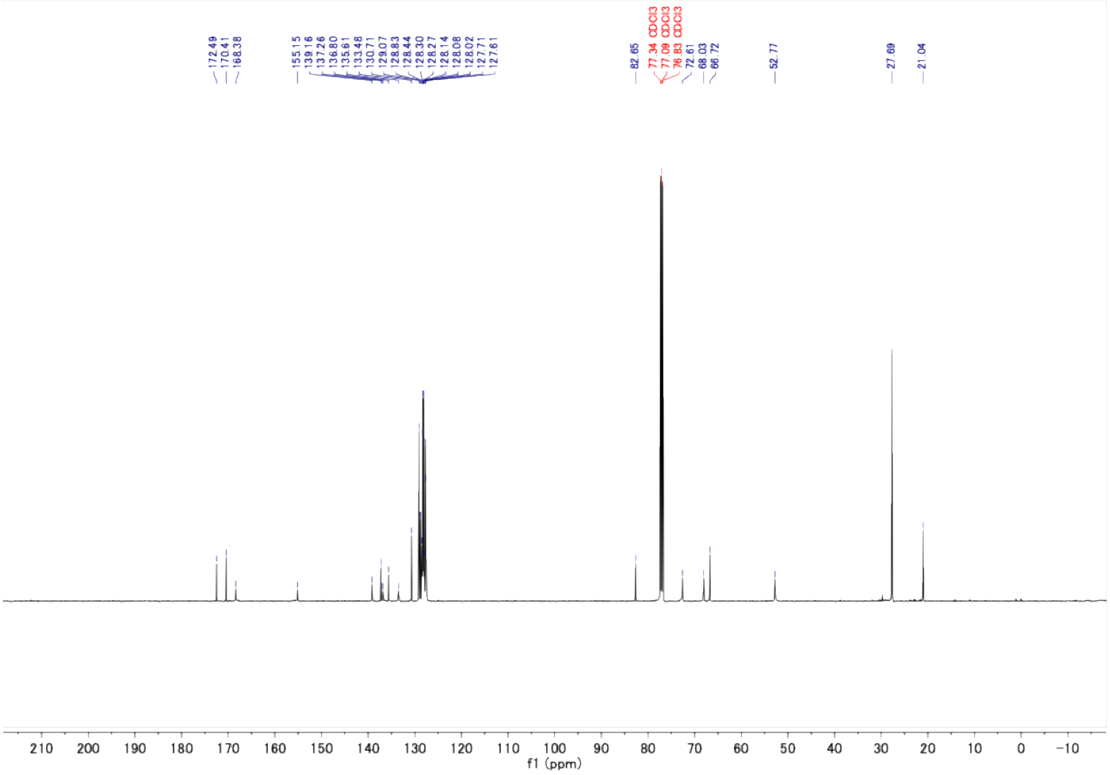

3i ^{1}H NMR ($CDCl_3$, 300 MHz)

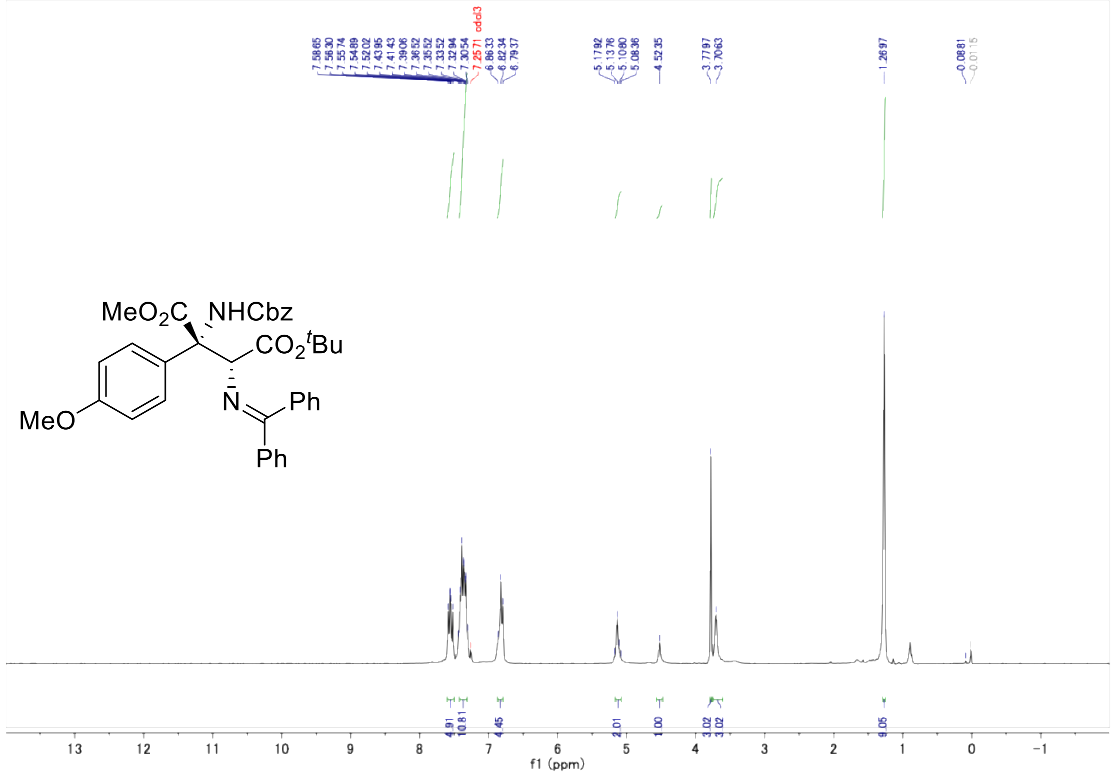

3i ^{13}C NMR ($CDCl_3$, 125 MHz)

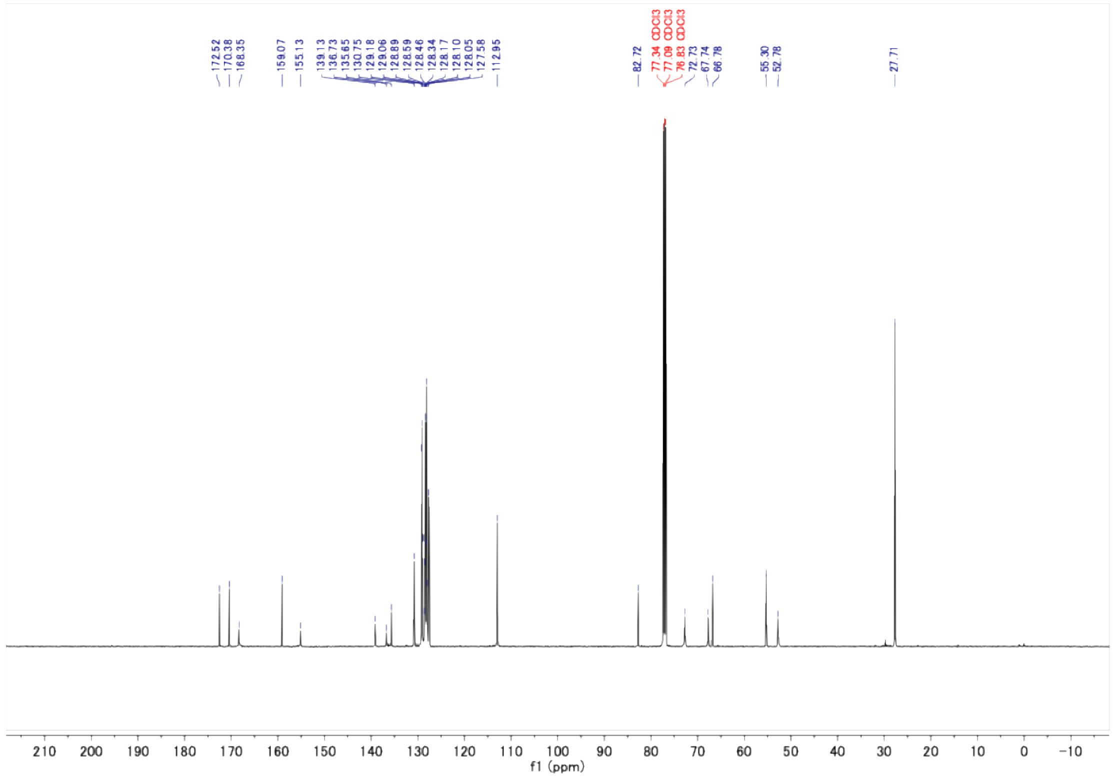

3j ^{1}H NMR ($CDCl_3$, 300 MHz)

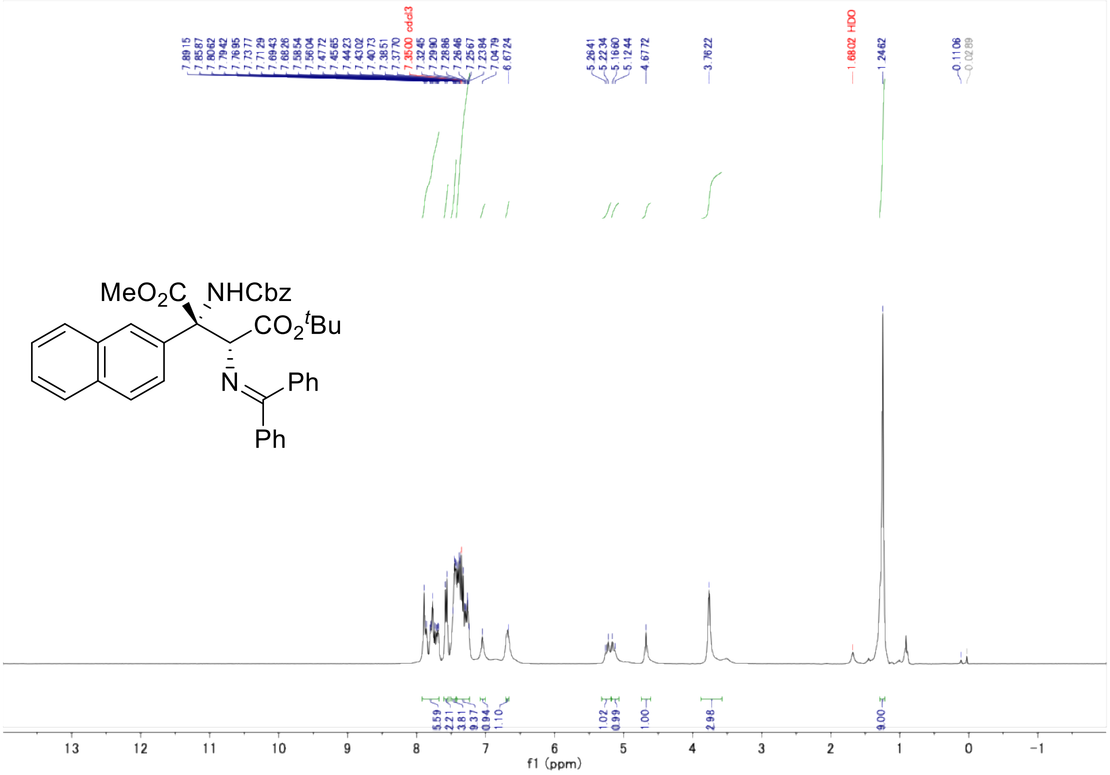

3j ^{13}C NMR ($CDCl_3$, 125 MHz)

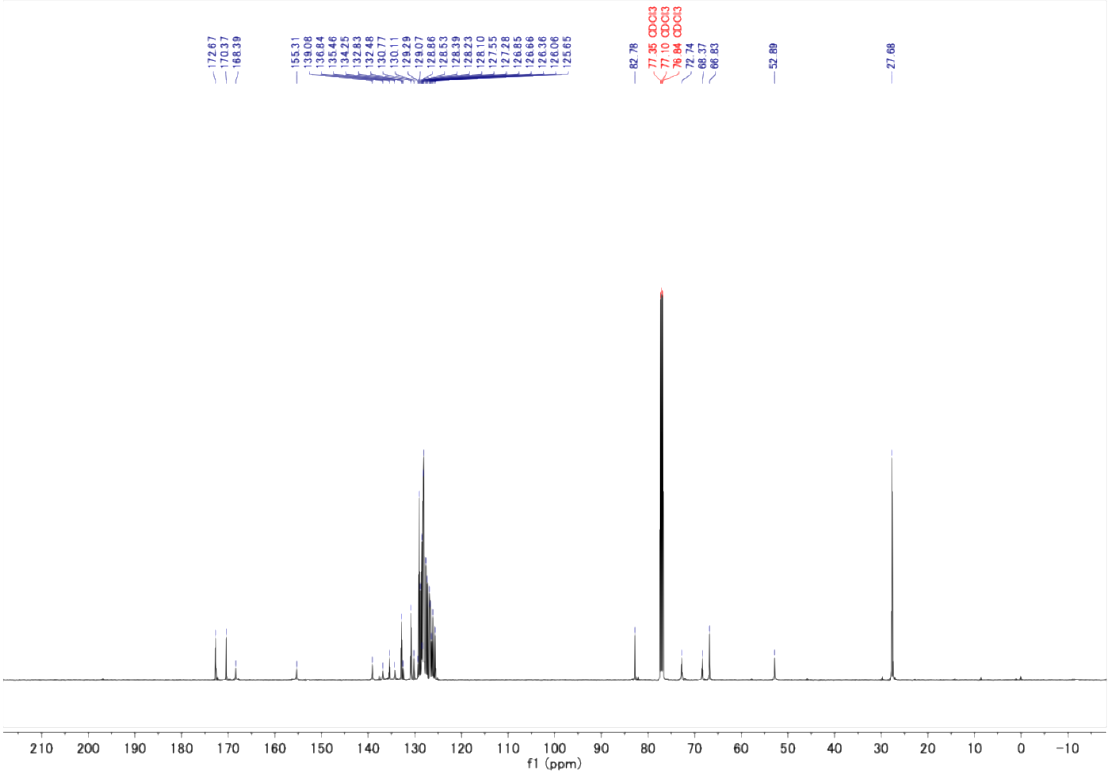

3k ^{1}H NMR ($CDCl_3$, 300 MHz)

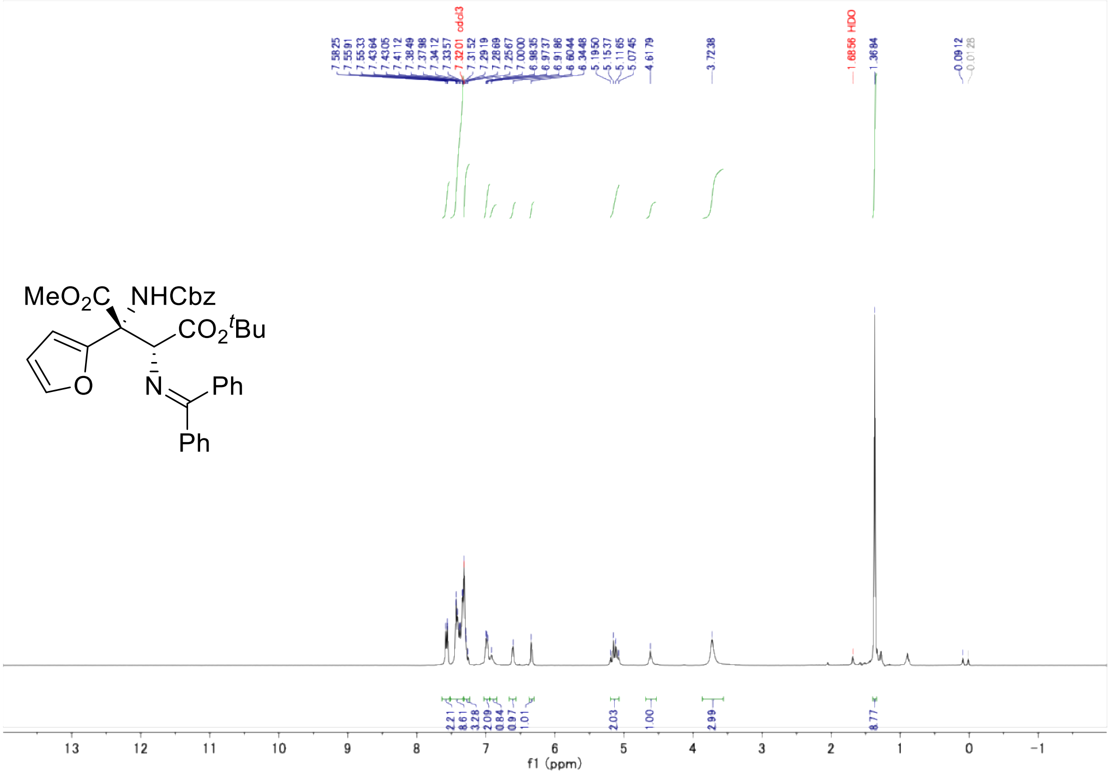

3k ^{13}C NMR ($CDCl_3$, 125 MHz)

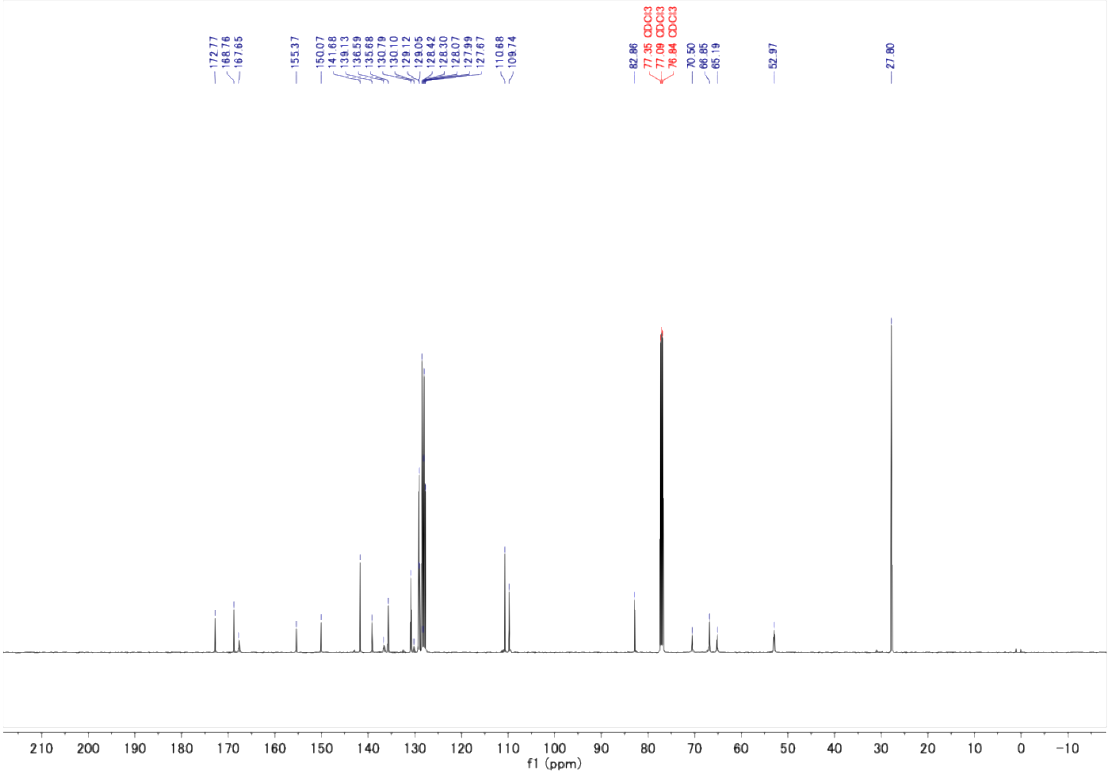

3l ^{1}H NMR ($CDCl_3$, 300 MHz)

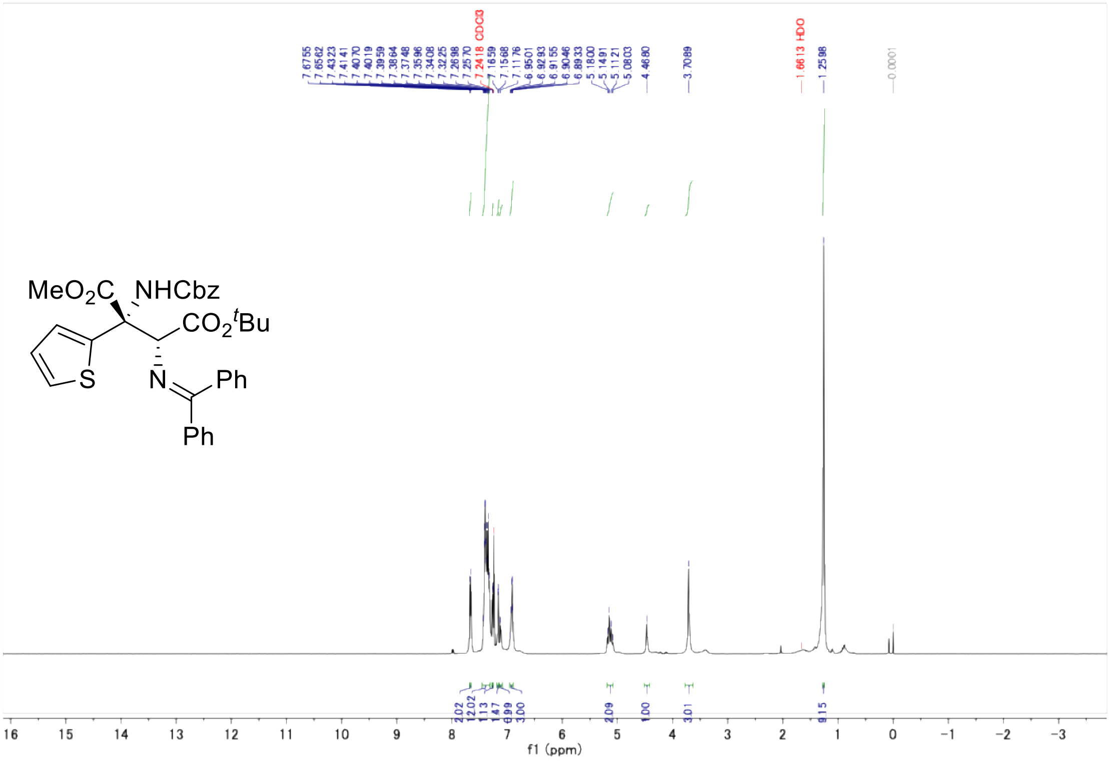

3l ^{13}C NMR ($CDCl_3$, 125 MHz)

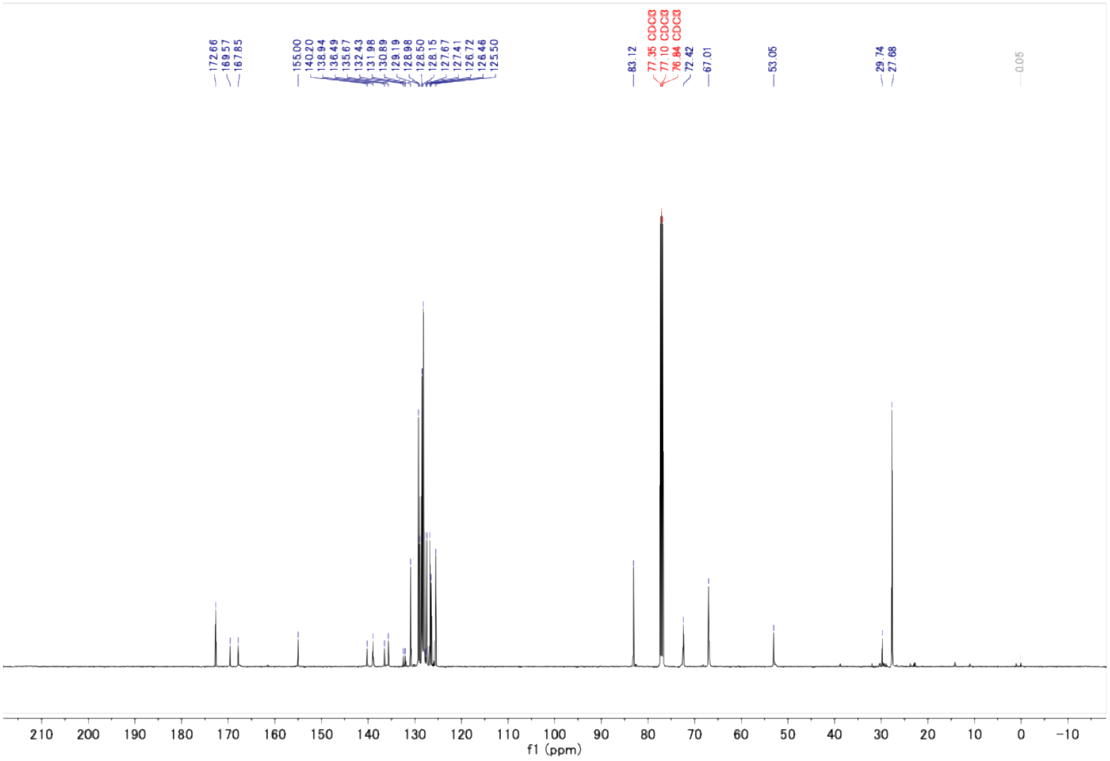

3m ^{1}H NMR ($CDCl_3$, 300 MHz)

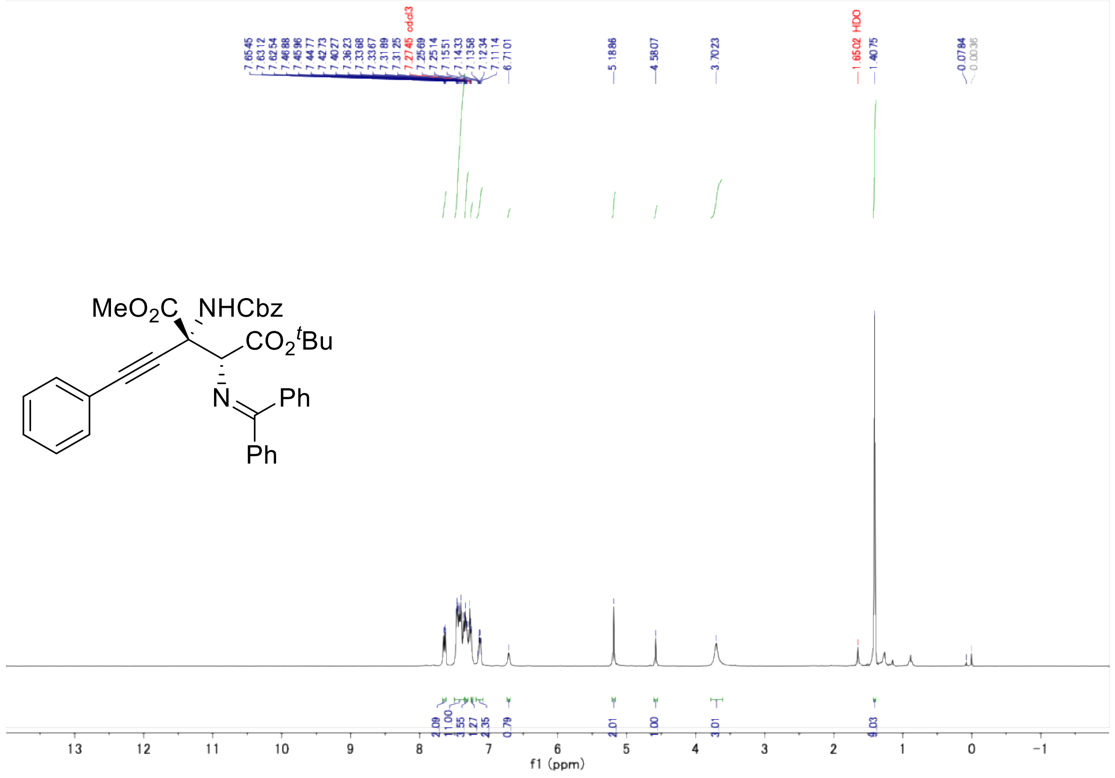

3m ^{13}C NMR ($CDCl_3$, 125 MHz)

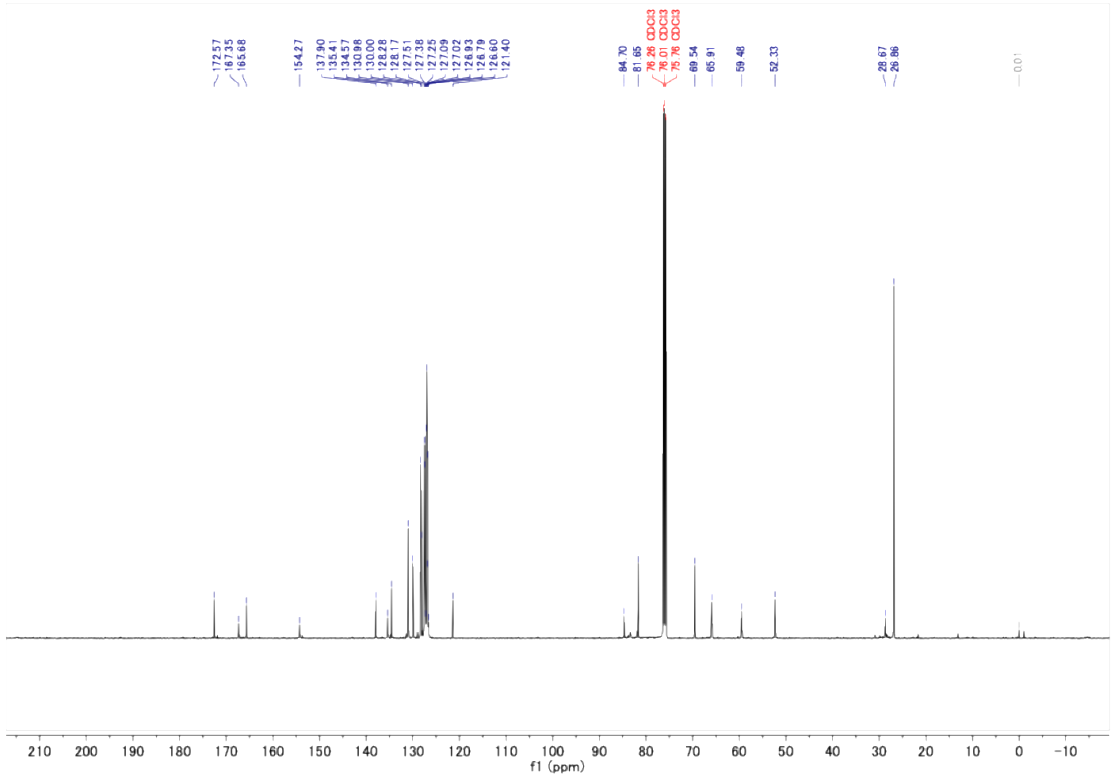

3n ^{1}H NMR ($CDCl_3$, 700 MHz)

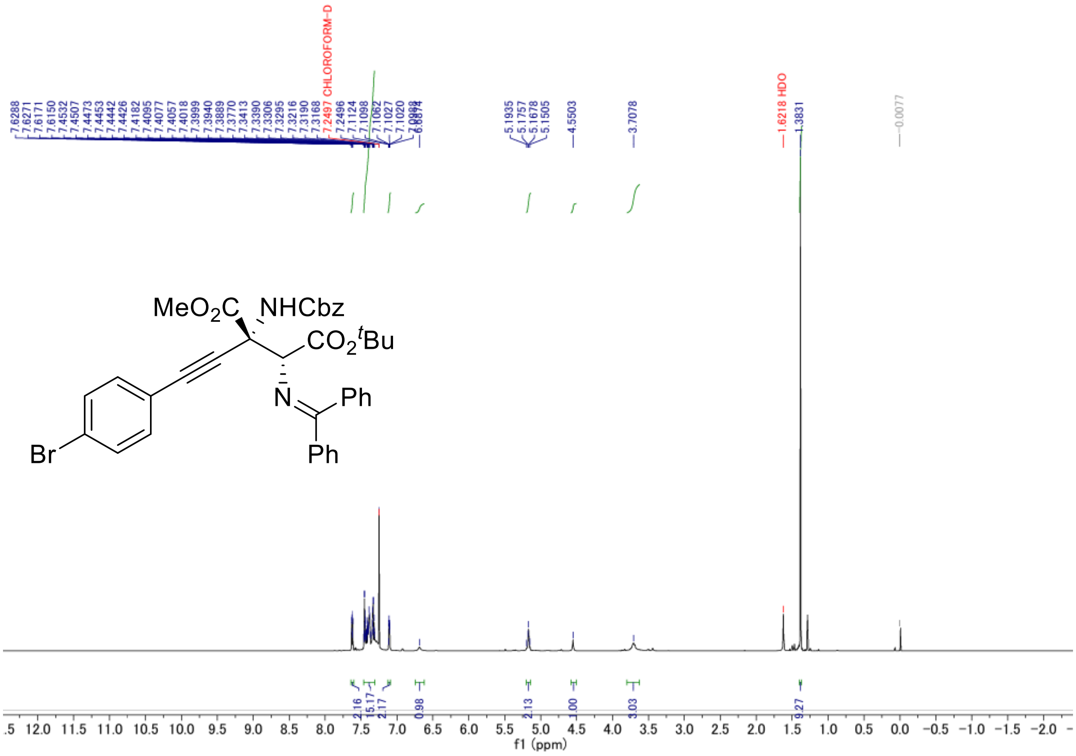

3n ^{13}C NMR ($CDCl_3$, 176 MHz)

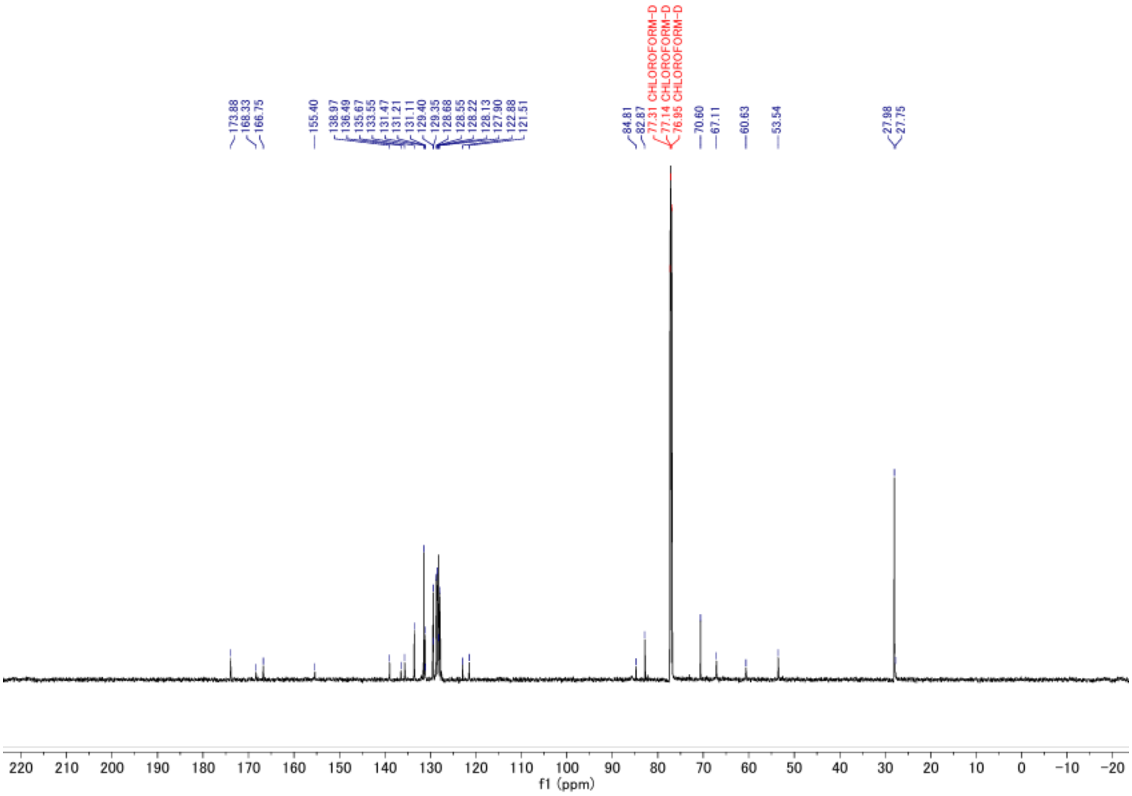

3o ^{1}H NMR ($CDCl_3$, 400 MHz)

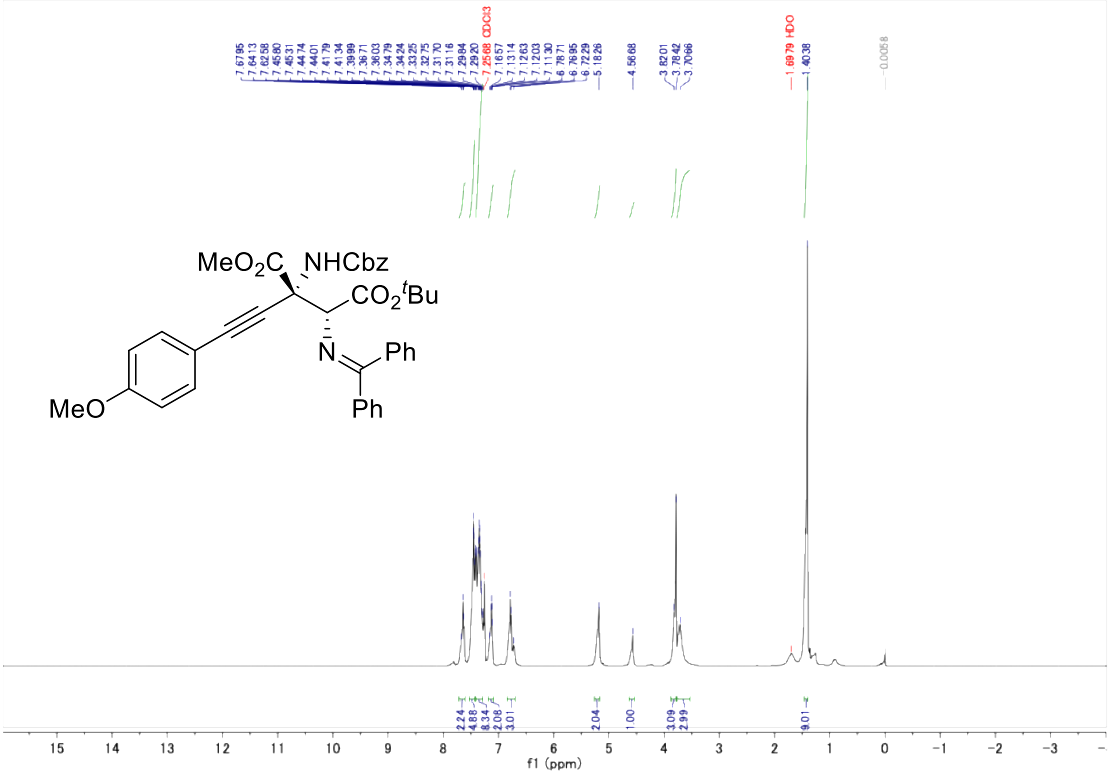

3o ^{13}C NMR ($CDCl_3$, 125 MHz)

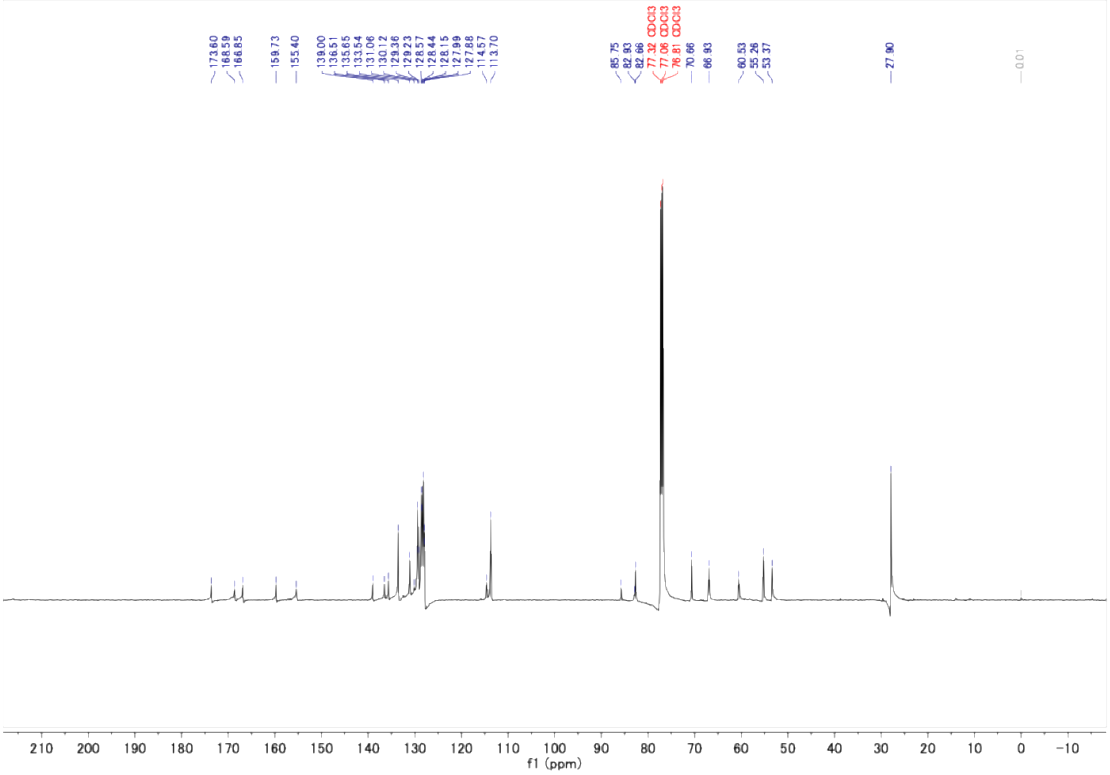

3p ^{1}H NMR ($CDCl_3$, 300 MHz)

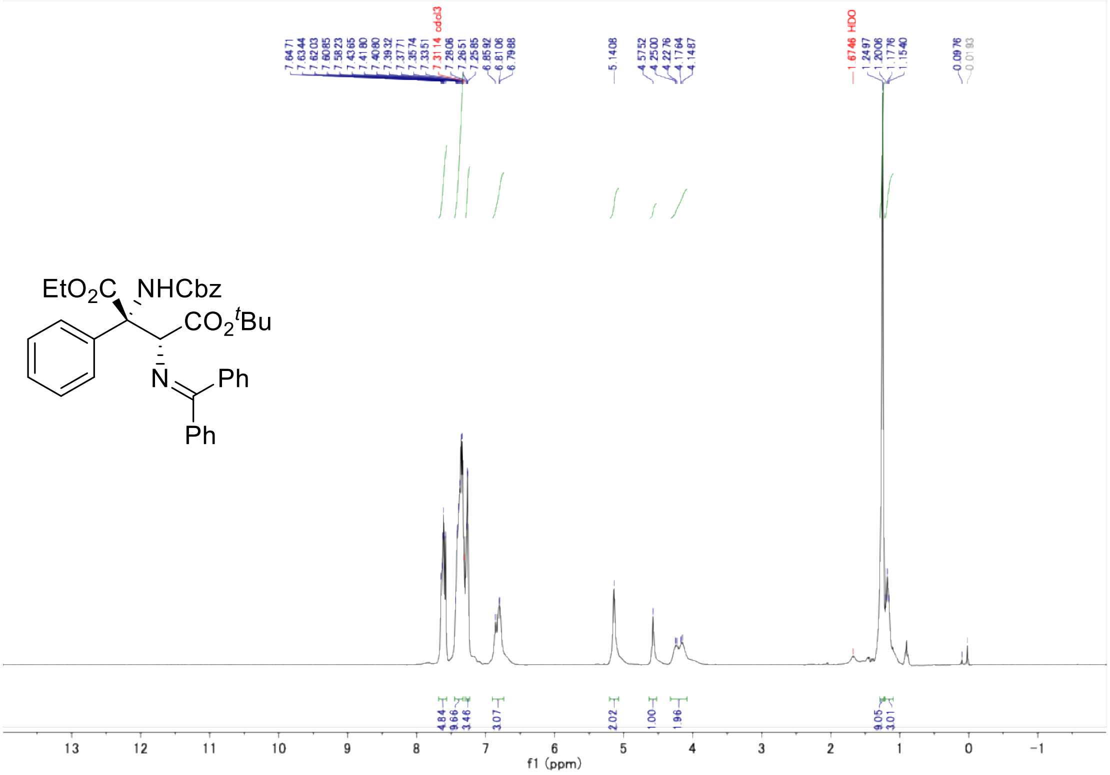

3p ^{13}C NMR ($CDCl_3$, 125 MHz)

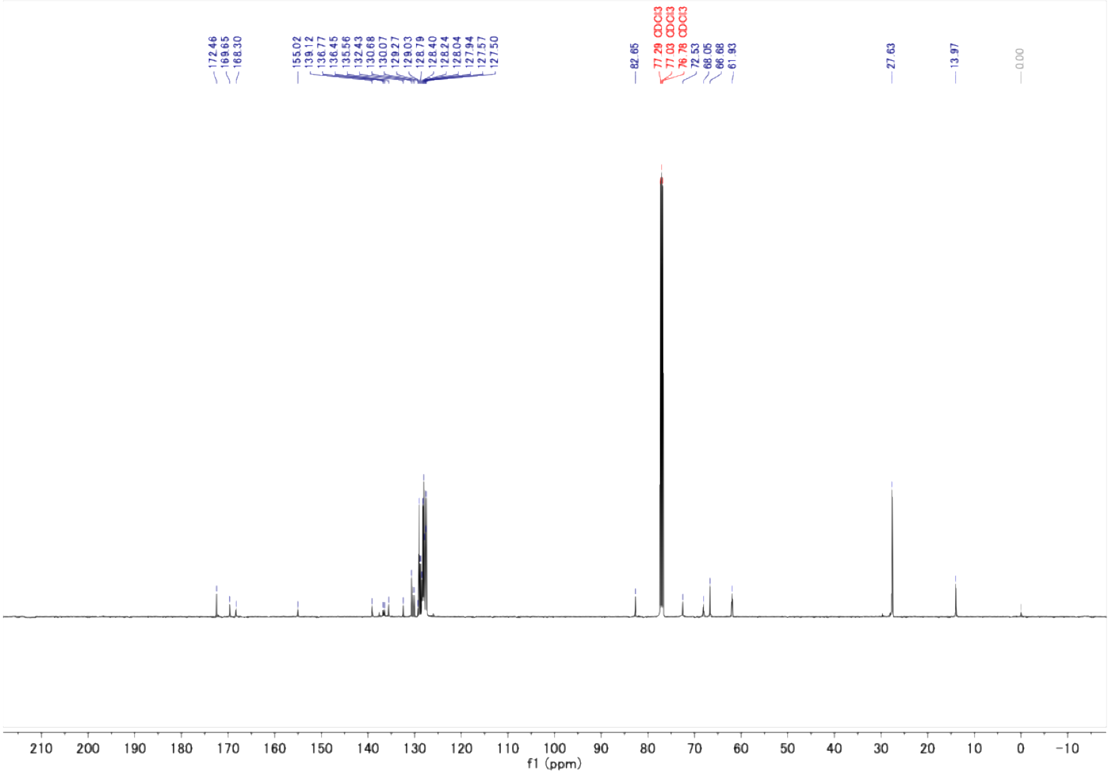

3q ^{1}H NMR ($CDCl_3$, 300 MHz)

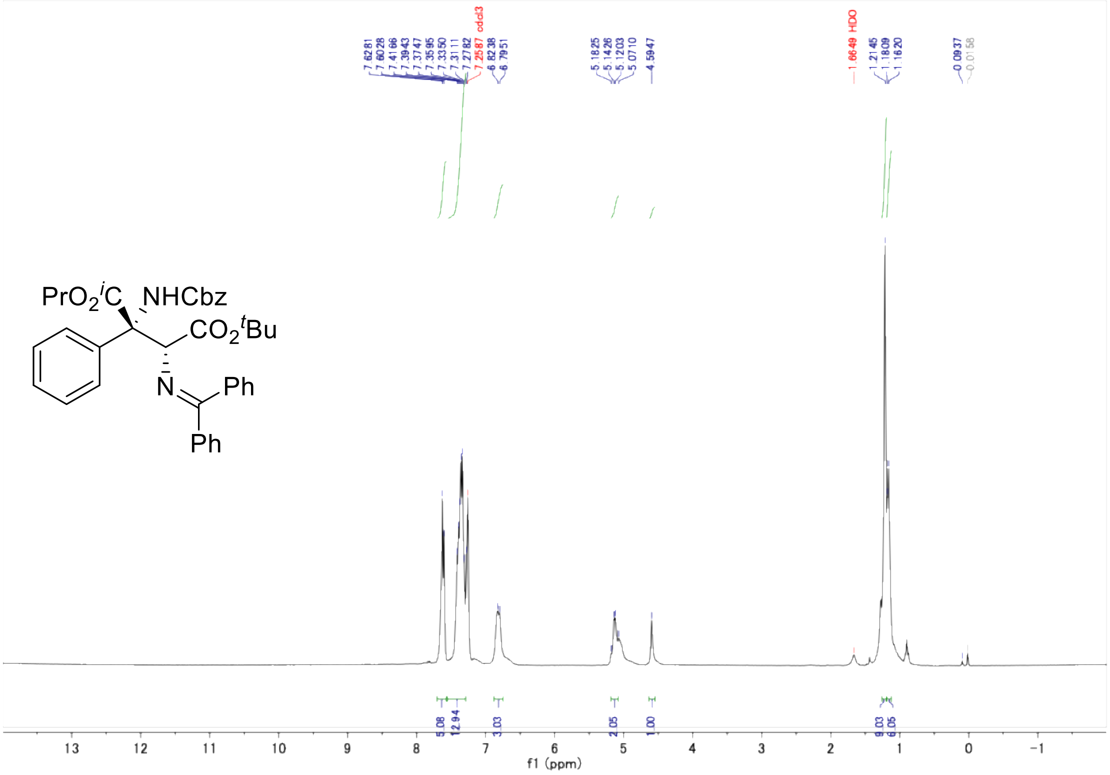

3q ^{13}C NMR ($CDCl_3$, 125 MHz)

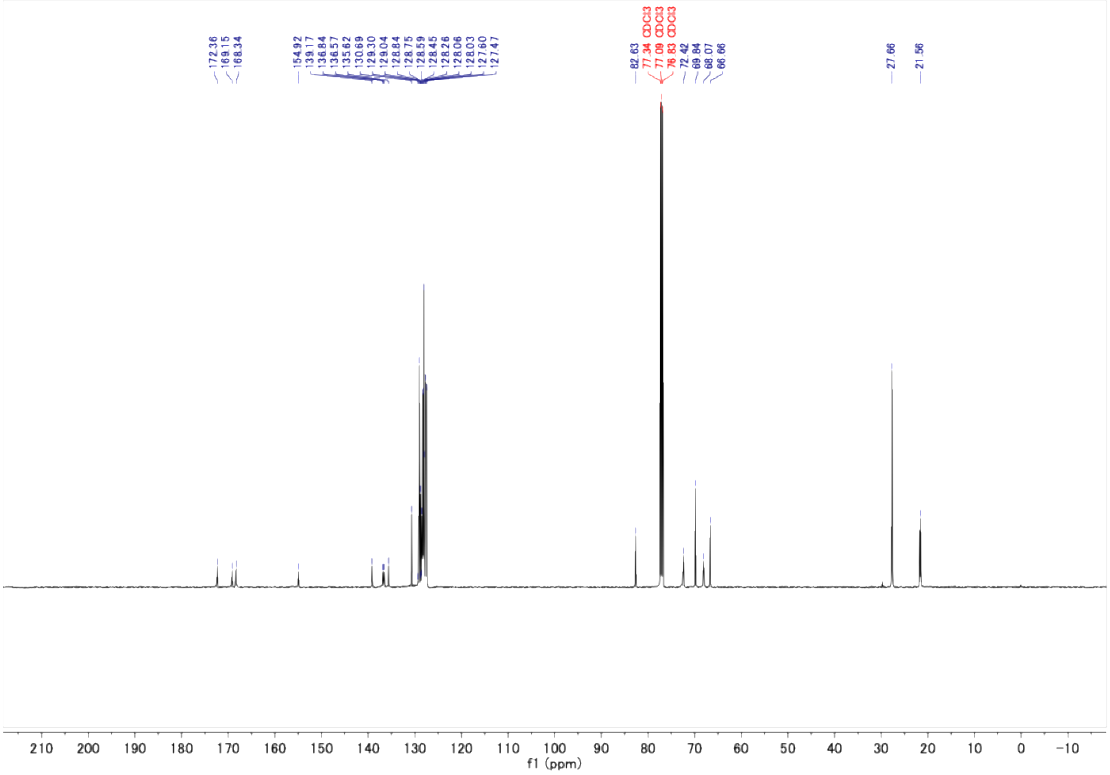

3r ^{1}H NMR ($CDCl_3$, 300 MHz)

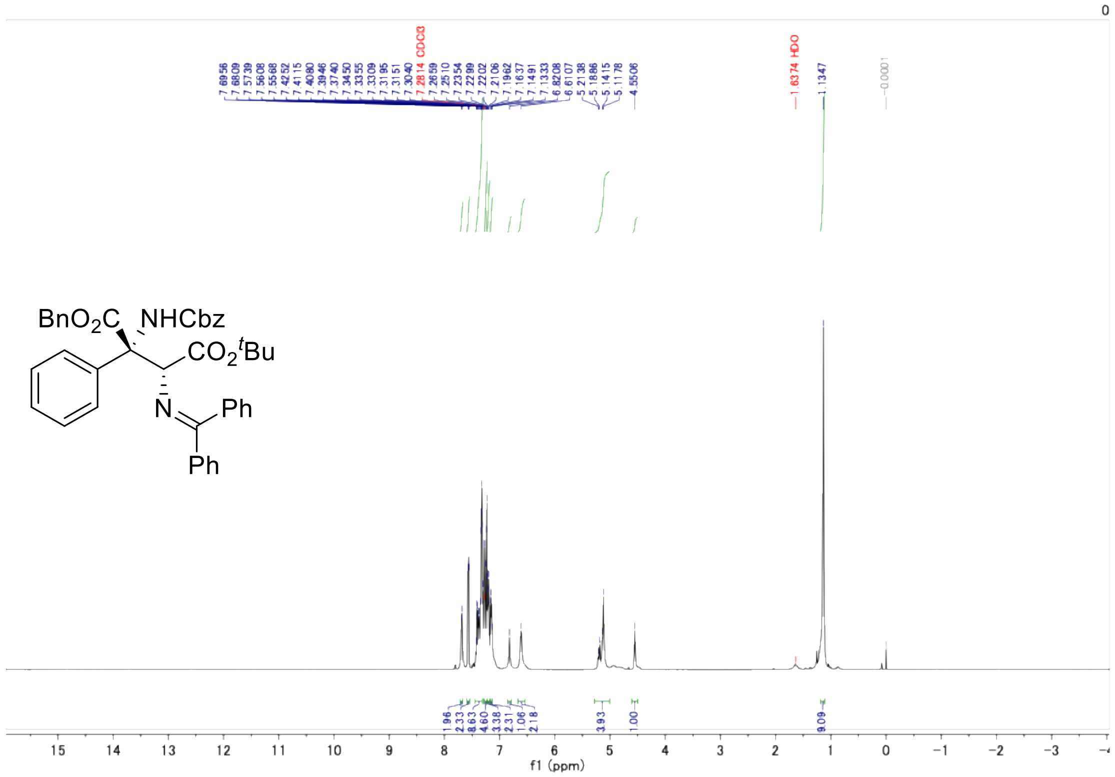

3r ^{13}C NMR ($CDCl_3$, 125 MHz)

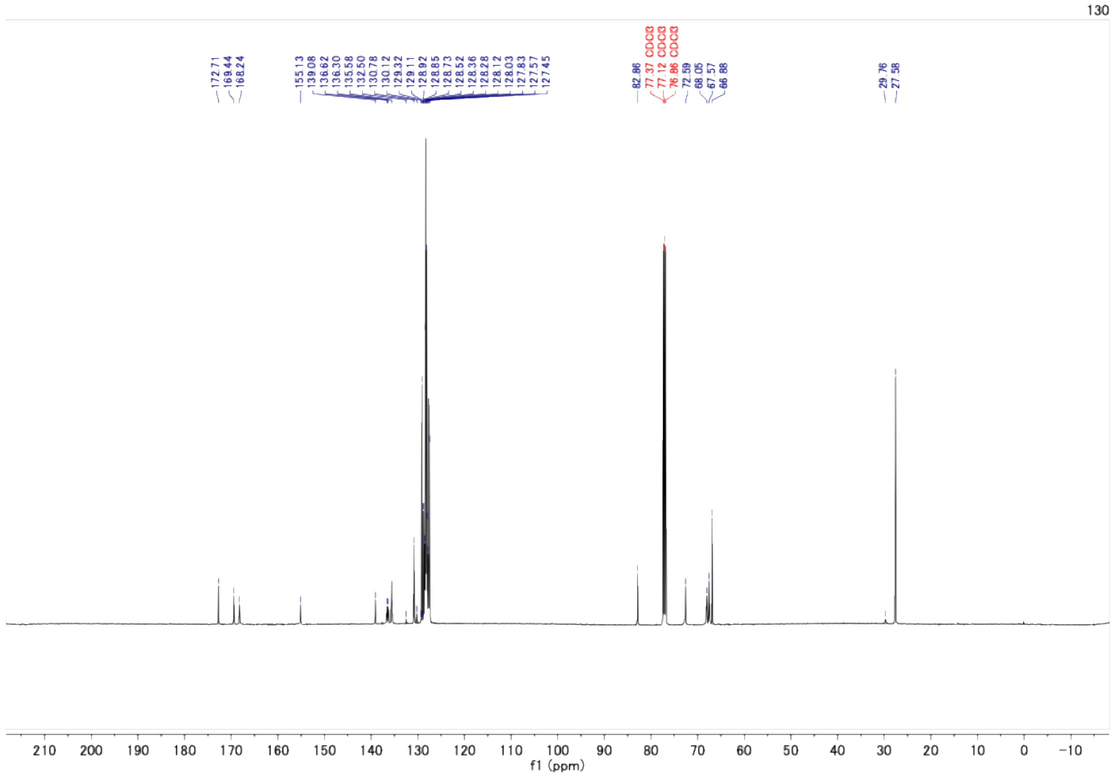

3s ^{1}H NMR ($CDCl_3$, 400 MHz)

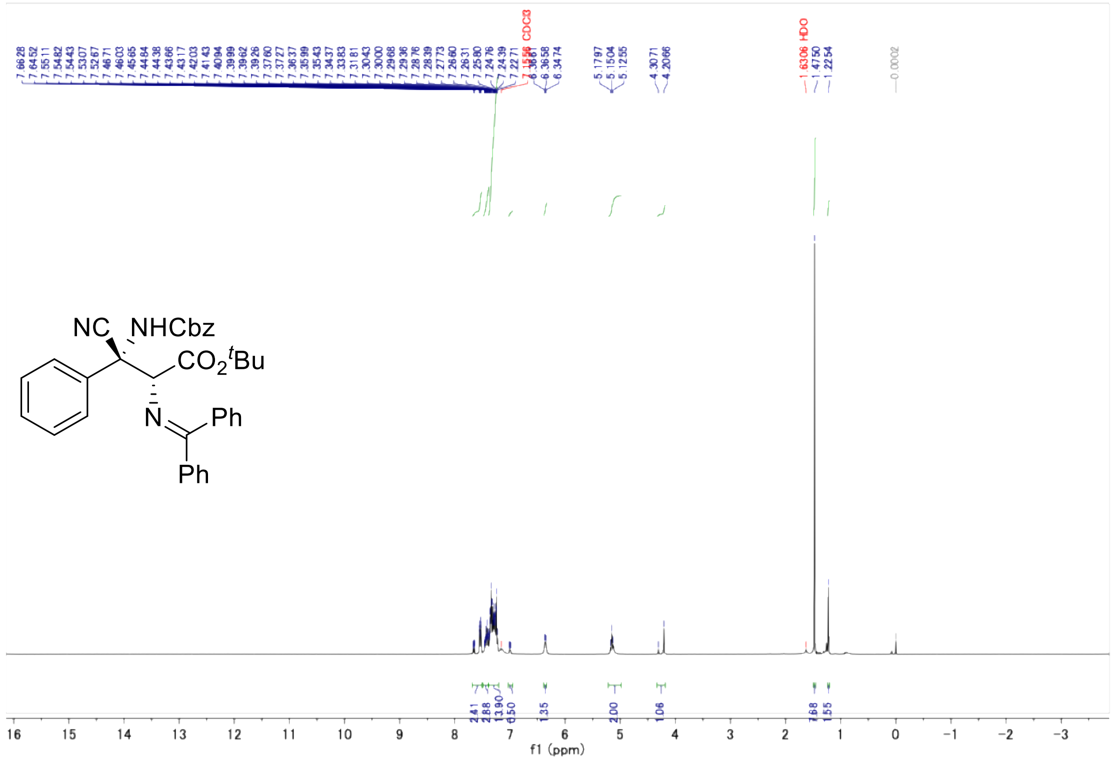

3s ^{13}C NMR ($CDCl_3$, 125 MHz)

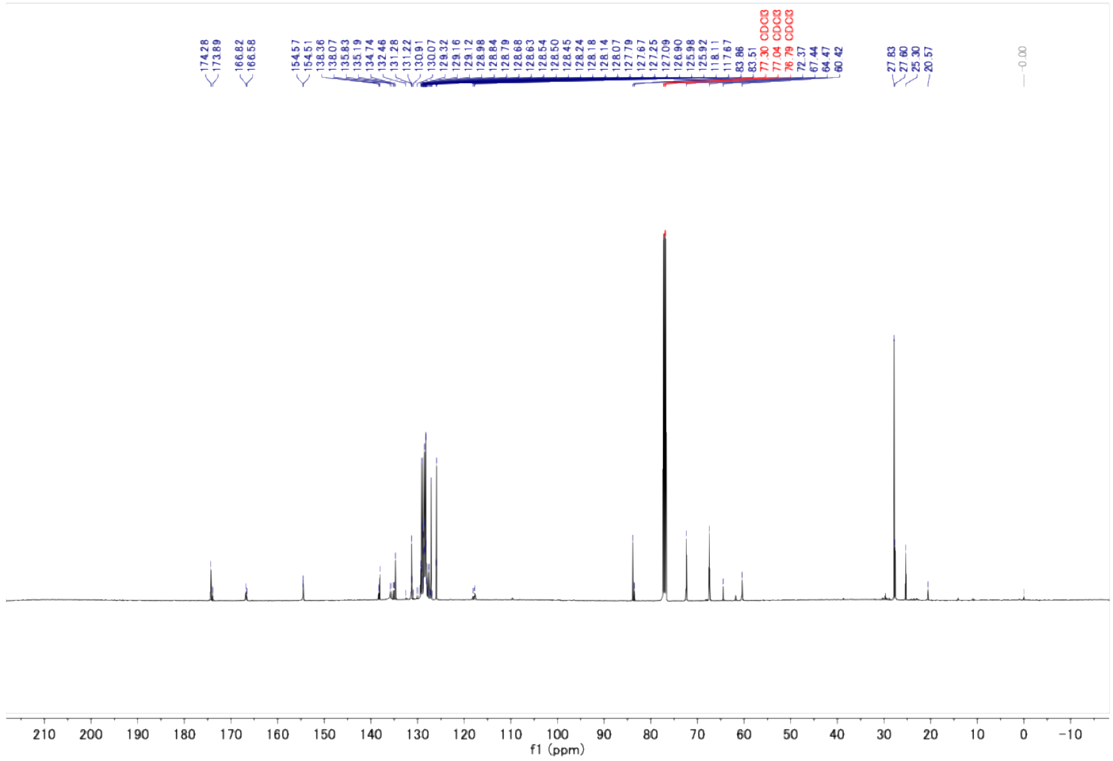

3t ^{1}H NMR ($CDCl_3$, 400 MHz)

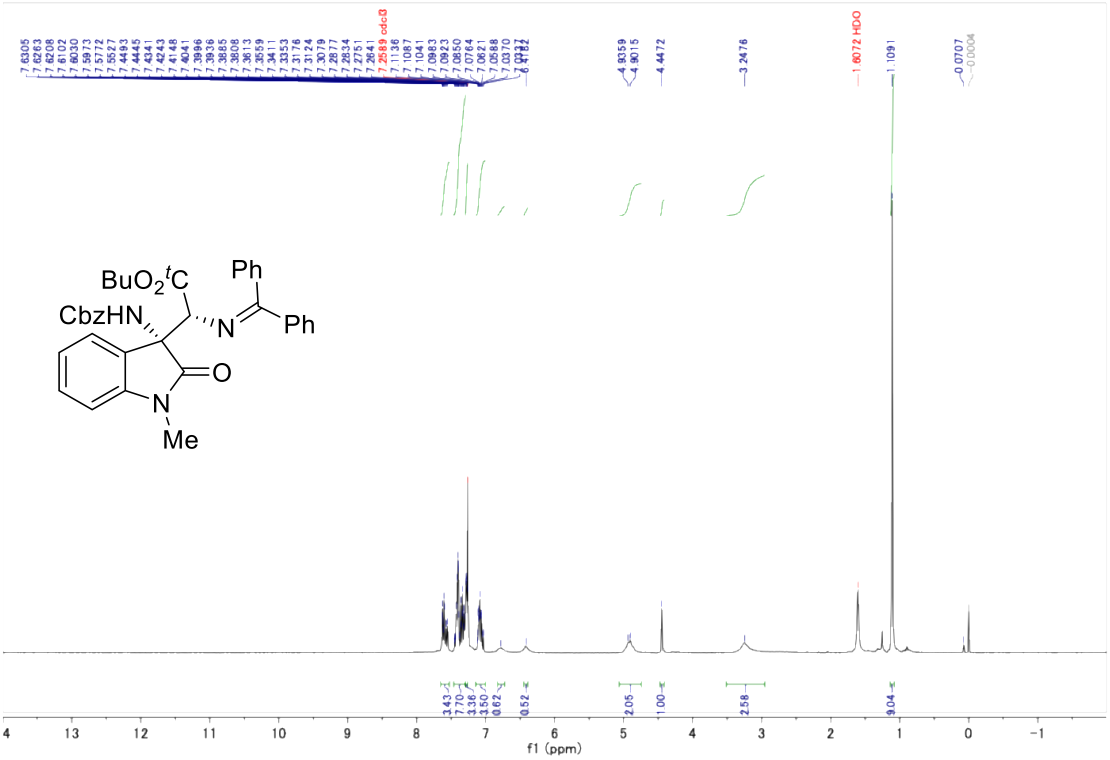

3t ^{13}C NMR ($CDCl_3$, 125 MHz)

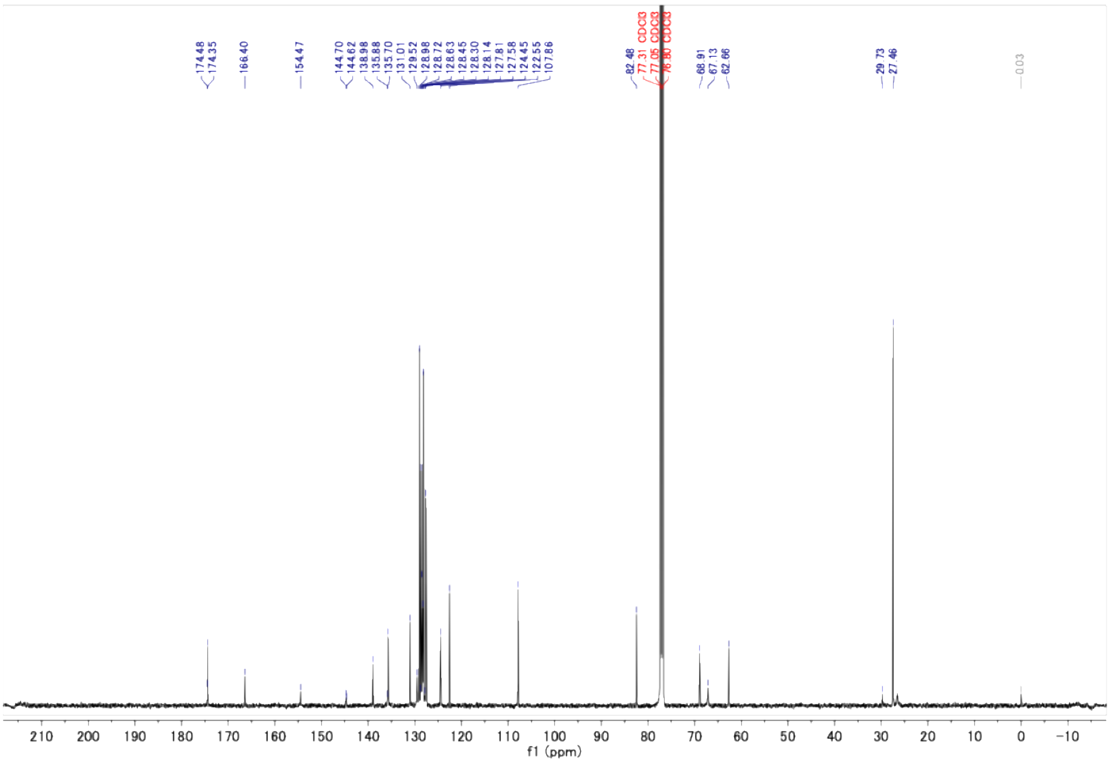

3u ^{1}H NMR ($CDCl_3$, 500 MHz)

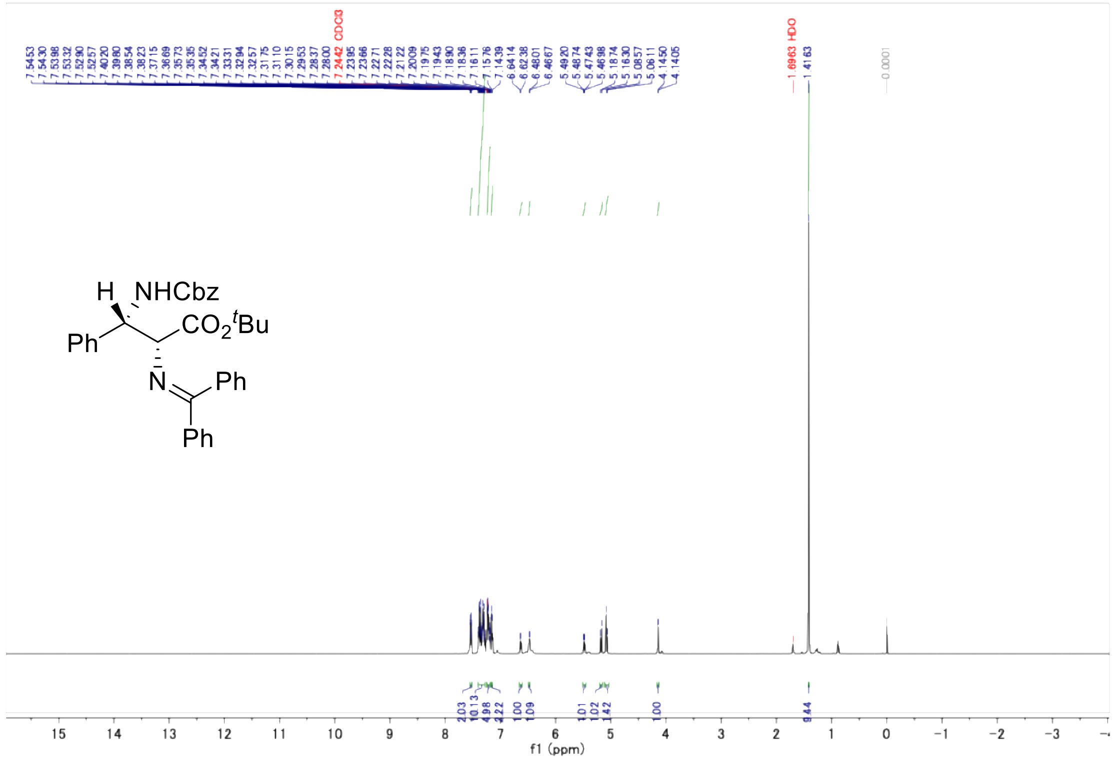

3u ^{13}C NMR ($CDCl_3$, 125 MHz)

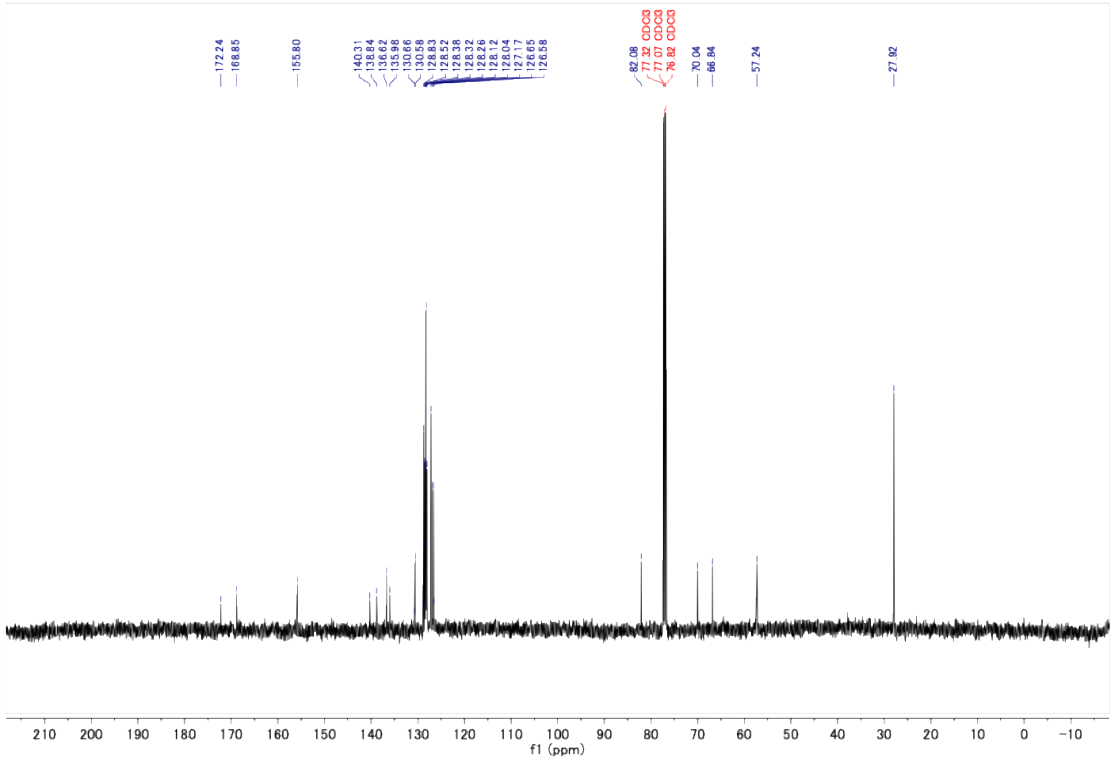

4 ^{1}H NMR ($CDCl_3$, 400 MHz)

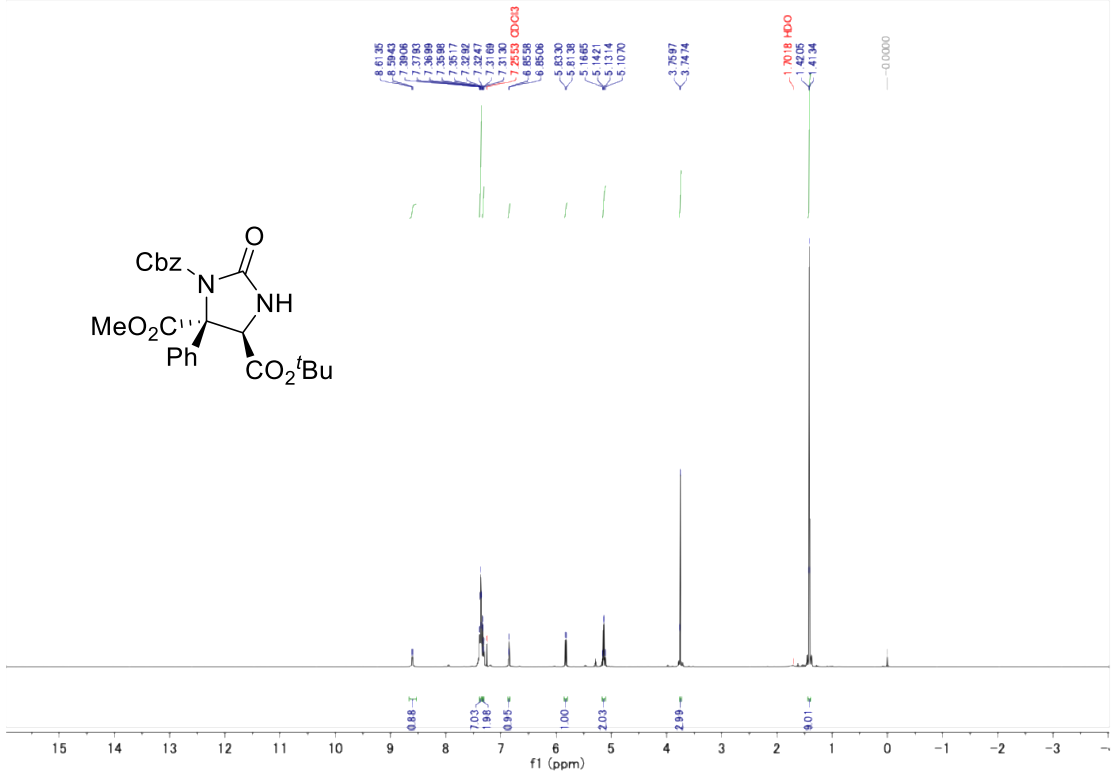

4 ^{13}C NMR ($CDCl_3$, 125 MHz)

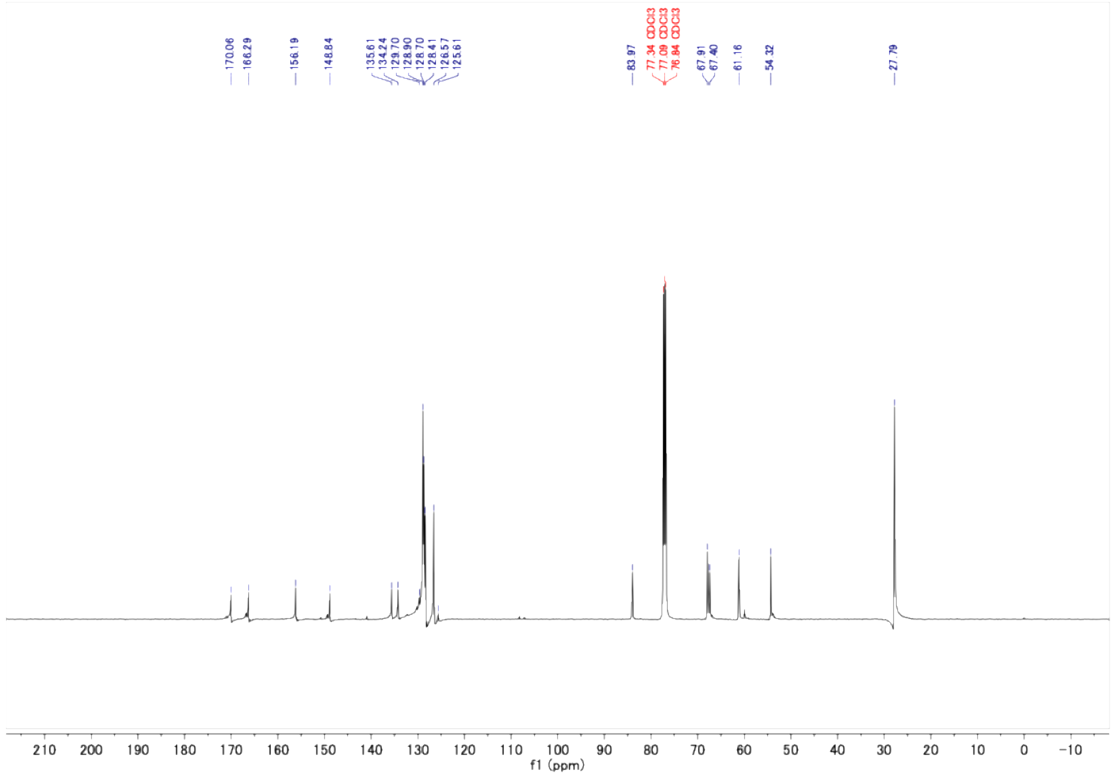

5 ^{1}H NMR ($CDCl_3$, 400 MHz)

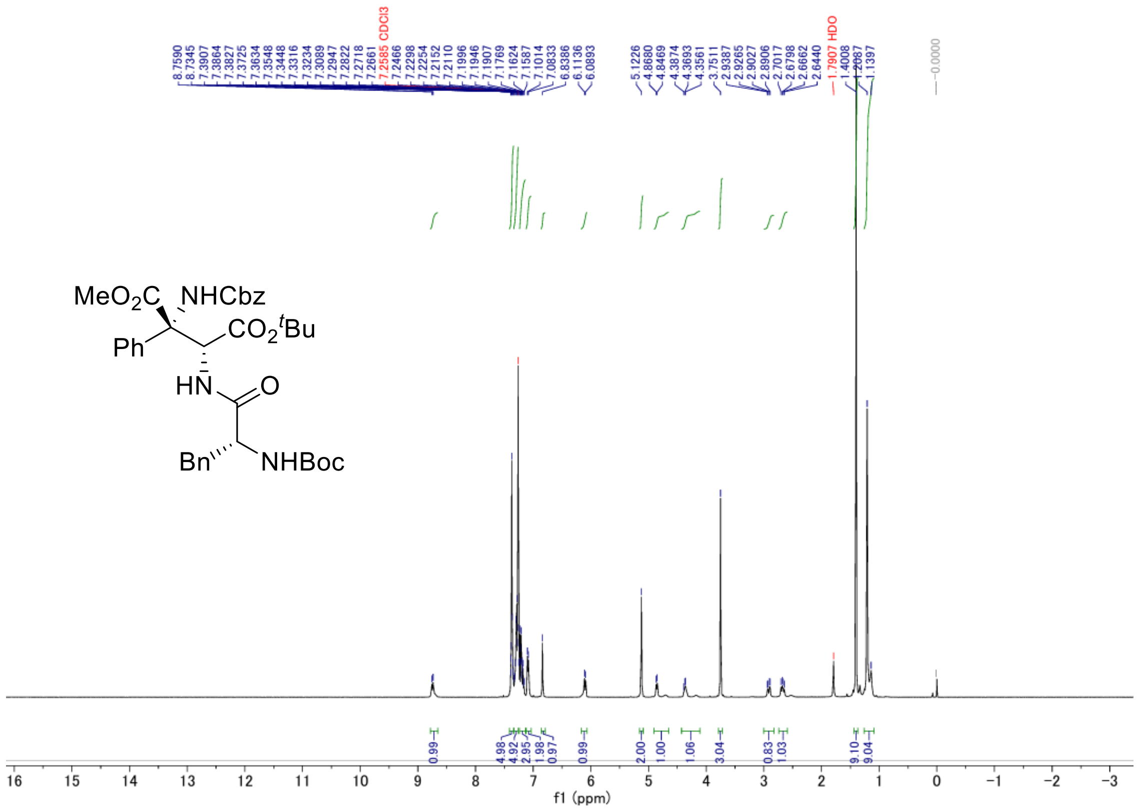

5 ^{13}C NMR ($CDCl_3$, 176 MHz)

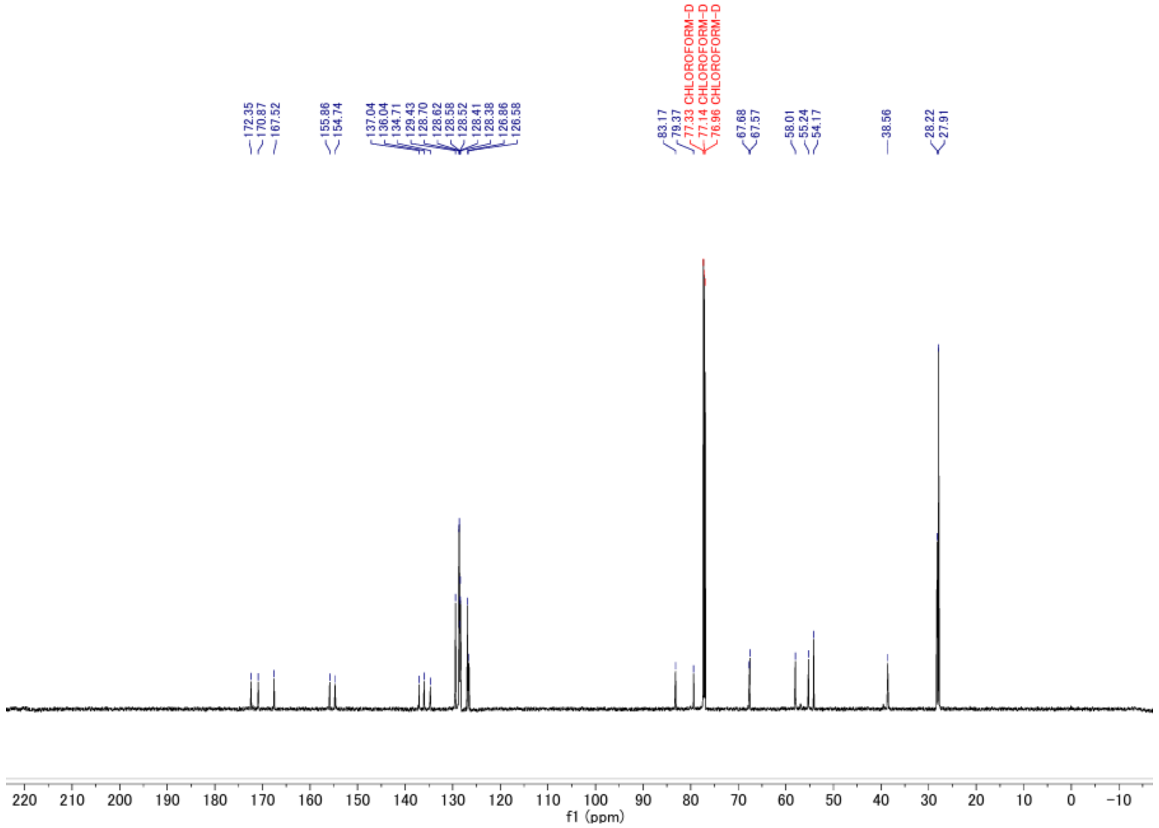

6 ^{1}H NMR (CD_3OD, 400 MHz)

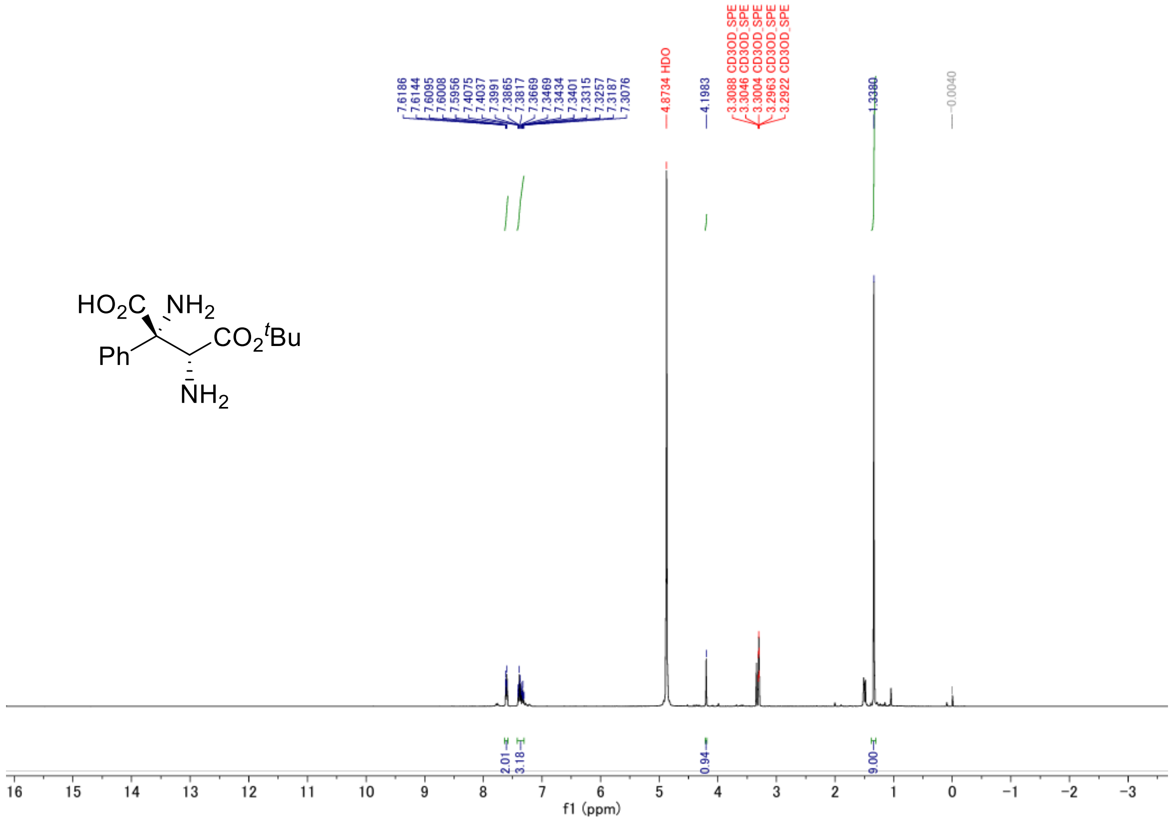

6 ^{13}C NMR (CD_3OD, 176 MHz)

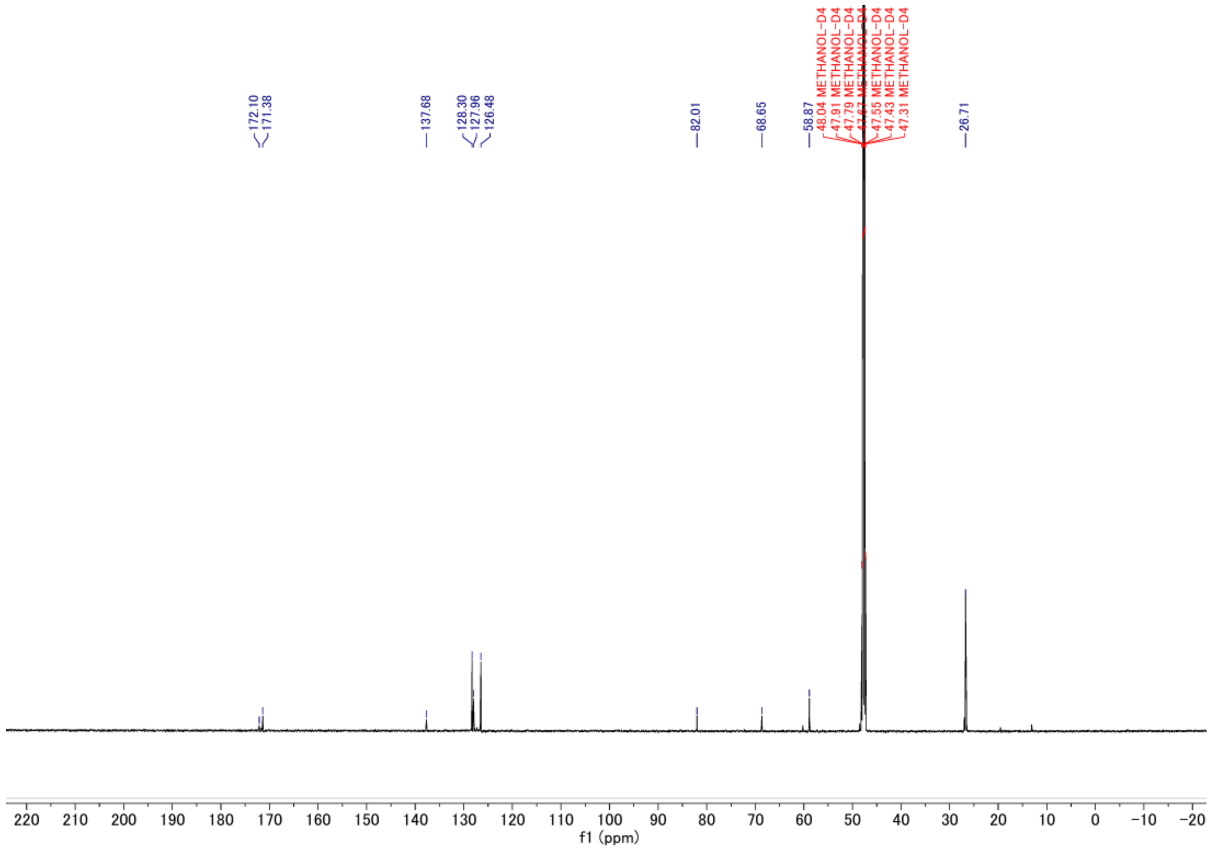

Supplement: Supplementary file 1 [file ja5c18426_si_001.pdf]
